# Pyrrolidines and Piperidines by Ligand‐Enabled Aza‐Heck Cyclizations and Cascades of *N*‐(Pentafluorobenzoyloxy)carbamates

**DOI:** 10.1002/anie.201801109

**Published:** 2018-03-22

**Authors:** Ian R. Hazelden, Rafaela C. Carmona, Thomas Langer, Paul G. Pringle, John F. Bower

**Affiliations:** ^1^ School of Chemistry University of Bristol Bristol BS8 1TS UK; ^2^ Pharmaceutical Technology & Development AstraZeneca Charter Way Macclesfield SK10 2NA UK

**Keywords:** aza-Heck reaction, cascade reactions, N-heterocycles, palladium

## Abstract

Ligand‐enabled aza‐Heck cyclizations and cascades of *N*‐(pentafluorobenzoyloxy)carbamates are described. These studies encompass the first examples of efficient non‐biased 6‐*exo* aza‐Heck cyclizations. The methodology provides direct and flexible access to carbamate protected pyrrolidines and piperidines.

Pyrrolidines and piperidines are two of the most common saturated heterocycles used in pharmaceutical development (Scheme 1 A).^1^ Consequently, efficient and general methods for their preparation are required. A conceptually appealing approach lies in the intramolecular aza‐Wacker process, where oxidative cyclization of an NH nucleophile with an alkene occurs under Pd^II^‐catalyzed conditions (Scheme 1 B).^2^ This method has been developed extensively, but, in general, still requires relatively acidic NH units, such as sulfonamides (PG=SO_2_R), for efficient reactivity.^2^, ^3^, ^4^ Aza‐Wacker cyclizations of less acidic carbamates (PG=CO_2_R) are much slower3e and are limited to 5‐ring cyclizations involving more reactive classes of alkene (e.g., cyclic or sterically undemanding variants).3c,3e,3f, 4 Because carbamate protecting groups (e.g., Boc, Cbz) offer the greatest downstream flexibility, methods that can circumvent these limitations and provide direct access to protected pyrrolidines and piperidines are likely to find broad use.

**Scheme 1:**
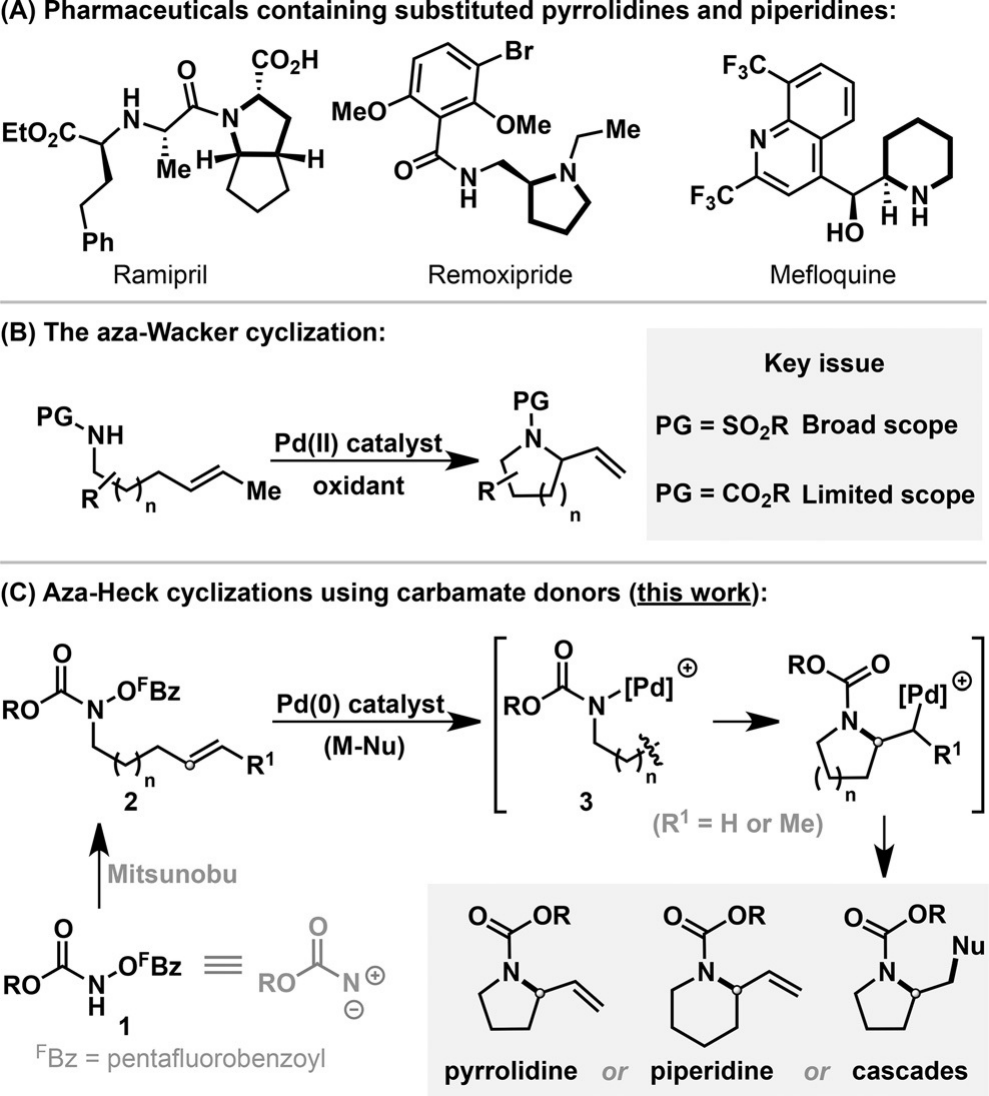
Introduction.

A solution to the aza‐Wacker “carbamate problem” potentially resides in the development of an aza‐Heck process where an activated *N*‐hydroxycarbamate unit (**2**) is exploited for N−O oxidative addition (to **3**) prior to C−N bond forming migratory insertion of the alkene (Scheme 1 C).^5^ The key to this umpoled approach is that it relies on the electrophilicity of the N‐center rather than on its acidity (cf. Scheme 1 B), such that wide scope might be expected. Further potential benefits include: (a) direct access to the substrates by Mitsunobu alkylation of bifunctional amino reagents **1**,^6^ (b) the avoidance of (hazardous) external oxidants,^7^ which, in turn, should allow highly tunable/stabilizing phosphine ligands to be used, (c) a compatibility with organometallic reagents in cascade processes,^8^ and (d) predictable *syn*‐amino‐palladation of the alkene.^4^ To date, the range of catalytically useful N−O oxidative addition processes developed with Pd^0^‐systems is still very limited,^9^, ^11^, ^12^ such that the viability of the approach in Scheme 1 C was deemed uncertain. Nevertheless, as described below, the identification of a privileged ligand set has allowed us to achieve both the envisaged aza‐Heck cyclizations, as well as related cascade processes. The new method is efficient for both 5‐*exo* and non‐biased 6‐*exo* cyclizations; this latter aspect is particularly significant as prior aza‐Heck protocols cannot achieve cyclizations of this type.^5^, ^10^, ^11^, ^12^ The end result is a highly flexible method that enables the two‐step conversion of bis‐ or trishomoallylic alcohols to carbamate protected pyrrolidines or piperidines.

At the outset of our studies, only three principal classes of aza‐Heck N−O donor were known: O‐^F^Bz ketoxime esters reported by Narasaka et al. in 1999 (Class 1),^10^ O‐^F^Bz hydroxysulfonamides reported by our group in 2016 (Class 2),^11^ and O‐phenyl hydroxamates reported by Watson and co‐workers in 2016 (Class 3).^12^ Each system exhibits prescriptive ligand requirements, although, in general, electron poor P‐based ligands are required for efficient reactivity. Class 1 and Class 2 N−O donors cyclize via a cationic aza‐Pd^II^ intermediate, access to which is driven by facile protodecarboxylation of the pentafluorobenzoate leaving group.10f Given these considerations, we elected to evaluate the cyclization of O‐^F^Bz carbamate **2 a** in the presence of Pd systems modified by weak donor ligands. Gratifyingly, we found that the target cyclization was feasible, and, for this non‐demanding system, a variety of triarylphosphine ligands were reasonably effective using Pd_2_(dba)_3_ as the precatalyst (see the Supporting Information). Ultimately, the optimal system was PA‐Ph (**L‐1**, PA=1,3,5,7‐tetramethyl‐2,4,8‐trioxa‐6‐phosphaadamantanyl) and, using this ligand, we were able to access target **4 a** in 85 % yield after optimization of other reaction parameters. **L‐1** is a bulky and electron poor ligand, with the latter facet resulting from its constrained C−P−C bond angle and inductively withdrawing oxygen atoms.^13^ The bulky *tert*‐butyl unit of **2 a** is also beneficial, with less sterically demanding systems **2 b** and **2 c** cyclizing in lower but acceptable yields.

The efficacy of **L‐1** prompted us to undertake the one‐step synthesis of a variety of electronically tuned variants via arylation of commercially available PA‐H (see the SI). These studies revealed that systems with electron withdrawing groups at the *para*‐position of the aryl unit were especially effective, such that **L‐2** and **L‐3** emerged as complementary ligands for subsequent studies. Using this ligand set, we explored the scope of the catalyst system for 5‐*exo* aza‐Heck cyclizations and found it to be highly effective across a wide range of substrates (Table 1). Different carbamates are tolerated (**4 a**–**d**), diastereoselective processes are readily achieved (**4 f**–**h**), tetrasubstituted stereocenters can be constructed (**4 i**, **4 k**, and **4 m**) and electron poor alkenes participate efficiently (**2 j** to **4 j**). The method is especially powerful for bicyclic ring construction; 5‐*exo* cyclization onto exocyclic (**4 k**) or cyclic (**4 l**) alkenes provided complex perhydroindole scaffolds, and spiro (**4 m**) or transannular (**4 n**) C−N bond formations were also efficient. For demanding systems (e.g., **4 i**) **L‐2** or **L‐3** provide 10–15 % higher yields than **L‐1** (selected comparisons are given in the SI). The results in Table 1 show that the aza‐Heck method offers far greater scope for 5‐*exo* cyclizations than currently available aza‐Wacker protocols.

**Table 1:** Carbamate protected pyrrolidines by aza‐Heck cyclization. 
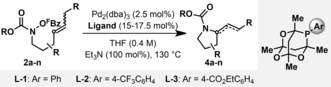

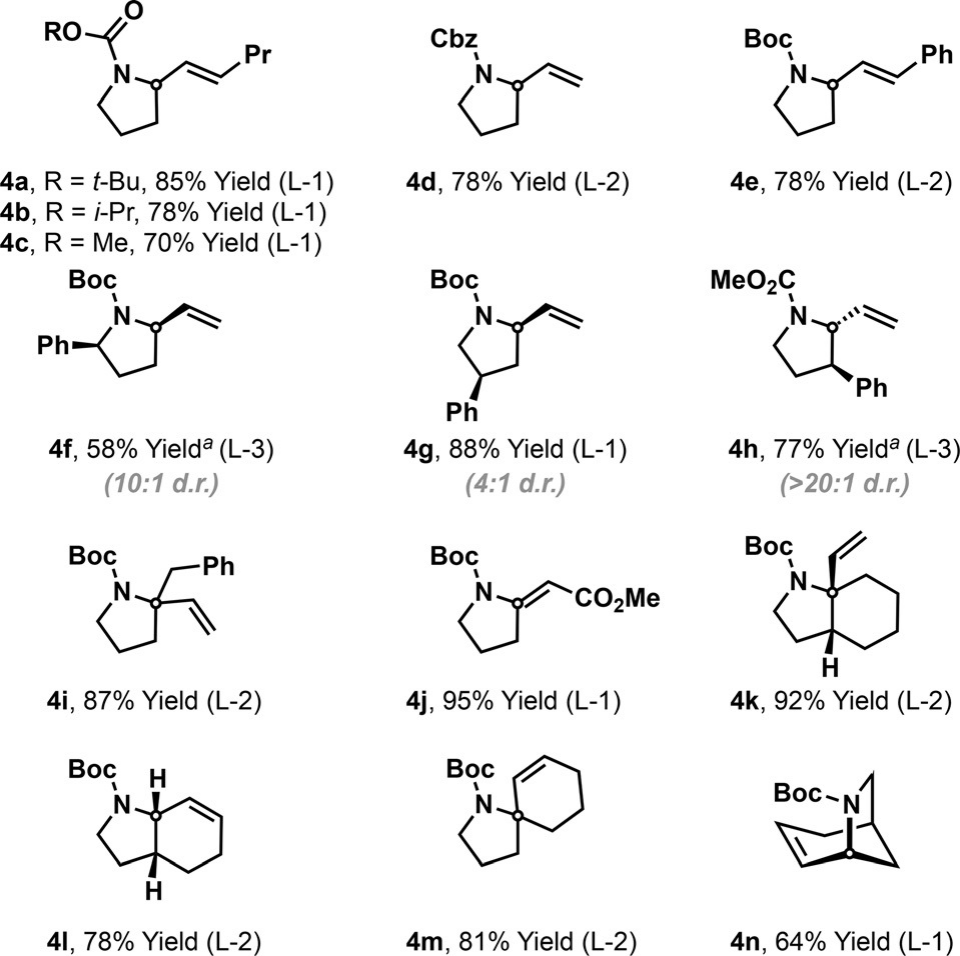

[a] Dioxane (0.3 M) was used as solvent. Alkene geometry of substrates: **2 a**, *E*; **2 b**, *E*; **2 c**, *E*; **2 d**, *E*; **2 e**, 6:1 *E*:*Z*; **2 f**, *E*; **2 g**, *E*; **2 h**, *E*; **2 i**, *Z*; **2 j**, *E*; **2 k**, *E*.

Prior classes of aza‐Heck process cannot achieve efficient 6‐*exo* cyclizations of non‐biased systems, and a solution to this issue represents a longstanding challenge of the area.5a We were pleased to find that the present method addresses this, as demonstrated by the cyclizations of **2 o** and **2 p**, which occurred with good levels of efficiency to afford **4 o** and **4 p**, respectively (Table 2). More highly substituted systems can also be generated (e.g., **4 s** and **4 t**), with the method offering particularly good scope for the construction of tetrahydroisoquinolines (**4 r** and **4 u**), as well as unusual aza‐variants (**4 v** and **4 w**). The process is effective for cyclizations involving both electron rich (**4 r**) and electron poor (e.g. **4 u**) alkenes. For these more demanding 6‐*exo* cyclizations an *N*‐Boc group is required; cyclization to afford methyl carbamate system **4 q** occurred in only 33 % yield under optimized conditions. The use of PA‐Ar ligand systems is also critical for the processes in Table 2, with **L‐2** or **L‐3** being the preferred variants. Triarylphosphines that were effective for 5‐*exo* cyclization generated **4 p** in less than 10 % yield (see the SI). The PA‐Ar ligand system even enabled 7‐*exo* cyclization to afford **4 x**, albeit in modest yield.

**Table 2:** Carbamate protected heterocycles by 6‐ and 7‐*exo* aza‐Heck cyclization. 
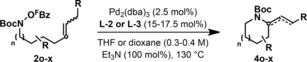

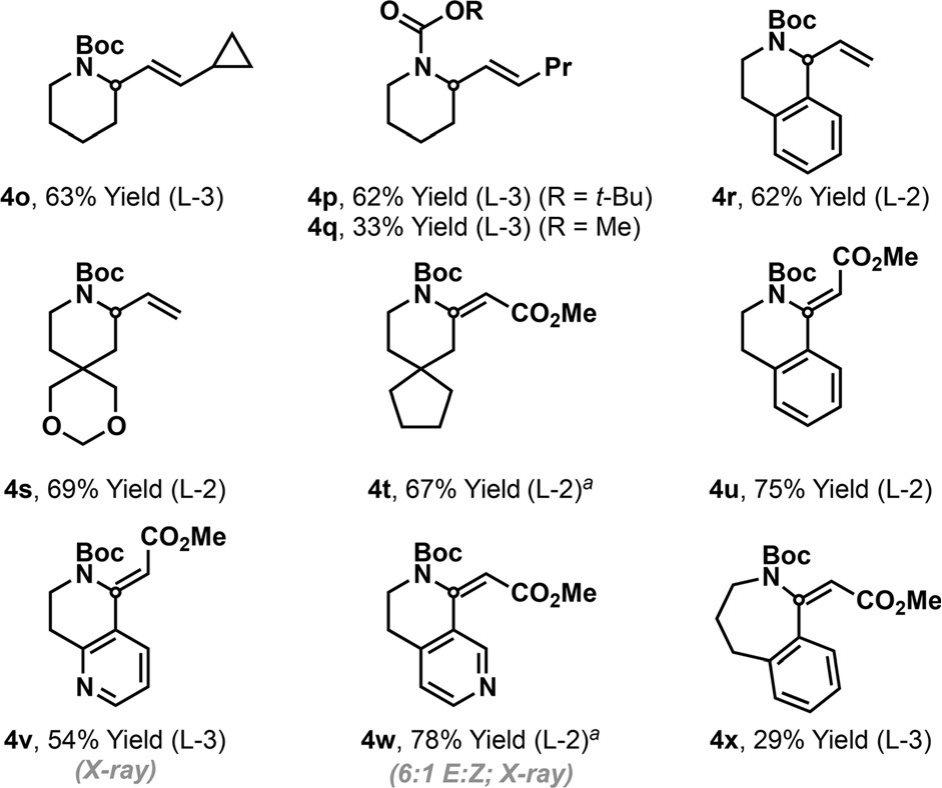

[a] Et_3_N (300 mol%) was used. Alkene geometry of substrates: **2 o**, *Z*; **2 p**, *Z*; **2 q**, *Z*; **2 r**, 3:1 *Z*:*E*; **2 s**, *E*; **2 t**, *E*; **2 u**, *E*; **2 v**, *E*; **2 w**, *E*; **2 x**, *E*.

For the processes described here, our collective observations are supportive of an aza‐Heck pathway akin to that proposed for Class 1 and Class 2 N−O donors.10f, 11 Under optimized conditions, cyclization of O^F^Bz system **2 k** in the presence of NH system **5** provided target **4 k** in 79 % yield and aza‐Wacker product **4 a** was not observed (Scheme 2 A). This result confirms that the N−O bond acts as an internal oxidant only. Accordingly, N−O oxidative addition to **3** should be followed by *syn*‐stereospecific amino‐palladation of the alkene.^14^ Consistent with this, cyclization of *trans*‐acrylate **2 t** delivered adduct **4 t** as a single geometric isomer, in which the alkene substituents that were present in the starting material are now in a *cis*‐arrangement. The observed switch in geometry is consistent with a sequence of *syn*‐amino‐palladation and *syn*‐β‐hydride elimination (Scheme 2 B); a similar phenomenon is observed in the conventional Heck reaction.^15^ For the cyclization of **2 u** and **2 v**, this geometry inversion was not observed at full conversion, with **4 u** and **4 v** formed in >25:1 *Z*:*E* ratios. However, when the cyclization of **2 u** was run to partial conversion (3 h), **4 u** was generated in a 14:1 *E*:*Z* ratio, such that isomerization of the initially formed product likely accounts for the geometry of isolated material (see the SI for details).^16^ As with Class 1 and 2 aza‐Heck processes, protodecarboxylation of the pentafluorobenzoate leaving group likely plays a key role in the processes described here. ^19^F and ^1^H NMR studies revealed that this process is intimately linked to turnover; in the cyclization of **CF_3_‐2 i**, C_6_F_5_H was formed at the same rate as cyclization product **CF_3_‐4 i** (Scheme 2 C). Accordingly, we suggest that a cationic aza‐Pd^II^ intermediate is required for cyclization and access to this is driven by triethylammonium mediated protodecarboxylation of pentafluorobenzoate, a process that we have shown to be facile.10f The efficiency of the PA‐Ar ligand system is consistent with studies by Hanley and Hartwig where electron poor and bulky P‐based ligands were found to accelerate alkene aza‐palladation in other contexts.^17^ For the current processes, the synergy of a bulky ligand system and a bulky N‐protecting group may be especially beneficial, and this might account for the higher efficiencies observed for *N*‐Boc protected systems. The conformational control that this unit provides is also likely a key factor.

**Scheme 2:**
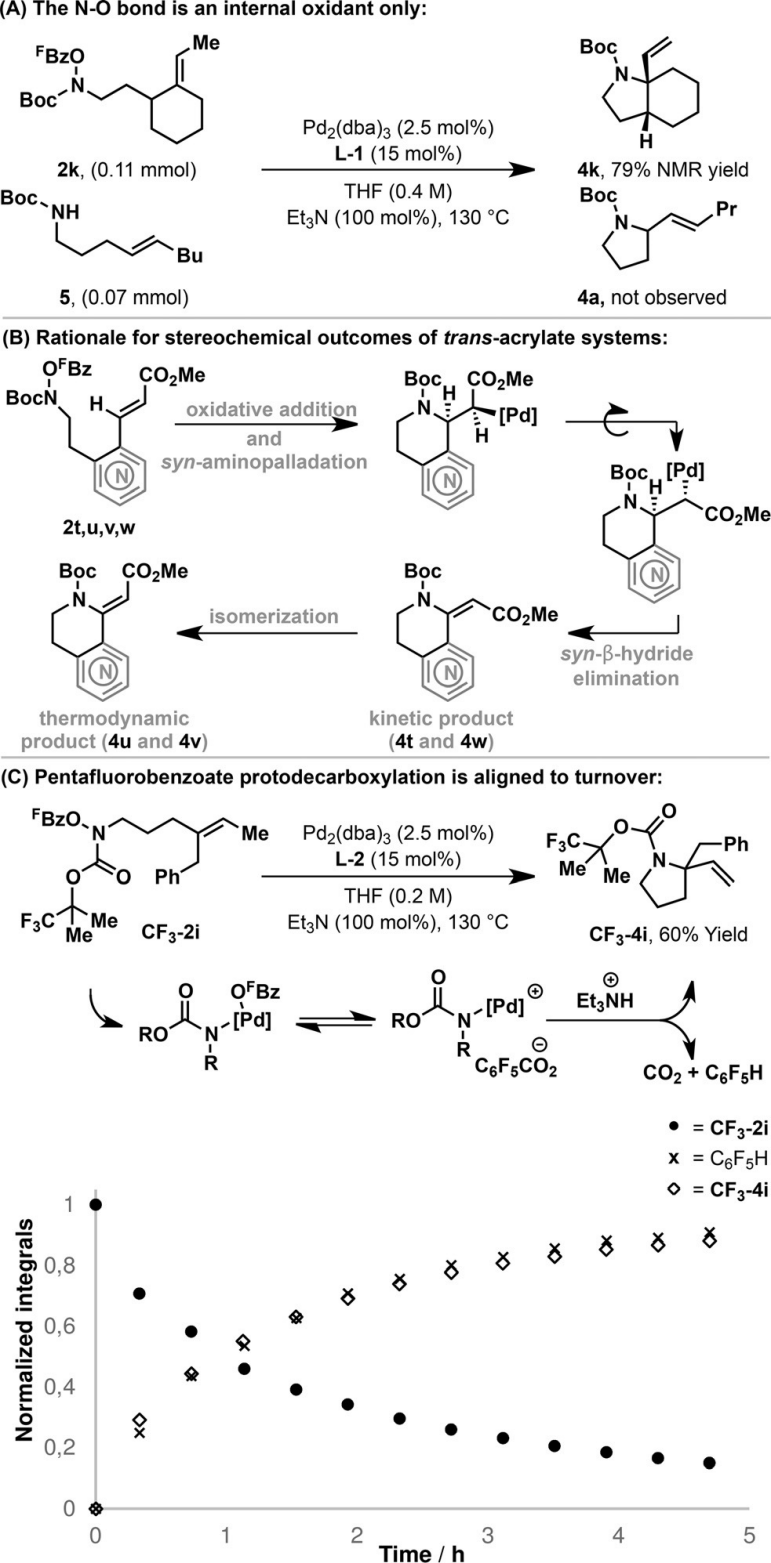
Key mechanistic observations.

The aza‐Heck process can also be adapted to cascade sequences where the alkyl‐Pd^II^ intermediate formed upon alkene amino‐palladation is diverted to a subsequent C−C bond forming event. For example, aza‐Heck–Heck cyclization of bis‐alkenyl system **2 y** delivered spirocycle **4 y** in 68 % yield (Scheme 3 A). We have also assessed the feasibility of partially intermolecular cascade processes as a means of providing a modular and flexible approach to alkene 1,2‐carboamination (Scheme 3 B).10f, 18, 19 Cyclization of **6 a** in the presence of *N*‐methylindole‐2‐boronic acid pinacol ester (200 mol %) provided 1,2‐amino‐arylation product **7 aa** in 73 % yield. Other electron rich heteroaryl boronic esters were also able to trap the alkyl‐Pd^II^ intermediate efficiently to give 1,2‐amino‐arylation products **7 ab**–**7 ad**.

**Scheme 3:**
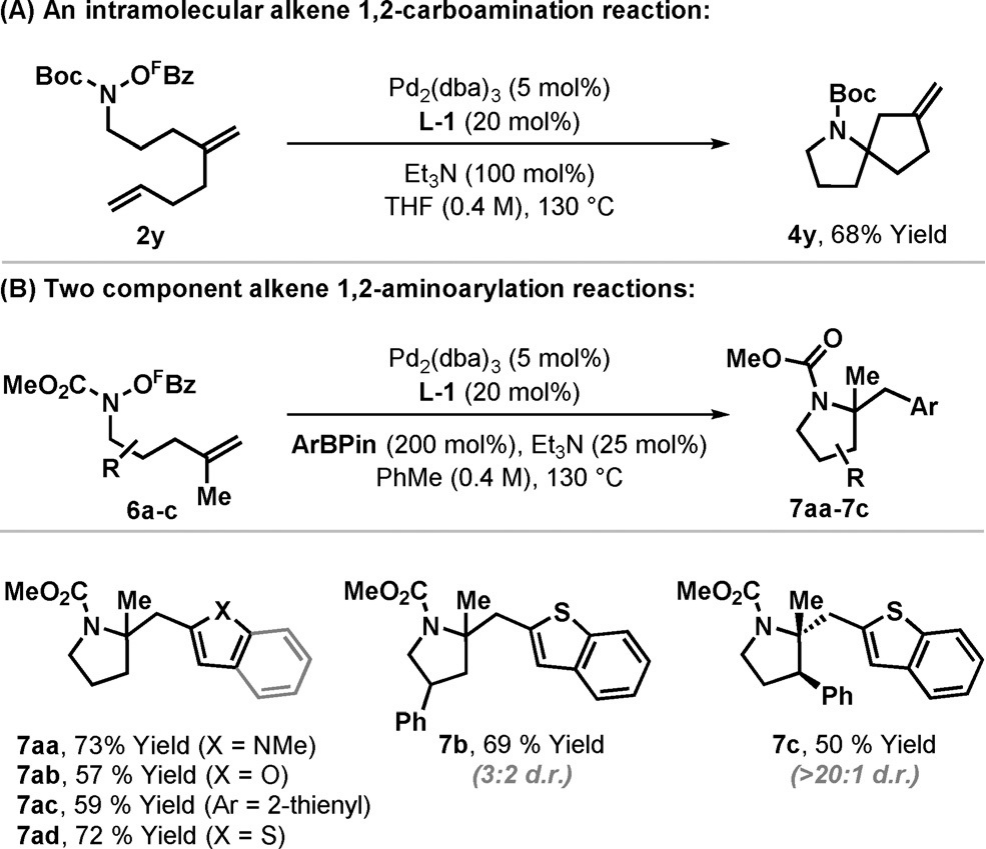
Cascade processes.

In summary, we outline highly efficient aza‐Heck cyclizations of activated *N*‐hydroxycarbamates. The chemistry is reliant on PA‐Ar ligand systems, and, importantly, these allow, for the first time, efficient non‐biased 6‐*exo* cyclizations. Further generalization of the approach, including the development of asymmetric variants^20^ and other classes of cascade reaction, will be reported in due course. In broader terms, the studies described here have uncovered a new entry to aza‐Pd^II^ intermediates via N−O oxidative addition. Given the now broad utility of oxime ester derived imino‐Pd^II^ intermediates,^10^, ^21^ application of this unusual initiation mode^9^ in the design of other redox neutral C−N bond formations can be anticipated.

## Conflict of interest

The authors declare no conflict of interest.

## Supporting information

As a service to our authors and readers, this journal provides supporting information supplied by the authors. Such materials are peer reviewed and may be re‐organized for online delivery, but are not copy‐edited or typeset. Technical support issues arising from supporting information (other than missing files) should be addressed to the authors.

Supplementary
