# Supplementary material for: Pyrrolidines and Piperidines by Ligand‐Enabled Aza‐Heck Cyclizations and Cascades of N‐(Pentafluorobenzoyloxy)carbamates
Source: Angew Chem Int Ed Engl. 2018 Mar 22;57(18):5124–8. doi: 10.1002/anie.201801109 (PMC5969259; doi:10.1002/anie.201801109)
Supplement: Supplementary file 1 — Supplementary [file ANIE-57-5124-s001.pdf]

Angewandte Chemie
Eine Zeitschrift der Gesellschaft Deutscher Chemiker

Supporting Information

Pyrrolidines and Piperidines by Ligand-Enabled Aza-Heck Cyclizations and Cascades of *N*-(Pentafluorobenzoyloxy)carbamates

*Ian R. Hazelden, Rafaela C. Carmona, Thomas Langer, Paul G. Pringle, and John F. Bower**

anie_201801109_sm_miscellaneous_information.pdf

Contents

General experimental details 2
General procedures 3
Preactivated reagents for Mitsunobu reactions 5
Phosphaadamantane ligand synthesis 10
Cyclizations to form 5-membered rings 12
Cyclizations to form 6-membered rings 44
Mechanistic investigations 69
Cascade reactions 73
Comparison of diastereocontrol achieved with aza-Heck and aza-Wacker protocols 84
References 86
^{1}H and ^{13}C NMR spectra of novel compounds 88

The experiments specifically identified in the main text as being in the supporting information are highlighted in blue.

General experimental details

Unless stated, all materials were purchased from commercial sources (Acros, Aldrich, Alfa Aesar, Fluorochem and Strem) and used without any further treatment. Reagents requiring purification were purified using standard laboratory techniques according to methods published by Perrin, Armarego, and Perrin (Pergamon Press, 1966). Catalytic reactions were carried out in Young-type re-sealable tubes. Anhydrous solvents were obtained by distillation using standard procedures or by passage through drying columns supplied by Anhydrous Engineering Ltd. High-boiling solvents were removed from the reaction crudes employing rotary evaporators connected with high-vacuum pumps. Flash column chromatography (FCC) was performed using silica gel (Aldrich 40-63 µm, 230-400 mesh). Thin layer chromatography was performed using aluminium backed $60F_{254}$ silica plates. Visualization was achieved by UV fluorescence or a basic $KMnO_4$ solution and heat. Proton nuclear magnetic resonance spectra (NMR) were recorded at 400 MHz or 500 MHz as stated. ^{13}C NMR spectra were recorded at 100 MHz or 125 MHz as stated. ^{19}F NMR spectra were recorded at 283 MHz. Chemical shifts (δ) are given in parts per million (ppm). Peaks are described as singlets (s), doublets (d), triplets (t), quartets (q), multiplets (m) and broad (br). Coupling constants (J) are quoted to the nearest 0.5 Hz. All assignments of NMR spectra were based on 2D NMR data (COSY, HSQC and HMBC). In situ yields were determined by employing 1,3,5-trimethoxybenzene as internal standard. Mass spectra were recorded using a Brüker Daltonics FT-ICR-MS Apex 4e 7.0T FT-MS (ESI^+ mode) and a Shimadzu GCMS QP2010+ (EI^+ mode). Infrared spectra were recorded on a Perkin Elmer Spectrum Two FTIR spectrometer as thin films or solids compressed on a diamond plate. Melting points were determined using Stuart SMP30 melting point apparatus and are reported uncorrected. Enantiomeric excess was determined by integration of chromatogram peaks. Chiral SFC was performed on an Agilent 1260 Infinity SFC Control Module system equipped with a quaternary pump, diode array detector and column thermostat under the conditions specified. The numbering of compound structures does not necessarily reflect the numbering contained in the systematic names.

General procedures

General procedure A: Pentafluorobenzoylation of *N*-hydroxycarbamates

To a suspension of *N*-hydroxycarbamate (1.0 eq.) and pentafluorobenzoic acid (1.0 eq.) in CH_2Cl_2 (10 mL/mmol) at 0 °C was added a solution of *N*-*N'*-dicyclohexylcarbodiimide (1.1 eq.) in CH_2Cl_2 (5 mL/mmol) dropwise. The resulting mixture was stirred at 0 °C for the time noted before filtration to remove the colorless precipitate. The filtrate was concentrated *in vacuo* and the crude product was purified by FCC.

General procedure B: Johnson-Claisen rearrangement

A solution of propionic acid (0.20 eq.) in triethyl orthoacetate (10 eq.) was heated at 110 °C for 1 hour, after this time allylic alcohol (1.0 eq.) was added and the reaction mixture heated at reflux for the time noted. The reaction mixture was cooled to room temperature and concentrated *in vacuo* to afford the crude product which was purified by FCC.

General procedure C: Reduction of carboxylic acids or esters

To a solution of carboxylic acid/ester (1.0 eq.) in anhydrous THF or Et_2O (*approx.* 2.5 mL/mmol) at 0 °C was added $LiAlH_4$ (*equivalents specified*) dropwise. The reaction mixture was warmed to room temperature and monitored by TLC. Upon completion, the reaction mixture was cooled to 0 °C before addition of water (1 mL/g of $LiAlH_4$), 15 % aqueous NaOH (1 mL/g of $LiAlH_4$) and a final portion of water (3 mL/g of $LiAlH_4$). The reaction mixture was stirred at room temperature for around 15 minutes before being dried over Na_2SO_4 and concentrated *in vacuo* to afford the product.

General procedure D: Mitsunobu reaction employing diisopropyl azodicarboxylate

To a solution of alcohol (1.0 eq.), hydroxylamine-derived pronucleophile (1.3 eq.) and PPh_3 (1.5 eq.) in anhydrous THF:PhMe (3:1, 8 mL/mmol) at 0 °C was added a solution of DIAD (1.5 eq.) in anhydrous PhMe (2 mL/mmol) dropwise. The reaction mixture was stirred at room temperature for the time noted before being concentrated *in vacuo* and loaded directly onto silica gel for purification by FCC.

General procedure E: Palladium-catalyzed cyclization

A flame-dried re-sealable tube, fitted with a magnetic stirrer, was charged with cyclization substrate, $Pd_2(dba)_3$ and phosphine ligand. The tube was fitted with a rubber septum and purged with nitrogen before solvent and Et_3N were added *via* syringe. The tube was sealed and heated at the specified temperature for 24 hours unless otherwise noted. The reaction mixture was cooled to room temperature, concentrated *in vacuo* and the crude product was purified by FCC.

General procedure F: *N*-Boc deprotection

A solution of carbamate in TFA (2 mL) and CH_2Cl_2 (2 mL) was stirred at room temperature for 1 hour before being concentrated *in vacuo* to afford the product.

General procedure G: Alkylation and decarboxylation of diethyl malonate

To a suspension of NaH (60% weight in mineral oil, 2.0 eq.) in anhydrous THF (*approx.* 3 mL/mmol) at 0 °C was added diethyl malonate (2.0 eq.) dropwise. The reaction mixture was stirred at 0 °C for 1 hour before dropwise addition of allylic bromide (1.0 eq.). The reaction mixture was warmed to room temperature and monitored by TLC. Upon completion, the reaction mixture was poured into a solution of KOH (12 eq.) in water:MeOH (1:1) and stirred for 30 minutes at room temperature. The reaction mixture was acidified with 10 M aqueous HCl (20 eq.), concentrated to an aqueous solution and extracted with EtOAc (*approx.* 3 × 5 mL/mmol). The crude mixture of malonic acids was dissolved in DMF (*approx.* 2 mL/mmol) and heated at reflux for 3 hours before being concentrated *in vacuo* to afford the crude decarboxylated product.

General procedure E: Palladium-catalyzed cascade reaction

A flame-dried re-sealable tube, fitted with a magnetic stirrer, was charged with cyclization substrate, $Pd_2(dba)_3$, phosphine ligand and aryl boronic acid pinacol ester. The tube was fitted with a rubber septum and purged with nitrogen before solvent and Et_3N were added *via* syringe. The tube was sealed and heated at the specified temperature for 48 hours. The reaction mixture was cooled to room temperature, filtered through a pad of silica, eluted with EtOAc and concentrated *in vacuo*. The crude product was purified by FCC.

Preactivated reagents for Mitsunobu reactions

tert-Butyl ((pentafluorobenzoyl)oxy)carbamate (1a)

BocNHO^{F}Bz

This compound was prepared according to a literature procedure.[1]

To a solution of *tert*-butyl *N*-hydroxycarbamate (6.66 g, 50.0 mmol) in CH_2Cl_2 (150 mL) at 0 °C was added pentafluorobenzoyl chloride (6.90 mL, 50.0 mmol) followed by Et_3N (7.00 mL, 50.0 mmol). The reaction mixture was stirred at room temperature for 3 hours before addition of water (100 mmol). The resulting phases were separated and the aqueous phase was extracted with CH_2Cl_2 (70 mL). The organic phase was dried over Na_2SO_4 and concentrated *in vacuo*. The crude product was purified by FCC (eluent: 9:1 hexane:EtOAc) to afford **1a** (14.7 g, 90 %) as a colorless crystalline solid.

m.p. 72-74 °C (CH_2Cl_2:petroleum ether, *cubes*).

ν_{max} / cm^{-1}: (*solid*) 3270 (br s), 2996 (m), 1779 (s), 1719 (s), 1654 (s), 1505 (s), 1152 (s).

δ_H (301 MHz, $CDCl_3$) 8.10 (1H, s, N$\underline{H}$), 1.52 (9H, s, OC(C$\underline{H}_3$)$_3$).

δ_C (101 MHz, $CDCl_3$) 154.9 (Boc $\underline{C}$=O), 84.4 (O$\underline{C}$(CH_3)$_3$), 28.1 (OC($\underline{C}H_3$)$_3$).

The signals corresponding to the pentafluorobenzoyl group could not be resolved due to their weak intensity.

δ_F (283 MHz, $CDCl_3$) -135.3 – -135.5 (2F, m), -145.5 (1F, tt, *J* = 21.0, 5.5 Hz), -159.1 – -159.4 (2F, m).

HRMS: (ESI^+) Calculated for $C_{12}H_{10}F_5NNaO_4$: 350.0422. Found $[M+Na]^+$: 350.0433.

The spectroscopic properties were consistent with the data available in the literature.[1]

Isopropyl *N*-hydroxycarbamate

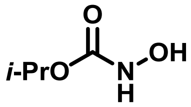

To a suspension of $NH_2OH{\cdot}HCl$ (5.00 g, 72.0 mmol) and K_2CO_3 (8.29 g, 60.0 mmol) in THF (120 mL) at 0 °C was added a solution of isopropyl chloroformate (1.0 M in PhMe, 60 mL, 60 mmol) dropwise over around 30 minutes. The reaction mixture was stirred at room temperature for 48 hours before being filtered. The filtrate was concentrated *in vacuo* and the crude product was purified by FCC (eluent: 1:1 hexane:EtOAc) to afford the title compound (2.18 g, 31 %) as a colorless crystalline solid.

ν_{max} / cm^{-1}: (*film*) 3290 (br s), 2984 (m), 2934 (m), 1704 (s), 1267 (s), 1103 (s).

δ_H (400 MHz, $CDCl_3$) 7.28 – 7.11 (2H, m, N$\underline{H}$ and O$\underline{H}$), 4.99 (1H, hept, *J* = 6.5 Hz, OC$\underline{H}$(CH_3)$_2$), 1.26 (6H, d, *J* = 6.5 Hz, OCH(C$\underline{H}_3$)$_2$).

δ_C (101 MHz, $CDCl_3$) 159.4 ($\underline{C}$=O), 70.5 ($O\underline{C}H(CH_3)_2$), 22.1 ($OCH(\underline{C}H_3)_2$).

HRMS: (ESI^+) Calculated for $C_4H_9NNaO_3$: 142.0475. Found $[M+Na]^+$: 142.0478.

Isopropyl ((pentafluorobenzoyl)oxy)carbamate (1b)

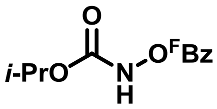

General procedure A: The preceding compound (2.00 g, 16.8 mmol) was employed. The reaction time was 18 hours. FCC (eluent: 9:1 hexane:EtOAc) afforded **1b** (3.86 g, 73 %) as a colorless crystalline solid.

m.p. 54-55 °C (CH_2Cl_2:petroleum ether, *columnar*).

ν_{max} / cm^{-1}: (*solid*) 3230 (br s), 2988 (m), 1776 (s), 1717 (s), 1654 (m), 1489 (s), 1191 (s).

δ_H (400 MHz, $CDCl_3$) 8.22 (1H, s, $N\underline{H}$), 5.05 (1H, hept, J = 6.5 Hz, $OC\underline{H}(CH_3)_2$), 1.30 (6H, d, J = 6.5 Hz, $OC\underline{H}(CH_3)_2$).

δ_C (101 MHz, $CDCl_3$) 155.5 (*i*-PrO–$\underline{C}$=O), 71.9 ($O\underline{C}H(CH_3)_2$), 21.7 ($OCH(\underline{C}H_3)_2$).

The signals corresponding to the pentafluorobenzoyl group could not be resolved due to their weak intensity.

δ_F (377 MHz, $CDCl_3$) -135.3 – -135.5 (2F, m), -145.3 (1F, tt, J = 21.0, 6.0 Hz), -159.1 – -159.2 (2F, m).

HRMS: (ESI^+) Calculated for $C_{11}H_8F_5NNaO_4$: 336.0266. Found $[M+Na]^+$: 336.0281.

Methyl *N*-hydroxycarbamate

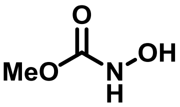

To a solution of $NH_2OH\cdot HCl$ (17.8 g, 400 mmol) and K_2CO_3 (44.4 g, 300 mmol) in Et_2O (120 mL) and water (2 mL) at 0 °C was added methyl chloroformate (18.9 mL, 244 mmol) over around 20 minutes. The reaction mixture was stirred at 0 °C for 30 minutes before being warmed to room temperature and stirred for 16 hours. The reaction mixture was filtered, rinsed with Et_2O (100 mL) and the filtrate was concentrated *in vacuo*. The crude product was crystallized from Et_2O to afford the title compound (12.4 g, 56%) as a colorless crystalline solid.

ν_{max} / cm^{-1}: (*solid*) 3264 (br s), 2967 (m), 2881 (m), 1683 (s), 1463 (s), 1275 (s), 1116 (s).

δ_H (400 MHz, $CDCl_3$) 7.63 – 7.29 (2H, br s, $N\underline{H}$ and $O\underline{H}$), 3.77 (3H, s, $OC\underline{H}_3$).

δ_C (101 MHz, $CDCl_3$) 160.1 ($\underline{C}$=O), 53.3 ($O\underline{C}H_3$).

HRMS: (ESI^+) Calculated for $C_2H_5NNaO_3$: 114.0162. Found $[M+Na]^+$: 114.0166.

Methyl ((pentafluorobenzoyl)oxy)carbamate (1c)

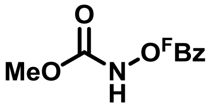

To a solution of methyl *N*-hydroxycarbamate (4.55 g, 50.0 mmol) in CH_2Cl_2 (150 mL) at 0 °C was added pentafluorobenzoyl chloride (6.90 mL, 50.0 mmol) followed by Et_3N (7.00 mL, 50.0 mmol). The reaction mixture was stirred at room temperature for 5 hours before addition of water (100 mmol). The resulting phases were separated and the aqueous phase was extracted with CH_2Cl_2 (70 mL). The organic phase was dried over Na_2SO_4 and concentrated *in vacuo*. The crude product was purified by FCC (eluent: 4:1 hexane:EtOAc) to afford **1c** (12.2 g, 86 %) as a colorless crystalline solid.

m.p. 46-47 °C (CH_2Cl_2:petroleum ether, *prisms*).

ν_{max} / cm^{-1}: (*solid*) 3220 (br s), 2971 (m), 1794 (s), 1737 (s), 1654 (m), 1503 (s), 1169 (s).

δ_H (400 MHz, $CDCl_3$) 8.34 (1H, s, N$\underline{\text{H}}$), 3.87 (3H, s, OC$\underline{\text{H}}_3$).

δ_C (101 MHz, $CDCl_3$) 156.5 (MeO–$\underline{\text{C}}$=O), 54.1 (O$\underline{\text{C}}$$H_3$).

The signals corresponding to the pentafluorobenzoyl group could not be resolved due to their weak intensity.

δ_F (377 MHz, $CDCl_3$) -135.2 – -135.4 (2F, m), -145.2 (1F, tt, *J* = 21.0, 6.0 Hz), -159.2 – -159.4 (1F, m).

HRMS: (ESI^+) Calculated for $C_9H_4F_5NNaO_4$: 307.9953. Found $[M+Na]^+$: 307.9964.

Benzyl ((pentafluorobenzoyl)oxy)carbamate (1d)

CbzNHO^{F}Bz

General procedure A: Benzyl *N*-hydroxycarbamate (9.72 g, 58.2 mmol) was employed. The reaction time was 16 hours. FCC (eluent: 4:1 hexane:EtOAc) afforded **1d** (15.6 g, 74 %) as a colorless crystalline solid.

δ_H (400 MHz, $CDCl_3$) 8.35 (1H, br s), 7.37 (5H, br s), 5.26 (2H, s).

δ_C (101 MHz, $CDCl_3$) 155.7, 134.6, 128.8, 128.7, 128.4, 69.0.

The signals corresponding to the pentafluorobenzoyl group could not be resolved due to their weak intensity.

δ_F (377 MHz, $CDCl_3$) -135.1 – 135.2 (2F, m), -145.1 (1F, tt, *J* = 21.0, 6.0 Hz), -159.1 – -159.3 (2F, m).

The spectroscopic properties were consistent with the data available in the literature.[2]

1,1,1-Trifluoro-2-methylpropan-2-yl 1*H*-imidazole-1-carboxylate

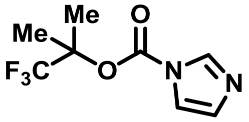

A solution of 1,1,1-trifluoro-2-methylpropan-2-ol (4.92 g, 38.4 mmol) and carbonyldiimidazole (12.5 g, 76.8 mmol) in $CHCl_3$ (80 mL) was stirred at room temperature for 22 hours. To the reaction mixture was added water (80 mL), the resulting phases were separated and the aqueous phase was extracted with $CHCl_3$ (40 mL). The combined $CHCl_3$ phases were washed with brine (100 mL), dried over Na_2SO_4 and concentrated *in vacuo* to afford to afford the title compound (5.51 g, 65 %) as a colorless crystalline solid.

ν_{max} / cm^{-1}: (*solid*) 3151 (m), 3127 (m), 1759 (s), 1154 (s).

δ_H (400 MHz, $CDCl_3$) 8.08 (1H, dd, *J* = 1.0, 1.0 Hz, ArC<u>H</u>), 7.37 (1H, dd, *J* = 1.5, 1.0 Hz, ArC<u>H</u>), 7.07 (1H, dd, *J* = 1.5, 1.0 Hz, ArC<u>H</u>), 1.83 (6H, q, *J* = 1.0 Hz, $OC(CF_3)(C\underline{H}_3)_2$).

δ_C (101 MHz, $CDCl_3$) 146.0 (<u>C</u>=O), 137.2 (Ar<u>C</u>H), 131.0 (Ar<u>C</u>H), 124.40 (q, *J* = 282.5 Hz, $OC(\underline{C}F_3)(CH_3)_2$), 117.2 (Ar<u>C</u>H), 83.7 (q, *J* = 30.5 Hz, $O\underline{C}(CF_3)(CH_3)_2$), 19.4 (q, *J* = 1.5 Hz, $OC(CF_3)(\underline{C}H_3)_2$).

δ_F (377 MHz, $CDCl_3$) -83.7 (3F, s).

HRMS: (ESI^+) Calculated for $C_8H_9F_3N_2NaO_2$: 245.0508. Found $[M+Na]^+$: 245.0511.

1,1,1-Trifluoro-2-methylpropan-2-yl hydroxycarbamate

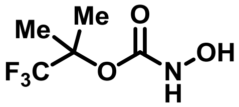

A solution of $NH_2OH{\cdot}HCl$ (3.00 g, 43.2 mmol) and $NaHCO_3$ (5.44 g, 64.8 mmol) in THF (60 mL) and water (60 mL) was stirred at room temperature for 30 minutes before addition of the preceding compound (4.80 g, 21.6 mmol). The reaction mixture was stirred for 20 hours before being partitioned between EtOAc (100 mL) and brine (100 mL). The phases were separated and the organic phase was concentrated *in vacuo*. The crude material was redissolved in EtOAc (100 mL) and washed with water (3 × 40 mL) before being dried over Na_2SO_4 and concentrated in vacuo to afford the title compound (1.77 g, 44 %) as a colorless crystalline solid.

ν_{max} / cm^{-1}: (*film*) 3284 (br s), 2956 (m) 1703 (s), 1132 (s).

δ_H (400 MHz, $CDCl_3$) 7.43 – 7.16 (2H, br s, N<u>H</u> and O<u>H</u>), 1.71 (6H, q, *J* = 1.0 Hz, $OC(CF_3)(C\underline{H}_3)_2$).

δ_C (101 MHz, $CDCl_3$) 156.7 (<u>C</u>=O), 124.8 (q, *J* = 282.5 Hz, $OC(\underline{C}F_3)(CH_3)_2$), 81.3 (q, *J* = 30.0 Hz, $O\underline{C}(CF_3)(CH_3)_2$), 19.7 (q, *J* = 1.5 Hz, $OC(CF_3)(\underline{C}H_3)_2$).

δ_F (377 MHz, $CDCl_3$) -83.8 (3F, s).

HRMS: (ESI^+) Calculated for $C_5H_8F_3NNaO_3$: 210.0348. Found $[M+Na]^+$: 210.0347.

1,1,1-Trifluoro-2-methylpropan-2-yl ((pentafluorobenzoyl)oxy)carbamate (CF_3-1a)

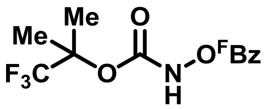

General procedure A: The preceding compound (1.76 g, 9.41 mmol) was employed. The reaction time was 21 hours. FCC (gradient elution: 15:1 – 7:1 hexane:EtOAc) afforded **CF_3-1a** (2.67 g, 74 %) as a colorless crystalline solid.

m.p. 87-88 °C (CH_2Cl_2:petroleum ether, *needles*).

ν_{max} / cm^{-1}: (*solid*) 3366 (s), 2940 (m), 1782 (s), 1763 (s), 1655 (s), 1498 (s), 1133 (s).

δ_H (400 MHz, $CDCl_3$) 8.34 (1H, br s, N<u>H</u>), 1.75 (6H, q, *J* = 1.0 Hz, $OC(CF_3)(C\underline{H}_3)_2$).

δ_C (101 MHz, $CDCl_3$) 158.5 (^{F}Bz <u>C</u>=O), 153.1 (Boc <u>C</u>=O), 124.3 (q, *J* = 282.5 Hz, $OC(\underline{C}F_3)(CH_3)_2$), 82.5 (q, *J* = 30.5 Hz, $O\underline{C}(CF_3)(CH_3)_2$), 19.3 (q, *J* = 1.5 Hz, $OC(CF_3)(\underline{C}H_3)_2$).

The aromatic signals corresponding to the pentafluorobenzoyl group could not be resolved due to their weak intensity.

δ_F (376 MHz, $CDCl_3$) -83.8 (3F, s), -135.0 – -135.2 (2F, m), -144.8 (1F, tt, *J* = 21.0, 6.0 Hz), -159.0 – -159.2 (2F, m).

HRMS: (ESI^+) Calculated for $C_{12}H_7F_8NNaO_4$: 404.0140. Found $[M+Na]^+$: 404.0157.

Phosphaadamantane ligand synthesis

1,3,5,7-Tetramethyl-8-(4-(trifluoromethyl)phenyl)-2,4,6-trioxa-8-phosphaadamantane (L-2)

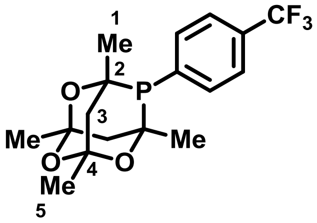

This compound was prepared using an adaptation of a literature procedure.[3]

A suspension of PA-H[4] (1.30 g, 6.00 mmol), $Pd(PPh_3)_4$ (173 mg, 150 µmol), 1-bromo-4-(trifluoromethyl)benzene (0.70 mL, 5.00 mmol) and K_2CO_3 (1.38 g, 10.0 mmol) in anhydrous xylenes (10 mL, sparged with argon) was heated at 110 °C for 20 hours. The reaction mixture was cooled to room temperature before being filtered through silica and rinsed with Et_2O (40 mL). The filtrate was concentrated *in vacuo*. FCC (gradient elution: 1:9 – 0:1 hexane:PhMe) afforded PA-(4-$(CF_3)C_6H_4$) (**L-2**) (1.34 g, 74 %) as a colorless crystalline solid.

m.p. 132-133 °C (CH_2Cl_2:petroleum ether, *plates*).

ν_{max} / cm^{-1}: (*solid*) 3005 (m), 2920 (m), 1606 (m), 1322 (s), 1119 (s).

δ_H (400 MHz, $CDCl_3$) 7.96 (2H, ddq, *J* = 8.5, 7.5, 1.0 Hz, ArCH), 7.61 (2H, dq, *J* = 7.5, 1.0 Hz, ArCH), 2.06 (1H, dd, *J* = 13.5, 7.5 Hz, C**3**-H), 1.94 (1H, dd, *J* = 24.5, 13.5 Hz, C**3**-H'), 1.66 (1H, d, *J* = 13.5 Hz, C**3'**-H), 1.52 (3H, d, *J* = 13.0 Hz, C**1**-H_3), 1.50 (1H, dd, *J* = 13.5, 4.0 Hz, C**3'**-H'), 2 × 1.42 (3H, s, C**5**-H_3 and 3H, s, C**5'**-H_3), 1.26 (3H, d, *J* = 13.0 Hz, C**1'**-H_3).

δ_C (101 MHz, $CDCl_3$) 139.2 (d, *J* = 31.0 Hz, ArC), 135.2 (d, *J* = 19.5 Hz, ArCH), 131.3 (q, *J* = 32.5 Hz, ArC), 124.9 (dq, *J* = 7.5, 3.5 Hz, ArCH), 96.8 (C**4**), 96.1 (C**4'**), 73.3 (d, *J* = 22.5 Hz, C**2**), 73.1 (d, *J* = 8.0 Hz, C**2'**), 45.1 (d, *J* = 17.5 Hz, C**3**), 36.3 (d, *J* = 2.0 Hz, C**3'**), 28.0 (C**5'**), 27.7 (C**5**), 27.4 (d, *J* = 22.0 Hz, C**1'**), 26.8 (d, *J* = 11.0 Hz, C**1**).

The signal corresponding to the trifluoromethyl group could not be resolved due to its weak intensity.

δ_F (377 MHz, $CDCl_3$) -63.0.

δ_P (162 MHz, $CDCl_3$) -25.0.

HRMS: (ESI^+) Calculated for $C_{17}H_{21}F_3O_3P$: 361.1175. Found $[M+H]^+$: 361.1174.

Ethyl 4-(1,3,5,7-tetramethyl-2,4,6-trioxa-8-phosphaadamantan-8-yl)benzoate (L-3)

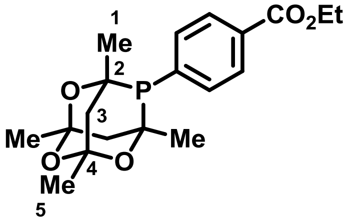

This compound was prepared using an adaptation of a literature procedure.[3]

A solution/suspension of PA-H[4] (432 mg, 2.00 mmol), $Pd(PPh_3)_4$ (69.3 mg, 60 µmol), ethyl 4-bromobenzoate (0.39 mL, 2.40 mmol) and K_2CO_3 (552 mg, 4.00 mmol) in anhydrous xylenes (10 mL, sparged with argon) was heated at 110 °C for 16 hours. The reaction mixture was cooled to room temperature before being filtered through silica and rinsed with Et_2O (40 mL). The filtrate was concentrated *in vacuo*. FCC (eluent: 39:1 pentane:acetone) afforded PA-(4-(CO_2Et)C_6H_4) (**L-3**) (636 mg, 87 %) as a colorless crystalline solid.

m.p. 114-116 °C (CH_2Cl_2:petroleum ether, *cubes*).

ν_{max} / cm^{-1}: (*solid*) 2985 (m), 2917 (m), 1715 (s), 1596 (m), 1371 (s), 1262 (s).

δ_H (400 MHz, $CDCl_3$) 8.02 (2H, d, *J* = 8.0 Hz, ArC<u>H</u>), 7.91 (2H, dd, *J* = 8.0, 7.0 Hz, ArC<u>H</u>), 4.39 (1H, q, *J* = 7.0 Hz, OC<u>H</u>$_2$CH$_3$), 2.06 (1H, dd, *J* = 13.5, 7.5 Hz, C**3**-<u>H</u>), 1.94 (1H, dd, *J* = 24.5, 13.5 Hz, C**3**-<u>H</u>'), 1.67 (1H, d, *J* = 13.5 Hz, C**3'**-<u>H</u>), 1.53 (3H, d, *J* = 13.0 Hz, C**1**-<u>H</u>$_3$), 1.47 (1H, dd, *J* = 13.5, 4.0 Hz, C**3'**-<u>H</u>'), 1.43 – 1.37 (9H, m, C**5**-<u>H</u>$_3$, C**5'**-<u>H</u>$_3$ and OCH$_2$C<u>H</u>$_3$), 1.25 (3H, d, *J* = 13.0 Hz, C**1'**-<u>H</u>$_3$).

δ_C (101 MHz, $CDCl_3$) 166.5 (<u>C</u>=O), 140.3 (d, *J* = 30.5 Hz, Ar<u>C</u>)), 135.0 (d, *J* = 19.5 Hz, Ar<u>C</u>H), 131.3 (Ar<u>C</u>), 129.2 (d, *J* = 7.0 Hz, Ar<u>C</u>H), 97.0 (C**4**), 96.2 (C**4'**), 2 × 73.4 (d, *J* = 22.0 Hz, C**2** and d, *J* = 8.0 Hz, C**2'**), 61.3 (O<u>C</u>H$_2$CH$_3$), 45.3 (d, *J* = 17.5 Hz, C**3**), 36.5 (d, *J* = 2.0 Hz, C**3'**), 28.1 (C**5'**), 27.9 (C**5**), 27.6 (d, *J* = 21.5 Hz, C**1'**), 27.0 (d, *J* = 11.0 Hz, C**1**), 14.5 (OCH$_2$<u>C</u>H$_3$).

δ_P (162 MHz, $CDCl_3$) -24.6.

HRMS: (ESI$^+$) Calculated for $C_{19}H_{25}NaO_5P$: 387.1332. Found $[M+Na]^+$: 387.1333.

Cyclizations to form 5-membered rings

Ethyl (*E*)-non-4-enoate

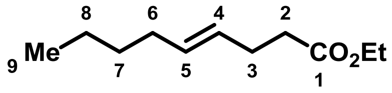

General procedure B: Hept-1-en-3-ol (5.71 g, 50.0 mmol) was employed. The reaction time was 14 hours. FCC (eluent: 49:1 hexane:EtOAc) afforded the title compound (7.10 g, 77 %) as a colorless oil.

ν_{max} / cm^{-1}: (*film*) 2959 (m), 2927 (m), 1735 (s), 1446 (m), 1150 (s).

δ_H (400 MHz, $CDCl_3$) 5.51 – 5.33 (2H, m, C**4**-H and C**5**-H), 4.12 (2H, q, *J* = 7.0 Hz, OCH_2CH_3), 2.38 – 2.26 (4H, m, C**2**-H_2 and C**3**-H_2), 1.97 (2H, dt, *J* = 6.5, 6.5 Hz, C**6**-H_2), 1.35 – 1.26 (4H, m, C**7**-H_2 and C**8**-H_2), 1.25 (3H, t, *J* = 7.0 Hz, OCH_2CH_3), 0.90 – 0.85 (3H, m, C**9**-H_3).

δ_C (101 MHz, $CDCl_3$) 173.3 (C**1**), 131.9 (C**5**), 128.0 (C**4**), 60.3 (OCH_2CH_3), 34.5 (C**2**), 32.3 (C**6**), 31.7 (C**7**), 28.1 (C**3**), 22.2 (C**8**), 14.4 (OCH_2CH_3), 14.0 (C**9**).

HRMS: (ESI^+) Calculated for $C_{11}H_{20}NaO_2$: 207.1356. Found $[M+Na]^+$: 207.1354.

(*E*)-Non-4-en-1-ol

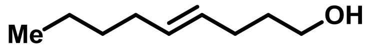

General procedure C: The preceding ester (7.10 g, 38.5 mmol) was employed using anhydrous Et_2O as solvent and 0.8 eq. $LiAlH_4$ (1.0 M in Et_2O). The title compound (5.42 g, 99 %) was isolated as a colorless oil.

δ_H (400 MHz, $CDCl_3$) 5.49 – 5.36 (2H, m), 3.65 (2H, t, *J* = 6.5 Hz), 2.07 (2H, dt, *J* = 7.0, 7.0 Hz), 1.98 (2H, dt, *J* = 6.0, 6.0 Hz), 1.63 (2H, tt, *J* = 7.0, 6.5 Hz), 1.39 – 1.23 (5H, m), 0.88 (3H, t, *J* = 7.0 Hz).

δ_C (101 MHz, $CDCl_3$) 131.4, 129.5, 62.7, 32.6, 32.4, 31.9, 29.1, 22.3, 14.1.

The spectroscopic properties were consistent with the data available in the literature.[5]

***tert*-Butyl (*E*)-non-4-en-1-yl((pentafluorobenzoyl)oxy)carbamate (2a)**

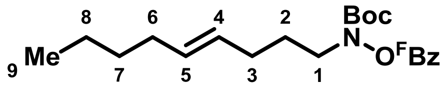

General procedure D: (*E*)-Non-4-en-1-ol (*vide supra*, 213 mg, 1.50 mmol) was employed with **1a**. The reaction time was 23 hours. FCC (eluent: 1:1 petroleum ether:PhMe) afforded **2a** (450 mg, 66 %) as a colorless oil.

ν_{max} / cm^{-1}: (*film*) 2931 (m), 1784 (s), 1722 (s), 1652 (m), 1505 (s), 1154 (s).

δ_H (400 MHz, $CDCl_3$) 5.51 – 5.34 (2H, m, C**4**-$\underline{H}$ and C**5**-$\underline{H}$), 3.69 (2H, t, J = 7.0 Hz, C**1**-$\underline{H}_2$), 2.09 (2H, td, J = 7.5, 7.0 Hz, C**3**-$\underline{H}_2$), 2.00 (2H, dt, J = 8.0, 6.0 Hz, C**6**-$\underline{H}_2$), 1.72 (2H, tt, J = 7.5, 7.0 Hz, C**2**-$\underline{H}_2$), 1.52 (9H, s, OC($C\underline{H}_3)_3$), 1.39 – 1.26 (4H, m, C**7**-$\underline{H}_2$ and C**8**-$\underline{H}_2$), 0.94 – 0.87 (3H, m, C**9**-$\underline{H}_3$).

δ_C (101 MHz, $CDCl_3$) 154.6 (Boc $\underline{C}$=O), 131.7 (C**5**), 128.5 (C**4**), 83.2 (O$\underline{C}(CH_3)_3$), 50.5 (C**1**), 32.2 (C**6**), 31.7 (C**7**), 29.4 (C**3**), 28.0 (OC($\underline{C}H_3)_3$), 26.8 (C**2**), 22.2 (C**8**), 13.9 (C**9**).

The signals corresponding to the pentafluorobenzoyl group could not be resolved due to their weak intensity.

δ_F (377 MHz, $CDCl_3$) -136.4 – -136.7 (2F, m), -146.7 (1F, tt, J = 21.0, 5.0 Hz), -159.4 – -159.6 (2F, m).

HRMS: (ESI^+) Calculated for $C_{21}H_{26}F_5NNaO_4$: 474.1674. Found $[M+Na]^+$: 474.1658.

***tert*-Butyl (*E*)-2-(pent-1-en-1-yl)pyrrolidine-1-carboxylate (4a)**

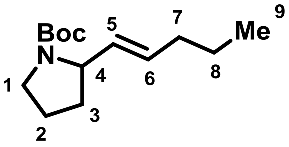

__General procedure E:__ Conditions: 2.5 mol% $Pd_2(dba)_3$; 15 mol% PA-Ph (**L-1**); 100 mol% Et_3N; THF (0.4 M); 130 °C. Substrate **2a** (47.4 mg, 0.105 mmol) was employed. FCC (gradient elution: 39:1 - 19:1 PhMe:EtOAc) afforded **4a** (21.4 mg, 85 %) as a pale yellow oil.

*Selected NMR yields obtained when screening ligands for the conversion of **2a** to **4a**: PPh_3, 62 %; $P(4-(CF_3)C_6H_4)_3$, 78 %; $P(3,5-(CF_3)_2C_6H_3)_3$, 65 %; $P(4-(CN)C_6H_4)_3$, 74 %.*

ν_{max} / cm^{-1}: (*film*) 2964 (m), 2928 (m), 1692 (s), 1389 (s), 1167 (s).

δ_H (400 MHz, $CDCl_3$) 5.52 – 5.37 (1H, m, C**6**-$\underline{H}$), 5.37 – 5.22 (1H, m, C**5**-$\underline{H}$), 4.38 – 4.10 (1H, m, C**4**-$\underline{H}$), 3.46 – 3.23 (2H, m, C**1**-$\underline{H}_2$), 2.01 – 1.92 (3H, m, C**3**-$\underline{H}$ and C**7**-$\underline{H}_2$), 1.89 – 1.74 (2H, m, C**2**-$\underline{H}_2$), 1.65 (1H, dddd, J = 12.5, 6.0, 3.0, 3.0 Hz, C**3**-$\underline{H}$’), 1.46 – 1.31 (11H, m, C**8**-$\underline{H}_2$ and OC($C\underline{H}_3)_3$), 0.88 (3H, t, J = 7.5 Hz, C**9**-$\underline{H}_3$).

δ_C (101 MHz, $CDCl_3$) 154.8 ($\underline{C}$=O), 130.8 (C**5**), 130.2 (C**6**), 79.0 (O$\underline{C}(CH_3)_3$), 58.7 (C**4**), 46.2 (C**1**), 34.4 (C**7**), 32.6 (C**3**), 28.7 (OC($\underline{C}H_3)_3$), 23.0 (C**2**), 22.6 (C**8**), 13.8 (C**9**).

HRMS: (ESI^+) Calculated for $C_{14}H_{25}NNaO_2$: 262.1777. Found $[M+Na]^+$: 262.1776.

Isopropyl (*E*)-non-4-en-1-yl((pentafluorobenzoyl)oxy)carbamate (2b)

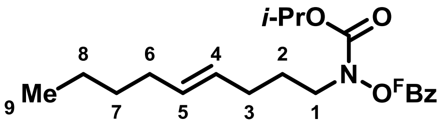

General procedure D: (*E*)-Non-4-en-1-ol (*vide supra*, 284 mg, 2.00 mmol) was employed with **1b**. The reaction time was 15 hours. FCC (gradient elution: 2:3 – 1:4 hexane:PhMe) afforded **2b** (550 mg, 63 %) as a pale yellow oil.

ν_{max} / cm^{-1}: (*film*) 2931 (m), 1786 (s), 1724 (s), 1652 (m), 1505 (s), 1326 (s), 1172 (s).

δ_H (400 MHz, $CDCl_3$) 5.49 – 5.32 (2H, m, C**4**-H and C**5**-H), 5.01 (1H, hept, *J* = 6.5 Hz, OCH(CH_3)$_2$), 3.73 – 3.67 (2H, m, C**1**-H_2), 2.11 – 2.03 (2H, m, C**3**-H_2), 2.02 – 1.94 (2H, m, C**6**-H_2), 1.70 (2H, tt, *J* = 7.5, 7.5 Hz, C**2**-H_2), 1.34 – 1.28 (4H, m, C**7**-H_2 and C**8**-H_2), 1.28 (6H, d, *J* = 6.5 Hz, OCH(CH_3)$_2$), 0.90 – 0.86 (3H, m, C**9**-H_3).

δ_C (101 MHz, $CDCl_3$) 157.4 (^{F}Bz C=O), 155.4 (*i*-PrO–C=O), 132.0 (C**5**), 128.5 (C**4**), 71.4 (OCH(CH_3)$_2$), 50.6 (C**1**), 32.4 (C**6**), 31.8 (C**7**), 29.5 (C**3**), 26.9 (C**2**), 22.3 (C**8**), 22.0 (OCH(CH_3)$_2$), 14.1 (C**9**).

The aromatic signals corresponding to the pentafluorobenzoyl group could not be resolved due to their weak intensity.

δ_F (377 MHz, $CDCl_3$) -136.3 – -136.7 (2F, m), -146.5 (1F, tt, *J* = 21.0, 5.0 Hz), -159.2 – -159.4 (2F, m).

HRMS: (ESI$^+$) Calculated for $C_{20}H_{24}F_5NNaO_4$: 460.1518. Found [M+Na]$^+$: 460.1512.

Isopropyl (*E*)-2-(pent-1-en-1-yl)pyrrolidine-1-carboxylate (4b)

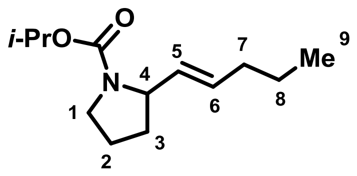

General procedure E: Conditions: 2.5 mol% $Pd_2(dba)_3$; 15 mol% PA-Ph (**L-1**); 100 mol% Et_3N; THF (0.4 M); 130 °C. Substrate **2b** (45.9 mg, 0.105 mmol) was employed. FCC (gradient elution: 29:1 – 19:1 PhMe:EtOAc) afforded **4b** (18.5 mg, 78 %) as a pale yellow oil.

ν_{max} / cm^{-1}: (*film*) 2959 (m), 2928 (m), 1697 (s), 1403 (s), 1113 (s).

δ_H (500 MHz, $CDCl_3$) 5.59 – 5.42 (1H, m C**6**-H), 5.41 – 5.26 (1H, m, C**5**-H), 4.92 (1H, hept, *J* = 6.5 Hz, OCH(CH_3)$_2$), 4.42 – 4.20 (1H, m, C**4**-H), 3.53 – 3.29 (2H, m, C**1**-H_2), 2.04 – 1.96 (3H, m, C**2**-H and C**7**-H_2), 1.92 – 1.79 (2H, m, C**3**-H_2), 1.73 – 1.67 (1H, m, C**2**-H'), 1.39 (2H, tq, *J* = 7.5, 7.5 Hz, C**8**-H_2), 1.27 – 1.18 (6H, m, OCH(CH_3)$_2$), 0.90 (3H, t, *J* = 7.5 Hz, C**9**-H_3).

δ_C (126 MHz, $CDCl_3$) 155.0 (C=O), 2 × 130.4 (C**5** and C**6**), 67.8 (OCH(CH_3)$_2$), 58.5 (C**4**), 46.2 (C**1**), 34.2 (C**7**), 32.4 (C**2**), 22.9 (C**3**), 2 × 22.4 (C**8** and OCH(CH_3)(CH_3)'), 22.3 (OCH(CH_3)(CH_3)'), 13.6 (C**9**).

HRMS: (ESI$^+$) Calculated for $C_{13}H_{23}NNaO_2$: 248.1621. Found [M+Na]$^+$: 248.1626.

Methyl (*E*)-non-4-en-1-yl((pentafluorobenzoyl)oxy)carbamate (2c)

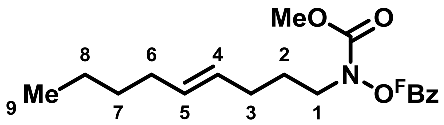

General procedure D: (*E*)-Non-4-en-1-ol (*vide supra*, 284 mg, 2.00 mmol) was employed with **1c**. The reaction time was 15 hours. FCC (*two times*, first eluent: 1:4 hexane:PhMe; second eluent: 24:1 hexane:EtOAc) afforded **2c** (413 mg, 50 %) as a colorless oil.

ν_{max} / cm^{-1}: (*film*) 2930 (m), 1786 (s), 1730 (s), 1652 (m), 1498 (s), 1326 (s), 1172 (s).

δ_H (400 MHz, $CDCl_3$) 5.48 – 5.31 (2H, m, C**4**-H and C**5**-H), 3.81 (3H, s, OCH_3), 3.74 – 3.69 (2H, m, C**1**-H_2), 2.07 (2H, dt, *J* = 7.0, 7.0 Hz, C**3**-H_2), 2.01 – 1.94 (2H, m, C**6**-H_2), 1.71 (2H, tt, *J* = 7.5, 7.0 Hz, C**2**-H_2), 1.35 – 1.26 (4H, m, C**7**-H_2 and C**8**-H_2), 0.91 – 0.85 (3H, m, C**9**-H_3).

δ_C (101 MHz, $CDCl_3$) 157.4 (^{F}Bz C=O), 156.2 (MeO–C=O), 132.0 (C**5**), 128.5 (C**4**), 54.0 (OCH_3), 50.9 (C**1**), 32.4 (C**6**), 31.8 (C**7**), 29.5 (C**3**), 26.8 (C**2**), 22.3 (C**8**), 14.1 (C**9**).

The aromatic signals corresponding to the pentafluorobenzoyl group could not be resolved due to their weak intensity.

δ_F (377 MHz, $CDCl_3$) -135.9 – -136.1 (2F, m), -146.1 (1F, tt, *J* = 20.5, 5.0 Hz), -159.2 – -159.4 (2F, m).

HRMS: (ESI$^+$) Calculated for $C_{18}H_{20}F_5NNaO_4$: 432.1205. Found $[M+Na]^+$: 432.1209.

Methyl (*E*)-2-(pent-1-en-1-yl)pyrrolidine-1-carboxylate (4c)

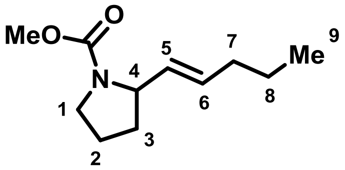

General procedure E: Conditions: 2.5 mol% $Pd_2(dba)_3$; 15 mol% PA-Ph (**L-1**); 100 mol% Et_3N; THF (0.4 M); 130 °C. Substrate **2c** (43.0 mg, 0.105 mmol) was employed. FCC (eluent: 19:1 PhMe:EtOAc) afforded **4c** (14.6 mg, 70 %) as a colorless oil.

ν_{max} / cm^{-1}: (*film*) 2956 (m), 2873 (m), 1699 (s), 1447 (s), 1381 (s).

^{1}H and ^{13}C spectra acquired at high temperature:

δ_H (500 MHz, CD_3CN, 65 °C) 5.57 – 5.48 (1H, m, C**6**-H), 5.43 (1H, ddt, *J* = 15.0, 6.0, 1.0 Hz, C**5**-H), 4.33 – 4.26 (1H, m, C**4**-H), 3.64 (3H, s, OCH_3), 3.42 – 3.37 (2H, m, C**1**-H_2), 2.08 – 1.99 (3H, m, C**3**-H and C**7**-H_2), 1.95 – 1.80 (2H, m, C**2**-H_2), 1.71 (1H, dddd, *J* = 12.0, 6.5, 4.0, 3.0 Hz, C**3**-H'), 1.43 (2H, tq, *J* = 7.5, 7.5 Hz, C**8**-H_2), 0.93 (3H, t, *J* = 7.5 Hz, C**9**-H_3).

δ_C (126 MHz, CD_3CN, 65 °C) 155.1 (C=O), 130.9 (C**5**), 129.9 (C**6**), 58.6 (C**4**), 51.2 (OCH_3), 46.2 (C**1**), 33.8 (C**7**), 31.8 (C**3**), 22.9 (C**2**), 22.2 (C**8**), 12.7 (C**9**).

HRMS: (ESI^+) Calculated for $C_{11}H_{19}NNaO_2$: 220.1308. Found $[M+Na]^+$: 220.1303.

Benzyl (*E*)-hex-4-en-1-yl((pentafluorobenzoyl)oxy)carbamate (2d)

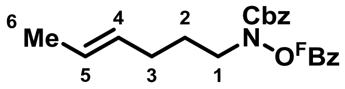

<u>General procedure D:</u> (*E*)-Hex-4-en-1-ol (0.33 mL, 2.80 mmol) was employed with **1d**. The reaction time was 16 hours. FCC (eluent: 2:3 hexane:PhMe) afforded **2d** (734 mg, 59 %) as a colorless oil.

ν_{max} / cm^{-1}: (*film*) 2940 (m), 1786 (s), 1730 (s), 1653 (m), 1500 (s), 1175 (s).

δ_H (400 MHz, $CDCl_3$) 7.39 – 7.30 (5H, m, ArCH), 5.50 – 5.33 (2H, m, C**4**-H and C**5**-H), 5.22 (2H, s, OCH_2Ph), 3.75 (2H, t, *J* = 7.0 Hz, C**1**-H_2), 2.06 (2H, td, *J* = 7.5, 6.5 Hz, C**3**-H_2), 1.71 (2H, tt, *J* = 7.5, 7.0 Hz, C**2**-H_2), 1.63 (3H, dq, *J* = 6.0, 1.0 Hz, C**6**-H_3).

δ_C (101 MHz, $CDCl_3$) 155.5 (Cbz C=O), 135.4 (ArC), 129.7 (C**4**), 128.7 (ArCH), 128.6 (ArCH), 128.2 (ArCH), 126.3 (C**5**), 68.9 (OCH_2Ph), 50.8 (C**1**), 29.4 (C**3**), 26.8 (C**2**), 18.0 (C**6**).

The signals corresponding to the pentafluorobenzoyl group could not be resolved due to their weak intensity.

δ_F (377 MHz, $CDCl_3$) -135.8 – -136.1 (2F, m), -145.9 – -146.1 (1F, m), -159.2 – -159.4 (2F, m).

HRMS: (ESI^+) Calculated for $C_{21}H_{18}F_5NNaO_4$: 466.1048. Found $[M+Na]^+$: 466.1050.

Benzyl 2-vinylpyrrolidine-1-carboxylate (4d)

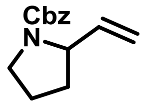

<u>General procedure E:</u> Conditions: 2.5 mol% $Pd_2(dba)_3$; 15 mol% PA-(4-(CF_3)C_6H_4) (**L-2**); 100 mol% Et_3N; THF (0.4 M); 130 °C. Substrate **2d** (46.6 mg, 0.105 mmol) was employed. FCC (eluent: 29:1 PhMe:EtOAc) afforded **4d** (18.9 mg, 78 %) as a colorless oil.

*When **2d** was heated in the absence of catalyst at 130 °C in THF for 24 hours no conversion to **4d** was observed.*

δ_H (500 MHz, $CDCl_3$) 7.41 – 7.27 (5H, m), 5.83 – 5.70 (1H, m), 5.19 – 4.98 (4H, m), 4.46 – 4.35 (1H, m), 3.53 – 3.39 (2H, m), 2.08 – 1.96 (1H, m), 1.94 – 1.79 (2H, m), 1.78 – 1.67 (1H, m).

This compound exists as an approximately 1:1 mixture of rotamers, this results in the doubling of several signals in the carbon spectrum.

δ_C (126 MHz, $CDCl_3$) 155.1 and 154.8, 138.5 and 138.0, 137.1, 128.4 and 128.3, 127.8, 127.7, 114.3 and 114.1, 66.6, 59.5 and 59.0, 46.7 and 46.4, 32.0 and 31.2, 23.5 and 22.6.

The spectroscopic properties were consistent with the data available in the literature.[6]

(*E*)-6-Phenylhex-4-en-1-ol

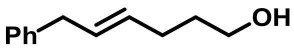

To a solution of Hoveyda-Grubbs 2nd generation catalyst (62.7 mg, 0.100 mmol) in anhydrous, CH_2Cl_2 (120 mL, sparged with argon) was added 4-penten-1-ol (1.03 mL, 10.0 mmol) and allyl benzene (13.2 mL, 100 mmol). The reaction mixture was heated under reflux for 14 hours before being concentrated *in vacuo* and purified by FCC (eluent: 5:1 toluene:EtOAc) to afford the title compound (835 mg, 47 %, 6:1 mixture of *E* and *Z* isomers) as a light brown oil (the coloration was due to the presence of trace amounts of Ru-impurities).

ν_{max} / cm^{-1}: (film) 3334 (s), 3026 (m), 2932 (m), 1603 (m), 1494 (s), 1452 (s), 1054 (s).

Spectroscopic data for the major E isomer:

δ_H (400 MHz, $CDCl_3$) 7.36 – 7.28 (2H, m), 7.25 – 7.18 (3H, m), 5.65 (1H, dt, *J* = 15.0, 6.5 Hz), 5.55 (1H, dt, *J* = 15.0, 7.0 Hz), 3.66 (2H, t, *J* = 6.5 Hz), 3.37 (2H, d, *J* = 6.5 Hz), 2.15 (2H, dt, *J* = 7.0, 6.5 Hz), 1.96 (1H, br s), 1.71 – 1.63 (2H, m).

δ_C (101 MHz, $CDCl_3$) 140.9, 131.1, 129.6, 128.5, 128.4, 125.6, 62.4, 39.1, 32.4, 28.8.

Characteristic signals for the minor Z isomer:

δ_H (400 MHz, $CDCl_3$) 3.45 (2H, d, *J* = 7.0 Hz), 2.28 (1H, dt, *J* = 7.0, 7.0 Hz).

The spectroscopic properties were consistent with the data available in the literature.[7,8]

***tert*-Butyl (*E*)-(6-phenylhex-4-en-1-yl)((pentafluorobenzoyl)oxy)carbamate (2e)**

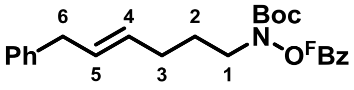

General procedure D: The preceding alcohol (824 mg, 4.67 mmol) was employed with **1a**. The reaction time was 16 hours. FCC (eluent: 1:4 hexane:PhMe) afforded **2e** (1.18 g, 52 %, 6:1 mixture of *E* and *Z* isomers) as a colorless oil.

ν_{max} / cm^{-1}: (*film*) 2938 (m), 1784 (s), 1722 (s), 1653 (m), 1506 (s), 1327 (s), 1154 (s).

Spectroscopic data for the major E isomer:

δ_H (400 MHz, $CDCl_3$) 7.30 – 7.25 (2H, m, ArC$\underline{H}$), 7.21 – 7.15 (3H, m, ArC$\underline{H}$), 5.66 – 5.57 (1H, m, C**5**-$\underline{H}$), 5.54 – 5.45 (1H, m, C**4**-$\underline{H}$), 3.68 (2H, t, *J* = 7.0 Hz, C**1**-$\underline{H}_2$), 3.33 (2H, d, *J* = 6.5 Hz, C**6**-$\underline{H}_2$), 2.12 (2H, dt, *J* = 7.5, 7.5 Hz, C**3**-$\underline{H}_2$), 1.73 (2H, tt, *J* = 7.5, 7.0 Hz, C**2**-$\underline{H}_2$), 1.49 (9H, s, OC(C$\underline{H}_3$)$_3$).

δ_C (101 MHz, $CDCl_3$) 154.7 (Boc C=O), 140.9 (ArC), 130.4 (C**4**), 130.2 (C**5**), 128.6 (ArCH), 128.5 (ArCH), 126.1 (ArCH), 83.4 (OC(CH_3)$_3$), 50.6 (C**1**), 39.1 (C**6**), 29.5 (C**3**), 28.2 (OC(CH_3)), 26.8 (C**2**).

The signals corresponding to the pentafluorobenzoyl group could not be resolved due to their weak intensity.

δ_F (377 MHz, $CDCl_3$) -136.4 – -136.7 (2F, m), -146.7 (1F, tt, *J* = 21.0, 5.0 Hz), -159.3 – -159.5 (2F, m).

Characteristic signals for the minor Z isomer:

δ_H (400 MHz, $CDCl_3$) 3.40 (2H, d, *J* = 7.5 Hz), 2.26 (2H, dt, *J* = 7.0, 7.0 Hz).

HRMS: (ESI^+) Calculated for $C_{24}H_{24}F_5NNaO_4$: 508.1518. Found $[M+Na]^+$: 508.1525.

***tert*-Butyl (*E*)-2-styrylpyrrolidine-1-carboxylate (4e)**

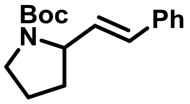

General procedure E: Conditions: 2.5 mol% $Pd_2(dba)_3$; 15 mol% PA-(4-(CF_3)C_6H_4) (**L-2**); 100 mol% Et_3N; THF (0.4 M); 130 °C. Substrate **2e** (51.0 mg, 0.105 mmol) was employed. FCC (gradient elution: 99:1 – 49:1 – 24:1 PhMe:EtOAc) afforded **4e** (22.3 mg, 78 %) as a yellow crystalline solid.

δ_H (400 MHz, $CDCl_3$) 7.38 – 7.27 (4H, m), 7.25 – 7.16 (1H, m), 6.40 (1H, br d, *J* = 15.5 Hz), 6.18 – 6.01 (1H, m), 4.61 – 4.30 (1H, m), 3.46 (2H, br s), 2.16 – 2.01 (1H, m), 1.99 – 1.73 (3H, m), 1.43 (9H, s).

δ_C (126 MHz, $CDCl_3$) 154.8, 137.2, 130.9, 129.5, 128.6, 127.4, 126.4, 79.3, 59.1, 46.4, 32.7, 28.7, 23.2.

The spectroscopic properties were consistent with the data available in the literature.[9]

(*E*)-Hex-4-enal

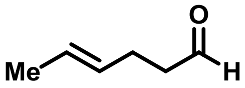

This compound was prepared according to a literature procedure.[10]

δ_H (400 MHz, $CDCl_3$) 9.77 (1H, t, *J* = 1.5 Hz), 5.57 – 5.36 (2H, m), 2.52 – 2.47 (2H, m), 2.33 (2H, q, *J* = 7.0 Hz), 1.65 (3H, d, *J* = 5.0 Hz).

δ_C (101 MHz, $CDCl_3$) 202.3, 128.8, 126.3, 43.4, 25.1, 17.8.

The spectroscopic properties were consistent with the data available in the literature.[10]

(*E*)-1-Phenylhex-4-en-1-ol

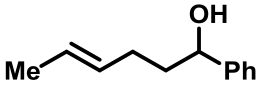

To a solution of phenyl magnesium bromide (16.0 mmol) in anhydrous Et_2O (26 mL) at 0 °C was added a solution of the preceding aldehyde (981 mg, 10.0 mmol) in anhydrous Et_2O (5 mL). The reaction mixture was stirred for 2 hours before addition of saturated aqueous NH_4Cl (15 mL). The resulting phases were separated and the aqueous phase was extracted with Et_2O (2 × 20 mL). The organic phase was dried over Na_2SO_4 and concentrated *in vacuo*. FCC (eluent: 7:1 hexane:EtOAc) afforded the title compound (1.33 g, 75 %) as a pale yellow oil.

δ_H (400 MHz, $CDCl_3$) 7.37 – 7.32 (4H, m), 7.31 – 7.24 (1H, m), 5.52 – 5.39 (2H, m), 4.68 (1H, ddd, *J* = 7.5, 5.5, 3.5 Hz), 2.17 – 1.99 (2H, m), 1.98 – 1.94 (m, 1H), 1.91 – 1.71 (2H, m), 1.67 – 1.63 (3H, m).

δ_C (101 MHz, $CDCl_3$) 144.8, 130.7, 128.6, 127.6, 126.0, 125.7, 74.2, 38.9, 29.1, 18.1.

HRMS: (ESI^+) Calculated for $C_{12}H_{16}NaO$: 199.1093. Found $[M+Na]^+$: 199.1087.

The spectroscopic properties were consistent with the data available in the literature.[11]

***tert*-Butyl (*E*)-(1-phenylhex-4-en-1-yl)((pentafluorobenzoyl)oxy)carbamate (2f)**

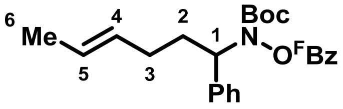

General procedure D: The preceding alcohol (176 mg, 1.00 mmol) was employed with **1a** and the reaction was performed at -78 °C. The reaction time was 18 hours. FCC (eluent: 2:3 hexane:PhMe) afforded impure material which was dissolved in Et_2O (15 mL) and washed with 10 % aqueous AcOH (2 × 10 mL). The Et_2O phase was dried over Na_2SO_4 and concentrated *in vacuo*. FCC (eluent: 19:1 hexane:EtOAc) afforded **2f** (248 mg, 51 %) as a colorless oil.

ν_{max} / cm^{-1}: (*film*) 2981 (m), 1787 (s), 1720 (s), 1652 (m), 1506 (s), 1326 (s), 1158 (s).

δ_H (500 MHz, $CDCl_3$) 7.43 – 7.37 (2H, m, ArC<u>H</u>), 7.37 – 7.29 (3H, m, ArC<u>H</u>), 5.52 – 5.40 (2H, m, C**4**-<u>H</u> and C**5**-<u>H</u>), 5.38 – 5.21 (1H, m, C**1**-<u>H</u>), 2.23 – 2.04 (3H, m, C**2**-<u>H</u> and C**3**-<u>H</u>$_2$), 1.99 (1H, br s, C**2**-<u>H</u>'), 1.68 (2H, d, *J* = 4.5 Hz, C**6**-<u>H</u>$_3$), 1.56 – 1.36 (9H, br s, OC(C<u>H</u>$_3$)$_3$).

δ_C (126 MHz, $CDCl_3$) 157.0 (^{F}Bz <u>C</u>=O), 154.4 (Boc <u>C</u>=O), 145.4 (d, *J* = 261.0 Hz, Ar<u>C</u>F), 143.7 (d, *J* = 261.0 Hz, Ar<u>C</u>F), 138.8 (Ar<u>C</u>), 137.8 (d, *J* = 255.0 Hz, Ar<u>C</u>F), 129.7 (C**4** or C**5**), 128.4 (Ar<u>C</u>H), 128.0 (Ar<u>C</u>H), 127.9 (Ar<u>C</u>H), 126.3 (C**4** or C**5**), 106.1 (m, Ar<u>C</u>), 83.5 (O<u>C</u>(CH$_3$)$_3$), 63.1 (C**1**), 31.5 (C**2**), 29.2 (C**3**), 28.0 (OC(<u>C</u>H$_3$)$_3$), 17.9 (C**6**).

δ_F (377 MHz, $CDCl_3$) -136.0 – -136.7 (2F, m), -147.0 (1F, tt, *J* = 21.0, 5.0Hz), -159.2 – -159.9 (2F, m).

HRMS: (ESI^+) Calculated for $C_{24}H_{24}F_5NNaO_4$: 508.1518. Found $[M+Na]^+$: 508.1516.

tert-Butyl (2S*,5R*)-2-phenyl-5-vinylpyrrolidine-1-carboxylate (4f)

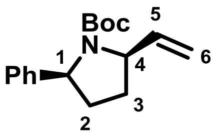

General procedure E: Conditions: 2.5 mol% $Pd_2(dba)_3$; 17.5 mol% PA-(4-(CO_2Et)C_6H_4) (**L-3**); 100 mol% Et_3N; dioxane (0.3 M); 130 °C. Substrate **2f** (51.0 mg, 0.105 mmol) was employed. FCC (*two times*, first eluent: 59:1 petroleum ether:acetone; second eluent: 99:1 PhMe:EtOAc) afforded **4f** (16.6 mg, 58 %, 10:1 mixture of *cis* and *trans* diastereomers) as a colorless oil.

*It was not possible to determine the relative stereochemistry of **4f** by NOE analysis. The removal of the Boc group was necessary to obtain satisfactory data, details of this are given below.*

ν_{max} / cm^{-1}: (*film*) 2974 (m), 1693 (s), 1380 (s), 1165 (s).

Spectroscopic data for the major cis diastereomer:

δ_H (500 MHz, $CDCl_3$) 7.34 – 7.27 (2H, m, ArCH), 7.27 – 7.24 (2H, m, ArCH), 7.23 – 7.18 (1H, m, ArCH), 6.13 – 5.93 (1H, m, C**5**-H), 5.34 – 5.14 (2H, m, C**6**-H_2), 4.93 – 4.70 (1H, m, C**1**-H), 4.55 – 4.34 (1H, m, C**4**-H), 2.26 (1H, dddd, *J* = 12.5, 6.5, 6.0, 6.0 Hz, C**2**-H), 2.06 (1H, dddd, *J* = 12.0, 8.0, 7.5, 6.5 Hz, C**3**-H), 1.88 (1H, dddd, *J*=12.5, 7.5, 6.5, 6.5 Hz, C**2**-H'), 1.84 – 1.75 (1H, m, C**3**-H'), 1.47 – 1.10 (9H, m, OC(CH_3)$_3$).

δ_C (126 MHz, $CDCl_3$) 155.1 (C=O), 144.6 (ArC), 139.5 (C**5**), 128.3 (ArCH), 126.6 (ArCH), 126.0 (ArCH), 115.3 (C**6**), 79.6 (OC(CH_3)$_3$), 63.1 (C**1**), 61.2 (C**4**), 34.9 (C**2**), 30.7 (C**3**), 28.4 (OC(CH_3)$_3$).

Characteristic signals for the minor trans diastereomer:

δ_H (500 MHz, $CDCl_3$) 5.91 – 5.78 (1H, m), 2.37 (1H, dddd, *J* = 13.5, 11.5, 6.0, 5.5 Hz), 2.18 (1H, dddd, *J* = 13.0, 8.5, 6.0, 6.0 Hz).

HRMS: (ESI$^+$) Calculated for $C_{17}H_{23}NNaO_2$: 296.1621. Found $[M+Na]^+$: 296.1630.

(2S*,5R*)-2-Phenyl-5-vinylpyrrolidin-1-ium trifluoroacetate

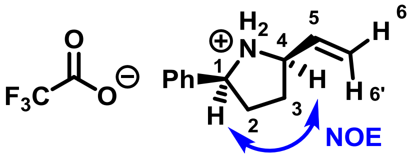

General procedure F: Pyrrolidine **4f** (15.7 mg, 57.4 μmol) was employed to afford the title compound (15.7 mg, 95 %, 14:1 mixture of *cis* and *trans* diastereomers) as a pale yellow crystalline solid.

*The major product was assigned as the cis diastereomer based on the observed NOE correlation between the C**1** and the C**4** protons.*

ν_{max} / cm^{-1}: (*film*) 2924 (m), 1666 (s), 1432 (s), 1130 (s).

Spectroscopic data for the major cis diastereomer:

δ_H (500 MHz, $CDCl_3$) 10.52 (1H, br s, N<u>H</u>), 8.71 (1H, br s, N<u>H</u>’), 5.72 (1H, ddd, *J* = 17.0, 10.5, 8.0 Hz, C**5**-<u>H</u>), 5.19 (1H, d, *J* = 17.0 Hz, C**6**-<u>H</u>’), 4.99 (1H, d, *J* = 10.5 Hz, C**6**-<u>H</u>), 4.57 – 4.48 (1H, m, C**1**-<u>H</u>), 4.10 – 3.99 (1H, m, C**4**-<u>H</u>), 2.45 – 2.24 (3H, m, C**2**-$\underline{H}_2$ and C**3**-<u>H</u>), 2.16 – 2.09 (1H, m, C**3**-<u>H</u>’).

δ_C (101 MHz, $CDCl_3$) 133.9 (Ar<u>C</u>), 131.7 (C**5**), 129.3 (Ar<u>C</u>H), 128.9 (Ar<u>C</u>H), 127.7 (Ar<u>C</u>H), 121.4 (C**6**), 63.1 (C**1**), 62.7 (C**4**), 29.9 (C**2**), 29.2 (C**3**).

The signals corresponding to the trifluoroacetate counterion could not be resolved due to their weak intensity.

δ_F (377 MHz, $CDCl_3$) -75.8 (3F, s).

Characteristic signals for minor trans diastereomer:

δ_H (500 MHz, $CDCl_3$) 9.87 (1H, br s), 9.17 (1H, br s), 5.11 (1H, d, *J* = 10.5 Hz), 4.22 – 4.13 (1H, m).

HRMS: (ESI^+) Calculated for $C_{12}H_{16}N$: 174.1277. Found $[M–F_3CCO_2]^+$: 174.1280.

Methyl (*E*)-2-phenylhex-4-enoate

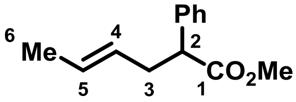

To a suspension of NaH (60 % weight in mineral oil, 852 mg, 21.3 mmol) in anhydrous THF (25 mL) and DMF (25 mL) was added methyl phenylacetate (3.00 mL, 21.3 mmol). The reaction mixture was stirred at room temperature for 1.5 hours before addition of crotyl bromide (1.46 mL, 14.2 mmol). After two hours brine (50 mL) was added, the resulting phases were separated and the aqueous phase was extracted with Et_2O (3 × 60 mL). The organic phase was dried over Na_2SO_4 and concentrated *in vacuo*. FCC (eluent: 39:1 hexane:EtOAc) to afford the title compound (2.38 g, 82 %) as a colorless oil.

ν_{max} / cm^{-1}: (film) 3029 (m), 2951 (m), 1734 (s), 1435 (s), 1160 (s).

δ_H (400 MHz, $CDCl_3$) 7.35 – 7.28 (4H, m, ArC<u>H</u>), 7.28 – 7.23 (1H, m, ArC<u>H</u>), 5.50 (1H, dqdd, *J* = 15.0, 6.5, 1.5, 1.0 Hz, C**5**-<u>H</u>), 5.40 – 5.28 (1H, m, C**4**-<u>H</u>), 3.65 (3H, s, OC$\underline{H}_3$), 3.59 (1H, dd, *J* = 8.5, 6.5 Hz, C**2**-<u>H</u>), 2.81 – 2.71 (1H, m, C**3**-<u>H</u>), 2.48 – 2.39 (1H, m, C**3**-<u>H</u>’), 1.61 (3H, dd, *J* = 6.5, 1.5 Hz, C**6**-$\underline{H}_3$).

δ_C (101 MHz, $CDCl_3$) 174.0 (C**1**), 138.8 (Ar<u>C</u>), 128.6 (Ar<u>C</u>H), 127.9 (Ar<u>C</u>H), 127.7 (C**4**), 127.6 (C**5**), 127.2 (Ar<u>C</u>H), 52.0 (C**2**), 51.9 (O$\underline{C}H_3$), 36.6 (C**3**), 17.9 (C**6**).

HRMS: (ESI^+) Calculated for $C_{13}H_{16}NaO_2$: 227.1043. Found $[M+Na]^+$: 227.1044.

(*E*)-2-Phenylhex-4-en-1-ol

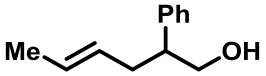

General procedure C: The preceding ester (2.05 g, 10.0 mmol) was employed using anhydrous THF as the solvent and 0.6 eq. $LiAlH_4$ (1.0 M in THF) to afford the title compound (1.57 g, 89 %) as a colorless oil.

δ_H (500 MHz, $CDCl_3$) 7.35 – 7.31 (2H, m), 7.26 – 7.20 (3H, m), 5.46 (1H, dqdd, *J* = 15.0, 6.5, 1.0, 1.0 Hz), 5.35 (1H, dddq, *J* = 15.0, 7.5, 6.0, 1.5 Hz), 3.80 (1H, dd, *J* = 11.0, 5.5 Hz), 3.73 (1H, dd, *J* = 11.0, 7.5 Hz), 2.84 (1H, dddd, *J* = 7.5, 7.5, 7.5, 5.5 Hz), 2.40 (1H, ddddq, *J* = 14.0, 7.5, 7.5, 1.0, 1.0 Hz), 2.37 – 2.27 (1H, m), 1.61 (3H, dddd, *J* = 6.5, 1.5, 1.5, 1.0 Hz), 1.36 (1H, br s).

δ_C (126 MHz, $CDCl_3$) 142.4, 128.9, 128.8, 128.2, 127.1, 126.9, 67.1, 48.7, 35.7, 18.1.

The spectroscopic properties were consistent with the data available in the literature.[12]

***tert*-Butyl (*E*)-(2-phenylhex-4-en-1-yl)((pentafluorobenzoyl)oxy)carbamate (2g)**

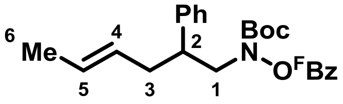

General procedure D: The preceding alcohol (441 mg, 2.50 mmol) was employed with **1a**. The reaction time was 15 hours. FCC (*two times*, first eluent: 2:3 hexane:PhMe; second eluent: 29:1 hexane:EtOAc) afforded **2g** (353 mg, 29 %) as a pale yellow oil.

ν_{max} / cm^{-1}: (*film*) 3026 (m), 2981 (m), 1783 (s), 1721 (s), 1652 (m), 1497 (s), 1153 (s).

δ_H (400 MHz, $CDCl_3$) 7.30 – 7.24 (2H, m, ArC<u>H</u>), 7.22 – 7.13 (3H, m, ArC<u>H</u>), 5.49 – 5.38 (1H, m, C**5**-<u>H</u>), 5.32 – 5.22 (1H, m, C**4**-<u>H</u>), 3.97 (1H, dd, *J* = 15.0, 7.0 Hz, C**1**-<u>H</u>), 3.84 (1H, dd, *J* = 15.0, 8.0 Hz, C**1**-<u>H</u>'), 3.00 (1H, dtd, *J* = 8.0, 7.5, 7.0 Hz, C**2**-<u>H</u>), 2.50 – 2.31 (2H, m, C**3**-$\underline{H_2}$), 1.57 (3H, dd, *J* = 6.5, 1.5 Hz, C**6**-$\underline{H_3}$), 1.39 (9H, s, $OC(C\underline{H}_3)_3$).

δ_C (101 MHz, $CDCl_3$) 157.1 (^{F}Bz <u>C</u>=O), 154.0 (Boc <u>C</u>=O), 141.9 (Ar<u>C</u>), 128.3 (Ar<u>C</u>H), 2 × 128.0 (C4 and Ar<u>C</u>H), 127.4 (C**5**), 126.5 (Ar<u>C</u>H), 83.1 ($O\underline{C}(CH_3)_3$), 55.6 (C**1**), 44.1 (C**2**), 36.5 (C**3**), 27.9 ($OC(\underline{C}H_3)_3$), 17.9 (C**6**).

The aromatic signals corresponding to the pentafluorobenzoyl group could not be resolved due to their weak intensity.

δ_F (377 MHz, $CDCl_3$) -136.0 – -136.4 (2F, m), -146.6 (1F, tt, *J* = 21.0, 5.0 Hz), -159.5 – -159.7 (2F, m).

HRMS: (ESI^+) Calculated for $C_{24}H_{24}F_5NNaO_4$: 508.1518. Found $[M+Na]^+$: 508.1514.

tert-Butyl (2_R_*,4_S_*)-4-phenyl-2-vinylpyrrolidine-1-carboxylate (4g)

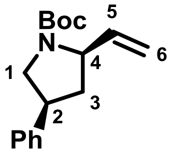

General procedure E: Conditions: 2.5 mol% $Pd_2(dba)_3$; 15 mol% PA-Ph (**L-1**); 100 mol% Et_3N; THF (0.4 M); 130 °C. Substrate **2g** (51.0 mg, 0.105 mmol) was employed. FCC (gradient elution: 12:1 – 9:1 hexane:EtOAc) afforded **4g** (25.3 mg, 88 %, 4:1 mixture of *cis* and *trans* diastereomers) as a pale yellow oil.

*When **2g** was heated in the absence of catalyst at 130 °C in THF for 24 hours no conversion to **4g** was observed. It was not possible to determine the relative stereochemistry of **4g** by NOE analysis. The removal of the Boc group was necessary to obtain satisfactory data, details of this are given below.*

ν_{max} / cm^{-1}: (*film*) 2975 (m), 2928 (m), 1689 (s), 1390 (s), 1164 (s).

Spectroscopic data for the major cis diastereomer:

δ_H (500 MHz, $CDCl_3$) 7.38 – 7.32 (2H, m, ArC<u>H</u>), 7.28 – 7.24 (3H, m, ArC<u>H</u>), 5.96 – 5.73 (1H, m, C**5**-<u>H</u>), 5.29 – 5.04 (2H, m, C**6**-<u>H</u>$_2$), 4.42 – 4.24 (1H, m, C**4**-<u>H</u>), 4.20 – 3.94 (1H, m, C**1**-<u>H</u>), 3.34 – 3.25 (2H, m, C**1**-<u>H</u>' and C**2**-<u>H</u>), 2.59 – 2.48 (1H, m, C**3**-<u>H</u>), 1.88 (1H, ddd, J = 12.0, 11.0, 11.0 Hz, C**3**-<u>H</u>'), 1.48 (9H, s, OC(C<u>H</u>$_3$)$_3$).

Although not observable in the ^{1}H spectrum, from the ^{13}C spectrum it is apparent that this compound exists as an approximately 3:2 mixture of rotamers A and B.

δ_C (126 MHz, $CDCl_3$) 154.9 (A: <u>C</u>=O), 154.6 (B: <u>C</u>=O), 140.6 (A and B: Ar<u>C</u>), 140.2 (A: C**5**), 139.7 (B: C**5**), 128.7 (A and B: Ar<u>C</u>H), 127.3 (A and B: Ar<u>C</u>H), 127.0 (A and B: Ar<u>C</u>H), 114.4 (A and B: C**6**), 79.7 (A and B: O<u>C</u>(CH$_3$)$_3$), 60.5 (A and B: C**4**), 53.8 (B: C**1**), 52.9 (A: C**1**), 43.3 (B: C**2**), 42.8 (A: C**2**), 41.1 (A: C**3**), 40.7 (B: C**3**), 28.6 (A and B: OC(<u>C</u>H$_3$)$_3$).

Characteristic signals for the minor trans diastereomer:

δ_H (500 MHz, $CDCl_3$) 3.93 – 3.80 (1H, m), 2.26 – 2.17 (1H, m), 2.13 – 2.04 (1H, m).

HRMS: (ESI$^+$) Calculated for $C_{17}H_{23}NNaO_2$: 296.1621. Found [M+Na]$^+$: 296.1632.

(2_R_*,4_S_*)-4-Phenyl-2-vinylpyrrolidin-1-ium trifluoroacetate

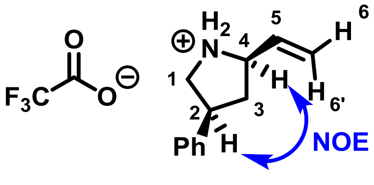

General procedure F: Pyrrolidine **4g** (25.3 mg, 92.5 μmol) was employed to afford the title compound (24.9 mg, 94 %, 4:1 mixture of *cis* and *trans* diastereomers) as an orange oil.

*The major product was assigned as the cis diastereomer based on the observed NOE correlation between the C**2** and the C**4** protons.*

ν_{max} / cm^{-1}: (*film*) 3404 (br s), 2979 (m), 1668 (s), 1429 (m), 1129 (s).

Spectroscopic data for the major cis diastereomer:

δ_H (400 MHz, $CDCl_3$) 10.01 (1H, br s, NH), 9.45 (1H, br s, NH'), 7.38 – 7.31 (2H, m, ArCH), 7.31 – 7.22 (3H, m, ArCH), 6.00 (1H, ddd, *J* = 17.5, 10.5, 8.0 Hz, C**5**-H), 5.46 (1H, d, *J* = 17.5 Hz, C**6**-H'), 5.36 (1H, d, *J* = 10.5 Hz, C**6**-H), 4.26 – 4.11 (1H, m, C**4**-H), 3.80 – 3.65 (1H, m, C**1**-H), 3.64 – 3.50 (1H, m, C**2**-H), 3.40 – 3.22 (1H, m, C**1**-H'), 2.50 (1H, ddd, *J* = 12.5, 6.0, 6.0 Hz, C**3**-H), 2.10 (1H, ddd, *J* = 12.5, 12.0, 12.0 Hz, C**3**-H').

δ_C (101 MHz, $CDCl_3$) 138.1 (ArC), 131.7 (C**5**), 129.0 (ArCH), 127.7 (ArCH), 127.1 (ArCH), 122.1 (C**6**), 62.8 (C**4**), 50.6 (C**1**), 43.7 (C**2**), 39.3 (C**3**).

The signals corresponding to the trifluoroacetate counterion could not be resolved due to their weak intensity.

δ_F (377 MHz, $CDCl_3$) -75.6 (3F, s).

Characteristic signals for the minor trans diastereomer:

δ_H (400 MHz, $CDCl_3$) 10.13 (1H, br s), 9.34 (1H, br s), 5.44 (1H, d, *J* = 17.0 Hz), 4.41 – 4.29 (1H, m), 2.41 – 2.26 (2H, m).

δ_C (101 MHz, $CDCl_3$) 61.6, 51.1, 42.2, 38.3.

HRMS: (ESI^+) Calculated for $C_{12}H_{16}N$: 174.1277. Found $[M–F_3CCO_2]^+$: 174.1281.

(*E*)-4-Phenylbut-3-en-2-ol

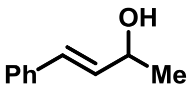

To a solution of cinnamaldehyde (10.0 g, 75.6 mmol) in anhydrous Et_2O (*approx.* 250 mL) at 0 °C was added MeLi (1.6 M in Et_2O, 56.7 mL, 90.7 mmol). The reaction mixture was stirred for 2 hours at room temperature before addition of water (10 mL) followed by saturated aqueous NH_4Cl (150 mL). The resulting phases were separated and the aqueous phase was extracted with Et_2O (2 × 100 mL). The organic phase was dried over Na_2SO_4 and concentrated *in vacuo*. FCC (eluent: 4:1 hexane:EtOAc) afforded the title compound (8.87 g, 79 %) as a yellow oil.

δ_H (400 MHz, $CDCl_3$) 7.39 – 7.34 (2H, m), 7.33 – 7.28 (2H, m), 7.26 – 7.20 (1H, m), 6.55 (1H, d, *J* = 16.0 Hz), 6.25 (1H, dd, *J* = 16.0, 6.5 Hz), 4.47 (1H, dq, *J* = 6.5, 6.5 Hz), 2.16 (1H, br s), 1.36 (3H, d, *J* = 6.5 Hz).

δ_C (101 MHz, $CDCl_3$) 136.8, 133.7, 129.4, 128.6, 127.7, 126.5, 68.9, 23.5.

The spectroscopic properties were consistent with the data available in the literature.[13]

Ethyl (*E*)-3-phenylhex-4-enoate

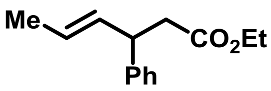

General procedure B: The preceding allylic alcohol (1.39 g, 9.38 mmol) was employed. The reaction time was 15 hours. FCC (eluent: 49:1 hexane:EtOAc) afforded the title compound (1.61 g, 79 %) as a colorless oil.

δ_H (500 MHz, $CDCl_3$) 7.32 – 7.27 (2H, m), 7.22 – 7.18 (3H, m), 5.60 (1H, ddq, *J* = 15.0, 7.5, 1.5 Hz), 5.50 (1H, dqd, *J* = 15.0, 6.5, 1.0 Hz), 4.10 – 4.04 (2H, m), 3.81 (1H, br dt, *J* = 7.5, 7.5 Hz), 2.72 – 2.67 (2H, m), 1.66 (3H, ddd, *J* = 6.5, 1.5, 1.0 Hz), 1.17 (3H, t, *J* = 7.0 Hz).

δ_C (126 MHz, $CDCl_3$) 172.1, 143.5, 133.3, 128.6, 127.6, 126.6, 125.8, 60.4, 45.1, 41.2, 18.1, 14.3.

The spectroscopic properties were consistent with the data available in the literature.[14]

(*E*)-3-Phenylhex-4-en-1-ol

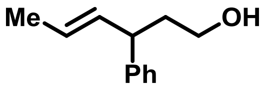

General procedure C: The preceding ester (1.61 g, 7.34 mmol) was employed using anhydrous THF as the solvent and 0.8 eq. $LiAlH_4$ (1.0 M in THF). FCC (eluent: 3:1 hexane:EtOAc) afforded **40** (821 mg, 63 %) as a colorless oil.

δ_H (400 MHz, $CDCl_3$) 7.33 – 7.28 (2H, m), 7.23 – 7.17 (3H, m), 5.60 (1H, ddq, *J* = 15.0, 8.0, 1.5 Hz), 5.56 – 5.46 (1H, m), 3.67 – 3.57 (2H, m), 3.41 (1H, br dt, *J* = 8.0, 8.0 Hz), 2.00 – 1.90 (2H, m), 1.68 (3H, ddd, *J* = 5.5, 1.5, 1.0 Hz), 1.42 (1H, br s).

δ_C (101 MHz, $CDCl_3$) 144.8, 134.8, 128.7, 127.6, 126.3, 125.3, 61.3, 45.6, 38.8, 18.1.

The spectroscopic properties were consistent with the data available in the literature.[15]

Methyl (*E*)-(3-phenylhex-4-en-1-yl)((pentafluorobenzoyl)oxy)carbamate (2h)

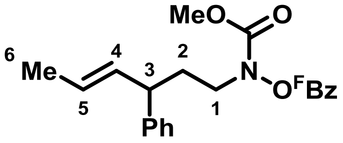

General procedure D: The preceding alcohol (353 mg, 2.00 mmol) was employed with **1c**. The reaction time was 16 hours. FCC (*two times*, first eluent: 1:4 petroleum ether:PhMe; second eluent: 19:1 petroleum ether:acetone) afforded **2h** (514 mg, 58 %) as a colorless oil.

ν_{max} / cm^{-1}: (*film*) 3030 (m), 2959 (m), 1784 (s), 1731 (s), 1652 (m), 1497 (s), 1173 (s).

δ_H (400 MHz, $CDCl_3$) 7.32 – 7.27 (2H, m, ArCH), 7.22 – 7.16 (3H, m, ArCH), 5.60 – 5.44 (2H, m, C**4**-H and C**5**-H), 3.79 (3H, s, OCH3), 3.75 – 3.61 (2H, m, C**1**-H2), 3.35 (1H, dt, *J* = 7.5, 7.5 Hz, C**3**-H), 2.02 (2H, dt, *J* = 7.5, 7.5 Hz, C**2**-H2), 1.67 (3H, dd, *J* = 5.5, 1.0 Hz, C**6**-H3).

δ_C (101 MHz, $CDCl_3$) 156.2 (MeO–C=O), 144.1 (ArC), 133.8 (C**4**), 128.7 (ArCH), 127.5 (ArCH), 126.5 (ArCH), 126.0 (C**5**), 54.1 (OCH3), 49.9 (C**1**), 46.1 (C**3**), 32.9 (C**2**), 18.1 (C**6**).

The signals corresponding to the pentafluorobenzoyl group could not be resolved due to their weak intensity.

δ_F (377 MHz, $CDCl_3$) -135.9 – -136.1 (2F, m), -146.1 (1F, tt, *J* = 21.0, 5.5 Hz), -159.2 – -159.5 (2F, m).

HRMS: (ESI$^+$) Calculated for $C_{21}H_{18}F_5NNaO_4$: 466.1048. Found [M+Na]$^+$: 466.1049.

Methyl (2*S,3*S**)-3-phenyl-2-vinylpyrrolidine-1-carboxylate (4h)**

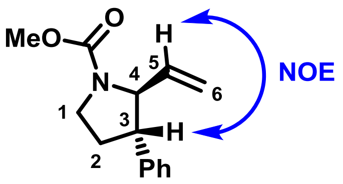

General procedure E: Conditions: 2.5 mol% $Pd_2(dba)_3$; 17.5 mol% PA-(4-(CO_2Et)C_6H_4) (**L-3**); 100 mol% Et_3N; dioxane (0.3 M); 130 °C. Substrate **2h** (46.6 mg, 0.105 mmol) was employed. FCC (eluent: 7:1 petroleum ether:EtOAc) afforded **4h** (18.8 mg, 77 %) as a colorless oil.

*The product was assigned as the trans diastereomer based on the observed NOE correlation between the C**3** and the C**5** protons.*

ν_{max} / cm^{-1}: (*film*) 3030 (m), 2953 (m), 1701 (s), 1448 (s), 1383 (s).

δ_H (500 MHz, $CDCl_3$) 7.34 – 7.30 (2H, m, ArCH), 7.26 – 7.22 (1H, m, ArCH), 7.22 – 7.18 (2H, m, ArCH), 5.83 (1H, ddd, *J* = 17.0, 10.5, 6.0 Hz, C**5**-H), 5.17 – 4.94 (2H, m, C**6**-H2), 4.45 – 4.25 (1H, m, C**4**-H), 3.84 – 3.64 (4H, m, C**1**-H and OCH3), 3.56 – 3.44 (1H, m, C**1**-H'), 3.13 (1H, ddd, *J* = 6.5, 6.5, 6.5 Hz, C**3**-H), 2.27 (1H, dddd, *J* = 13.5, 6.5, 6.5, 6.5 Hz, C**2**-H), 1.98 (1H, dddd, *J* = 13.5, 7.0, 7.0, 6.5 Hz, C**2**-H').

This compound exists as an approximately 1:1 mixture of rotamers, this results in in the doubling of several signals in the carbon spectrum.

δ_C (126 MHz, $CDCl_3$) 155.9 and 155.6 (C=O), 142.1 and 141.9 (ArC), 138.2 and 137.7 (C**5**), 128.8 (ArCH), 127.3 (ArCH), 127.0 (ArCH), 115.4 and 114.7 (C**6**), 66.3 (C**4**), 52.5 (OCH_3), 51.2 and 50.4 (C**3**), 46.3 and 46.0 (C**1**), 32.3 and 31.4 (C**2**).

HRMS: (ESI^+) Calculated for $C_{14}H_{18}NO_2$: 232.1332. Found $[M+H]^+$: 232.1332.

2-Benzylacrylaldehyde

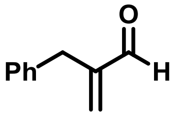

This compound was prepared according to a literature procedure.[16]

ν_{max} / cm^{-1}: (film) 3063 (m), 3029 (m), 2918 (br m), 2827 (br m), 1688 (s), 1496 (s), 1454 (s).

δ_H (400 MHz, $CDCl_3$) 9.61 (1H, s), 7.34 – 7.28 (2H, m), 7.25 – 7.22 (1H, m), 7.22 – 7.16 (2H, m), 6.11 (1H, td, J = 1.5, 1.0 Hz), 6.07 (1H, dt, J = 1.0, 1.0 Hz), 3.58 – 3.57 (2H, m).

δ_C (101 MHz, $CDCl_3$) 194.1, 149.8, 138.2, 135.3, 129.3, 128.7, 126.6, 34.3.

The spectroscopic properties were consistent with the data available in the literature.[16]

3-Benzylbut-3-en-2-ol

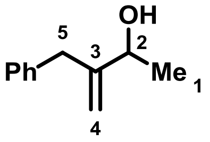

To a solution of MeLi (1.6 M in Et_2O, 16.0 mL, 25.6 mmol) in anhydrous THF (20 mL) at 0 °C was added a solution of the preceding aldehyde (2.50 g, 17.1 mmol) in anhydrous THF (15 mL) dropwise. The reaction mixture was stirred for 1 hour at room temperature before addition of saturated aqueous NH_4Cl (50 mL). The resulting phases were separated and the aqueous phase was extracted with Et_2O (2 × 50 mL). The organic phase was dried over Na_2SO_4 and concentrated *in vacuo* to afford the title compound (2.67 g, 96 %) as a pale yellow oil.

ν_{max} / cm^{-1}: (film) 3349 (br s), 3027 (m), 2975 (m), 1647 (m), 1453 (m), 1070 (s).

δ_H (400 MHz, $CDCl_3$) 7.33 – 7.27 (2H, m, ArCH), 7.24 – 7.19 (3H, m, ArCH), 5.16 – 5.14 (1H, m, C**4**-H), 4.75 (1H, d, J = 1.5 Hz, C**4**-H'), 4.26 (1H, q, J = 6.5 Hz, C**2**-H), 3.48 (1H, d, J = 15.5 Hz, C**5**-H), 3.36 (1H, d, J = 15.5 Hz, C**5**-H'), 1.52 (1H, br s, OH), 1.31 (3H, d, J = 6.5 Hz, C**1**-H_3).

δ_C (101 MHz, $CDCl_3$) 152.7 (C**3**), 139.5 (ArC), 129.3 (ArCH), 128.5 (ArCH), 126.3 (ArCH), 111.0 (C**4**), 70.3 (C**2**), 39.1 (C**5**), 22.4 (C**1**).

HRMS: (ESI^+) Calculated for $C_{11}H_{14}NaO$: 185.0939. Found $[M+Na]^+$: 185.0941.

Ethyl (*Z*)-4-benzylhex-4-enoate

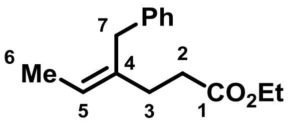

General procedure B: The preceding allylic alcohol (2.43 g, 15.0 mmol) was employed. The reaction time was 15 hours. FCC (eluent: 29:1 hexane:EtOAc) afforded the title compound (2.98 g, 86 %) as a colorless oil.

ν_{max} / cm^{-1}: (film) 3027 (m), 2980 (m), 1732 (s), 1602 (m), 1494 (m), 1452 (s), 1164 (s).

δ_H (400 MHz, $CDCl_3$) 7.30 – 7.24 (2H, m, ArCH), 7.21 – 7.13 (3H, m, ArCH), 5.45 (1H, q, *J* = 6.5 Hz, C**5**-H), 4.09 (2H, q, *J* = 7.0 Hz, OCH_2CH_3), 3.42 (2H, s, C**7**-H_2), 2.39 – 2.31 (2H, m, C**2**-H_2), 2.29 – 2.23 (2H, m, C**3**-H_2), 1.72 (3H, d, *J* = 6.5 Hz, C**6**-H_3), 1.22 (3H, t, *J* = 7.0 Hz, OCH_2CH_3).

δ_C (101 MHz, $CDCl_3$) 173.5 (C**1**), 140.0 (ArC), 137.0 (C**4**), 128.6 (ArCH), 128.5 (ArCH), 126.1 (ArCH), 121.1 (C**5**), 60.3 (OCH_2CH_3), 35.8 (C**7**), 33.2 (C**2**), 31.9 (C**3**), 14.4 (OCH_2CH_3), 13.8 (C**6**).

HRMS: (ESI^+) Calculated for $C_{15}H_{20}NaO_2$: 255.1356. Found $[M+Na]^+$: 255.1354.

(*Z*)-4-Benzylhex-en-1-ol

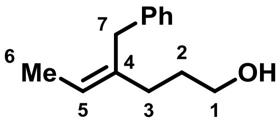

General procedure C: The preceding ester (1.50 g, 6.46 mmol) was employed using anhydrous THF as the solvent and 0.8 eq. $LiAlH_4$ (1.0 M in THF). FCC (eluent: 4:1 hexane:EtOAc) afforded the title compound (1.12 g, 91 %) as a colorless oil.

ν_{max} / cm^{-1}: (film) 3330 (br s), 3026 (m), 2929 (m), 1601 (m), 1452 (m), 1055 (s).

δ_H (400 MHz, $CDCl_3$) 7.33 – 7.24 (2H, m, ArCH), 7.22 – 7.12 (3H, m, ArCH), 5.46 (1H, q, *J* = 7.0 Hz, C**5**-H), 3.58 (2H, t, *J* = 6.5 Hz, C**1**-H_2), 3.42 (2H, s, C**7**-H_2), 2.02 – 1.97 (2H, m, C**3**-H_2), 1.73 (3H, d, *J* = 7.0 Hz, C**6**-H_3), 1.68 – 1.60 (2H, m, C**2**-H_2), 1.35 (1H, br s, OH).

δ_C (101 MHz, $CDCl_3$) 140.3 (ArC), 138.2 (C**4**), 128.6 (ArCH), 128.5 (ArCH), 126.0 (ArCH), 120.7 (C**5**), 62.9 (C**1**), 35.7 (C**7**), 32.9 (C**3**), 31.0 (C**2**), 13.8 (C**6**).

HRMS: (ESI^+) Calculated for $C_{13}H_{18}NaO$: 213.1250. Found $[M+Na]^+$: 213.1248.

***tert*-Butyl (*Z*)-(4-benzylhex-4-en-1-yl)((pentafluorobenzoyl)oxy)carbamate (2i)**

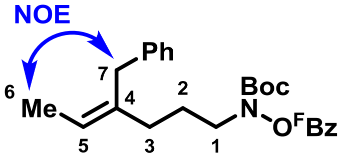

General procedure D: The preceding alcohol (860 mg, 4.52 mmol) was employed with **1a**. The reaction time was 16 hours. FCC (eluent: 3:7 hexane:PhMe) afforded **2i** (1.35 g, 60 %) as a colorless oil.

*The product was assigned as the Z isomer based on the observed NOE correlation between the C**6** and the C**7** protons.*

ν_{max} / cm^{-1}: (*film*) 3030 (m), 2935 (m), 1783 (s), 1721 (s), 1653 (m), 1505 (s), 1326 (s), 1150 (s).

δ_H (400 MHz, $CDCl_3$) 7.26 – 7.21 (2H, m, ArCH), 7.17 – 7.12 (3H, m, ArCH), 5.44 (1H, q, *J* = 7.0 Hz, C**5**-H), 3.59 (2H, t, *J* = 7.0 Hz, C**1**-H_2), 3.39 (2H, s, C**7**-H_2), 1.98 (2H, t, *J* = 7.5 Hz, C**3**-H_2), 1.74 – 1.65 (5H, m, C**2**-H_2 and C**6**-H_3), 1.46 (9H, s, $OC(CH_3)_3$).

δ_C (101 MHz, $CDCl_3$) 154.5 (Boc C=O), 140.0 (ArC), 137.2 (C**4**), 128.4 (ArCH), 128.3 (ArCH), 125.8 (ArCH), 121.1 (C**5**), 83.2 ($OC(CH_3)_3$), 50.6 (C**1**), 35.5 (C**7**), 33.4 (C**3**), 28.0 ($OC(CH_3)_3$), 25.1 (C**2**), 13.7 (C**6**).

The signals corresponding to the pentafluorobenzoyl group could not be resolved due to their weak intensity.

δ_F (377 MHz, $CDCl_3$) -136.3 – -136.5 (2F, m), -146.6 (1F, tt, *J* = 21.0, 5.5 Hz), -159.3 – -159.5 (2F, m).

HRMS: (ESI^+) Calculated for $C_{25}H_{26}F_5NNaO_4$: 522.1674. Found $[M+Na]^+$: 522.1666.

***tert*-Butyl 2-benzyl-2-vinylpyrrolidine-1-carboxylate (4i)**

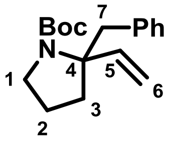

General procedure E: Conditions: 2.5 mol% $Pd_2(dba)_3$; 15 mol% PA-(4-(CF_3)C_6H_4) (**L-2**); 100 mol% Et_3N; THF (0.4 M); 130 °C. Substrate **2i** (52.4 mg, 0.105 mmol) was employed. FCC (eluent: 39:1 hexane:EtOAc) afforded **4i** (26.3 mg, 87 %) as a pale yellow oil.

Enantioselective reaction:

General procedure E: Conditions: 5.0 mol% $Pd_2(dba)_3$; 20 mol% (*S,R,R*)-(+)-(3,5-dioxa-4-phosphacyclohepta[2,1-*a*:3,4-*a'*]dinaphthalen-4-yl)bis(1-phenylethyl)amine (CAS: 497883-22-4); 100 mol% Et_3N; THF (0.4 M); 130 °C. Substrate **21** (52.4 mg, 0.105 mmol) was employed. FCC (*two times*, first eluent: 39:1 petroleum ether:EtOAc; second eluent: 74:1 PhMe:EtOAc) afforded **4i** (13.2 mg, 44 %, 48 % e.e.) as a colorless oil.

SFC conditions: *column*: CHIRALPACK IC, *elute*: 1.2 % MeOH/CO_2, *detector*: 250 nm, *flow rate*: 2 mL/min, *temperature*: 40 °C, *retention times*: (enantiomer A) t_1 = 10.6 min, (enantiomer B) t_2 = 11.2 min.

*When the reaction was performed using PA-Ph (**L-1**) under analogous conditions the yield was 75 %.*

ν_{max} / cm^{-1}: (*film*) 2974 (m), 1686 (s), 1381 (s), 1168 (s).

This compound exists as an approximately 1:1 mixture of rotamers A and B.

δ_H (400 MHz, $CDCl_3$) 7.31 – 7.13 (5H, m, A and B: ArCH), 6.10 (0.5H, dd, *J* = 17.5, 10.5 Hz, A: C**5**-H), 5.99 (0.5H, dd, *J* = 17.5, 10.5 Hz, B: C**5**-H), 5.12 – 4.96 (2H, m, A and B: C**6**-H_2), 3.67 (0.5H, d, *J* = 13.5 Hz, B: C**7**-H), 3.55 – 3.46 (1H, m, A: C**1**-H and C**7**-H), 3.40 (0.5H, ddd, *J* = 11.5, 8.0, 4.0 Hz, B: C**1**-H), 3.06 (0.5H, ddd, *J* = 11.0, 7.5, 7.5 Hz, A: C**1**-H’), 3.01 – 2.82 (1.5H, m, A: C**7**-H’; B: C**1**-H’ and C**7**-H’), 2.04 – 1.92 (1H, m, A and B: C**3**-H), 1.80 – 1.70 (1H, m, A and B: C**3**-H’), 1.60 – 1.48 (10H, m, A and B: C**2**-H and OC(CH_3)$_3$), 1.26 – 1.08 (1H, m, A and B: C**2**-H’).

δ_C (101 MHz, $CDCl_3$) 154.5 (B: C=O), 153.8 (A: C=O), 143.5 (B: C**5**), 142.5 (A: C**5**), 138.2 (A: ArC), 138.1 (B: ArC), 131.0 (B: ArCH), 130.7 (A: ArCH), 128.3 (A: ArCH), 128.0 (B: ArCH), 126.5 (B: ArCH), 126.3 (A: ArCH), 112.0 (B: C**6**), 111.7 (A: C**6**), 79.9 (B: OC(CH_3)$_3$), 79.0 (A: OC(CH_3)$_3$), 67.3 (B: C**4**), 66.8 (A: C**4**), 2 × 48.8 (A and B: C**1**), 42.3 (A: C**7**), 41.5 (B: C**7**), 37.9 (A: C**3**), 36.3 (B: C**3**), 28.8 (A and B: OC(CH_3)$_3$), 21.4 (B: C**2**), 21.0 (A: C**2**).

HRMS: (ESI^+) Calculated for $C_{18}H_{25}NNaO_2$: 310.1778. Found $[M+Na]^+$: 310.1788.

Methyl (*E*)-6-hydroxyhex-2-enoate

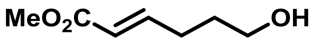

To a solution of Hoveyda-Grubbs 2nd generation catalyst (15.7 mg, 25.0 μmol) in anhydrous, degassed CH_2Cl_2 (40 mL) was added methyl acrylate (2.25 mL, 25.0 mmol) and pent-4-en-1-ol (0.26 mL, 2.50 mmol). The reaction mixture was heated at reflux for 15 hours before being concentrated *in vacuo*. The crude mixture was purified by FCC (eluent: 2:1 hexane:EtOAc) to afford the title compound (357 mg, 99 %) as a light brown oil (the coloration was due to the presence of trace amounts of Ru-impurities).

ν_{max} / cm^{-1}: (film) 3417 (br s), 2950 (m), 1720 (s), 1656 (s), 1436 (s), 1272 (s).

δ_H (400 MHz, $CDCl_3$) 6.98 (1H, dt, *J* = 15.5, 7.0 Hz), 5.85 (1H, dt, *J* = 15.5, 1.5 Hz), 3.72 (3H, s), 3.67 (2H, t, *J* = 6.5 Hz), 2.30 (2H, dtd, *J* = 7.0, 7.0, 1.5 Hz), 1.77 – 1.68 (2H, m), 1.51 (1H, br s).

δ_C (101 MHz, $CDCl_3$) 167.2, 148.9, 121.4, 62.1, 51.6, 31.0, 28.7.

The spectroscopic properties were consistent with the data available in the literature.[17]

Methyl (*E*)-6-((*tert*-butoxycarbonyl)((pentafluorobenzoyl)oxy)amino)hex-2-enoate (2j)

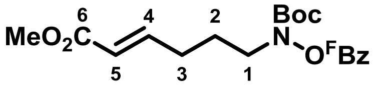

General procedure D: The preceding alcohol (214 mg, 1.48 mmol) was employed with **1a**. The reaction time was 16 hours. FCC (eluent: 49:1 PhMe:EtOAc) afforded **2j** (381 mg, 57 %) as a pale yellow oil.

ν_{max} / cm^{-1}: (*film*) 2952 (m), 1782 (s), 1721 (s), 1654 (m), 1504 (s), 1151 (s).

δ_H (400 MHz, $CDCl_3$) 6.95 (1H, dt, *J* = 15.5, 7.0 Hz, C**4**-H), 5.85 (1H, dt, *J* = 15.5, 1.5 Hz, C**5**-H), 3.73 – 3.68 (5H, m, C**1**-H_2 and OCH_3), 2.32 (2H, tdd, *J* = 7.5, 7.0, 1.5 Hz, C**3**-H_2), 1.80 (2H, tt, *J* = 7.5, 7.0 Hz, C**2**-H_2), 1.49 (9H, s, $OC(CH_3)_3$).

δ_C (101 MHz, $CDCl_3$) 167.0 (C**6**), 154.6 (Boc C=O), 147.8 (C**4**), 121.9 (C**5**), 83.7 ($OC(CH_3)_3$), 51.6 (OCH_3), 50.2 (C**1**), 29.1 (C**3**), 28.1 ($OC(CH_3)_3$), 25.6 (C**2**).

The signals corresponding to the pentafluorobenzoyl group could not be resolved due to their weak intensity.

δ_F (377 MHz, $CDCl_3$) -136.2 – -136.6 (2F, m), -146.2 (1F, tt, *J* = 20.0, 4.5 Hz), -159.0 – -159.4 (2F, m).

HRMS: (ESI^+) Calculated for $C_{19}H_{20}F_5NNaO_6$: 476.1103. Found $[M+Na]^+$: 476.1114.

***tert*-Butyl (*E*)-2-(2-methoxy-2-oxoethylidene)pyrrolidine-1-carboxylate (4j)**

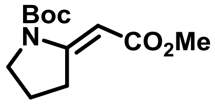

General procedure E: Conditions: 2.5 mol% $Pd_2(dba)_3$; 15 mol% PA-Ph (**L-1**); 100 mol% Et_3N; THF (0.4 M); 130 °C. Substrate **2j** (47.6 mg, 0.105 mmol) was employed. FCC (eluent: 9:1 hexane:EtOAc) afforded **4j** (24.0 mg, 95 %) as a colorless crystalline solid.

δ_H (400 MHz, $CDCl_3$) 6.44 (1H, t, *J* = 2.0 Hz), 3.68 – 3.63 (5H, m), 3.17 (2H, td, *J* = 7.5, 2.0 Hz), 1.87 (2H, tt, *J* = 7.5, 7.5 Hz), 1.51 (9H, s).

δ_C (101 MHz, $CDCl_3$) 169.5, 157.8, 152.1, 95.7, 82.2, 50.8, 49.9, 32.1, 28.3, 21.1.

m/z (ESI^+) 264 ($[M+Na]^+$, 100 %), 164 ($[M-(t\text{-}BuO\text{-}C{=}O)+H+Na]^+$, 22 %).

The spectroscopic properties were consistent with the data available in the literature.[18]

1-(Cyclohex-1-en-1-yl)ethan-1-ol

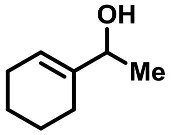

General procedure C: 1-Acetyl-1-cyclohexene (5.14 mL, 40.0 mmol) was employed using anhydrous Et_2O as solvent and 0.5 eq. $LiAlH_4$ (1.0 M in Et_2O). The title compound (3.98 g, 79 %) was isolated as a colorless oil.

δ_H (400 MHz, $CDCl_3$) 5.68 – 5.60 (1H, m), 4.20 – 4.10 (1H, m), 2.09 – 1.90 (4H, m), 1.69 – 1.47 (5H, m), 1.24 (3H, d, J = 6.5 Hz).

δ_C (101 MHz, $CDCl_3$) 141.4, 121.6, 72.3, 25.0, 23.8, 22.8, 22.7, 21.6.

The spectroscopic properties were consistent with the data available in the literature.[19]

Ethyl (*E*)-2-(2-ethylidenecyclohexyl)acetate

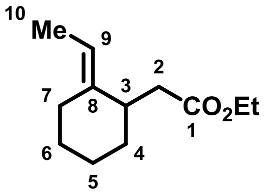

General procedure B: The preceding allylic alcohol (2.52 g, 20.0 mmol) was employed. The reaction time was 19 hours. FCC (eluent: 49:1 hexane:EtOAc) afforded the title compound (1.99 g, 51 %) as a colorless oil.

δ_H (400 MHz, $CDCl_3$) 5.10 (1H, q, J = 6.5 Hz, C**9**-H), 4.16 – 4.06 (2H, m, OCH_2CH_3), 2.59 – 2.48 (2H, m, C**2**-H and C**3**-H), 2.36 – 2.24 (2H, m, C**2**-H’ and C**7**-H), 2.04 – 1.93 (1H, m, C**7**-H’), 1.75 – 1.39 (8H, m, C**4**-H, C**5**-H_2, C**6**-H_2 and C**10**-H_3), 1.32 – 1.19 (4H, m, C**4**-H’ and OCH_2CH_3).

δ_C (101 MHz, $CDCl_3$) 173.2 (C**1**), 141.2 (C**8**), 114.2 (C**9**), 60.1 (OCH_2CH_3), 41.0 (C**3**), 38.0 (C**2**), 33.8 (C**4**), 27.6 (C**6**), 26.8 (C**7**), 24.4 (C**5**), 14.3 (OCH_2CH_3), 12.6 (C**10**).

The spectroscopic properties were consistent with the data available in the literature,[20] *although the ^1H spectrum is incorrectly assigned. The assignment provided here is supported by 2D NMR data.*

(*E*)-2-(2-Ethylidenecyclohexyl)ethan-1-ol

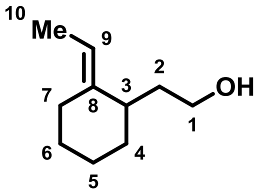

General procedure C: The preceding ester (1.98 g, 10.1 mmol) was employed using anhydrous THF as solvent and 0.8 eq. $LiAlH_4$ (1.0 M in THF). FCC (eluent: 4:1 hexane:EtOAc) afforded the title compound (627 mg, 40 %) as a colorless oil.

ν_{max} / cm^{-1}: (*film*) 3323 (br s), 2922 (s), 2854 (m), 1446 (m), 1053 (s).

δ_H (400 MHz, $CDCl_3$) 5.18 (1H, q, J = 6.5 Hz, C**9**-H), 3.62 (2H, dd, J = 6.5, 6.5 Hz, C**1**-H_2), 2.24 – 2.04 (3H, m, C**3**-H and C**7**-H_2), 1.90 (1H, ddt, J = 13.5, 9.0, 6.5 Hz, C**2**-H), 1.70 – 1.32 (10H, m, C**2**-H’, C**4**-H_2, C**5**-H_2, C**6**-H_2 and C**10**-H_3).

δ_C (101 MHz, $CDCl_3$) 142.4 (C**8**), 115.0 (C**9**), 61.9 (C**1**), 41.4 (C**3**), 34.8 (C**2**), 33.9 (C**4**), 27.8 (C**6**), 25.7 (C**7**), 23.3 (C**5**), 12.6 (C**10**).

HRMS: (ESI^+) Calculated for $C_{10}H_{18}NaO$: 177.1250. Found $[M+Na]^+$: 177.1248.

***tert*-Butyl (*E*)-(2-(2-ethylidenecyclohexyl)ethyl)((pentafluorobenzoyl)oxy)carbamate (2k)**

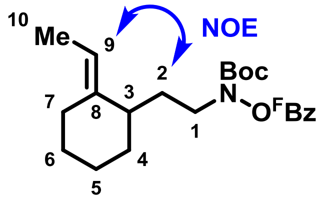

General procedure D: The preceding alcohol (309 mg, 2.00 mmol) was employed with **1a**. The reaction time was 14 hours. FCC (eluent: 1:1 hexane:PhMe) afforded **2k** (555 mg, 60 %) as a colorless oil.

*The product was assigned as the E isomer based on the observed NOE correlation between the C**2** and the C**9** protons.*

ν_{max} / cm^{-1}: (*film*) 2929 (m), 1785 (s), 1723 (s), 1653 (m), 1507 (s), 1156 (s).

δ_H (400 MHz, $CDCl_3$) 5.15 (1H, q, *J* = 6.5 Hz, C**9**-H), 3.62 (2H, dd, *J* = 7.5, 7.5 Hz, C**1**-H_2), 2.18 – 2.05 (3H, m, C**3**-H and C**7**-H_2), 1.93 (1H, ddt, *J* = 13.5, 8.0, 7.5 Hz, C**2**-H), 1.72 – 1.34 (19H, s, C**2**-H’, C**4**-H_2, C**5**-H_2, C**6**-H_2, C**10**-H_3 and $OC(CH_3)_3$).

δ_C (101 MHz, $CDCl_3$) 154.7 (Boc C=O), 141.2 (C**8**), 115.5 (C**9**), 83.3 ($OC(CH_3)_3$), 50.0 (C**1**), 41.9 (C**3**), 33.9 (C**4**), 29.0 (C**2**), 28.2 ($OC(CH_3)_3$), 27.9 (C**6**), 26.0 (C**7**), 23.7 (C**5**), 12.8 (C**10**).

The signals corresponding to the pentafluorobenzoyl group could not be resolved due to their weak intensity.

δ_F (377 MHz, $CDCl_3$) -136.4 – -136.6 (2F, m), -146.7 (1F, tt, *J* = 21.0, 5.5 Hz), -159.2 – -159.5 (2F, m).

HRMS: (ESI^+) Calculated for $C_{22}H_{26}F_5NNaO_4$: 486.1674. Found $[M+Na]^+$: 486.1681.

***tert*-Butyl (3a*R**,7a*S**)-7a-vinyloctahydro-1*H*-indole-1-carboxylate (4k)**

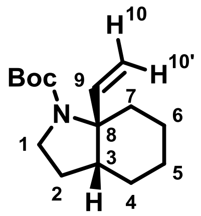

General procedure E: Conditions: 2.5 mol% $Pd_2(dba)_3$; 15 mol% PA-(4-(CF_3)C_6H_4) (**L-2**); 100 mol% Et_3N; THF (0.4 M); 130 °C. Substrate **2k** (48.7 mg, 0.105 mmol) was employed. FCC (eluent: 79:1 pentane:acetone) afforded **4k** (24.2 mg, 92 %) as a colorless oil.

*When the reaction was performed using PA-Ph (**L-1**) under analogous conditions the yield was 72 %.*
*It was not possible to determine the relative stereochemistry of **4k** by NOE analysis. The removal of the Boc group was necessary to obtain satisfactory data, details of this are given below.*

ν_{max} / cm^{-1}: (*film*) 2928 (m), 1682 (s), 1364 (s).

This compound exists as an approximately 3:2 mixture of rotamers A and B.

δ_H (500 MHz, $CDCl_3$) 5.83 – 5.69 (1H, m, A and B: C**9**-H), 5.10 – 5.03 (1H, m, A and B: C**10**-H), 4.99 (1H, d, *J* = 17.5 Hz, A and B: C**10**-H'), 3.63 – 3.47 (1H, m, A and B: C**1**-H), 3.46 – 3.34 (1H, m, A and B: C**1**-H'), 2.53 – 2.43 (0.4H, m, B: C**7**-H), 2.13 – 2.04 (0.6H, m, A: C**7**-H), 1.99 – 1.85 (1H, m, A and B: C**3**-H), 1.84 – 1.67 (2H, m, A and B: C**2**-H and C**7**-H'), 1.62 – 1.26 (16H, m, A and B: C**2**-H, C**4**-H_2, C**5**-H_2, C**6**-H_2 and $OC(CH_3)_3$).

δ_C (126 MHz, $CDCl_3$) 155.3 (A: C=O), 154.2 (B: C=O), 143.0 (A: C**9**), 142.6 (B: C**9**), 112.7 (A: C**10**), 112.5 (B: C**10**), 79.4 (A: $OC(CH_3)_3$), 78.7 (B: $OC(CH_3)_3$), 66.2 (B: C**8**), 65.4 (A: C**8**), 46.6 (B: C**1**), 46.5 (A: C**1**), 44.8 (A: C**3**), 43.4 (B: C**3**), 32.0 (A: C**7**), 31.3 (B: C**7**), 28.8 (B: $OC(CH_3)_3$), 28.7 (A: $OC(CH_3)_3$), 27.1 (B: C**4**), 26.7 (B: C**2**), 26.6 (A: C**4**), 26.2 (A: C**2**), 23.8 (B: C**5**), 23.2 (A: C**5**), 22.1 (A: C**6**), 22.0 (B: C**6**).

HRMS: (ESI^+) Calculated for $C_{15}H_{25}NNaO_2$: 275.1778. Found $[M+Na]^+$: 274.1776.

(3a*R,7a*S**)-7a-Vinyloctahydro-1*H*-indol-1-ium trifluoroacetate**

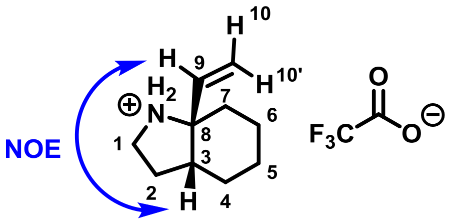

__General procedure F:__ Pyrrolidine **4k** (13.0 mg, 51.7 μmol) was employed to afford the title compound (13.5 mg, 98 %) as a colorless oil.

*The relative stereochemistry of the product was assigned based on the observed NOE correlation between the C**3** and the C**9** protons.*

ν_{max} / cm^{-1}: (*film*) 3418 (br s), 2941 (m), 1670 (s), 1425 (m), 1201 (s).

δ_H (400 MHz, $CDCl_3$) 9.45 (1H, br s, NH), 9.19 (1H, br s, NH'), 5.94 (1H, dd, *J* = 17.5, 11.0 Hz, C**9**-H), 5.50 (1H, d, *J* = 17.5 Hz, C**10**-H'), 5.44 (1H, d, *J* = 11.0 Hz, C**10**-H), 3.45 – 3.31 (2H, m, C**1**-H_2), 2.44 (1H, tt, *J* = 8.5, 4.0 Hz, C**3**-H), 2.13 – 2.02 (2H, m, C**2**-H_2), 1.93 (1H, ddd, *J* = 13.0, 3.5, 3.5 Hz, C**7**-H), 1.74 – 1.55 (4H, m, C**4**-H_2, C**6**-H and C**7**-H'), 1.52 – 1.42 (2H, m, C**5**-H_2), 1.41 – 1.27 (1H, m, C**6**-H').

δ_C (101 MHz, $CDCl_3$) 136.1 (C**9**), 120.0 (C**10**), 67.5 (C**8**), 41.9 (C**1**), 41.0 (C**3**), 28.9 (C**7**), 26.5 (C**2**), 24.2 (C**4**), 21.7 (C**6**), 20.0 (C**5**).

The signals corresponding to the trifluoroacetate counterion could not be resolved due to their weak intensity.

δ_F (377 MHz, $CDCl_3$) -75.6 (3F, s).

HRMS: (ESI^+) Calculated for $C_8H_{12}N$: 152.1434. Found $[M–F_3CCO_2]^+$: 152.1435.

2-(Cyclohex-2-en-1-yl)acetic acid

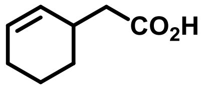

<u>General procedure G:</u> 3-Bromocyclohexene (6.90 mL, 60.0 mmol) was employed to afford the title compound as an orange oil (6.23 g, 74 %) which was used without further purification.

ν_{max} / cm^{-1}: (film) 3020 (m), 2927 (m), 1703 (s), 1289 (s).

δ_H (400 MHz, $CDCl_3$) 11.28 (1H, br s), 5.72 (1H, dtd, *J* = 10.0, 3.5, 2.0 Hz), 5.56 (1H, ddt, *J* = 10.0, 2.0, 2.0 Hz), 2.59 (1H, m), 2.36 (1H, dd, *J* = 15.5, 7.0 Hz), 2.29 (1H, dd, *J* = 15.5, 8.0 Hz), 1.98 (2H, dtt, *J* = 10.5, 5.5, 2.5 Hz), 1.90 – 1.81 (1H, m), 1.75 – 1.66 (1H, m), 1.61 – 1.50 (1H, m), 1.30 (1H, dddd, *J* = 13.5, 11.0, 8.5, 3.0 Hz).

δ_C (101 MHz, $CDCl_3$) 179.6, 129.9, 128.5, 40.7, 32.1, 28.8, 25.1, 21.0.

The spectroscopic properties were consistent with the data available in the literature.[21]

2-(Cyclohex-2-en-1-yl)ethan-1-ol

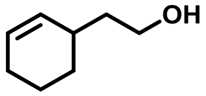

<u>General procedure C:</u> The preceding carboxylic acid (6.20 g, 44.0 mmol) was employed using anhydrous THF as solvent and 1.7 eq. $LiAlH_4$ (1.0 M in THF). The crude product was filtered through a short plug of silica, eluting with EtOAc, to afford the title compound (4.85 g, 87 %) as a yellow oil.

δ_H (400 MHz, $CDCl_3$) 5.67 (1H, dtd, *J* = 9.5, 3.5, 2.5 Hz), 5.56 (1H, ddt, *J* = 9.5, 2.0, 2.0 Hz), 3.71 (2H, tt, *J* = 7.0, 3.5 Hz), 2.22 (1H, dtdt, *J* = 11.5, 5.5, 2.5, 2.5 Hz), 1.96 (2H, tdd, *J* = 8.0, 4.0, 2.5 Hz), 1.79 (1H, dtd, *J* = 12.0, 6.0, 5.5, 2.5 Hz), 1.71 (1H, dqd, *J* = 12.0, 5.0, 2.5 Hz), 1.61 (1H, dt, *J* = 13.5, 6.5 Hz), 1.56 – 1.50 (1H, m), 1.49 (1H, s), 1.24 (1H, dddd, *J* = 12.5, 11.0, 8.5, 2.5 Hz).

δ_C (101 MHz, $CDCl_3$) 131.6, 127.4, 60.9, 39.2, 31.9, 29.1, 25.3, 21.4.

HRMS: (ESI^+) Calculated for $C_8H_{14}NaO$: 149.0937. Found $[M+Na]^+$: 149.0932.

The spectroscopic properties were consistent with the data available in the literature.[22]

tert-Butyl (2-(cyclohex-2-en-1-yl)ethyl)((pentafluorobenzoyl)oxy)carbamate (2l)

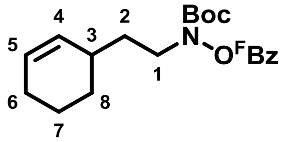

General procedure D: The preceding alcohol (240 mg, 1.90 mmol) was employed with **1a**. The reaction time was 16 hours. FCC (eluent: 2:3 hexane:PhMe) afforded **2l** (334 mg, 40 %) as a pale yellow oil.

ν_{max} / cm^{-1}: (*film*) 2932 (m), 1783 (s), 1721 (s), 1652 (m), 1504 (s), 1154 (s).

δ_H (400 MHz, $CDCl_3$) 5.69 (1H, dtd, *J* = 10.0, 3.5, 2.5 Hz, C**5**-H), 5.54 (1H, ddt, *J* = 10.0, 2.5, 2.5 Hz, C**4**-H), 3.73 (2H, t, *J* = 7.5 Hz, C**1**-H_2), 2.22 – 2.13 (1H, m, C**3**-H), 1.99 – 1.93 (2H, m, C**6**-H_2), 1.81 (1H, dddd, *J* = 12.5, 6.0, 6.0, 3.0 Hz, C**8**-H), 1.75 – 1.50 (4H, m, C**2**-H_2 and C**7**-H_2), 1.48 (9H, s, OC(CH_3)$_3$), 1.24 (1H, dddd, *J* = 12.5, 11.0, 8.5, 3.0 Hz, C**8**-H').

δ_C (101 MHz, $CDCl_3$) 154.7 (Boc C=O), 130.8 (C**4**), 127.9 (C**5**), 83.4 (OC(CH_3)$_3$), 49.0 (C**1**), 33.2 (C**2**), 32.6 (C**3**), 28.8 (C**8**), 28.2 (OC(CH_3)$_3$), 25.4 (C**6**), 21.4 (C**7**).

The signals corresponding to the pentafluorobenzoyl group could not be resolved due to their weak intensity.

δ_F (377 MHz, $CDCl_3$) -136.4 – -136.6 (2F, m), -146.6 (1F, tt, *J* = 21.0, 4.5 Hz), -159.2 – -159.4 (2F, m).

HRMS: (ESI$^+$) Calculated for $C_{20}H_{22}F_5NNaO_4$: 458.1361. Found [M+Na]$^+$: 458.1350.

tert-Butyl (3a*R,7a*S**)-2,3,3a,4,5,7a-hexahydro-1*H*-indole-1-carboxylate (4l)**

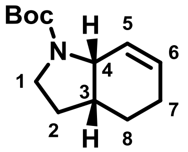

General procedure E: Conditions: 2.5 mol% $Pd_2(dba)_3$; 15 mol% PA-(4-(CF_3)C_6H_4) (**L-2**); 100 mol% Et_3N; THF (0.4 M); 130 °C. Substrate **2l** (45.7 mg, 0.105 mmol) was employed. FCC (eluent: 59:1 pentane:acetone) afforded **4l** (18.2 mg, 78 %) as a colorless oil.

*When the reaction was performed using PA-Ph (**L-1**) under analogous conditions the yield was 71 %. The Boc group of **4l** was removed to confirm that the presence of two sets of signals are due to the presence of two rotamers rather than two alkene isomers, details of this are given below.*

ν_{max} / cm^{-1}: (*film*) 2925 (m), 1690 (s), 1388 (s), 1114 (s).

This compound exists as an approximately 3:2 mixture of rotamers A and B.

δ_H (500 MHz, $CDCl_3$) 5.92 (0.4H, br d, J = 10.5 Hz, B: C**5**-H), 5.77 (0.6H, br d, J = 10.5 Hz, A: C**5**-H), 5.75 – 5.69 (1H, m, A and B: C**6**-H), 4.23 (0.4H, br s, B: C**4**-H), 4.13 (0.6H, br s, A: C**4**-H), 3.49 – 3.27 (2H, m, A and B: C**1**-H_2), 2.39 (1H, br s, A and B: C**3**-H), 2.10 – 1.92 (2H, m, A and B: C**7**-H_2), 1.85 – 1.72 (3H, m, A and B: C**2**-H_2 and C**8**-H), 1.71 – 1.63 (1H, m, A and B: C**8**-H'), 1.49 (5.4H, s, A: $OC(CH_3)_3$), 1.48 (3.6H, s, B: $OC(CH_3)_3$).

δ_C (126 MHz, $CDCl_3$) 154.8 (A and B: C=O), 127.6 (A: C**6**), 127.3 (B: C**6**), 127.0 (A: C**5**), 126.7 (B: C**5**), 79.2 (A: $OC(CH_3)_3$), 79.0 (B: $OC(CH_3)_3$), 55.1 (A: C**4**), 54.9 (B: C**4**), 45.4 (B: C**1**), 45.0 (A: C**1**), 36.6 (A: C**3**), 35.8 (B: C**3**), 28.7 (A and B: $OC(CH_3)_3$), 27.3 (B: C**2**), 26.5 (A: C**2**), 22.8 (A and B: C**8**), 2 × 20.9 (A and B: C**7**).

HRMS: (ESI$^+$) Calculated for $C_{13}H_{21}NNaO_2$: 246.1465. Found $[M+Na]^+$: 246.1470.

(3a*R*,7a*S***)-2,3,3a,4,5,7a-Hexahydro-1*H*-indol-1-ium trifluoroacetate**

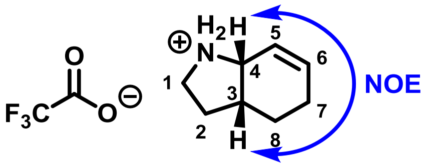

General procedure F: Pyrrolidine **4l** (12.3 mg, 55.1 μmol) was employed to afford the title compound (12.4 mg, 95 %) as a pale yellow oil.

*The product was assigned as the cis diastereomer based on the observed NOE correlation between the C**3** and the C**4** protons.*

ν_{max} / cm^{-1}: (*film*) 3425 (br s), 2936 (m), 1673 (s), 1429 (m), 1201(s).

δ_H (400 MHz, $CDCl_3$) 10.07 (1H, br s, NH), 8.98 (1H, br s, NH'), 6.16 – 6.10 (1H, m, C**6**-H), 5.75 (1H, dddd, J = 10.5, 4.0, 2.0, 2.0 Hz, C**5**-H), 3.96 (1H, br s, C**4**-H), 3.38 (1H, br s, C**1**-H), 3.28 (1H, br s, C**1**-H'), 2.55 – 2.41 (1H, m, C**3**-H), 2.24 – 2.11 (2H, m, C**2**-H and C**7**-H), 2.08 – 1.93 (1H, m, C**7**-H'), 1.86 (1H, dddd, J = 13.5, 8.5, 5.5, 5.5 Hz, C**2**-H'), 1.76 (1H, dddd, J = 13.5, 5.0, 5.0, 5.0 Hz, C**8**-H), 1.58 (1H, dddd, J = 13.5, 10.0, 8.0, 5.5 Hz, C**8**-H').

δ_C (101 MHz, $CDCl_3$) 135.3 (C**6**), 120.3 (C**5**), 56.3 (C**4**), 43.0 (C**1**), 35.1 (C**3**), 28.5 (C**2**), 22.8 (C**8**), 22.6 (C**7**).

The signals corresponding to the trifluoroacetate group could not be resolved due to their weak intensity.

δ_F (377 MHz, $CDCl_3$) -75.7 (3F, s).

HRMS: (ESI$^+$) Calculated for $C_8H_{14}N$: 124.1121. Found $[M–F_3CCO_2]^+$: 124.1120.

Cyclohex-1-en-ylmethanol

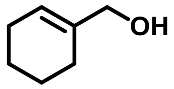

To a solution of methyl 1-cyclohexene-1-carboxylate (5.00 g, 35.6 mmol) in anhydrous CH_2Cl_2 (*approx.* 100 mL) at -78 °C was added diisobutyl aluminium hydride (1.0 M in CH_2Cl_2, 78 mL, 78 mmol). The reaction mixture was stirred at this temperature for 2 hours before addition of MeOH (70 mL) and saturated aqueous Rochelle's salt (70 mL). The mixture was warmed to room temperature and stirred overnight before the resulting phases were separated and the aqueous phase was extracted with EtOAc (2 × 100 mL). The EtOAc extracts were washed with brine (100 mL) and saturated aqueous Rochelle's salt (100 mL) before being dried over Na_2SO_4 and concentrated *in vacuo*. The crude mixture was purified by FCC (gradient elution 8:1 – 4:1 hexane:EtOAc) to afford the title compound (3.98 g, 100 %) as a colorless oil.

ν_{max} / cm^{-1}: (film) 3307 (br, s), 2924 (s), 2857 (s), 2835 (s), 1437 (s), 1004 (s).

δ_H (400 MHz, $CDCl_3$) 5.68 – 5.65 (1H, m), 3.96 (2H, s), 2.05 – 1.97 (4H, m), 1.68 – 1.54 (4H, m), 1.52 (1H, s).

δ_C (101 MHz, $CDCl_3$) 137.7, 123.1, 67.8, 25.7, 25.0, 22.7, 22.6.

The spectroscopic properties were consistent with the data available in the literature.[23]

1-(Bromomethyl)cyclohex-1-ene

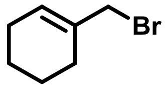

To a solution of the preceding allylic alcohol (2.14 g, 19.0 mmol) in Et_2O (80 mL) at 0 °C was added PBr_3 (0.89 mL, 9.49 mmol). The reaction mixture was warmed to room temperature and stirred for 18 hours before being poured into a solution of K_2CO_3 (2.63 g, 19.0 mmol) in water (200 mL). The phases were separated and the aqueous phase was extracted with Et_2O (2 × 100 mL). The Et_2O phases were washed with brine (150 mL), dried over Na_2SO_4 and concentrated *in vacuo* afford the title compound (2.76 g, 83 %) as a colorless oil.

δ_H (400 MHz, $CDCl_3$) 5.88 – 5.85 (m, 1H), 3.93 (s, 2H), 2.14 – 2.09 (m, 2H), 2.06 – 1.99 (m, 2H), 1.70 – 1.62 (m, 2H), 1.60 – 1.52 (m, 2H).

δ_C (101 MHz, $CDCl_3$) 134.6, 128.1, 39.8, 26.3, 25.4, 22.4, 21.8.

m/z (EI^+) 176 and 174 ($[M]^+$, 52 and 53 %), 95 ($[M-Br]^+$, 100 %), 84 (80 %).

The spectroscopic properties were consistent with the data available in the literature.[23]

3-(Cyclohex-1-en-yl)propanoic acid

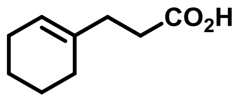

<u>General procedure G:</u> The preceding allylic bromide (1.36 mL, 10.0 mmol) was employed, the crude product was used in the next step without further purification.

δ_H (400 MHz, $CDCl_3$) 5.51 – 5.39 (1H, m), 2.45 (2H, dd, *J* = 9.0, 6.5 Hz), 2.25 (2H, t, *J* = 8.0 Hz), 2.00 – 1.88 (4H, m), 1.65 – 1.58 (2H, m), 1.57 – 1.50 (2H, m).

δ_C (101 MHz, $CDCl_3$) 178.5, 135.7, 121.7, 32.6, 32.3, 28.2, 25.1, 22.8, 22.3.

The spectroscopic properties were consistent with the data available in the literature.[24]

3-(Cyclohex-1-en-yl)propan-1-ol

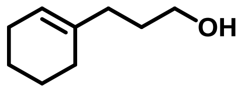

<u>General procedure C:</u> The preceding crude carboxylic acid was employed using anhydrous THF as the solvent and 1.5 eq. $LiAlH_4$ (1.0 M in Et_2O). The crude mixture was purified by FCC (eluent: 4:1 hexane:EtOAc) to afford the title compound (1.26 g, 90 % over two steps) as a pale yellow oil.

ν_{max} / cm^{-1}: (film) 3326 (br s), 2923 (s), 2834 (s), 1438 (m), 1058 (s).

δ_H (400 MHz, $CDCl_3$) 5.44 – 5.40 (1H, m), 3.61 (2H, t, *J* = 6.5 Hz), 2.02 – 1.88 (6H, m), 1.74 (1H, s), 1.69 – 1.48 (6H, m).

δ_C (101 MHz, $CDCl_3$) 137.5, 121.4, 63.0, 34.5, 30.6, 28.3, 25.3, 23.1, 22.6.

The spectroscopic properties were consistent with the data available in the literature.[24]

***tert*-Butyl (3-(cyclohex-1-en-1-yl)propyl)((pentafluorobenzoyl)oxy)carbamate (2m)**

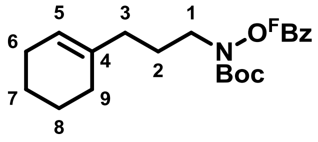

<u>General procedure D:</u> The preceding alcohol (280 mg, 2.00 mmol) was employed with **1a**. The reaction time was 16 hours. FCC (gradient elution: 3:7 – 0:1 hexane:PhMe) afforded **2m** (561 mg, 62 %) as a colorless oil.

ν_{max} / cm^{-1}: (*film*) 2932 (m), 1783 (s), 1721 (s), 1652 (m), 1505 (s), 1326 (s), 1150 (s).

δ_H (400 MHz, $CDCl_3$) 5.44 – 5.39 (1H, m, C**5**-<u>H</u>), 3.64 (2H, t, *J* = 7.5 Hz, C**1**-<u>H</u>$_2$), 2.03 – 1.94 (4H, m, C**3**-<u>H</u>$_2$ and C**6**-<u>H</u>$_2$), 1.93 – 1.87 (2H, m, C**9**-<u>H</u>$_2$), 1.74 (2H, tt, *J* = 7.5, 7.5 Hz, C**2**-<u>H</u>$_2$), 1.65 – 1.58 (2H, m, C**8**-<u>H</u>$_2$), 1.57 – 1.51 (2H, m, C**7**-<u>H</u>$_2$), 1.49 (9H, s, OC(C<u>H</u>$_3$)$_3$).

δ$_C$ (101 MHz, $CDCl_3$) 154.7 (Boc C=O), 136.5 (C**4**), 121.9 (C**5**), 83.3 (OC(CH_3)$_3$), 50.9 (C**1**), 34.9 (C**3**), 28.3 (C**9**), 28.2 (OC(CH_3)$_3$), 25.4 (C**6**), 24.9 (C**2**), 23.1 (C**8**), 22.6 (C**7**).

The signals corresponding to the pentafluorobenzoyl group could not be resolved due to their weak intensity.

δ$_F$ (377 MHz, $CDCl_3$) -136.4 – -136.8 (2F, m), -146.7 (1F, tt, J = 21.0, 5.0 Hz), -159.4 – -159.6 (2F, m).

HRMS: (ESI$^+$) Calculated for $C_{21}H_{24}F_5NNaO_4$: 472.1518. Found [M+Na]$^+$: 472.1514.

***tert*-Butyl 1-azaspiro[4.5]dec-6-ene-1-carboxylate (4m)**

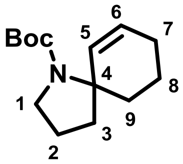

General procedure E: Conditions: 2.5 mol% $Pd_2(dba)_3$; 15 mol% PA-(4-(CF_3)C_6H_4) (**L-2**); 100 mol% Et_3N; THF (0.4 M); 130 °C. Substrate **2m** (47.2 mg, 0.105 mmol) was employed. FCC (eluent: 39:1 PhMe:EtOAc) afforded **4m** (20.3 mg, 81 %) as a colorless oil.

*When the reaction was performed using PA-Ph (**L-1**) under analogous conditions the yield was 70 %.* *The Boc group of **4m** was removed to confirm that the presence of two sets of signals are due to the presence of two rotamers rather than two alkene isomers, details of this are given below.*

ν_{max} / cm^{-1}: (*film*) 2969 (m), 2927 (m), 1683 (s), 1380 (s), 1159 (s).

This compound exists as an approximately 13:7 mixture of rotamers A and B.

δ$_H$ (500 MHz, $CDCl_3$) 5.79 – 5.70 (0.35H, m, B: C**5**-H), 5.68 – 5.60 (0.65H, m, A: C**5**-H), 5.51 (0.35H, br d, J = 10.0 Hz, B: C**6**-H), 5.41 (0.65H, br d, J = 10.0 Hz, A: C**6**-H), 3.61 – 3.45 (1H, m, A and B: C**1**-H), 3.43 – 3.31 (1H, m, A and B: C**1**-H'), 2.39 – 2.27 (0.35H, m, B: C**9**-H), 2.22 – 2.09 (1H, m, A: C**9**-H and B: C**7**-H), 2.00 – 1.88 (2.65H, m A: C**3**-H and C**7**-H_2, B: C**3**-H and C**7**-H'), 1.84 – 1.71 (4H, m, A and B: C**2**-H_2, C**3**-H' and C**8**-H), 1.65 – 1.50 (2H, m, A and B: C**8**-H' and C**9**-H'), 1.45 (9H, s, A and B: OC(CH_3)$_3$).

δ$_C$ (126 MHz, $CDCl_3$) 154.6 (A: C=O), 153.3 (B: C=O), 134.7 (A: C**6**), 134.1 (B: C**6**), 126.6 (B: C**5**), 126.0 (A: C**5**), 79.2 (A: OC(CH_3)$_3$), 78.8 (B: OC(CH_3)$_3$), 62.3 (A and B: C**4**), 47.9 (B: C**1**), 47.6 (A: C**1**), 40.1 (A: C**3**), 39.3 (B: C**3**), 32.1 (A: C**9**), 30.9 (B: C**9**), 28.8 (B: OC(CH_3)$_3$), 28.5 (A: OC(CH_3)$_3$), 24.5 (A: C**7**), 24.2 (B: C**7**), 22.5 (B: C**2**), 22.0 (A: C**2**), 21.9 (A: C**8**), 21.6 (B: C**8**).

HRMS: (ESI$^+$) Calculated for $C_{14}H_{23}NNaO_2$: 260.1621. Found [M+Na]$^+$: 260.1626.

1-Azaspiro[4.5]dec-6-en-1-ium trifluoroacetate

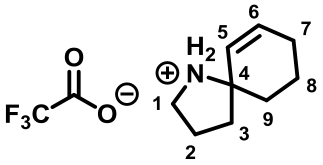

General procedure F: Pyrrolidine **4m** (17.3 mg, 72.9 μmol) was employed to afford the title compound (17.7 mg, 97 %) as a pale yellow oil.

ν_{max} / cm^{-1}: (*film*) 3422 (br s), 2946 (m), 1671 (s), 1431 (m), 1201 (s).

δ_H (400 MHz, $CDCl_3$) 9.25 (2H, br s, N$\underline{H}_2$), 6.00 (1H, dt, *J* = 10.0, 4.0 Hz, C**6**-$\underline{H}$), 5.70 (1H, d, *J* = 10.0 Hz, C**5**-$\underline{H}$), 3.44 – 3.24 (2H, m, C**1**-$\underline{H}_2$), 2.17 – 1.89 (7H, m, C**2**-$\underline{H}_2$, C**3**-$\underline{H}_2$, C**7**-$\underline{H}_2$ and C**9**-$\underline{H}$), 1.85 – 1.61 (3H, m, C**8**-$\underline{H}_2$ and C**9**-$\underline{H}$').

δ_C (101 MHz, $CDCl_3$) 134.0 (C**6**), 126.0 (C**5**), 65.6 (C**4**), 44.0 (C**1**), 37.6 (C**3**), 31.8 (C**9**), 24.6 (C**7**), 22.8 (C**2**), 19.5 (C**8**).

The signals corresponding to the trifluoroacetate group could not be resolved due to their weak intensity.

δ_F (377 MHz, $CDCl_3$) -75.6 (3F, s).

HRMS: (ESI^+) Calculated for $C_9H_{16}N$: 138.1277. Found $[M–F_3CCO_2]^+$: 138.1283.

Cyclohex-3-en-1-ylmethanol

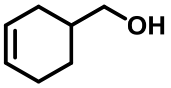

To a solution of 3-cyclohexene-1-carboxaldehyde (11.0 g, 100 mmol) in MeOH (150 mL) at 0 °C was added $NaBH_4$ (1.51 g, 40.0 mmol), the reaction mixture was stirred at this temperature for 1 hour before addition of water (100 mL) and extraction with Et_2O (3 × 150 mL). The organic phase was dried over Na_2SO_4 and concentrated *in vacuo*. FCC (gradient elution 3:1 – 1:1 pentane:Et_2O) afforded the title compound (8.00 g, 71 %) as a pale yellow oil.

δ_H (400 MHz, $CDCl_3$) 5.70 – 5.62 (2H, m), 3.56 – 3.46 (2H, m), 2.14 – 2.02 (3H, m), 1.86 – 1.67 (4H, m), 1.32 – 1.20 (1H, m).

δ_C (101 MHz, $CDCl_3$) 127.2, 126.0, 67.9, 36.4, 28.2, 25.3, 24.7.

The spectroscopic properties were consistent with the data available in the literature.[25]

***tert*-Butyl (cyclohex-3-en-1-ylmethyl)((pentafluorobenzoyl)oxy)carbamate (2n)**

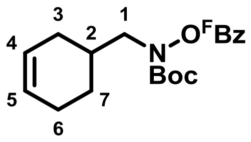

General procedure D: The preceding alcohol (224 mg, 2.00 mmol) was employed with **1a**. The reaction time was 21 hours. FCC (eluent: 2:3 hexane:PhMe) afforded **2n** (451 mg, 54 %) as a waxy solid.

ν_{max} / cm^{-1}: (*film*) 3026 (m), 2920 (m), 1784 (s), 1720 (s), 1652 (m), 1504 (s), 1326 (s), 1152 (s).

δ_H (400 MHz, $CDCl_3$) 5.72 – 5.61 (2H, m, C**4**-H and C**5**-H), 3.68 – 3.55 (2H, m, C**1**-H_2), 2.21 – 2.11 (1H, m, C**3**-H), 2.11 – 2.02 (2H, m, C**6**-H_2), 2.02 – 1.92 (1H, m, C**2**-H), 1.88 – 1.73 (2H, m, C**3**-H' and C**7**-H), 1.49 (9H, s, OC(CH_3)$_3$), 1.39 – 1.27 (1H, m, C**7**-H').

δ_C (101 MHz, $CDCl_3$) 154.7 (Boc C=O), 127.2, (C**5**) 125.6 (C**4**), 83.3 (OC(CH_3)$_3$), 56.3 (C**1**), 32.1 (C**2**), 29.2 (C**3**), 28.2 (OC(CH_3)$_3$), 26.1 (C**7**), 24.5 (C**6**).

The signals corresponding to the pentafluorobenzoyl group could not be resolved due to their weak intensity.

δ_F (377 MHz, $CDCl_3$) -136.2 – -136.6 (2F, m), -146.5 (1F, tt, J = 21.0, 5.0 Hz), -159.2 – -159.4 (2F, m).

HRMS: (ESI^+) Calculated for $C_{19}H_{20}F_5NNaO_4$: 444.1205. Found $[M+Na]^+$: 444.1207.

***tert*-Butyl (1*R**,5*R**)-6-azabicyclo[3.2.1]oct-3-ene-6-carboxylate (4n)**

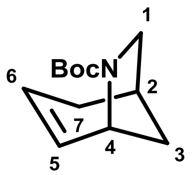

General procedure E: Conditions: 2.5 mol% $Pd_2(dba)_3$; 15 mol% PA-Ph (**L-1**); 100 mol% Et_3N; THF (0.4 M); 130 °C. Substrate **2n** (44.2 mg, 0.105 mmol) was employed. FCC (eluent: 49:1 PhMe:EtOAc) afforded **4n** (14.0 mg, 64 %) as a pale yellow oil.

ν_{max} / cm^{-1}: (*film*) 2928 (m), 1690 (s), 1397 (s), 1099 (s).

This compound exists as an approximately 3:2 mixture of rotamers A and B. The Boc group of ***4n*** *was removed to confirm that the presence of two sets of signals are due to the presence of two rotamers rather than two alkene isomers, details of this are given below.*

δ_H (400 MHz, $CDCl_3$) 6.18 (0.4H, dd, J = 8.0, 7.0 Hz, B: C**5**-H), 6.07 (0.6H, dd, J = 7.5, 7.5 Hz, A: C**5**-H), 5.60 – 5.51 (1H, m, A and B: C**6**-H), 4.26 – 4.20 (0.4H, m B: C**4**-H), 4.13 – 4.06 (0.6H, m, A: C**4**-H), 3.56 – 3.46 (1H, m, A and B: C**1**-H), 3.22 (0.6H, br d, J = 11.0 Hz, A: C**1**-H'), 3.14 (0.4H, br

d, *J* = 11.0 Hz, B: C**1**-H’), 2.57 – 2.42 (2H, m, A and B: C**2**-H and C**7**-H), 2.09 (1H, br d, *J* = 18.0 Hz, A and B: C**7**-H’), 1.90 – 1.77 (1H, m, A and B: C**3**-H), 1.73 (1H, br d, *J* = 10.5 Hz, A and B: C**3**-H’), 1.45 (9H, s, A and B: $OC(CH_3)_3$).

δ_C (126 MHz, $CDCl_3$) 154.2 (A: C=O), 153.8 (B: C=O), 131.7 (B: C**5**), 131.4 (A: C**5**), 127.4 (A: C**6**), 127.1 (B: C**6**), 79.0 (A and B: $OC(CH_3)_3$), 53.1 (B: C**1**), 52.9 (A: C**1**), 51.6 (A: C**4**), 50.8 (B: C**4**), 2 × 35.4 (A and B: C**7**), 34.5 (A: C**3**), 34.0 (B: C**3**), 33.2 (B: C**2**), 32.4 (A: C**2**), 28.7 (A and B: $OC(CH_3)_3$).

HRMS: (ESI^+) Calculated for $C_{12}H_{19}NNaO_2$: 232.1308. Found $[M+Na]^+$: 232.1315.

(1*R,5*R**)-6-Azabicyclo[3.2.1]oct-3-en-6-ium trifluoroacetate**

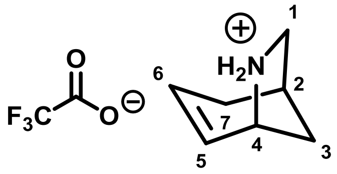

General procedure F: Pyrrolidine **4n** (4.8 mg, 22.9 µmol) was employed to afford the title compound (4.5 mg, 88 %) as a pale yellow oil.

ν_{max} / cm^{-1}: (*film*) 3415 (br s), 2981 (m), 1674 (s), 1430 (m), 1201 (s).

δ_H (500 MHz, $CDCl_3$) 9.64 (1H, br s, NH), 9.34 (1H, br s, NH’), 5.99 – 5.92 (1H, m, C**5**-H), 5.92 – 5.86 (1H, m, C**6**-H), 4.12 – 4.02 (1H, m, C**4**-H), 3.57 – 3.46 (1H, m, C**1**-H), 3.27 – 3.18 (1H, m, C**1**-H’), 2.78 – 2.69 (1H, m, C**2**-H), 2.56 (1H, br d, *J* = 19.0 Hz, C**7**-H), 2.19 (1H, br d, *J* = 19.0 Hz, C**7**-H’), 2.11 – 2.05 (1H, m, C**3**-H), 1.96 (1H, d, *J* = 12.0 Hz, C**3**-H’).

δ_C (126 MHz, $CDCl_3$) 132.2 (C**6**), 125.7 (C**5**), 52.6 (C**4**), 50.4 (C**1**), 35.3 (C**7**), 32.8 (C**3**), 32.5 (C**2**).

The signals corresponding to the trifluoroacetate group could not be resolved due to their weak intensity.

δ_F (377 MHz, $CDCl_3$) -75.64 (3F, s).

HRMS: (ESI^+) Calculated for $C_7H_{12}N$: 110.0964. Found $[M–F_3CCO_2]^+$: 110.0969.

Cyclizations to form 6-membered rings

tert-Butyl(hex-5-yn-1-yloxy)dimethylsilane

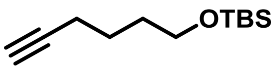

This compound was prepared according to a literature procedure.[26]

ν_{max} / cm^{-1}: (film) 3314 (m), 2930 (s), 2858 (s), 2120 (m), 1472 (m), 1254 (s), 1104 (s).

δ_H (400 MHz, $CDCl_3$) 3.62 (1H, t, *J* = 6.0 Hz), 2.20 (2H, td, *J* = 7.0, 2.5 Hz), 1.92 (1H, t, *J* = 2.5 Hz), 1.67 – 1.52 (4H, m), 0.88 (9H, s), 0.03 (6H, s).

δ_C (101 MHz, $CDCl_3$) 84.7, 68.4, 62.7, 32.0, 26.1, 25.1, 18.5, 18.4, -5.2.

HRMS: (ESI^+) Calculated for $C_{12}H_{24}NaOSi$: 235.1489. Found $[M+Na]^+$: 235.1479.

The spectroscopic properties were consistent with the data available in the literature.[27]

tert-Butyl((7-cyclopropylhept-5-yn-1-yl)oxy)dimethylsilane

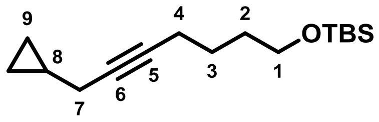

To a solution of the preceding alkyne (4.25 g, 20.0 mmol) in anhydrous THF (30 mL) and DMPU (30 mL) at -78 °C was added ^{n}BuLi (2.5 M in hexane, 8.4 mL, 21.0 mmol), the reaction mixture was stirred at this temperature for 1.5 hours before being warmed to 0 °C and addition of cyclopropylmethyl bromide (2.52 mL, 26.0 mmol). The reaction mixture was stirred at room temperature for 14 hours before addition of saturated aqueous NH_4Cl (50 mL), brine (50 mL) and Et_2O (100 mL). The resulting phases were separated and the aqueous phase was extracted with Et_2O (2 × 100 mL). The organic phase was dried over Na_2SO_4 and concentrated *in vacuo*. FCC (gradient elution: 19:1 – 9:1 – 4:1 petroleum ether:PhMe) resulted in impure material which was distilled (b.p. 110 °C, 2 mbar) to afford the title compound (1.56 g, 29 %) as a colorless oil.

ν_{max} / cm^{-1}: (*film*) 2929 (m), 2857 (m) 1472 (m), 1254 (s), 1104 (s).

δ_H (400 MHz, $CDCl_3$) 3.62 (2H, t, *J* = 6.0 Hz, C**1**-$\underline{H}_2$), 2.23 – 2.13 (4H, m, C**4**-$\underline{H}_2$ and C**7**-$\underline{H}_2$), 1.66 – 1.47 (4H, m, C**2**-$\underline{H}_2$ and C**3**-$\underline{H}_2$), 0.92 – 0.85 (1H, m, C**8**-$\underline{H}$), 0.89 (9H, s, $SiC(C\underline{H}_3)_3$), 0.48 – 0.40 (2H, m, 2 × C**9**-$\underline{H}$), 0.21 (2H, dt, *J* = 6.0, 4.5 Hz, 2 × C**9**-$\underline{H}$’), 0.05 (6H, s, $Si(C\underline{H}_3)_2$).

δ_C (101 MHz, $CDCl_3$) 80.5 (C**5**), 78.6 (C**6**), 62.9 (C**1**), 32.1 (C**2**), 26.1 ($SiC(\underline{C}H_3)_3$), 25.7 (C**3**), 23.1 (C**7**), 18.7 (C**4**), 18.5 ($Si\underline{C}(CH_3)_3$), 9.8 (C**8**), 3.9 (C**9**), -5.1 ($Si(\underline{C}H_3)_2$).

(*Z*)-*tert*-Butyl((7-cyclopropylhept-5-en-1-yl)oxy)dimethylsilane

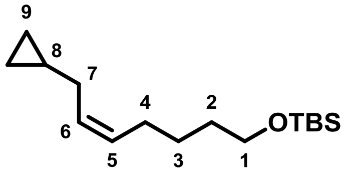

A solution of the preceding alkyne (1.33 g, 5.00 mmol), Lindlar catalyst (53.2 mg, 25.0 μmol) and quinoline (6.0 μL, 50.0 μmol) in hexane (25 mL) was stirred under an atmosphere of H_2 (balloon pressure) for 3 hours. The reaction mixture was filtered through celite and the filter cake was washed with CH_2Cl_2 (30 mL). The filtrate was concentrated *in vacuo*. FCC (eluent: 24:1 petroleum ether:PhMe) afforded the title compound (1.04 g, 77 %) as a colorless oil.

ν_{max} / cm^{-1}: (*film*) 3077 (m), 3006 (m), 2929 (m), 2857 (m), 1472 (m), 1254 (s), 1100 (s).

δ_H (400 MHz, $CDCl_3$) 5.51 – 5.33 (2H, m, C**5**-H and C**6**-H), 3.61 (2H, t, *J* = 6.5 Hz, C**1**-H_2), 2.03 (2H, dt, *J* = 7.5, 7.5 Hz, C**4**-H_2), 1.96 (2H, dd, *J* = 7.0, 7.0 Hz, C**7**-H_2), 1.59 – 1.47 (2H, m, C**2**-H_2), 1.38 (2H, tt, *J* = 7.5, 7.5 Hz, C**3**-H_2), 0.89 (9H, s, SiC(CH_3)$_3$), 0.76 – 0.65 (1H, m, C**8**-H), 0.44 – 0.38 (2H, m, 2 × C**9**-H), 0.09 – 0.02 (8H, m, 2 × C**9**-H’ and Si(CH_3)$_2$).

δ_C (101 MHz, $CDCl_3$) 130.0 (C**5**), 129.0 (C**6**), 63.3 (C**1**), 32.6 (C**2**), 32.0 (C**7**), 27.2 (C**4**), 26.2 (C**3**), 26.1 (SiC(CH_3)$_3$), 18.5 (SiC(CH_3)$_3$), 11.1 (C**8**), 4.2 (C**9**), -5.1 (Si(CH_3)$_2$).

HRMS: (ESI^+) Calculated for $C_{16}H_{33}OSi$: 269.2295. Found $[M+H]^+$: 269.2292.

(*Z*)-7-Cyclopropylhept-5-en-1-ol

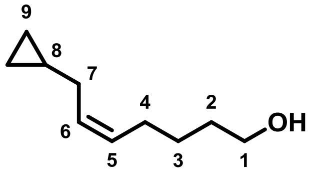

A solution of the preceding silyl ether (943 mg, 3.51 mmol) and TBAF (1.0 M in THF, 6.00 mL, 6.00 mmol) in THF (15 mL) was stirred at room temperature for 2.5 hours. The reaction mixture was concentrated *in vacuo*, dissolved in Et_2O (50 mL) and washed with water (25 mL) before being dried over Na_2SO_4 and concentrated *in vacuo*. The crude mixture was purified by FCC (eluent: 3:2 petroleum ether:Et_2O) to afford the title compound (510 mg, 94 %) as a colorless oil.

ν_{max} / cm^{-1}: (*film*) 3328 (br s), 3076 (m), 3005 (m), 2932 (s), 1458 (m), 1427 (m), 1043 (s).

δ_H (400 MHz, $CDCl_3$) 5.47 (1H, dtt, *J* = 11.0, 7.0, 1.5 Hz, C**6**-H), 5.42 – 5.33 (1H, m, C**5**-H), 3.64 (2H, t, *J* = 6.5 Hz, C**1**-H_2), 2.05 (2H, br dt, *J* = 7.5, 7.5 Hz, C**4**-H_2), 1.96 (2H, dd, *J* = 7.0, 7.0 Hz, C**7**-H_2), 1.63 – 1.52 (2H, m, C**2**-H_2), 1.46 – 1.36 (2H, m, C**3**-H_2), 0.77 – 0.64 (1H, m, C**8**-H), 0.45 – 0.37 (2H, m, 2 × C**9**-H), 0.09 – 0.03 (2H, m, 2 × C**9**-H’).

δ_C (101 MHz, $CDCl_3$) 129.7 (C**5**), 129.3 (C**6**), 63.1 (C**1**), 32.5 (C**2**), 32.0 (C**7**), 27.1 (C**4**), 26.0 (C**3**), 11.1 (C**8**), 4.2 (C**9**).

HRMS: (ESI^+) Calculated for $C_{10}H_{18}NaO$: 177.1250. Found $[M+Na]^+$: 177.1238.

***tert*-Butyl (*Z*)-(7-Cyclopropylhept-5-en-1-yl)((pentafluorobenzoyl)oxy)carbamate (2o)**

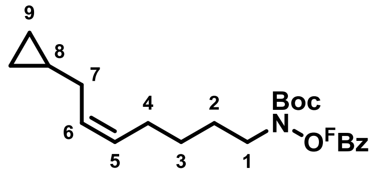

General procedure D: The preceding alcohol (309 mg, 2.00 mmol) was employed with 1a**.** The reaction time was 15 hours. FCC (eluent: 1:1 petroleum ether:PhMe) afforded **2o** (586 mg, 63 %) as a colorless oil.

ν_{max} / cm^{-1}: (*film*) 3078 (m), 3006 (m), 2936 (m), 1783 (s), 1721 (s), 1652 (m), 1505 (s), 1153 (s).

δ_H (400 MHz, $CDCl_3$) 5.52 – 5.43 (1H, m, C**6**-H), 5.40 – 5.32 (1H, m, C**5**-H), 3.67 (2H, t, *J* = 7.0 Hz, C**1**-H_2), 2.06 (2H, dt, *J* = 7.0, 7.0 Hz, C**4**-H_2), 1.95 (2H, dd, *J* = 7.0, 7.0 Hz, C**7**-H_2), 1.64 (2H, tt, *J* = 7.5, 7.0 Hz, C**2**-H_2), 1.49 (9H, s, $OC(CH_3)_3$), 1.43 (2H, tt, *J* = 7.5, 7.0 Hz, C**3**-H_2), 0.75 – 0.64 (1H, m, C**8**-H), 0.44 – 0.37 (2H, m, 2 × C**9**-H), 0.08 – 0.02 (2H, m, 2 × C**9**-H’).

δ_C (101 MHz, $CDCl_3$) 154.7 (Boc C=O), 129.5 (C**6**), 129.3 (C**5**), 83.4 ($OC(CH_3)_3$), 50.9 (C**1**), 31.9 (C**7**), 28.2 ($OC(CH_3)_3$), 26.9 (C**4**), 26.7 (C**3**), 26.6 (C**2**), 11.0 (C**8**), 4.2 (C**9**).

The signals corresponding to the pentafluorobenzoyl group could not be resolved due to their weak intensity.

δ_F (377 MHz, $CDCl_3$) -136.4 – -136.8 (2F, m), -146.7 (1F, tt, *J* = 21.0, 5.0 Hz), -159.4 – -159.6 (2F, m).

HRMS: (ESI^+) Calculated for $C_{22}H_{26}F_5NNaO_4$: 486.1674. Found $[M+Na]^+$: 486.1669.

***tert*-Butyl (*E*)-2-(2-cyclopropylvinyl)piperidine-1-carboxylate (4o)**

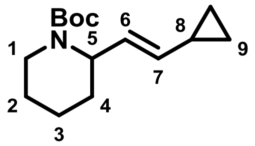

General procedure E: Conditions: 2.5 mol% $Pd_2(dba)_3$; 17.5 mol% PA-(4-(CO_2Et)C_6H_4) (**L-3**); 100 mol% Et_3N; dioxane (0.3 M); 130 °C. Substrate **2o** (116 mg, 0.250 mmol) was employed. FCC (gradient elution: 119:1 – 99:1 petroleum ether:acetone) afforded **4o** (39.4 mg, 63 %) as a colorless oil.

ν_{max} / cm^{-1}: (*film*) 2933 (m), 1689 (s), 1408 (s), 1159 (s).

δ_H (400 MHz, $CDCl_3$) 5.50 (1H, dd, *J* = 15.5, 5.0 Hz, C**6**-H), 4.98 (1H, ddd, *J* = 15.5, 8.5, 1.5 Hz, C**7**-H), 4.76 – 4.66 (1H, m, C**5**-H), 3.94 – 3.85 (1H, m, C**1**-H), 2.81 (1H, ddd, *J* = 13.0, 13.0, 3.0 Hz,

C**1**-H’), 1.70 – 1.30 (16H, m, C**2**-H_2, C**3**-H_2, C**4**-H_2, C**8**-H and OC(CH_3)$_3$), 0.72 – 0.64 (2H, m, 2 × C**9**-H), 0.37 – 0.27 (2H, m, 2 × C**9**-H’).

δ_C (101 MHz, $CDCl_3$) 155.5 (C=O), 135.7 (C**7**), 125.8 (C**6**), 79.2 (OC(CH_3)$_3$), 52.0 (C**5**), 39.7 (C**1**), 29.6 (C**4**), 28.6 (OC(CH_3)$_3$), 25.7 (C**2**), 19.6 (C**3**), 13.7 (C**8**), 6.7 (C**9**).

HRMS: (ESI$^+$) Calculated for $C_{15}H_{25}NNaO_2$: 274.1778. Found $[M+Na]^+$: 274.1785.

(*Z*)-Dec-5-en-1-ol

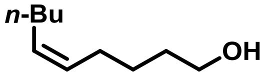

A solution of dec-5-yn-1-ol (771 mg, 5.00 mmol), Lindlar catalyst (53.2 mg, 25.0 μmol) and quinoline (6.0 μL, 50.0 μmol) in EtOAc (25 mL) was stirred under an atmosphere of H_2 (balloon pressure) for 3.5 hours. The reaction mixture was filtered through celite and the filter cake was washed with EtOAc. The filtrate was washed with 1.0 M aqueous HCl (50 mL) and brine (50 mL) before being concentrated *in vacuo* to afford the title compound (722 mg, 92 %) as a pale yellow oil.

δ_H (400 MHz, $CDCl_3$) 5.42 – 5.28 (2H, m), 3.63 (2H, t, *J* = 6.5 Hz), 2.09 – 1.98 (4H, m), 1.62 – 1.52 (3H, m), 1.41 (2H, tt, *J* = 7.5, 6.5 Hz), 1.34 – 1.26 (4H, m), 0.92 – 0.86 (3H, m).

δ_C (101 MHz, $CDCl_3$) 130.5, 129.4, 63.0, 32.5, 32.0, 27.1, 27.0, 26.0, 22.5, 14.1.

The spectroscopic properties were consistent with the data available in the literature.[28,29]

***tert*-Butyl (*Z*)-dec-5-en-1-yl((pentafluorobenzoyl)oxy)carbamate (2p)**

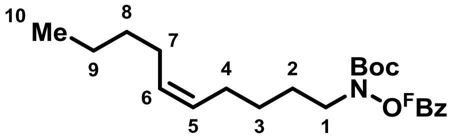

General procedure D: The preceding alcohol (932 mg, 5.96 mmol) was employed with **1a**. The reaction time was 16 hours. FCC (eluent: 2:3 hexane:PhMe) afforded **2p** (1.89 g, 68 %) as a colorless oil.

ν_{max} / cm^{-1}: (*film*) 2932 (m), 1784 (s), 1721 (s), 1652 (m), 1505 (s), 1153 (s).

δ_H (400 MHz, $CDCl_3$) 5.42 – 5.28 (2H, m, C**5**-H and C**6**-H), 3.68 (2H, t, *J* = 7.0 Hz, C**1**-H_2), 2.11 – 1.97 (4H, m, C**4**-H_2 and C**7**-H_2), 1.65 (2H, tt, *J* = 7.5, 7.0 Hz, C**2**-H_2), 1.49 (9H, s, OC(CH_3)$_3$), 1.47 – 1.38 (2H, m, C**3**-H_2), 1.34 – 1.26 (4H, m, C**8**-H_2 and C**9**-H_2), 0.92 – 0.85 (3H, m, C**10**-H_3).

δ_C (101 MHz, $CDCl_3$) 154.7 (Boc C=O), 130.7 (C**6**), 129.1 (C**5**), 83.3 (OC(CH_3)$_3$), 51.0 (C**1**), 32.0 (C**8**), 28.2 (OC(CH_3)$_3$), 27.1 (C**7**), 26.8 (C**4**), 26.7 (C**3**), 26.6 (C**2**), 22.5 (C**9**), 14.1 (C**10**).

The signals corresponding to the pentafluorobenzoyl group could not be resolved due to their weak intensity.

δ_F (377 MHz, $CDCl_3$) -136.5 – -136.8 (2F, m), -146.7 (1F, tt, *J* = 21.0, 5.0 Hz), -159.4 – -159.7 (2F, m).

HRMS: (ESI^+) Calculated for $C_{22}H_{28}F_5NNaO_4$: 488.1831. Found $[M+Na]^+$: 488.1831.

***tert*-Butyl (*E*)-2-(pent-1-en-1-yl)piperidine-1-carboxylate**

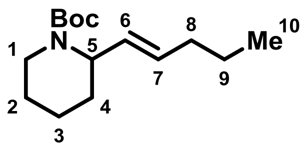

General procedure E: Conditions: 2.5 mol% $Pd_2(dba)_3$; 17.5 mol% PA-(4-(CO_2Et)C_6H_4) (**L-3**); 100 mol% Et_3N; dioxane (0.3 M); 130 °C. Substrate **2p** (116 mg, 0.250 mmol) was employed. FCC (eluent: 39:1 petroleum ether:EtOAc) afforded **4p** (39.4 mg, 62 %) as a colorless oil.

When the reaction was performed using P(4-(CF_3)C_6H_4)$_3$ under analogous conditions the yield was 9 % as determined by NMR.

ν_{max} / cm^{-1}: (*film*) 2932 (m), 2861 (m), 1693 (s), 1408 (s), 1164 (s).

δ_H (400 MHz, $CDCl_3$) 5.49 – 5.34 (2H, m, C**6**-H and C**7**-H), 4.72 (1H, br s, C**5**-H), 3.91 (1H, dd, *J* = 13.5, 4.0 Hz, C**1**-H), 2.81 (1H, ddd, *J* = 13.5, 13.0, 3.0 Hz, C**1**-H'), 2.00 (2H, td, *J* = 7.0, 6.0 Hz, C**8**-H_2), 1.69 – 1.29 (17H, m, C**2**-H_2, C**3**-H_2, C**4**-H_2, C**9**-H_2 and OC(CH_3)$_3$), 0.88 (3H, t, *J* = 7.5 Hz, C**10**-H_3).

δ_C (101 MHz, $CDCl_3$) 155.5 (Boc C=O), 131.7 (C**7**), 128.4 (C**6**), 79.2 (OC(CH_3)$_3$), 52.0 (C**5**), 39.7 (C**1**), 34.6 (C**8**), 29.6 (C**4**), 28.6 (OC(CH_3)$_3$), 25.7 (C**2**), 22.6 (C**9**), 19.6 (C**3**), 13.7 (C**10**).

HRMS: (ESI^+) Calculated for $C_{15}H_{27}NNaO_2$: 276.1934. Found $[M+Na]^+$: 276.1948.

Methyl (*Z*)-dec-5-en-1-yl((pentafluorobenzoyl)oxy)carbamate (2q)

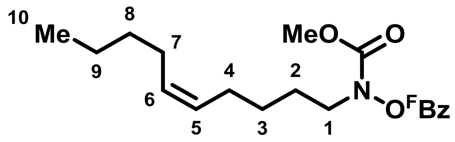

General procedure D: (*Z*)-Dec-5-en-1-ol (*vide supra*, 195 mg, 1.25 mmol) was employed with **1c**. The reaction time was 15 hours.FCC (eluent: 1:9 hexane:PhMe) afforded **2q** (352 mg, 67 %) as a pale yellow oil.

ν_{max} / cm^{-1}: (*film*) 2931 (m), 1786 (s), 1729 (s), 1652 (m), 1505 (s), 1170 (s).

δ_H (400 MHz, $CDCl_3$) 5.42 – 5.28 (2H, m, C**5**-H and C**6**-H), 3.81 (3H, s, OCH_3), 3.73 (2H, t, *J* = 7.0 Hz, C**1**-H_2), 2.11 – 1.93 (4H, m, C**4**-H_2 and C**7**-H_2), 1.66 (2H, tt, *J* = 7.5, 7.0 Hz, C**2**-H_2), 1.43 (2H, tt, *J* = 7.5, 7.5 Hz, C**3**-H_2), 1.37 – 1.24 (4H, m, C**8**-H_2 and C**9**-H_2), 0.93 – 0.83 (3H, m, C**10**-H_3).

δ_C (101 MHz, $CDCl_3$) 156.2 (Boc C=O), 130.8 (C**6**), 129.0 (C**5**), 54.1 (OCH_3), 51.3 (C**1**), 32.0 (C**8**), 27.1 (C7), 26.8 (C**4**), 2 × 26.6 (C**2** and C**3**), 22.5 (C**9**), 14.1 (C**10**).

The signals corresponding to the pentafluorobenzoyl group could not be resolved due to their weak intensity.

δ_F (377 MHz, $CDCl_3$) -136.0 – -136.2 (2F, m), -146.2 (1F, tt, J = 21.0, 5.5 Hz), -159.3 – -159.5 (2F, m).

HRMS: (ESI^+) Calculated for $C_{19}H_{22}F_5NNaO_4$: 446.1361. Found $[M+Na]^+$: 446.1362.

Methyl (*E*)-2-(pent-1-en-1-yl)piperidine-1-carboxylate (4q)

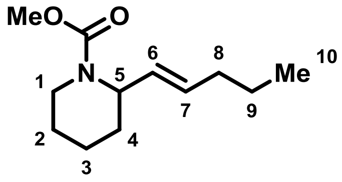

<u>General procedure E:</u> Conditions: 2.5 mol% $Pd_2(dba)_3$; 17.5 mol% PA-(4-(CO_2Et)C_6H_4) (**L-3**); 100 mol% Et_3N; dioxane (0.3 M); 130 °C. Substrate **2q** (106 mg, 0.250 mmol) was employed. FCC (*two times*, first eluent: 29:1 petroleum ether:acetone; second eluent: 149:1 PhMe:acetone) afforded **4q** (17.4 mg, 33 %) as a colorless oil.

ν_{max} / cm^{-1}: (*film*) 2933 (m), 2861 (m), 1699 (s), 1444 (s), 1261 (s).

δ_H (400 MHz, $CDCl_3$) 5.48 (1H, dtd, J = 15.5, 7.0, 1.5 Hz, C**7**-<u>H</u>), 5.39 (1H, dd, J = 15.5, 4.5 Hz, C**6**-<u>H</u>), 4.78 (1H, br s, C**5**-<u>H</u>), 3.96 (1H, br d, J = 13.5 Hz, C**1**-<u>H</u>), 3.68 (3H, s, OC<u>H</u>$_3$), 2.88 (1H, ddd, J = 13.5, 13.0, 3.0 Hz, C**1**-<u>H</u>’), 2.00 (1H, td, J = 7.5, 7.0 Hz, C**8**-<u>H</u>$_2$), 1.74 – 1.48 (5H, m, C**2**-<u>H</u>, C**3**-<u>H</u>$_2$ and C**4**-<u>H</u>$_2$), 1.46 – 1.31 (3H, m, C**2**-<u>H</u>’ and C**9**-<u>H</u>$_2$), 0.88 (3H, t, J = 7.5 Hz, C**10**-<u>H</u>$_3$).

δ_C (101 MHz, $CDCl_3$) 156.5 (<u>C</u>=O), 132.2 (C**7**), 128.2 (C**6**), 52.6 (O<u>C</u>H$_3$), 52.1 (C**5**), 40.1 (C**1**), 34.6 (C**8**), 29.6 (C**4**), 25.8 (C**2**), 22.5 (C**9**), 19.6 (C**3**), 13.8 (C**10**).

HRMS: (ESI^+) Calculated for $C_{12}H_{22}NO_2$: 212.1645. Found $[M+H]^+$: 212.1645.

1-Bromo-2-(prop-1-en-1-yl)benzene

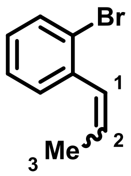

This compound was prepared according to a literature procedure[30] which afforded a 3:1 mixture of *Z* and *E* isomers.

ν_{max} / cm^{-1}: (film) 3022 (m), 2913 (m), 1467 (s), 1431 (s). 1023 (s).

Spectroscopic data for the major Z isomer:

δ_H (400 MHz, $CDCl_3$) 7.61 – 7.56 (1H, m, ArCH), 7.33 – 7.21 (2H, m, ArCH), 7.14 – 7.07 (1H, m, ArCH), 6.49 (1H, dq, *J* = 11.5, 2.0 Hz, C**1**-H), 5.90 (1H, dq, *J* = 11.5, 7.0 Hz, C**2**-H), 1.79 (3H, dd, *J* = 7.0, 2.0 Hz, C**3**-H_3).

δ_C (101 MHz, $CDCl_3$) 137.5 (ArC), 132.7 (ArCH), 130.8 (ArCH), 129.5 (C**1**), 128.3 (ArCH), 128.2 (C**2**), 126.9 (ArCH), 124.2 (ArC), 14.5 (C**3**).

Spectroscopic data for the minor E isomer:

δ_H (400 MHz, $CDCl_3$) 7.54 – 7.50 (1H, m, ArCH), 7.49 – 7.45 (1H, m, ArCH), 7.32 – 7.21 (1H, m, ArCH), 7.08 – 7.02 (1H, m, ArCH), 6.74 (1H, dq, *J* = 15.5, 2.0 Hz, C**1**-H), 6.19 (1H, dq, *J* = 15.5, 6.5 Hz, C**2**-H), 1.93 (3H, dd, *J* = 6.5, 2.0 Hz, C**3**-H_3).

δ_C (101 MHz, $CDCl_3$) 137.8 (ArC), 132.9 (ArCH), 130.0 (C**1**), 129.0 (C**2**), 128.2 (ArCH), 127.5 (ArCH), 126.9 (ArCH), 123.1 (ArC), 18.8 (C**3**).

HRMS: (EI^+) Calculated for C_9H_9Br: 195.9888. Found $[M]^+$: 195.9986.

2-(2-(Prop-1-en-1-yl)phenyl)ethan-1-ol

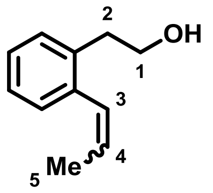

To a solution of the preceding bromide (2.96 g, 15.0 mmol) in anhydrous THF (40 mL) at -78 °C was added *n*-BuLi (1.55 M in hexane, 10.6 mL, 16.5 mmol). The reaction mixture was stirred at -78 °C for 2 hours before addition ethylene oxide (*approx.* 3 M in THF, 7.5 mL, 22.5 mmol). The reaction mixture was slowly warmed to room temperature and stirred for 18 hours before addition of saturated aqueous NH_4Cl (30 mL). The resulting phases were separated and the aqueous phase was extracted with Et_2O (2 × 40 mL). The organic phase was dried over Na_2SO_4 and concentrated *in vacuo*. FCC (eluent: 3:1 hexane:EtOAc) afforded the title compound (1.50 g, 62 %, 3:1 mixture of *Z* and *E* isomers) as a pale yellow oil.

ν_{max} / cm^{-1}: (film) 3322 (br s), 3017 (m), 2937 (m), 2876 (m), 1484 (s), 1446 (s), 1041 (s).

Spectroscopic data for the major Z isomer:

δ_H (400 MHz, $CDCl_3$) 7.25 – 7.15 (4H, m, ArCH), 6.55 (1H, dq, *J* = 11.5, 2.0 Hz, C**3**-H), 5.86 (1H, dq, *J* = 11.5, 7.0 Hz, C**4**-H), 3.78 (2H, t, *J* = 7.0 Hz, C**1**-H_2), 2.88 (2H, t, *J* = 7.0 Hz, C**2**-H_2), 1.73 (3H, dd, *J* = 7.0, 2.0 Hz, C**5**-H_3), 1.46 (1H, br s, OH).

δ_C (101 MHz, $CDCl_3$) 136.9 (ArC), 136.6 (ArC), 2 × 130.0 (2 × ArCH), 128.7 (C**3**), 127.8 (C**4**), 127.1 (ArCH), 126.3 (ArCH), 63.1 (C**1**), 36.9 (C**2**), 14.4 (C**5**).

Spectroscopic data for the minor E isomer:

δ_H (400 MHz, $CDCl_3$) 7.46 – 7.41 (1H, m, ArC<u>H</u>), 7.25 – 7.15 (3H, m, ArC<u>H</u>), 6.66 (1H, dq, *J* = 15.5, 2.0 Hz, C**3**-<u>H</u>), 6.12 (1H, dq, *J* = 15.5, 6.5 Hz, C**4**-<u>H</u>), 3.82 (2H, t, *J* = 7.0 Hz, C**1**-<u>H</u>$_2$), 2.96 (2H, t, *J* = 7.0 Hz, C**2**-<u>H</u>$_2$), 1.91 (3H, dd, *J* = 6.5, 2.0 Hz, C**5**-<u>H</u>$_3$), 1.46 (1H, br s, O<u>H</u>).

δ_C (101 MHz, $CDCl_3$) 137.5 (Ar<u>C</u>), 135.0 (Ar<u>C</u>), 130.3 (Ar<u>C</u>H), 128.5 (C**3**), 128.0 (C**4**), 127.1 (Ar<u>C</u>H), 127.0 (Ar<u>C</u>H), 126.3 (Ar<u>C</u>H), 63.3 (C**1**), 36.9 (C**2**), 18.9 (C**5**).

HRMS: (ESI^+) Calculated for $C_{11}H_{14}NaO$: 185.0937. Found $[M+Na]^+$: 185.0930.

***tert*-Butyl (2-(prop-1-en-1-yl)phenethyl)((pentafluorobenzoyl)oxy)carbamate (2r)**

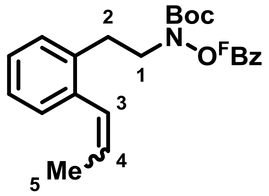

<u>General procedure D:</u> The preceding alcohol (324 g, 2.00 mmol) was employed with **1a**. The reaction time was 16 hours. FCC (eluent: 2:3 hexane:PhMe) afforded **2r** (493 mg, 52 %, 3:1 mixture of *Z* and *E* isomers) as a colorless oil.

ν_{max} / cm^{-1}: (*film*) 3020 (m), 2981 (m), 1783 (s), 1722 (s), 1652 (m), 1499 (s), 1326 (s), 1151 (s).

Spectroscopic data for the major Z isomer:

δ_H (400 MHz, $CDCl_3$) 7.24 – 7.12 (4H, m, ArC<u>H</u>), 6.53 (1H, dq, *J* = 11.5, 2.0 Hz, C**3**-<u>H</u>), 5.86 (1H, dq, *J* = 11.5, 7.0 Hz, C**4**-<u>H</u>), 3.88 – 3.77 (2H, m, C**1**-<u>H</u>$_2$), 3.00 – 2.92 (2H, m, C**2**-<u>H</u>$_2$), 1.71 (3H, dd, *J* = 7.0, 2.0 Hz, C**5**-<u>H</u>$_3$), 1.46 (9H, s, OC(C<u>H</u>$_3$)$_3$).

δ_C (101 MHz, $CDCl_3$) 154.2 (Boc <u>C</u>=O), 136.6 (Ar<u>C</u>), 136.0 (Ar<u>C</u>), 2 × 129.7 (2 × Ar<u>C</u>H), 2 × 128.0 (C**3** and C**4**), 127.1 (Ar<u>C</u>H), 126.3(Ar<u>C</u>H), 83.3 (O<u>C</u>(CH$_3$)$_3$), 51.4 (C**1**), 31.1 (C**2**), 28.0 (OC(<u>C</u>H$_3$)$_3$), 14.3 (C**5**).

The signals corresponding to the pentafluorobenzoyl group could not be resolved due to their weak intensity.

Spectroscopic data for the minor E isomer:

δ_H (400 MHz, $CDCl_3$) 7.41 (1H, d, *J* = 7.0 Hz, ArC<u>H</u>), 7.24 – 7.12 (3H, m, ArC<u>H</u>), 6.64 (1H, dq, *J* = 15.5, 1.5 Hz, C**3**-<u>H</u>), 6.13 (1H, dq, *J* = 15.5, 6.5 Hz, C**4**-<u>H</u>), 3.88 – 3.77 (2H, m, C**1**-<u>H</u>$_2$), 3.07 – 3.00 (2H, m, C**2**-<u>H</u>$_2$), 1.89 (3H, dd, *J* = 6.5, 1.5 Hz, C**5**-<u>H</u>$_3$), 1.46 (9H, s, OC(C<u>H</u>$_3$)$_3$).

δ_C (101 MHz, $CDCl_3$) 154.2 (Boc <u>C</u>=O), 137.2 (Ar<u>C</u>), 134.4 (Ar<u>C</u>), 130.1 (Ar<u>C</u>H), 128.2 (C**4**), 127.9 (C**3**), 127.1 (Ar<u>C</u>H), 127.0 (Ar<u>C</u>H), 126.1 (Ar<u>C</u>H), 83.3 (O<u>C</u>(CH$_3$)$_3$), 51.7 (C**1**), 30.8 (C**2**), 28.0 (OC(<u>C</u>H$_3$)$_3$), 18.7 (C**5**).

The signals corresponding to the pentafluorobenzoyl group could not be resolved due to their weak intensity.

^{19}F signals for both isomers:

δ_F (377 MHz, $CDCl_3$) -136.2 – -136.4 (2F, m), -146.2 – -146.5 (1F, m), -159.2 – -159.4 (2F, m).

HRMS: (ESI$^+$) Calculated for $C_{23}H_{22}F_5NNaO_4$: 494.1361. Found [M+Na]$^+$: 494.1362.

***tert*-Butyl 1-vinyl-3,4-dihydroisoquinoline-2(1*H*)-carboxylate (4r)**

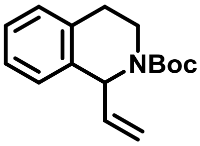

General procedure E: Conditions: 2.5 mol% $Pd_2(dba)_3$; 15 mol% PA-(4-(CF_3)C_6H_4) (**L-2**); 100 mol% Et_3N; dioxane (0.4 M); 130 °C. Substrate **2r** (49.5 mg, 0.105 mmol) was employed. FCC (*two times*, first eluent: 79:1 pentane:acetone; second eluent: PhMe) afforded **4r** (16.8 mg, 62 %) as a colorless oil.

δ_H (400 MHz, $CDCl_3$) 7.20 – 7.11 (4H, m), 5.96 (1H, ddd, *J* = 17.0, 10.0, 5.5 Hz), 5.55 (1H, br s), 5.15 (1H, ddd, *J* = 10.0, 1.5, 1.5 Hz), 5.05 (1H, ddd, *J* = 17.0, 1.5, 1.5 Hz), 4.11 (1H, br s), 3.21 (1H, br s), 2.98 – 2.83 (1H, m), 2.73 (1H, ddd, *J* = 16.0, 4.0, 4.0 Hz), 1.49 (9H, s).

δ_C (126 MHz, $CDCl_3$) 154.9, 137.9, 135.1, 135.0, 129.0, 128.0, 126.8, 126.2, 115.8, 80.0, 57.3, 37.8, 28.9, 28.6.

The spectroscopic properties were consistent with the data available in the literature.[31]

Methyl 2-oxotetrahydrofuran-3-carboxylate

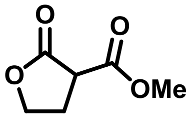

To a suspension of sodium hydride (60 % in mineral oil, 1.44 g, 36.0 mmol) in dimethyl carbonate (2.53 mL, 30.0 mmol) and anhydrous THF (25 mL) was added γ-butyrolactone (2.31 mL, 30.0 mmol) dropwise. The reaction mixture was heated at reflux for 16 hours before cooling to room temperature, addition of 0.5 M aqueous HCl (40 mL) and extraction with MTBE (3 × 50 mL). The organic phase was dried over Na_2SO_4 and concentrated *in vacuo* to afford the title compound (1.97 g, 68 %) as a pale yellow oil.

δ_H (400 MHz, $CDCl_3$) 4.46 (1H, ddd, *J* = 9.0, 8.0, 5.5 Hz), 4.31 (1H, ddd, *J* = 9.0, 7.5, 7.5 Hz), 3.79 (3H, s), 3.56 (1H, dd, *J* = 9.5, 8.0 Hz), 2.66 (1H, dddd, *J* = 13.0, 8.0, 8.0, 7.5 Hz), 2.50 (1H, dddd, *J* = 13.0, 9.5, 7.5, 5.5 Hz).

δ_C (101 MHz, $CDCl_3$) 172.4, 168.3, 67.5, 53.2, 45.9, 26.5.

The spectroscopic properties were consistent with the data available in the literature.[32]

Methyl (*E*)-3-(but-2-en-1-yl)-2-oxotetrahydrofuran-3-carboxylate

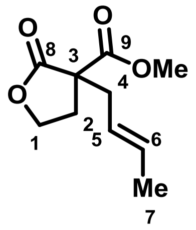

To a suspension of sodium hydride (60 % in mineral oil, 1.03 g, 25.7 mmol) in anhydrous THF (50 mL) at 0 °C was added a solution of the preceding dicarbonyl compound (3.71 g, 25.7 mmol) in anhydrous THF (15 mL). The reaction mixture was stirred at 0 °C for an hour before addition of crotyl bromide (2.91 mL, 28.3 mmol). The reaction mixture was stirred at room temperature for 4 hours before addition of 0.5 M aqueous HCl (100 mL), the resulting phases were separated and the aqueous phase was extracted with CH_2Cl_2 (2 × 50 mL). The organic phase was dried over Na_2SO_4 and concentrated *in vacuo*. FCC (gradient elution: 6:1 – 2:1) afforded the title compound (3.33 g, 65 %) as a colorless oil.

ν_{max} / cm^{-1}: (*film*) 2956 (m), 1771 (s), 1731 (s), 1164 (s), 1025 (s).

δ_H (500 MHz, $CDCl_3$) 5.61 (1H, dqdd, *J* = 15.0, 6.5, 1.0 Hz, C**6**-H), 5.31 (1H, dddq, *J* = 15.0, 7.5, 7.0, 1.5 Hz, C**5**-H), 4.36 – 4.26 (2H, m, C**1**-H_2), 3.77 (3H, s, OCH_3), 2.73 – 2.62 (2H, m, C**2**-H and C**4**-H), 2.54 (1H, ddqd, *J* = 14.0, 7.0, 1.5, 1.0 Hz, C**4**-H'), 2.30 (1H, dt, *J* = 13.0, 8.5 Hz, C**2**-H'), 1.67 (3H, dddd, *J* = 6.5, 1.5, 1.5, 1.5 Hz, C**7**-H_3).

δ_C (101 MHz, $CDCl_3$) 174.7 (C**8**), 170.2 (C**9**), 131.4 (C**6**), 124.2 (C**5**), 66.3 (C**1**), 54.2 (C**3**), 53.3 (OCH_3), 37.2 (C**4**), 31.0 (C**2**), 18.2 (C**7**).

HRMS: (ESI^+) Calculated for $C_{10}H_{14}NaO_4$: 221.0784. Found $[M+Na]^+$: 221.0786.

(*E*)-2-(But-2-en-1-yl)-2-(hydroxymethyl)butane-1,4-diol

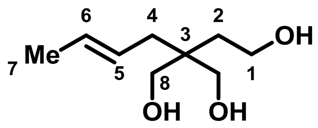

General procedure C: The preceding ester (3.27 g, 16.5 mmol) was employed using anhydrous THF as solvent and 2.0 eq. $LiAlH_4$ (1.0 M in THF). The title compound (2.04 g, 71 %) was isolated as a colorless oil.

ν_{max} / cm^{-1}: (*film*) 3317 (br s), 2919 (s), 1439 (m), 1034 (s).

δ_H (400 MHz, $CDCl_3$) 5.54 – 5.43 (1H, m, C**6**-H), 5.43 – 5.33 (1H, m, C**5**-H), 3.78 (2H, t, *J* = 5.5 Hz, C**1**-H_2), 3.56 (4H, s, 2 × C**8**-H_2), 2.91 (3H, br s, 3 × OH), 1.95 (2H, d, *J* = 7.0 Hz, C**4**-H_2), 1.72 – 1.64 (5H, m, C**2**-H_2 and C**7**-H_3).

δ_C (101 MHz, $CDCl_3$) 129.0 (C**6**), 126.0 (C**5**), 68.1 (C**8**), 58.7 (C**1**), 42.0 (C**3**), 36.4 (C**4**), 35.3 (C**2**), 18.2 (C**7**).

HRMS: (ESI^+) Calculated for $C_9H_{18}NaO_3$: 197.1148. Found $[M+Na]^+$: 197.1154.

(*E*)-2-(5-(But-2-en-1-yl)-1,3-dioxan-5-yl)ethan-1-ol

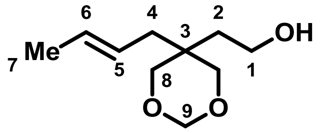

This compound was prepared using an adaptation of a literature procedure.[33]

A solution of the preceding alcohol (375 mg, 2.15 mmol), lithium bromide (37.3 mg, 430 μmol), $TsOH \cdot H_2O$ (40.9 mg, 215 μmol) and dimethoxymethane (0.65 mL, 7.35 mmol) in CH_2Cl_2 (1.1 mL) was stirred at room temperature for 18 hours. The reaction mixture was heated at 110 °C for 10 minutes before being cooled to room temperature, the resulting solvent-free material was purified by FCC (gradient elution: 9:1 – 2:1 hexane:EtOAc) to afford the title compound (148 mg, 37 %) as a colorless oil.

ν_{max} / cm^{-1}: (*film*) 3401 (br s), 2853 (m), 2768 (m), 1452 (m), 1165 (s), 1027 (s).

δ_H (500 MHz, $CDCl_3$) 5.50 (1H, dqt, *J* = 15.0, 6.5, 1.0 Hz, C**6**-H), 5.34 (1H, dtq, *J* = 15.0, 7.5, 1.5 Hz, C**5**-H), 4.90 (1H, d, *J* = 6.0 Hz, C**9**-H), 4.73 (1H, d, *J* = 6.0 Hz, C**9**-H'), 3.84 – 3.79 (2H, m, C**1**-H_2), 3.72 (2H, d, *J* = 11.5 Hz, 2 × C**8**-H), 3.59 (2H, d, *J* = 11.5 Hz, 2 × C**8**-H'), 2.38 (1H, br s, OH), 2.01 (2H, br d, *J* = 7.5 Hz, C**4**-H_2), 1.69 – 1.65 (5H, m, C**2**-H_2 and C**7**-H_3).

δ_C (126 MHz, $CDCl_3$) 129.5 (C**6**), 124.8 (C**5**), 94.1 (C**9**), 75.0 (C**8**), 59.1 (C**1**), 2 × 37.5 (C**2** and C**4**), 35.7 (C**3**), 18.2 (C**7**).

HRMS: (ESI^+) Calculated for $C_{10}H_{18}NaO_3$: 209.1148. Found $[M+Na]^+$: 209.1157.

***tert*-Butyl (*E*)-(2-(5-(but-2-en-1-yl)-1,3-dioxan-5-yl)ethyl)((pentafluorobenzoyl)oxy)carbamate (2s)**

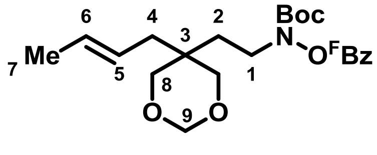

General procedure D: The preceding alcohol (587 mg, 3.15 mmol) was employed with **1a**. The reaction time was 16 hours. FCC (gradient elution: 1:0 – 19:1 PhMe:EtOAc) afforded **2s** (671 mg, 43 %) as a pale yellow oil.

ν_{max} / cm^{-1}: (*film*) 2980 (m), 2851 (m), 2727 (m), 1783 (s), 1721 (s), 1653 (m), 1504 (s), 1149 (s).

δ_H (500 MHz, $CDCl_3$) 5.58 – 5.49 (1H, m, C**6**-H), 5.36 (1H, dtq, *J* = 15.0, 7.5, 1.5 Hz, C**5**-H), 4.82 (1H, d, *J* = 6.0 Hz, C**9**-H), 4.77 (1H, d, *J* = 6.0 Hz, C**9**-H'), 3.79 – 3.73 (2H, m, C**1**-H_2), 3.64 – 3.56 (4H, m, 2 × C**8**-H_2), 2.10 (2H, d, *J* = 7.5 Hz, C**4**-H_2), 1.78 – 1.71 (2H, m, C**2**-H_2), 1.68 – 1.64 (3H, m, C**7**-H_3), 1.50 (9H, s, $OC(CH_3)_3$).

δ_C (126 MHz, $CDCl_3$) 154.6 (Boc C=O), 129.8 (C**6**), 124.5 (C**5**), 94.4 (C**9**), 83.7 ($OC(CH_3)_3$), 74.4 (C**8**), 46.7 (C**1**), 2 × 35.5 (C**3** and C**4**), 29.5 (C**2**), 28.2 ($OC(CH_3)_3$), 18.1 (C**7**).

The signals corresponding to the pentafluorobenzoyl group could not be resolved due to their weak intensity.

δ_F (470 MHz, $CDCl_3$) -136.4 – -136.6 (2F, m), -146.4 (1F, tt, *J* = 21.0, 5.0 Hz), -159.2 – -159.4 (2F, m).

HRMS: (ESI^+) Calculated for $C_{22}H_{26}F_5NNaO_6$: 518.1572. Found $[M+Na]^+$: 518.1574.

***tert*-Butyl 8-vinyl-2,4-dioxa-9-azaspiro[5.5]undecane-9-carboxylate (4s)**

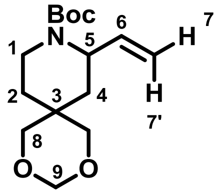

General procedure E: Conditions: 2.5 mol% $Pd_2(dba)_3$; 15 mol% PA-(4-(CF_3)C_6H_4) (**XX**); 100 mol% Et_3N; THF (0.4 M); 130 °C. Substrate **2s** (52.0 mg, 0.105 mmol) was employed. FCC (eluent: 19:1 petroleum ether:acetone) afforded **4s** (20.4 mg, 69 %) as a colorless oil.

ν_{max} / cm^{-1}: (*film*) 2977 (m), 2845 (m), 2765 (m), 1688 (s), 1407 (s), 1157 (s).

δ_H (400 MHz, $CDCl_3$) 5.72 (1H, ddd, *J* = 17.5, 10.5, 3.5 Hz, C**6**-H), 5.11 (1H, ddd, *J* = 10.5, 2.5, 1.0 Hz, C**7**-H), 4.99 (1H, ddd, *J* = 17.5, 2.5, 1.0 Hz, C**7**-H'), 4.81 (1H, d, *J* = 6.0 Hz, C**9**-H), 4.77 (1H, d, *J* = 6.0 Hz, C**9**-H'), 4.76 – 4.71 (1H, m, C**5**-H), 4.00 – 3.88 (1H, m, C**1**-H), 3.79 (1H, d, *J* = 11.5 Hz, C**8**-H), 3.70 (1H, d, *J* = 11.5 Hz, C**8**-H'), 3.48 (1H, d, *J* = 11.0 Hz, C**8'**-H), 3.41 (1H, d, *J* = 11.0 Hz, C**8'**-H'), 2.95 (1H, ddd, *J* = 13.5, 13.0, 3.0 Hz, C**1**-H'), 1.84 – 1.74 (2H, m, C**2**-H and C**4**-H), 1.53 (1H, dd, *J* = 14.0, 7.0 Hz, C**4**-H'), 1.44 (9H, s, $OC(CH_3)_3$), 1.29 (1H, ddd, *J* = 13.0, 13.0, 5.5 Hz, C**2**-H').

δ_C (101 MHz, $CDCl_3$) 155.4 (C=O), 138.2 (C**6**), 114.0 (C**7**), 94.5 (C**9**), 79.9 ($OC(CH_3)_3$), 77.7 (C**8'**), 73.3 (C**8**), 51.1 (C**5**), 35.5 (C**1**), 33.8 (C**4**), 32.5 (C**3**), 30.0 (C**2**), 28.5 ($OC(CH_3)_3$).

HRMS: (ESI^+) Calculated for $C_{15}H_{25}NNaO_4$: 306.1676. Found $[M+Na]^+$: 306.1679.

8-Oxaspiro[4.5]decan-7-one

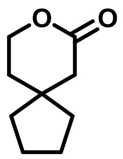

This compound was prepared according to a literature procedure.[34]

δ_H (400 MHz, $CDCl_3$) 4.36 (2H, t, *J* = 6.0 Hz), 2.41 (2H, s), 1.76 (2H, t, *J* = 6.0 Hz), 1.72 – 1.64 (4H, m), 1.59 – 1.45 (4H, m).

δ_C (101 MHz, $CDCl_3$) 171.4, 67.4, 42.5, 40.8, 38.7, 34.4, 23.8.

The spectroscopic properties were consistent with the data available in the literature.[35]

8-Oxaspiro[4.5]decan-7-ol

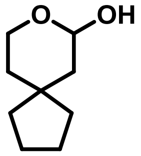

To a solution of the preceding lactone (2.31 g, 15.0 mmol) in anhydrous CH_2Cl_2 (35 mL) at -78 °C was added DiBAl-H (1.0 M in CH_2Cl_2, 16.5 mL, 16.5 mmol). The reaction mixture was stirred at -78 °C for 2.5 hours before being diluted with Et_2O and warmed to 0 °C. The reaction mixture was quenched slowly with water (0.65 mL), 4.0 M aqueous NaOH (0.65 mL) and a further portion of water (1.65 mL) before warming to room temperature. The reaction mixture was stirred for 20 minutes before being dried over Na_2SO_4, filtered and concentrated *in vacuo*. FCC (gradient elution: 9:1 – 6:1 PhMe:acetone) to afford the title compound (2.02 g, 86 %) as a colorless oil.

δ_H (400 MHz, $CDCl_3$) 4.87 – 4.81 (1H, m), 3.94 (1H, ddd, *J* = 11.5, 4.0, 4.0 Hz), 3.59 (1H, ddd, *J* = 11.5, 10.5, 2.5 Hz), 3.34 – 3.13 (1H, m), 1.74 – 1.28 (12H, m).

δ_C (101 MHz, $CDCl_3$) 94.1, 62.4, 44.2, 41.2, 40.9, 36.5, 36.3, 24.3, 23.7.

The spectroscopic properties were consistent with the data available in the literature.[35]

Methyl (*E*)-4-(1-(2-hydroxyethyl)cyclopentyl)but-2-enoate

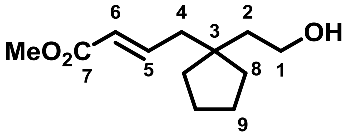

A solution of the preceding hemiacetal (1.56 g, 10.0 mmol) and methyl (triphenylphosphoranylidene)acetate (5.02 g, 15.0 mmol) in anhydrous PhMe (50 mL) was heated at reflux for 5 hours. The reaction mixture was cooled to room temperature and concentrated *in vacuo*. FCC (eluent: 4:1 petroleum ether:acetone) afforded the title compound (1.82 g, 86 %, 9:1 mixture of *E* and *Z* isomers) as a colorless oil.

ν_{max} / cm^{-1}: (*film*) 3417 (br s), 2948 (m), 1721 (s), 1436 (s), 1166 (s).

Spectroscopic data for the major E isomer:

δ_H (400 MHz, $CDCl_3$) 6.97 (1H, dt, *J* = 15.5, 7.5 Hz, C**5**-H), 5.84 (1H, dt, *J* = 15.5, 1.5 Hz, C**6**-H), 3.72 (3H, s, OCH_3), 3.71 – 3.66 (2H, m, C**1**-H_2), 2.19 (2H, dd, *J* = 7.5, 1.5 Hz, C**4**-H_2), 1.66 – 1.56 (6H, m, C**2**-H_2 and 2 × C**9**-H_2), 1.50 – 1.41 (4H, m, 2 × C**8**-H_2), 1.38 (1H, br s, OH).

δ_C (101 MHz, $CDCl_3$) 167.0 (C**7**), 147.2 (C**5**), 123.1 (C**6**), 60.1 (C**1**), 51.6 (OCH_3), 44.5 (C**3**), 41.9 (C**2**), 41.6 (C**4**), 37.8 (C**8**), 24.5 (C**9**).

HRMS: (ESI^+) Calculated for $C_{12}H_{20}NaO_3$: 235.1305. Found $[M+Na]^+$: 235.1305.

Characteristic signals for the minor Z isomer:

δ_H (400 MHz, $CDCl_3$) 6.29 (1H, dt, *J* = 11.5, 7.5 Hz), 2.69 (2H, dd, *J* = 7.5, 2.0 Hz).

δ_C (101 MHz, $CDCl_3$) 148.2, 120.8, 37.4.

Methyl (*E*)-4-(1-(2-((*tert*-butoxycarbonyl)((pentafluorobenzoyl)oxy)amino)ethyl)cyclopentyl)but-2-enoate (2t)

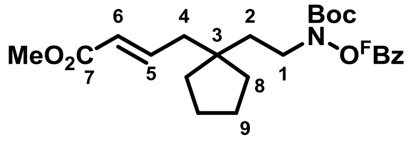

General procedure D: The preceding alcohol (531 mg, 2.50 mmol) was employed with **1a**. The reaction time was 16 hours. FCC (gradient elution: 1:0 – 49:1 PhMe:EtOAc) afforded **2t** (859 mg, 66 %) as a colorless oil.

The E and Z isomers were separable by FCC and hence this compound was isolated as a single alkene isomer.

ν_{max} / cm^{-1}: (film) 2952 (m), 1783 (s), 1722 (s), 1654 (m), 1506 (s), 1158 (s).

δ_H (400 MHz, $CDCl_3$) 6.93 (1H, dt, *J* = 15.5, 8.0 Hz, C**5**-H), 5.85 (1H, dt, *J* = 15.5, 1.5 Hz, C**6**-H), 3.71 (3H, s, OCH_3), 3.70 – 3.65 (2H, m, C**1**-H_2), 2.20 (2H, dd, *J* = 8.0, 1.5 Hz, C**4**-H_2), 1.70 – 1.60 (6H, m, C**2**-H_2 and 2 × C**9**-H_2), 1.52 – 1.43 (13H, m, 2 × C**8**-H_2 and $OC(CH_3)_3$).

δ_C (101 MHz, $CDCl_3$) 166.8 (C**7**), 154.6 (Boc C=O), 146.3 (C**5**), 123.4 (C**6**), 83.6 ($OC(CH_3)_3$), 51.5 (OCH_3), 47.9 (C**1**), 44.3 (C**3**), 41.1 (C**4**), 37.5 (C**8**), 35.4 (C**2**), 28.2 ($OC(CH_3)_3$), 24.6 (C**9**).

The signals corresponding to the pentafluorobenzoyl group could not be resolved due to their weak intensity.

δ_F (377 MHz, $CDCl_3$) -136.5 – -136.7 (2F, m), -146.7 (1F, tt, *J* = 21.0, 5.0 Hz), -159.4 – -159.5 (2F, m).

HRMS: (ESI^+) Calculated for $C_{24}H_{28}F_5NNaO_6$: 544.1729. Found $[M+Na]^+$: 544.1722.

tert-Butyl (_E_)-7-(2-methoxy-2-oxoethylidene)-8-azaspiro[4.5]decane-8-carboxylate (4t)

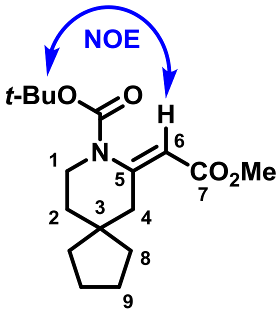

General procedure E: Conditions: 2.5 mol% $Pd_2(dba)_3$; 15 mol% PA-(4-(CF_3)C_6H_4) (**L-2**); 300 mol% Et_3N; THF (0.4 M); 130 °C, 8 hours. Substrate **2t** (54.8 mg, 0.105 mmol) was employed. FCC (*two times*, first, gradient elution: 1:0 – 149:1 PhMe:acetone; second eluent: 24:1 petroleum ether:acetone) afforded **4t** (21.7 mg, 67 %) as a colorless oil.

*The product was assigned as the E isomer based on the observed NOE correlation between the C***6** *and the t-Bu protons.*

ν_{max} / cm^{-1}: (*film*) 2949 (m), 1698 (s), 1365 (s), 1163 (s).

δ_H (400 MHz, $CDCl_3$) 5.96 (1H, s, C**6**-H), 3.67 (3H, s, OCH_3), 3.61 – 3.57 (2H, m, C**1**-H_2), 2.87 (2H, s, C**4**-H_2), 1.74 – 1.56 (6H, m, C**2**-H_2 and 2 × C**9**-H_2), 1.55 – 1.44 (11H, m, 2 × C**8**-H and OC(CH_3)$_3$), 1.42 – 1.33 (2H, m, 2 × C**8**-H').

δ_C (101 MHz, $CDCl_3$) 168.0 (C**7**), 155.2 (C**5**), 153.4 (Boc C=O), 111.0 (C**6**), 81.3 (OC(CH_3)$_3$), 51.1 (OCH_3), 44.2 (C**1**), 43.2 (C**3**), 38.5 (C**4**), 38.2 (C**8**), 36.5 (C**2**), 28.4 (OC(CH_3)$_3$), 24.3 (C**9**).

HRMS: (ESI^+) Calculated for $C_{17}H_{27}NNaO_4$: 332.1823. Found $[M+Na]^+$: 332.1838.

Methyl (_E_)-3-(2-(2-hydroxyethyl)phenyl)acrylate

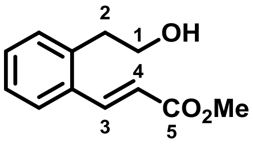

This compound was prepared according to a literature procedure.[36]

A solution of 2-bromophenethyl alcohol (2.01 g, 10.0 mmol), $Pd(OAc)_2$ (112 mg, 500 µmol), tri(*o*-tolyl)phosphine (304 mg, 1.00 mmol) and methyl acrylate (1.35 mL, 15.0 mmol) in anhydrous Et_3N (7.0 mL) was sparged with argon for five minutes. The reaction mixture was heated at 95 °C in a sealed tube for 19 hours before being filtered through celite, the filter cake was rinsed with EtOAc and the filtrate was concentrated *in vacuo*. FCC (gradient elution: 2:1 – 3:2 petroleum ether:EtOAc) afforded the title compound (1.57 g, 76 %) as a pale yellow oil.

ν_{max} / cm^{-1}: (*film*) 3406 (br s), 2950 (m), 1698 (s), 1630 (s), 1318 (s), 1170 (s).

δ_H (400 MHz, $CDCl_3$) 8.02 (1H, d, *J* = 16.0 Hz, C**3**-H), 7.60 – 7.57 (1H, m, ArCH), 7.37 – 7.31 (1H, m, ArCH), 7.29 – 7.24 (2H, m, ArCH), 6.38 (1H, d, *J* = 16.0 Hz, C**4**-H), 3.87 – 3.78 (5H, m, C**1**-H_2 and OCH_3), 3.04 (2H, t, *J* = 7.0 Hz, C**2**-H_2), 1.71 – 1.64 (1H, br s, OH).

δ_C (101 MHz, $CDCl_3$) 167.5 (C**5**), 142.2 (C**3**), 138.2 (ArC), 133.7 (ArC), 131.0 (ArCH), 130.3 (ArCH), 127.3 (ArCH), 127.0 (ArCH), 119.7 (C**4**), 63.5 (C**1**), 51.9 (OCH_3), 36.6 (C**2**).

HRMS: (ESI^+) Calculated for $C_{12}H_{14}NaO_3$: 229.0835. Found $[M+Na]^+$: 229.0845.

Methyl (*E*)-3-(2-(2-((pentafluorobenzoyloxy)(*tert*-butoxycarbonyl)amino)ethyl)phenyl)acrylate (2u)

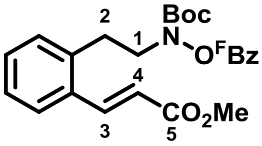

General procedure D: The preceding alcohol (619 mg, 3.00 mmol) was employed with **1a**. The reaction time was 16 hours. FCC (gradient elution: 99:1 – 19:1 PhMe:EtOAc) afforded **2u** (1.07 g, 69 %) as a pale yellow oil.

ν_{max} / cm^{-1}: (*film*) 2982 (m), 1783 (s), 1716 (s), 1652 (m), 1634 (m), 1500 (s), 1150 (s).

δ_H (400 MHz, $CDCl_3$) 7.97 (1H, d, *J* = 16.0 Hz, C**3**-H), 7.58 – 7.54 (1H, m, ArCH), 7.35 – 7.29 (1H, m, ArCH), 7.29 – 7.23 (2H, m, ArCH), 6.37 (1H, d, *J* = 16.0 Hz, C**4**-H), 3.87 (2H, t, *J* = 7.5 Hz, C**1**-H_2), 3.77 (3H, s, OCH_3), 3.12 (2H, t, *J* = 7.5 Hz, C**2**-H_2), 1.42 (9H, s, $OC(CH_3)_3$).

δ_C (101 MHz, $CDCl_3$) 167.0 (C**5**), 154.0 (Boc C=O), 141.4 (C**3**), 137.3 (ArC), 133.5 (ArC), 130.7 (ArCH), 130.2 (ArCH), 127.4 (ArCH), 126.8 (ArCH), 120.0 (C**4**), 83.4 ($OC(CH_3)_3$), 51.8 (C**1**), 51.6 (OCH_3), 30.6 (C**2**), 27.9 ($OC(CH_3)_3$).

The signals corresponding to the pentafluorobenzoyl group could not be resolved due to their weak intensity.

δ_F (377 MHz, $CDCl_3$) -136.1 – -136.4 (2F, m), -146.6 (1F, tt, *J* = 21.0, 5.5 Hz), -159.5 – -159.7 (2F, m).

HRMS: (ESI^+) Calculated for $C_{24}H_{22}F_5NNaO_6$: 538.1259. Found $[M+Na]^+$: 538.1272.

***tert*-Butyl (*Z*)-1-(2-methoxy-2-oxoethylidene)-3,4-dihydroisoquinoline-2(1*H*)-carboxylate (4u)**

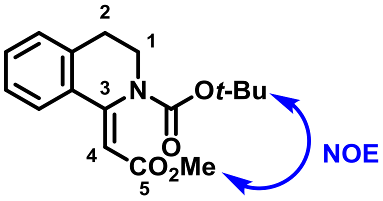

General procedure E: Conditions: 2.5 mol% $Pd_2(dba)_3$; 15 mol% PA-(4-(CF_3)C_6H_4 (**L-2**); 100 mol% Et_3N; THF (0.4 M); 130 °C. Substrate **2u** (54.1 mg, 0.105 mmol) was employed. FCC (gradient elution: 49:1 - 19:1 PhMe:EtOAc) afforded **4u** (23.9 mg, 75 %) as a pale yellow oil.

The product was assigned as the Z isomer based on the observed NOE correlation between the Me and the t-Bu protons.

When the reaction is run to partial completion:

General procedure E: Conditions: 2.5 mol% $Pd_2(dba)_3$; 15 mol% PA-(4-(CF_3)C_6H_4 (**L-2**); 500 mol% Et_3N; THF (0.4 M); 130 °C, 3 hours. Substrate **2u** (54.1 mg, 0.105 mmol) was employed. By NMR analysis of the crude product, **4u** was found to be produced as a 14:1 mixture of *E* and *Z* isomers. FCC (eluent: 29:1 PhMe:EtOAc) afforded exclusively the *Z* isomer of **4u** (23.9 mg, 75 %) as a colorless oil.

ν_{max} / cm^{-1}: (*film*) 2976 (m), 2929 (m), 1698 (s), 1629 (s), 1149 (s).

δ_H (400 MHz, $CDCl_3$) 7.69 – 7.65 (1H, m, ArCH), 7.33 – 7.20 (2H, m, ArCH), 7.18 – 7.14 (1H, m, ArCH), 6.30 (1H, s, C**4**-H), 4.24 – 3.54 (2H, br s, C**1**-H_2), 3.74 (3H, s, OCH_3), 2.90 (2H, br s, C**2**-H_2), 1.43 (9H, s, $OC(CH_3)_3$).

δ_C (101 MHz, $CDCl_3$) 166.4 (C**5**), 152.9 (Boc C=O), 145.8 (C**3**), 136.7 (ArC), 131.6 (ArC), 129.9 (ArCH), 129.3 (ArCH), 126.9 (ArCH), 124.5 (ArCH), 106.9 (C**4**), 81.3 ($OC(CH_3)_3$), 51.4 (OCH_3), 43.5 (C**1**), 29.0 (C**2**), 28.3 ($OC(CH_3)_3$).

HRMS: (ESI^+) Calculated for $C_{17}H_{21}NNaO_4$: 326.1363. Found $[M+Na]^+$: 326.1365.

Methyl 2-(3-bromopyridin-2-yl)acetate

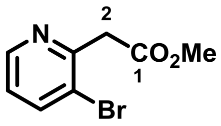

To a solution of LHDMS (75.0 mmol) in anhydrous THF (150 mL) was added 3-bromo-2-methylpyridine (2.88 mL, 25.0 mmol) dropwise. The reaction mixture was stirred for 2.5 hours before dropwise addition of dimethyl carbonate (3.37 mL, 40.0 mmol) and then stirred for a further 14 hours before being partially concentrated *in vacuo*. The reaction mixture was partitioned between EtOAc (100 mL) and water (50 mL), the phases were separated and the aqueous phase was extracted with EtOAc (2 × 30 mL). The organic phase was washed with brine (50 mL), dried over Na_2SO_4 and concentrated *in vacuo* to afford the title compound (5.63 g, 98 %) as a red oil which was used without further purification.

ν_{max} / cm^{-1}: (*film*) 2953 (m), 1736 (s), 1574 (m), 1429 (s), 1023 (s).

δ_H (400 MHz, $CDCl_3$) 8.49 (1H, dd, J = 4.5, 1.5 Hz, ArCH), 7.86 (1H, dd, J = 8.0, 1.5 Hz, ArCH), 7.10 (1H, dd, J = 8.0, 4.5 Hz, ArCH), 4.06 (2H, s, C**2**-H_2), 3.73 (3H, s, OCH_3).

δ_C (101 MHz, $CDCl_3$) 170.0 (C**1**), 153.6 (ArC), 147.9 (ArCH), 140.3 (ArCH), 123.6 (ArCH), 122.0 (ArC), 52.3 (OCH_3), 43.6 (C**2**).

HRMS: (ESI^+) Calculated for $C_8H_9BrNO_2$: 229.9811. Found $[M+H]^+$: 229.9811.

2-(3-Bromopyridin-2-yl)ethan-1-ol

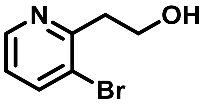

This compound was prepared according to a literature procedure.[37]

δ_H (500 MHz, $CDCl_3$) 8.45 (1H, dd, J = 5.0, 1.5 Hz), 7.86 (1H, dd, J = 8.0, 1.5 Hz), 7.07 (1H, ddt, J = 8.0, 5.0, 0.5 Hz), 4.08 (2H, t, J = 5.5 Hz), 3.16 (2H, td, J = 5.5, 0.5 Hz).

δ_C (126 MHz, $CDCl_3$) 159.0, 147.2, 140.3, 122.7, 121.8, 60.5, 38.1.

The spectroscopic properties were consistent with the data available in the literature.[37]

Methyl (*E*)-3-(2-(2-hydroxyethyl)pyridin-3-yl)acrylate

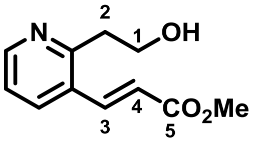

A solution of the preceding aryl bromide (1.21 g, 6.00 mmol), $Pd(OAc)_2$ (67.4 mg, 300 µmol), tri(*o*-tolyl)phosphine (183 mg, 600 µmol), methyl acrylate (0.81 mL, 9.00 mmol) and Et_3N (1.26 mL, 9.00 mmol) in anhydrous DMF (12 mL) was sparged with argon for five minutes. The reaction mixture was heated at 90 °C in a sealed tube for 21 hours before being diluted with brine (25 mL) and extracted with EtOAc (3 × 40 mL). The organic phase was dried over Na_2SO_4 and concentrated *in vacuo*. FCC (gradient elution: 1:0 – 9:1 EtOAc:MeOH) afforded the title compound (1.22 g, 98 %) as a red crystalline solid.

ν_{max} / cm^{-1}: (*solid*) 3163 (br s), 2855 (m), 1712 (s), 1637 (s), 1169 (s), 1053 (s).

δ_H (400 MHz, $CDCl_3$) 8.53 (1H, dd, J = 5.0, 2.0 Hz, ArCH), 7.92 (1H, d, J = 16.0 Hz, C**3**-H), 7.85 (1H, dd, J = 8.0, 2.0 Hz, ArCH), 7.25 (1H, dd, J = 8.0, 5.0 Hz, ArCH), 6.40 (1H, d, J = 16.0 Hz, C**4**-H), 4.11 (2H, t, J = 5.5 Hz, C**1**-H_2), 3.85 (3H, s, OCH_3), 3.17 (2H, t, J = 5.5 Hz, C**2**-H_2).

δ_C (101 MHz, $CDCl_3$) 166.6 (C**5**), 159.2 (ArC), 149.6 (ArCH), 140.0 (C**3**), 134.3 (ArCH), 129.2 (ArC), 121.9 (ArCH), 121.7 (C**4**), 60.8 (C**1**), 51.9 (OCH_3), 35.7 (C**2**).

HRMS: (ESI^+) Calculated for $C_{11}H_{13}NNaO_3$: 230.0788. Found $[M+Na]^+$: 230.0797.

Methyl (*E*)-3-(2-(2-((*tert*-butoxycarbonyl)((pentafluorobenzoyl)oxy)amino)ethyl)pyridin-3-yl) acrylate (2v)

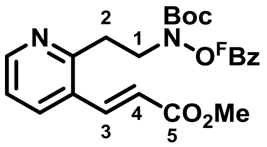

General procedure D: **T**he preceding alcohol (518 mg, 2.50 mmol) was employed with **1a**. The reaction time was 16 hours. FCC (gradient elution: 19:1 – 14:1 – 9:1 PhMe:EtOAc) afforded **2v** (837 mg, 65 %) as a pale yellow oil.

ν_{max} / cm^{-1}: (*film*) 2982 (m), 1782 (s), 1720 (s), 1651 (m), 1501 (s), 1154 (s).

δ_H (400 MHz, $CDCl_3$) 8.53 (1H, dd, *J* = 5.0, 2.0 Hz, ArC$\underline{H}$), 7.94 (1H, d, *J* = 16.0 Hz, C**3**-$\underline{H}$), 7.79 (1H, dd, *J* = 8.0, 2.0 Hz, ArC$\underline{H}$), 7.19 (1H, dd, *J* = 8.0, 5.0 Hz, ArC$\underline{H}$), 6.36 (1H, d, *J* = 16.0 Hz, C**4**-$\underline{H}$), 4.14 (2H, t, *J* = 7.5 Hz, C**1**-$\underline{H}_2$), 3.79 (3H, s, OC$\underline{H}_3$), 3.30 (2H, t, *J* = 7.5 Hz, C**2**-$\underline{H}_2$), 1.45 (9H, s, OC(C$\underline{H}_3$)$_3$).

δ_C (101 MHz, $CDCl_3$) 166.6 (C**5**), 157.1 (Ar$\underline{C}$), 154.2 (Boc $\underline{C}$=O), 150.5 (Ar$\underline{C}$H), 140.2 (C**3**), 134.3 (Ar$\underline{C}$H), 129.5 (Ar$\underline{C}$), 122.2 (Ar$\underline{C}$H), 122.0 (C**4**), 83.6 (O$\underline{C}$(CH_3)$_3$), 51.9 (O$\underline{C}H_3$), 50.5 (C**1**), 32.7 (C**2**), 28.1 (OC($\underline{C}H_3$)$_3$).

The signals corresponding to the pentafluorobenzoyl group could not be resolved due to their weak intensity.

δ_F (377 MHz, $CDCl_3$) -135.9 – -136.4 (2F, m), -146.7 (1F, tt, *J* = 21.0, 5.0 Hz), -159.5 – -159.7 (2F, m).

HRMS: (ESI^+) Calculated for $C_{23}H_{22}F_5N_2O_6$: 517.1393. Found $[M+H]^+$: 517.1410.

***tert*-Butyl (*E*)-5-(2-methoxy-2-oxoethylidene)-7,8-dihydro-1,6-naphthyridine-6(5*H*)-carboxylate (4v)**

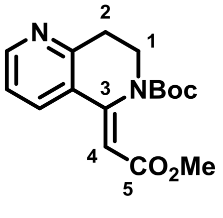

General procedure E: Conditions: 2.5 mol% $Pd_2(dba)_3$; 17.5 mol% PA-(4-(CO_2Et)C_6H_4) (**L-3**); 100 mol% Et_3N; dioxane (0.3 M); 130 °C. Substrate **2v** (54.2 mg, 0.105 mmol) was employed. FCC (*two times*, first eluent: 3:2 PhMe:EtOAc; second eluent: 14:1 PhMe:acetone) afforded **4v** (17.2 mg, 54 %) as a colorless crystalline solid.

*The structure of **4v** was confirmed by single-crystal X-ray diffraction after recrystallisation from CH_2Cl_2:petroleum ether.*

ν_{max} / cm^{-1}: (*film*) 2976 (m), 1720 (s), 1633 (s), 1150 (s).

δ_H (400 MHz, $CDCl_3$) 8.52 (1H, dd, *J* = 5.0, 1.5 Hz, ArCH), 7.96 (1H, dd, *J* = 8.0, 1.5 Hz, ArCH), 7.21 (1H, dd, *J* = 8.0, 5.0 Hz, ArCH), 6.30 (1H, s, C**4**-H), 4.19 – 3.80 (2H, br s, C**1**-H_2), 3.76 (3H, s, OCH_3), 3.09 (2H, t, *J* = 6.5 Hz, C**2**-H_2), 1.45 (9H, s, $OC(CH_3)_3$).

δ_C (101 MHz, $CDCl_3$) 165.8 (C**5**), 156.0 (ArC), 152.4 (Boc C=O), 150.4 (ArCH), 143.4 (ArC), 131.8 (ArCH), 126.8 (C**3**), 121.9 (ArCH), 107.7 (C**4**), 81.7 ($OC(CH_3)_3$), 51.4 (OCH_3), 42.7 (C**1**), 32.2 (C**2**), 28.1 ($OC(CH_3)_3$).

HRMS: (ESI$^+$) Calculated for $C_{16}H_{20}N_2NaO_4$: 327.1315. Found $[M+Na]^+$: 327.1311.

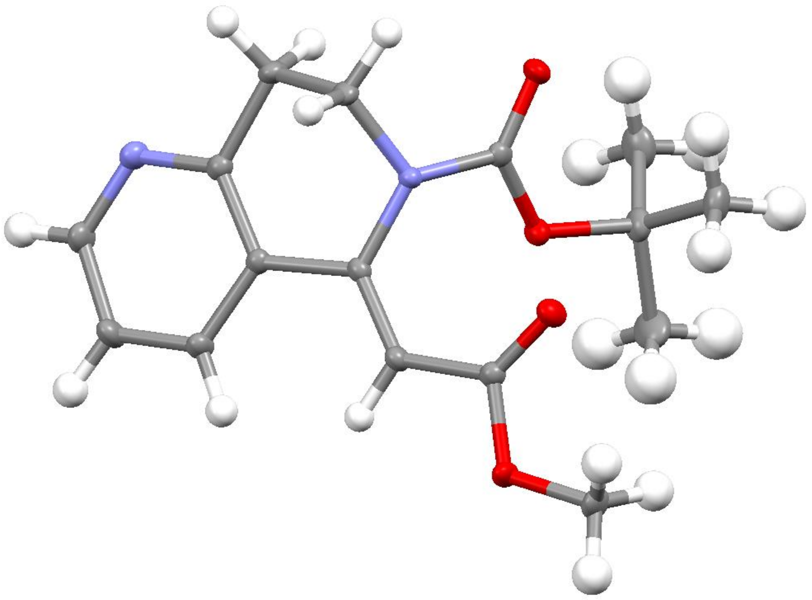

*ORTEP view of **4v***

Methyl 2-(3-bromopyridin-4-yl)acetate

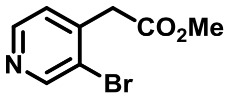

To a solution of LHDMS (75.0 mmol) in anhydrous THF (150 mL) was added 3-bromo-4-methylpyridine (2.88 mL, 25.0 mmol) dropwise. The reaction mixture was stirred for 1.5 hours before dropwise addition of dimethyl carbonate (3.37 mL, 40.0 mmol) and then stirred for a further 15 hours before being partially concentrated *in vacuo*. The reaction mixture was partitioned between EtOAc (100 mL) and water (50 mL), the phases were separated and the aqueous phase was extracted with EtOAc (2 × 40 mL). The organic phase was dried over Na_2SO_4 and concentrated *in vacuo* to afford the title compound (5.51 g, 96 %) as a red oil which was used without further purification.

δ_H (400 MHz, $CDCl_3$) 8.71 (1H, s), 8.47 (1H, d, *J* = 5.0 Hz), 7.25 (1H, d, *J* = 5.0 Hz), 3.79 (2H, s), 3.73 (3H, s).

δ_C (101 MHz, $CDCl_3$) 169.6, 152.2, 148.5, 142.8, 126.2, 123.6, 52.6, 40.8.

The spectroscopic properties were consistent with the data available in the literature.[38]

2-(3-Bromopyridin-4-yl)ethan-1-ol

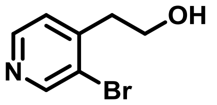

This compound was prepared according to a literature procedure.[37]

δ_H (400 MHz, $CDCl_3$) 8.65 (1H, s), 8.39 (1H, d, *J* = 5.0 Hz), 7.24 (1H, d, *J* = 5.0 Hz), 3.93 (2H, t, *J* = 6.5 Hz), 3.01 (2H, t, *J* = 6.5 Hz), 1.87 (1H, br s).

δ_C (101 MHz, $CDCl_3$) 152.0, 148.2, 147.3, 126.2, 123.5, 61.1, 38.6.

The spectroscopic properties were consistent with the data available in the literature.[37]

Methyl (*E*)-3-(4-(2-hydroxyethyl)pyridin-3-yl)acrylate

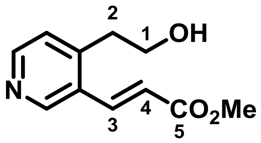

A solution of the preceding aryl bromide (1.21 g, 6.00 mmol), $Pd(OAc)_2$ (67.4 mg, 300 μmol), tri(*o*-tolyl)phosphine (183 mg, 600 μmol), methyl acrylate (0.81 mL, 9.00 mmol) and Et_3N (1.26 mL, 9.00 mmol) in anhydrous DMF (12 mL) was sparged with argon for five minutes. The reaction mixture was heated at 90 °C in a sealed tube for 17 hours before being diluted with brine (25 mL) and extracted with EtOAc (3 × 40 mL). The organic phase was dried over Na_2SO_4 and concentrated *in vacuo*. FCC (gradient elution: 1:0 – 19:1 EtOAc:MeOH) afforded the title compound (1.16 g, 93 %) as a yellow oil.

ν_{max} / cm^{-1}: (*film*) 3356 (br s), 2952 (m), 1717 (s), 1594 (m), 1436 (m), 1318 (s).

δ_H (400 MHz, $CDCl_3$) 8.72 (1H, s, ArCH), 8.48 (1H, d, *J* = 5.0 Hz, ArCH), 7.94 (1H, d, *J* = 16.0 Hz, C**3**-H), 7.21 (1H, d, *J* = 5.0 Hz, ArCH), 6.44 (1H, d, *J* = 16.0 Hz, C**4**-H), 3.89 (2H, t, *J* = 6.5 Hz, C**1**-H_2), 3.83 (3H, s, OCH_3), 3.01 (2H, t, *J* = 6.5 Hz, C**2**-H_2), 1.82 (1H, br s, OH).

δ_C (101 MHz, $CDCl_3$) 166.9 (C**5**), 150.2 (ArCH), 147.8 (ArCH), 147.2 (ArC), 139.3 (C**3**), 130.2 (ArC), 125.2 (ArCH), 121.3 (C**4**), 62.1 (C**1**), 52.1 (OCH_3), 35.9 (C**2**).

HRMS: (ESI^+) Calculated for $C_{11}H_{13}NNaO_3$: 230.0788. Found $[M+Na]^+$: 230.0792.

Methyl (*E*)-3-(4-(2-((tert-butoxycarbonyl)((pentafluorobenzoyl)oxy)amino)ethyl)pyridin-3-yl)acrylate (2w)

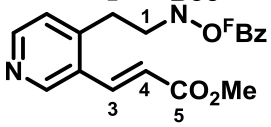

General procedure D: The preceding alcohol (518 mg, 2.50 mmol) was employed with **1a**. The reaction time was 16 hours. FCC (*two times*, first eluent: 39:1 PhMe:Et_3N; second, gradient elution: 5:1 – 5:2 PhMe:EtOAc) afforded **2w** (400 mg, 31 %) as a pale yellow oil.

ν_{max} / cm^{-1}: (*film*) 2983 (m), 1784 (s), 1720 (s), 1653 (m), 1505 (s), 1155 (s).

δ_H (400 MHz, $CDCl_3$) 8.74 (1H, s, ArCH), 8.51 (1H, d, *J* = 5.0 Hz, ArCH), 7.90 (1H, d, *J* = 16.0 Hz, C**3**-H), 7.21 (1H, d, *J* = 5.0 Hz, ArCH), 6.45 (1H, d, *J* = 16.0 Hz, C**4**-H), 3.92 (2H, t, *J* = 7.0 Hz, C**1**-H_2), 3.79 (3H, s, OCH_3), 3.10 (2H, t, *J* = 7.0 Hz, C**2**-H_2), 1.41 (9H, s, $OC(CH_3)_3$).

δ_C (101 MHz, $CDCl_3$) 166.6 (C**5**), 154.0 (Boc C=O), 150.7 (ArCH), 148.3 (ArCH), 145.7 (ArC), 138.6 (C**3**), 130.1 (ArC), 125.0 (ArCH), 122.0 (C**4**), 84.0 ($OC(CH_3)_3$), 52.0 (OCH_3), 50.8 (C**1**), 30.2 (C**2**), 28.0 ($OC(CH_3)_3$).

The signals corresponding to the pentafluorobenzoyl group could not be resolved due to their weak intensity.

δ_F (377 MHz, $CDCl_3$) -136.2 – -136.5 (2F, m), -146.1 (1F, tt, *J* = 21.0, 5.5 Hz), -159.2 – -159.4 (2F, m).

HRMS: (ESI^+) Calculated for $C_{23}H_{22}F_5N_2O_6$: 517.1393. Found $[M+H]^+$: 517.1391.

***tert*-Butyl (*E*)-1-(2-methoxy-2-oxoethylidene)-3,4-dihydro-2,7-naphthyridine-2(1*H*)-carboxylate (4w)**

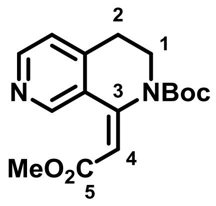

General procedure E: Conditions: 2.5 mol% $Pd_2(dba)_3$; 15 mol% PA-(4-(CF_3)C_6H_4) (**L-2**); 300 mol% Et_3N; THF (0.4 M); 130 °C, 10 hours. Substrate **2w** (54.2 mg, 0.105 mmol) was employed. FCC (*two times*, first, gradient elution: 10:1 – 8:1 PhMe:acetone; second eluent: 19:1 PhMe:MeOH) afforded **4w** (25.0 mg, 78 %, 6:1 mixture of *E* and *Z* alkene isomers) as a colorless oil.

The structure of ***4w*** *was confirmed by single-crystal X-ray diffraction after recrystallisation from* CH_2Cl_2*:petroleum ether.*

ν_{max} / cm^{-1}: (*film*) 2977 (m), 1707 (s), 1627 (s), 1321 (s), 1139 (s).

Spectroscopic data for the major E isomer:

δ_H (400 MHz, $CDCl_3$) 8.81 (1H, s, ArCH), 8.52 (1H, d, *J* = 5.0 Hz, ArCH), 7.13 (1H, d, *J* = 5.0 Hz, ArCH), 6.61 (1H, s, C**4**-H), 3.67 (3H, s, OCH_3), 3.62 (2H, t, *J* = 6.5 Hz, C**1**-H_2), 2.83 (2H, t, *J* = 6.5 Hz, C**2**-H_2), 1.52 (9H, s, $OC(CH_3)_3$).

δ_C (126 MHz, $CDCl_3$) 167.2 (C**5**), 153.1 (Boc <u>C</u>=O), 2 × 150.2 (2 × Ar<u>C</u>H), 145.4 (Ar<u>C</u>), 145.1 (C**3**), 128.7 (Ar<u>C</u>), 120.8 (Ar<u>C</u>H), 110.4 (C**4**), 82.4 (O<u>C</u>(CH_3)$_3$), 51.5 (O<u>C</u>H_3), 43.4 (C**1**), 28.5 (C**2**), 28.4 (OC(<u>C</u>H_3)$_3$).

Characteristic signals for the minor Z isomer:

δ_H (400 MHz, $CDCl_3$) 7.10 (1H, d, J = 5.0 Hz), 6.40 (1H, s), 3.75 (3H, s), 1.43 (9H, s).

HRMS: (ESI^+) Calculated for $C_{16}H_{21}N_2O_4$: 305.1496. Found $[M+H]^+$: 305.1504.

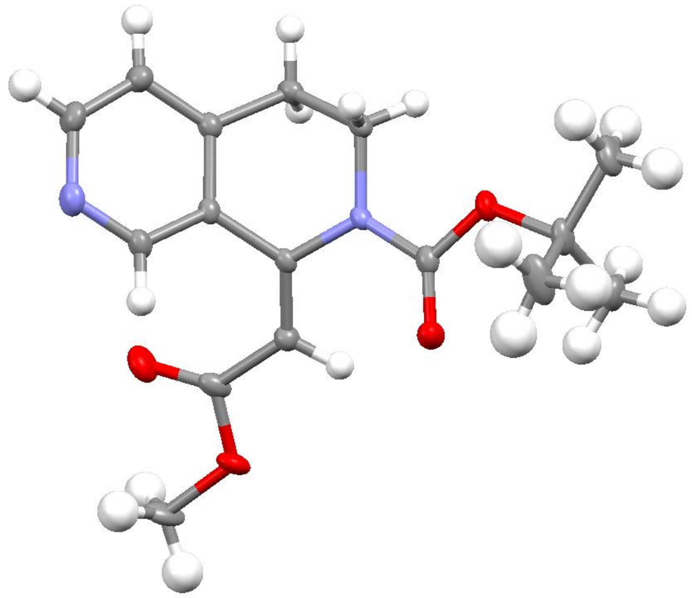

ORTEP view of ***4w***

3-(2-Bromophenyl)propan-1-ol

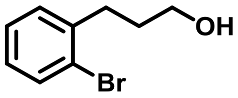

<u>General procedure C:</u> 3-(2-Bromophenyl)propionic acid (2.29 g, 10.0 mmol) was employed using anhydrous Et_2O as solvent and 1.05 eq. $LiAlH_4$ (1.0 M in Et_2O). The title compound (2.09 g, 97 %) was isolated as a colorless oil.

δ_H (400 MHz, $CDCl_3$) 7.54 (1H, d, J = 8.0 Hz), 7.26 – 7.21 (2H, m), 7.10 – 7.03 (1H, m), 3.71 (2H, t, J = 6.5 Hz), 2.84 (2H, t, J = 7.5 Hz), 1.95 – 1.86 (2H, m), 1.45 (1H, br s).

δ_C (101 MHz, $CDCl_3$) 141.2, 133.0, 130.6, 127.8, 127.6, 124.6, 62.3, 32.9, 32.5.

The spectroscopic properties were consistent with the data available in the literature.[39]

Methyl (*E*)-3-(2-(3-hydroxypropyl)phenyl)acrylate

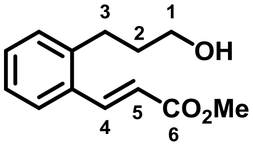

A solution of the preceding aryl bromide (1.29 g, 6.00 mmol), $Pd(OAc)_2$ (67.4 mg, 300 µmol), tri(*o*-tolyl)phosphine (183 mg, 600 µmol) and methyl acrylate (0.81 mL, 9.00 mmol) in anhydrous Et_3N (4.5 mL) was sparged with argon for five minutes. The reaction mixture was heated at 95 °C in a sealed tube for 20 hours before being filtered through celite, the filter cake was rinsed with EtOAc and the filtrate was concentrated *in vacuo*. FCC (eluent: 2:1 petroleum ether:EtOAc) afforded the title compound (1.23 g, 93 %) as a pale yellow oil.

ν_{max} / cm^{-1}: (*film*) 3417 (br s), 2948 (m), 1699 (s), 1630 (s), 1318 (s), 1172 (s).

δ_H (400 MHz, $CDCl_3$) 8.04 (1H, d, *J* = 16.0 Hz, C**4**-H), 7.56 (1H, dd, *J* = 8.0, 1.5 Hz, ArCH), 7.34 – 7.29 (1H, m, ArCH), 7.25 – 7.20 (2H, m, ArCH), 6.37 (1H, d, *J* = 16.0 Hz, C**5**-H), 3.81 (3H, s, OCH_3), 3.68 (2H, t, *J* = 6.5 Hz, C**1**-H_2), 2.87 (2H, t, *J* = 7.5 Hz, C**3**-H_2), 1.88 – 1.80 (2H, m, C**2**-H_2), 1.59 (1H, br s, OH).

δ_C (101 MHz, $CDCl_3$) 167.6 (C**6**), 142.5 (C**4**), 141.7 (ArC), 133.1 (ArC), 130.3 (ArCH), 130.2 (ArCH), 126.8 (ArCH), 126.7 (ArCH), 119.3 (C**5**), 62.1 (C**1**), 51.9 (OCH_3), 34.4 (C**2**), 29.5 (C**3**).

HRMS: (ESI^+) Calculated for $C_{13}H_{16}NaO_3$: 243.0992. Found $[M+Na]^+$: 243.1000.

Methyl (*E*)-3-(2-(3-((*tert*-butoxycarbonyl)((pentafluorobenzoyl)oxy)amino)propyl)phenyl)-acrylate (2x)

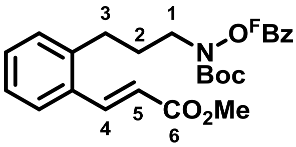

General procedure D: The preceding alcohol (551 mg, 2.50 mmol) was employed with **1a**. The reaction time was 15 hours. FCC (gradient elution: 1:0 – 49:1 PhMe:EtOAc) afforded **2x** (884 mg, 67 %) as a pale yellow oil.

ν_{max} / cm^{-1}: (*film*) 2952 (m), 1783 (s), 1718 (s), 1506 (s), 1154 (s).

δ_H (400 MHz, $CDCl_3$) 7.98 (1H, d, *J* = 16.0 Hz, C**4**-H), 7.55 (1H, d, *J* = 7.0 Hz, ArCH), 7.34 – 7.28 (1H, m, ArCH), 7.26 – 7.20 (2H, m, ArCH), 6.36 (1H, d, *J* = 16.0 Hz, C**5**-H), 3.78 (3H, s, OCH_3), 3.73 (2H, t, *J* = 7.0 Hz, C**1**-H_2), 2.86 (2H, t, *J* = 7.5 Hz, C**3**-H_2), 1.91 (2H, tt, *J* = 7.5, 7.0 Hz, C**2**-H_2), 1.49 (9H, s, $OC(CH_3)_3$).

δ_C (101 MHz, $CDCl_3$) 167.4 (C**6**), 157.5 (^{F}Bz <u>C</u>=O), 154.7 (Boc <u>C</u>=O), 142.1 (C**5**), 140.9 (Ar<u>C</u>), 133.1 (Ar<u>C</u>), 130.3 (Ar<u>C</u>H), 130.2 (Ar<u>C</u>H), 2 × 126.9 (2 × Ar<u>C</u>H), 119.6 (C**4**), 83.6 (O<u>C</u>(CH_3)$_3$), 51.8 (O<u>C</u>H_3), 50.4 (C**1**), 30.2 (C**3**), 28.6 (C**2**), 28.1 (OC(<u>C</u>H_3)$_3$).

The aromatic signals corresponding to the pentafluorobenzoyl group could not be resolved due to their weak intensity.

δ_F (377 MHz, $CDCl_3$) -136.4 – -136.7 (2F, m), -146.7 (1F, tt, *J* = 21.0, 5.0 Hz), -159.4 – -159.6 (2F, m).

HRMS: (ESI$^+$) Calculated for $C_{25}H_{24}F_5NNaO_6$: 552.1416. Found [M+Na]$^+$: 552.1411.

***tert*-Butyl (*E*)-1-(2-methoxy-2-oxoethylidene)-1,3,4,5-tetrahydro-2*H*-benzo[*c*]azepine-2-carboxylate (4x)**

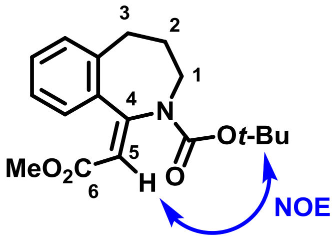

<u>**General procedure E:**</u> Conditions: 2.5 mol% $Pd_2(dba)_3$; 17.5 mol% PA-(4-(CO_2Et)C_6H_4) (**L-3**); 100 mol % Et_3N; dioxane (0.3 M); 130 °C. Substrate **2x** (55.6 mg, 0.105 mmol) was employed. FCC (eluent: 49:1 PhMe:EtOAc) afforded **4x** (9.7 mg, 29 %) as a colorless oil.

*The product was assigned as the E isomer based on the observed NOE correlation between the C***5** *and the t-Bu protons.*

ν_{max} / cm^{-1}: (*film*) 2979 (m), 2931 (m), 1717 (s), 1168 (s).

δ_H (400 MHz, $CDCl_3$) 7.38 – 7.10 (4H, m, ArC<u>H</u>), 6.55 (1H, s, C**5**-<u>H</u>), 3.54 (3H, s, OC<u>H</u>$_3$), 3.50 (2H, t, *J* = 6.0 Hz, C**1**-<u>H</u>$_2$), 2.78 (2H, t, *J* = 6.5 Hz, C**3**-<u>H</u>$_2$), 1.88 (2H, tt, *J* = 6.5, 6.0 Hz, C**2**-<u>H</u>$_2$), 1.46 (9H, s, OC(C<u>H</u>$_3$)$_3$).

δ_C (101 MHz, $CDCl_3$) 167.8 (C**6**), 154.9 (C**4**), 153.5 (Boc <u>C</u>=O), 138.3 (Ar<u>C</u>), 134.6 (Ar<u>C</u>), 131.4 (Ar<u>C</u>H), 130.2 (Ar<u>C</u>H), 128.6 (Ar<u>C</u>H), 125.8 (Ar<u>C</u>H), 109.5 (C**5**), 81.8 (O<u>C</u>(CH_3)$_3$), 51.2 (O<u>C</u>H_3), 46.7 (C**1**), 31.2 (C**3**), 28.3 (OC(<u>C</u>H_3)$_3$), 28.1 (C**2**).

HRMS: (ESI$^+$) Calculated for $C_{18}H_{23}NNaO_4$: 340.1519. Found [M+Na]$^+$: 340.1531.

Mechanistic investigations

(*E*)-1-Iodonon-4-ene

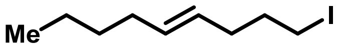

To a solution of PPh_3 (3.15 g, 12.0 mmol) and imidazole (817 mg, 12.0 mmol) in CH_2Cl_2 (15 mL) was added iodine (3.05 g, 12.0 mmol) gradually over a period of around 5 minutes. The reaction mixture was stirred for 30 minutes before addition of (*E*)-non-4-en-1-ol (*vide supra*, 1.42 g, 10.0 mmol). The reaction mixture was stirred at room temperature for 13 hours before addition of hexane (25 mL) and filtration. The filtrate was concentrated *in vacuo*. FCC (eluent: pentane) afforded the title compound (2.40 g, 95 %) as a colorless oil.

ν_{max} / cm^{-1}: (*film*) 2955 (m), 2924 (m), 1438 (m), 1219 (m).

δ_H (400 MHz, $CDCl_3$) 5.51 (1H, dtt, *J* = 15.0, 7.0, 1.5 Hz), 5.35 (1H, dtt, *J* = 15.0, 7.0, 1.5 Hz), 3.21 (2H, t, *J* = 7.0 Hz), 2.12 (2H, dtd, *J* = 7.0, 7.0, 1.5 Hz), 2.01 (2H, dtd, *J* = 7.0, 7.0, 1.5 Hz), 1.90 (2H, tt, *J* = 7.0 Hz), 1.40 – 1.28 (4H, m), 0.94 – 0.88 (3H, m).

δ_C (101 MHz, $CDCl_3$) 132.4, 127.8, 2 × 33.3, 32.4, 31.8, 22.3, 14.1, 6.8.

The spectroscopic properties were consistent with the data available in the literature.[40,41]

(*E*)-2-(Non-4-en-1-yl)isoindoline-1,3-dione

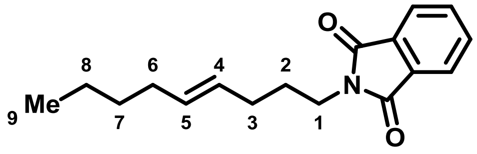

A solution of the preceding iodide (504 mg, 2.00 mmol) and potassium pthalimide (370 mg, 2.00 mmol) in anhydrous DMF (8 mL) was heated at 60 °C for 16 hours. The reaction mixture was cooled to room temperature, diluted with Et_2O (70 mL) and washed with brine (4 × 40 mL). The Et_2O phase was dried over Na_2SO_4 and concentrated *in vacuo* to afford the title compound (523 mg, 96 %) as a pale yellow oil.

ν_{max} / cm^{-1}: (*film*) 2927 (m), 1707 (s), 1394 (s).

δ_H (400 MHz, $CDCl_3$) 7.83 (2H, dd, *J* = 5.5, 3.0 Hz, ArCH), 7.70 (2H, dd, *J* = 5.5, 3.0 Hz, ArCH), 5.48 – 5.33 (2H, m, C**4**-H and C**5**-H), 3.68 (2H, t, *J* = 7.5 Hz, C**1**-H_2), 2.05 (2H, dt, *J* = 7.0, 7.0 Hz, C**3**-H_2), 1.97 – 1.90 (2H, m, C**6**-H_2), 1.74 (2H, tt, *J* = 7.5, 7.0 Hz, C**2**-H_2), 1.33 – 1.24 (4H, m, C**7**-H_2 and C**8**-H_2), 0.90 – 0.83 (3H, m, C**9**-H_3).

δ_C (101 MHz, $CDCl_3$) 168.4 (C=O), 133.8 (ArCH), 132.2 (ArC), 131.5 (C**5**), 128.5 (C**4**), 123.1 (ArCH), 37.7 (C**1**), 32.2 (C**6**), 31.6 (C**7**), 29.9 (C**3**), 28.3 (C**2**), 22.2 (C**8**), 13.9 (C**9**).

HRMS: (ESI^+) Calculated for $C_{17}H_{21}NNaO_2$: 294.1464. Found $[M+Na]^+$: 294.1469.

***tert*-Butyl (*E*)-non-4-en-1-ylcarbamate (5)**

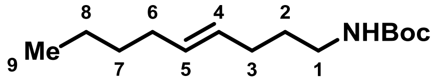

A solution of the preceding pthalimide (505 mg, 1.86 mmol) and hydrazine (55 % aqueous solution, 0.31 mL, 5.58 mmol) in EtOH (10 mL) was heated at reflux for 2.5 hours. The reaction mixture was cooled to room temperature and filtered. The filtrate was concentrated *in vacuo* and redissolved in CH_2Cl_2 (15 mL) before addition of Boc_2O (0.52 mL, 2.23 mmol) and Et_3N (0.39 mL, 2.79 mmol). The reaction mixture was stirred at room temperature for 16 hours before being concentrated *in vacuo*. FCC (eluent: 9:1 petroleum ether:EtOAc) afforded **5** (332 mg, 74 %) as a colorless oil.

ν_{max} / cm^{-1}: (*film*) 3348 (br s), 2927 (m), 1689 (s), 1514 (s), 1169 (s).

δ_H (400 MHz, $CDCl_3$) 5.46 – 5.29 (2H, m, C**4**-H and C**5**-H), 4.54 (1H, br s, NH), 3.09 (2H, td, *J* = 7.0, 6.5 Hz, C**1**-H_2), 2.04 – 1.91 (4H, m, C**3**-H_2 and C**6**-H_2), 1.52 (2H, tt, *J* = 7.5, 7.0 Hz, C**2**-H_2), 1.42 (9H, s, $OC(CH_3)_3$), 1.34 – 1.25 (4H, m, C**7**-H_2 and C**8**-H_2), 0.89 – 0.83 (3H, m, C**9**-H_3).

δ_C (101 MHz, $CDCl_3$) 156.1 (C=O), 131.5 (C**5**), 129.1 (C**4**), 79.1 ($OC(CH_3)_3$), 40.3 (C**1**), 32.4 (C**6**), 31.8 (C**7**), 2 × 30.0 (C**2** and C**3**), 28.6 ($OC(CH_3)_3$), 22.3 (C**8**), 14.1 (C**9**).

HRMS: (ESI^+) Calculated for $C_{14}H_{27}NNaO_2$: 264.1934. Found $[M+Na]^+$: 264.1942.

***tert*-Butyl (3a*R**,7a*S**)-7a-vinyloctahydro-1*H*-indole-1-carboxylate**

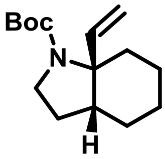

In the following experiment a mixture of aza-Heck and aza-Wacker substrates were submitted to the reaction conditions. The fact that only the aza-Heck substrate afforded cyclized product is consistent with the mechanism we propose.

General procedure E: Conditions: 2.5 mol% $Pd_2(dba)_3$; 15 mol% PA-Ph (**L-1**); 100 mol% Et_3N; THF (0.4 M); 130 °C. Substrates **2k** (48.7 mg, 0.105 mmol) and **5** (16.9 mg, 70.0 μmol) were employed. From the ^1H NMR spectrum the yield of **4k** was determined to be 79 % and no aza-Wacker product (**4a**) was observed.

1,1,1-Trifluoro-2-methylpropan-2-yl (*Z*)-(4-benzylhex-4-en-1-yl)((pentafluorobenzoyl)oxy)carbamate (CF_3-2i)

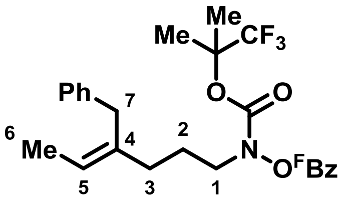

General procedure D: (*Z*)-4-Benzylhex-en-1-ol (*vide supra*, 381 mg, 2.00 mmol) was employed with **CF_3-1a**. The reaction time was 15 hours. FCC (gradient elution: 3:2 – 3:7 hexane:PhMe) afforded **CF_3-2i** (587 mg, 53 %) as a colorless oil.

ν_{max} / cm^{-1}: (*film*) 2947 (m), 1786 (s), 1731 (s), 1652 (m), 1497 (s), 1166 (s).

δ_H (500 MHz, $CDCl_3$) 7.27 – 7.22 (2H, m, ArC$\underline{H}$), 7.19 – 7.12 (3H, m, ArC$\underline{H}$), 5.45 (1H, q, *J* = 7.0 Hz, C**5**-$\underline{H}$), 3.62 (2H, t, *J* = 7.0 Hz, C**1**-$\underline{H}_2$), 3.39 (2H, s, C**7**-$\underline{H}_2$), 1.99 (2H, t, *J* = 7.5 Hz, C**3**-$\underline{H}_2$), 1.75 – 1.68 (11H, m, C**2**-$\underline{H}_2$, C**6**-$\underline{H}_3$ and OC(CF_3)(C$\underline{H}_3$)$_2$).

δ_C (126 MHz, $CDCl_3$) 152.1 (Carbamate $\underline{C}$=O), 139.7 (Ar$\underline{C}$), 136.7 (C**4**), 2 × 128.1 (2 × Ar$\underline{C}$H), 125.6 (Ar$\underline{C}$H), 121.0 (C**5**), 81.6 (q, *J* = 30.0 Hz, O$\underline{C}$(CF_3)(CH_3)$_2$), 50.3 (C**1**), 35.2 (C**7**), 33.0 (C**3**), 24.8 (C**2**), 19.0 (OC(CF_3)($\underline{C}H_3$)$_2$), 13.4 (C**6**).

The signals corresponding to the pentafluorobenzoyl or trifluoromethyl groups could not be resolved due to their weak intensity.

δ_F (470 MHz, $CDCl_3$) -84.0 (3F, s), -135.6 – -135.8 (2F, m), -145.6 (1F, tt, *J* = 21.0, 5.5 Hz), -159.2 – -159.4 (2F, m).

HRMS: (ESI^+) Calculated for $C_{25}H_{23}F_8NNaO_4$: 576.1392. Found $[M+Na]^+$: 576.1373.

1,1,1-Trifluoro-2-methylpropan-2-yl 2-benzyl-2-vinylpyrrolidine-1-carboxylate (CF_3-4i)

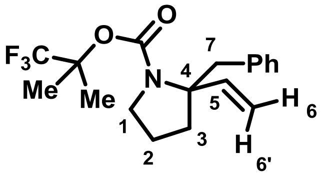

The following reaction was conducted in an NMR spectrometer (without a magnetic stirrer) and the reaction progress was monitored by collecting ^{19}F and solvent-supressed ^1H spectra.

General procedure E: Conditions: 2.5 mol% $Pd_2(dba)_3$; 15 mol% PA-(4-(CF_3)C_6H_4) (**L-2**); 100 mol% Et_3N; THF (0.2 M); 130 °C. Substrate **CF_3-2i** (58.1 mg, 0.105 mmol) was employed. FCC (*two times*, first eluent: PhMe; second eluent: 59:1 petroleum ether:acetone) afforded **CF_3-4i** (21.6 mg, 60 %) as a colorless oil.

ν_{max} / cm^{-1}: (*film*) 2978 (m), 1701 (s), 1382 (s), 1132 (s).

This compound exists as an approximately 3:2 mixture of rotamers A and B.

δ_H (400 MHz, $CDCl_3$) 7.31 – 7.19 (3H, m, A and B: ArCH), 7.19 – 7.11 (2H, m, A and B: ArCH), 6.07 (0.6H, dd, *J* = 17.5, 10.5 Hz, A: C**5**-H), 5.99 (0.4H, dd, *J* = 17.5, 10.5 Hz, B: C**5**-H), 5.12 (0.6H, d, *J* = 10.5 Hz, A: C**6**-H), 5.08 (0.4H, d, *J* = 10.5 Hz, B: C**6**-H), 5.01 (0.6H, d, *J* = 17.5 Hz, A: C**6**-H'), 4.98 (0.4H, d, *J* = 17.5 Hz, B: C**6**-H'), 3.59 (0.6H, d, *J* = 13.5 Hz, A: C**7**-H), 3.53 (0.4H, ddd, *J* = 12.0, 7.5, 5.0 Hz, B: C**1**-H), 3.44 (0.6H, ddd, *J* = 11.5, 8.0, 4.0 Hz, A: C**1**-H), 3.38 (0.4H, d, *J* = 13.5 Hz, B: C**7**-H), 3.10 (0.4H, ddd, *J* = 11.0, 7.5, 7.5 Hz, B: C**1**-H'), 3.01 (0.6H, ddd, *J* = 11.0, 8.0, 7.0 Hz, A: C**1**-H'), 2.98 – 2.90 (1H, m, A and B: C**7**-H'), 2.09 – 1.95 (1H, m, A and B: C**3**-H), 1.84 – 1.70 (7H, m, A and B: C**3**-H', $OC(CF_3)(CH_3)(CH_3)$' and $OC(CF_3)(CH_3)(CH_3)$'), 1.64 – 1.51 (1H, m, A and B: C**2**-H), 1.34 – 1.16 (1H, m, A and B: C**2**-H').

δ_C (101 MHz, $CDCl_3$) 152.5 (B: C=O), 151.6 (A: C=O), 142.1 (B: C**5**), 141.5 (A: C**5**), 137.6 (B: ArC), 137.5 (A: ArC), 130.7 (A: ArCH), 130.5 (B: ArCH), 128.2 (B: ArCH), 128.0 (A: ArCH), 126.5 (B: ArCH), 126.4 (A: ArCH), 112.3 (A: C**6**), 112.2 (B: C**6**), 79.8 (q, *J* = 30.0 Hz, B: $OC(CF_3)(CH_3)_2$), 79.3 (q, *J* = 29.0 Hz, A: $OC(CF_3)(CH_3)_2$), 67.7 (A: C**4**), 67.3 (B: C**4**), 49.1 (B: C**1**), 48.8 (A: C**1**), 42.1 (B: C**7**), 41.2 (A: C**7**), 37.7 (B: C**3**), 36.1 (A: C**3**), 21.3 (A: C**2**), 20.9 (B: C**2**), 20.0 (q, *J* = 1.5 Hz, B: $OC(CF_3)(CH_3)(CH_3)$'), 2 × 19.8 (q, *J* = 1.5 Hz, A: $OC(CF_3)(CH_3)(CH_3)$' and q, *J* = 1.5 Hz, A: $OC(CF_3)(CH_3)(CH_3)$'), 19.7 (q, *J* = 1.5 Hz, B: $OC(CF_3)(CH_3)(CH_3)$').

The signal corresponding to the trifluoromethyl group could not be resolved due to its weak intensity.

δ_F (377 MHz, $CDCl_3$) -82.8 (1.2F, s, B: CF_3), -84.0 (1.8F, s, B: CF_3).

HRMS: (ESI^+) Calculated for $C_{18}H_{23}F_3NO_2$: 342.1675. Found $[M+H]^+$: 342.1691.

Cascade reactions

2-Methylenehex-5-en-1-ol

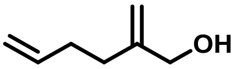

This compound was prepared according to a literature procedure.[42]

ν_{max} / cm^{-1}: (film) 3319 (br s), 3078 (m), 2921 (s), 1641 (s), 1449 (s), 1022 (s).

δ_H (400 MHz, $CDCl_3$) 5.81 (1H, ddt, *J* = 17.0, 10.0, 6.5 Hz), 5.03 (1H, br s) 5.02 (1H, ddt, *J* = 17.0, 2.0, 1.5 Hz), 4.96 (1H, ddt, *J* = 10.0, 2.0, 1.0 Hz), 4.87 (1H, br s), 4.05 (2H, s), 2.25 – 2.18 (2H, m), 2.17 – 2.11 (2H, m), 1.94 (1H, br s).

δ_C (101 MHz, $CDCl_3$) 148.3, 138.1, 114.8, 109.6, 65.8, 32.2, 31.9.

The spectroscopic properties were consistent with the data available in the literature.[42,43]

Ethyl 4-methyleneoct-7-enoate

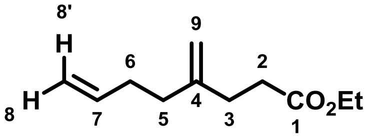

General procedure B: The preceding allylic alcohol (2.24 g, 20.0 mmol) was employed. The reaction time was 16 hours. FCC (eluent: 29:1 hexane:EtOAc) afforded the title compound (2.26 g, 62 %) as a colorless oil.

ν_{max} / cm^{-1}: (film) 3079 (m), 2981 (s), 2934 (s), 1785 (s), 1643 (s), 1445 (s), 1371 (s), 1154 (s).

δ_H (400 MHz, $CDCl_3$) 5.80 (1H, ddt, *J* = 17.0, 10.5, 6.5 Hz, C**7**-H), 5.02 (1H, ddt, *J* = 17.0, 2.0, 1.5 Hz, C**8**-H’), 4.95 (1H, ddt, *J* = 10.5, 2.0, 1.0 Hz, C**8**-H), 4.76 (1H, br s, C**9**-H), 4.74 (1H, br s, C**9**-H’), 4.12 (2H, q, *J* = 7.0 Hz, $OC\underline{H_2}CH_3$), 2.47 – 2.42 (2H, m, C**2**-$\underline{H_2}$), 2.36 – 2.30 (2H, m, C**3**-$\underline{H_2}$), 2.23 – 2.16 (2H, m, C**6**-$\underline{H_2}$), 2.14 – 2.08 (2H, m, C**5**-$\underline{H_2}$), 1.25 (3H, t, *J* = 7.0 Hz, $OCH_2C\underline{H_3}$).

δ_C (101 MHz, $CDCl_3$) 173.4 (C**1**), 147.5 (C**4**), 138.3 (C**7**), 114.8 (C**8**), 109.7 (C**9**), 60.5 ($O\underline{C}H_2CH_3$), 35.7 (C**5**), 32.9 (C**2**), 32.1 (C**6**), 31.1 (C**3**), 14.4 ($OCH_2\underline{C}H_3$).

HRMS: (ESI^+) Calculated for $C_{11}H_{18}NaO_2$: 205.1199. Found $[M+Na]^+$: 205.1190.

4-Methyleneoct-7-en-1-ol

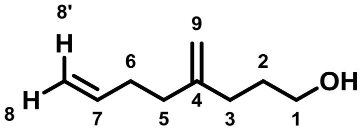

General procedure C: The preceding ester (1.00 g, 5.49 mmol) was employed using anhydrous Et_2O as the solvent and 0.8 eq. $LiAlH_4$ (1.0 M in Et_2O). The title compound (765 mg, 99 %) was isolated as a colorless oil.

ν_{max} / cm^{-1}: (film) 3323 (br s), 3078 (m), 2932 (s), 1642 (s), 1442 (s), 1057 (s).

δ_H (400 MHz, $CDCl_3$) 5.81 (1H, ddt, J = 17.0, 10.0, 6.5 Hz, C**7**-H), 5.01 (1H, ddt, J = 17.0, 1.5, 1.5 Hz, C**8**-H'), 4.97 – 4.92 (1H, m, C**8**-H), 4.76 (1H, br s, C**9**-H), 4.75 (1H, br s, C**9**-H'), 3.64 (2H, t, J = 6.5 Hz, C**1**-H_2), 2.23 – 2.15 (2H, m, C**6**-H_2), 2.14 – 2.05 (4H, m, C**3**-H_2 and C**5**-H_2), 1.77 (1H, br s, OH), 1.70 (2H, tt, J = 7.5, 6.5 Hz, C**2**-H_2).

δ_C (101 MHz, $CDCl_3$) 148.7 (C**4**), 138.5 (C**7**), 114.7 (C**8**), 109.5 (C**9**), 62.7 (C**1**), 35.4 (C**5**), 32.5 (C**3**), 32.1 (C**6**), 30.7 (C**2**).

HRMS: (EI^+) Calculated for C_9H_{14}: 122.1096. Found $[M-H_2O]^+$: 122.1091.

***tert*-Butyl (4-methyleneoct-7-en-1-yl)((pentafluorobenzoyl)oxy)carbamate (2y)**

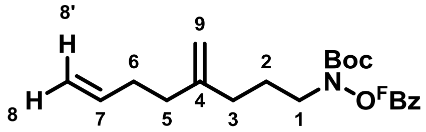

General procedure D: The preceding alcohol (280 mg, 2.00 mmol) was employed with **1a**. FCC (eluent: 1:4 hexane:PhMe) afforded **2y** (399 mg, 44 %) as a colorless oil.

ν_{max} / cm^{-1}: 3676, 2987, 2926, 1783, 1723, 1650, 1523, 1506, 1369, 1326, 1252, 1183, 1077.

δ_H (400 MHz, $CDCl_3$) 5.81 (1H, ddt, J = 17.0, 10.0, 6.5 Hz, C**7**-H), 5.02 (1H, ddt, J = 17.0, 2.0, 1.0 Hz, C**8**-H'), 4.95 (1H, ddt, J = 10.0, 2.0, 1.0 Hz, C**8**-H), 4.77 (2H, m, C**9**-H_2), 3.68 (2H, t, J = 7.5 Hz, C**1**-H_2), 2.26 – 2.15 (2H, m, C**3**-H_2), 2.12 – 2.09 (4H, m, C**5**-H_2 and C**6**-H_2), 1.79 (2H, tt, J = 7.5, 7.5 Hz, C**2**-H_2), 1.49 (9H, s, $OC(CH_3)_3$).

δ_C (101 MHz, $CDCl_3$) 154.6 (Boc C=O), 147.7 (C**4**), 138.3 (C**7**), 114.6 (C**8**), 109.9 (C**9**), 83.3 ($OC(CH_3)_3$), 50.6 (C**1**), 35.3 (C**5**), 32.8 (C**3**), 31.9 (C**6**), 28.0 ($OC(CH_3)_3$), 24.9 (C**2**).

The signals corresponding to the pentafluorobenzoyl group could not be resolved due to their weak intensity.

δ_F (377 MHz, $CDCl_3$) -136.6 (2F, d, J = 20.5 Hz), -146.6 (1F, tt, J = 20.5, 5.0 Hz), -159.3 – -159.5 (2F, m).

HRMS: (ESI^+) Calculated for $C_{21}H_{24}F_5NNaO_4$: 472.1518. Found $[M+Na]^+$: 472.1511.

***tert*-Butyl 7-methylene-1-azaspiro[4.4]nonane-1-carboxylate (4y)**

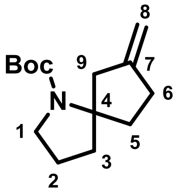

General procedure E: Conditions: 5.0 mol% $Pd_2(dba)_3$; 20 mol% PA-Ph (**L-1**); 100 mol% Et_3N; THF (0.4 M); 130 °C. Substrate **2y** (44.9 mg, 0.100 mmol) was employed. FCC (eluent: 19:1 PhMe:EtOAc) afforded **4y** (16.1 mg, 68 %) as a colorless oil.

ν_{max} / cm^{-1}: 2968, 2928, 2872, 1687, 1453, 1382, 1364, 1243, 1169, 1131, 1112.

^{1}H spectrum acquired at high temperature:

δ_H (500 MHz, CD_3CN, 65 °C) 4.86 – 4.82 (2H, m, C**8**-$\underline{H}_2$), 3.43 – 3.38 (2H, m, C**1**-$\underline{H}_2$), 3.21 (1H, br d, J = 15.0 Hz, C**9**-$\underline{H}$), 2.63 – 2.56 (1H, m, C**6**-$\underline{H}$), 2.52 – 2.47 (1H, m, C**5**-$\underline{H}$), 2.33 – 2.26 (1H, m, C**5**-$\underline{H}$'), 2.12 – 2.08 (1H, m, C**9**-$\underline{H}$'), 1.86 – 1.83 (2H, m, C**3**-$\underline{H}_2$), 1.79 – 1.74 (2H, m, C**2**-$\underline{H}_2$), 1.56 – 1.46 (1H, m, C**6**-$\underline{H}$'), 1.46 (9H, s, OC(C$\underline{H}_3$)$_3$).

At room temperature this compound exists as an approximately 7:3 mixture of rotamers A and B.

δ_C (126 MHz, CD_3CN) 154.6 (B: $\underline{C}$=O), 154.1 (A: $\underline{C}$=O), 151.6 (A and B: C**7**), 106.4 (A and B: C**8**), 79.8 (B: O$\underline{C}$(CH_3)$_3$), 79.2 (A: O$\underline{C}$(CH_3)$_3$), 69.8 (A: C**4**), 69.3 (B: C**4**), 49.1 (A and B: C**1**), 42.5 (A: C**9**), 42.3 (B: C**9**), 40.8 (A and B: C**3**), 35.2 (A and B: C**6**), 31.6 (B: C**5**), 31.5 (A: C**5**), 28.8 (A and B: OC($\underline{C}H_3$)$_3$), 22.9 (A: C**2**), 22.5 (B: C**2**).

HRMS: (ESI^+) Calculated for $C_{14}H_{23}NNaO_2$: 260.1621. Found $[M+Na]^+$: 260.1614.

***tert*-Butyl (4-methylpent-4-en-1-yl)((pentafluorobenzoyl)oxy)carbamate (6a)**

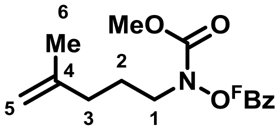

General procedure D: 4-Methylpent-4-en-1-ol (400 mg, 4.00 mmol) was employed with **1c**. FCC (eluent: 1:4 hexane:PhMe) afforded **6a** (734 mg, 50 %) as a colorless oil.

ν_{max} / cm^{-1}: 3077, 2944, 1785, 1730, 1651, 1524, 1503, 1448, 1326, 1174, 1078, 1000.

δ_H (400 MHz, $CDCl_3$) 4.75 – 4.74 (1H, m, C**5**-$\underline{H}$), 4.70 – 4.69 (1H, m, C**5**-$\underline{H}$'), 3.81 (3H, s, OC$\underline{H}_3$), 3.72 (2H, t, J = 7.0 Hz, C**1**-$\underline{H}_2$), 2.10 (2H, t, J = 7.5 Hz, C**3**-$\underline{H}_2$), 1.86 – 1.75 (2H, m, C**2**-$\underline{H}_2$), 1.72 (3H, s, C**6**-$\underline{H}_3$).

δ_C (101 MHz, $CDCl_3$) 156.0 (MeO–$\underline{C}$=O), 144.3 (C**4**), 110.7 (C**5**), 53.9 (O$\underline{C}H_3$), 50.9 (C**1**), 34.4 (C**3**), 24.7 (C**2**), 22.2 (C**6**).

The signals corresponding to the pentafluorobenzoyl group could not be resolved due to their weak intensity.

δ_F (377 MHz, $CDCl_3$) -136.2 (2F, dq, *J* = 18.0, 6.5 Hz), -146.1 (1F, tt, *J* = 21.0, 5.5 Hz), -159.2 – -159.5 (2F, m).

HRMS: (ESI^+) Calculated for $C_{15}H_{14}F_5NNaO_4$: 390.0741. Found $[M+Na]^+$: 390.0740.

Methyl 2-methyl-2-((1-methyl-1*H*-indol-2-yl)methyl)pyrrolidine-1-carboxylate (7aa)

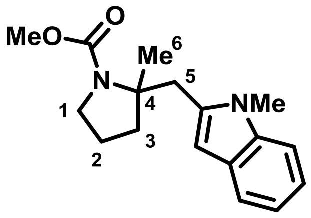

General procedure H: Conditions: 5.0 mol% $Pd_2(dba)_3$; 20 mol% PA-Ph (**L-1**); 25 mol% Et_3N; 200 mol% 1-methyl-2-indoleboronic acid pinacol ester; PhMe (0.4 M); 130 °C. Substrate **6a** (36.7 mg, 0.100 mmol) was employed. FCC (eluent: 19:1 PhMe:EtOAc) afforded **7aa** (20.9 mg, 73 %) as a pale yellow oil.

ν_{max} / cm^{-1}: 2948, 2922, 2874, 1695, 1445, 1375, 1339, 1317, 1216, 1191, 1129.

This compound exists as an approximately 7:3 mixture of rotamers A and B.

δ_H (400 MHz, $CDCl_3$) 7.55 (1H, d, *J* = 8.0 Hz, A and B: ArCH), 7.30 – 7.26 (1H, m, A and B: ArCH), 7.19 – 7.15 (1H, m, A and B: ArCH), 7.10 – 7.06 (1H, m, A and B: ArCH), 6.28 – 6.26 (1H, m, A and B: ArCH), 3.80 (0.9H, s, B: OCH_3), 3.73 (4.2H, s, A: OCH_3 and NCH_3), 3.69 (0.9H, s, B: NCH_3), 3.56 – 3.50 (0.3H, m, B: C**1**-H), 3.47 – 3.28 (2.7H, m, A: C**1**-H_2 and C**5**-H, B: C**1**-H’ and C**5**-H), 3.36 (0.7H, d, *J* = 15.0 Hz, A: C**5**-H’), 3.13 (0.3H, d, *J* = 15.0 Hz, B: C**5**-H’), 2.18 – 2.08 (1H, m, A and B: C**3**-H), 1.74 – 1.64 (2H, m, A and B: C**2**-H and C**3**-H’), 1.52 – 1.41 (4H, m, A and B: C**2**-H’ and C**6**-H_3).

δ_C (101 MHz, $CDCl_3$) 154.7 (A and B: C=O), 137.8 (A and B: ArC), 137.3 (A and B: ArC), 128.2 (B: ArC), 127.9 (A: ArC), 120.8 (B: ArCH), 120.7 (A: ArCH), 119.9 (B: ArCH), 119.7 (A: ArCH), 119.4 (B: ArCH), 119.3 (A: ArCH), 109.2 (A and B: ArCH), 101.6 (B: ArCH), 101.4 (A: ArCH), 64.2 (A: C**4**), 63.4 (B: C**4**), 52.1 (B: OCH_3), 51.8 (A: OCH_3), 49.1 (B: C**1**), 48.0 (A: C**1**), 39.6 (B: C**3**), 38.7 (A: C**3**), 34.8 (B: C**5**), 33.6 (A: C**5**), 30.9 (B: NCH_3), 30.0 (A: NCH_3), 26.3 (B: C**6**), 24.6 (A: C**6**), 22.0 (A: C**2**), 21.5 (B: C**2**).

HRMS: (ESI^+) Calculated for $C_{17}H_{23}N_2NaO_3$: 287.1754. Found $[M+Na]^+$: 287.1760.

Methyl 2-(benzofuran-2-ylmethyl)-2-methylpyrrolidine-1-carboxylate (7ab)

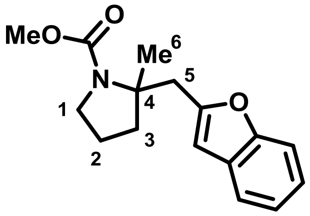

General procedure H: Conditions: 5.0 mol% $Pd_2(dba)_3$; 20 mol% PA-Ph (**L-1**); 25 mol% Et_3N; 200 mol% 2-benzofuranylboronic acid pinacol ester; PhMe (0.4 M); 130 °C. Substrate **6a** (36.7 mg, 0.100 mmol) was employed. FCC (eluent: 19:1 PhMe:EtOAc) afforded **7ab** (15.6 mg, 57 %) as a pale yellow oil.

ν_{max} / cm^{-1}: 2955, 2922, 2871, 1693, 1584, 1446, 1373, 1253, 1186, 1080.

This compound exists as an approximately 7:3 mixture of rotamers A and B.

δ_H (400 MHz, $CDCl_3$) 7.50 – 7.48 (1H, m, A and B: ArC<u>H</u>), 7.42 – 7.36 (1H, m, A and B: ArC<u>H</u>), 7.23 – 7.16 (2H, m, A and B: ArC<u>H</u>), 6.42 (0.7H, s, A: ArC<u>H</u>), 6.40 (0.3H, s, B: ArC<u>H</u>), 3.79 (0.9H, s, B: OC<u>H</u>3), 3.72 (2.1H, s, A: OC<u>H</u>3), 3.56 – 3.40 (1H, m, A and B: C**1**-<u>H</u>), 3,49 (0.7H, d, *J* = 14.0 Hz, A: C**5**-<u>H</u>), 3.34 – 3.28 (0.3H, m, B: C**1**-<u>H</u>’), 3.29 (0.3H, d, *J* = 14.0 Hz, B: C**5**-<u>H</u>), 3.23 – 3.17 (0.7H, m, A: C**1**-<u>H</u>’), 3.21 (0.7H, d, *J* = 14.0 Hz, A: C**5**-<u>H</u>’), 3.09 (0.3H, d, *J* = 14.0 Hz, B: C**5**-<u>H</u>’), 2.26 – 2.18 (1H, m, A and B: C**3**-<u>H</u>), 1.79 – 1.67 (2H, m, A and B: C**2**-<u>H</u> and C**3**-<u>H</u>’), 1.63 – 1.35 (1H, m, A and B: C**2**-<u>H</u>’) 1.49 (2.1H, s, A: C**6**-<u>H</u>3), 1.44 (0.9H, s, B: C**6**-<u>H</u>3).

δ_C (101 MHz, $CDCl_3$) 156.5 (A and B: <u>C</u>=O), 154.7 (A and B: Ar<u>C</u>), 154.6 (A and B: Ar<u>C</u>) 128.8 (A and B: Ar<u>C</u>),123.4 (B: Ar<u>C</u>H), 123.2 (A: Ar<u>C</u>H), 122.5 (B: Ar<u>C</u>H), 122.4 (A: Ar<u>C</u>H), 120.3 (A and B: Ar<u>C</u>H), 110.8 (A and B: Ar<u>C</u>H), 104.7 (A and B: Ar<u>C</u>H), 63.2 (A: C**4**), 62.5 (B: C**4**), 52.1 (B: O<u>C</u>H3), 51.8 (A: O<u>C</u>H3), 49.0 (B: C**1**), 48.0 (A: C**1**), 39.5 (B: C**3**), 38.4 (A: C**3**), 37.8 (B: C**5**), 36.5 (A: C**5**), 26.3 (B: C**6**), 25.2 (A: C**6**), 22.0 (A: C**2**), 21.5 (B: C**2**).

HRMS: (ESI^+) Calculated for $C_{16}H_{19}NNaO_3$: 296.1263. Found $[M+Na]^+$: 296.1271.

Methyl 2-methyl-2-(thiophen-2-ylmethyl)pyrrolidine-1-carboxylate (7ac)

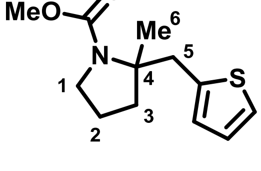

General procedure H: Conditions: 5.0 mol% $Pd_2(dba)_3$; 20 mol% PA-Ph (**L-1**); 25 mol% Et_3N; 200 mol% thiophene-2-boronic acid pinacol ester; PhMe (0.4 M); 130 °C. Substrate **6a** (36.7 mg, 0.100 mmol) was employed. FCC (eluent: 19:1 PhMe:EtOAc) afforded **7ac** (14.1 mg, 59 %) as a pale yellow oil.

ν_{max} / cm^{-1}: 2953, 1690, 1500, 1440, 1369, 1188, 1128, 1078.

This compound exists as an approximately 7:3 mixture of rotamers A and B.

δ_H (400 MHz, $CDCl_3$) 7.17 – 7.13 (1H, m, A and B: ArCH), 6.92 (1H, dd, *J* = 5.0, 3.5 Hz, A and B: ArCH), 6.78 – 6.76 (1H, m, A and B: ArCH), 3.79 – 3.67 (0.7H, m, A: C**5**-H), 3.79 (0.9H s, B: OCH_3), 3.71 (2.1H, s, A: OCH_3), 3.51 – 3.40 (1.3H, m, A and B: C**1**-H, B: C**5**-H), 3.17 – 3.11 (1H, m, A and B: C**1**-H'), 3.03 (0.7H, d, *J* = 14.5 Hz, A: C**5**-H'), 2.99 (0.3H, d, *J* = 14.5 Hz, B: C**5**-H') 2.07 – 2.00 (1H, m, A and B: C**3**-H), 1.69 – 1.61 (2H, m, A and B: C**2**-H and C**3**-H'), 1.47 – 1.37 (1H, m, A and B: C**2**-H'), 1.47 (2.1H, s, A: C**6**-H_3), 1.41 (0.9H, s, B: C**6**-H_3).

δ_C (101 MHz, $CDCl_3$) 154.6 (A and B: C=O), 140.4 (B: ArC), 140.3 (A: ArC), 126.8 (A and B: ArCH), 126.4 (A and B: ArCH), 124.5 (B: ArCH), 124.3 (A: ArCH), 64.0 (B: C**4**), 63.6 (A: C**4**), 52.1 (B: OCH_3), 51.9 (A: OCH_3), 49.2 (B: C**1**), 48.2 (A: C**1**), 2 × 38.8 (B: C**3** and C**5**), 2 × 37.7 (A: C**3** and C**5**), 25.4 (A and B: C**6**), 22.0 (A and B: C**2**).

HRMS: (ESI$^+$) Calculated for $C_{12}H_{17}NNaO_2S$: 262.0878. Found $[M+Na]^+$: 262.0882.

Methyl 2-(benzo[*b*]thiophen-2-ylmethyl)-2-methylpyrrolidine-1-carboxylate (7ad)

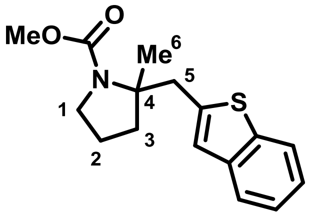

General procedure H: Conditions: 5.0 mol% $Pd_2(dba)_3$; 20 mol% PA-Ph (**L-1**); 25 mol% Et_3N; 200 mol% benzo[*b*]thiophene-2-boronic acid pinacol ester; PhMe (0.4 M); 130 °C. Substrate **6a** (36.7 mg, 0.100 mmol) was employed. FCC (eluent: 19:1 PhMe:EtOAc) afforded **7ad** (20.8 mg, 72 %) as a pale yellow oil.

ν_{max} / cm^{-1}: 2956, 2874, 1691, 1501, 1441, 1371, 1189, 1079.

This compound exists as an approximately 7:3 mixture of rotamers A and B.

δ_H (400 MHz, $CDCl_3$) 7.76 (1H, d, *J* = 8.0 Hz, A and B: ArCH), 7.69 (1H, d, *J* = 8.0 Hz, A and B: ArCH), 7.33 – 7.24 (2H, m, A and B: ArCH), 7.02 – 7.00 (1H, m, A and B: ArCH), 3.83 – 3.68 (0.7H, m, A: C**5**-H), 3.83 (0.9H, s, B: OCH_3), 3.76 (2.1H, s, A: OCH_3), 3.60 – 3.55 (m, 0.6H, B: C**1**-H and C**5**-H) 3.49 – 3.43 (0.7H, m, A: C**1**-H), 3.31 – 3.20 (1H, m, A and B: C**1**-H'), 3.16 (0.7H, d, *J* = 14.5 Hz, A: C**5**-H'), 3.07 (0.3H, d, *J* = 14.5 Hz, B: C**5**-H'), 2.17 – 2.06 (1H, m, A and B: C**3**-H), 1.76 – 1.65 (2H, m, A and B: C**2**-H and C**3**-H'), 1.52 – 1.43 (1H, m, A and B: C**2**-H') 1.52 (2.1H s, A: C**6**-H_3), 1.46 (0.9H, s, B: C**6**-H_3).

δ_C (101 MHz, $CDCl_3$) 154.6 (A and B: C=O), 141.6 (A and B: ArC), 140.2 (A and B: ArC), 139.7 (A and B: ArC), 124.0 (B: ArCH), 123.9 (A: ArCH), 123.6 (B: ArCH), 123.5 (A: ArCH), 123.4 (A: ArCH), 123.3 (B: ArCH) 122.8 (A and B: ArCH), 122.1 (B: ArCH), 122.0 (A: ArCH), 63.6 (A and B: C**4**), 52.1 (B: OCH_3), 51.9 (A: OCH_3), 49.2 (B: C**1**), 48.2 (A: C**1**), 39.9 (B: C**5**) 38.8 (B: C**3**), 38.7 (A: C**5**), 37.7 (A: C**3**), 26.6 (B: C**6**), 25.4 (A: C**6**), 22.0 (A: C**2**), 21.5 (B: C**2**).

HRMS: (ESI^+) Calculated for $C_{16}H_{19}NNaO_2S$: 312.1034. Found $[M+Na]^+$: 312.1037.

Ethyl 4-methyl-2-phenylpent-4-enoate

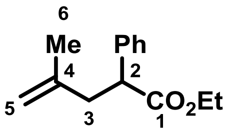

To a solution of diisopropylamine (4.38 mL, 31.3 mmol) in anhydrous THF (38 mL) at 0 °C was added ^nBuLi (1.6 M, 18.6 mL, 29.8 mmol) dropwise over around 15 minutes. The reaction mixture was cooled to -78 °C and a solution of ethyl phenylacetate (3.99 mL, 25 mmol) in anhydrous THF (75 mL) was added dropwise. The reaction mixture was stirred at -78 °C for 40 minutes before addition of methallyl chloride (3.67 mL, 37.5 mmol), the reaction mixture was stirred at -78 °C for a futher 2 hours before addition of saturated aqueous NH_4Cl (50 mL) and extraction with Et_2O (100 mL). The organic phase was washed with 1.0 M HCl (50 mL) and brine (50 mL) before being dried over Na_2SO_4 and concentrated *in vacuo*. FCC (eluent: 97:3 hexane:EtOAc) afforded the title compound (3.27 g, 60 %) as a colorless oil.

ν_{max} / cm^{-1}: (*film*) 3079 (m), 2980 (m), 1731 (s), 1454 (m), 1152 (s).

δ_H (400 MHz, $CDCl_3$) 7.40 – 7.31 (4H, m, ArCH), 7.30 – 7.25 (1H, m, ArCH), 4.78 – 4.77 (1H, m, C**5**-H), 4.73 – 4.72 (1H, m, C**5**-H’), 4.27 – 3.98 (2H, m, OCH_2CH_3), 3.81 (1H, dd, *J* = 9.5, 6.5 Hz, C**2**-H), 2.86 (1H, dd, *J* = 15.0, 9.5 Hz, C**3**-H), 2.45 (1H, dd, *J* = 15.0, 6.5 Hz, C**3**-H’), 1.75 (3H, s, C**6**-H_3), 1.23 (3H, t, *J* = 7.0 Hz, OCH_2CH_3).

δ_C (101 MHz, $CDCl_3$) 173.5 (C**1**), 142.7 (C**4**), 139.0 (ArC), 128.5 (ArCH), 127.9 (ArCH), 127.2 (ArCH), 112.1 (C**5**), 60.7 (OCH_2CH_3), 50.1 (C**2**), 41.4 (C**3**), 22.6 (C**6**), 14.1 (OCH_2CH_3).

HRMS: (ESI^+) Calculated for $C_{14}H_{19}O$: 219.1380. Found $[M+H]^+$: 219.1386.

4-Methyl-2-phenylpent-4-en-1-ol

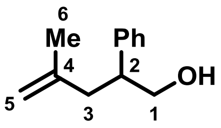

General procedure C: The preceding ester (2.73 g, 12.5 mmol) was employed using anhydrous Et_2O as solvent and 1.0 eq. $LiAlH_4$ (1.0 M in Et_2O). FCC (eluent: 7:3 hexane:Et_2O) afforded the title compound (2.07 g, 94 %) as a colorless oil.

ν_{max} / cm^{-1}: 3341, 3028, 2923, 1939, 1766, 1648, 1494 1452, 1374, 1172, 1066, 1031.

δ_H (400 MHz, $CDCl_3$) 7.41 – 7.31 (2H, m, ArCH), 7.29 – 7.25 (3H, m, ArCH), 4.76 – 4.73 (1H, m, C**5**-H), 4.71 – 4.68 (1H, m, C**5**-H’), 3.82 (1H, ddd, *J* = 11.0, 7.5, 5.5 Hz, C**1**-H), 3.75 (1H, ddd, *J* = 11.0, 7.5, 5.5 Hz, C**1**-H’), 3.06 (1H, dddd, *J* = 8.0, 7.5, 7.5, 5.5 Hz, C**2**-H), 2.49 (1H, ddd, *J* = 14.0, 7.5, 1.0 Hz, C**3**-H), 2.37 (1H, ddd, *J* = 14.0, 8.0, 1.0 Hz, C**3**-H’), 1.73 (3H, s, C**6**-H_3), 1.38 – 1.32 (1H, m, OH).

δ_C (101 MHz, $CDCl_3$) 143.4 (ArC), 142.1 (C**4**), 128.6 (ArCH), 128.0 (ArCH), 126.8 (ArCH), 112.4 (C**4**), 67.3 (C**1**), 46.2 (C**2**), 40.8 (C**3**), 22.4 (C**6**).

HRMS: (ESI$^+$) Calculated for $C_{12}H_{16}ONa$: 199.1093. Found $[M+Na]^+$: 199.1091.

Methyl (4-methyl-2-phenylpent-4-en-1-yl)((pentafluorobenzoyl)oxy)carbamate (6b)

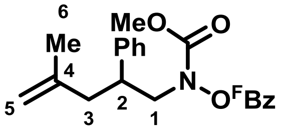

General procedure D: The preceding alcohol (264 mg, 1.50 mmol) was employed with **1c**. FCC (eluent: 1:4 hexane:PhMe) afforded **6c** (275 mg, 41 %) as a pale yellow oil.

ν_{max} / cm^{-1}: 2959, 2924, 1786, 1735, 1651, 1524, 1503, 1450, 1378, 1326, 1229, 1176, 1077, 1003.

δ_H (400 MHz, $CDCl_3$) 7.31 – 7.23 (2H, m, ArCH), 7.22 – 7.12 (3H, m, ArCH), 4.71 – 4.70 (1H, m, C**5**-H), 4.63 – 4.62 (1H, m, C**5**-H’), 4.00 (1H, dd, *J* = 15.0, 6.5 Hz, C**1**-H), 3.88 (1H, dd, *J* = 15.0, 8.5 Hz, C**1**-H’), 3.65 (3H, s, OCH_3), 3.22 (1H, dddd, *J* = 8.5, 8.5, 6.5, 6.5 Hz, C**2**-H), 2.52 (1H, dd, *J* = 14.0, 6.5 Hz, C**3**-H), 2.38 (1H, dd, *J* = 14.0, 8.5 Hz, C**3**-H’), 1.66 (3H, s, C**6**-H_3).

δ_C (101 MHz, $CDCl_3$) 155.8 (MeO-C=O), 142.5 (ArC), 141.4 (C**4**), 128.4 (ArCH), 127.9 (ArCH), 126.8 (ArCH), 112.9 (C**5**), 56.4 (C**1**), 53.8 (OCH_3), 41.7 (C**2**), 41.7 (C**3**), 22.2 (C**6**).

The signals corresponding to the pentafluorobenzoyl group could not be resolved due to their weak intensity.

δ_F (377 MHz, $CDCl_3$) -135.7 (2F, dp, *J* = 18.5, 6.5 Hz), -145.9 (1F, tt, *J* = 21.0, 5.5 Hz), -159.1 – -159.4 (2F, m).

HRMS: (ESI$^+$) Calculated for $C_{21}H_{18}F_5NNaO_4$: 466.1054. Found $[M+Na]^+$: 466.1047.

Methyl 2-(benzo[*b*]thiophen-2-ylmethyl)-2-methyl-4-phenylpyrrolidine-1-carboxylate (7b)

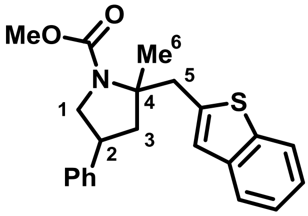

General procedure H: Conditions: 5.0 mol% $Pd_2(dba)_3$; 20 mol% PA-Ph (**L-1**); 25 mol% Et_3N; 200 mol% benzo[*b*]thiphene-2-boronic acid pinacol ester; PhMe (0.4 M); 130 °C. Substrate **6b** (44.3 mg, 0.100 mmol) was employed. FCC (eluent: 19:1 PhMe:EtOAc) afforded **7b** (25.4 mg, 69 %, 3:2 mixture of diastereomers A and B) as a pale yellow oil.

ν_{max} / cm^{-1}: 3028, 2951, 2925, 2864, 1696, 1603, 1520, 1499, 1442, 1372, 1321, 1288, 1249, 1220, 1191, 1123, 1094, 1072.

^{1}H and ^{13}C spectra acquired at high temperature:

δ_H (500 MHz, CD_3CN, 65 °C) 7.85 (1H, dd, *J* = 9.0, 8.5 Hz, A and B: ArC<u>H</u>), 7.78 (1H, dd, *J* = 9.0, 9.0 Hz, A and B: ArC<u>H</u>), 7.39 – 7.08 (8H, m, A and B: ArC<u>H</u>), 4.04 – 3.89 (1H, m, A: C**1**-<u>H</u>, B: C**5**-<u>H</u>), 3.87 – 3.69 (3.8H, m, A: OC$\underline{H}_3$, B: C**1**-<u>H</u>, C**5**-<u>H</u>’ and OC$\underline{H}_3$), 3.48 (0.6H, dddd, *J* = 12.5, 11.5, 7.0, 7.0 Hz, A: C**2**-<u>H</u>), 3.38 (0.4H, dd, *J* = 10.5, 10.5 Hz, B: C**1**-<u>H</u>’), 3.30 – 3.19 (1.2H, m, A: C**5**-$\underline{H}_2$), 3.06 (0.6H, dd, *J* = 11.5, 11.0 Hz, A: C**1**-<u>H</u>’), 2.86 – 2.75 (0.4H, m, B: C**2**-<u>H</u>), 2.60 (0.4H, dd, *J* = 13.0, 7.0 Hz, B: C**3**-<u>H</u>), 2.39 – 2.25 (0.6H, m, A: C**3**-<u>H</u>), 2.17 – 2.07 (0.6H, m, A: C**3**-<u>H</u>’), 1.99 – 1.97 (0.4H, m, B: C**3**-<u>H</u>’), 1.62 (3H, s, A and B: C**6**-$\underline{H}_3$).

^{13}C signals for diastereomer A only:

δ_C (126 MHz, CD_3CN, 65 °C) 155.7 (<u>C</u>=O), 143.3 (Ar<u>C</u>), 142.8 (Ar<u>C</u>), 142.3 (Ar<u>C</u>), 141.5 (Ar<u>C</u>), 129.8 (Ar<u>C</u>H), 128.3 (Ar<u>C</u>H), 128.0 (Ar<u>C</u>H), 125.5 (Ar<u>C</u>H), 125.4 (Ar<u>C</u>H), 125.1 (Ar<u>C</u>H), 124.2 (Ar<u>C</u>H), 123.3 (Ar<u>C</u>H), 65.3 (C**4**), 55.8 (C**1**), 52.7 (O<u>C</u>H_3), 45.7 (C**3**), 41.0 (C**2**), 39.9 (C**5**), 26.0 (C**6**).

HRMS: (ESI^+) Calculated for $C_{22}H_{23}NNaO_2S$: 388.1342. Found $[M+Na]^+$: 388.1344.

Methyl 4-methyl-3-phenylpent-4-enoate

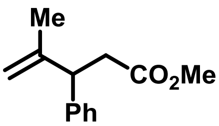

Prepared according to a literature procedure using 3-phenyl-2-methyl-2-propen-1-ol (2.47 mL, 17.2 mmol) and used in the next step without further purification.[44]

δ_H (400 MHz, $CDCl_3$) 7.31 – 7.24 (2H, m), 7.23 – 7.13 (3H, m), 4.93 – 4.89 (1H, m), 4.89 – 4.86 (1H, m), 3.79 (1H, dd, *J* = 8.0, 8.0 Hz), 3.61 (3H, s), 2.86 (1H, dd, *J* = 15.5, 8.0 Hz), 2.71 (1H, dd, *J* = 15.5, 8.0 Hz), 1.61 (3H, s).

The spectroscopic properties were consistent with the data available in the literature.[44]

4-Methyl-3-phenylpent-4-en-1-ol

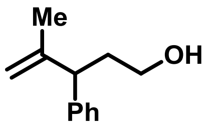

__General procedure C:__ The preceding crude ester was employed using anhydrous Et_2O as solvent and 1.0 eq. $LiAlH_4$ (1.0 M in Et_2O). FCC (eluent: 7:3 hexane:Et_2O) afforded the title compound (1.92 g, 63 % over two steps) as a colorless oil.

δ_H (400 MHz, $CDCl_3$) 7.34 – 7.27 (2H, m), 7.25 – 7.14 (3H, m), 4.95 (1H, dq, *J* = 1.5, 1.0 Hz), 4.86 (1H, dq, *J* = 1.5, 1.5 Hz), 3.68 – 3.51 (2H, m), 3.42 (1H, dd, *J* = 7.5, 7.5 Hz), 2.14 (1H, ddt, *J* = 13.5, 7.5, 7.5 Hz), 1.98 (1H, ddt, *J* = 13.5, 7.5, 6.0 Hz), 1.59 (3H, dd, *J* = 1.5, 1.0 Hz), 1.23 (1H, t, *J* = 5.5 Hz).

δ_C (101 MHz, $CDCl_3$) 147.8, 142.9, 128.4, 127.8, 126.4, 110.5, 61.3, 49.0, 35.8, 21.0.

The spectroscopic properties were consistent with the data available in the literature.[45]

Methyl (4-methyl-3-phenylpent-4-en-1-yl)((pentafluorobenzoyl)oxy)carbamate (6c)

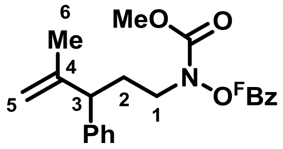

__General procedure D:__ The preceding alcohol (340 mg, 1.93 mmol) was employed with **1c**. FCC (eluent: 1:4 hexane:PhMe) afforded **6c** (455 mg, 53 %) as a colorless oil.

ν_{max} / cm^{-1}: 3027, 2942, 1785, 1733, 1650, 1523, 1502, 1448, 1374, 1326, 1266, 1174, 1078, 1000.

δ_H (400 MHz, $CDCl_3$) 7.34 – 7.25 (2H, m, ArC<u>H</u>), 7.24 – 7.19 (3H, m, ArC<u>H</u>), 4.94 – 4.93 (1H, m, C**5**-<u>H</u>), 4.89 – 4.88 (1H, m, C**5**-<u>H</u>’), 3.80 (3H, s, OC<u>H</u>3), 3.72 (1H, ddd, *J* = 15.0, 9.0, 6.0 Hz, C**1**-<u>H</u>), 3.63 (1H, ddd, *J* = 15.0, 9.0, 6.0 Hz, C**1**-<u>H</u>’), 3.34 (1H, dd, *J* = 7.5, 7.5 Hz, C**3**-<u>H</u>), 2.21 (1H, dddd, *J* = 13.5, 9.0, 7.5, 6.0 Hz, C**2**-<u>H</u>), 2.08 (1H, dddd, *J* = 13.5, 9.0, 7.5, 6.0 Hz, C**2**-<u>H</u>’), 1.59 (3H, s, C**6**-<u>H</u>3).

δ_C (101 MHz, $CDCl_3$) 156.0 (MeO–<u>C</u>=O), 146.8 (C**4**), 142.3 (Ar<u>C</u>), 128.4 (Ar<u>C</u>H), 127.7 (Ar<u>C</u>H), 126.6 (Ar<u>C</u>H), 110.9 (C**5**), 53.9 (O<u>C</u>H3), 50.1 (C**1**), 49.6 (C**3**), 30.1 (C**2**), 21.0 (C**6**).

The signals corresponding to the pentafluorobenzoyl group could not be resolved due to their weak intensity.

δ_F (377 MHz, $CDCl_3$) -136.1 (2F, dp, J = 18.0, 6.5 Hz), -146.1 (1F, tt, J = 21.0, 5.5 Hz), -158.9 – -160.3 (2F, m).

HRMS: (ESI^+) Calculated for $C_{21}H_{18}NNaO_4$: 466.1048. Found $[M+Na]^+$: 466.1035.

Methyl (2*R,3*R**)-2-(benzo[*b*]thiophen-2-ylmethyl)-2-methyl-3-phenylpyrrolidine-1-carboxylate (7c)**

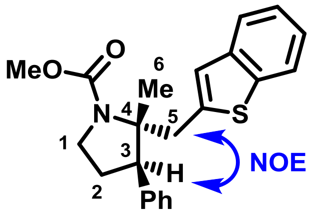

__**General procedure H:**__ Conditions: 5.0 mol% $Pd_2(dba)_3$; 20 mol% PA-Ph (**L-1**); 25 mol% Et_3N; 200 mol% benzo[*b*]thiphene-2-boronic acid pinacol ester; PhMe (0.4 M); 130 °C. Substrate **6c** (44.3 mg, 0.100 mmol) was employed. FCC (eluent: 19:1 PhMe:EtOAc) afforded **7c** (18.3 mg, 50 %) as a pale yellow oil.

*The product was assigned as the trans diastereomer based on the observed NOE correlation between the C**3** and the C**5** protons.*

ν_{max} / cm^{-1}: 2951, 2880, 1696, 1499, 1443, 1377, 1185, 1112, 1089, 1068.

This compound exists as an approximately 7:3 mixture of rotamers A and B.

δ_H (400 MHz, $CDCl_3$) 7.83 – 7.76 (2H, m, A and B: ArC<u>H</u>), 7.40 – 7.28 (7H, m, A and B: ArC<u>H</u>), 7.21 – 7.19 (1H, m, A and B: ArC<u>H</u>), 4.14 (0.7H, d, J = 15.0 Hz, A: C**5**-<u>H</u>), 3.85 – 3.82 (0.3H, m, B: C**1**-<u>H</u>) 3.85 (0.9H, s, B: $OC\underline{H}_3$), 3.82 (2.1H, s, A: $OC\underline{H}_3$), 3.77 – 3.69 (1H, m, A: C**1**-<u>H</u>, B: C**5**-<u>H</u>), 3.39 (1H, dd, J = 13.0, 6.0 Hz, A and B: C**3**-<u>H</u>), 3.31 – 3.17 (1H, m, A and B: C**1**-<u>H</u>'), 3.02 (0.3H, d, J = 15.0 Hz, B: C**5**-<u>H</u>'), 2.97 (0.7H, d, J = 15.0 Hz, A: C**5**-<u>H</u>'), 2.33 – 2.19 (1H, m, A and B: C**2**-<u>H</u>), 1.89 – 1.81 (1H, m, A and B: C**2**-<u>H</u>'), 1.15 (2.1H, s, A: C**6**-$\underline{H}_3$), 1.09 (0.9H, s, B: C**6**-$\underline{H}_3$).

δ_C (101 MHz, $CDCl_3$) 155.5 (B: <u>C</u>=O), 154.7 (A: <u>C</u>=O), 141.4 (A: Ar<u>C</u>), 141.0 (B: Ar<u>C</u>), 140.4 (A: Ar<u>C</u>), 140.3 (B: Ar<u>C</u>), 139.5 (A: Ar<u>C</u>), 139.4 (B: Ar<u>C</u>), 138.1 (A: Ar<u>C</u>), 138.1 (B: Ar<u>C</u>), 129.2 (B: Ar<u>C</u>H), 129.0 (A: Ar<u>C</u>H), 2 × 128.3 (A and B: Ar<u>C</u>H), 2 × 127.2 (A and B: Ar<u>C</u>H), 124.1 (B: Ar<u>C</u>H), 124.0 (A: Ar<u>C</u>H), 2 × 123.8 (A and B: Ar<u>C</u>H), 123.7 (B: Ar<u>C</u>H), 123.6 (A: Ar<u>C</u>H), 122.9 (B: Ar<u>C</u>H), 122.8 (A: Ar<u>C</u>H), 122.1 (B: Ar<u>C</u>H), 122.0 (A: Ar<u>C</u>H), 66.0 (A: C**4**), 65.4 (B: C**4**), 52.3 (B: $O\underline{C}H_3$), 52.1 (A: $O\underline{C}H_3$), 50.1 (B: C**3**), 49.0 (A: C**3**), 46.9 (B: C**1**), 46.4 (A: C**1**), 37.6 (B: C**5**), 36.5 (A: C**5**), 26.9 (A: C**2**), 26.4 (B: C**2**), 22.4 (B: C**6**), 21.4 (A: C**6**).

HRMS: (ESI^+) Calculated for $C_{22}H_{23}NNaO_2S$: 388.1342. Found $[M+Na]^+$: 388.1337.

Comparison of diastereocontrol achieved with aza-Heck and aza-Wacker protocols

To compare the diastereoselectivities achievable in an aza-Heck process versus an aza-Wacker process, an aza-Wacker substrate (methyl (E)-(3-phenylhex-4-en-1-yl)carbamate) analogous to ***2h*** *was prepared and its cyclization was studied. Under aza-Heck conditions, cyclization of* ***2h*** *generated* ***4h*** *in 77% yield and >20:1 d.r. after 24 hours (vide supra). Under aza-Wacker conditions reported by Andersson,*[46] *cyclization of methyl (E)-(3-phenylhex-4-en-1-yl)carbamate to* ***4h*** *occurred in 71 % yield and 4:1 d.r. after 24 hours. Under aza-Wacker conditions reported by Stahl,*[47] *cyclization of methyl (E)-(3-phenylhex-4-en-1-yl)carbamate to* ***4h*** *occurred in 37 % yield and 4:3 d.r. after 24 hours. Thus, the aza-Heck protocol is more efficient in terms of both yield and diastereocontrol. The experimental details are as follows:*

Methyl (*E*)-(3-phenylhex-4-en-1-yl)carbamate

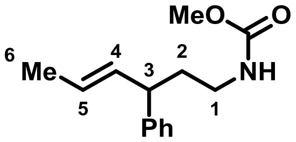

This compound was prepared from ***2h*** *by modification of General Procedure E. Formic acid was included to effect reduction of the N-O bond of* ***2h****.*

General procedure E: Conditions: 5 mol% $Pd_2(dba)_3$; 25 mol% PPh_3; 200 mol% Et_3N; 100 mol% HCO_2H; THF (0.1 M); 110 °C. Substrate **2h** (295 mg, 0.665 mmol) was employed. FCC (eluent: 5:1 petroleum ether:EtOAc) afforded the title compound (88.0 mg, 57 %) as a pale yellow oil.

ν_{max} / cm^{-1}: (*film*) 3333 (br s), 3026 (m), 2938 (m), 1697 (s), 1528 (s), 1251 (s).

δ_H (400 MHz, $CDCl_3$) 7.34 – 7.25 (2H, m, ArC$\underline{H}$), 7.24 – 7.14 (3H, m, ArC$\underline{H}$), 5.59 – 5.43 (2H, m, C**4**-$\underline{H}$ and C**5**-$\underline{H}$), 4.59 (1H, br s, N$\underline{H}$), 3.65 (3H, s, OC$\underline{H}_3$), 3.25 (1H, dt, *J* = 7.5, 7.5 Hz), 3.21 – 3.03 (2H, m, C**1**-$\underline{H}_2$), 1.93 – 1.83 (2H, m, C**2**-$\underline{H}_2$), 1.66 (3H, d, *J* = 6.0 Hz, C**6**-$\underline{H}_3$).

δ_C (101 MHz, $CDCl_3$) 157.1 (MeO-$\underline{C}$=O), 144.4 (Ar$\underline{C}$), 134.3 (C**4**), 128.7 (Ar$\underline{C}$H), 127.5 (Ar$\underline{C}$H), 126.4 (Ar$\underline{C}$H), 125.5 (C**5**), 52.1 (O$\underline{C}H_3$), 46.7 (C**3**), 39.7 (C**1**), 36.1 (C**2**), 18.1 (C**6**).

HRMS: (ESI^+) Calculated for $C_{14}H_{19}NNaO_2$: 256.1320. Found $[M+Na]^+$: 256.1313.

Methyl (2*S,3*S**)-3-phenyl-2-vinylpyrrolidine-1-carboxylate (4h)**

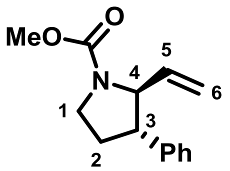

This compound was prepared using an adaptation of a literature procedure.[46]

A solution of the preceding compound (23.3 mg, 0.100 mmol) and $Pd(OAc)_2$ (2.2 mg, 10.0 μmol) in DMSO (0.4 mL) was stirred under an atmosphere of O_2 (balloon pressure) at 55 °C for 24 hours. The reaction mixture was diluted with Et_2O (5 mL) and washed with water (3 × 5 mL). The organic phase was dried over Na_2SO_4 and concentrated in vacuo. FCC (eluent: 9:1 petroleum ether:EtOAc) afforded **4h** (16.5 mg, 71 %, 4:1 mixture of *trans* and *cis* diastereomers) as a colorless oil.

*Conditions reported by Stahl ($Pd(OAc)_2$ (5 mol%); pyridine (10 mol%); O_2 (balloon pressure); xylene (0.1 M); heated to 80 °C for 24 hours)[47] for the aza-Wacker cyclization of carbamates provided **4h** in 37 % yield and approximately 4:3 d.r. (trans:cis) based on ^1H NMR analysis of the crude reaction mixture.*

The spectroscopic properties of the major diastereomer were the same as those described earlier.

The minor diastereomer exists as an approximately 1:1 mixture of rotamers. ^1H NMR signals for the minor cis diastereomer were obtained from 1D TOCSY analysis of the mixture of trans:cis diastereomers.

δ_H (400 MHz, $CDCl_3$) *Characteristic signals only:* 5.35 – 5.30 (1H, m, C**5**-H), 5.04 (1H, d, *J* = 12.5 Hz, C**6**-H), 4.97 (1H, d, *J* = 16.0 Hz, C**6**-H'), 4.77 – 4.70 and 4.67 – 4.60 (1H, m, C**4**-H), 3.72 – 3.61 (2H, m, C**1**-H_2), 3.59 – 3.45 (1H, m, C**3**-H), 2.36 – 2.22 (1H, m, C**2**-H), 2.17 – 2.08 (1H, m, C**2**-H').

δ_C (126 MHz, $CDCl_3$) *Characteristic signals only:* 134.7 and 134.2 (C**5**), 116.1 and 115.8 (C**6**), 62.9 and 62.5 (C**4**), 48.0 and 47.2 (C**3**), 45.7 and 45.5 (C**1**), 27.0 and 25.9 (C**2**).

References

(1) Masruri; Willis, A. C.; McLeod, M. D. *J. Org. Chem.* **2012**, *77*, 8480-8491.
(2) Ma, X.; Farndon, J. J.; Young, T. A.; Fey, N.; Bower, J. F. *Angew. Chem. Int. Ed.* **2017**, *56*, 14531-14535.
(3) Brenstrum, T.; Gerristma, D. A.; Adjabeng, G. M.; Frampton, C. S.; Britten, J.; Robertson, A. J.; McNulty, J.; Capretta, A. *J. Org. Chem.* **2004**, *69*, 7635-7639.
(4) Downing, J. H.; Floure, J.; Heslop, K.; Haddow, M. F.; Hopewell, J.; Lusi, M.; Phetmung, H.; Orpen, A. G.; Pringle, P. G.; Pugh, R. I.; Zambrano-Williams, D. *Organometallics* **2008**, *27*, 3216-3224.
(5) Matsumoto, K.; Aoki, Y.; Oshima, K.; Utimoto, K.; Rahman, N. A. *Tetrahedron* **1993**, *49*, 8487-8502.
(6) Seki, T.; Tanaka, S.; Kitamura, M. *Org. Lett.* **2012**, *14*, 608-611.
(7) Wang, Z.-X.; Shi, Y. *J. Org. Chem.* **1998**, *63*, 3099-3104.
(8) Kwon, H. Y.; Park, C. M.; Lee, S. B.; Youn, J.-H.; Kang, S. H. *Chem. Eur. J.* **2008**, *14*, 1023-1028.
(9) Heitz, D. R.; Rizwan, K.; Molander, G. A. *J. Org. Chem.* **2016**, *81*, 7308-7313.
(10) Hoover, J. M.; Stahl, S. S. *J. Am. Chem. Soc.* **2011**, *133*, 16901-16910.
(11) Kimura, M.; Ezoe, A.; Mori, M.; Iwata, K.; Tamaru, Y. *J. Am. Chem. Soc.* **2006**, *128*, 8559-8568.
(12) Fujita, S.; Abe, M.; Shibuya, M.; Yamamoto, Y. *Org. Lett.* **2015**, *17*, 3822-3825.
(13) Kelly, C. B.; Ovian, J. M.; Cywar, R. M.; Gosselin, T. R.; Wiles, R. J.; Leadbeater, N. E. *Org. Biomol. Chem.* **2015**, *13*, 4255-4259.
(14) Carman, L.; Kwart, L. D.; Hudlicky, T. *Synth. Commun* **1986**, *16*, 169-182.
(15) Ocejo, M.; Carrillo, L.; Badía, D.; Vicario, J. L.; Fernández, N.; Reyes, E. *J. Org. Chem.* **2009**, *74*, 4404-4407.
(16) Erkkilä, A.; Pihko, P. M. *Eur. J. Org. Chem.* **2007**, *2007*, 4205-4216.
(17) Bull, J. A.; Charette, A. B. *J. Am. Chem. Soc.* **2010**, *132*, 1895-1902.
(18) Zhang, Y. J.; Park, J. H.; Lee, S.-g. *Tetrahedron: Asymmetry* **2004**, *15*, 2209-2212.
(19) Jones-Mensah, E.; Nickerson, L. A.; Deobald, J. L.; Knox, H. J.; Ertel, A. B.; Magolan, J. *Tetrahedron* **2016**, *72*, 3748-3753.
(20) Zulfiqar, F.; Malik, A. *Z. Naturforsch., B: Chem. Sci.* **2001**, *56*, 1227.
(21) Armstrong, A.; Barsanti, P. A.; Clarke, P. A.; Wood, A. *J. Chem. Soc., Perkin Trans. 1* **1996**, 1373-1380.
(22) Lysenko, I. L.; Kim, K.; Lee, H. G.; Cha, J. K. *J. Am. Chem. Soc.* **2008**, *130*, 15997-16002.
(23) Faulkner, A.; Scott, J. S.; Bower, J. F. *Chem. Commun.* **2013**, *49*, 1521-1523.
(24) Klein, J. E. M. N.; Muller-Bunz, H.; Evans, P. *Org. Biomol. Chem.* **2009**, *7*, 986-995.
(25) Bothwell, J. M.; Angeles, V. V.; Carolan, J. P.; Olson, M. E.; Mohan, R. S. *Tetrahedron Lett.* **2010**, *51*, 1056-1058.
(26) Negishi, E.-i.; Pour, M.; Cederbaum, F. E.; Kotora, M. *Tetrahedron* **1998**, *54*, 7057-7074.
(27) Paioti, P. H. S.; Ketcham, J. M.; Aponick, A. *Org. Lett.* **2014**, *16*, 5320-5323.
(28) Tortajada, A.; Mestres, R.; Iglesias-Arteaga, M. A. *Synth. Commun* **2003**, *33*, 1809-1814.
(29) Belger, C.; Neisius, N. M.; Plietker, B. *Chem. Eur. J.* **2010**, *16*, 12214-12220.
(30) Shao, Y.; Yang, C.; Gui, W.; Liu, Y.; Xia, W. *Chem. Commun.* **2012**, *48*, 3560-3562.
(31) Miyata, K.; Kitamura, M. *Synthesis* **2012**, *44*, 2138-2146.
(32) Ha, M. W.; Lee, H.; Yi, H. Y.; Park, Y.; Kim, S.; Hong, S.; Lee, M.; Kim, M.-h.; Kim, T.-S.; Park, H.-g. *Adv. Synth. Catal.* **2013**, *355*, 637-642.
(33) Gras, J.-L.; Nouguier, R.; McHich, M. *Tetrahedron Lett.* **1987**, *28*, 6601-6604.
(34) Tian, H.; Liu, W.; Zhou, Z.; Shang, Q.; Liu, Y.; Xie, Y.; Liu, C.; Xu, W.; Tang, L.; Wang, J.; Zhao, G. *Molecules* **2016**, *21*, 1543.

(35) Quintavalla, A.; Lombardo, M.; Sanap, S. P.; Trombini, C. *Adv. Synth. Catal.* **2013**, *355*, 938-946.
(36) Miyata, K.; Kutsuna, H.; Kawakami, S.; Kitamura, M. *Angew. Chem. Int. Ed.* **2011**, *50*, 4649-4653.
(37) Jackl, M. K.; Kreituss, I.; Bode, J. W. *Org. Lett.* **2016**, *18*, 1713-1715.
(38) Bracher, F.; Mink, K. *Liebigs Ann. Chem.* **1995**, *1995*, 645-647.
(39) Kim, H.; Chang, S. *Angew. Chem. Int. Ed.* **2017**, *56*, 3344-3348.
(40) Zhang, Q.; Jin, H.-X.; Wu, Y. *Tetrahedron* **2006**, *62*, 11627-11634.
(41) Amouroux, R.; Ejjiyar, S. *Tetrahedron Lett.* **1991**, *32*, 3059-3062.
(42) Trost, B. M.; Shi, Y. *J. Am. Chem. Soc.* **1993**, *115*, 9421-9438.
(43) Tsimelzon, A.; Braslau, R. *J. Org. Chem.* **2005**, *70*, 10854-10859.
(44) Moretti, R. Perfuming ingredients of the floral and/or anis type. US2011/229426, 2011.
(45) Zhu, R.; Buchwald, S. L. *Angew. Chem. Int. Ed.* **2012**, *51*, 1926-1929.
(46) Rönn, M.; Bäckvall, J.-E.; Andersson, P. G. *Tetrahedron Lett.* **1995**, *36*, 7749-7752.
(47) Fix, S. R.; Brice, J. L.; Stahl, S. S. *Angew. Chem. Int. Ed.* **2002**, *41*, 164-166.

^{1}H and ^{13}C NMR spectra of novel compounds

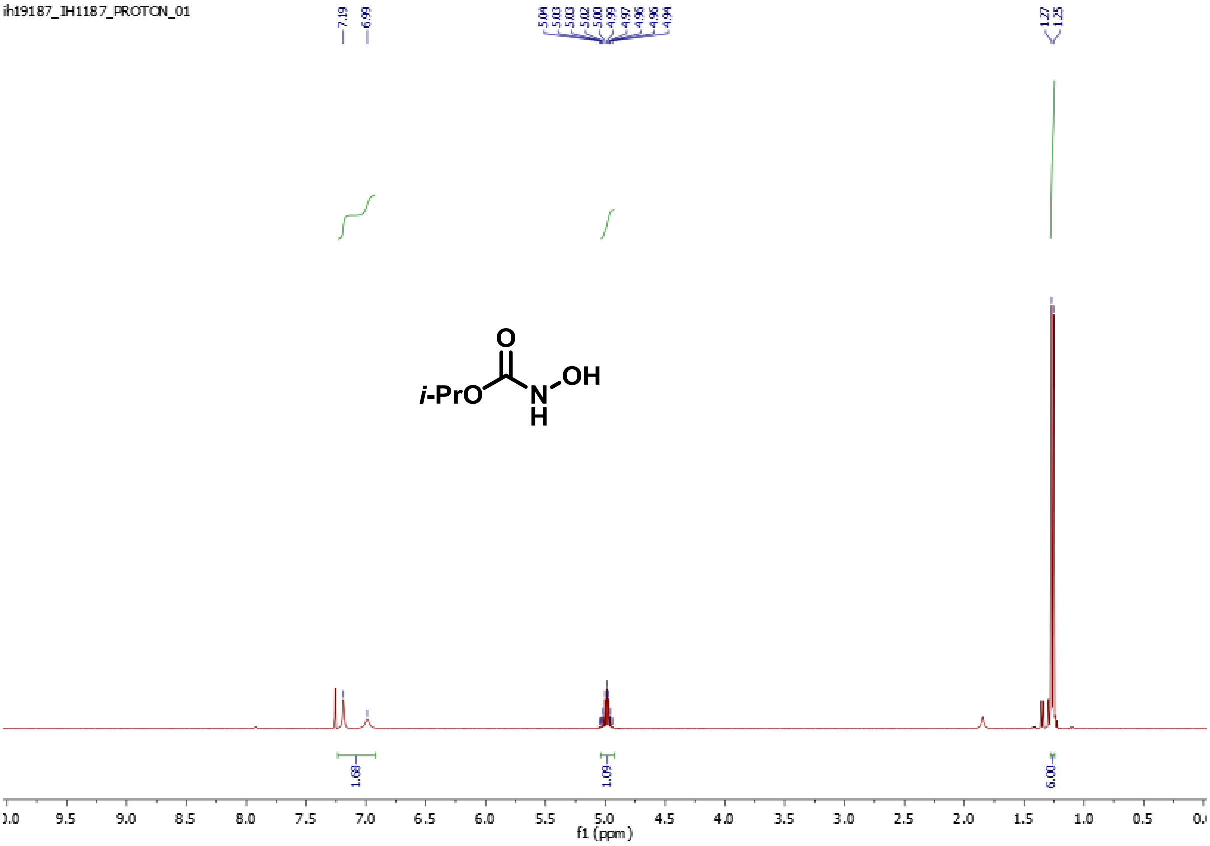

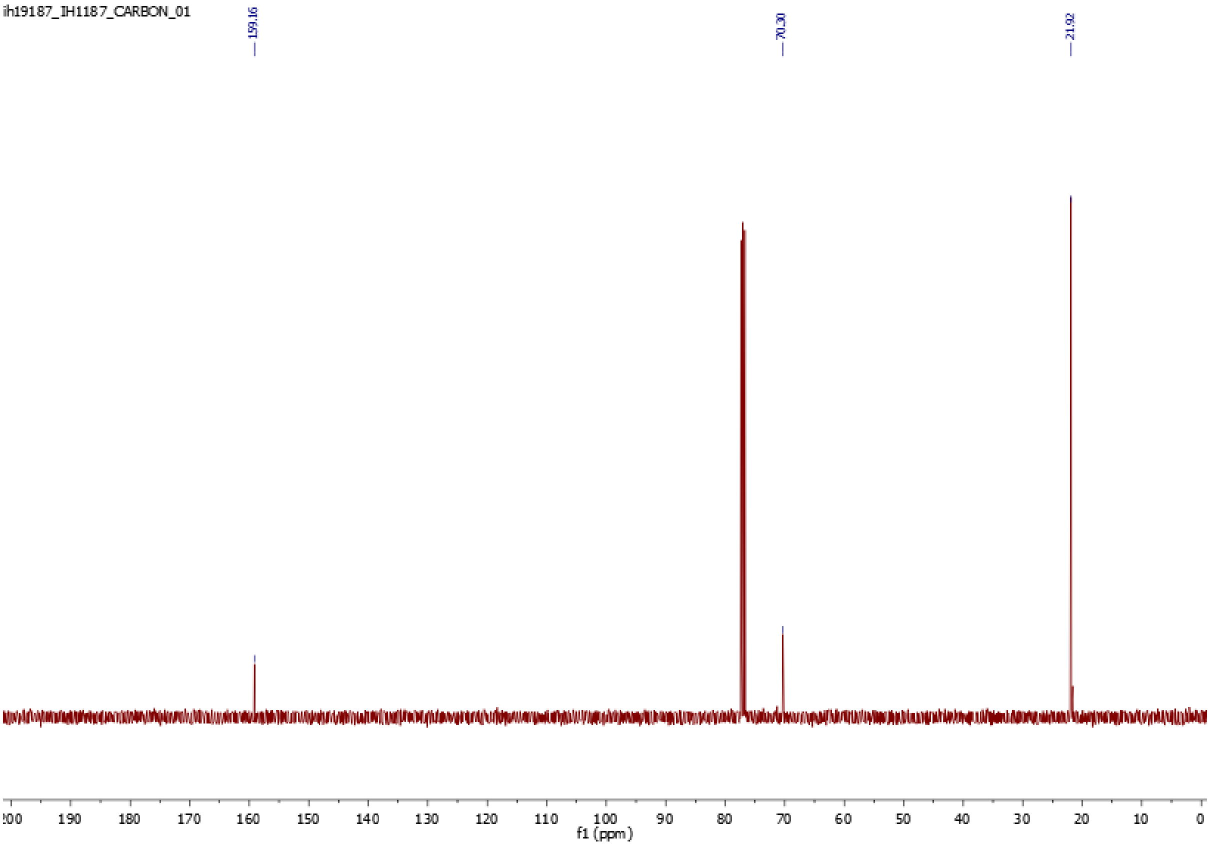

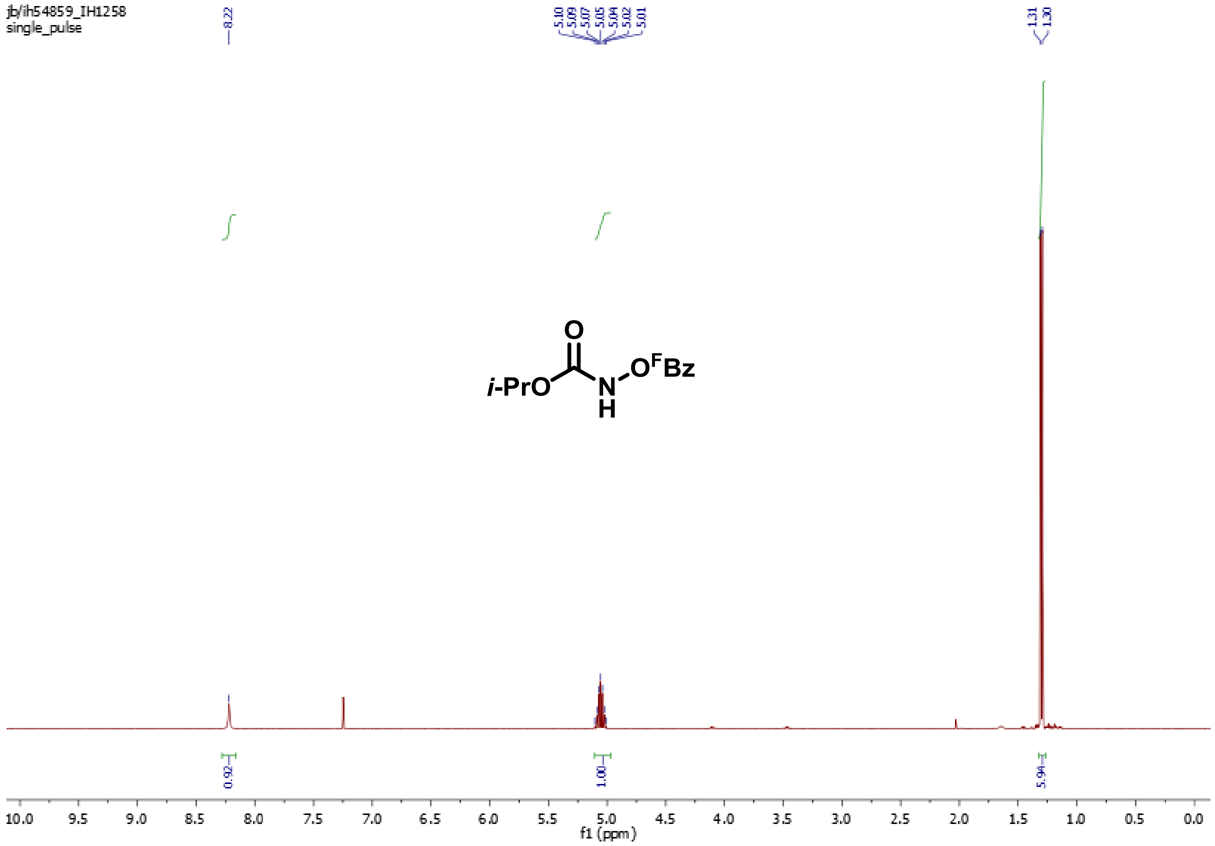
jb/ih54859_IH1258
single_pulse
8.22
5.10
5.09
5.07
5.05
5.04
5.02
5.01
1.31
1.30
i-PrO
O
N
H
O^FBz
0.92
1.00
5.94
f1 (ppm)

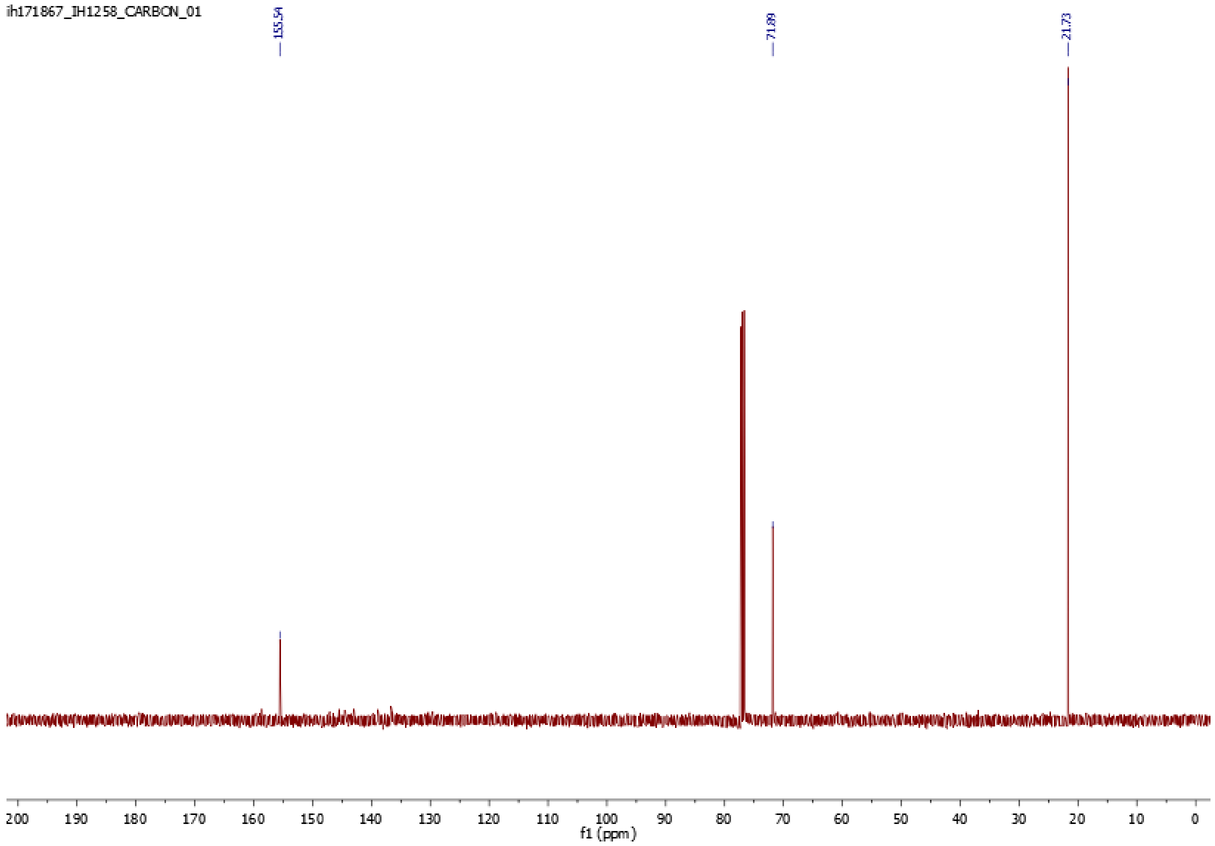
ih171867_IH1258_CARBON_01
155.54
71.89
21.73
f1 (ppm)

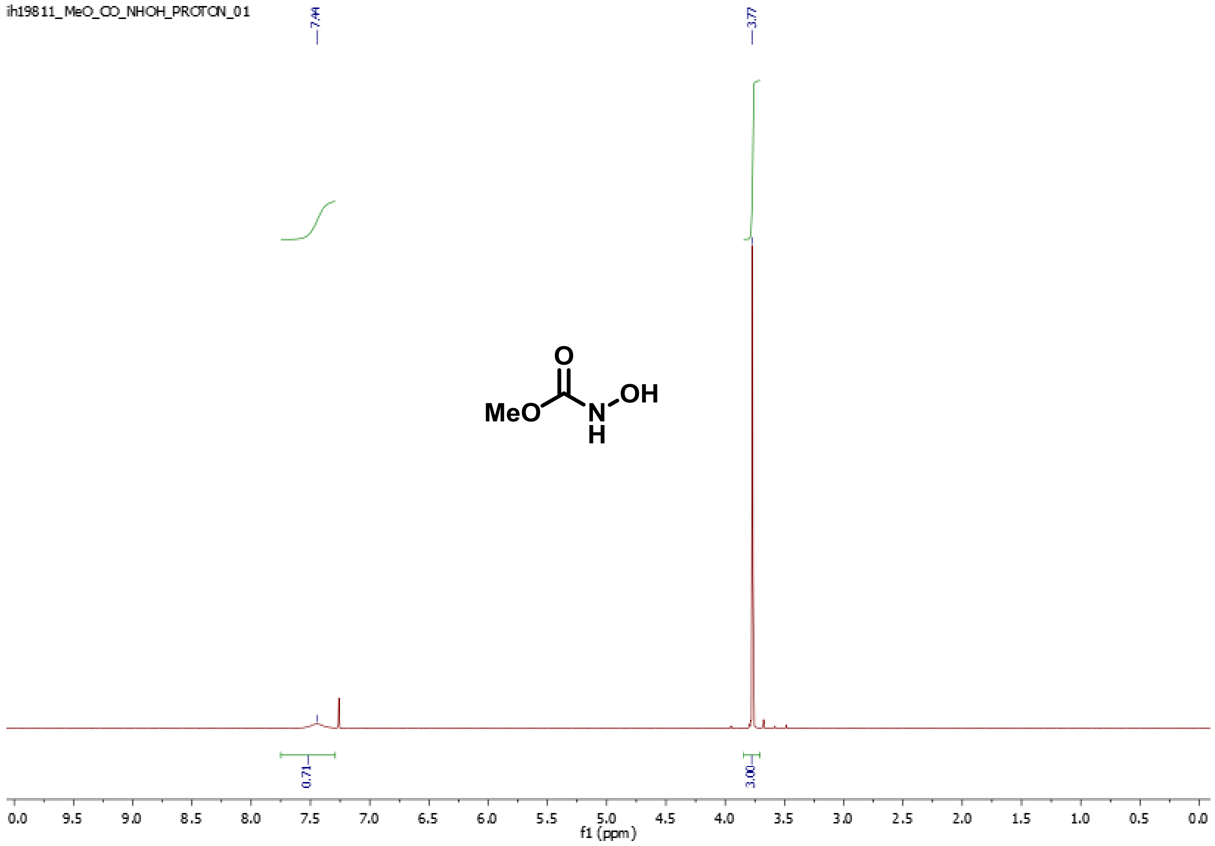
ih19811_MeO_CO_NHOH_PROTON_01
7.44
3.77
MeO
O
N
H
OH
0.71
3.00
f1 (ppm)

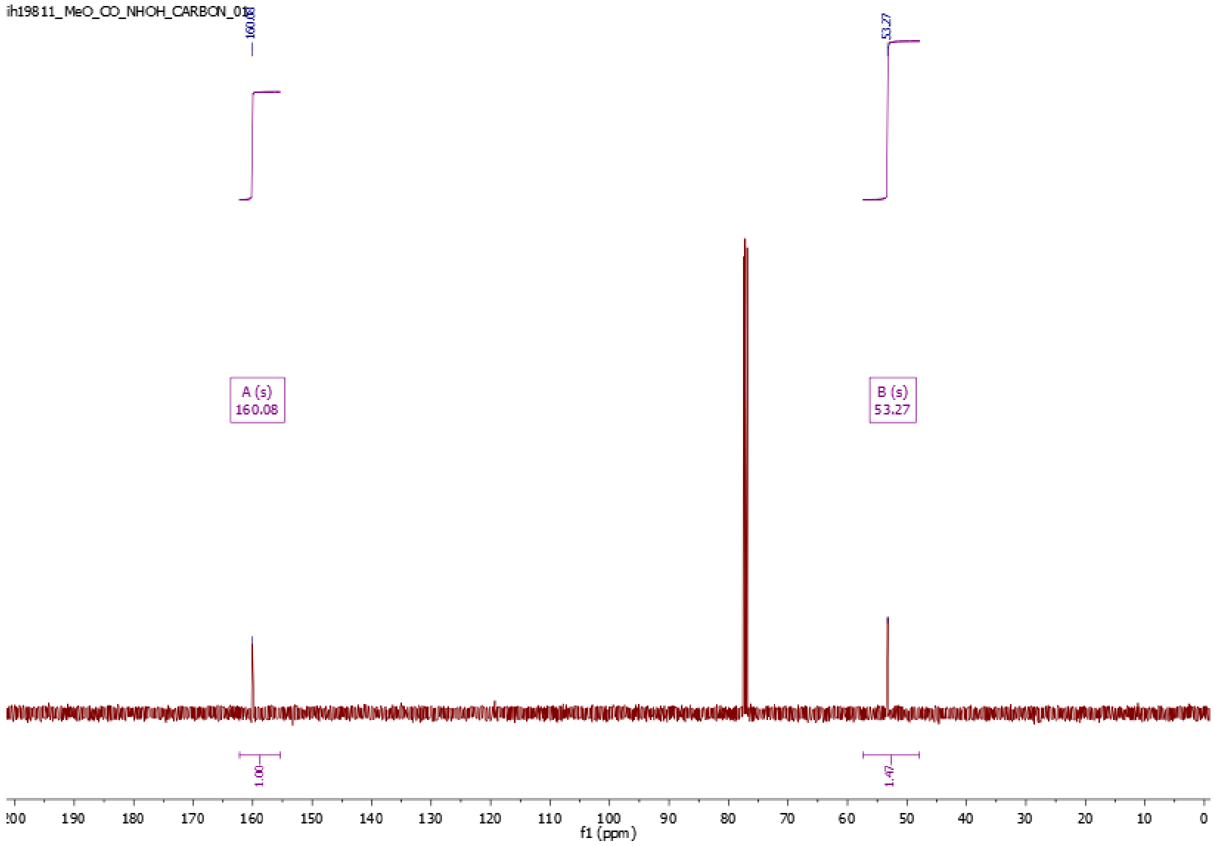
ih19811_MeO_CO_NHOH_CARBON_01
160.08
53.27
A (s)
160.08
B (s)
53.27
1.00
1.47
f1 (ppm)

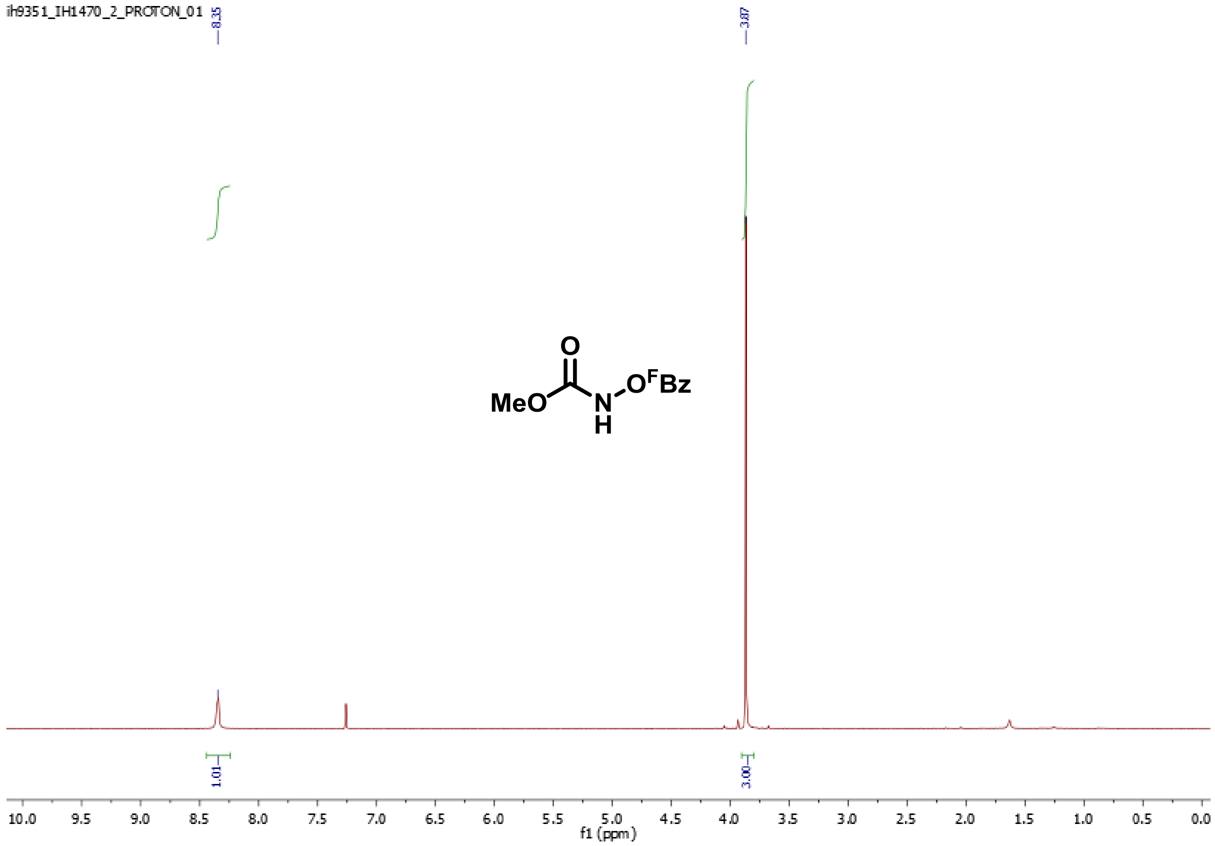
ih9351_IH1470_2_PROTON_01
8.35
3.87
MeO
O
N
H
O^FBz
1.01
3.00
f1 (ppm)

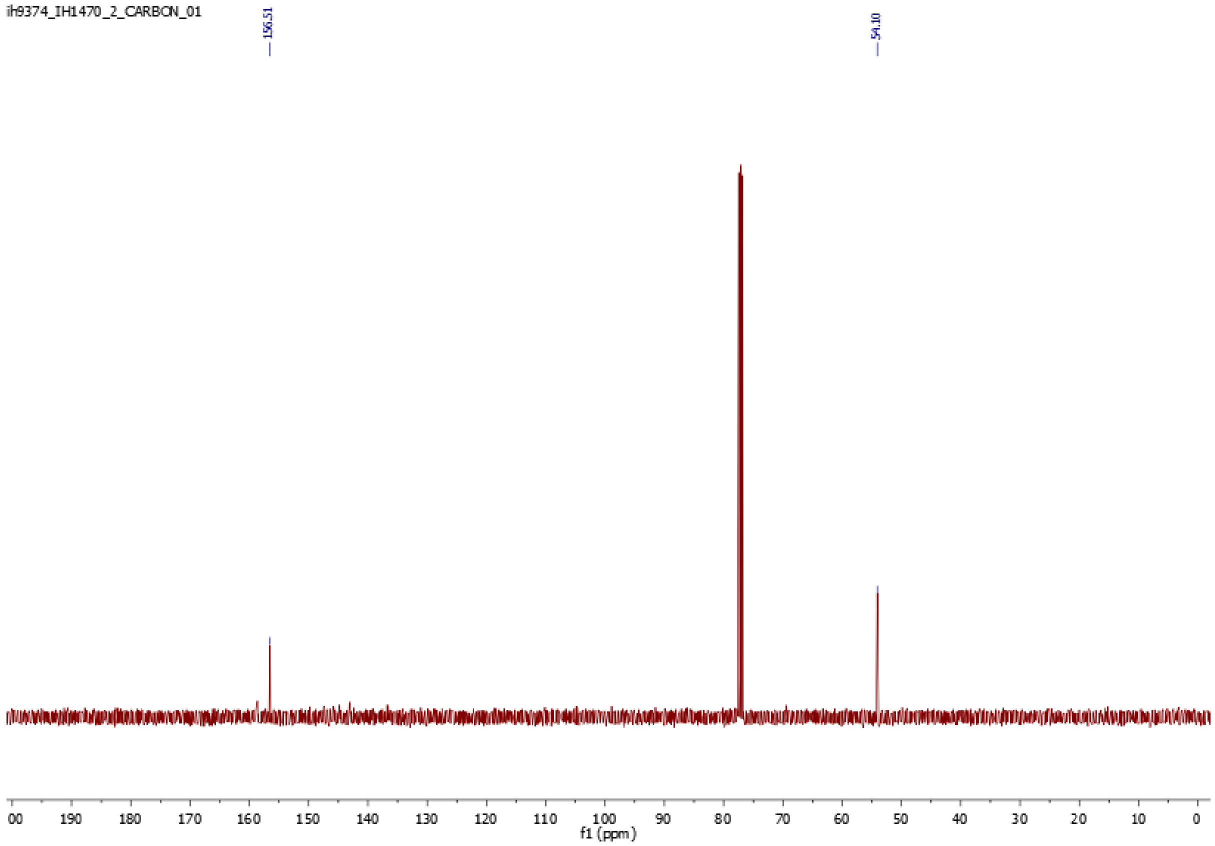
ih9374_IH1470_2_CARBON_01
156.51
54.10
f1 (ppm)

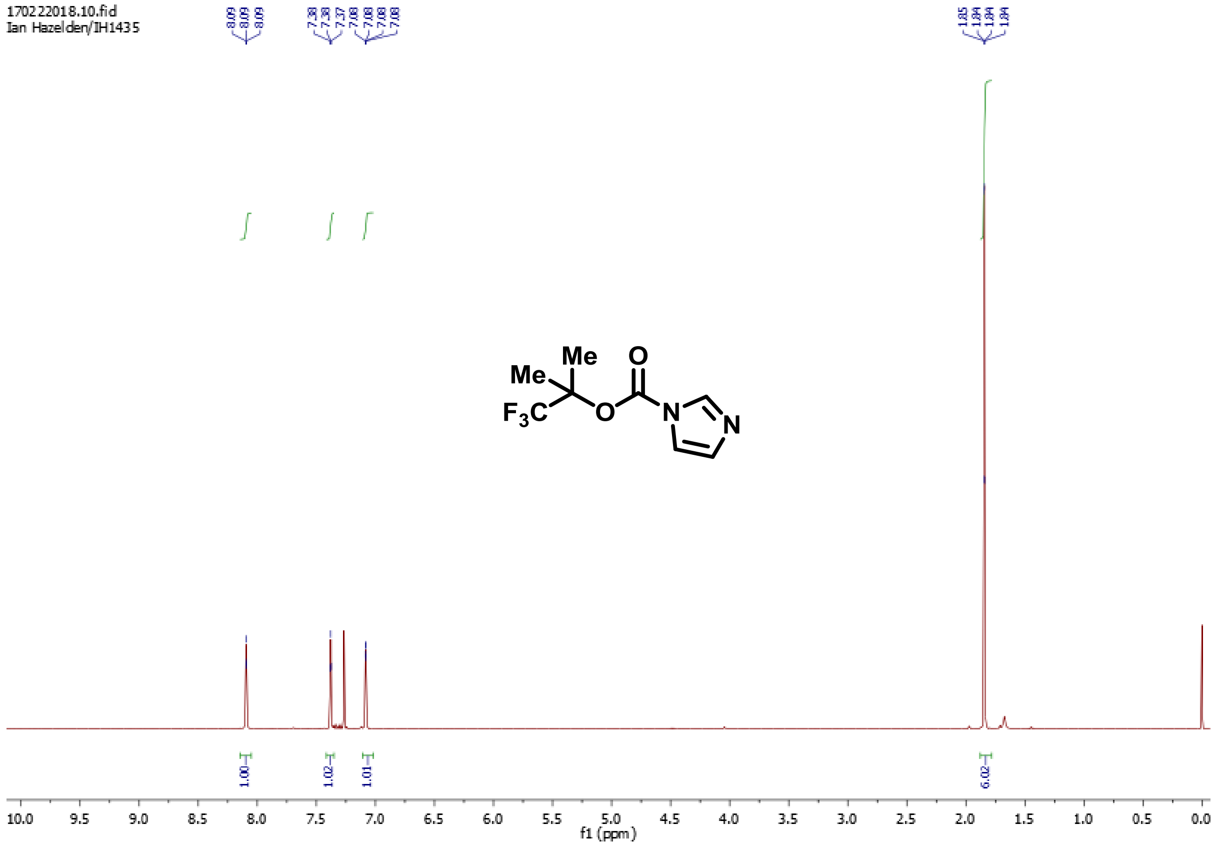
170222018.10.fid
Ian Hazelden/IH1435
8.09 8.09 8.09
7.38 7.38 7.37 7.08 7.08 7.08
1.85 1.84 1.84 1.84
Me
Me
O
F3C
O
N
N
1.00
1.02
1.01
6.02
f1 (ppm)

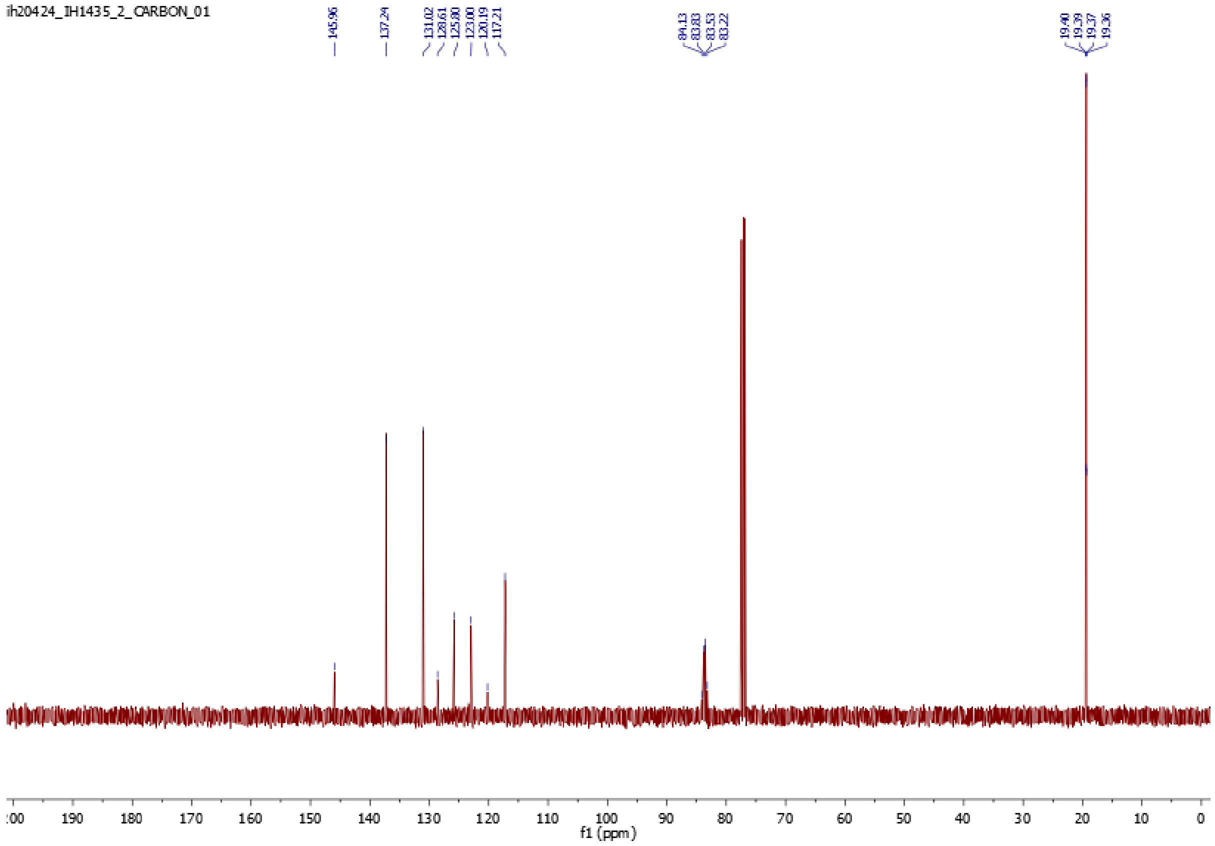
ih20424_IH1435_2_CARBON_01
145.96
137.24
131.02 128.61 125.80 123.00 120.19 117.21
84.13 83.83 83.53 83.22
19.40 19.39 19.37 19.36
f1 (ppm)

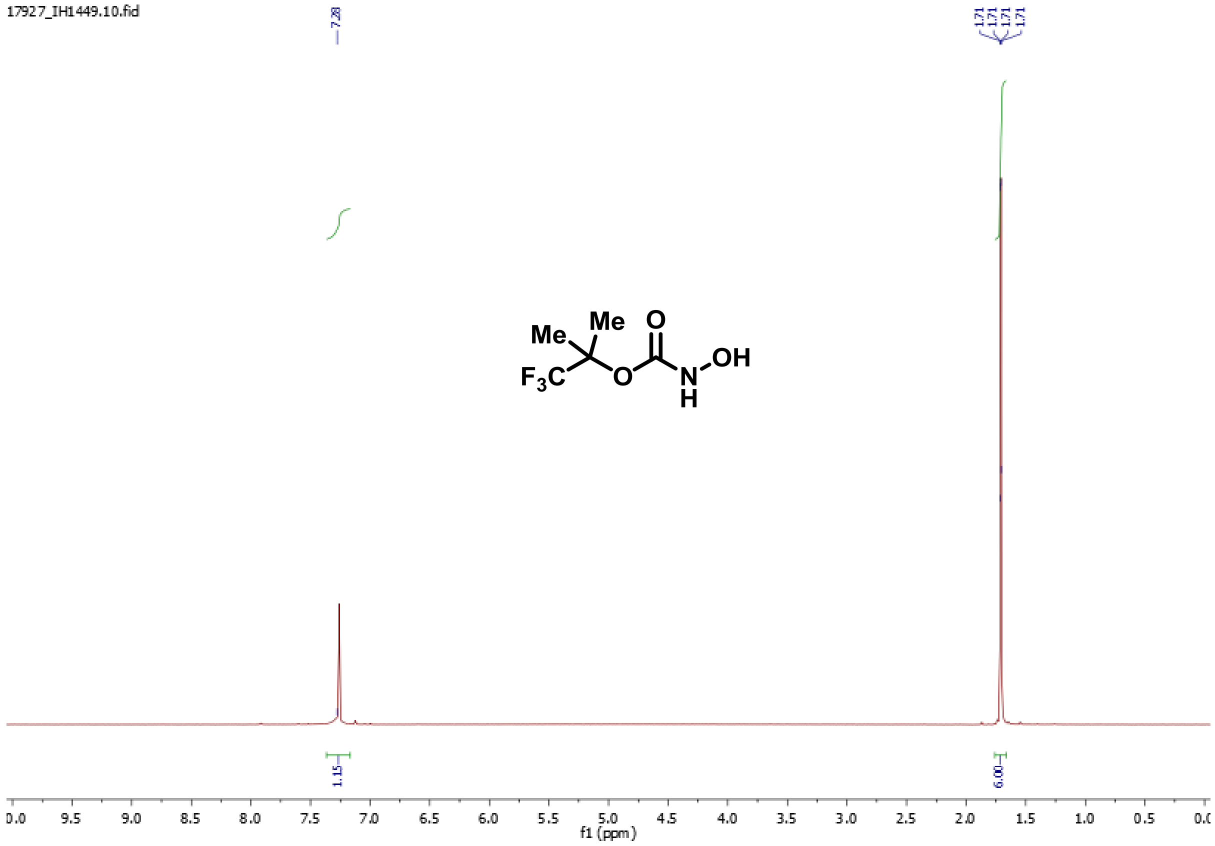
17927_IH1449.10.fid
7.28
1.71
1.71
1.71
1.71
Me
Me
O
F3C
O
N
OH
H
1.15
6.00
f1 (ppm)

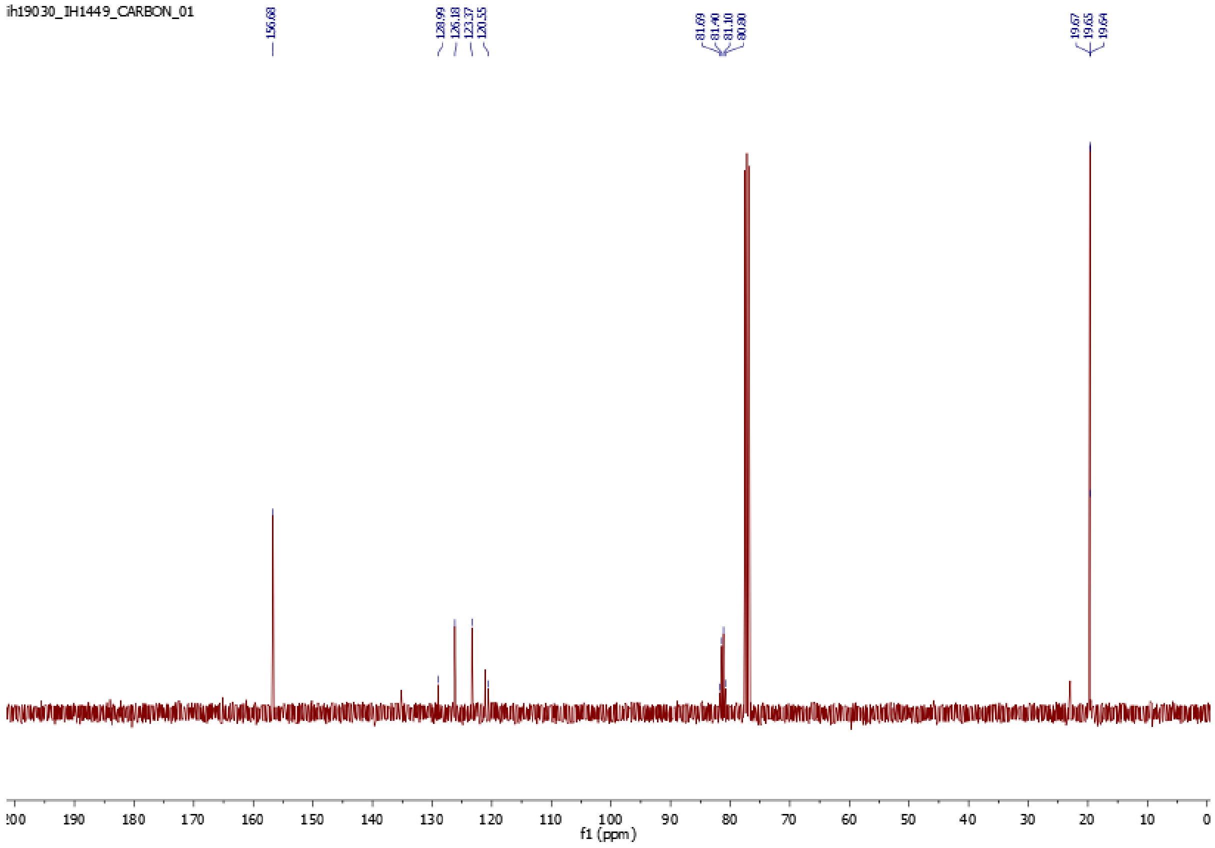
ih19030_IH1449_CARBON_01
156.68
128.99
126.18
123.37
120.55
81.69
81.40
81.10
80.80
19.67
19.65
19.64
f1 (ppm)

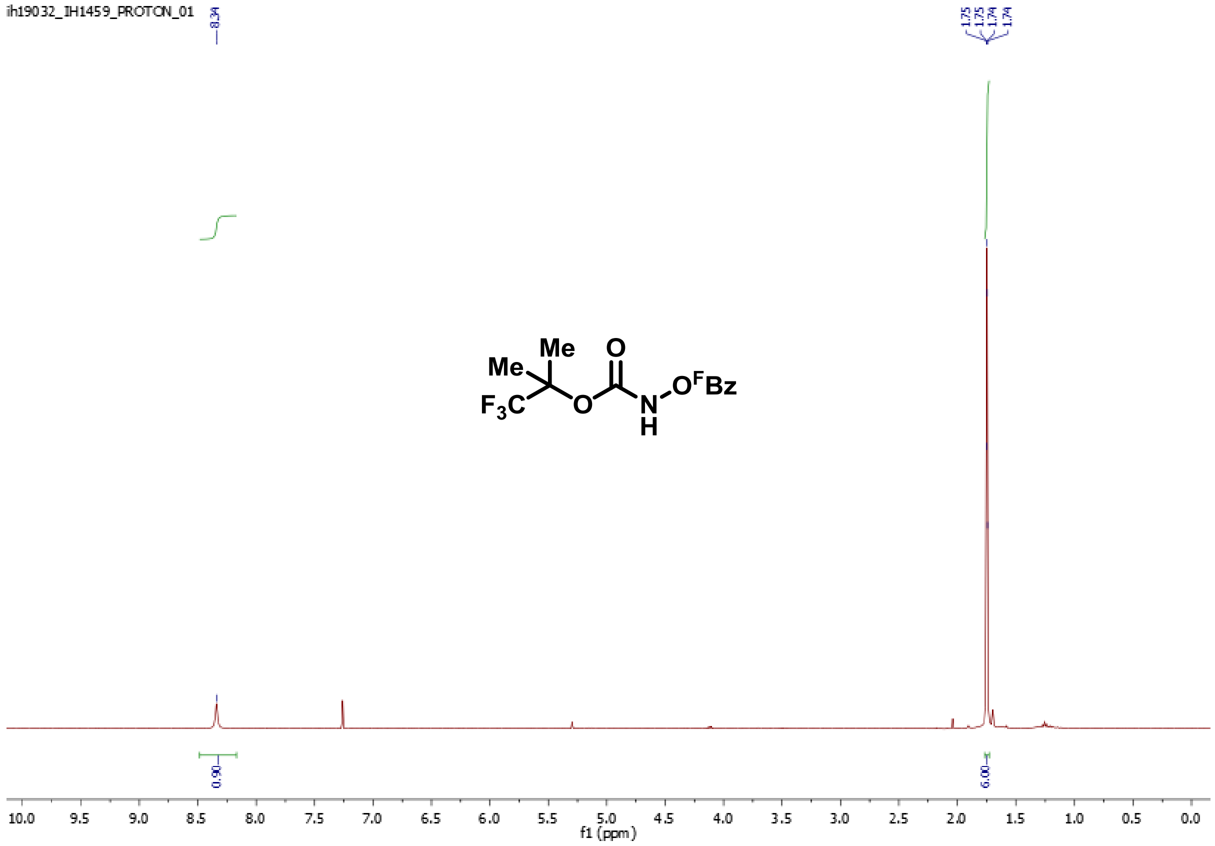
ih19032_IH1459_PROTON_01
8.34
1.75
1.75
1.74
1.74
Me
Me
O
F3C
O
N
H
OFBz
0.90
6.00
10.0 9.5 9.0 8.5 8.0 7.5 7.0 6.5 6.0 5.5 5.0 4.5 4.0 3.5 3.0 2.5 2.0 1.5 1.0 0.5 0.0
f1 (ppm)

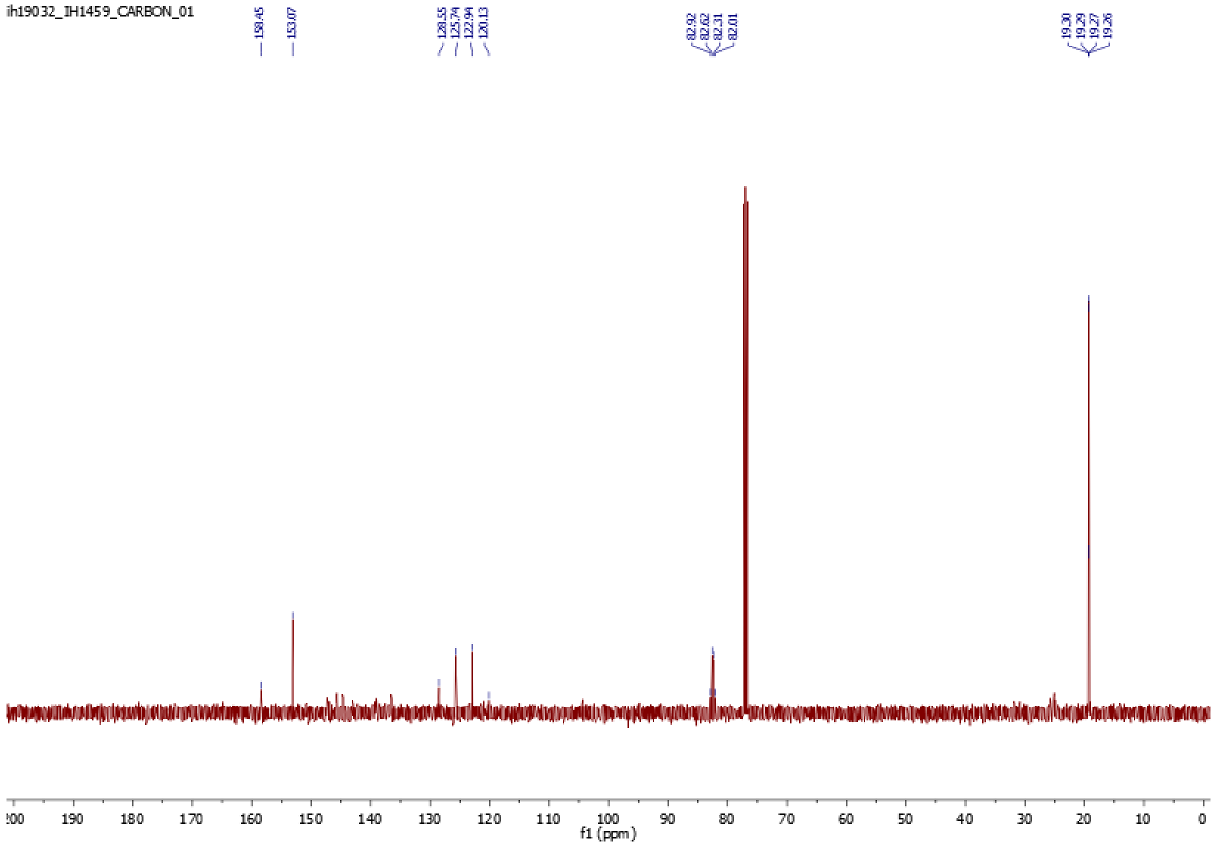
ih19032_IH1459_CARBON_01
158.45
153.07
128.55
125.74
122.94
120.13
82.92
82.62
82.31
82.01
19.30
19.29
19.27
19.26
200 190 180 170 160 150 140 130 120 110 100 90 80 70 60 50 40 30 20 10 0
f1 (ppm)

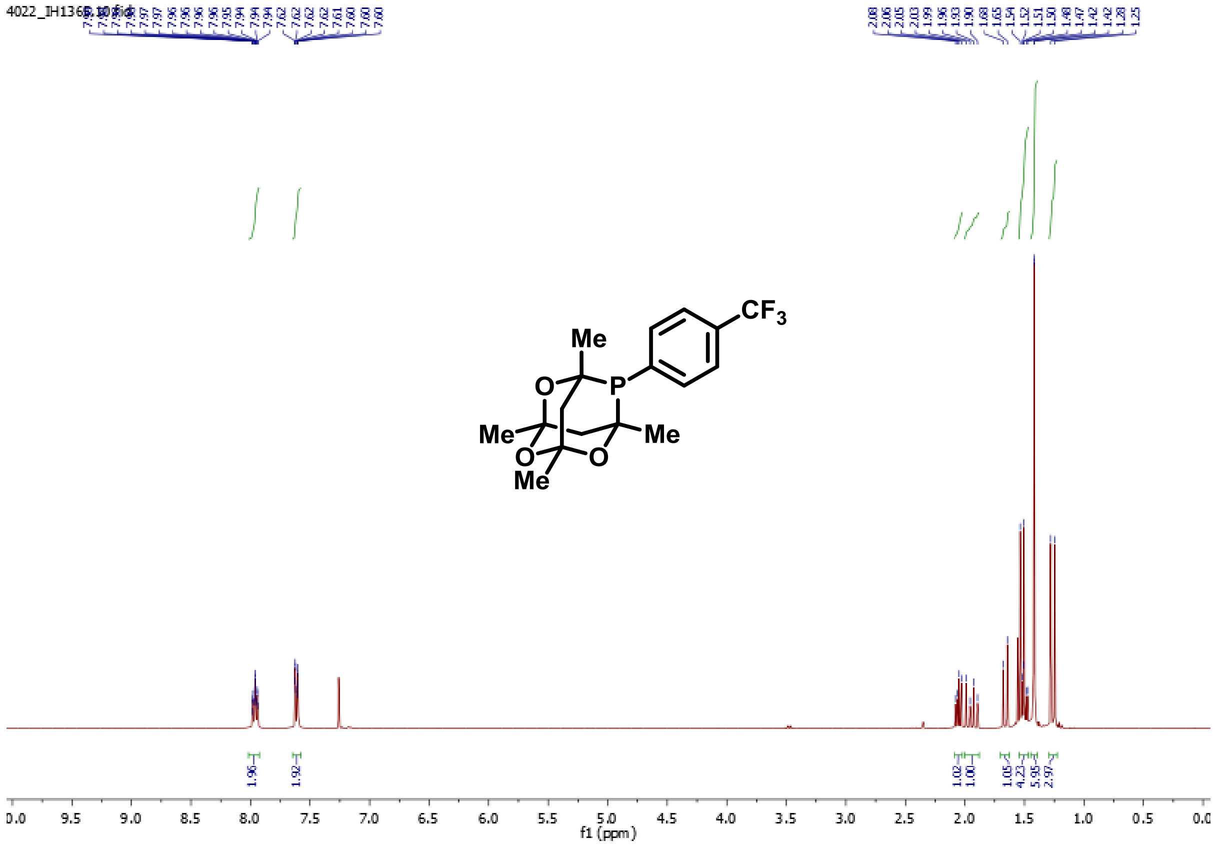

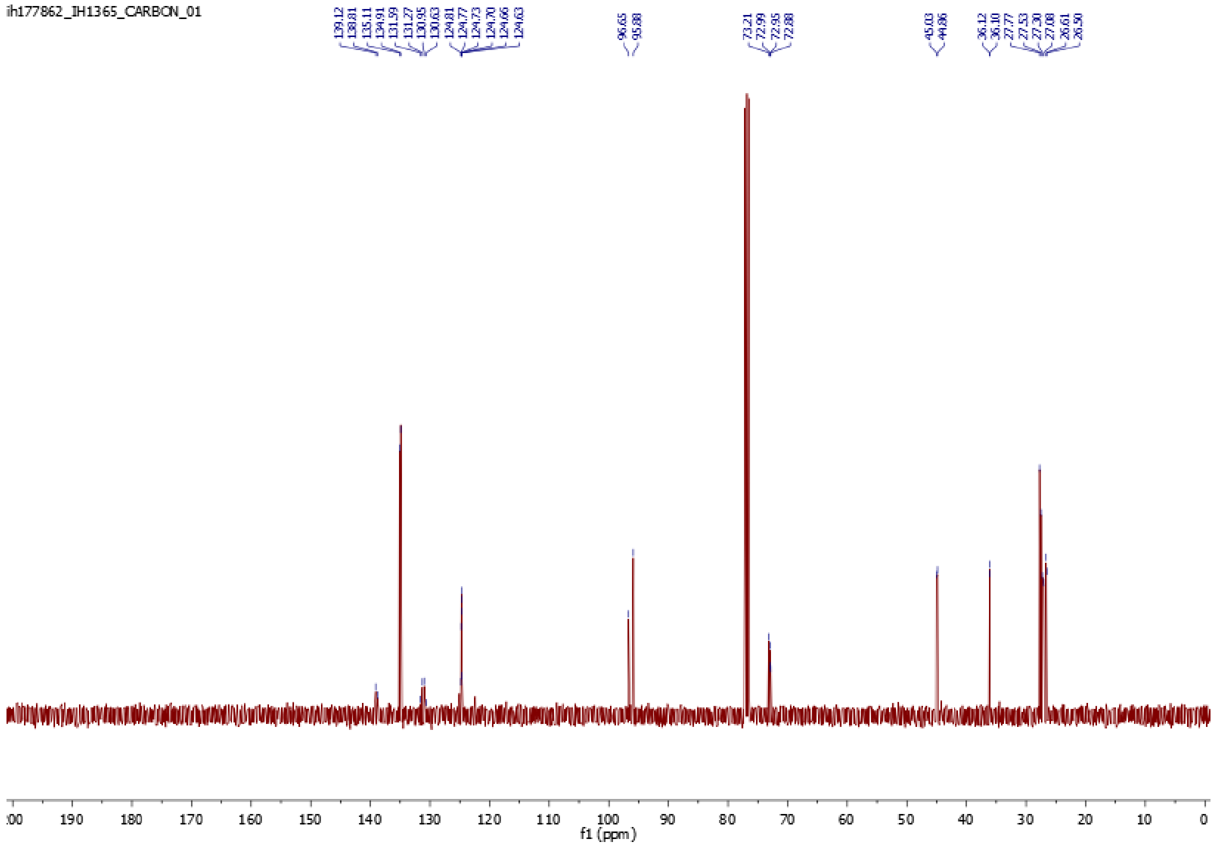

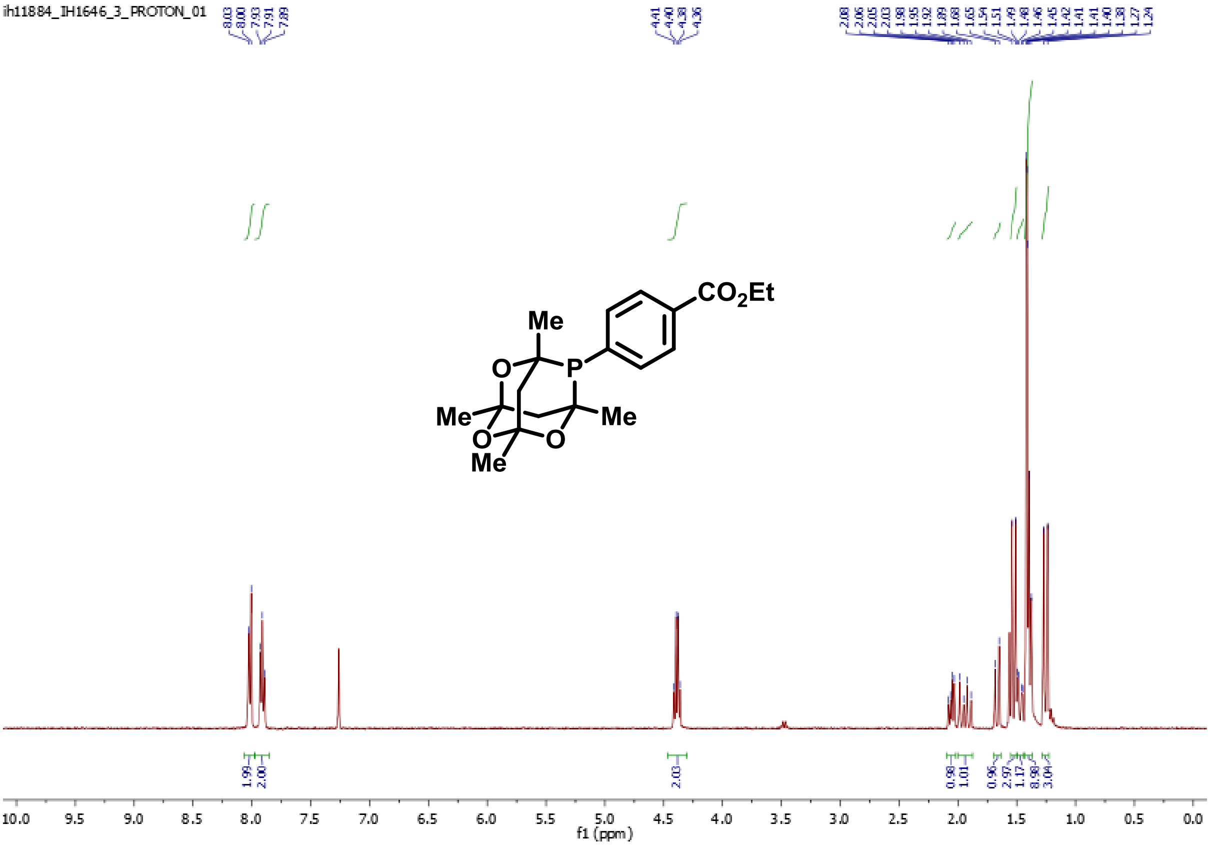
ih11884_IH1646_3_PROTON_01
CO2Et
Me
O
P
Me
Me
O
O
Me
f1 (ppm)

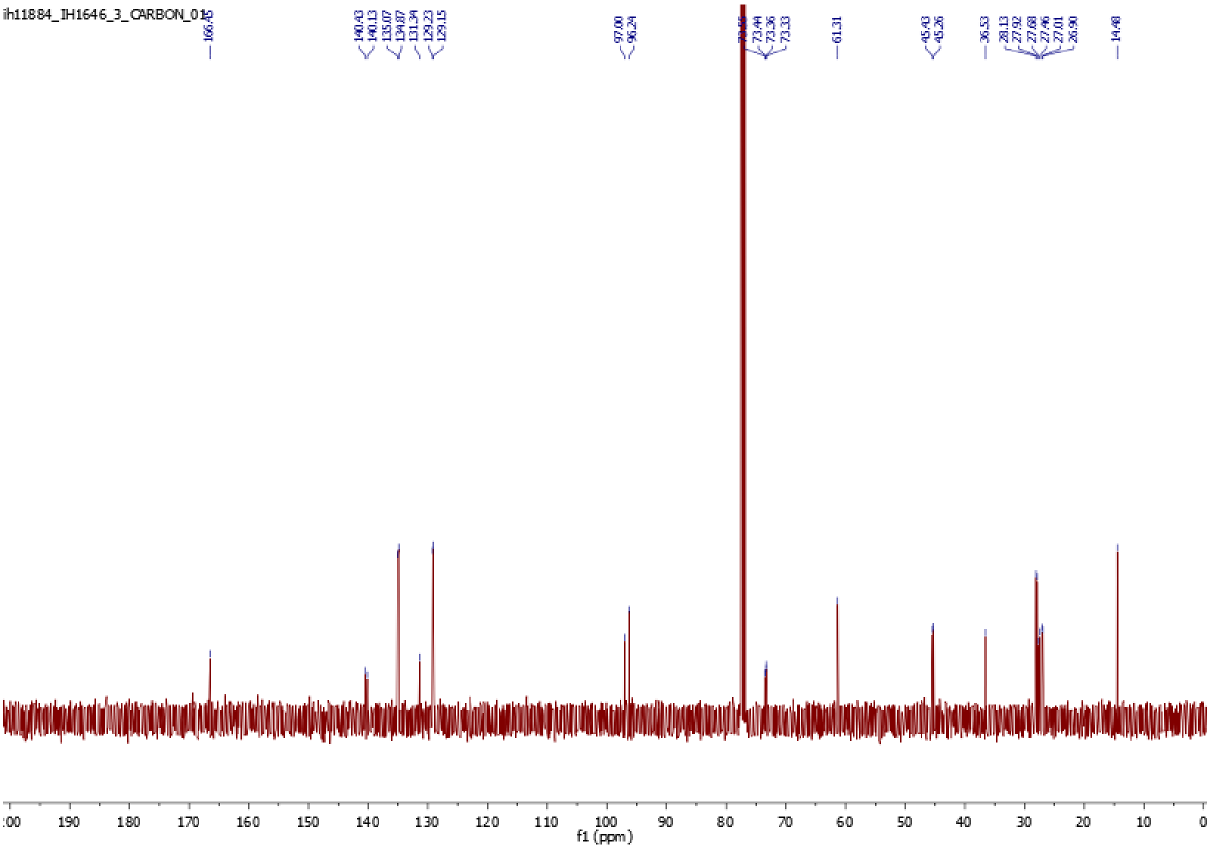
ih11884_IH1646_3_CARBON_01
f1 (ppm)

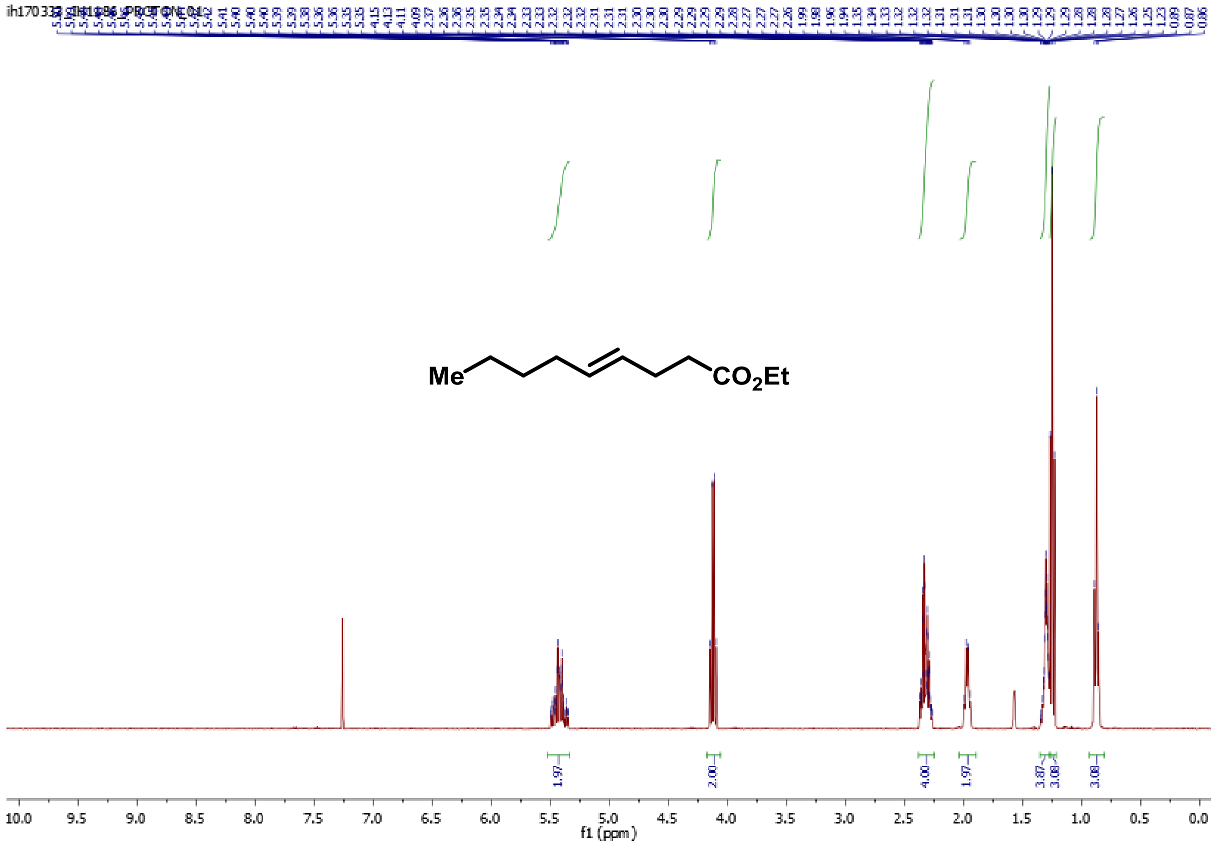

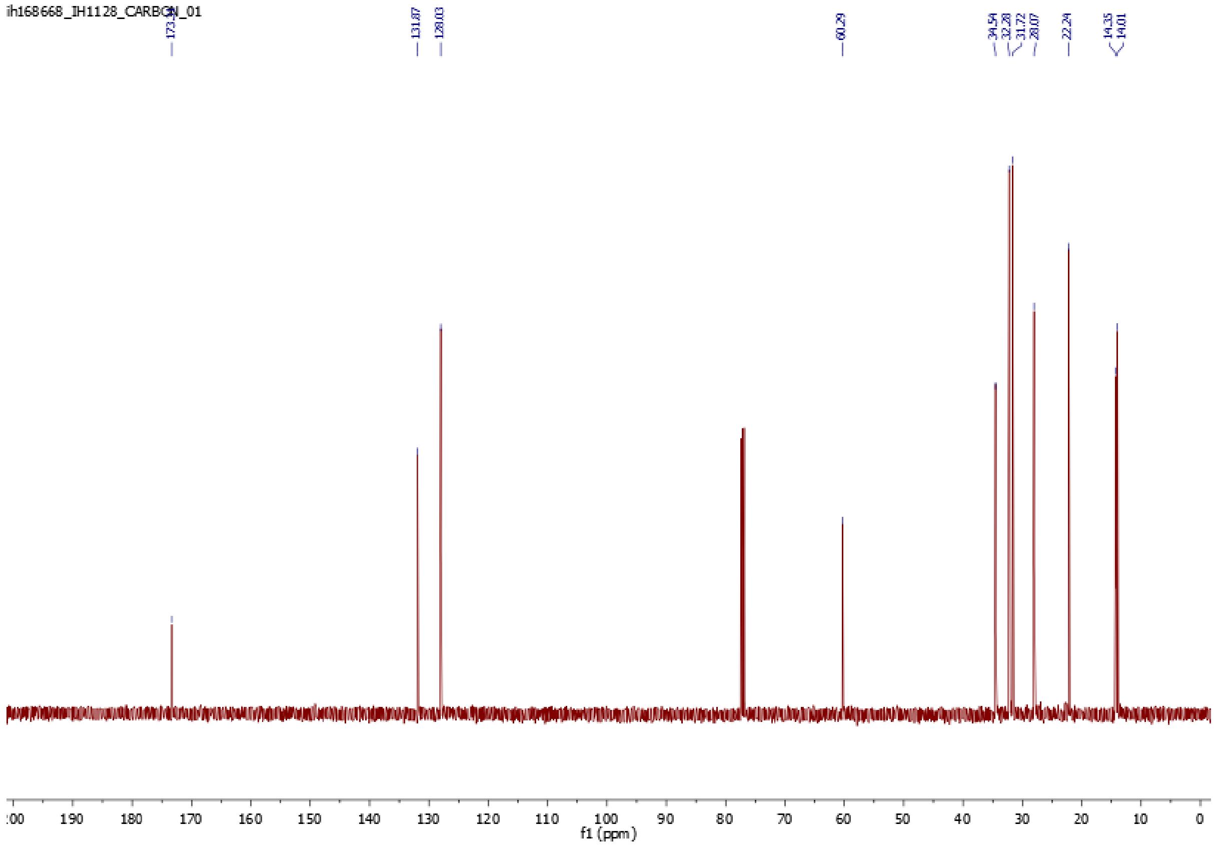

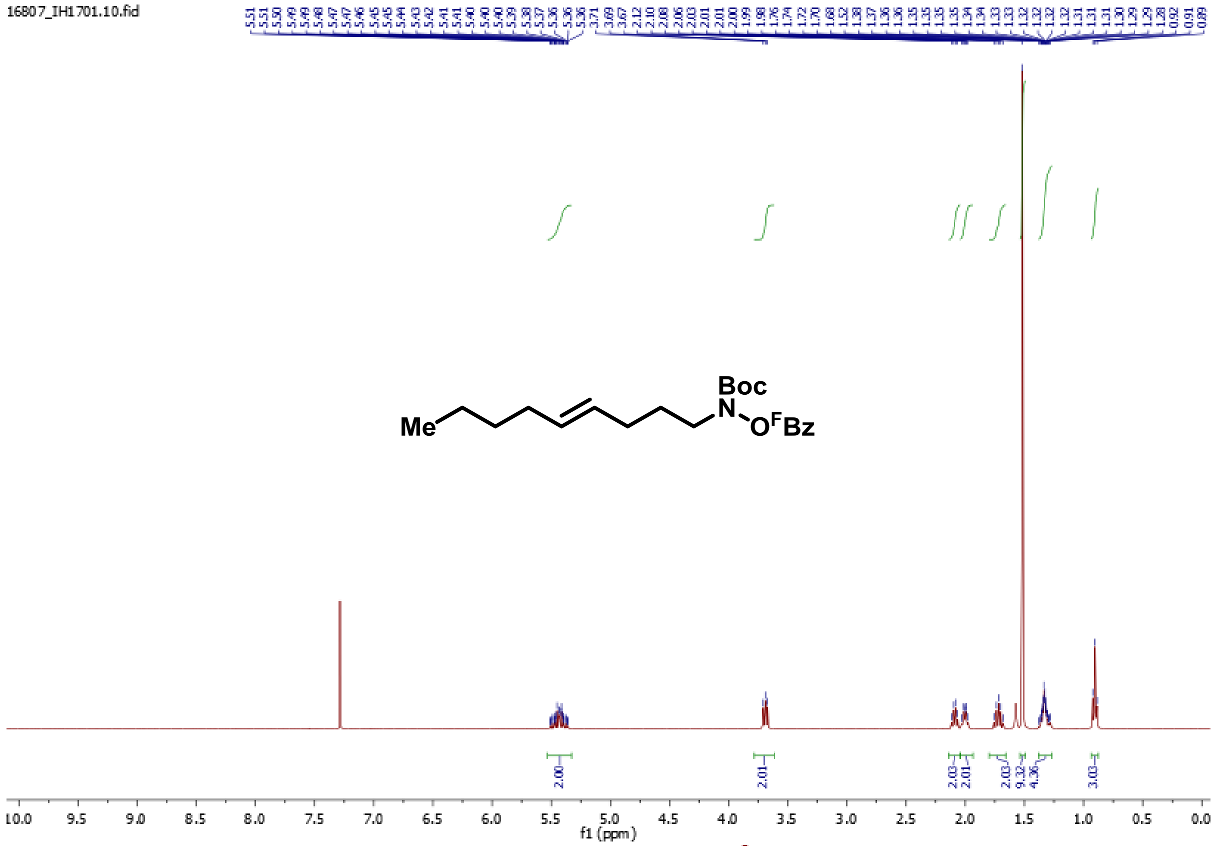
16807_IH1701.10.fid
Boc
N
O^FBz
Me
2.00
2.01
2.03
2.01
2.03
9.32
4.36
3.03
f1 (ppm)

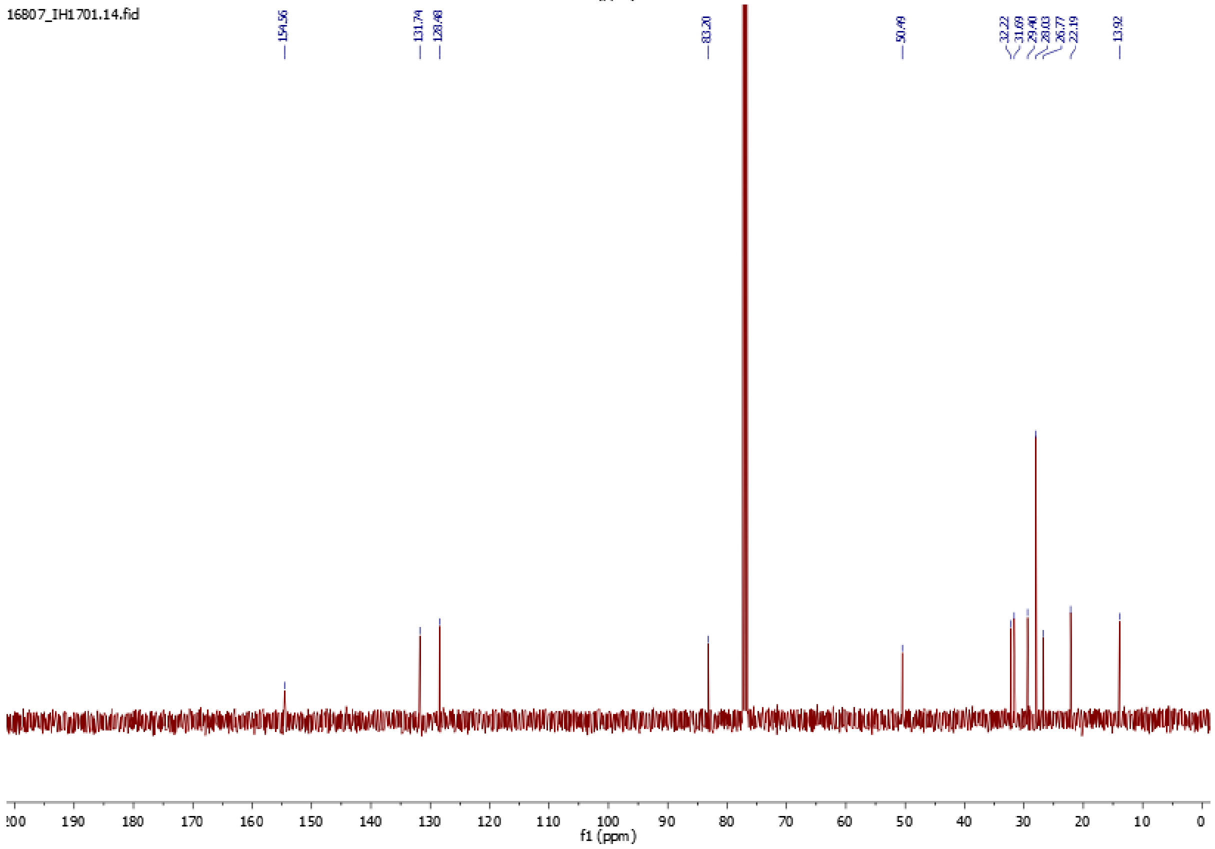
16807_IH1701.14.fid
154.56
131.74
128.48
83.20
50.49
32.22
31.69
29.40
28.03
26.77
22.19
13.92
f1 (ppm)

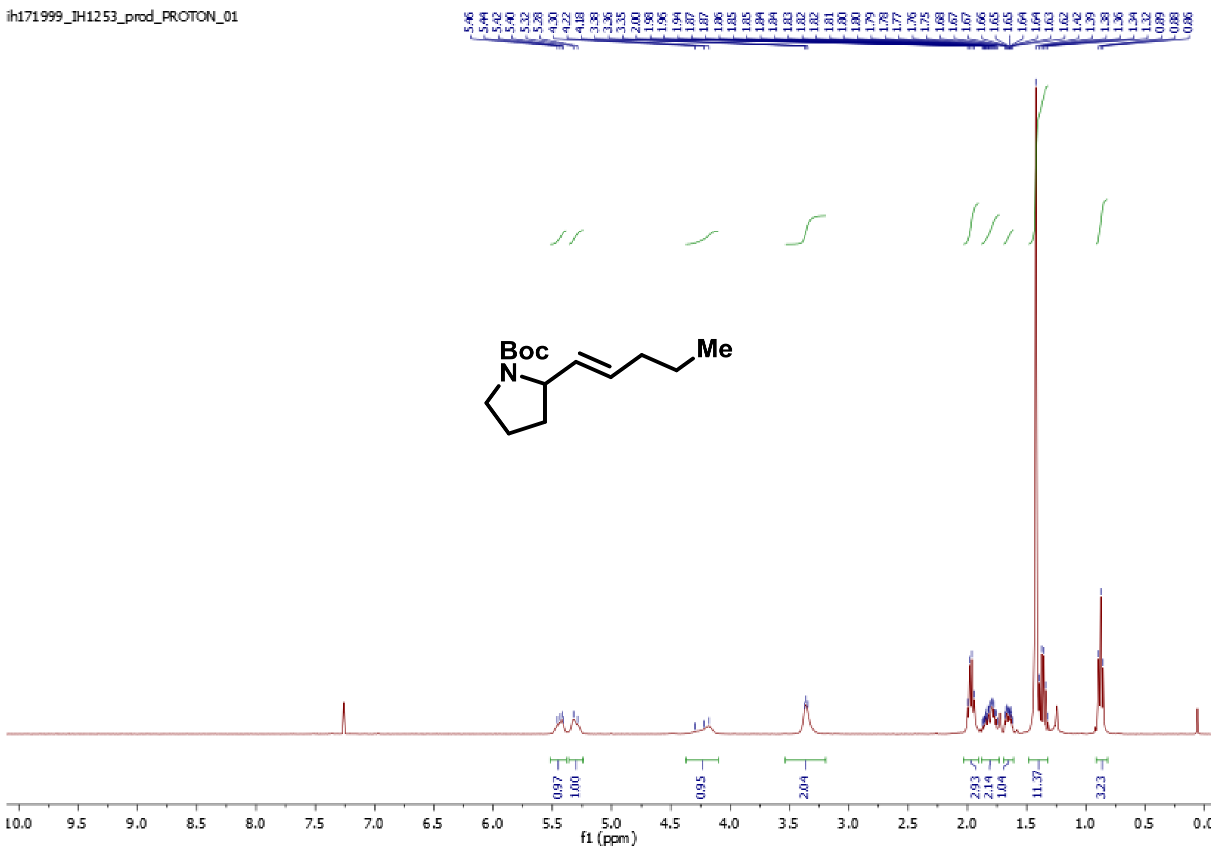
ih171999_IH1253_prod_PROTON_01
Boc
N
Me
f1 (ppm)

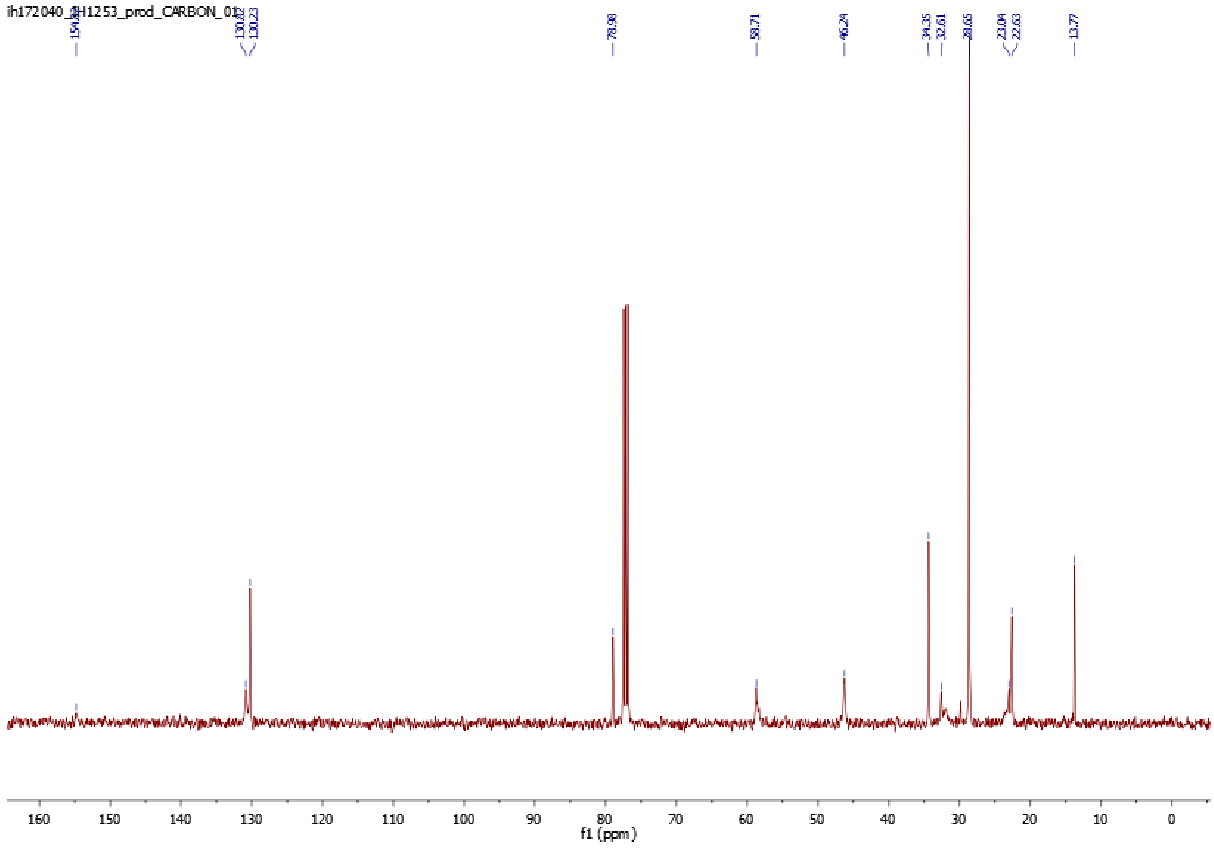
ih172040_IH1253_prod_CARBON_01
f1 (ppm)

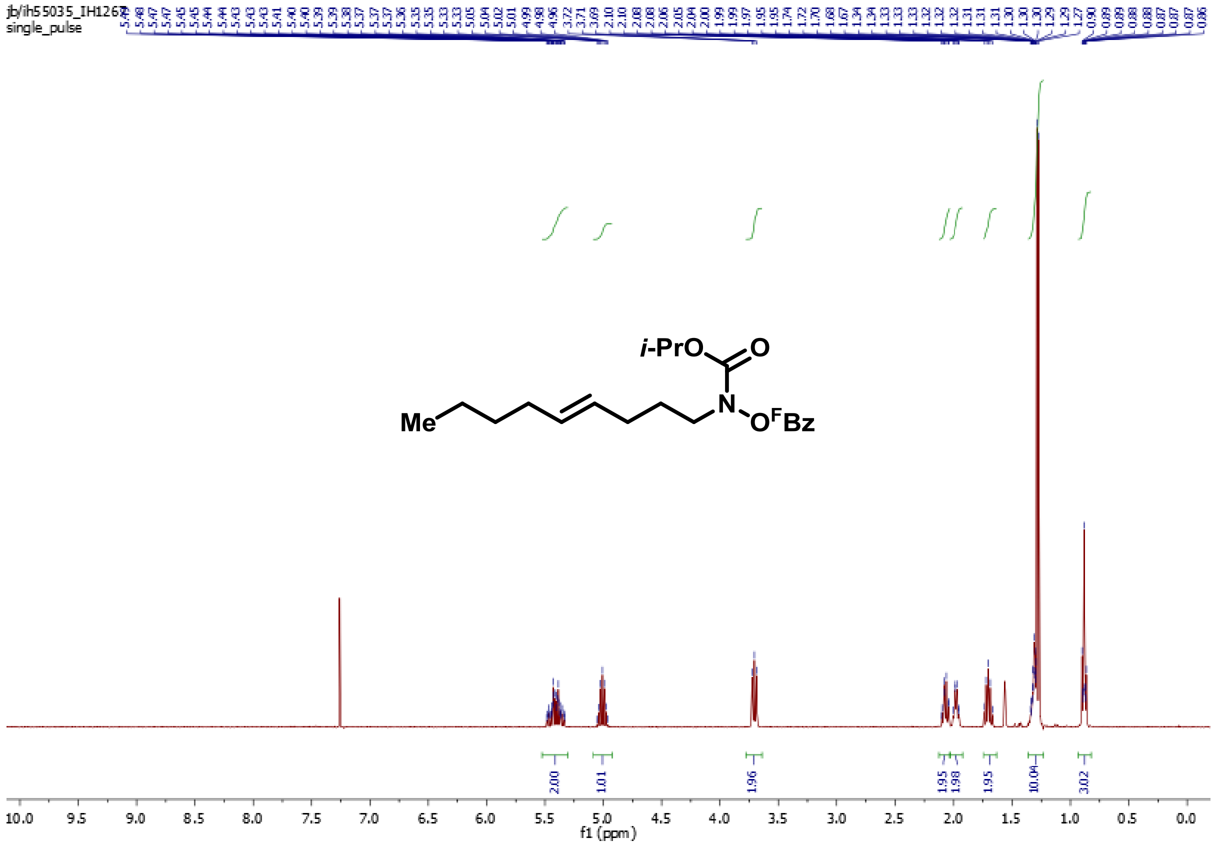
jb/ih55035_IH1267
single_pulse
i-PrO
O
N
Me
O^FBz
2.00
1.01
1.96
1.95
1.98
1.95
10.04
3.02
10.0 9.5 9.0 8.5 8.0 7.5 7.0 6.5 6.0 5.5 5.0 4.5 4.0 3.5 3.0 2.5 2.0 1.5 1.0 0.5 0.0
f1 (ppm)

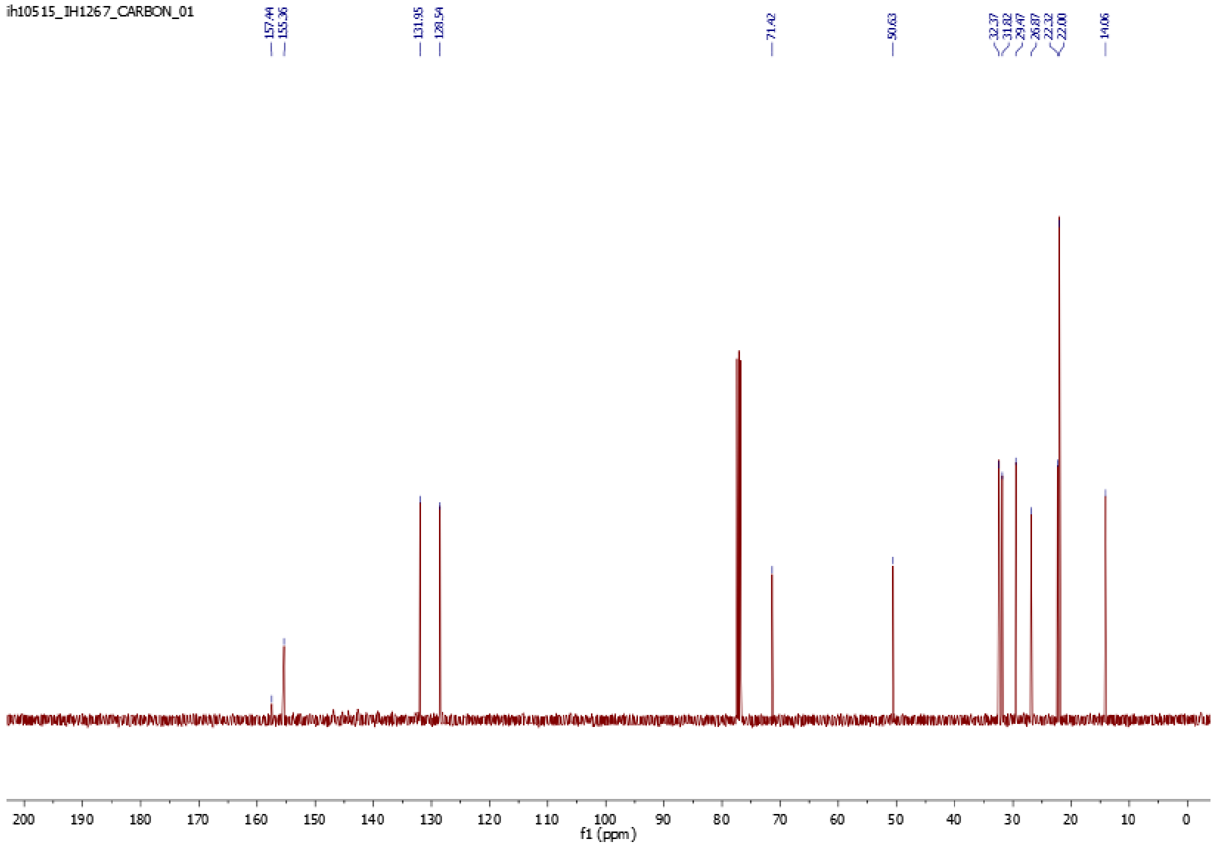
ih10515_IH1267_CARBON_01
157.44
155.36
131.95
128.54
71.42
50.63
32.37
31.82
29.47
26.87
22.32
22.00
14.06
200 190 180 170 160 150 140 130 120 110 100 90 80 70 60 50 40 30 20 10 0
f1 (ppm)

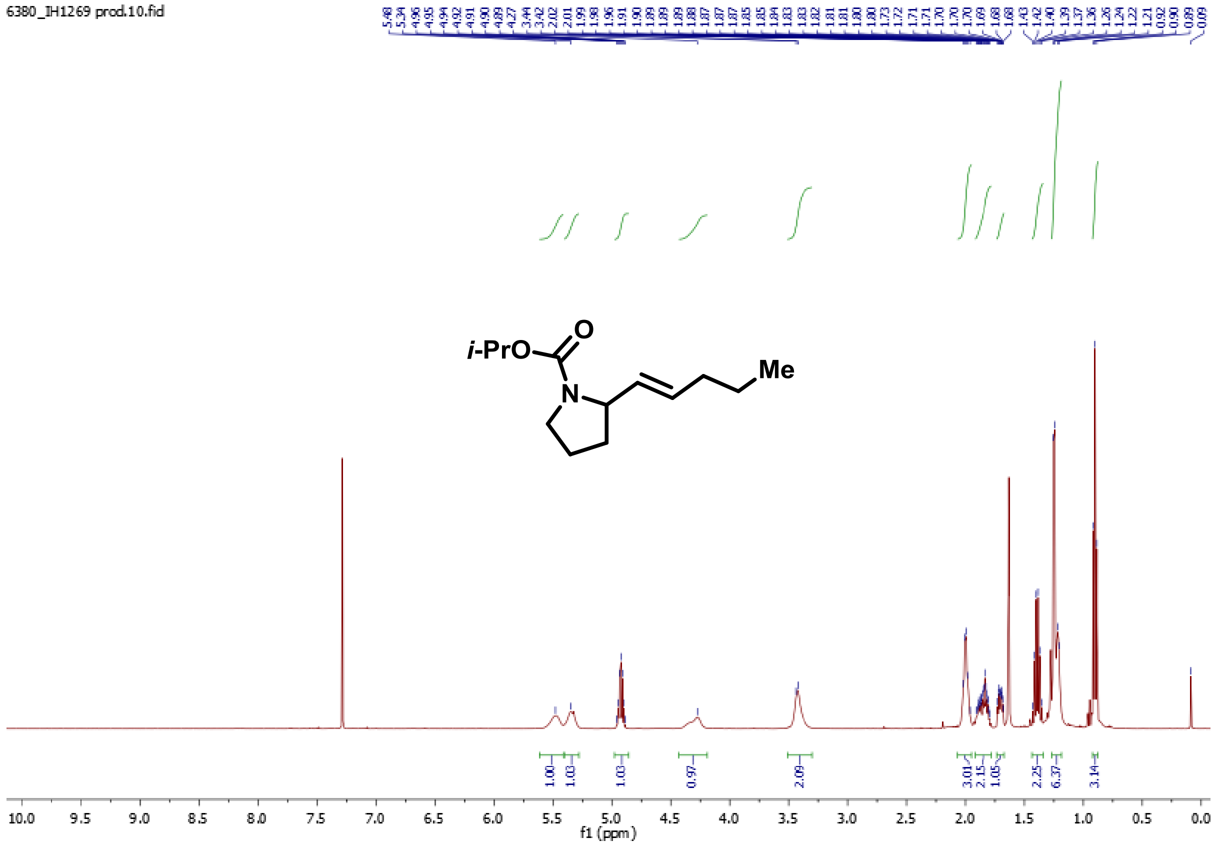
6380_IH1269 prod.10.fid
5.48 5.34 4.96 4.95 4.94 4.92 4.91 4.90 4.89 4.27 3.44 3.42 2.02 2.01 1.99 1.98 1.96 1.91 1.90 1.89 1.89 1.89 1.88 1.87 1.87 1.87 1.85 1.85 1.84 1.83 1.83 1.83 1.82 1.81 1.81 1.80 1.80 1.73 1.72 1.71 1.71 1.70 1.70 1.70 1.69 1.68 1.68 1.43 1.42 1.40 1.39 1.37 1.36 1.26 1.24 1.22 1.21 0.92 0.90 0.89 0.09
i-PrO
O
N
Me
1.00
1.03
1.03
0.97
2.09
3.01
2.15
1.05
2.25
6.37
3.14
10.0 9.5 9.0 8.5 8.0 7.5 7.0 6.5 6.0 5.5 5.0 4.5 4.0 3.5 3.0 2.5 2.0 1.5 1.0 0.5 0.0
f1 (ppm)

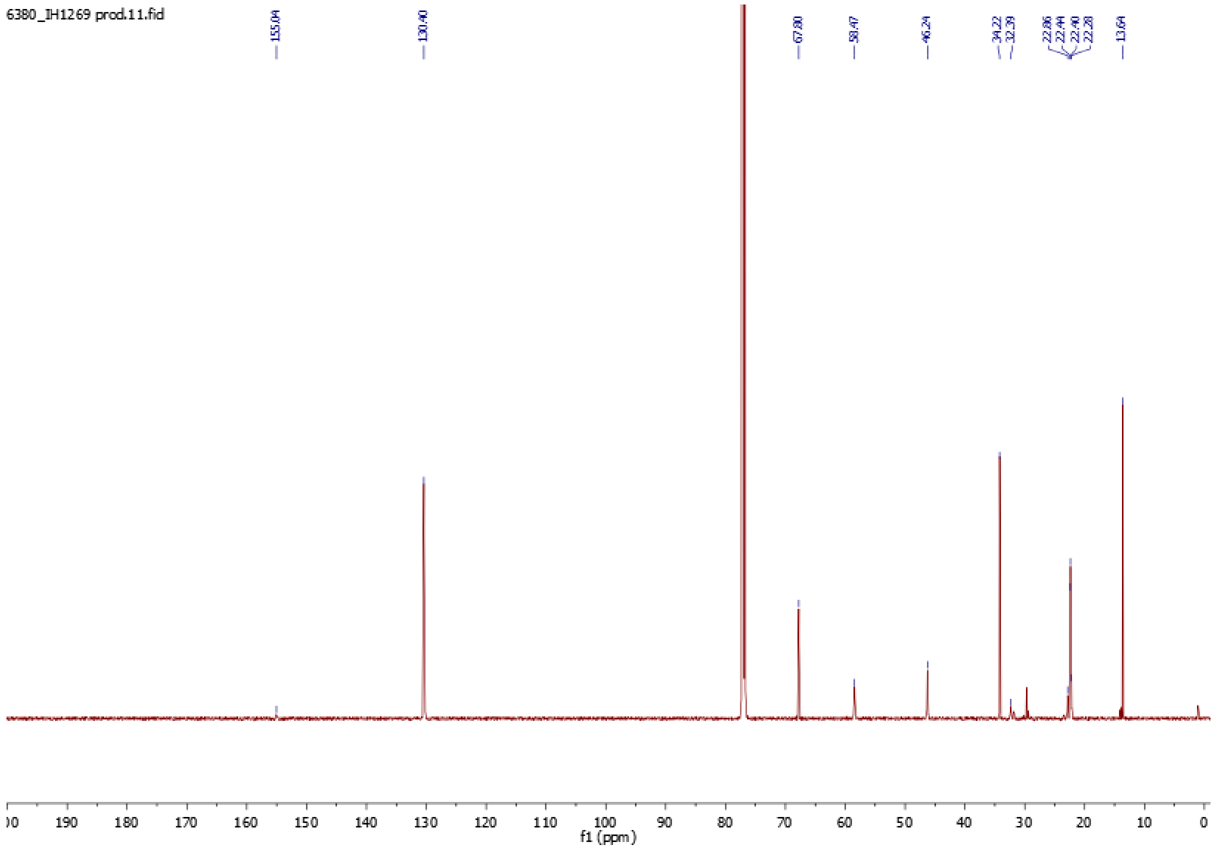
6380_IH1269 prod.11.fid
155.04
130.40
67.80
58.47
46.24
34.22
32.39
22.86
22.44
22.40
22.28
13.64
190 180 170 160 150 140 130 120 110 100 90 80 70 60 50 40 30 20 10 0
f1 (ppm)

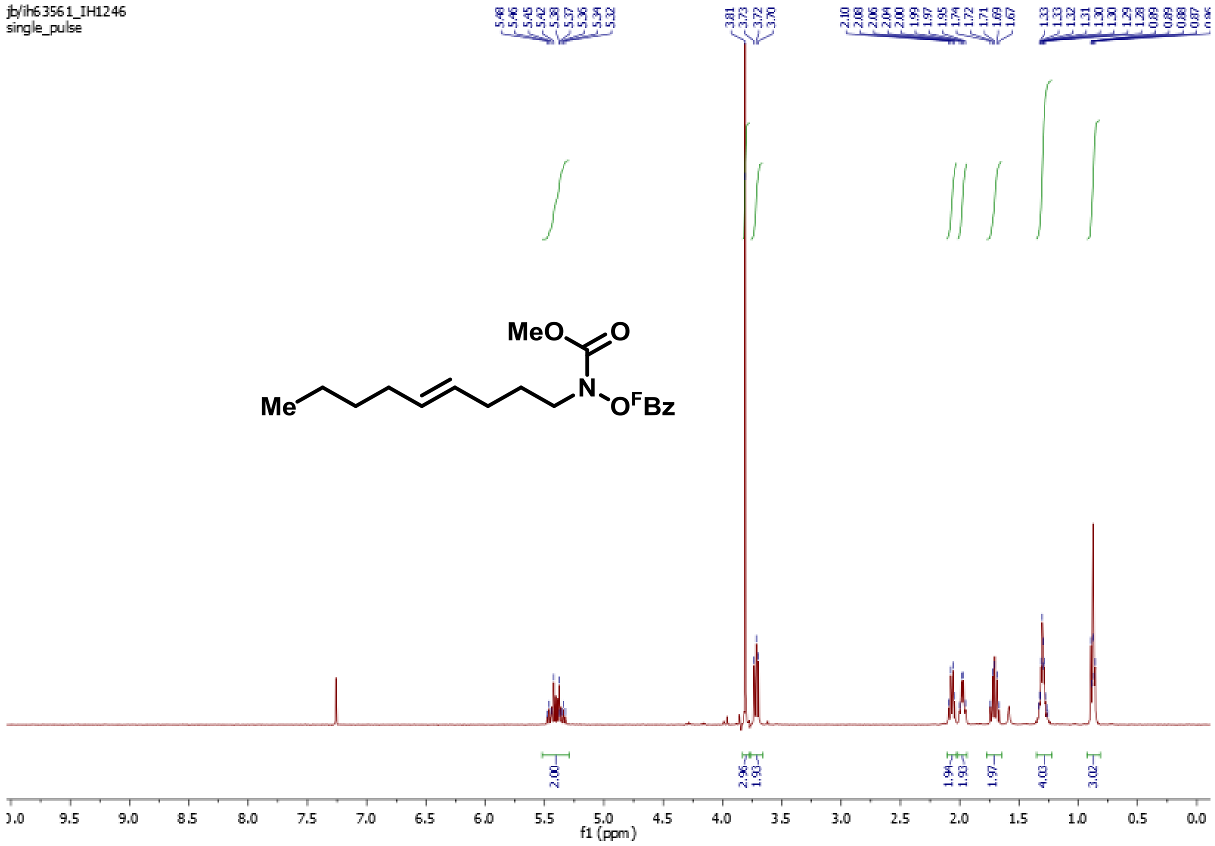
jb/ih63561_IH1246
single_pulse
MeO
O
N
O^FBz
Me
f1 (ppm)

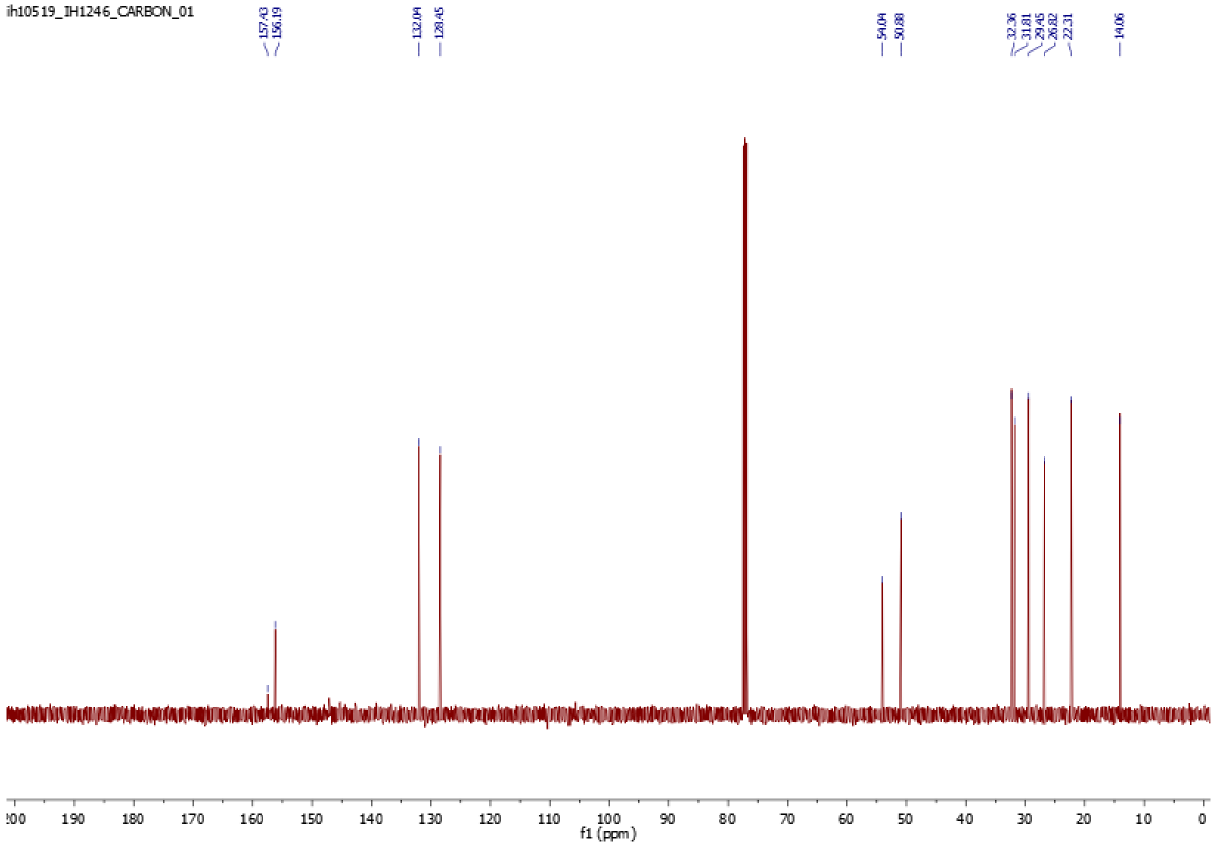
ih10519_IH1246_CARBON_01
157.43
156.19
132.04
128.45
54.04
50.88
32.36
31.81
29.45
26.82
22.31
14.06
f1 (ppm)

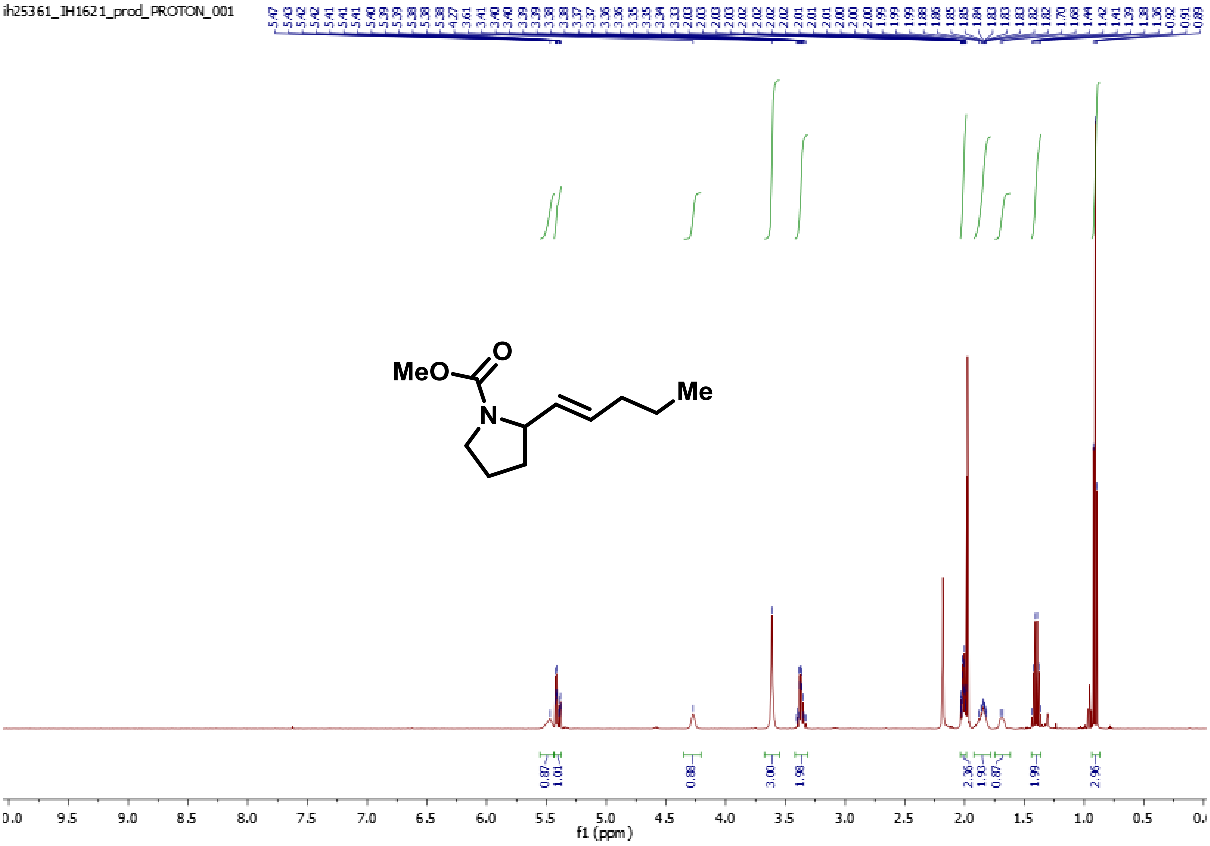
ih25361_IH1621_prod_PROTON_001
MeO
O
N
Me
0.87
1.01
0.88
3.00
1.98
2.36
1.93
0.87
1.99
2.96
f1 (ppm)

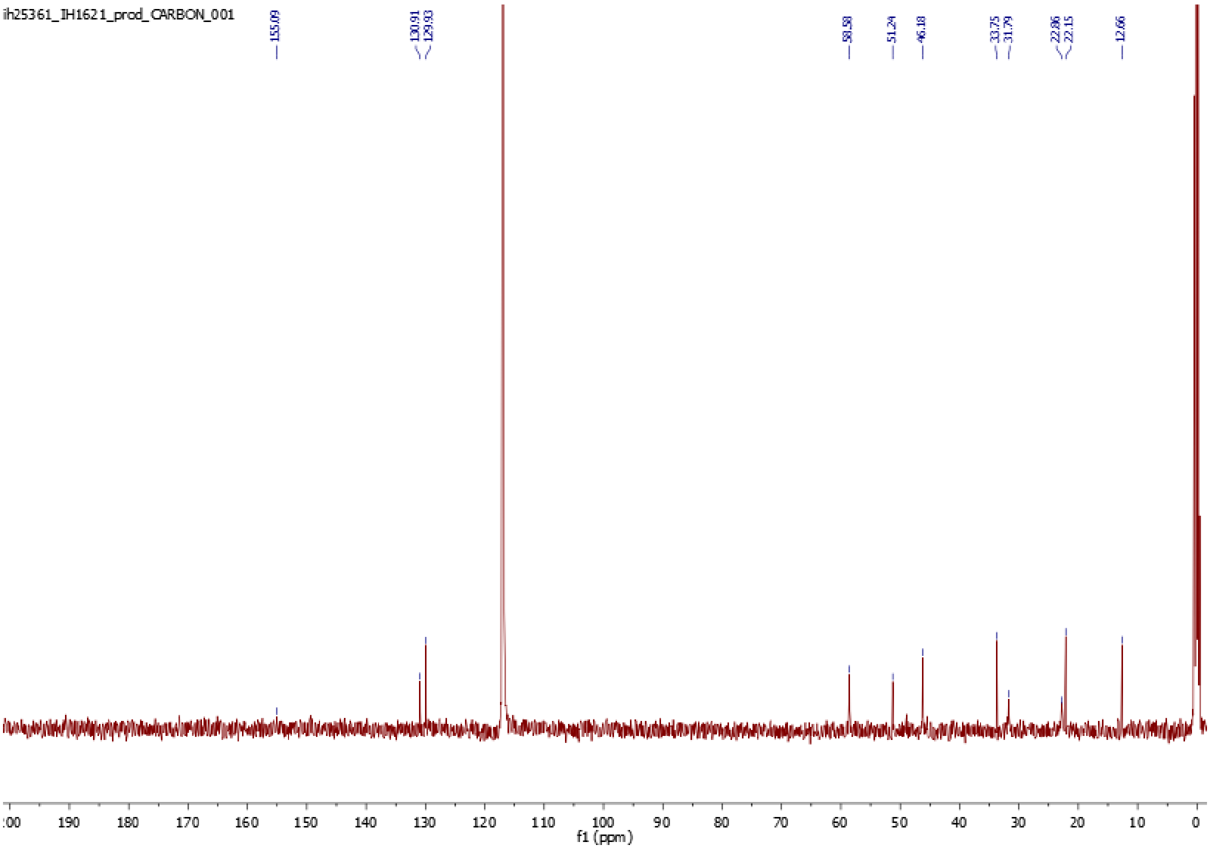
ih25361_IH1621_prod_CARBON_001
155.09
130.91
129.93
58.58
51.24
46.18
33.75
31.79
22.86
22.15
12.66
f1 (ppm)

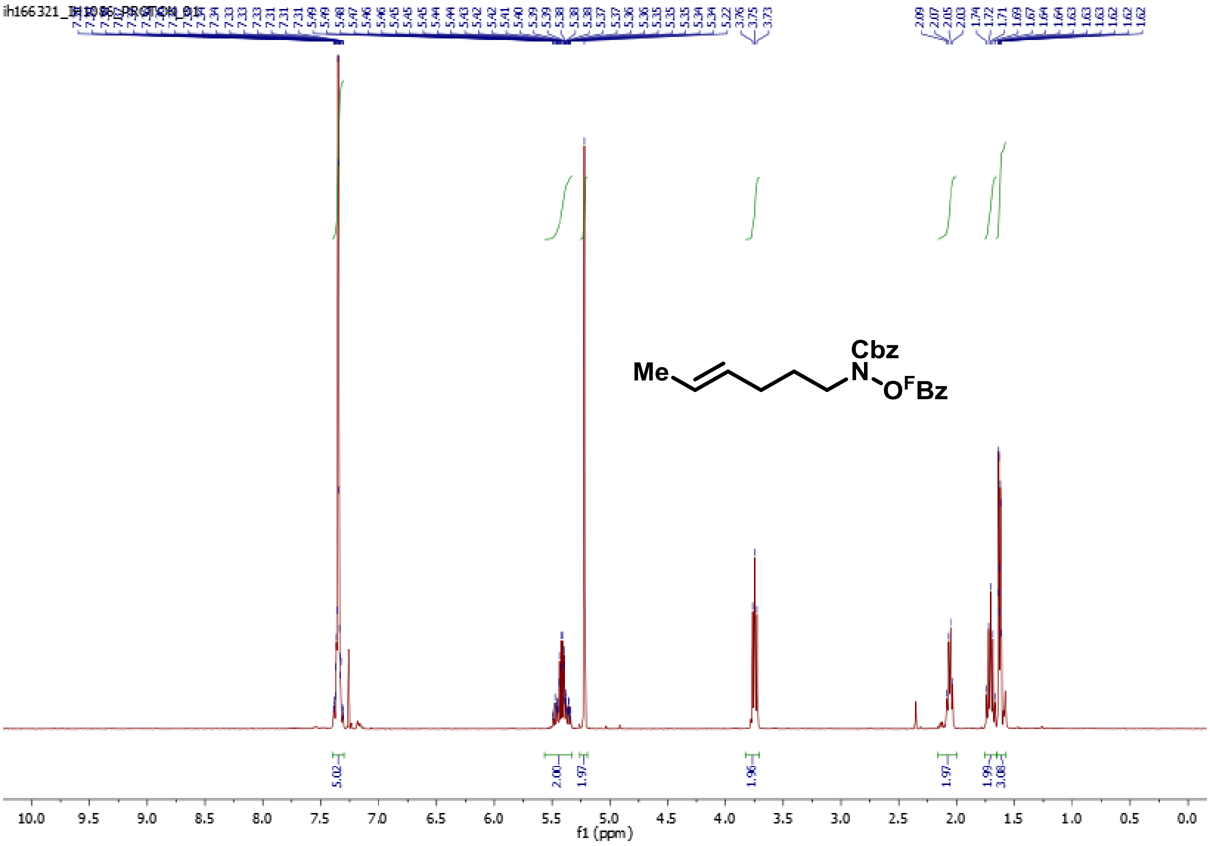
Me
Cbz
N
O^F Bz
5.02
2.00
1.97
1.96
1.97
1.99
3.08
10.0 9.5 9.0 8.5 8.0 7.5 7.0 6.5 6.0 5.5 5.0 4.5 4.0 3.5 3.0 2.5 2.0 1.5 1.0 0.5 0.0
f1 (ppm)

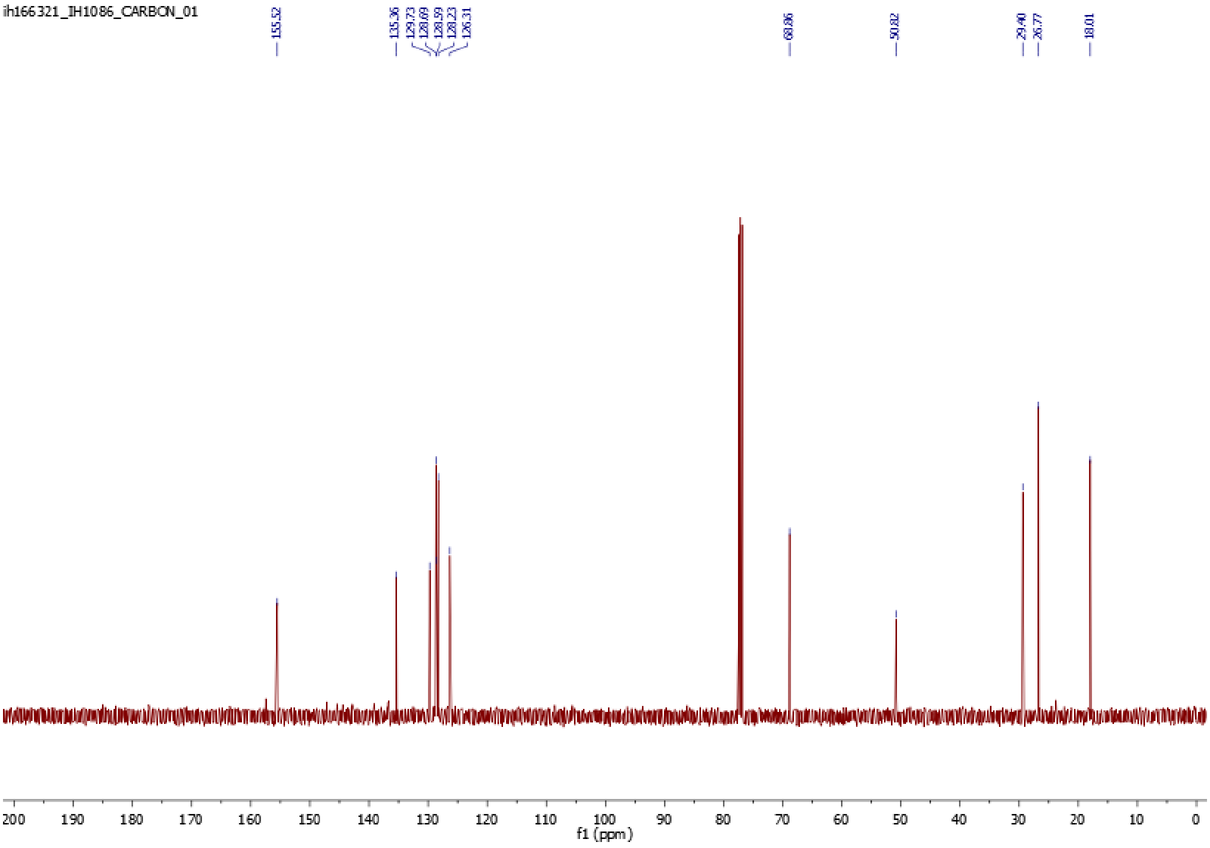
ih166321_IH1086_CARBON_01
155.52
135.36
129.73
128.69
128.59
128.23
126.31
68.86
50.82
29.40
26.77
18.01
200 190 180 170 160 150 140 130 120 110 100 90 80 70 60 50 40 30 20 10 0
f1 (ppm)

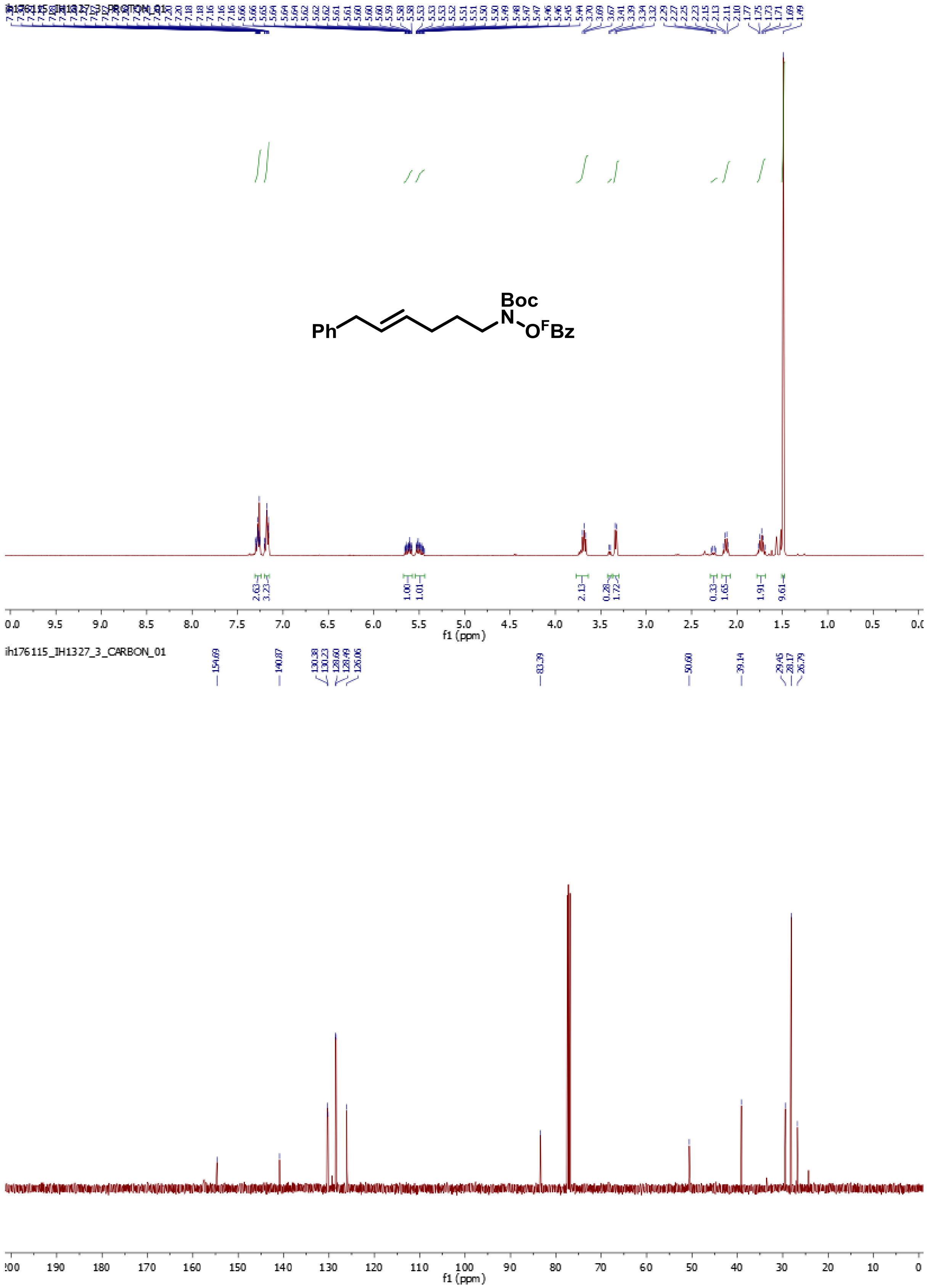

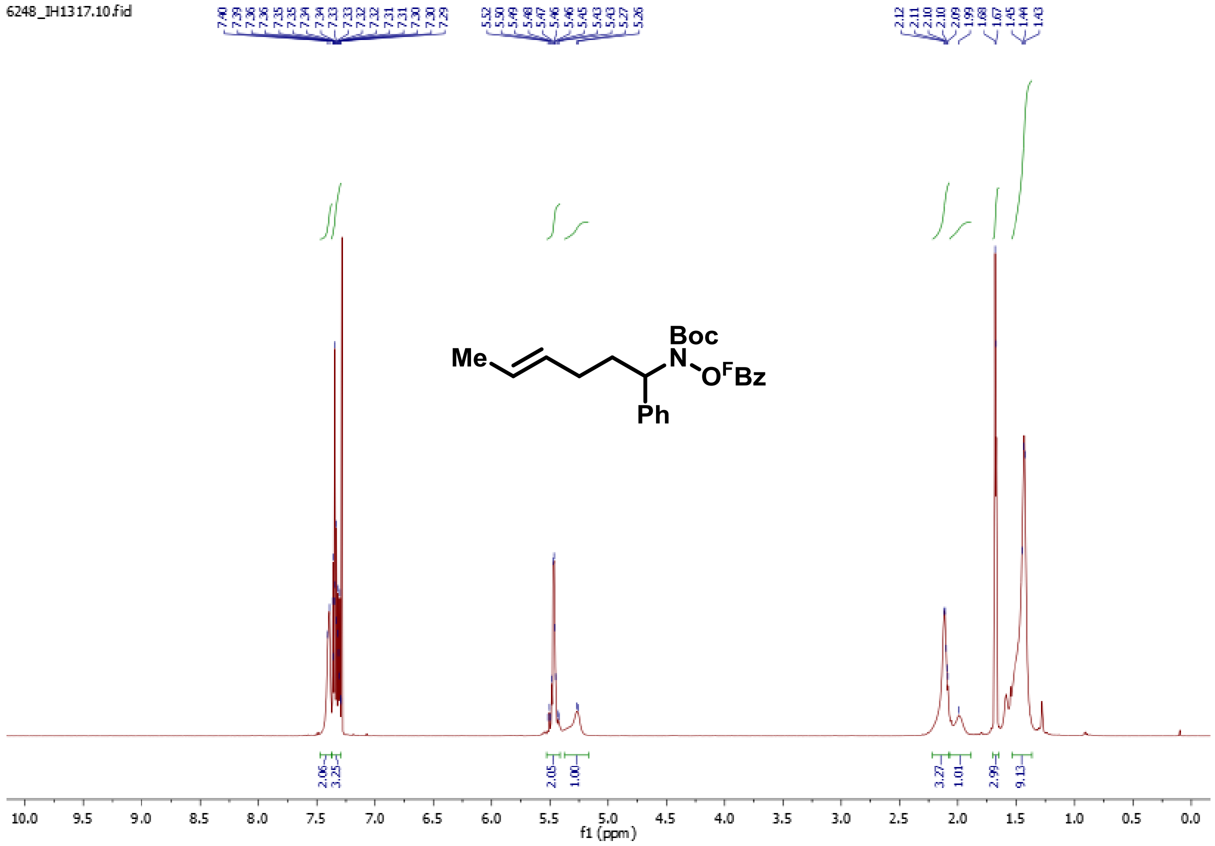
6248_IH1317.10.fid
7.40 7.39 7.36 7.36 7.35 7.35 7.34 7.34 7.33 7.33 7.32 7.32 7.31 7.31 7.30 7.30 7.29
5.52 5.50 5.49 5.48 5.47 5.46 5.46 5.45 5.43 5.43 5.27 5.26
2.12 2.11 2.10 2.10 2.09 1.99 1.68 1.67 1.45 1.44 1.43
Boc
N
O
F
Bz
Me
Ph
2.06 3.25 2.05 1.00 3.27 1.01 2.99 9.13
10.0 9.5 9.0 8.5 8.0 7.5 7.0 6.5 6.0 5.5 5.0 4.5 4.0 3.5 3.0 2.5 2.0 1.5 1.0 0.5 0.0
f1 (ppm)

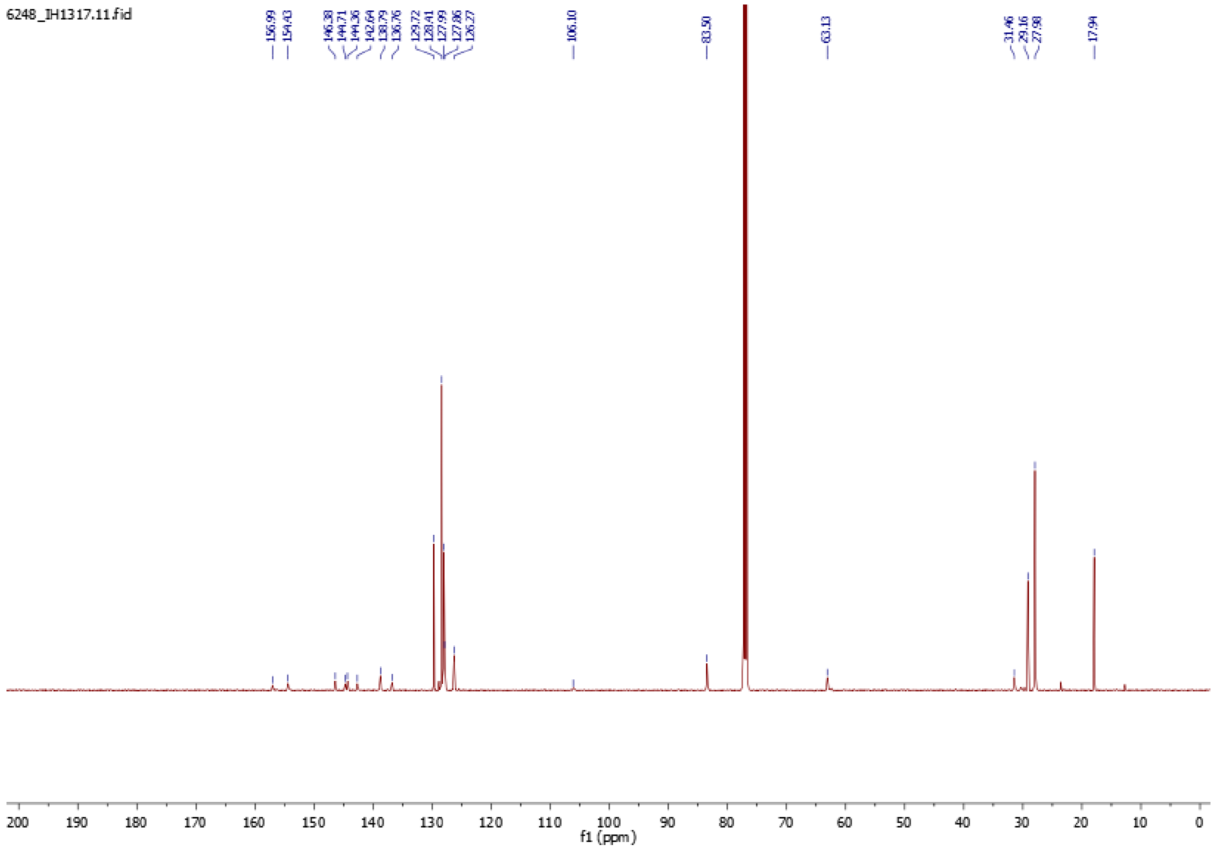
6248_IH1317.11.fid
156.99 154.43 146.38 144.71 144.36 142.64 138.79 136.76 129.72 128.41 127.99 127.86 126.27 106.10 83.50 63.13 31.46 29.16 27.98 17.94
200 190 180 170 160 150 140 130 120 110 100 90 80 70 60 50 40 30 20 10 0
f1 (ppm)

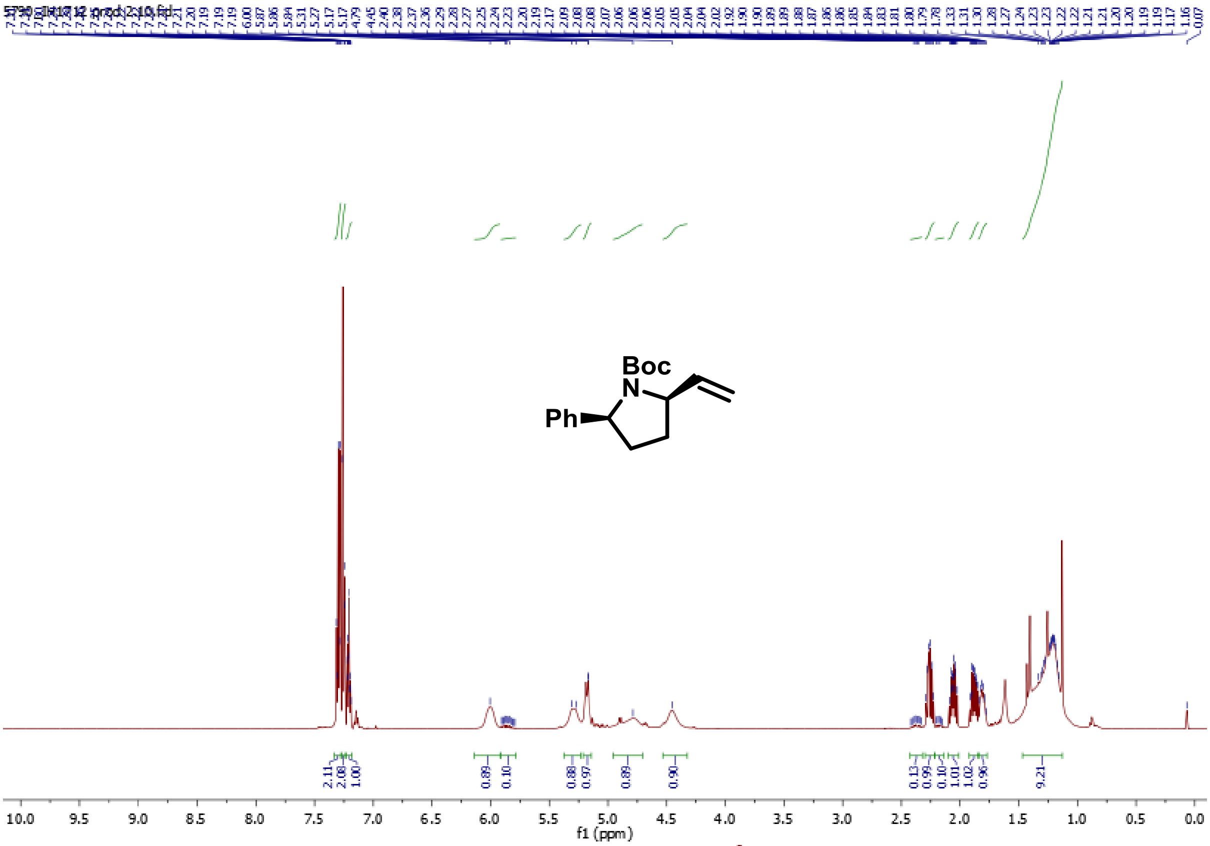
5790_IH1712 prod 2.10.fid
Boc
N
Ph
f1 (ppm)

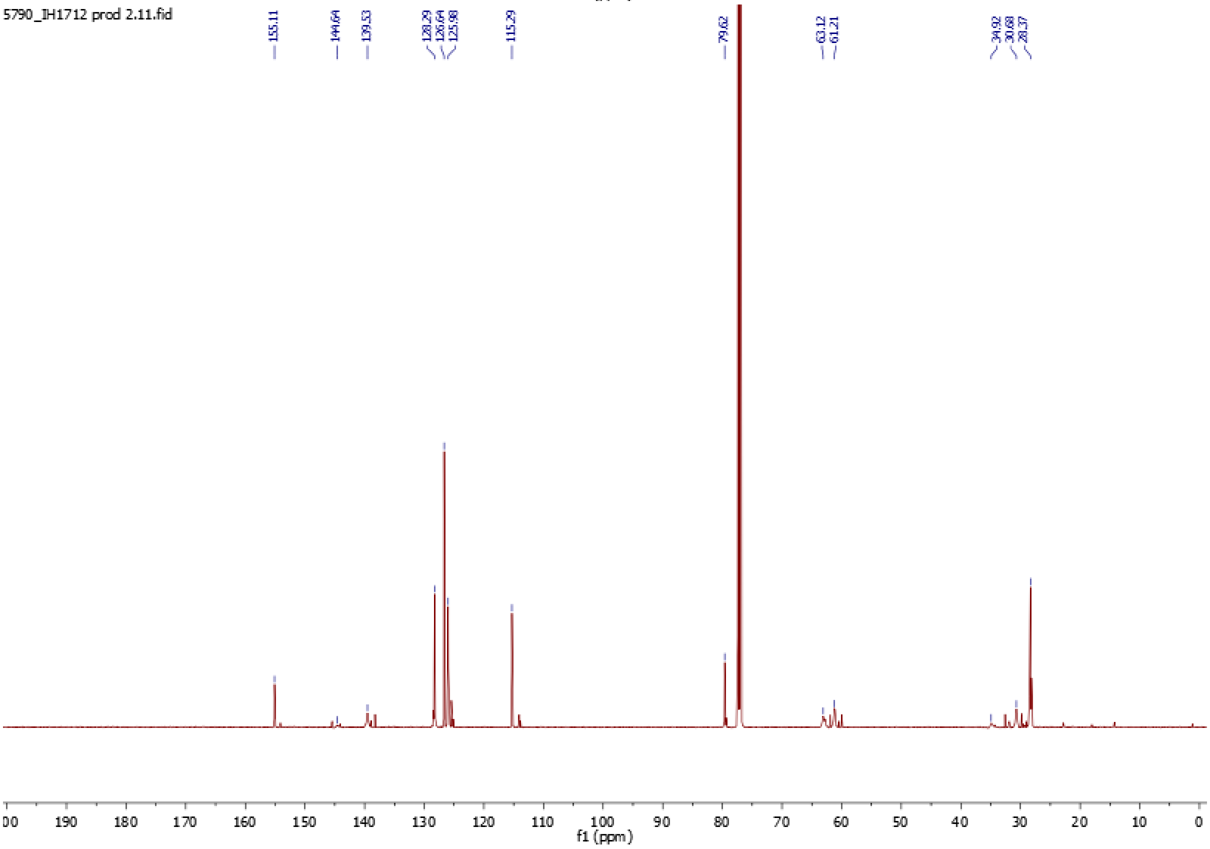
5790_IH1712 prod 2.11.fid
155.11
144.64
139.53
128.29
126.64
125.98
115.29
79.62
63.12
61.21
34.92
30.68
28.37
f1 (ppm)

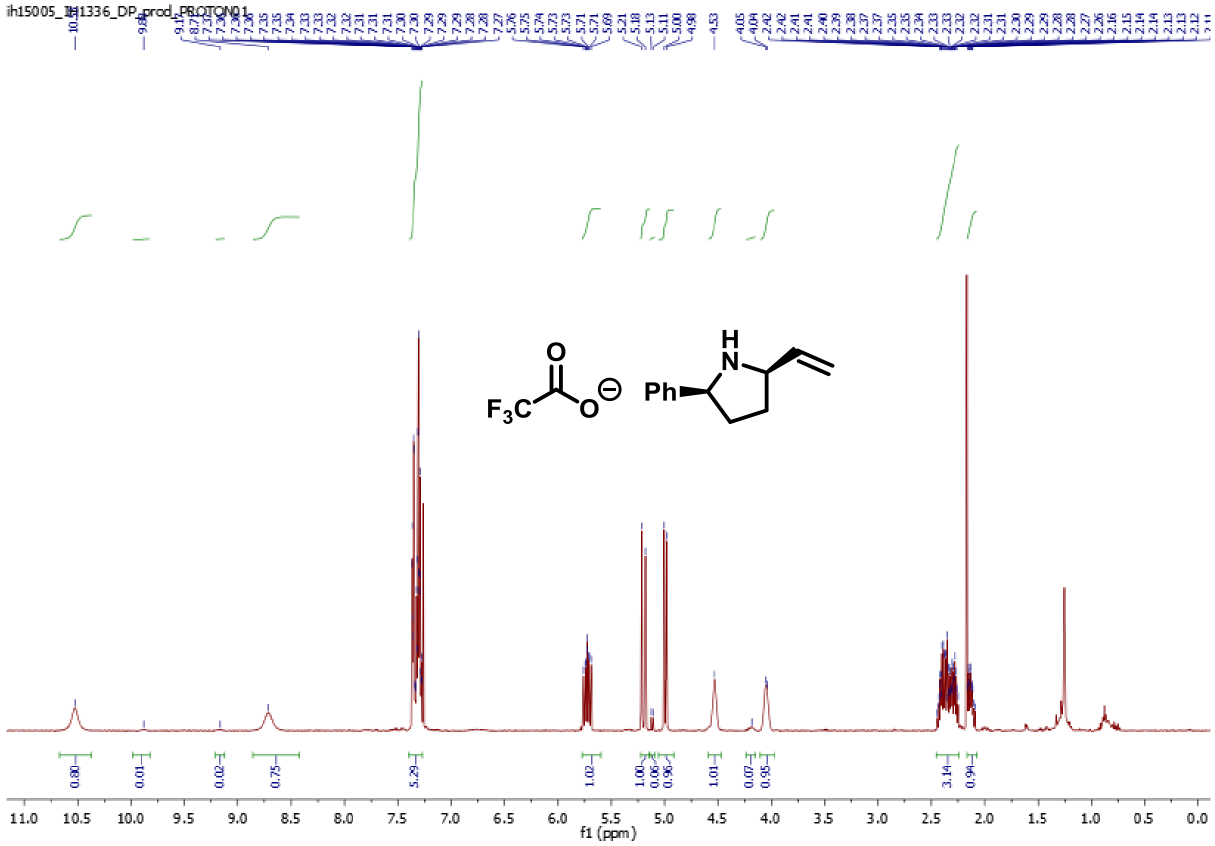
ih15005_IH1336_DP_prod_PROTON01
F3C
O
Ph
H
N
f1 (ppm)

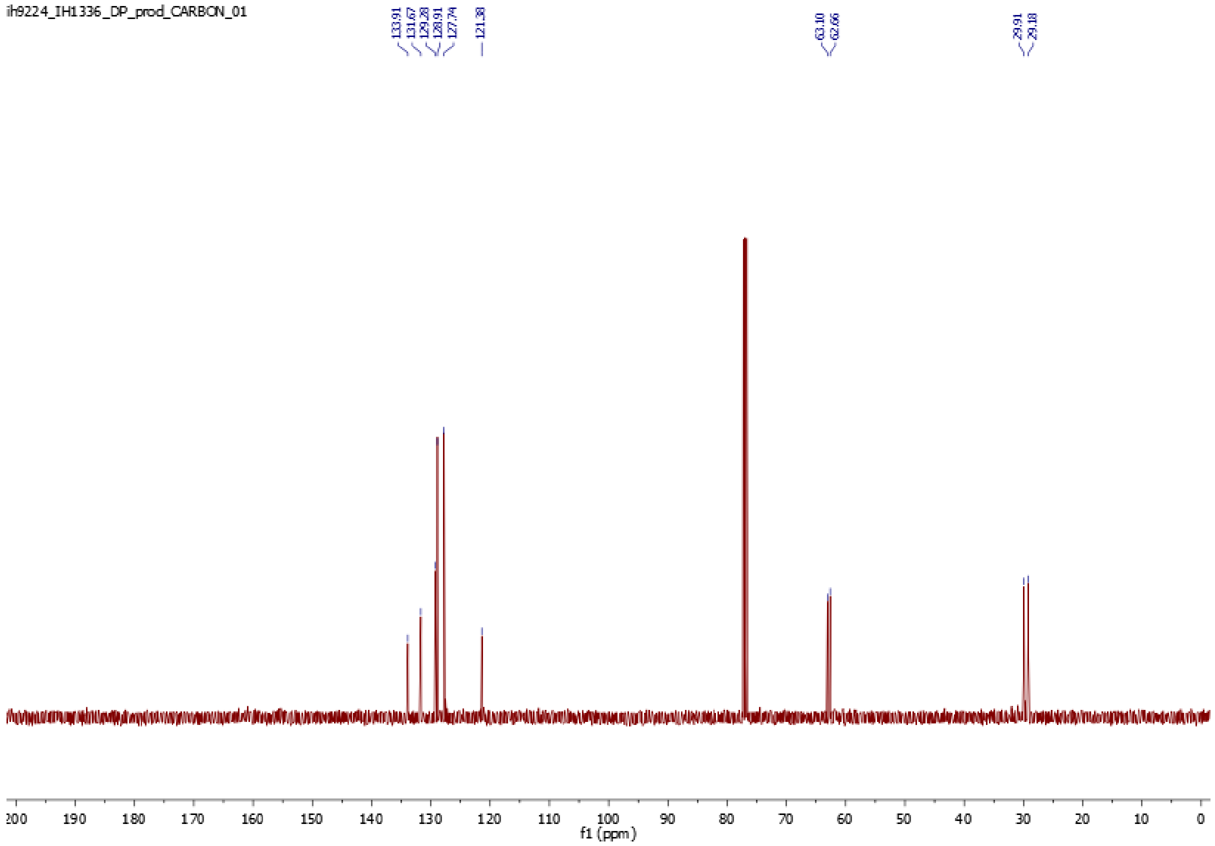
ih9224_IH1336_DP_prod_CARBON_01
133.91
131.67
129.28
128.91
127.74
121.38
63.10
62.66
29.91
29.18
f1 (ppm)

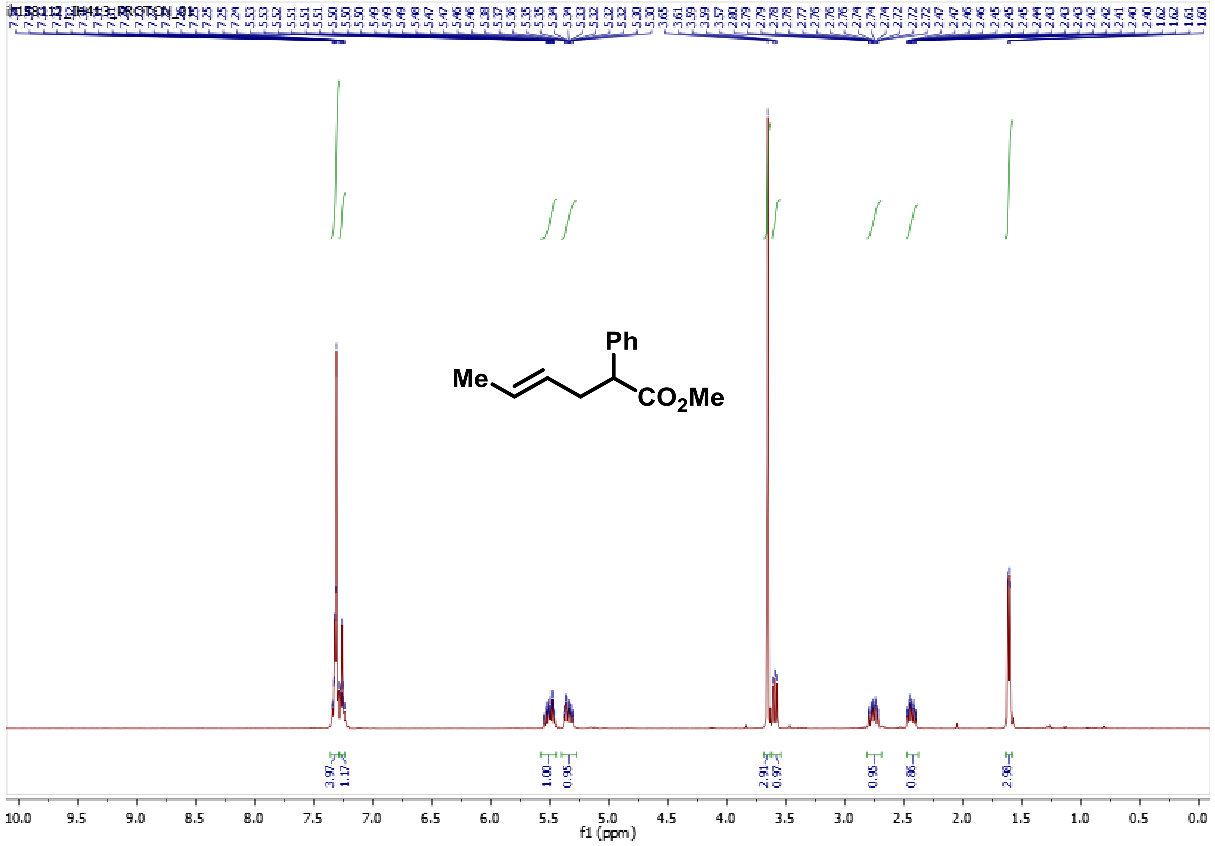
ih158112_IH413_PROTON_01
Ph
Me
CO_2Me
3.97
1.17
1.00
0.95
2.91
0.97
0.95
0.86
2.98
f1 (ppm)

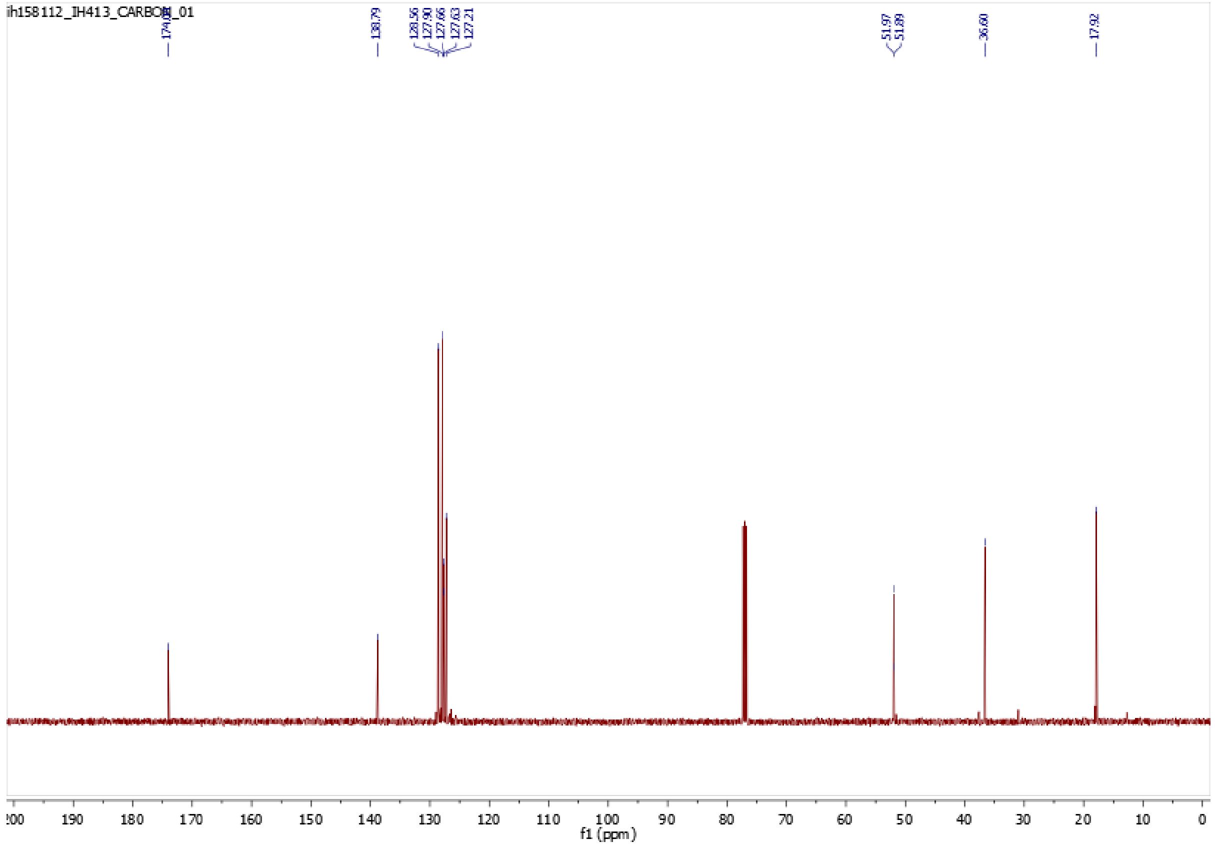
ih158112_IH413_CARBON_01
[illegible]
138.79
128.56
127.90
127.66
127.63
127.21
51.97
51.89
36.60
17.92
f1 (ppm)

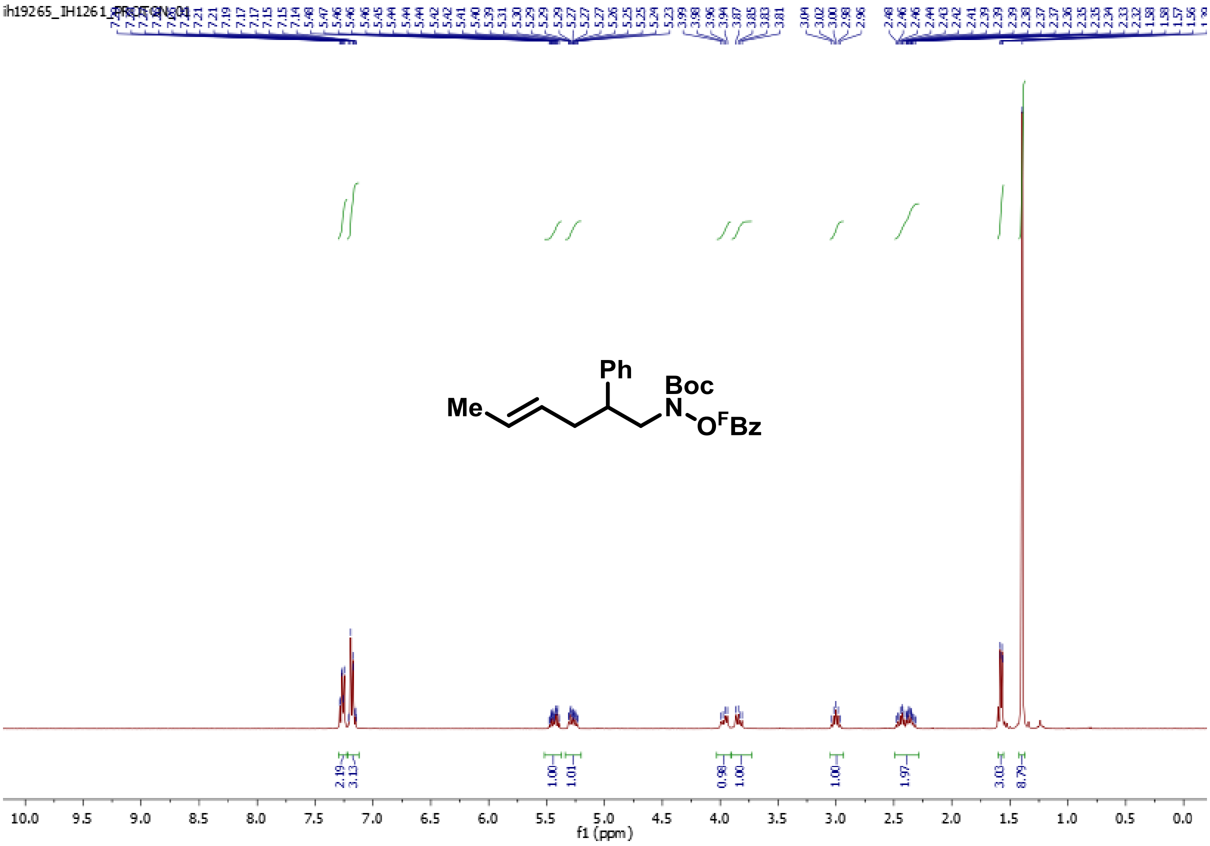
ih19265_IH1261_PROTON_01
Ph
Boc
Me
N
O
F
Bz
2.19
3.13
1.00
1.01
0.98
1.00
1.00
1.97
3.03
8.79
10.0 9.5 9.0 8.5 8.0 7.5 7.0 6.5 6.0 5.5 5.0 4.5 4.0 3.5 3.0 2.5 2.0 1.5 1.0 0.5 0.0
f1 (ppm)

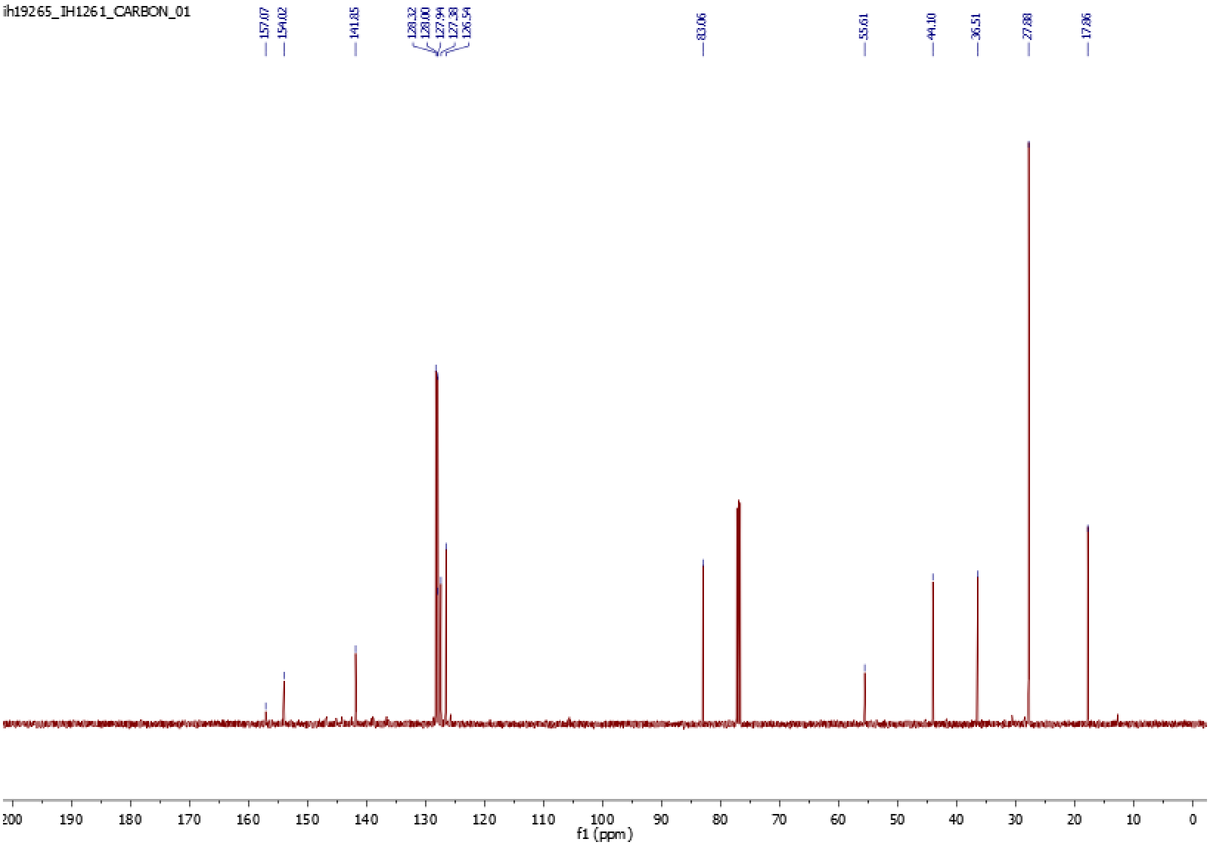
ih19265_IH1261_CARBON_01
157.07
154.02
141.85
128.32
128.00
127.94
127.38
126.54
83.06
55.61
44.10
36.51
27.88
17.86
200 190 180 170 160 150 140 130 120 110 100 90 80 70 60 50 40 30 20 10 0
f1 (ppm)

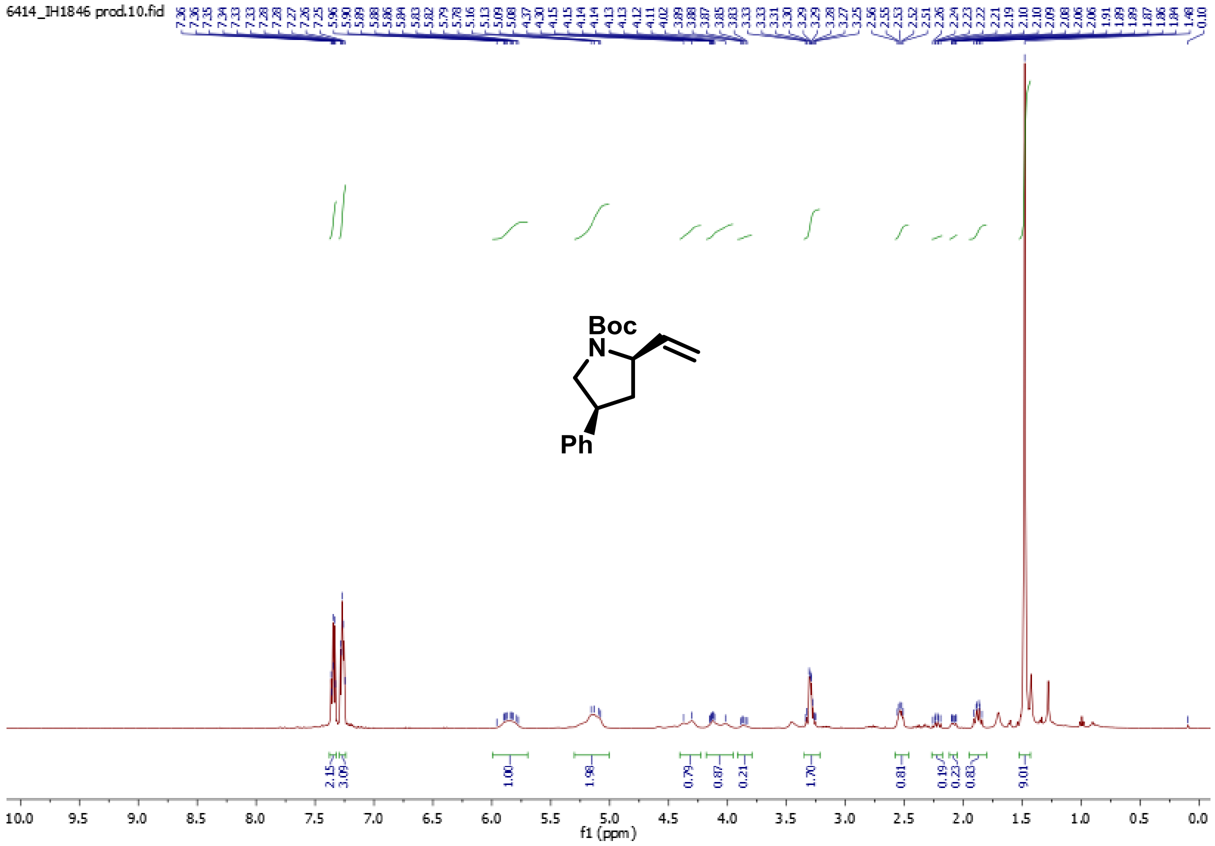
6414_IH1846 prod.10.fid
Boc
N
Ph
f1 (ppm)

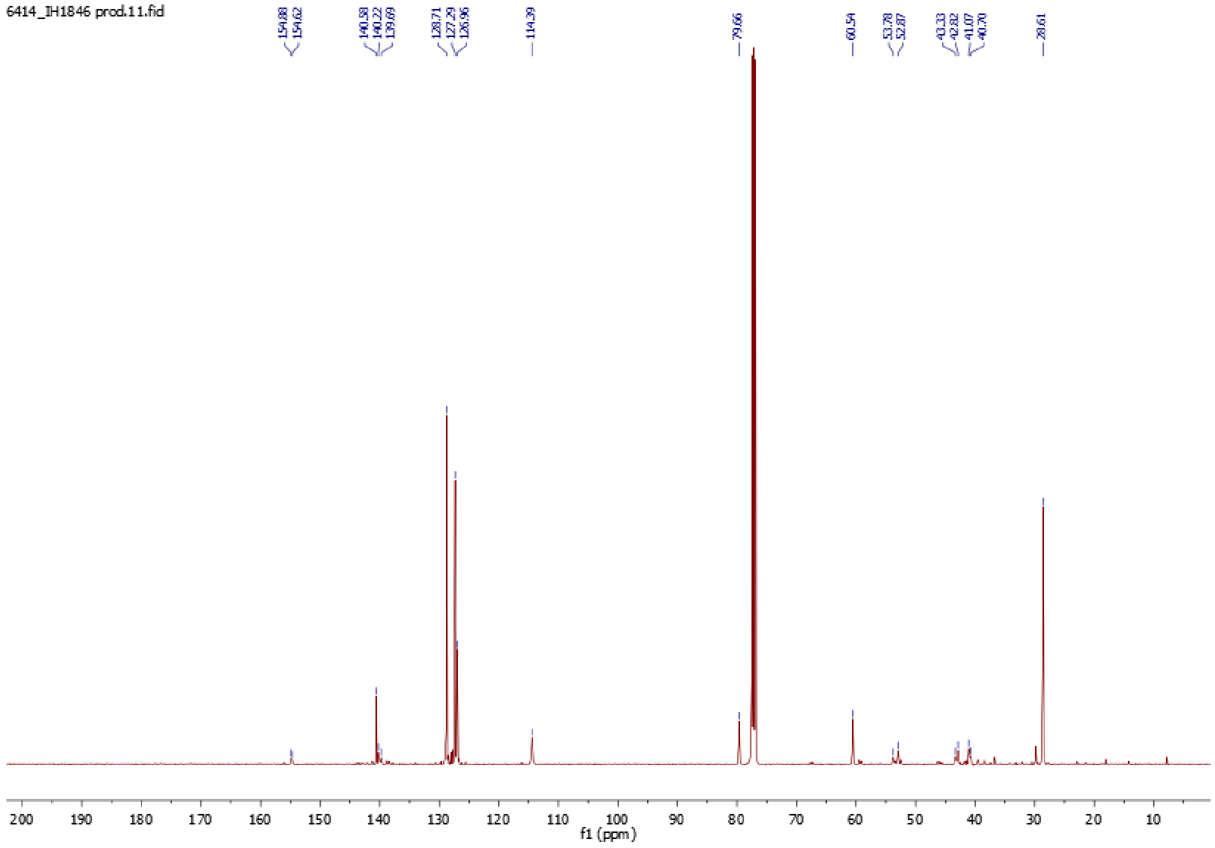
6414_IH1846 prod.11.fid
154.88
154.62
140.58
140.22
139.69
128.71
127.29
126.96
114.39
79.66
60.54
53.78
52.87
43.33
42.82
41.07
40.70
28.61
f1 (ppm)

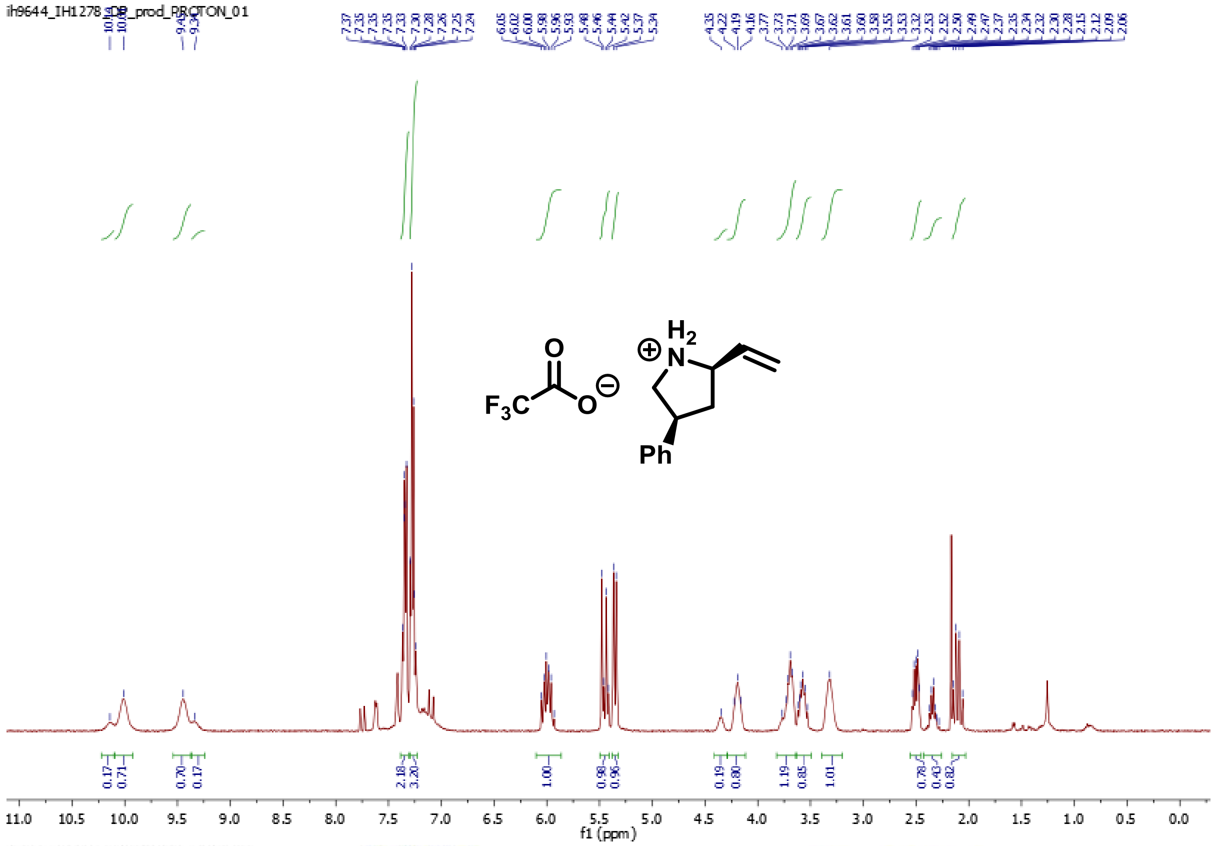

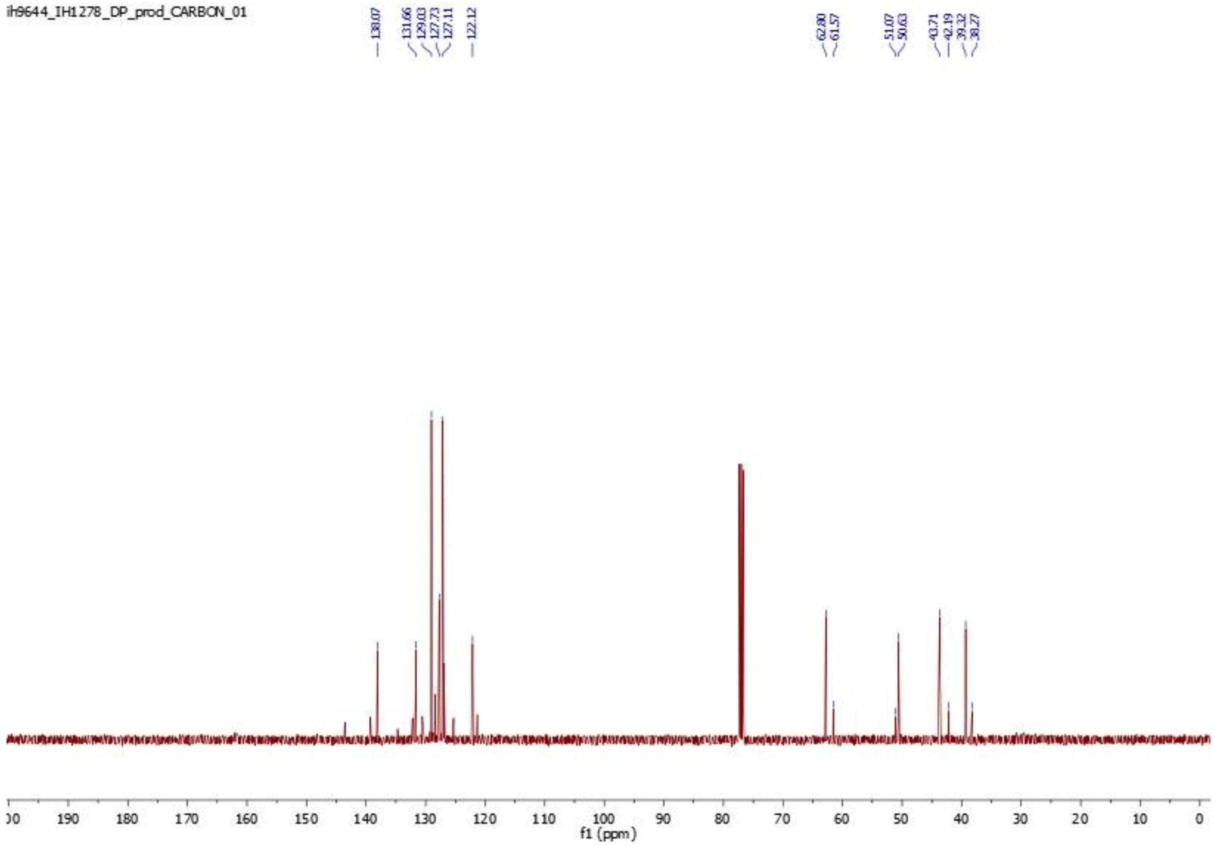

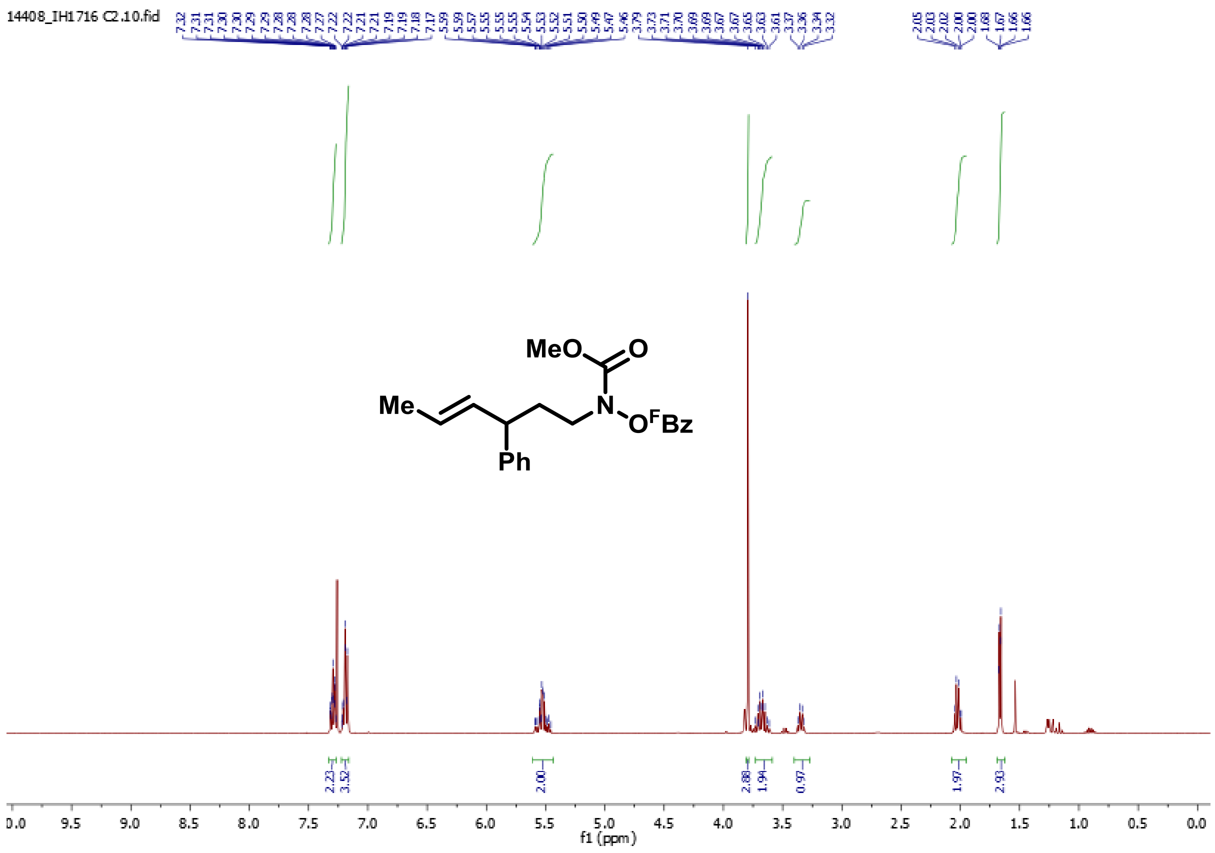
14408_IH1716 C2.10.fid
7.32 7.31 7.31 7.30 7.30 7.29 7.29 7.28 7.28 7.28 7.27 7.22 7.22 7.21 7.21 7.19 7.19 7.18 7.17 5.59 5.59 5.57 5.55 5.55 5.55 5.54 5.53 5.52 5.51 5.50 5.49 5.47 5.46 3.79 3.73 3.71 3.70 3.69 3.69 3.67 3.67 3.65 3.63 3.61 3.57 3.36 3.34 3.32
2.05 2.03 2.02 2.00 2.00 1.68 1.67 1.66 1.66
MeO
O
Me
N
O
F
Bz
Ph
2.23
3.52
2.00
2.88
1.94
0.97
1.97
2.93
f1 (ppm)

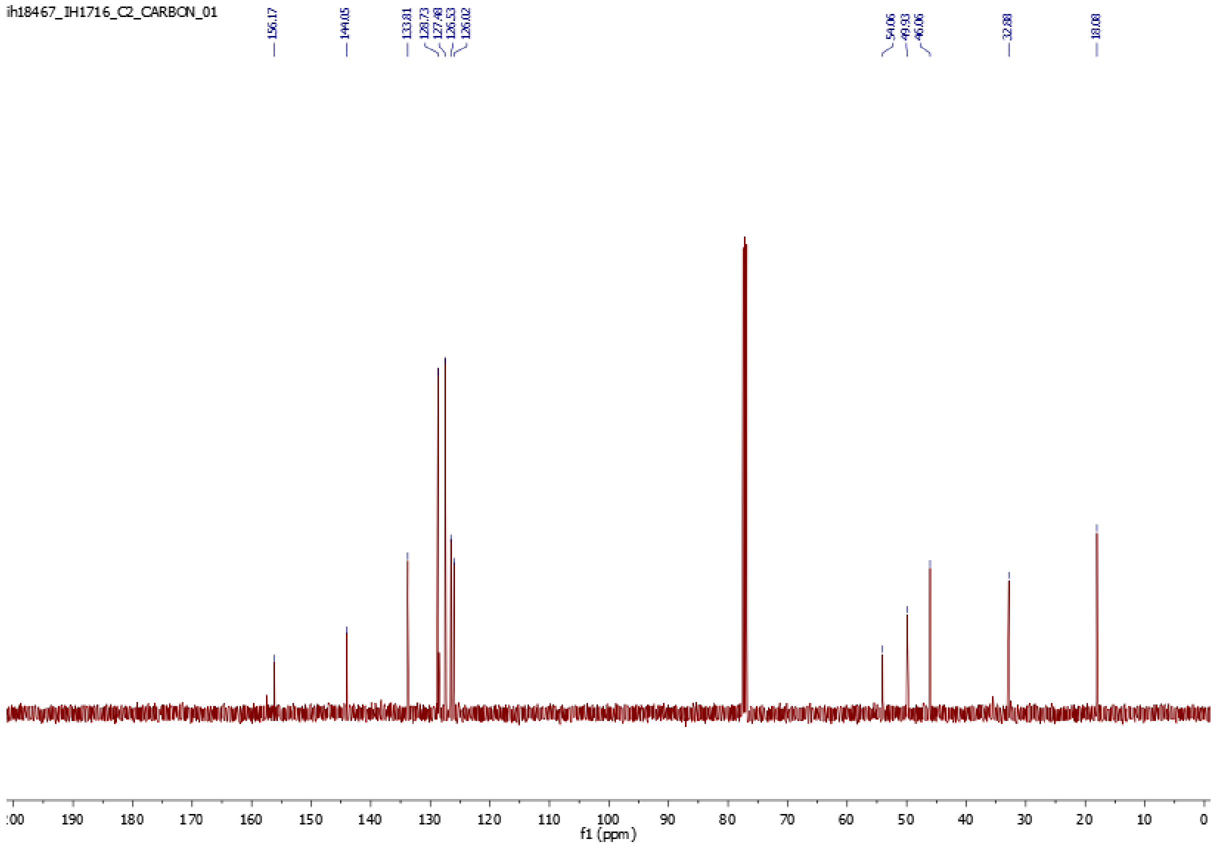
ih18467_IH1716_C2_CARBON_01
156.17
144.05
133.81
128.73
127.48
126.53
126.02
54.06
49.93
46.06
32.88
18.08
f1 (ppm)

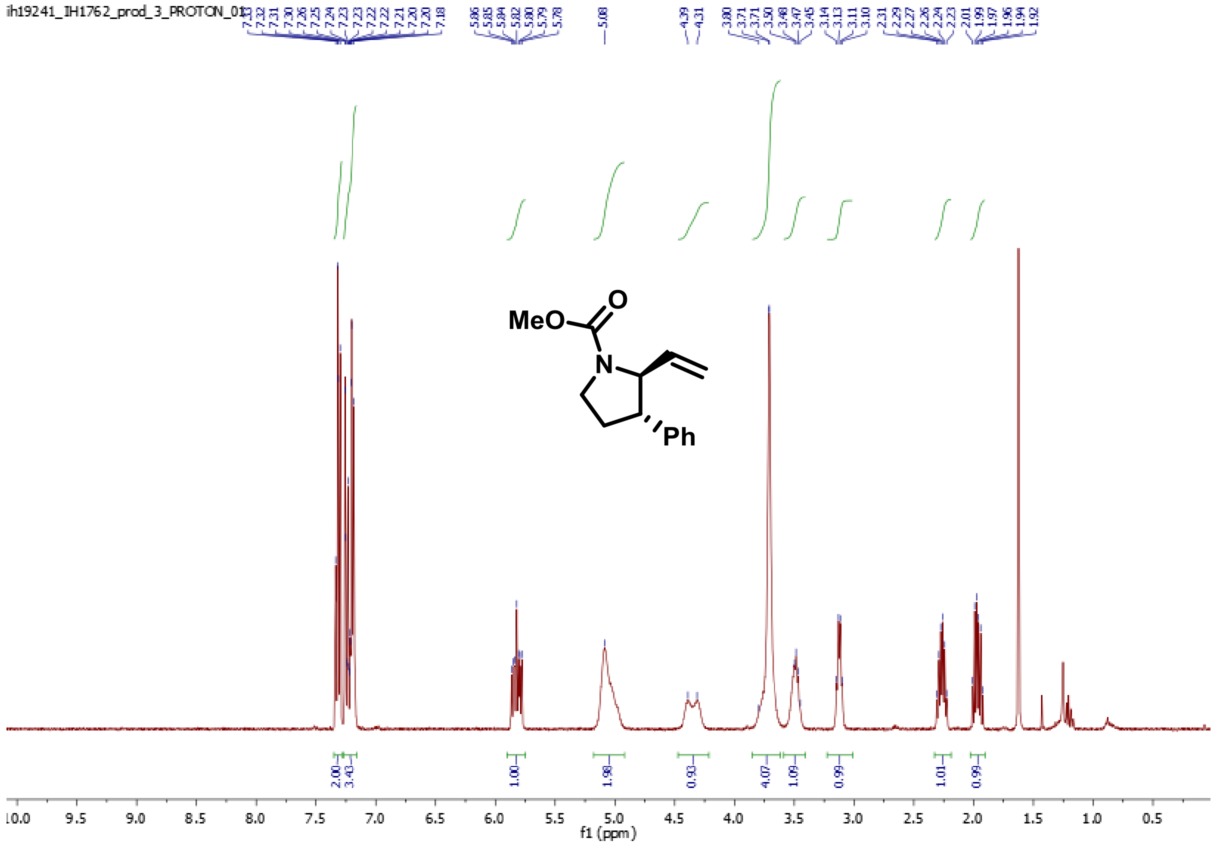
ih19241_IH1762_prod_3_PROTON_01
MeO
O
N
Ph
2.00
3.43
1.00
1.98
0.93
4.07
1.09
0.99
1.01
0.99
f1 (ppm)

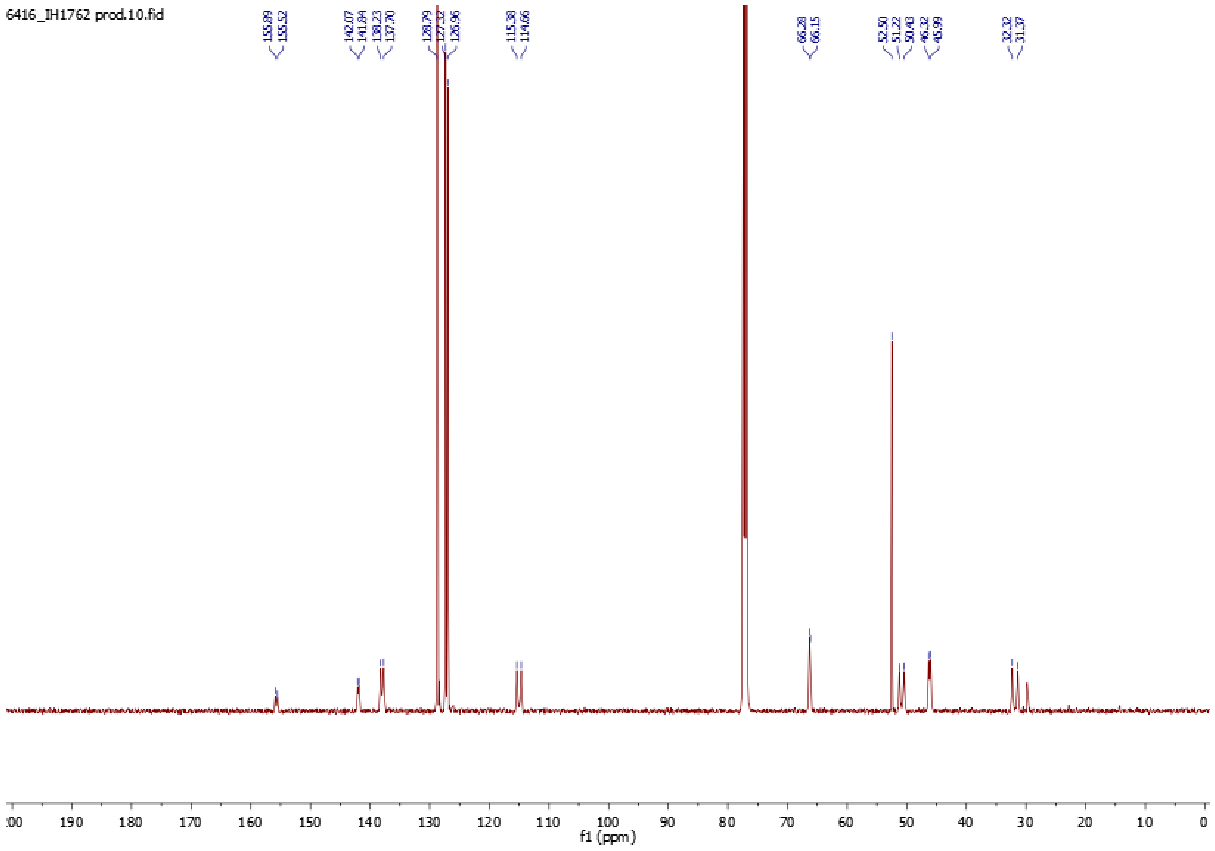
6416_IH1762 prod.10.fid
155.89
155.52
142.07
141.84
138.23
137.70
128.79
127.32
126.96
115.38
114.66
66.28
66.15
52.50
51.22
50.43
46.32
45.99
32.32
31.37
f1 (ppm)

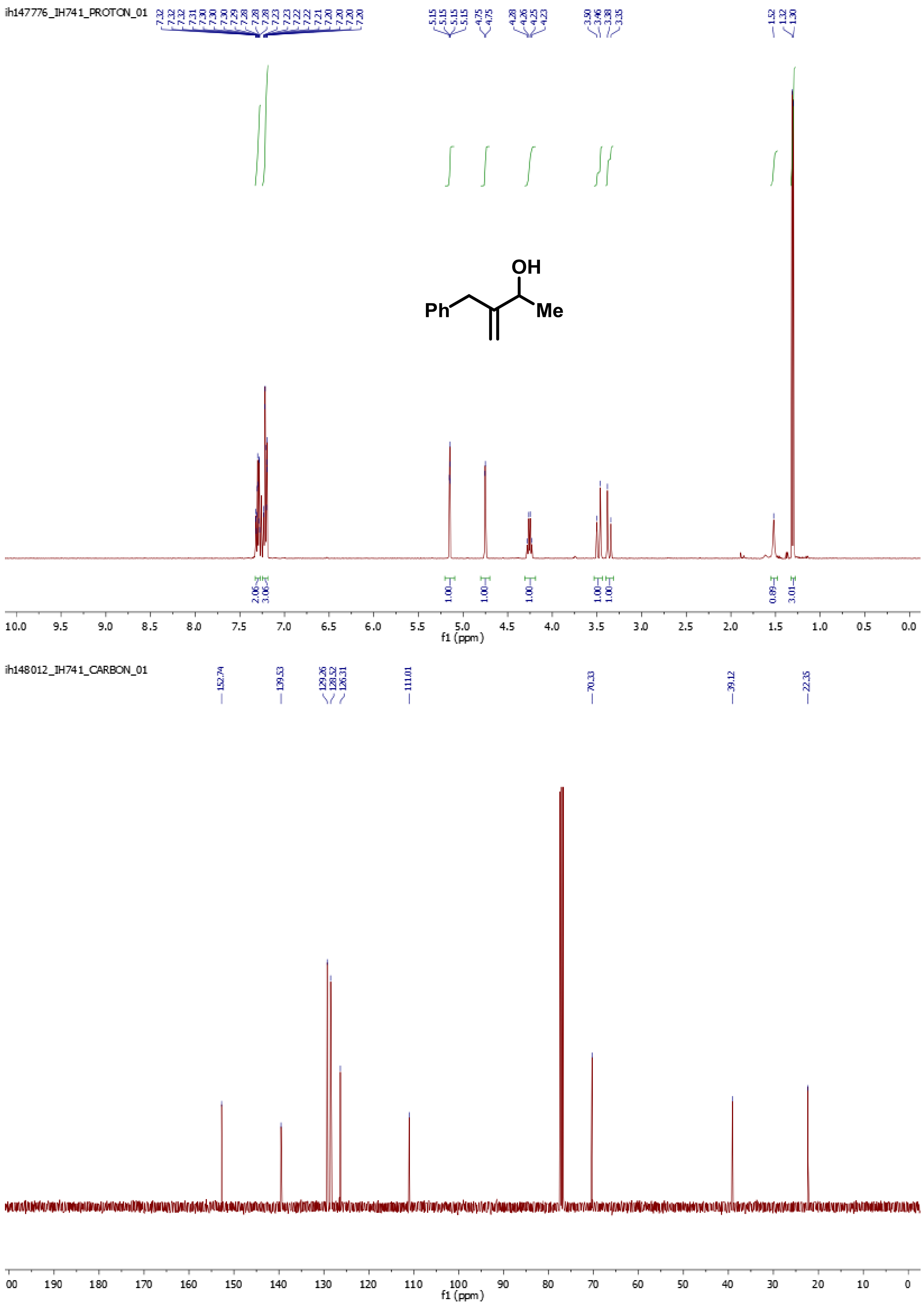
ih147776_IH741_PROTON_01
OH
Ph
Me
f1 (ppm)
ih148012_IH741_CARBON_01
152.74
139.53
129.26
128.52
126.31
111.01
70.33
39.12
22.35
f1 (ppm)

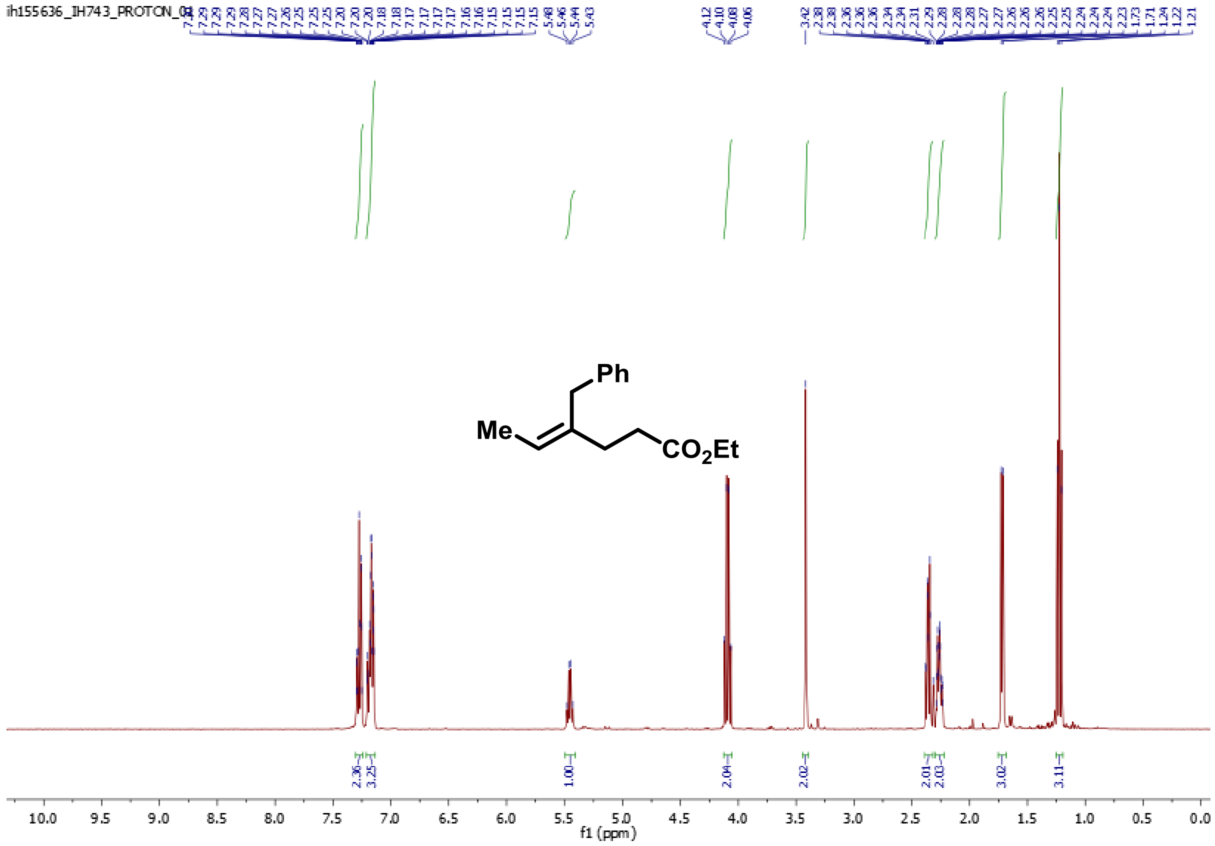
ih155636_IH743_PROTON_01
Ph
Me
CO2Et
f1 (ppm)

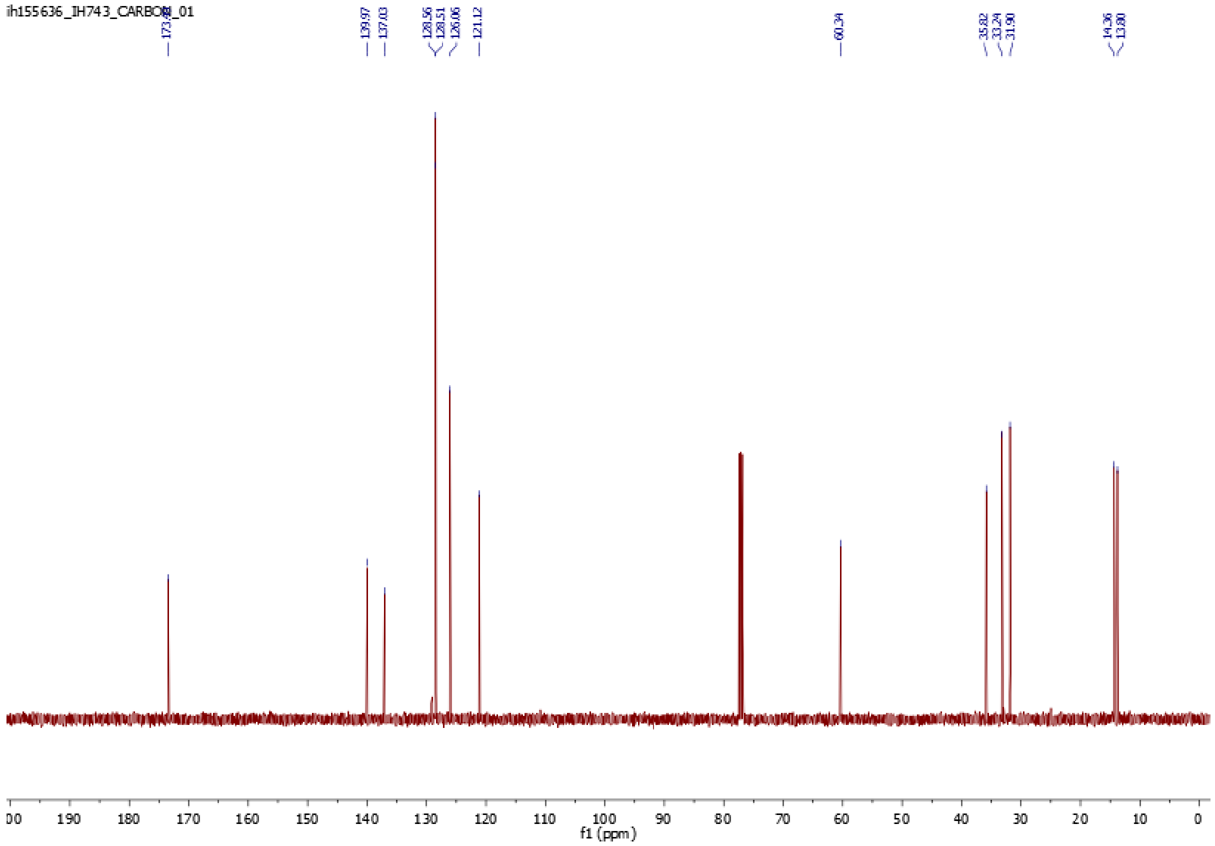
ih155636_IH743_CARBON_01
f1 (ppm)

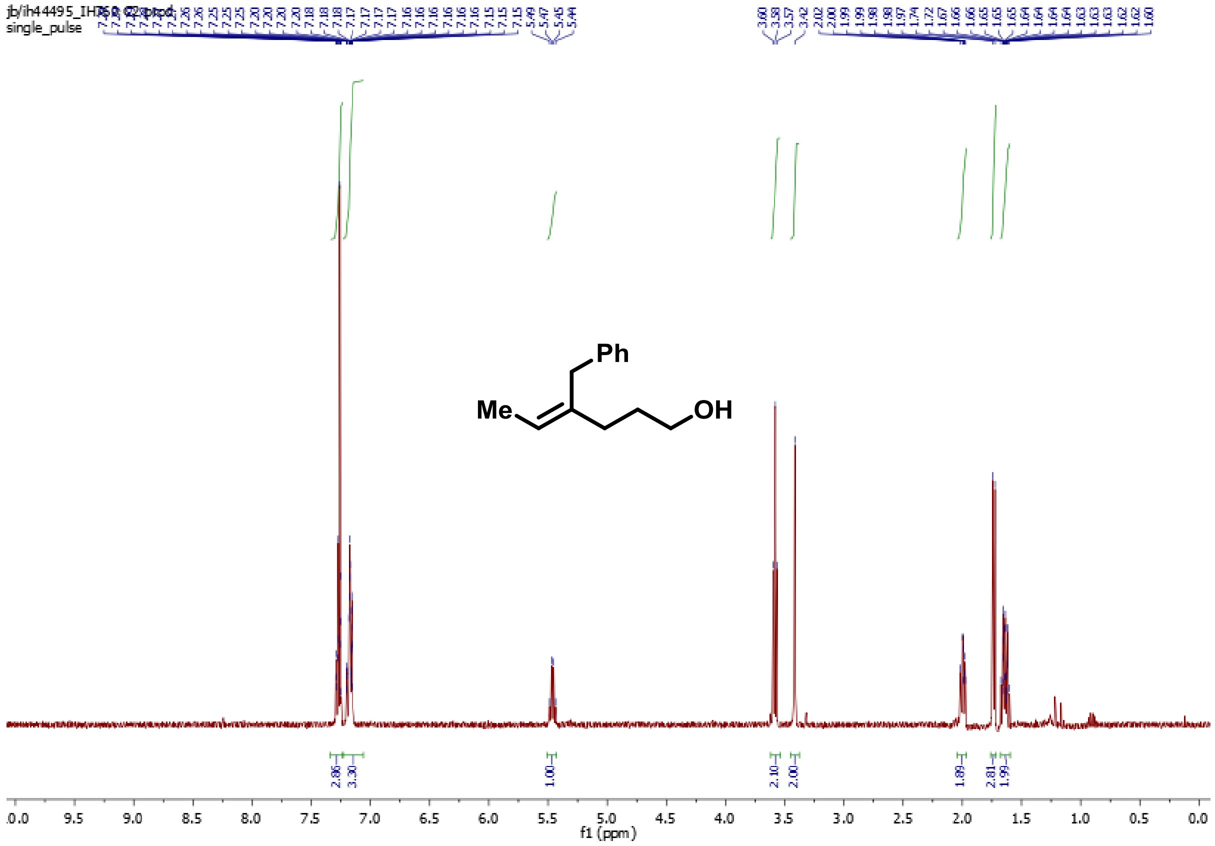

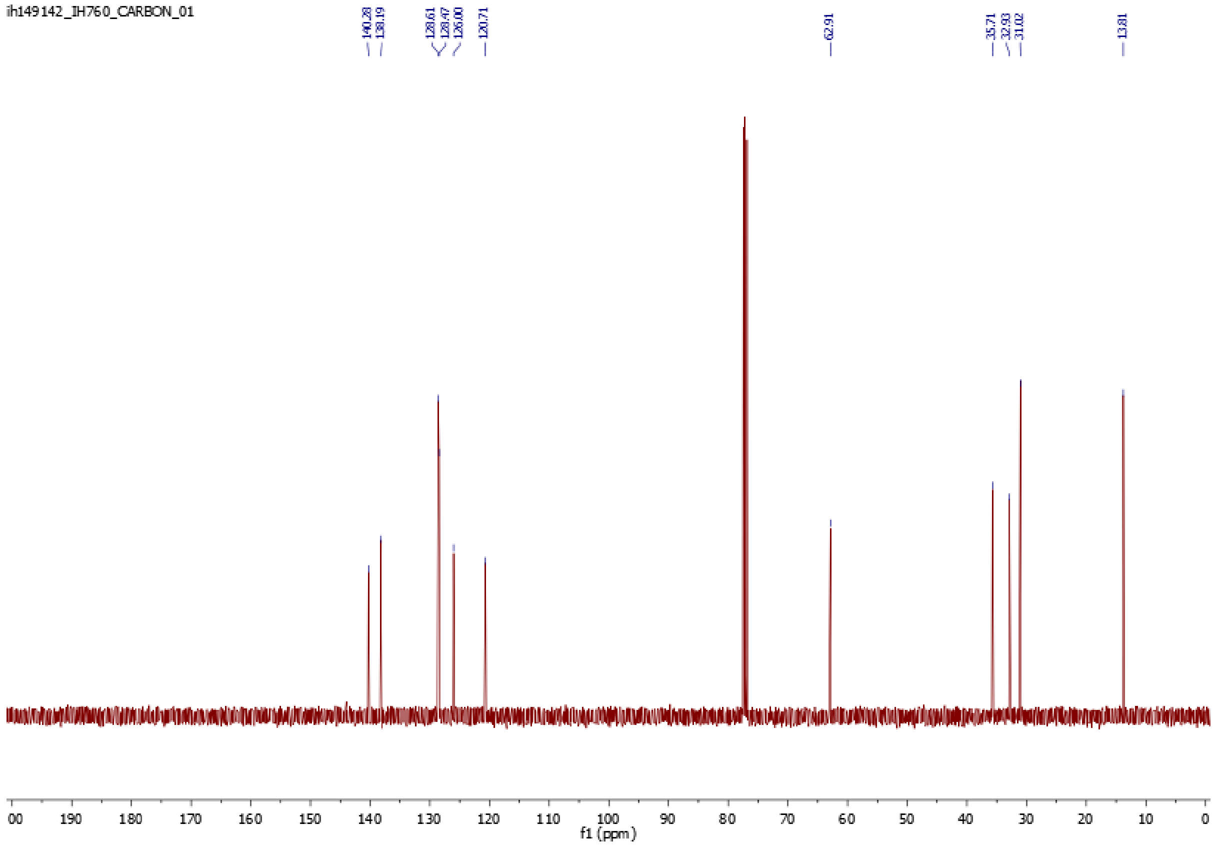

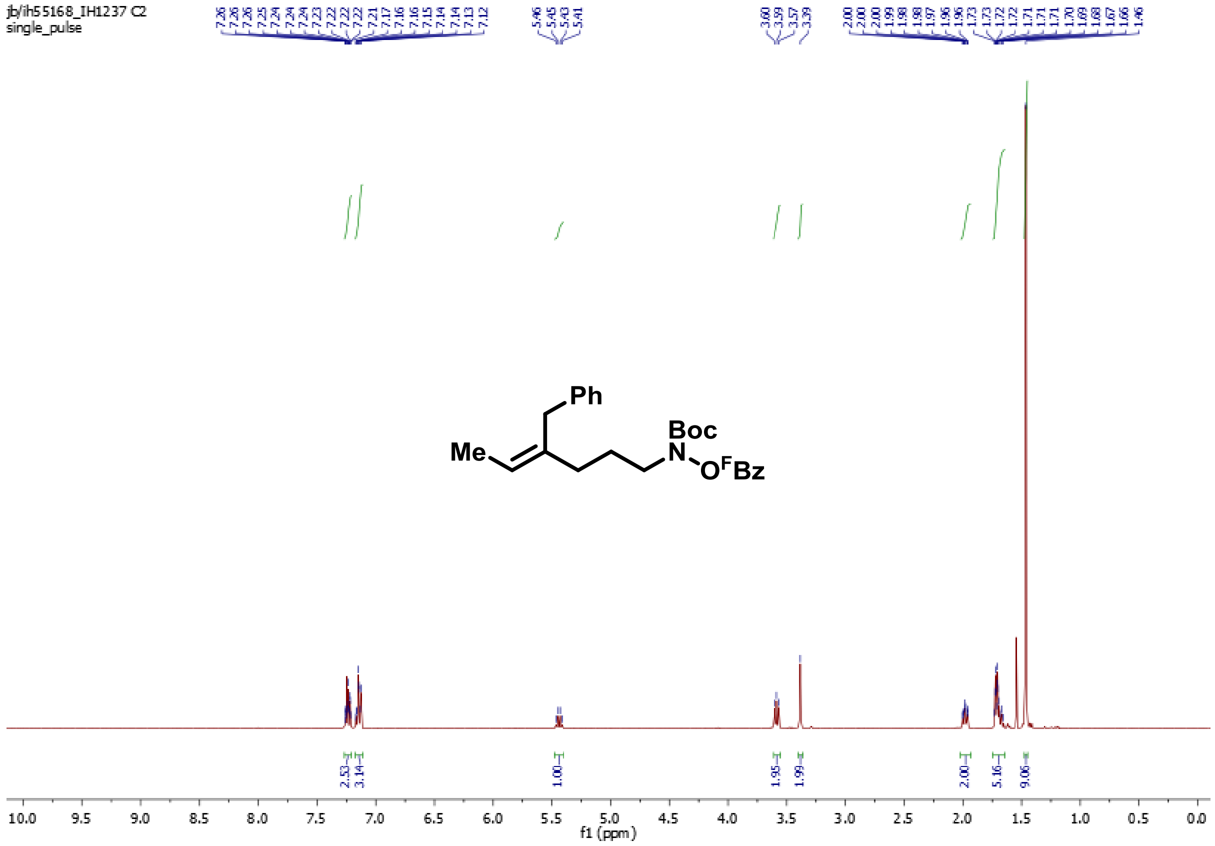
jb/ih55168_IH1237 C2
single_pulse
Ph
Boc
Me
N
O
F
Bz
f1 (ppm)

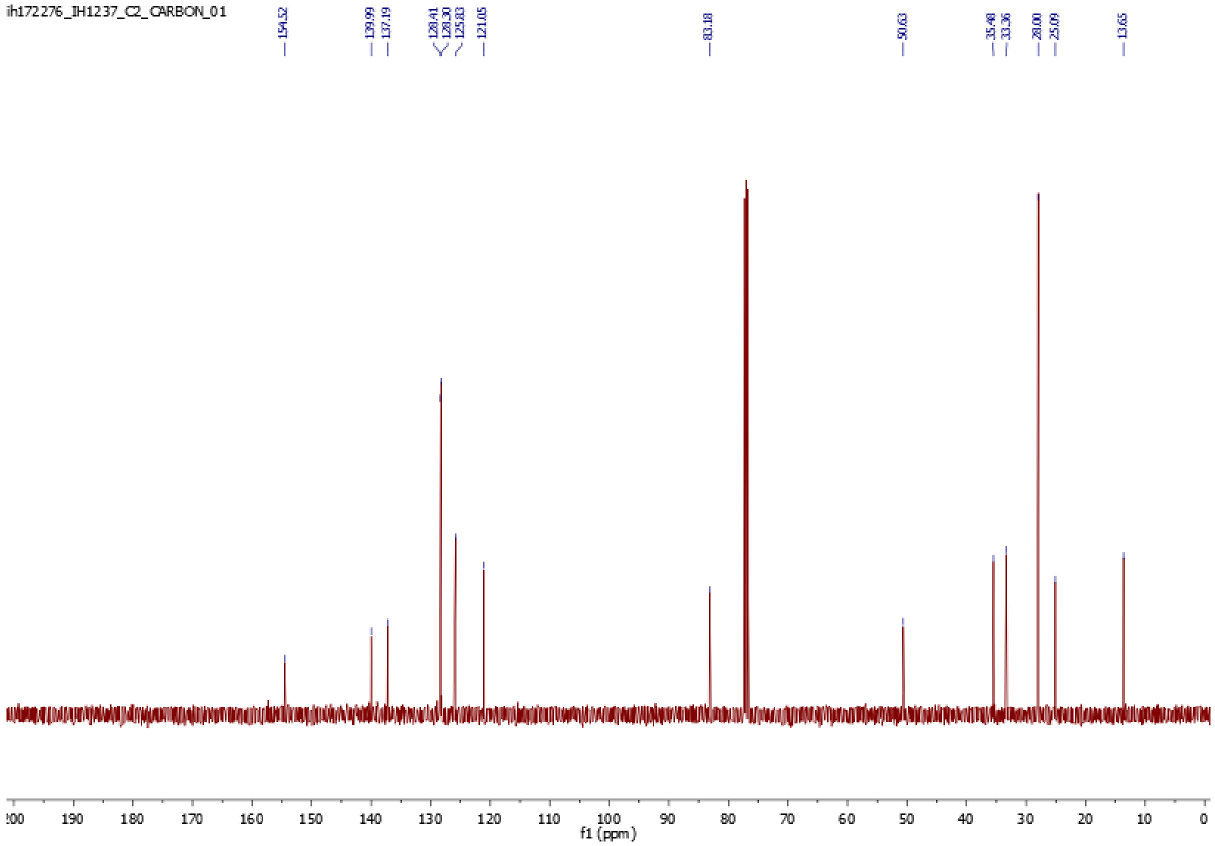
ih172276_IH1237_C2_CARBON_01
f1 (ppm)

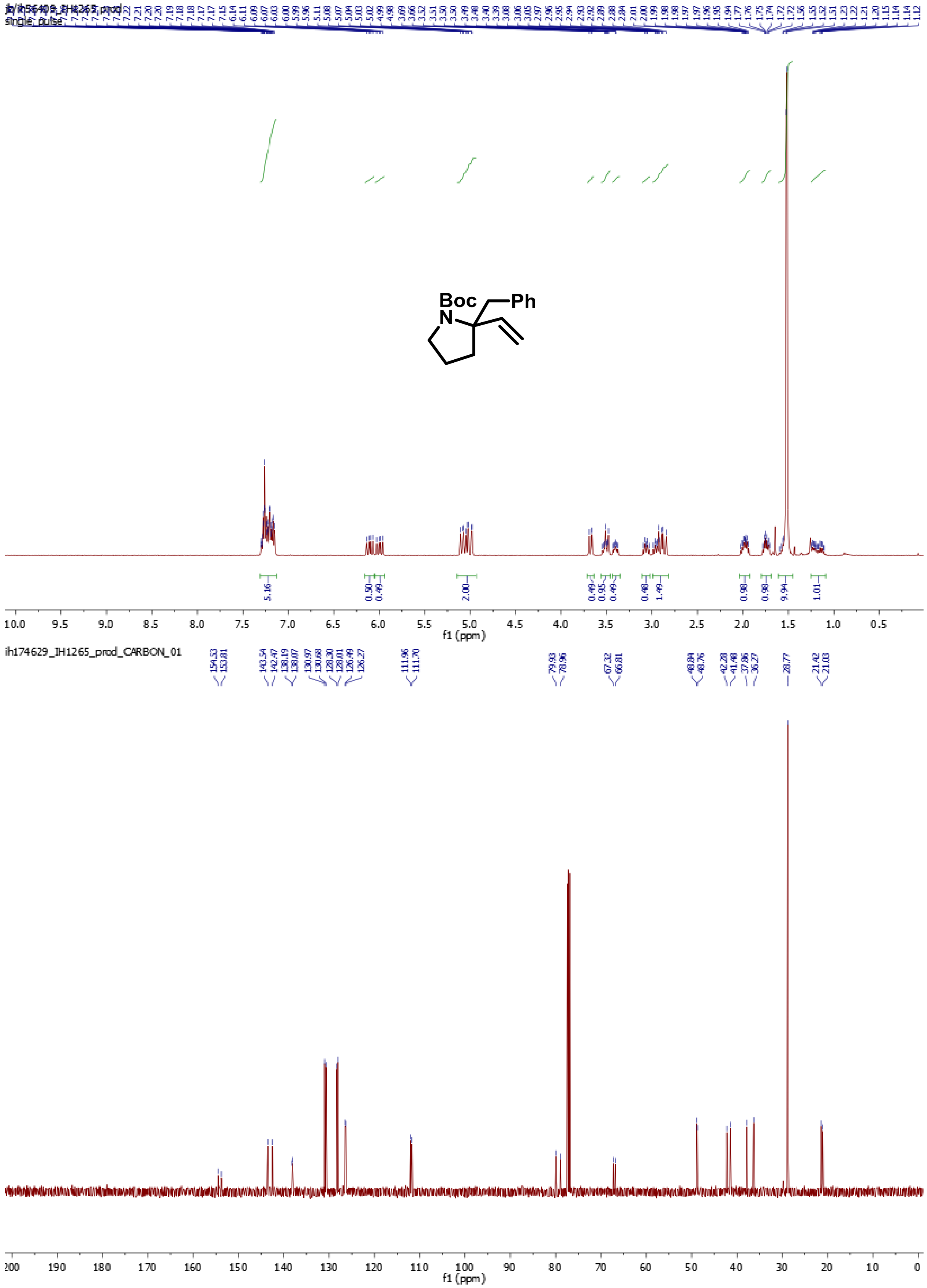
Boc
N
Ph
ih174629_IH1265_prod_CARBON_01
f1 (ppm)

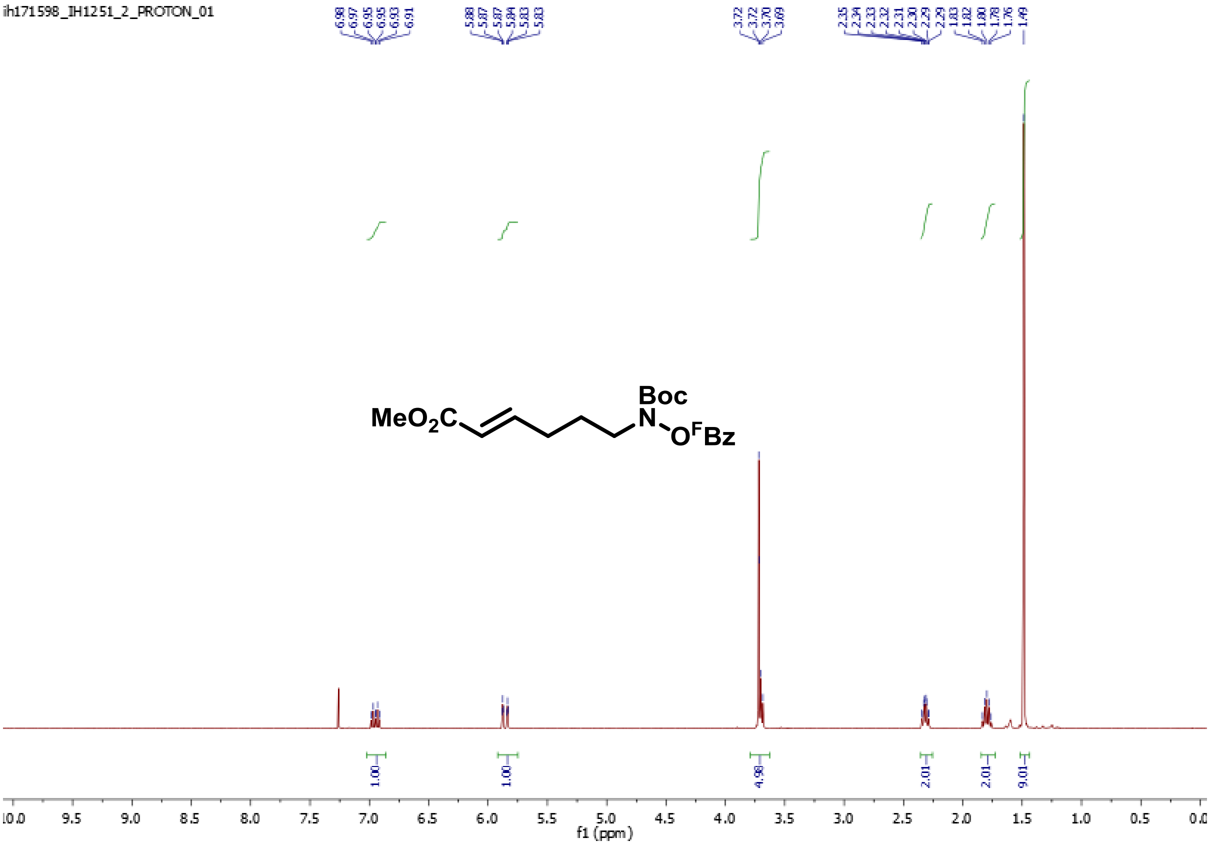
ih171598_IH1251_2_PROTON_01
6.98
6.97
6.95
6.95
6.93
6.91
5.88
5.87
5.87
5.84
5.83
5.83
3.72
3.72
3.70
3.69
2.35
2.34
2.33
2.32
2.31
2.30
2.29
2.29
1.83
1.82
1.80
1.78
1.76
1.49
MeO2C
Boc
N
OFBz
1.00
1.00
4.98
2.01
2.01
9.01
f1 (ppm)

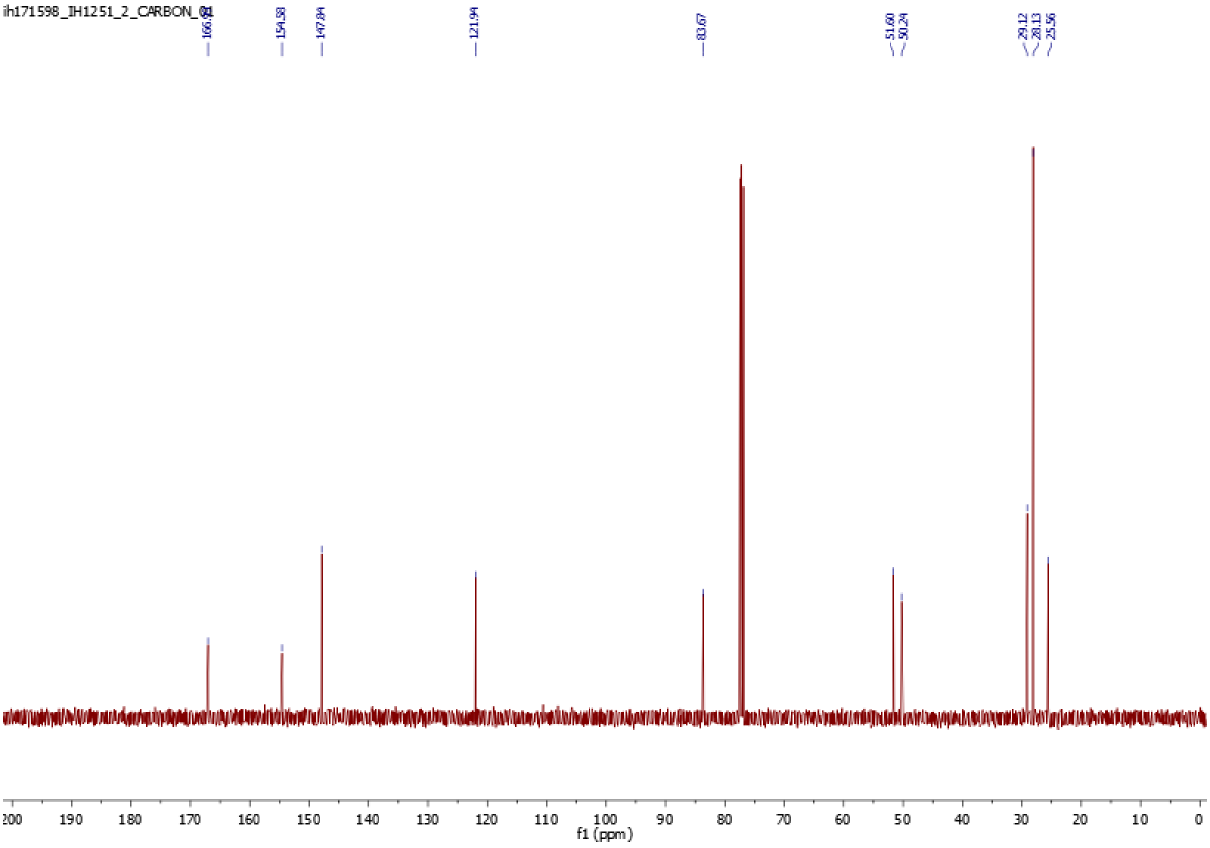
ih171598_IH1251_2_CARBON_01
166.91
154.58
147.84
121.94
83.67
51.60
50.24
29.12
28.13
25.56
f1 (ppm)

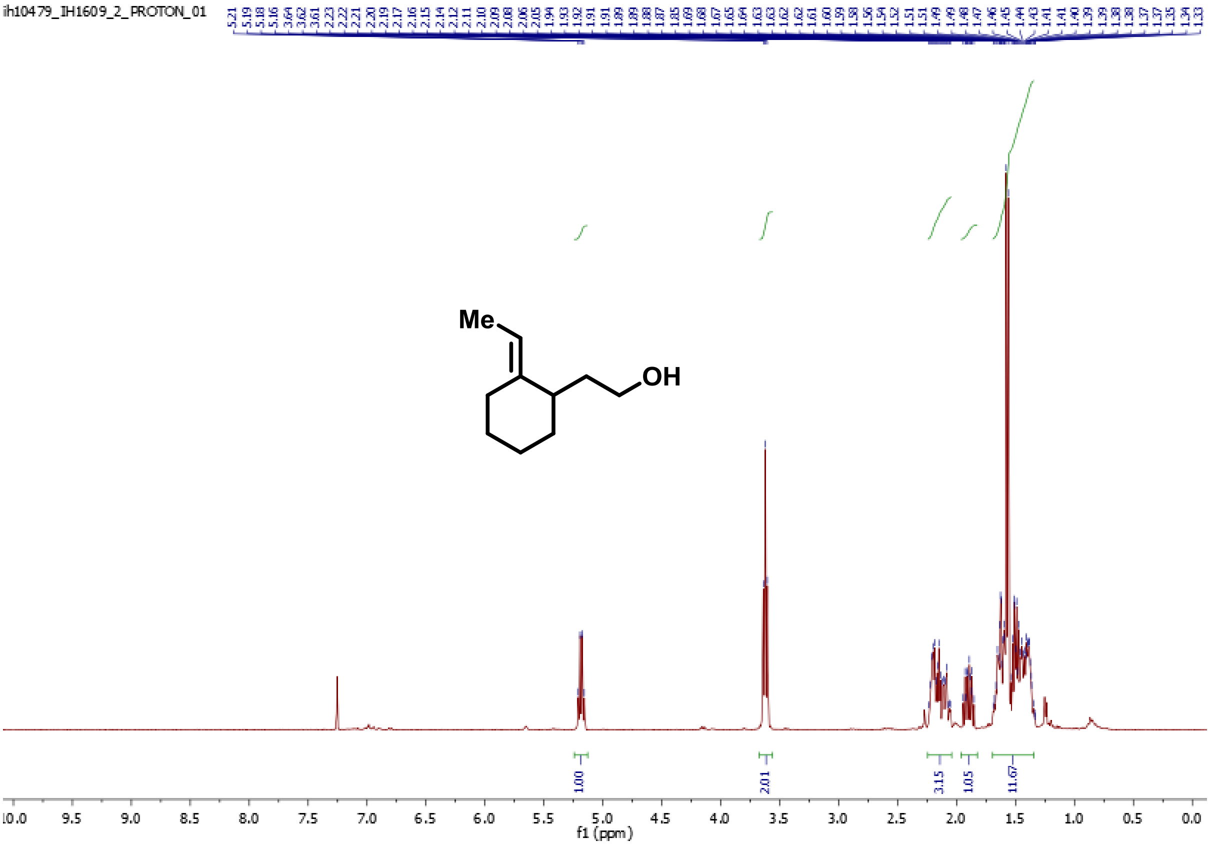
ih10479_IH1609_2_PROTON_01
5.21 5.19 5.18 5.16 3.64 3.62 3.61 2.23 2.22 2.21 2.20 2.19 2.17 2.16 2.15 2.14 2.12 2.11 2.10 2.09 2.08 2.06 2.05 1.94 1.93 1.92 1.91 1.91 1.89 1.88 1.87 1.85 1.69 1.68 1.67 1.65 1.64 1.63 1.63 1.62 1.62 1.61 1.60 1.59 1.58 1.56 1.54 1.52 1.51 1.51 1.49 1.49 1.48 1.47 1.46 1.45 1.44 1.43 1.41 1.40 1.39 1.39 1.38 1.38 1.37 1.37 1.35 1.34 1.33
Me
OH
1.00
2.01
3.15
1.05
11.67
f1 (ppm)

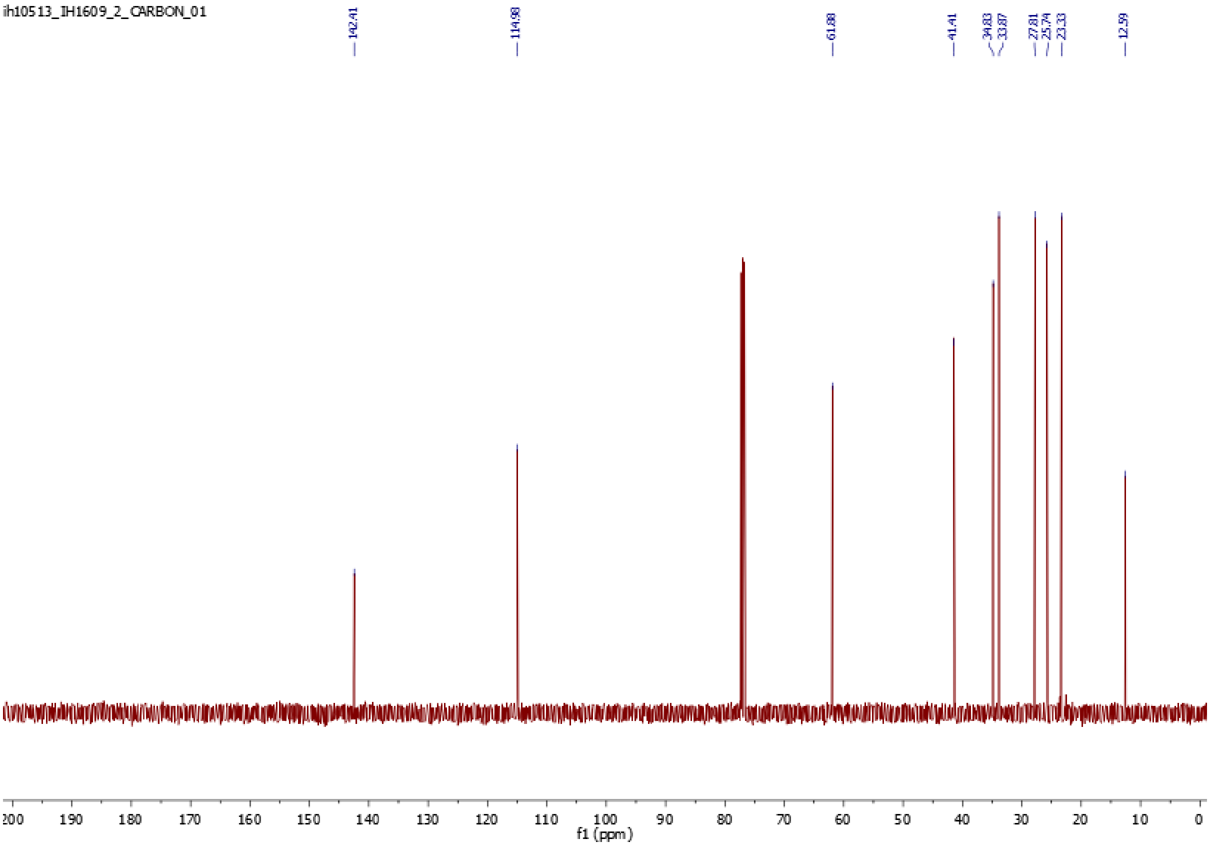
ih10513_IH1609_2_CARBON_01
142.41
114.98
61.88
41.41
34.83
33.87
27.81
25.74
23.33
12.59
f1 (ppm)

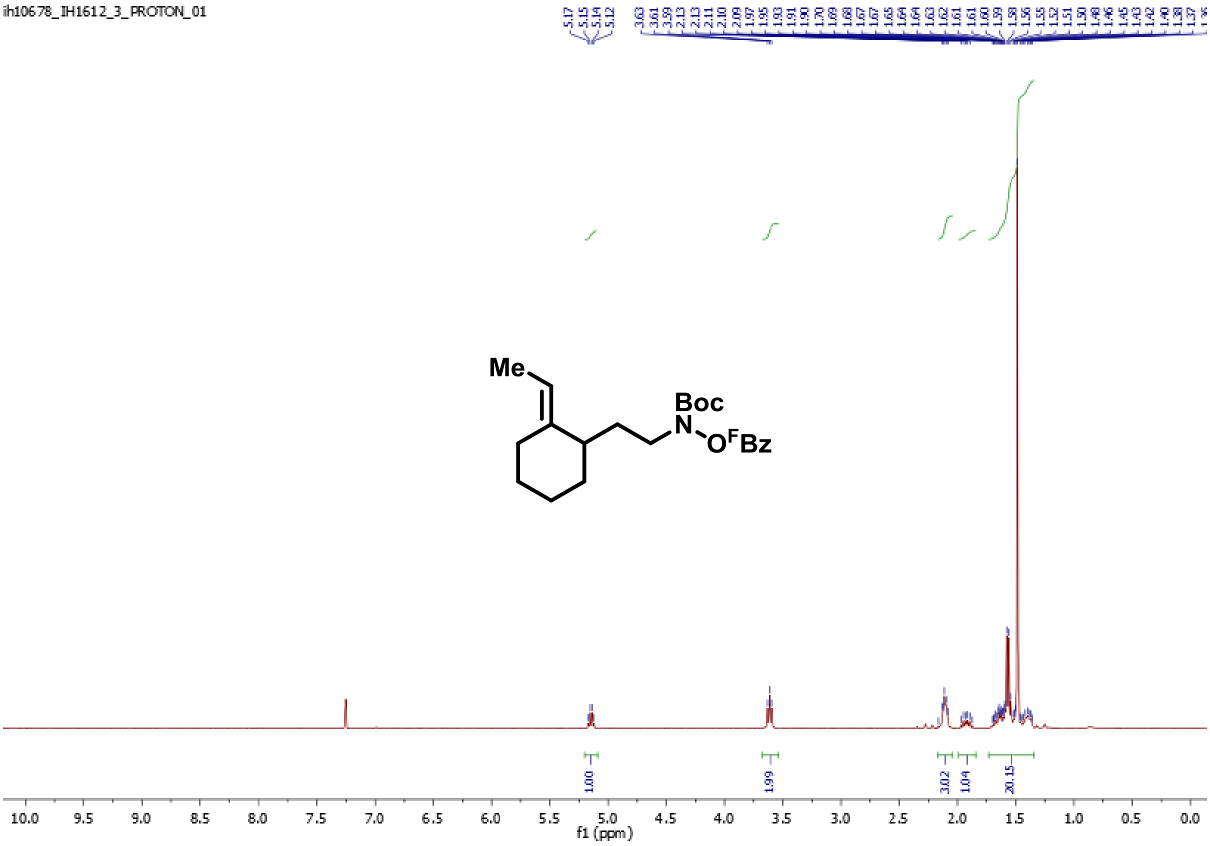
ih10678_IH1612_3_PROTON_01
5.17 5.15 5.14 5.12 3.63 3.61 3.59 2.13 2.13 2.11 2.10 2.09 1.97 1.95 1.93 1.91 1.90 1.70 1.69 1.68 1.67 1.67 1.65 1.64 1.64 1.63 1.62 1.61 1.61 1.60 1.59 1.58 1.56 1.55 1.52 1.51 1.50 1.48 1.46 1.45 1.43 1.42 1.40 1.38 1.37
Me
Boc
N
O
F
Bz
1.00
1.99
3.02
1.04
20.15
10.0 9.5 9.0 8.5 8.0 7.5 7.0 6.5 6.0 5.5 5.0 4.5 4.0 3.5 3.0 2.5 2.0 1.5 1.0 0.5 0.0
f1 (ppm)

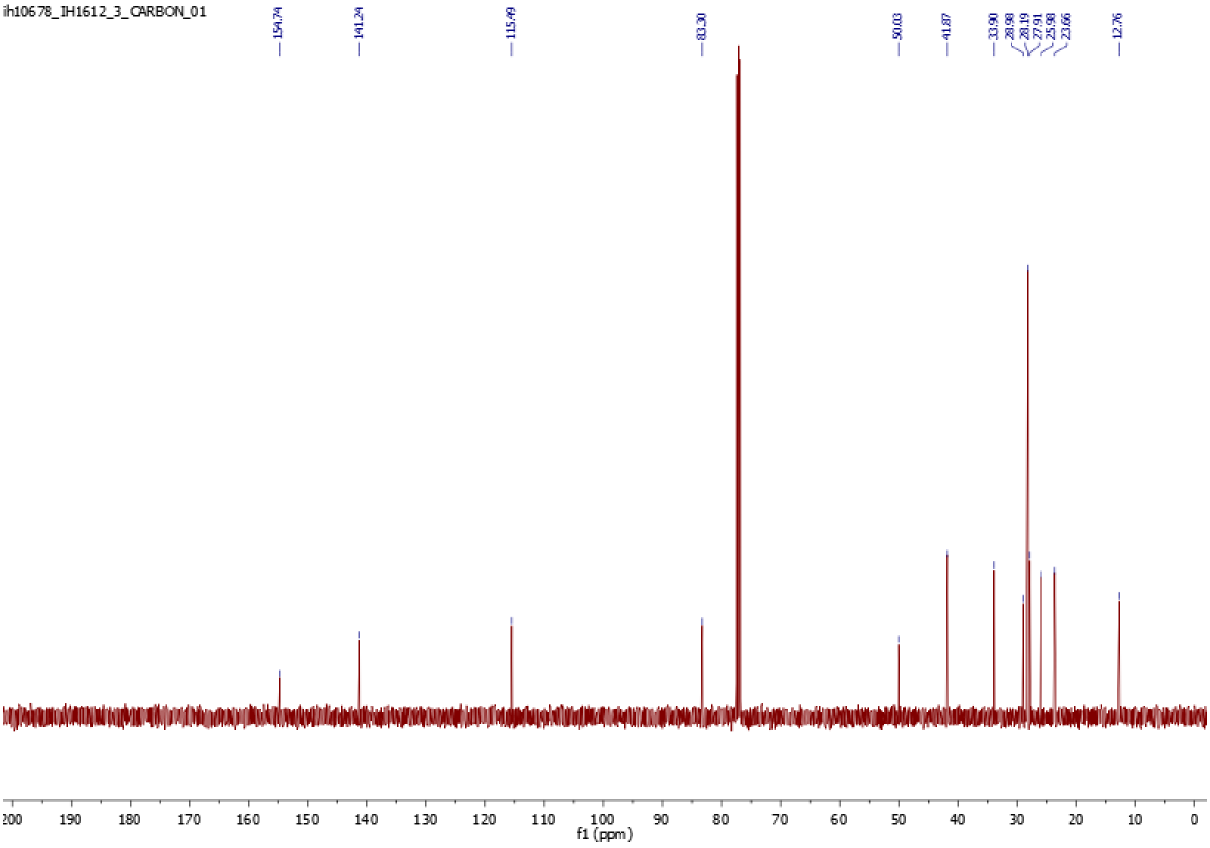
ih10678_IH1612_3_CARBON_01
154.74
141.24
115.49
83.30
50.03
41.87
33.90 28.98 28.19 27.91 25.98 23.66
12.76
200 190 180 170 160 150 140 130 120 110 100 90 80 70 60 50 40 30 20 10 0
f1 (ppm)

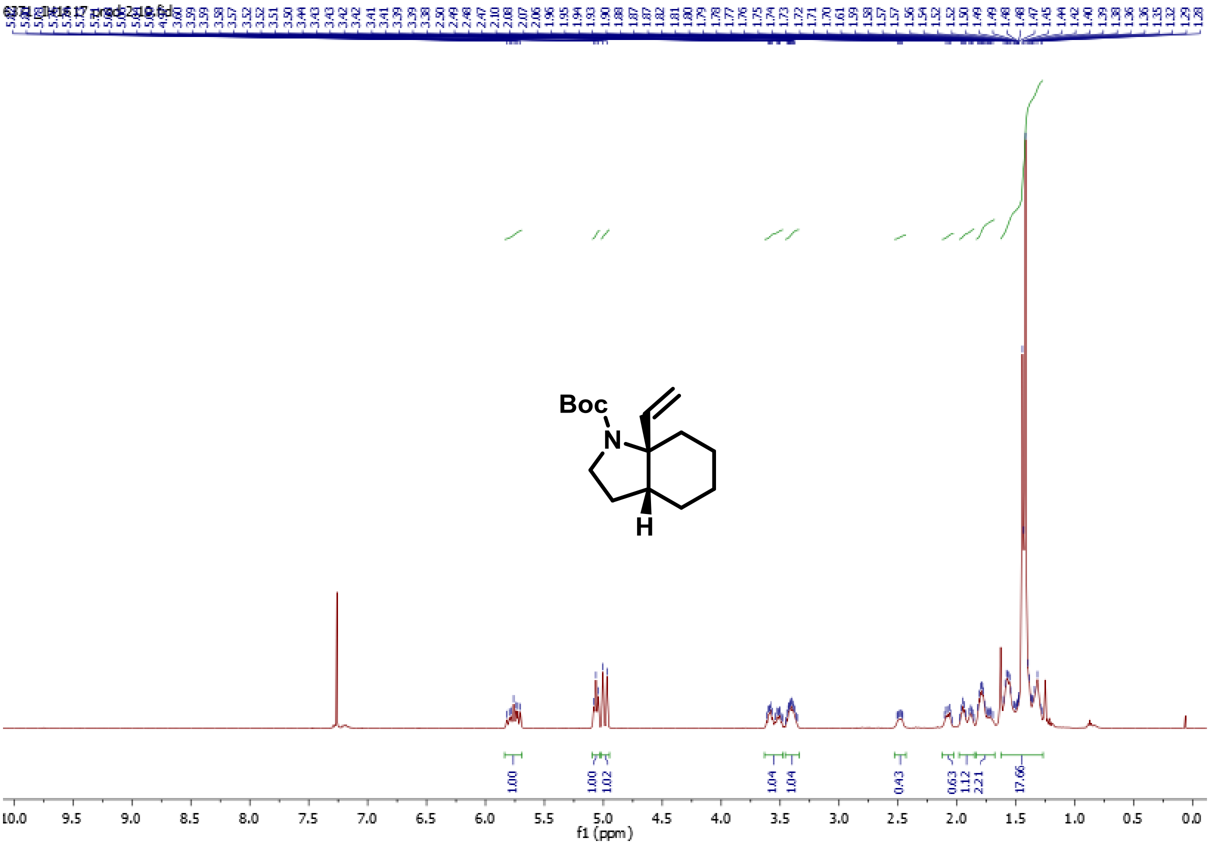
Boc
N
H
1.00
1.00
1.02
1.04
1.04
0.43
0.63
1.12
2.21
17.66
f1 (ppm)

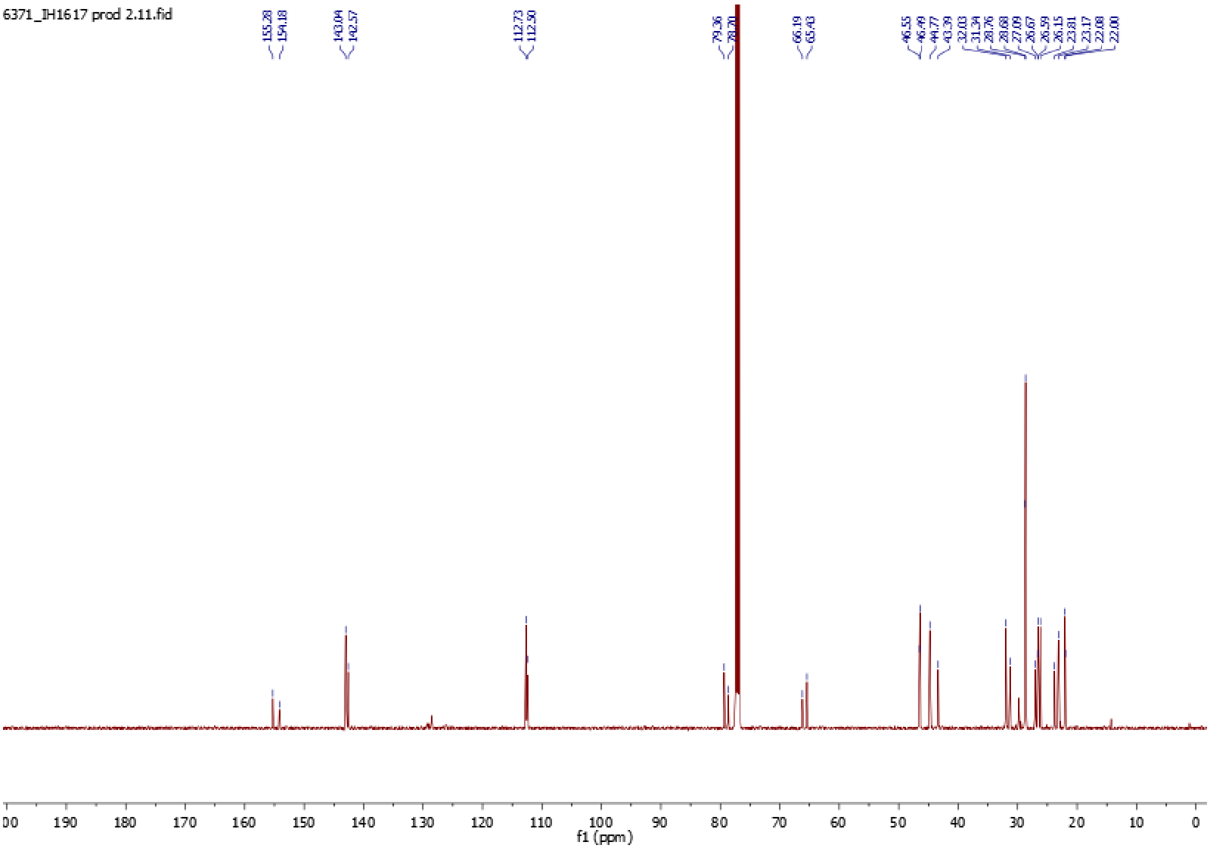
6371_IH1617 prod 2.11.fid
155.28
154.18
143.04
142.57
112.73
112.50
79.36
78.70
66.19
65.43
46.55
46.49
44.77
43.39
32.03
31.34
28.76
28.68
27.09
26.67
26.59
26.15
23.81
23.17
22.08
22.00
f1 (ppm)

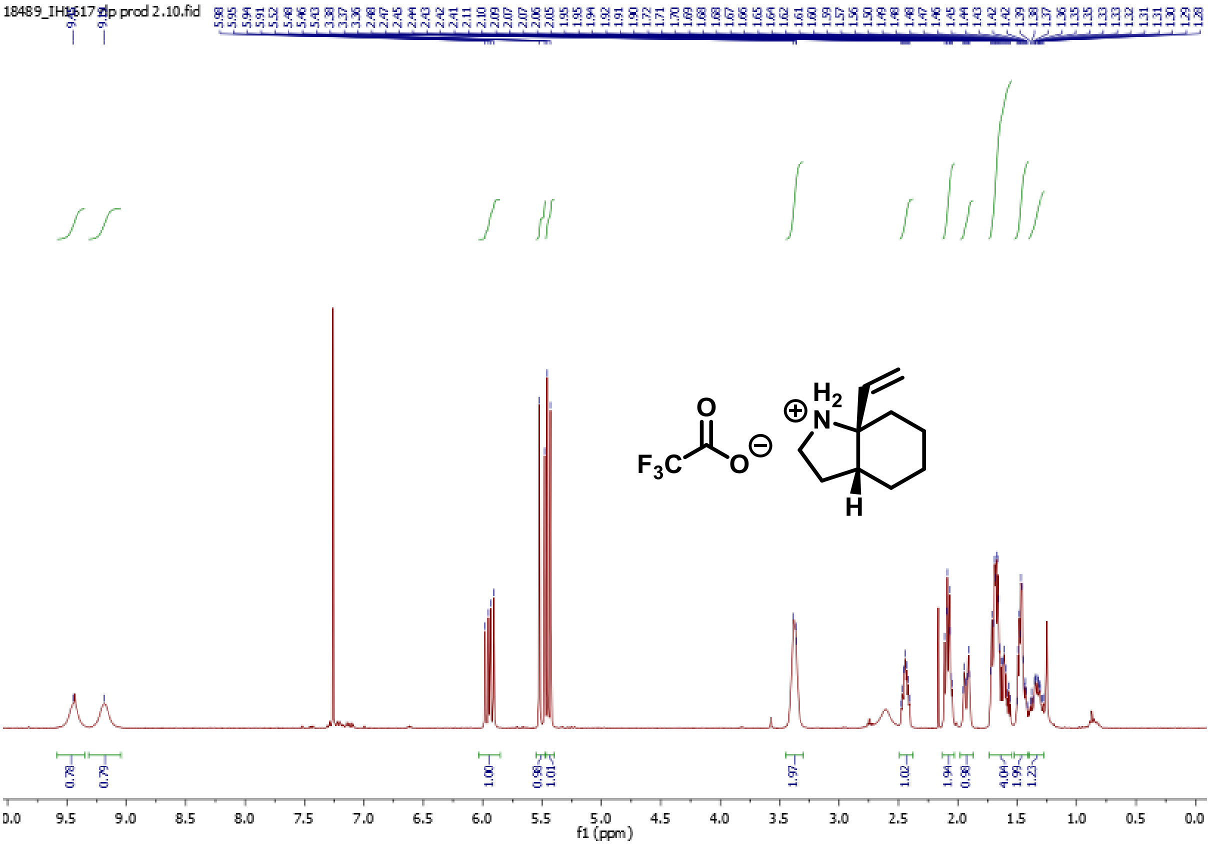
18489_IH1617 dp prod 2.10.fid
0.78
0.79
1.00
0.98
1.01
1.97
1.02
1.94
0.98
4.04
1.99
1.23
f1 (ppm)
O
F3C
O
H2
N
H

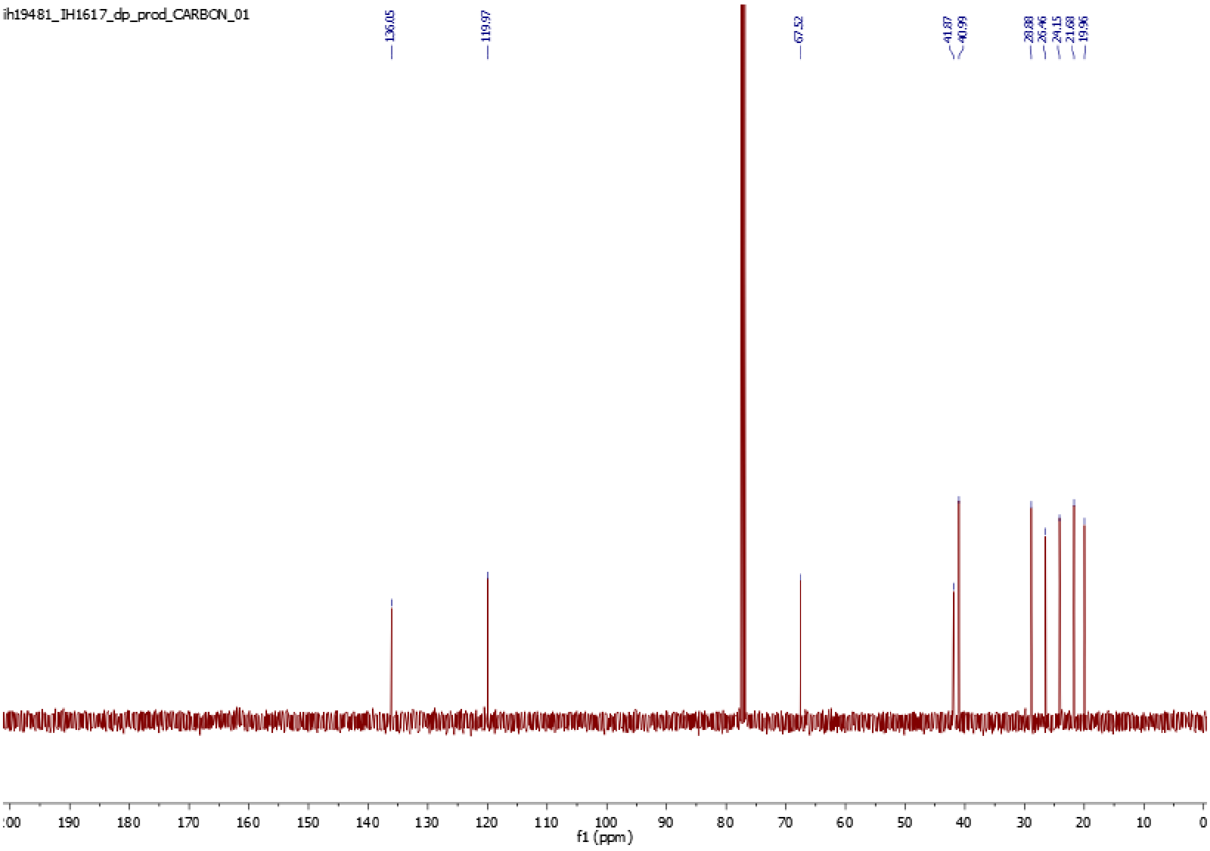
ih19481_IH1617_dp_prod_CARBON_01
136.05
119.97
67.52
41.87
40.99
28.88
26.46
24.15
21.68
19.96
f1 (ppm)

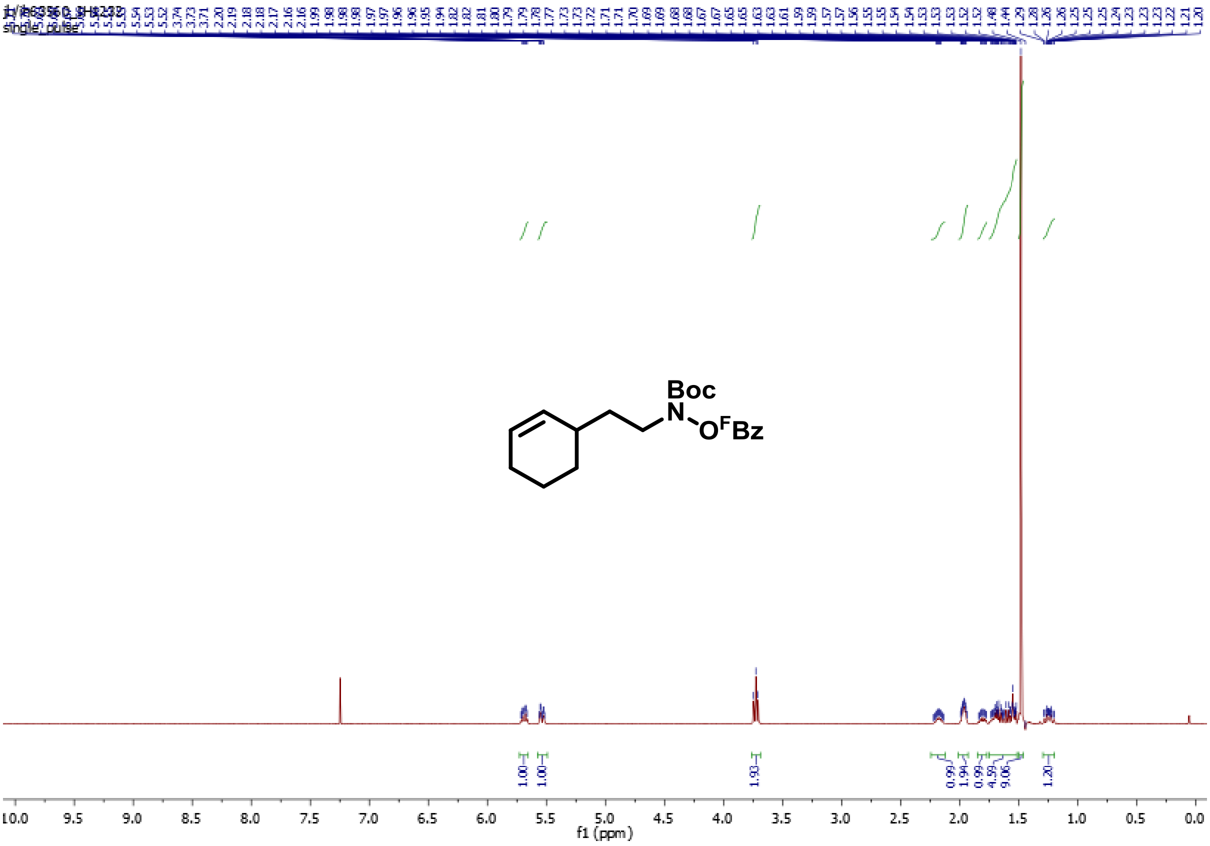
Boc
N
O
FBz
f1 (ppm)

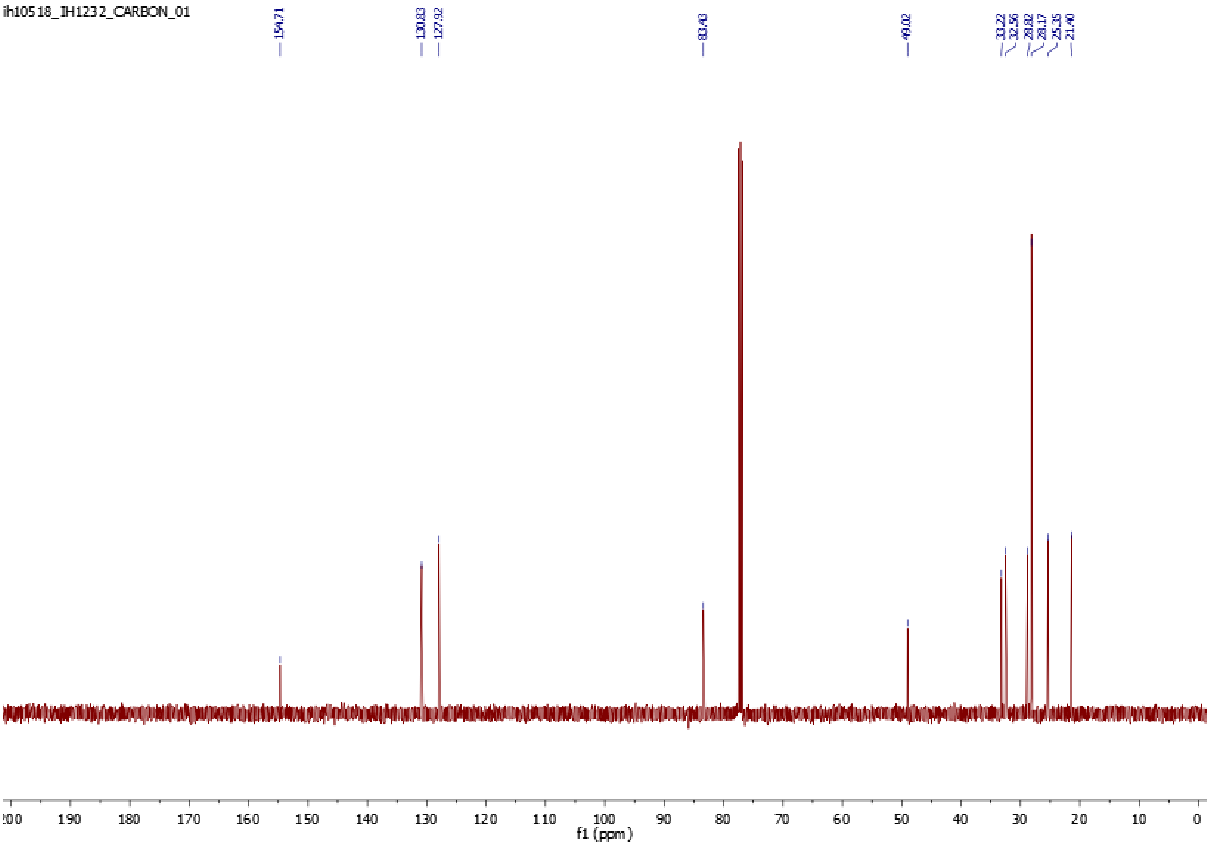
ih10518_IH1232_CARBON_01
154.71
130.83
127.92
83.43
49.02
33.22
32.56
28.82
28.17
25.35
21.40
f1 (ppm)

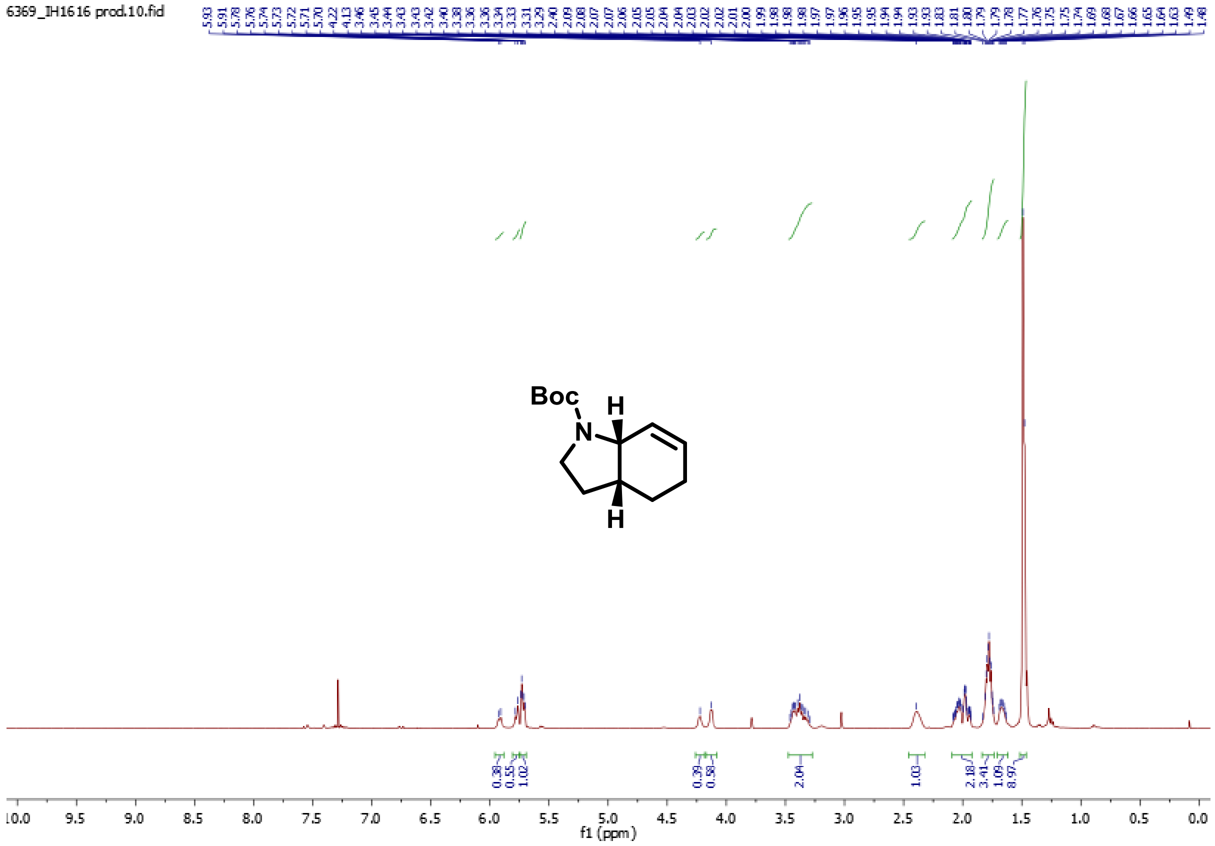
6369_IH1616 prod.10.fid
Boc
N
H
H
f1 (ppm)

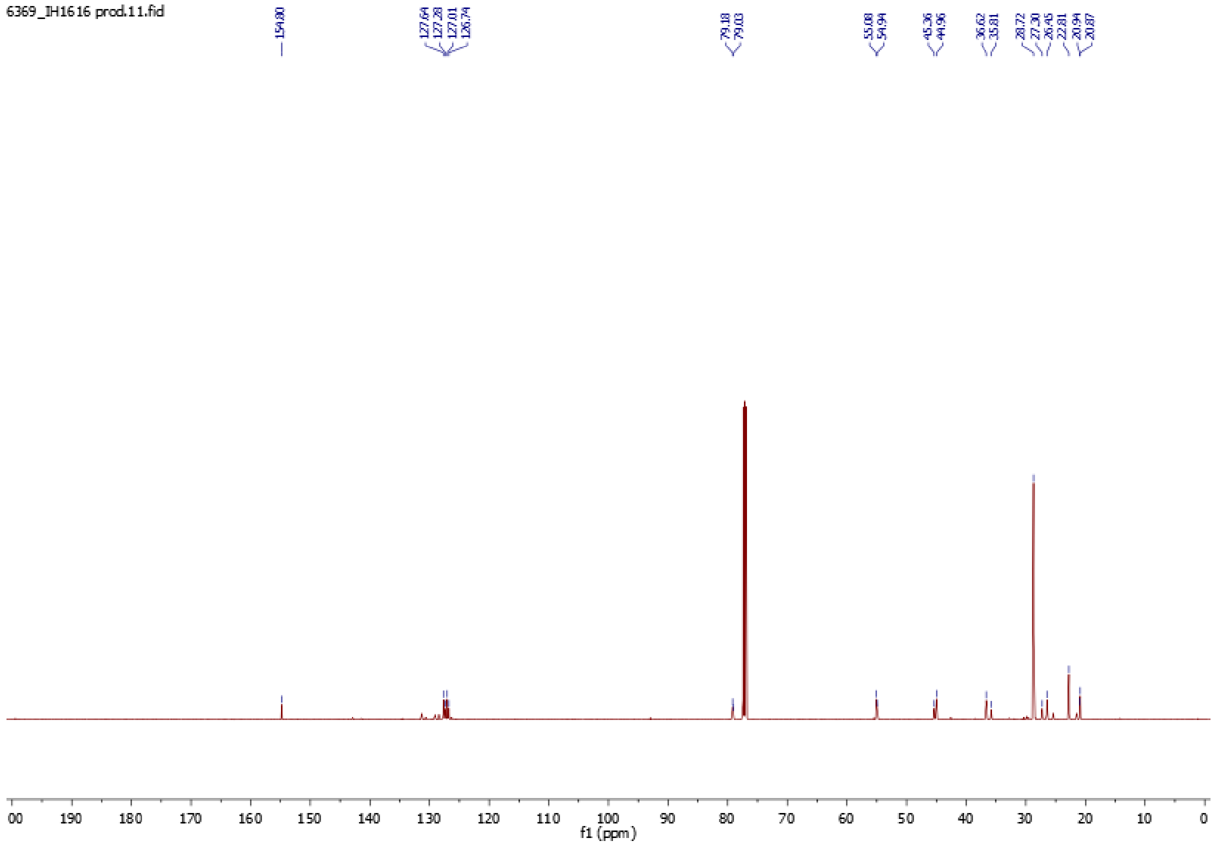
6369_IH1616 prod.11.fid
154.80
127.64
127.28
127.01
126.74
79.18
79.03
55.08
54.94
45.36
44.96
36.62
35.81
28.72
27.30
26.45
22.81
20.94
20.87
f1 (ppm)

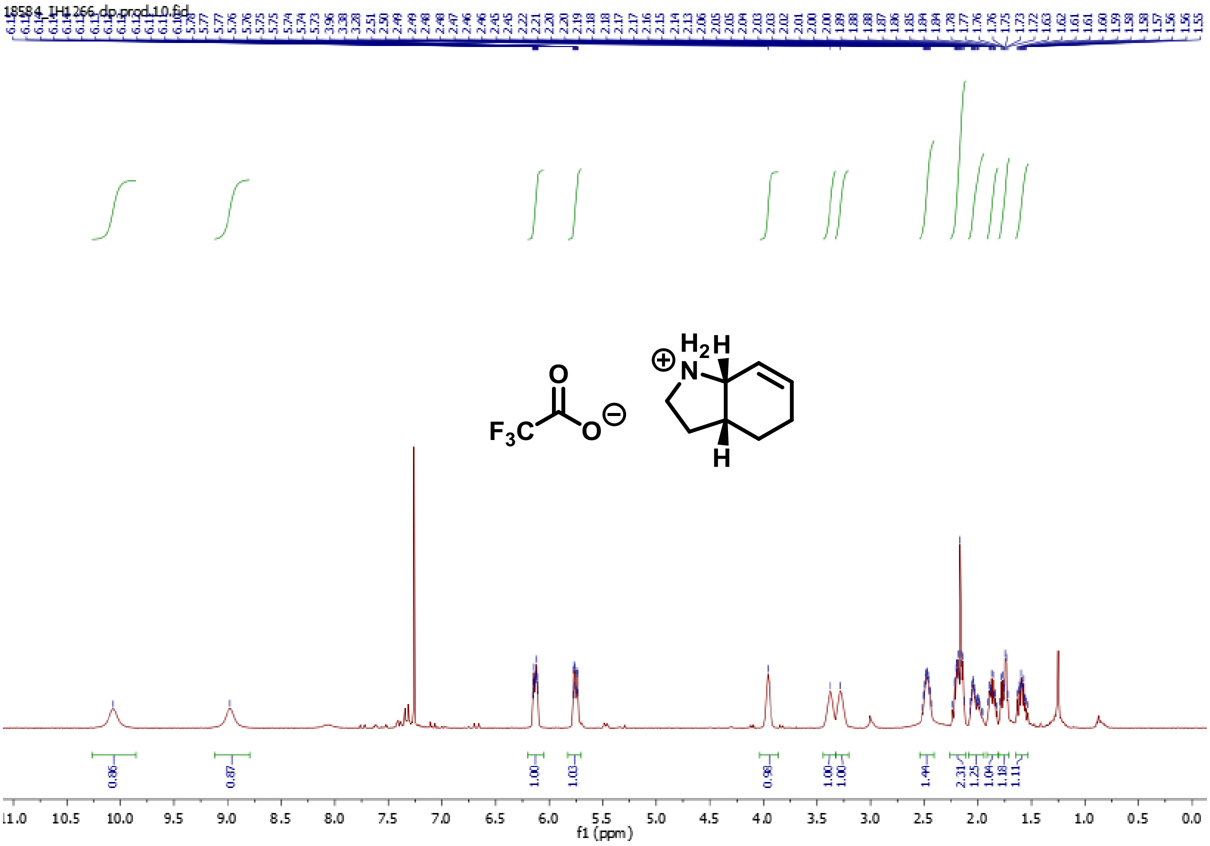
18584_IH1266 dp prod.10.fid
f1 (ppm)

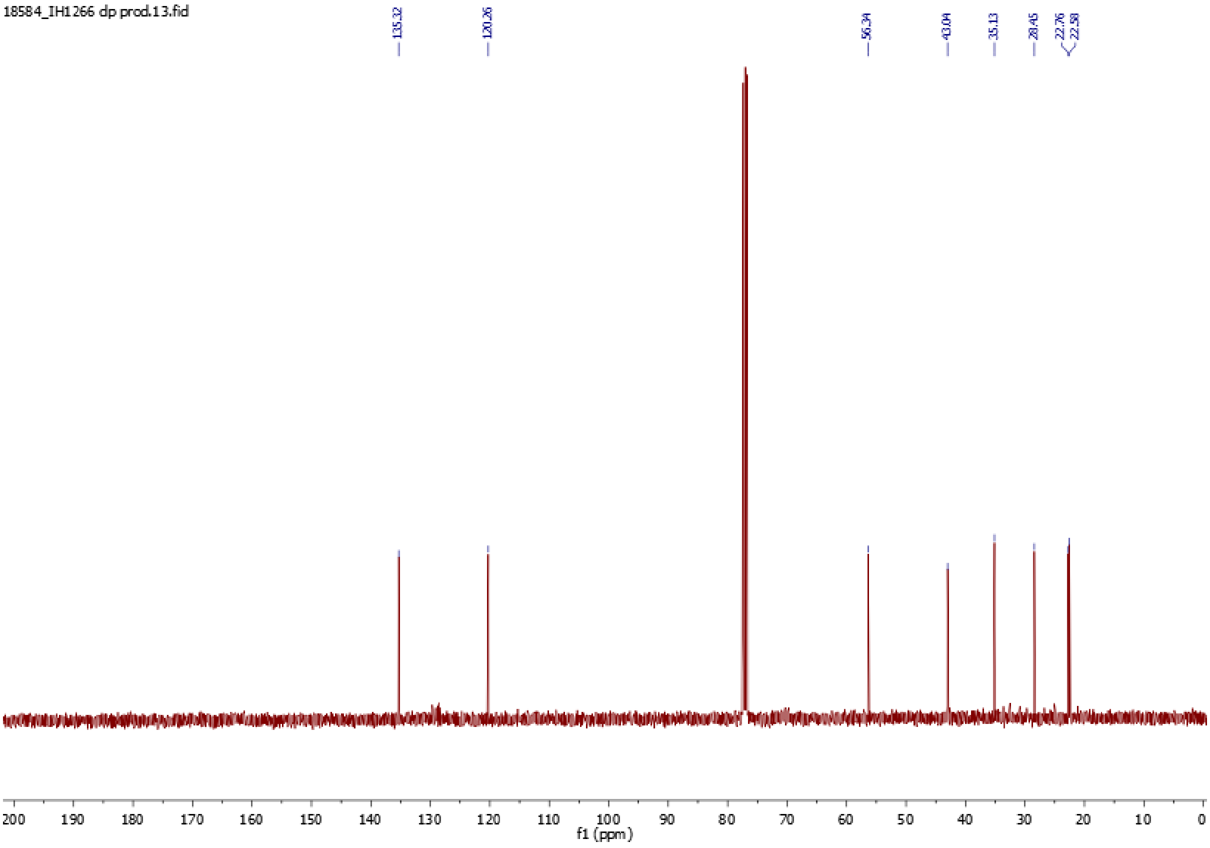
18584_IH1266 dp prod.13.fid
135.32
120.26
56.34
43.09
35.13
28.45
22.76
22.58
f1 (ppm)

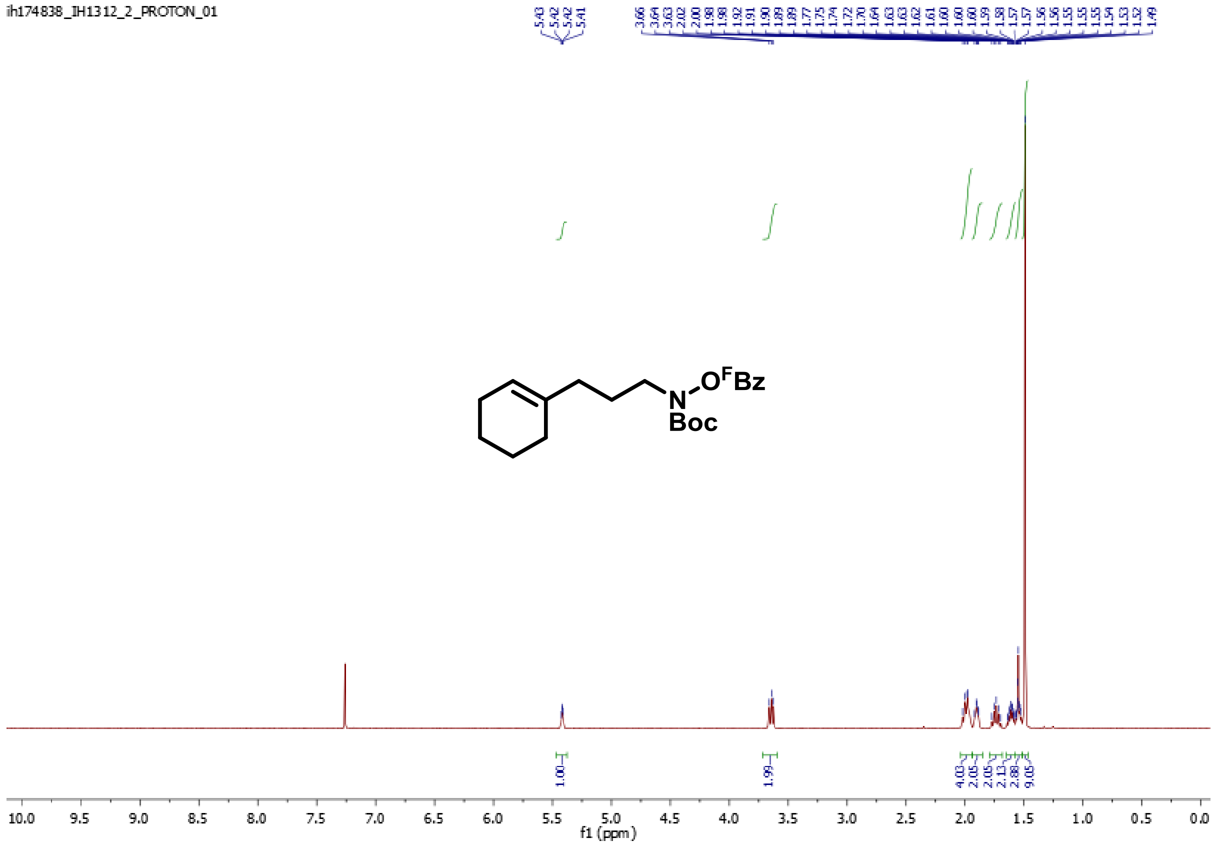
ih174838_IH1312_2_PROTON_01
5.43 5.42 5.42 5.41
3.66 3.64 3.63 2.02 2.00 1.98 1.98 1.92 1.91 1.90 1.89 1.89 1.77 1.75 1.74 1.72 1.70 1.64 1.63 1.63 1.62 1.61 1.60 1.60 1.60 1.59 1.58 1.57 1.57 1.56 1.56 1.55 1.55 1.54 1.53 1.52 1.49
O^FBz
N
Boc
1.00
1.99
4.03 2.05 2.05 2.13 2.88 9.05
10.0 9.5 9.0 8.5 8.0 7.5 7.0 6.5 6.0 5.5 5.0 4.5 4.0 3.5 3.0 2.5 2.0 1.5 1.0 0.5 0.0
f1 (ppm)

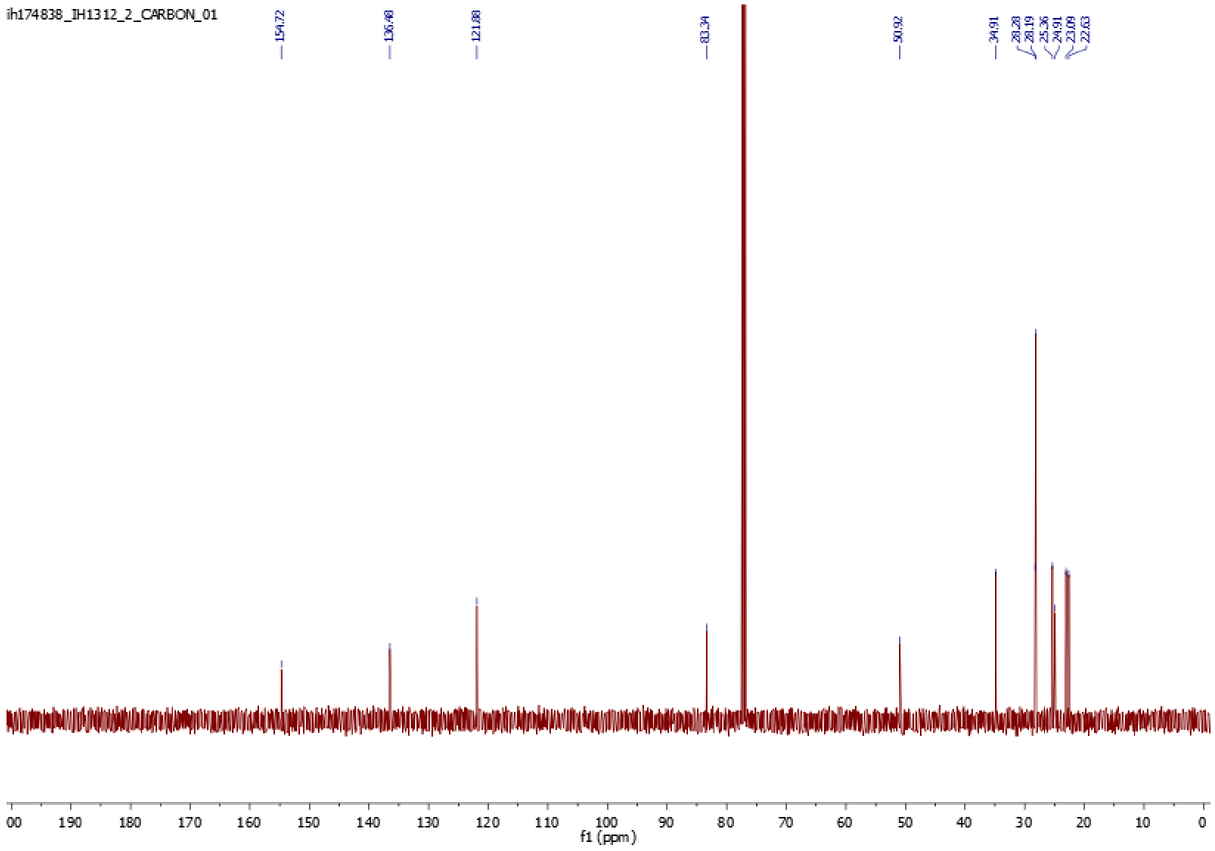
ih174838_IH1312_2_CARBON_01
154.72
136.48
121.88
83.34
50.92
34.91 28.28 28.19 25.36 24.91 23.09 22.63
00 190 180 170 160 150 140 130 120 110 100 90 80 70 60 50 40 30 20 10 0
f1 (ppm)

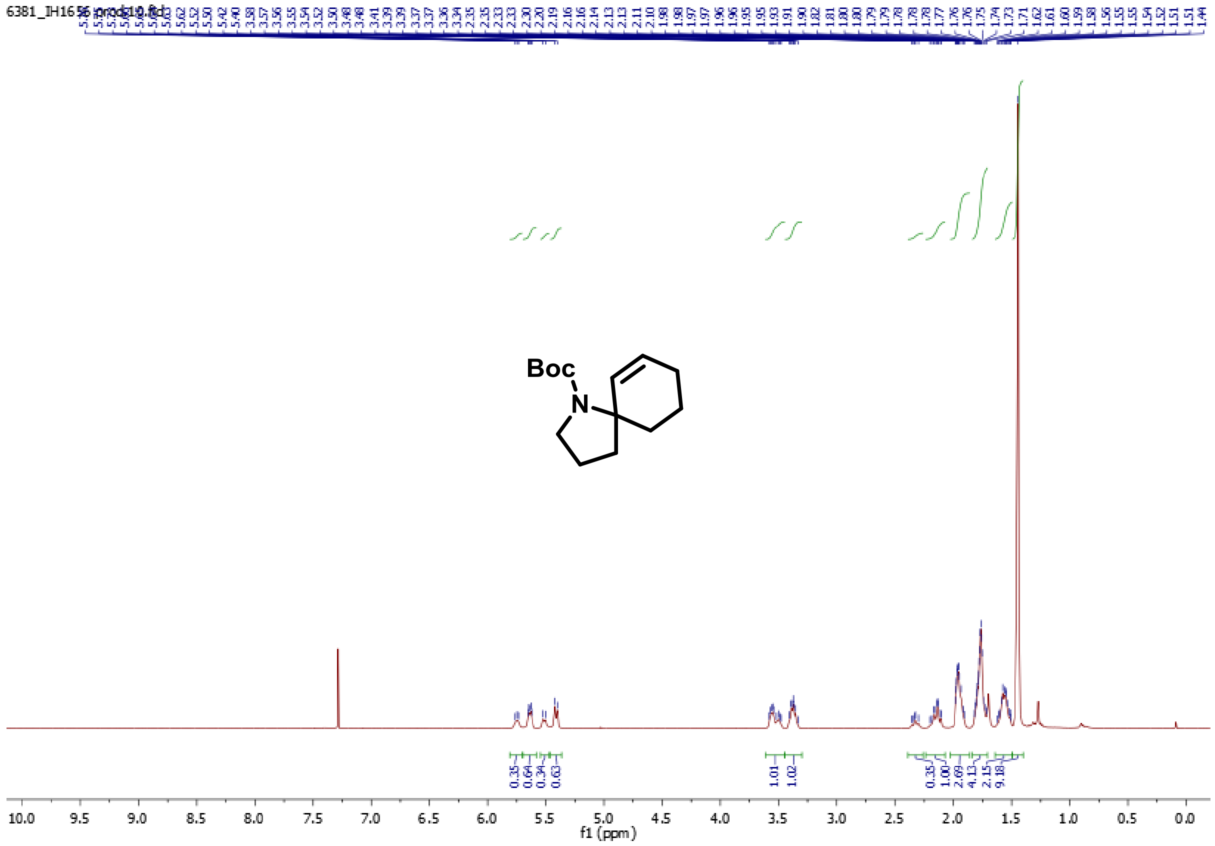
6381_IH1656 prod.10.fid
Boc
N
0.35
0.64
0.34
0.63
1.01
1.02
0.35
1.00
2.69
4.13
2.15
9.18
f1 (ppm)

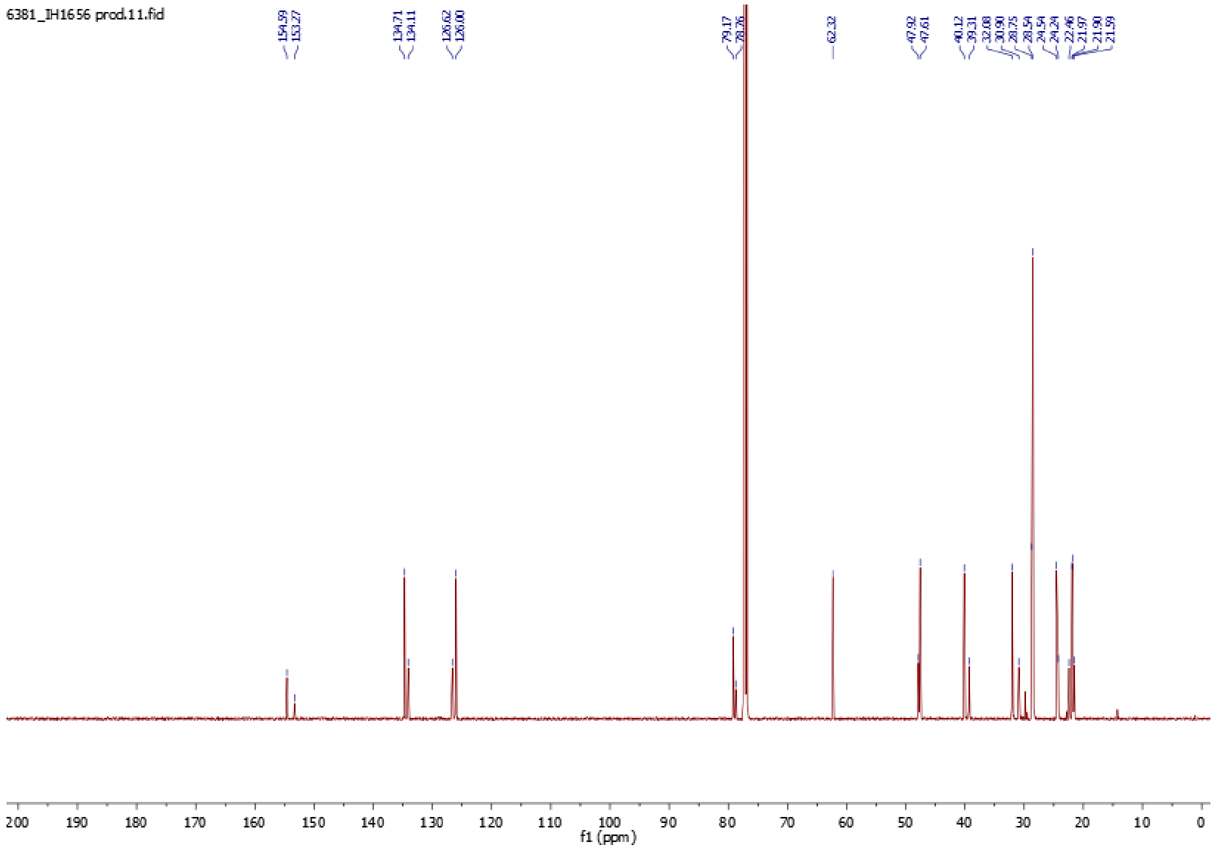
6381_IH1656 prod.11.fid
154.99
153.27
134.71
134.11
126.62
126.00
79.17
78.76
62.32
47.92
47.61
40.12
39.31
32.08
30.90
28.75
28.54
24.54
24.24
22.46
21.97
21.90
21.59
f1 (ppm)

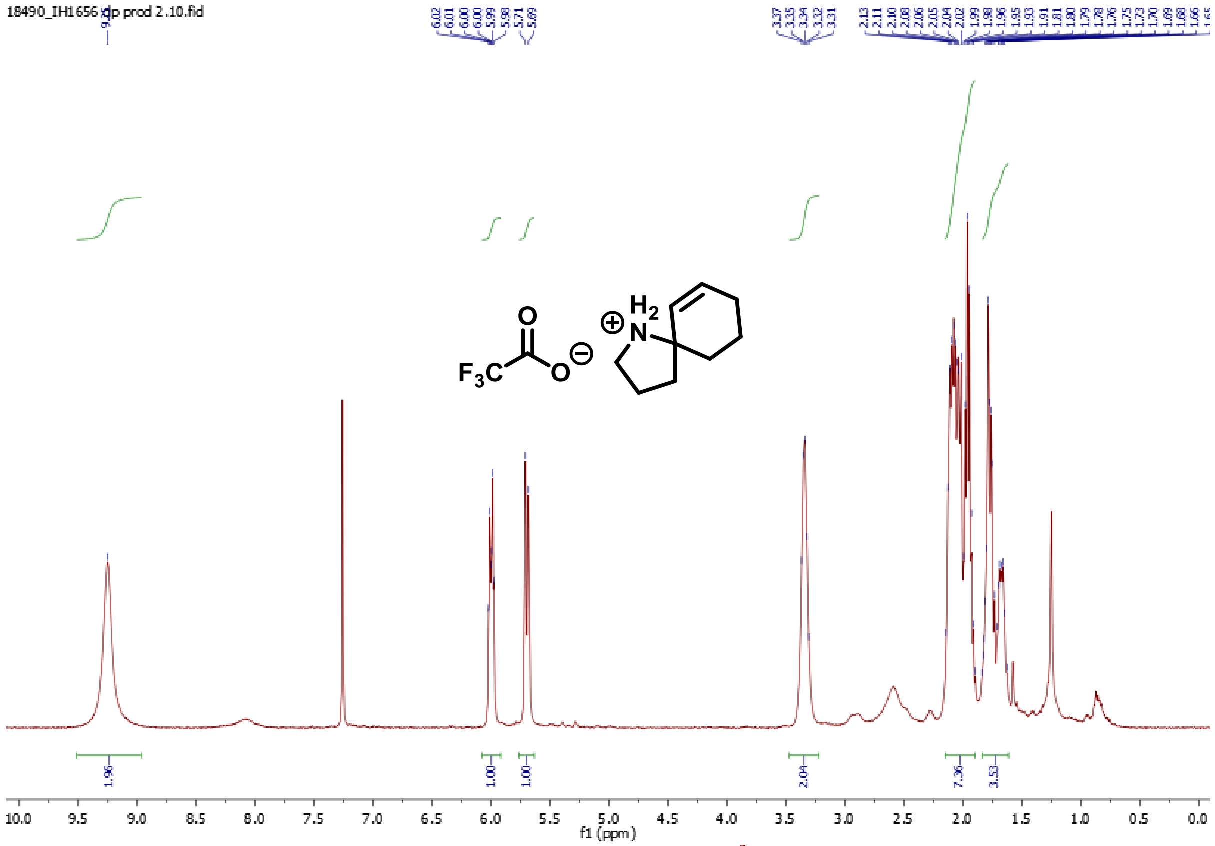
18490_IH1656 dp prod 2.10.fid
f1 (ppm)

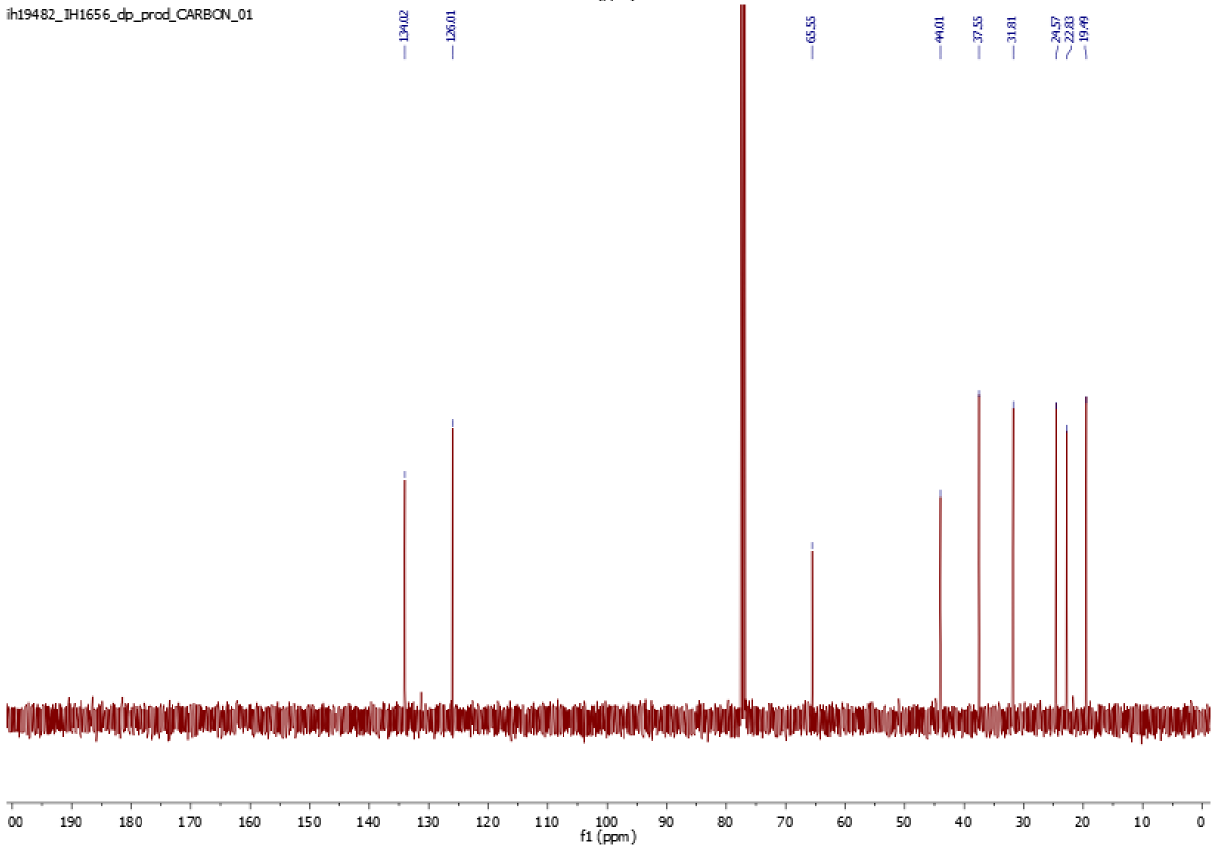
ih19482_IH1656_dp_prod_CARBON_01
134.02
126.01
65.55
44.01
37.55
31.81
24.57
22.83
19.49
f1 (ppm)

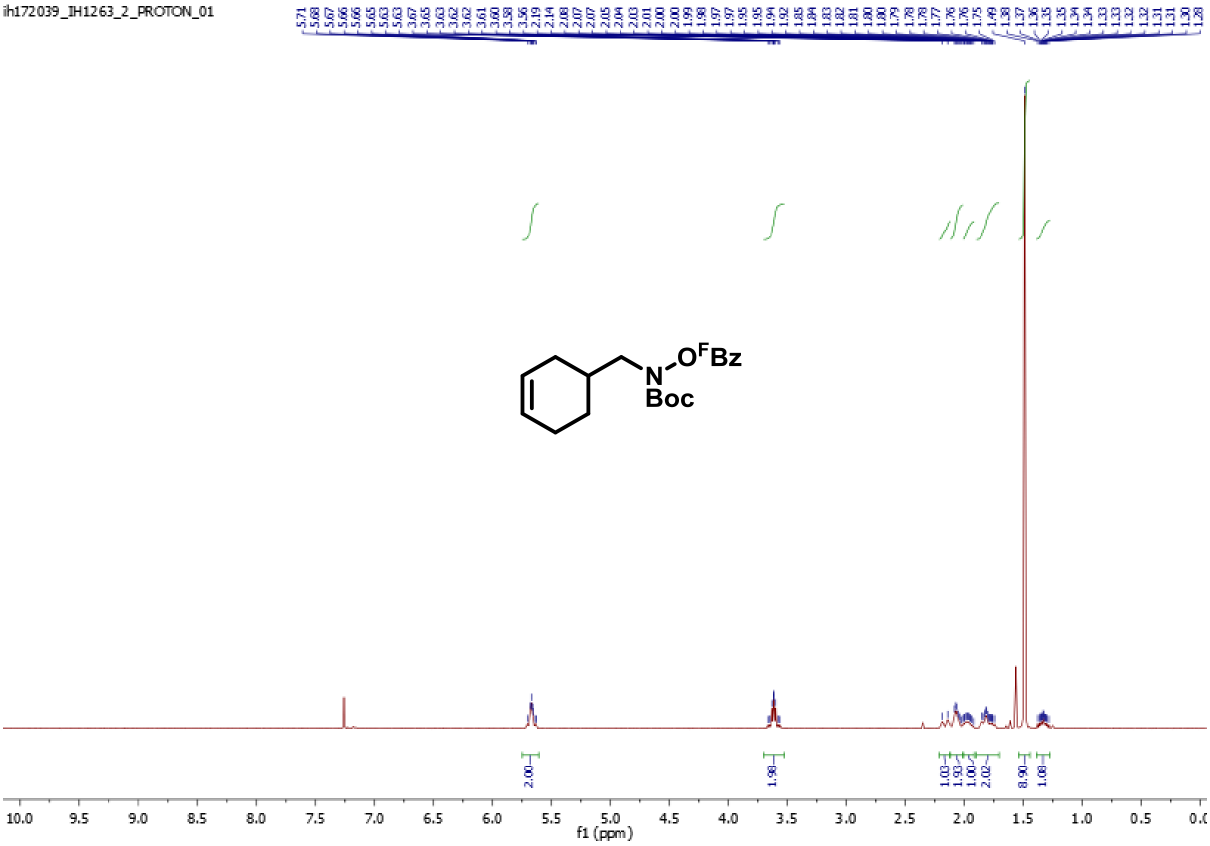
ih172039_IH1263_2_PROTON_01
5.71 5.68 5.67 5.66 5.66 5.65 5.63 5.63 3.67 3.65 3.63 3.62 3.62 3.61 3.60 3.58 3.56 2.19 2.14 2.08 2.07 2.07 2.05 2.04 2.03 2.01 2.00 2.00 1.99 1.98 1.97 1.97 1.95 1.95 1.94 1.92 1.85 1.84 1.83 1.82 1.81 1.80 1.80 1.79 1.78 1.78 1.77 1.76 1.76 1.75 1.49 1.38 1.37 1.36 1.35 1.35 1.34 1.34 1.33 1.33 1.32 1.32 1.31 1.31 1.30 1.28
N
O
F
Bz
Boc
2.00
1.98
1.03
1.93
1.00
2.02
8.90
1.08
10.0 9.5 9.0 8.5 8.0 7.5 7.0 6.5 6.0 5.5 5.0 4.5 4.0 3.5 3.0 2.5 2.0 1.5 1.0 0.5
f1 (ppm)

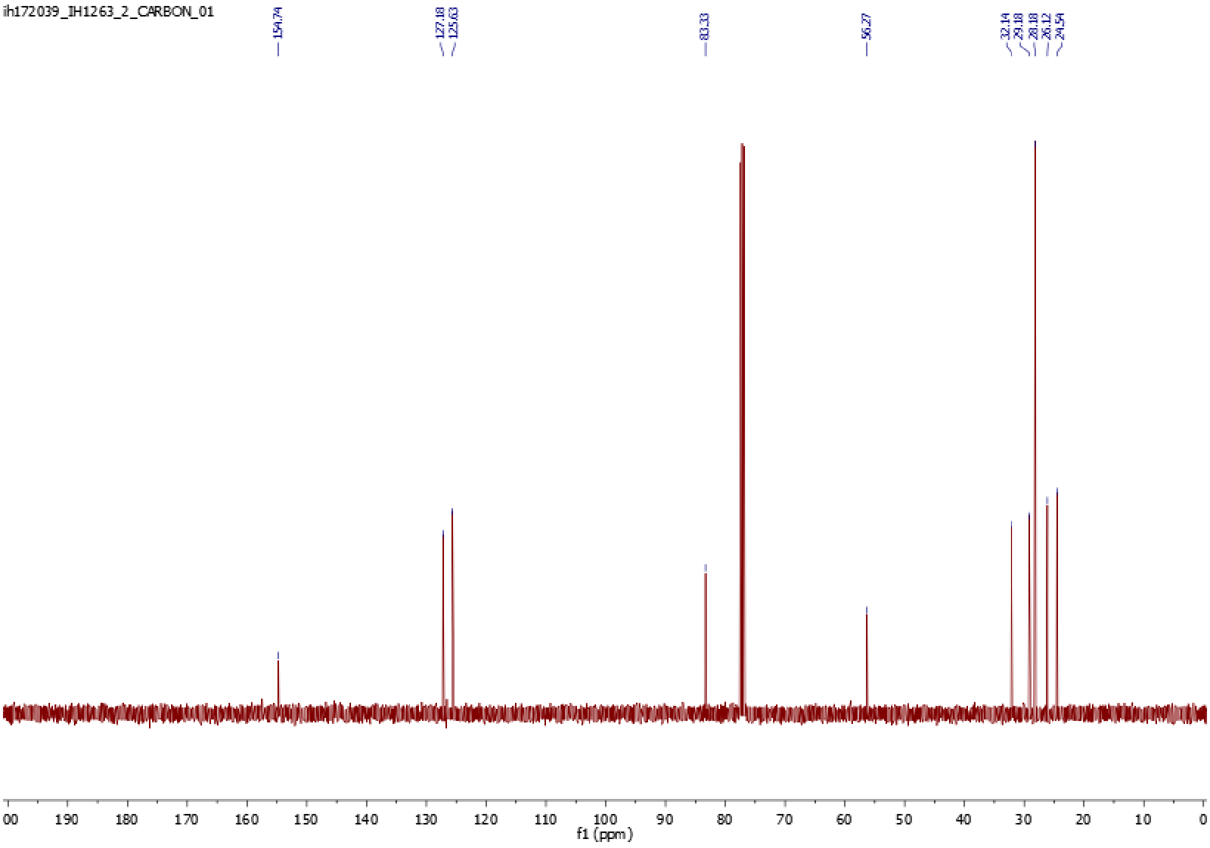
ih172039_IH1263_2_CARBON_01
154.74
127.18
125.63
83.33
56.27
32.14
29.18
28.18
26.12
24.54
190 180 170 160 150 140 130 120 110 100 90 80 70 60 50 40 30 20 10 0
f1 (ppm)

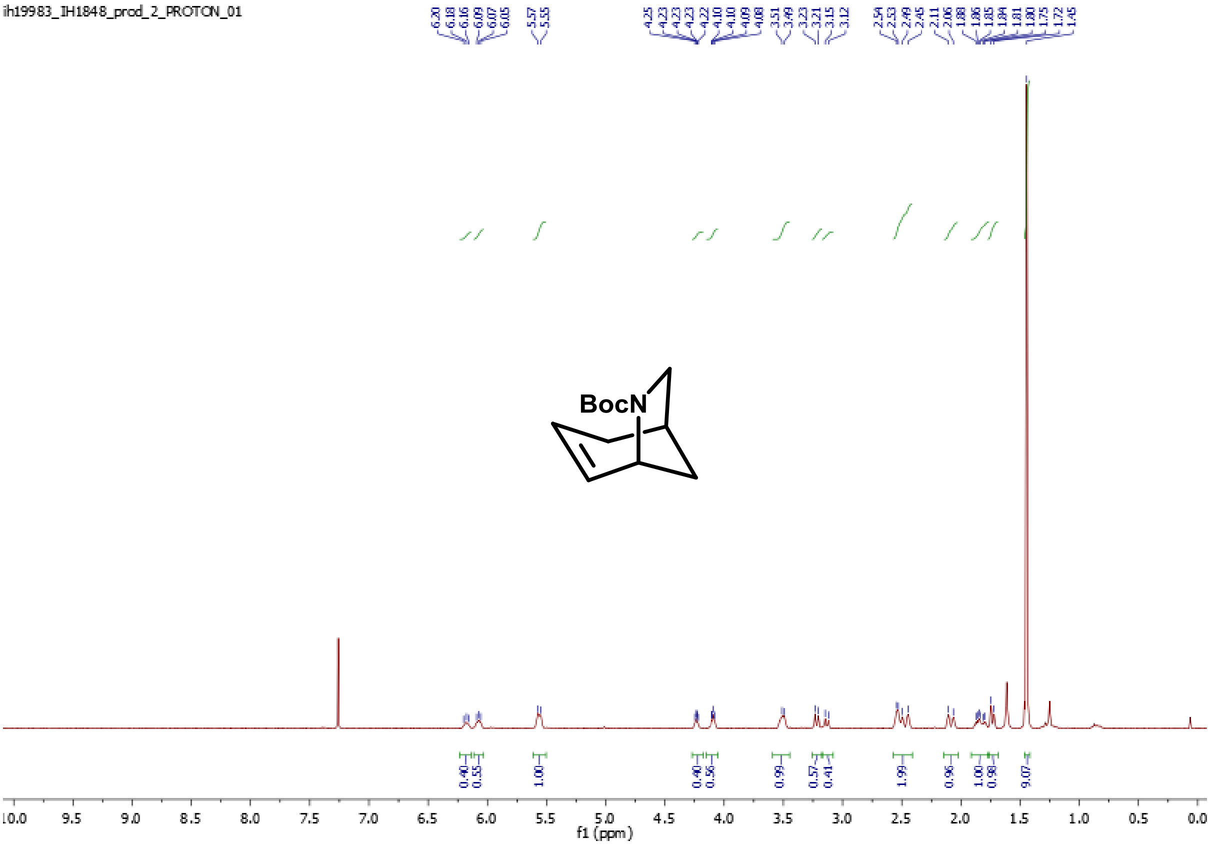
ih19983_IH1848_prod_2_PROTON_01
BocN
f1 (ppm)

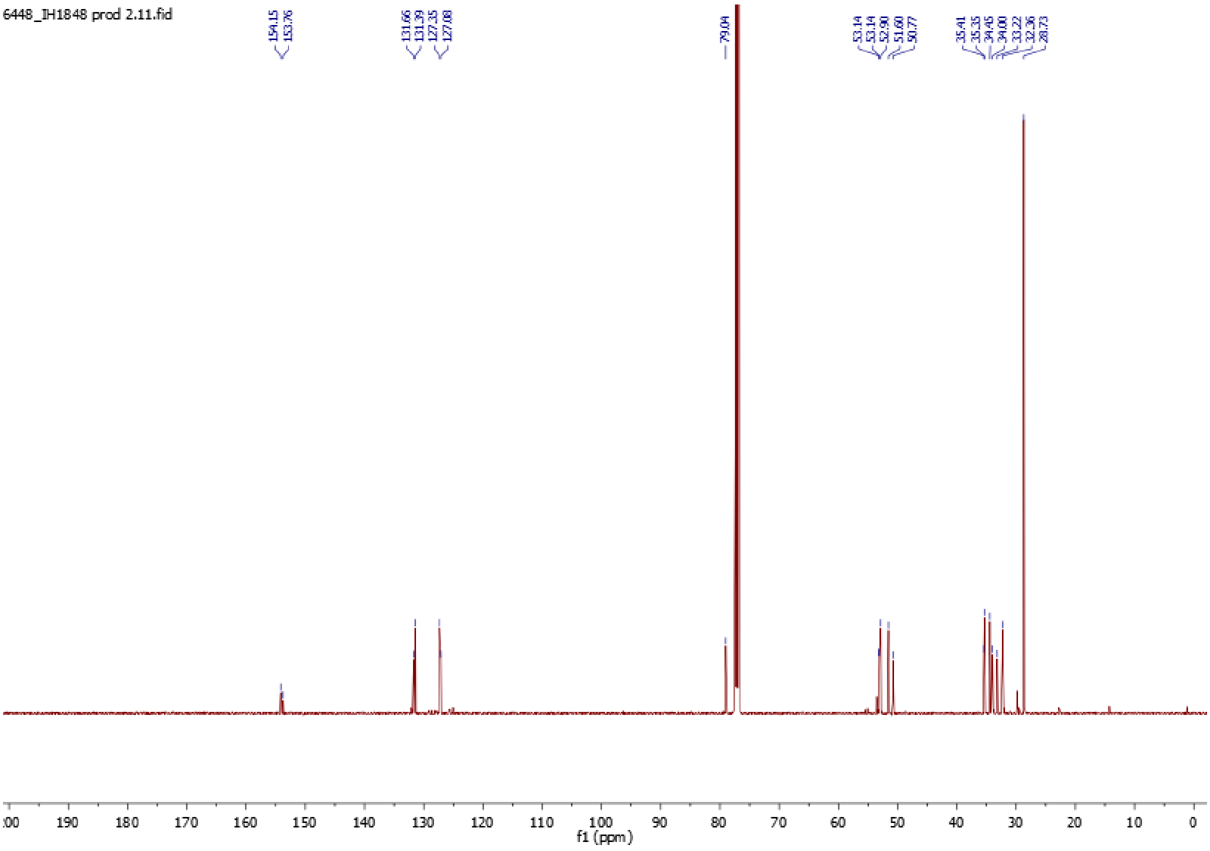
6448_IH1848 prod 2.11.fid
f1 (ppm)

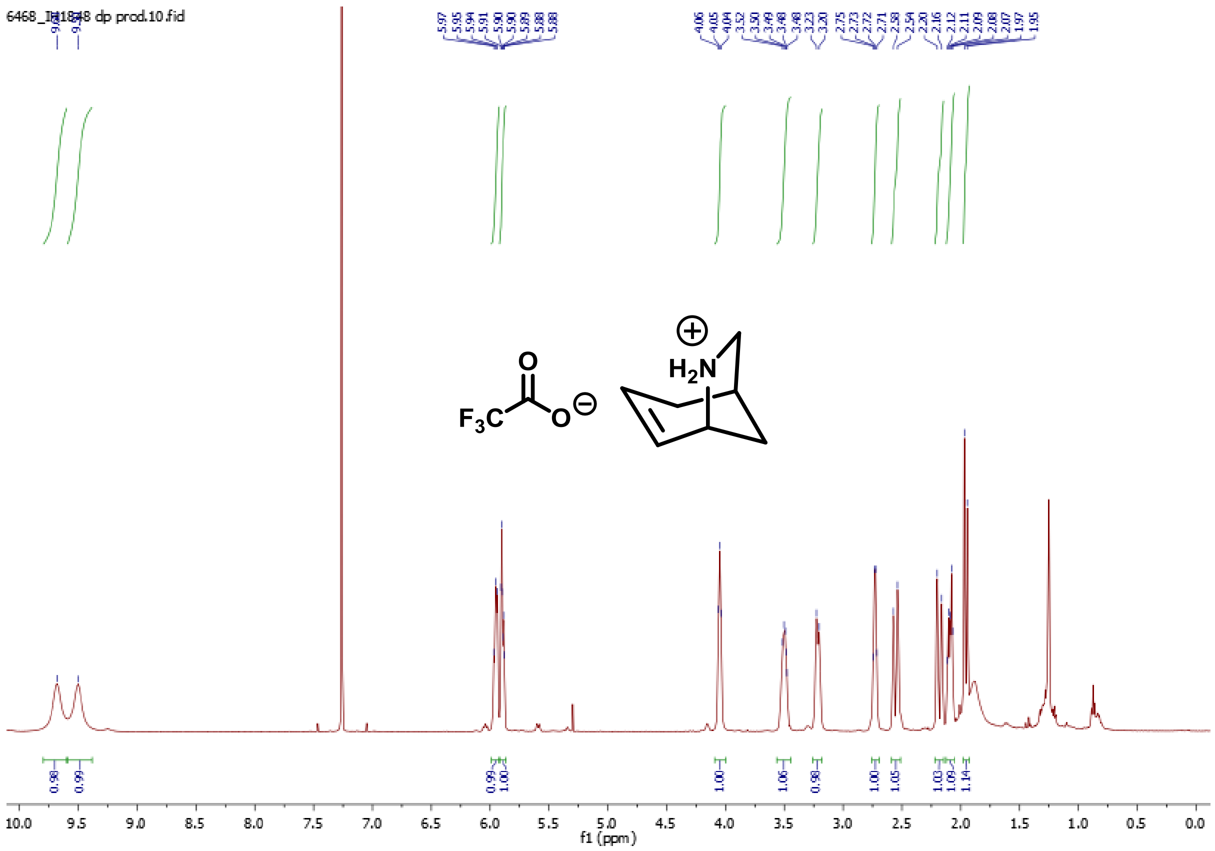
6468_IH1848 dp prod.10.fid
H2N
F3C
O
f1 (ppm)

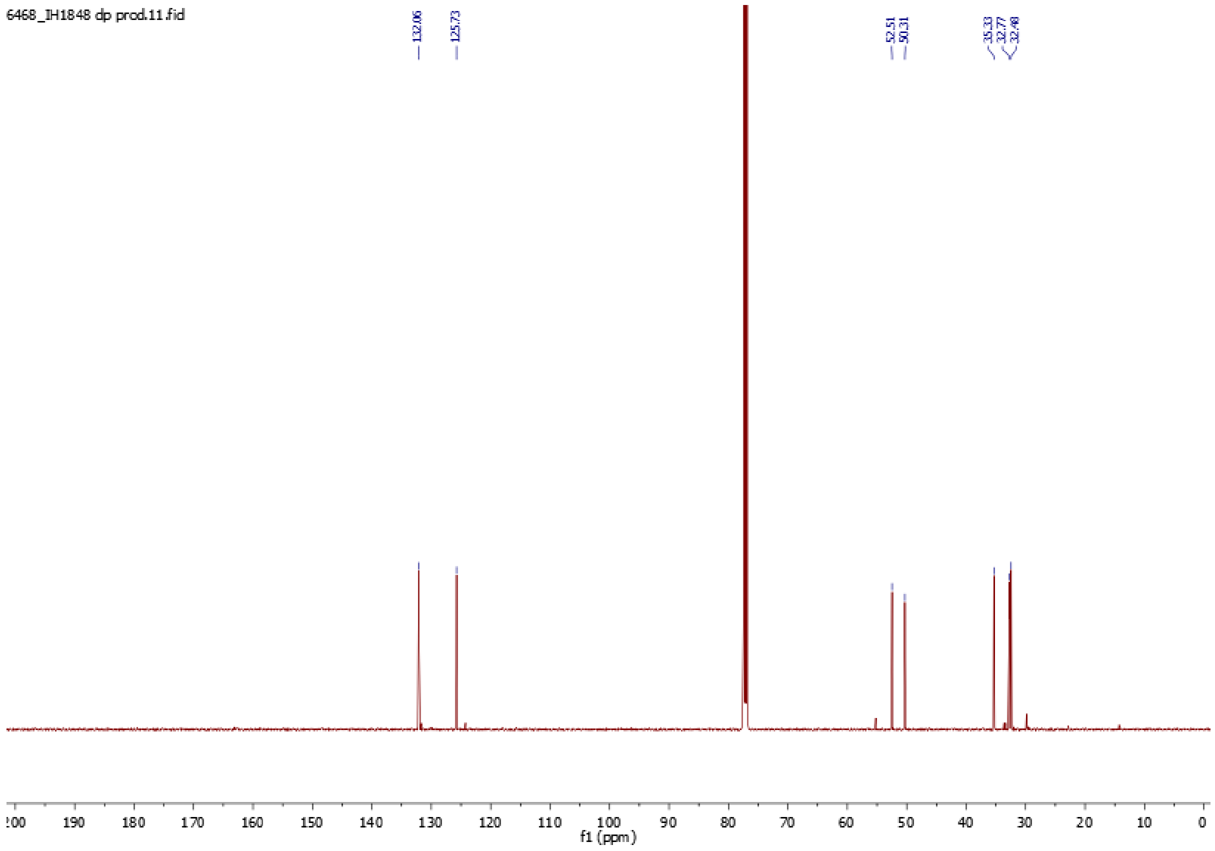
6468_IH1848 dp prod.11.fid
132.06
125.73
52.51
50.31
35.33
32.77
32.49
f1 (ppm)

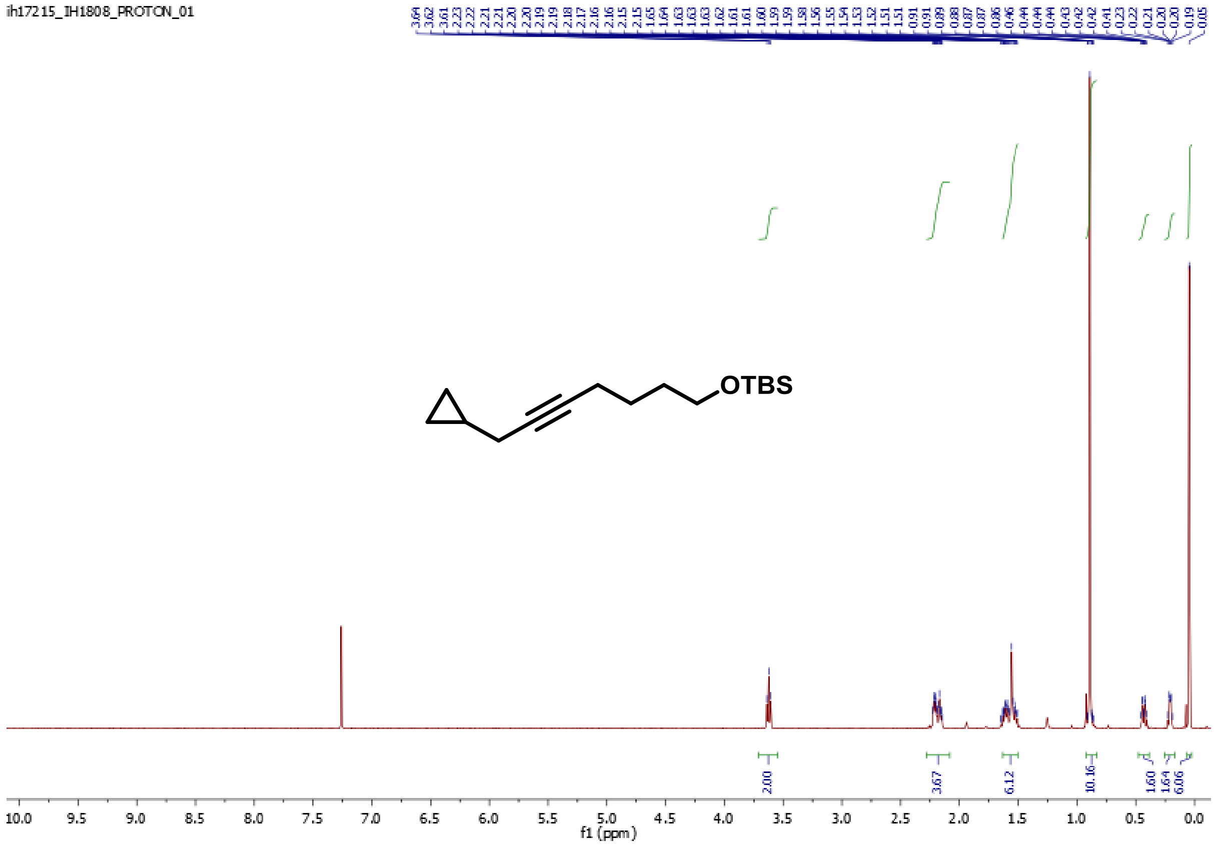
ih17215_IH1808_PROTON_01
OTBS
2.00
3.67
6.12
10.16
1.60
1.64
6.06
f1 (ppm)

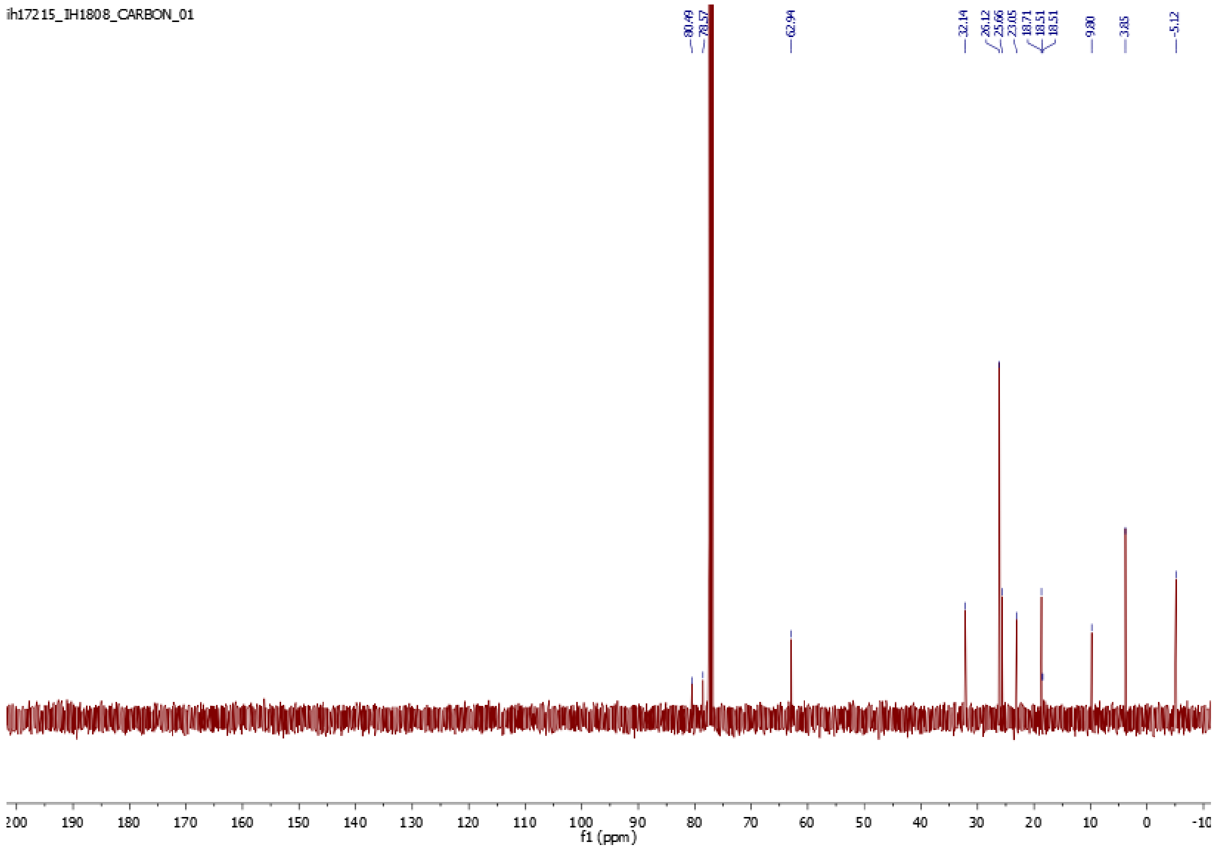
ih17215_IH1808_CARBON_01
80.49
78.57
62.94
32.14
26.12
25.66
23.05
18.71
18.51
18.51
9.80
3.85
-5.12
f1 (ppm)

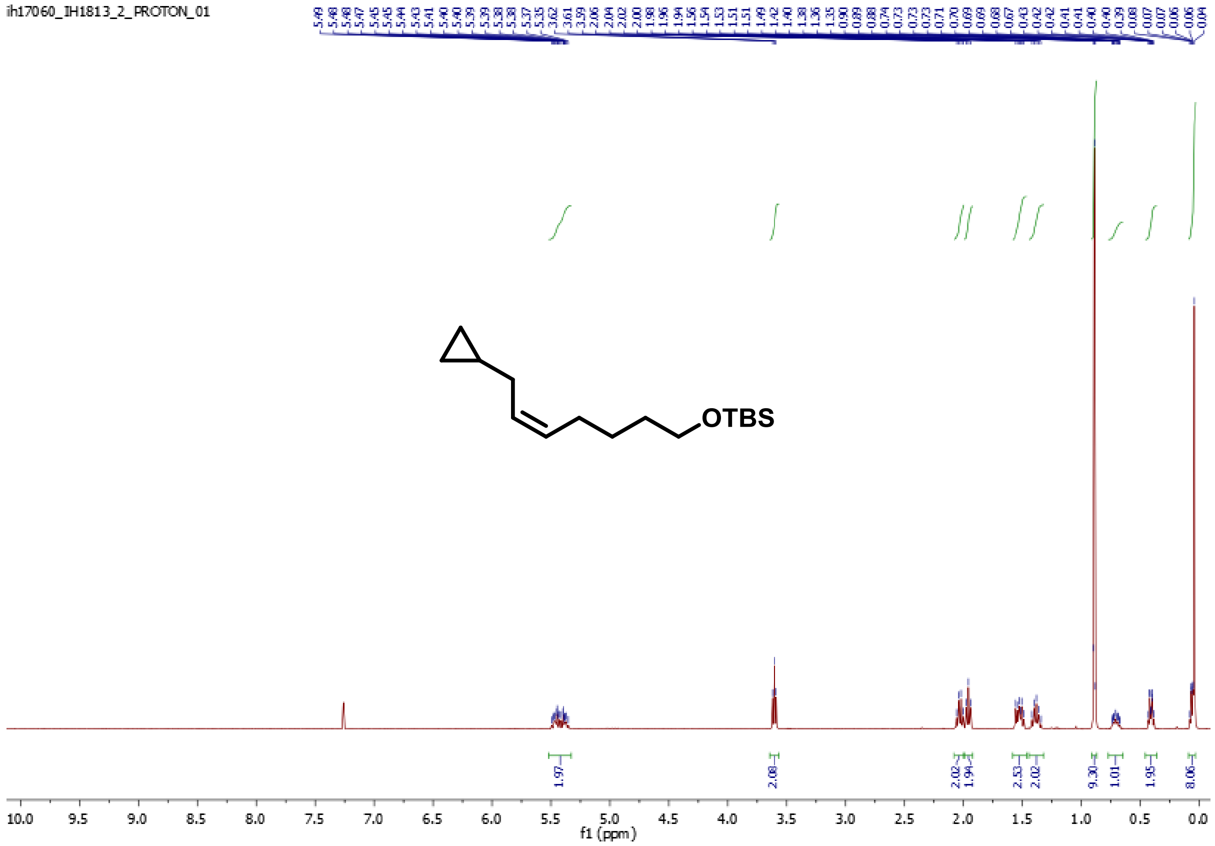
ih17060_IH1813_2_PROTON_01
OTBS
1.97
2.08
2.02
1.94
2.53
2.02
9.30
1.01
1.95
8.06
f1 (ppm)

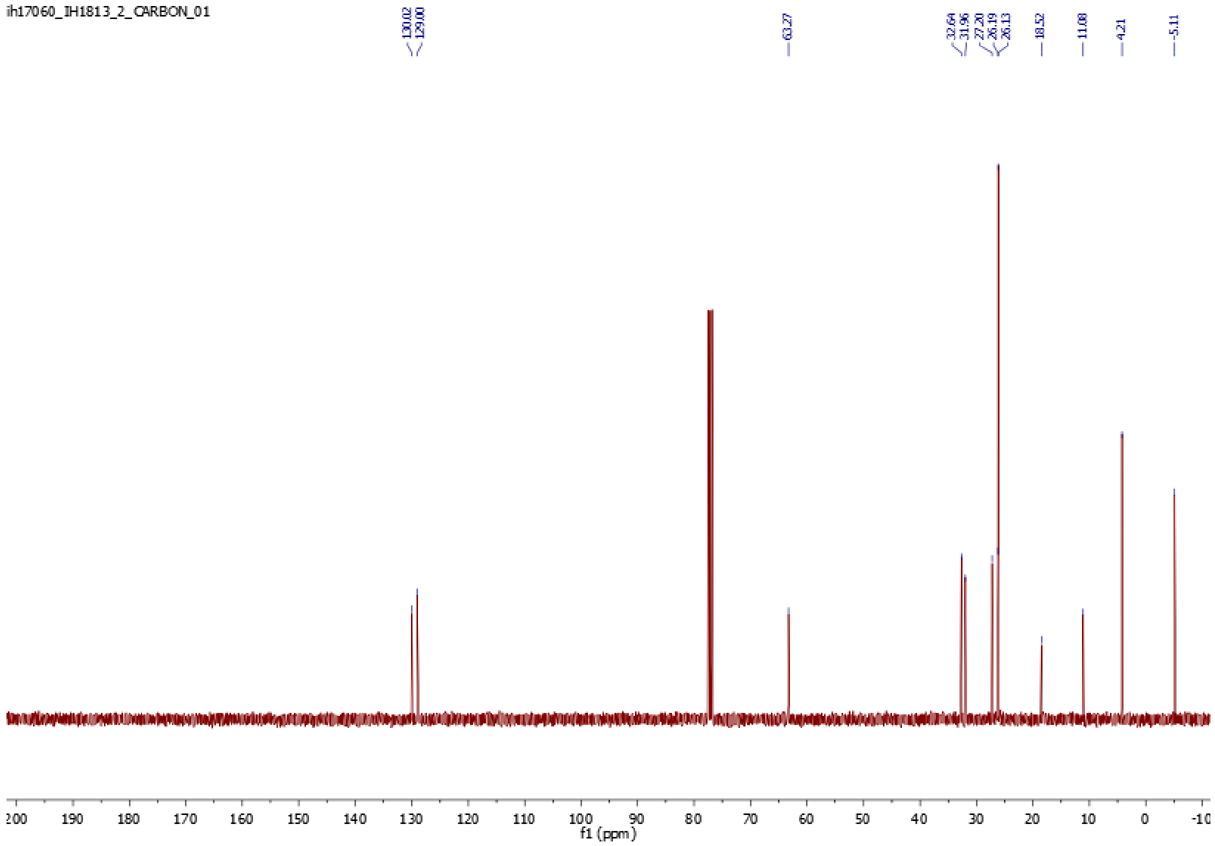
ih17060_IH1813_2_CARBON_01
130.02
129.00
63.27
32.64
31.96
27.20
26.19
26.13
18.52
11.08
4.21
-5.11
f1 (ppm)

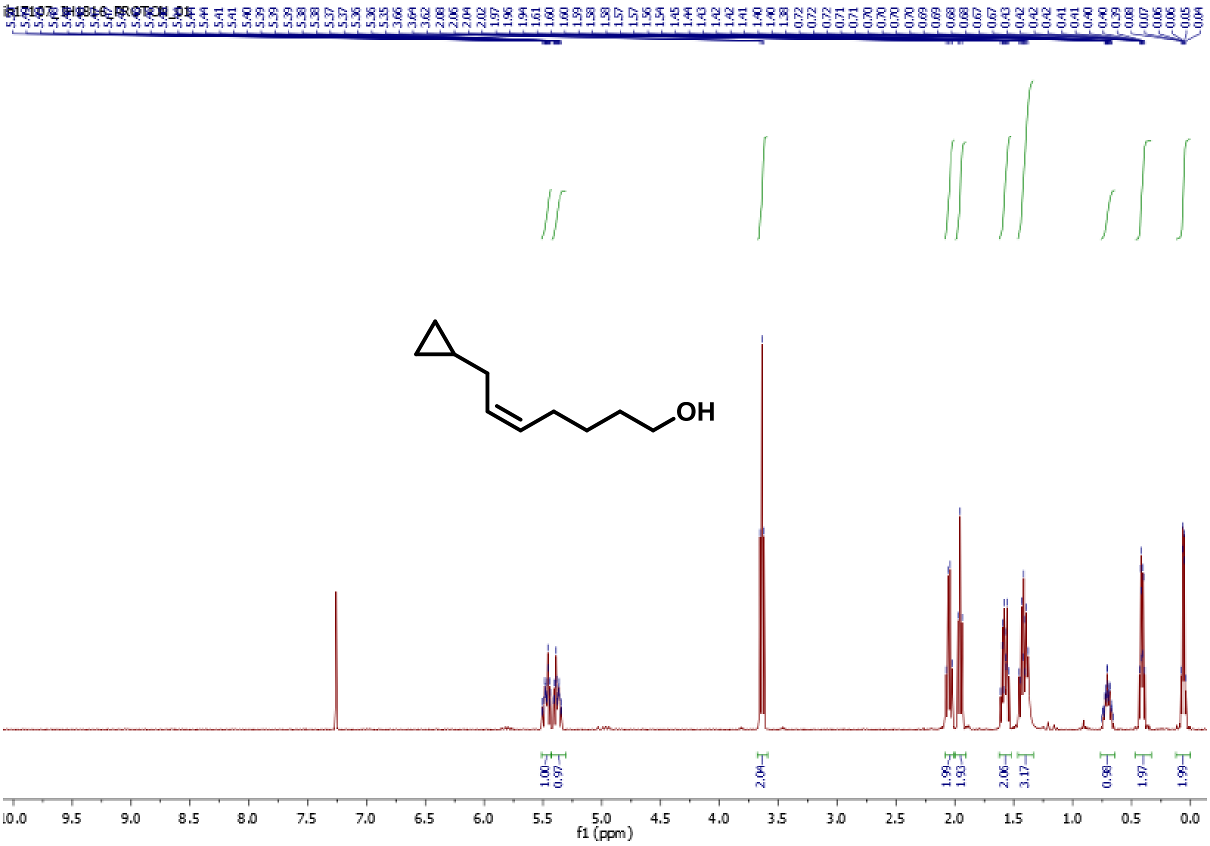
OH
1.00
0.97
2.04
1.99
1.93
2.06
3.17
0.98
1.97
1.99
f1 (ppm)

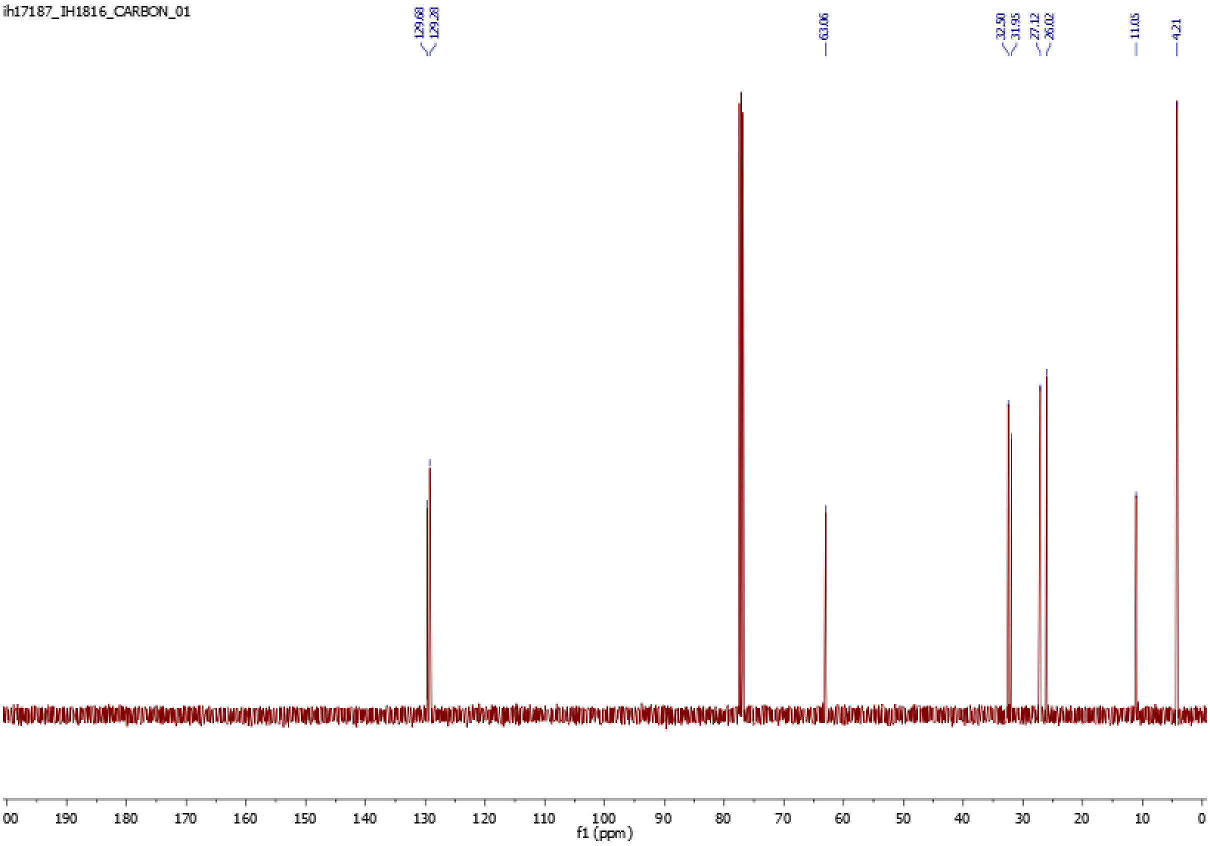
ih17187_IH1816_CARBON_01
129.68
129.28
63.06
32.50
31.95
27.12
26.02
11.05
4.21
f1 (ppm)

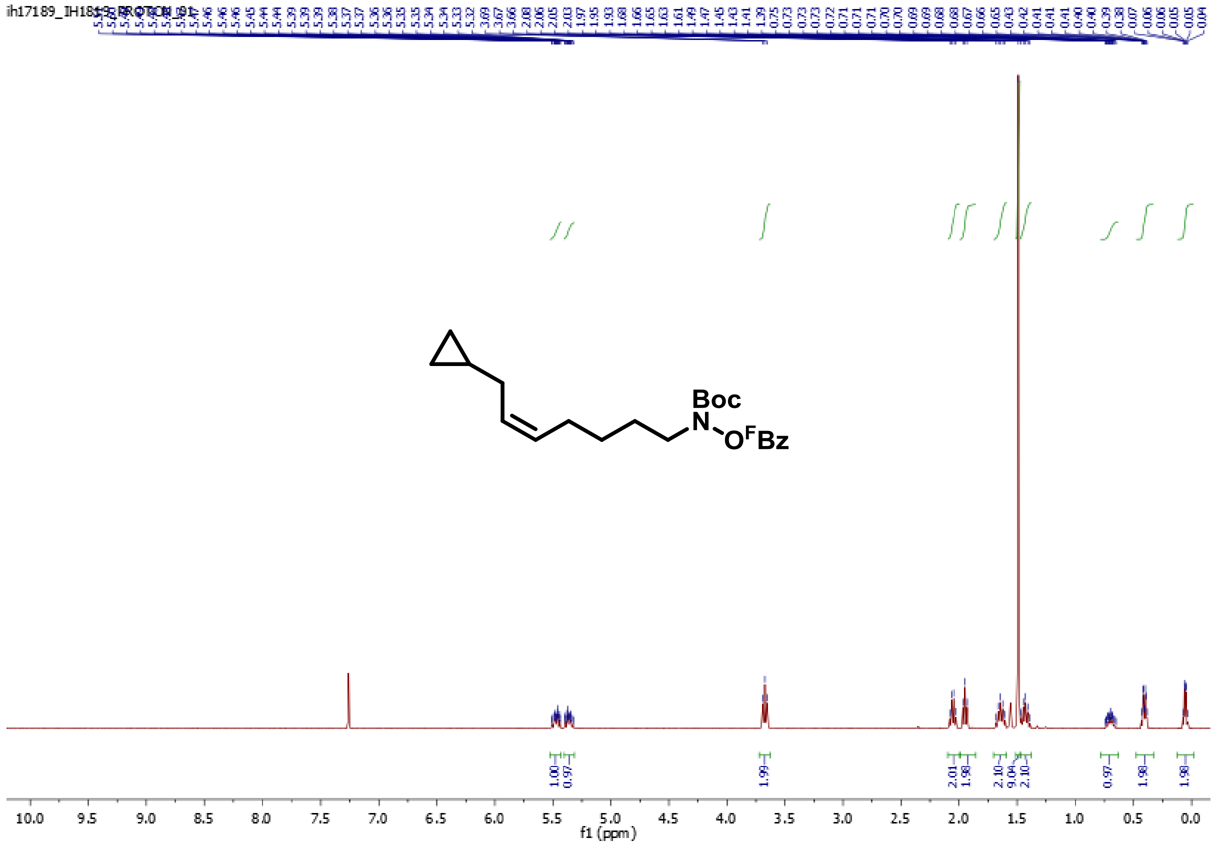
ih17189_IH1819_PROTON_01
Boc
N
O
FBz
1.00
0.97
1.99
2.01
1.98
2.10
9.04
2.10
0.97
1.98
1.98
10.0 9.5 9.0 8.5 8.0 7.5 7.0 6.5 6.0 5.5 5.0 4.5 4.0 3.5 3.0 2.5 2.0 1.5 1.0 0.5 0.0
f1 (ppm)

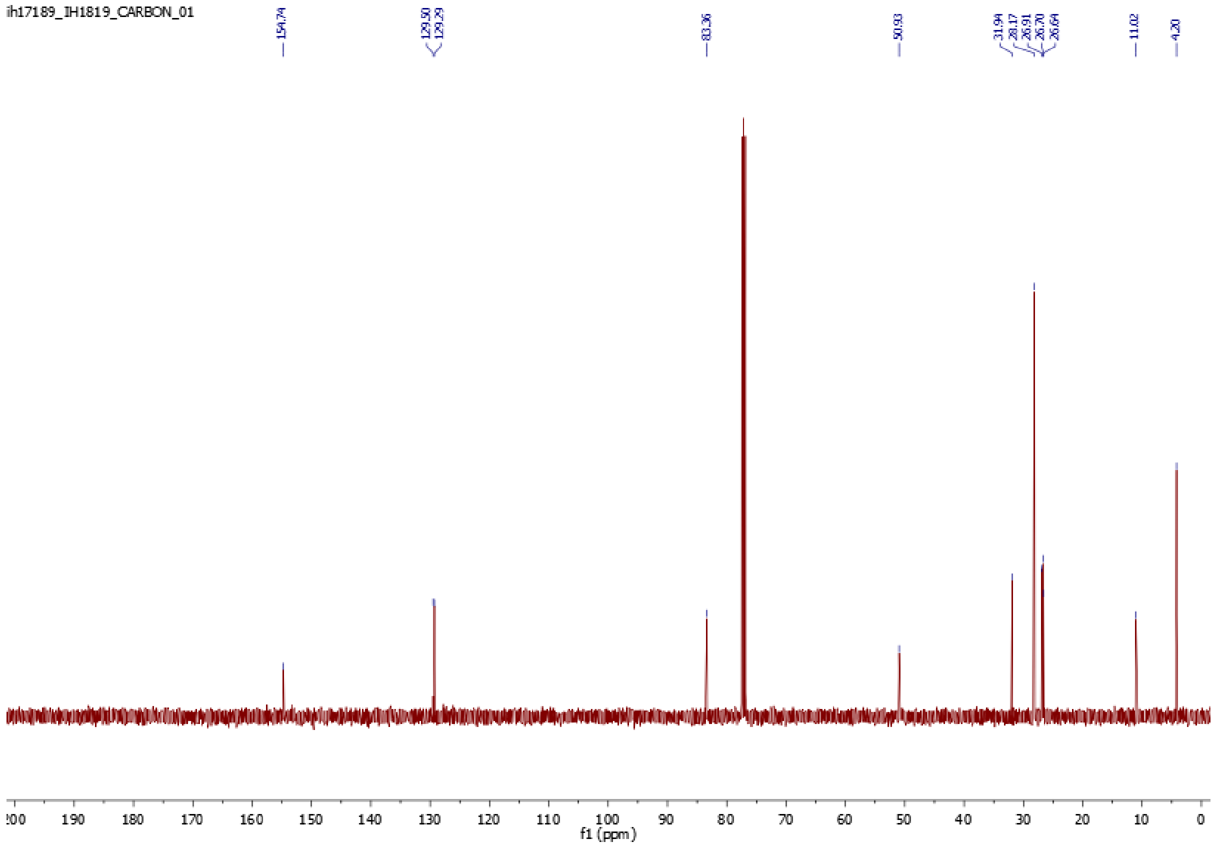
ih17189_IH1819_CARBON_01
154.74
129.50
129.29
83.36
50.93
31.94
28.17
26.91
26.70
26.64
11.02
4.20
190 180 170 160 150 140 130 120 110 100 90 80 70 60 50 40 30 20 10 0
f1 (ppm)

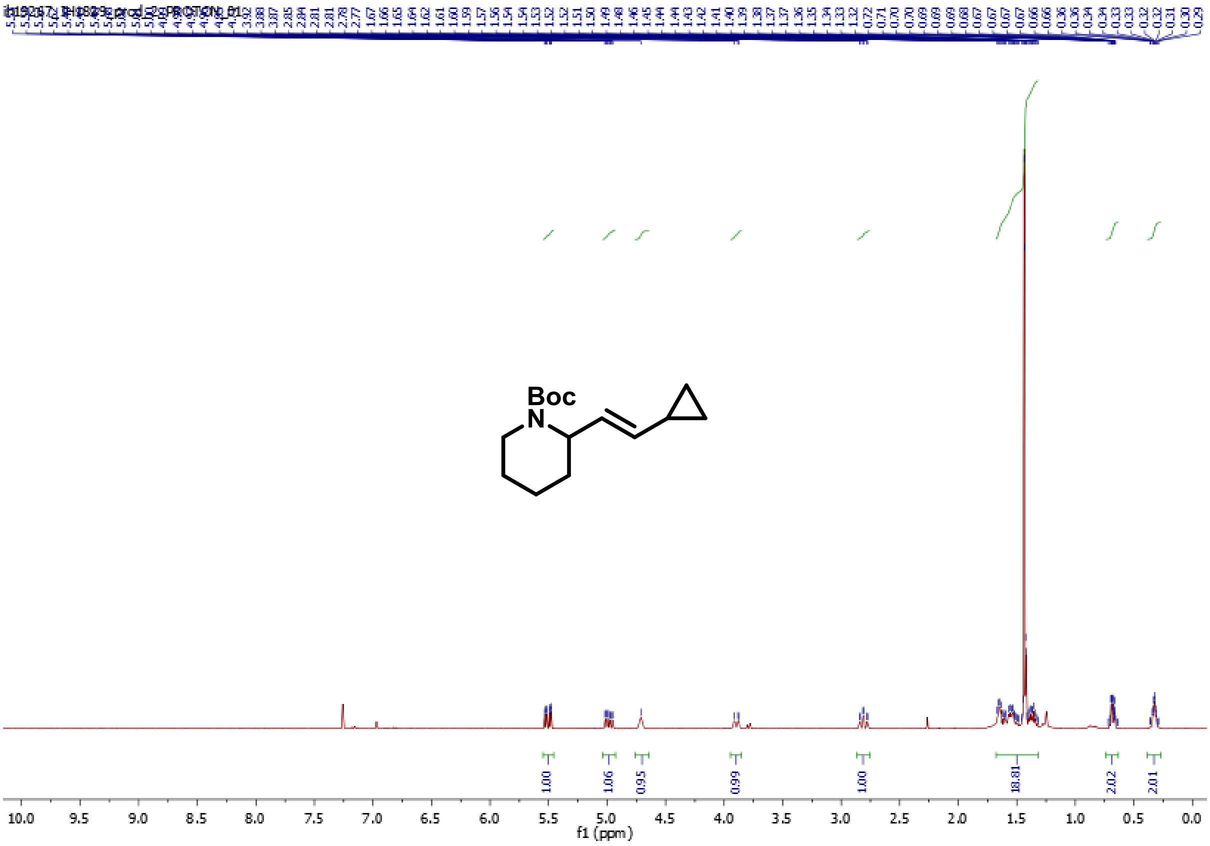

Boc
N
1.00
1.06
0.95
0.99
1.00
18.81
2.02
2.01
10.0 9.5 9.0 8.5 8.0 7.5 7.0 6.5 6.0 5.5 5.0 4.5 4.0 3.5 3.0 2.5 2.0 1.5 1.0 0.5 0.0
f1 (ppm)

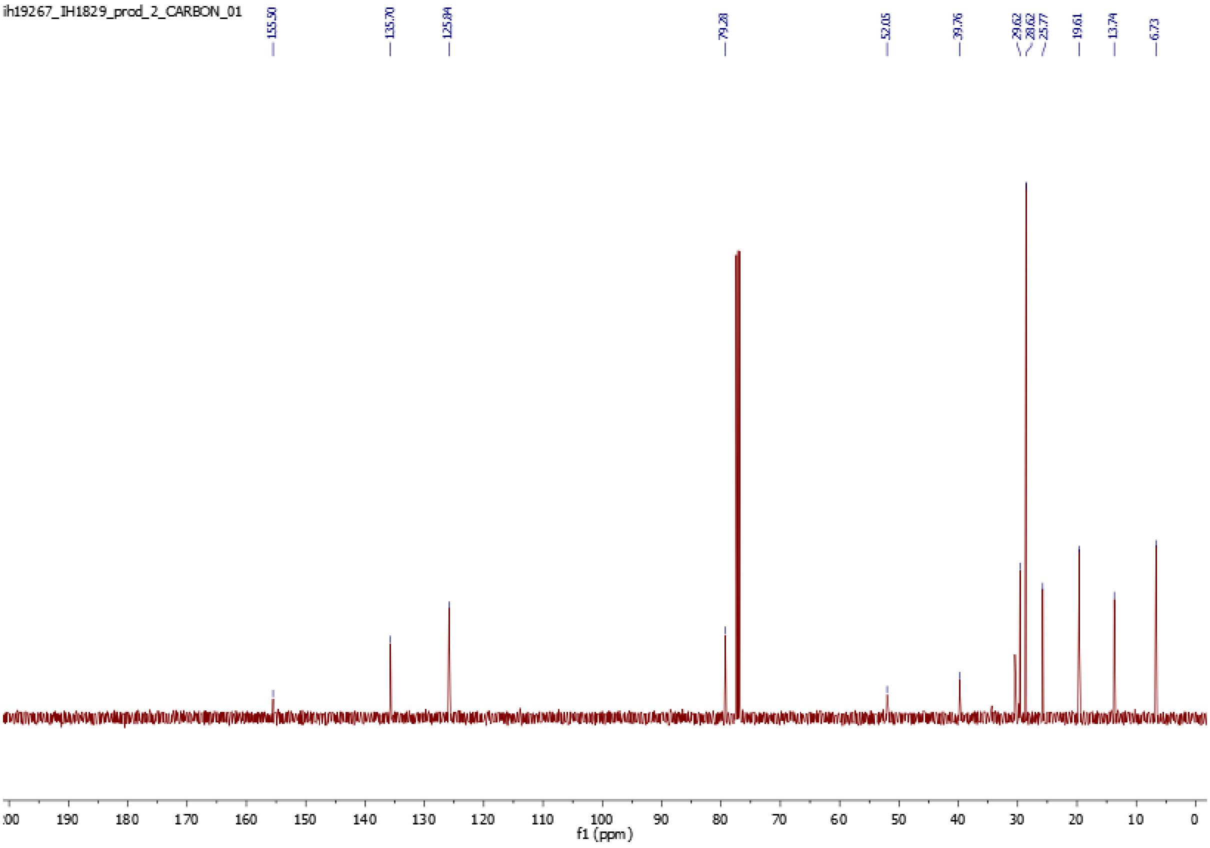

ih19267_IH1829_prod_2_CARBON_01
155.50
135.70
125.84
79.28
52.05
39.76
29.62
28.62
25.77
19.61
13.74
6.73
190 180 170 160 150 140 130 120 110 100 90 80 70 60 50 40 30 20 10 0
f1 (ppm)

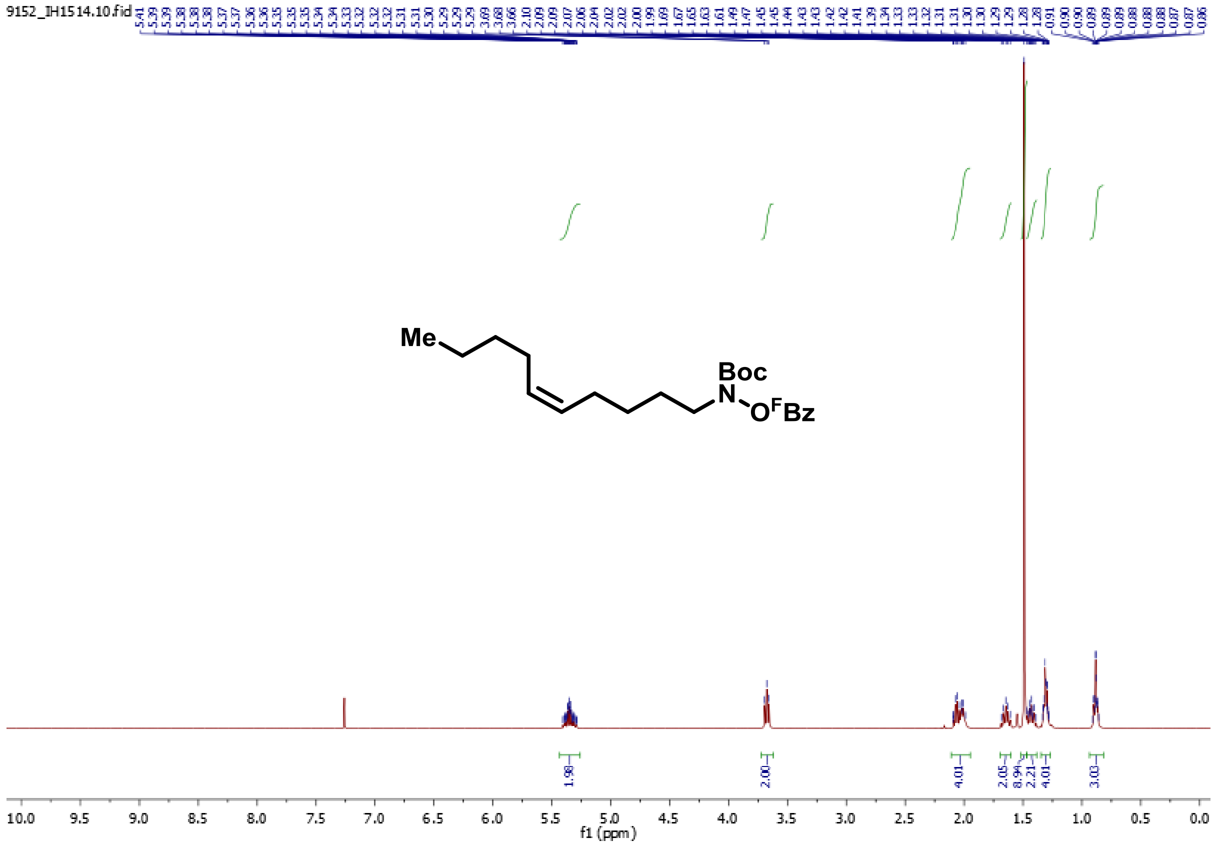
9152_IH1514.10.fid
Me
Boc
N
O^{F}Bz
f1 (ppm)

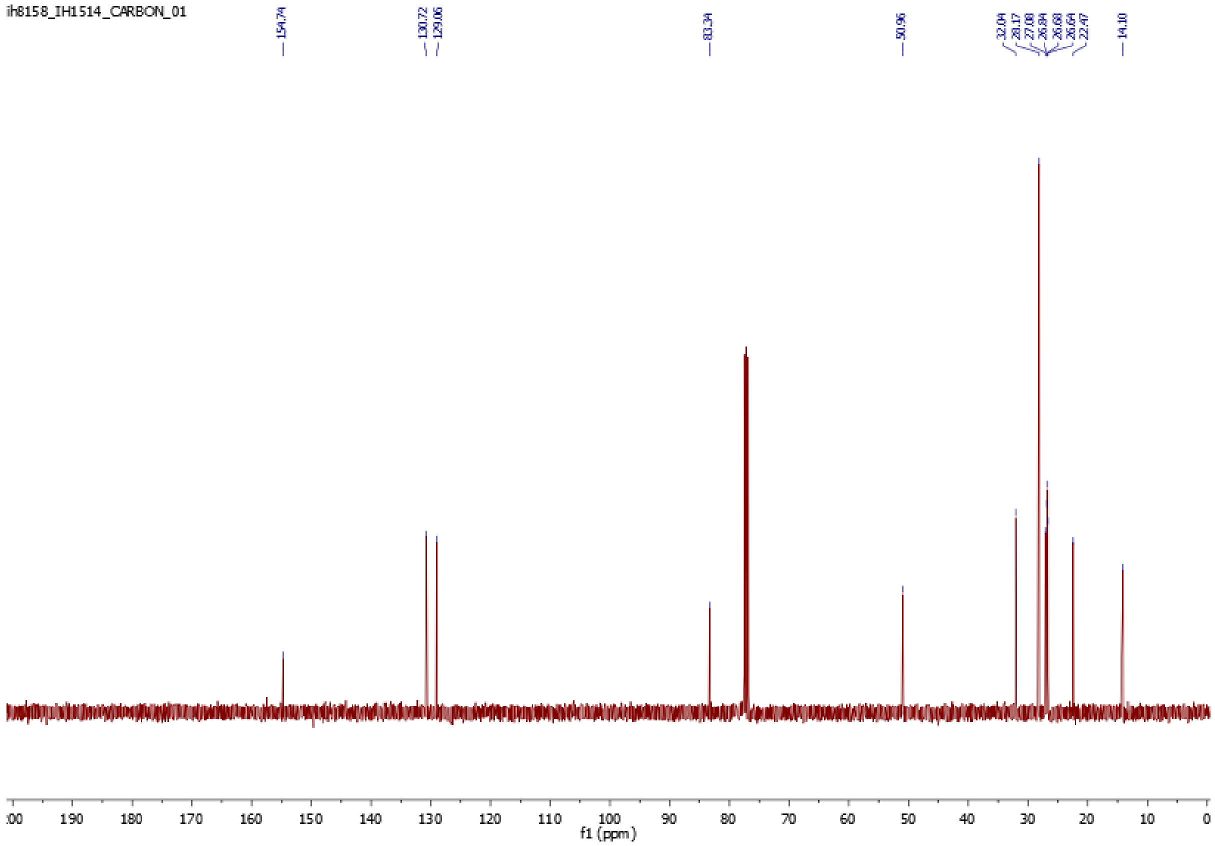
ih8158_IH1514_CARBON_01
154.74
130.72
129.06
83.34
50.96
32.04
28.17
27.08
26.84
26.68
26.64
22.47
14.10
f1 (ppm)

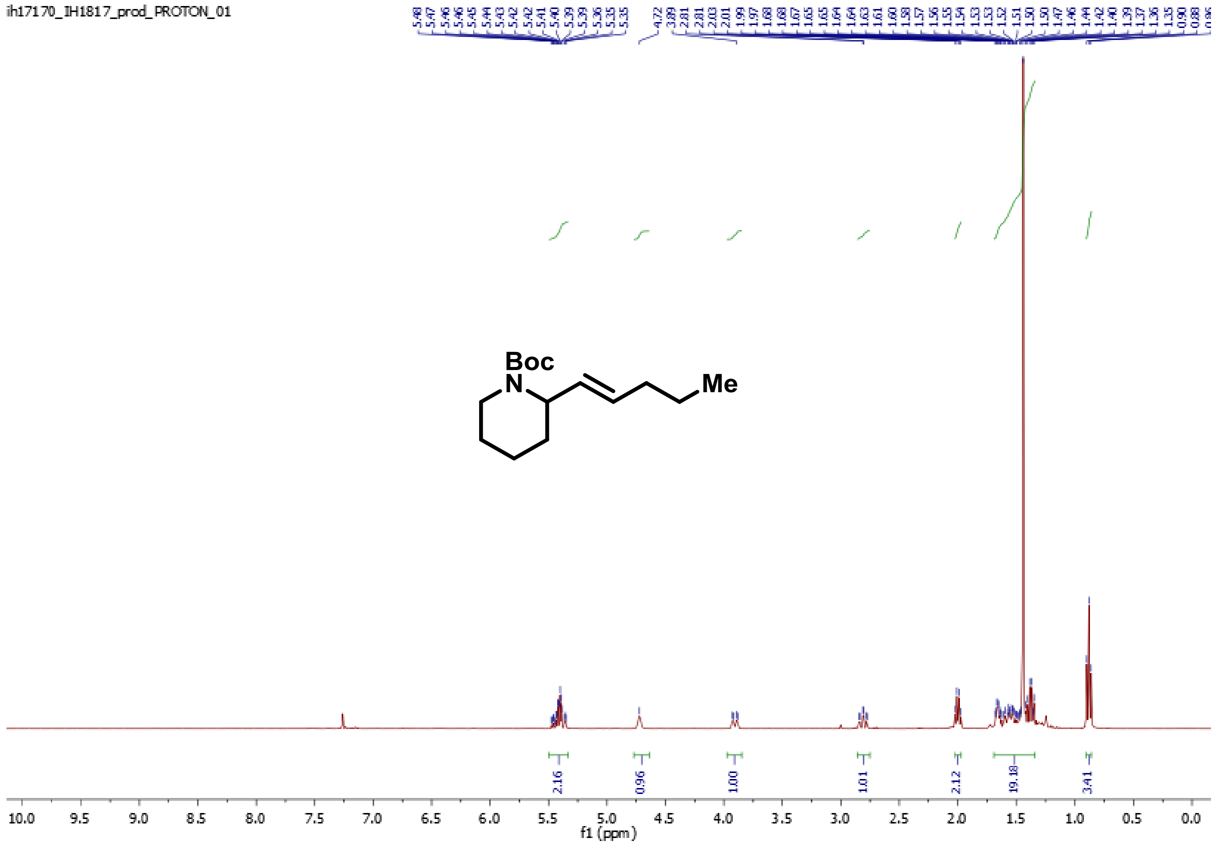
ih17170_IH1817_prod_PROTON_01
Boc
N
Me
2.16
0.96
1.00
1.01
2.12
19.18
3.41
f1 (ppm)

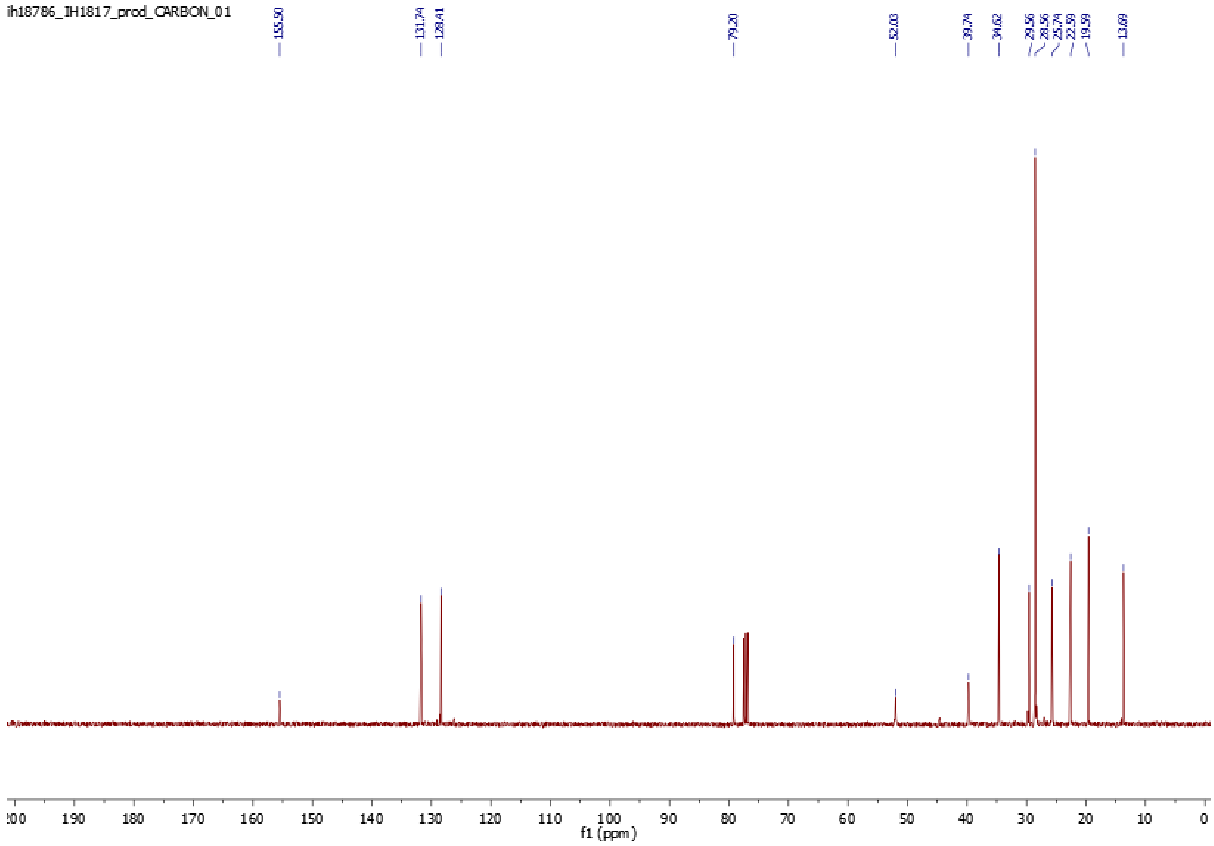
ih18786_IH1817_prod_CARBON_01
155.50
131.74
128.41
79.20
52.03
39.74
34.62
29.56
28.56
25.74
22.59
19.59
13.69
f1 (ppm)

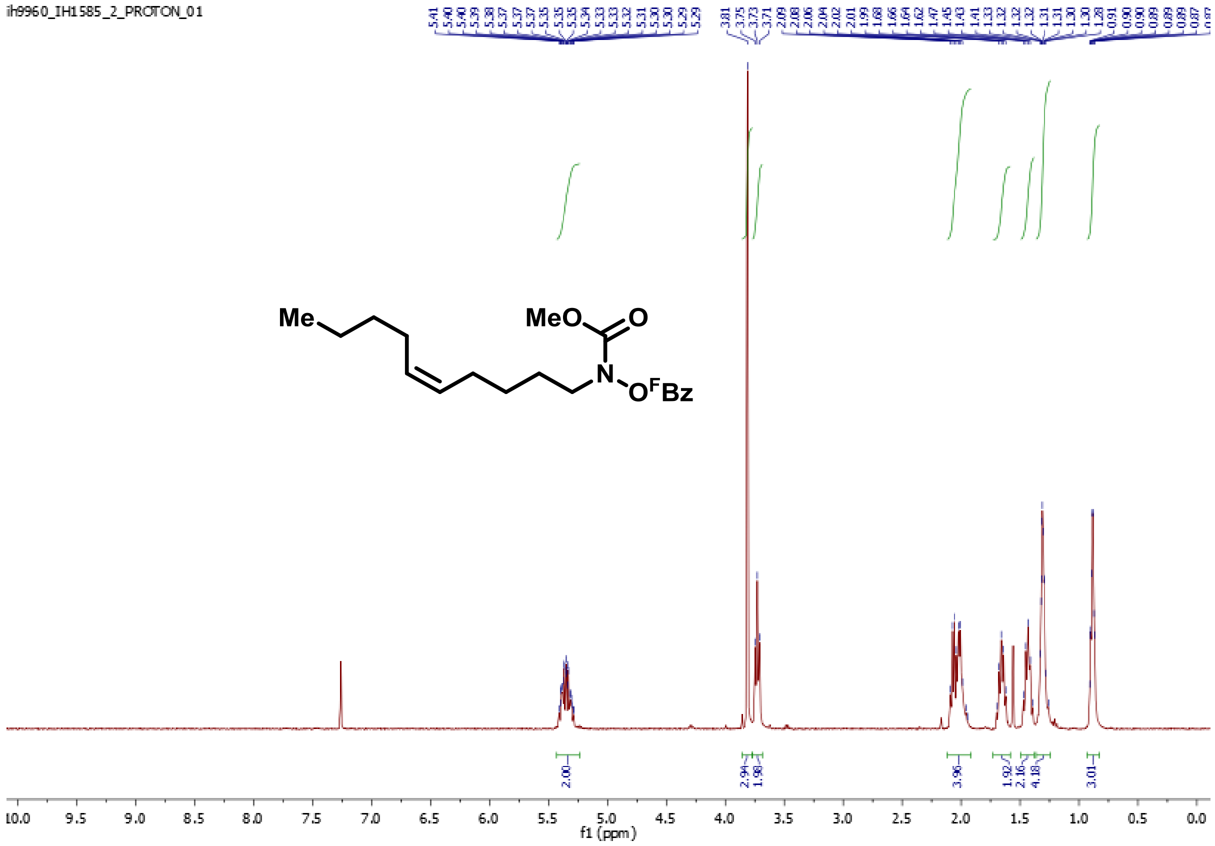
ih9960_IH1585_2_PROTON_01
Me
MeO
O
N
O^FBz
2.00
2.94
1.98
3.96
1.92
2.16
4.18
3.01
f1 (ppm)

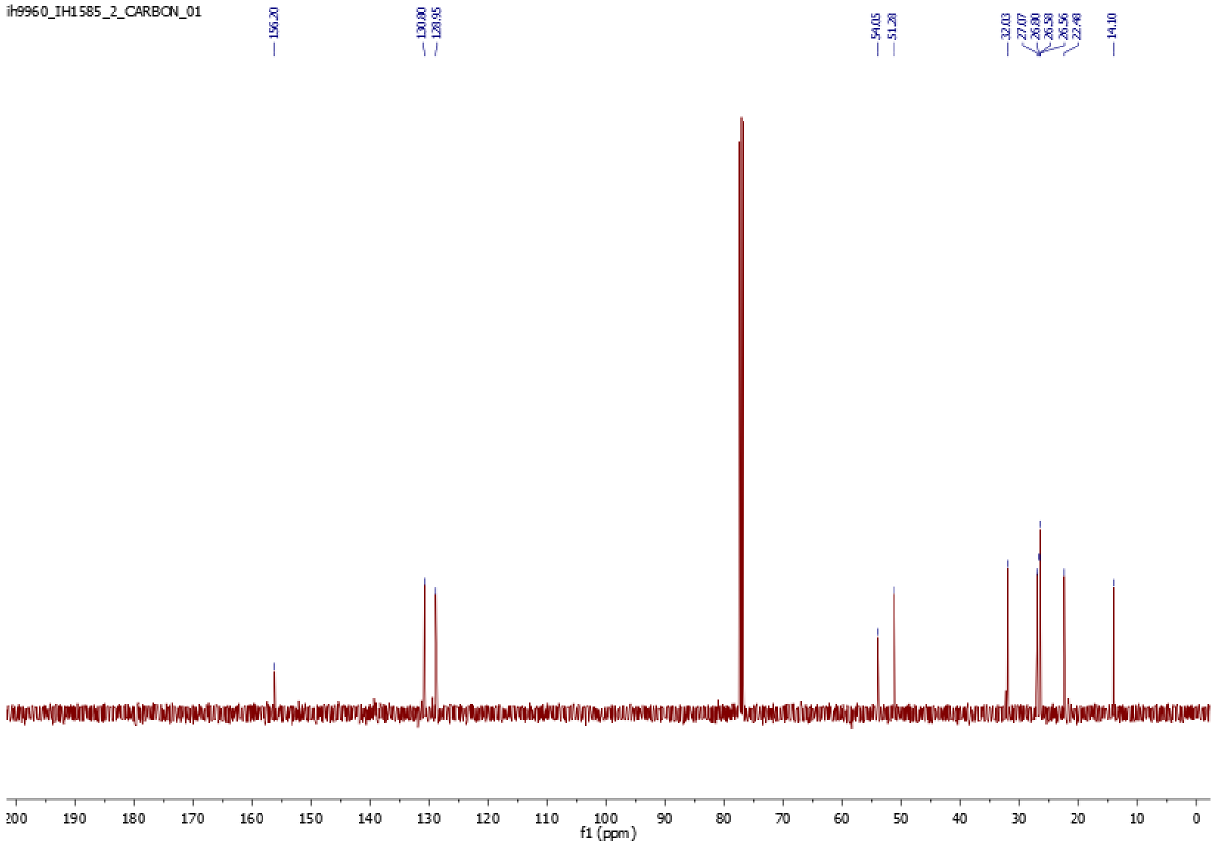
ih9960_IH1585_2_CARBON_01
156.20
130.80
128.95
54.05
51.28
32.03
27.07
26.80
26.58
26.56
22.48
14.10
f1 (ppm)

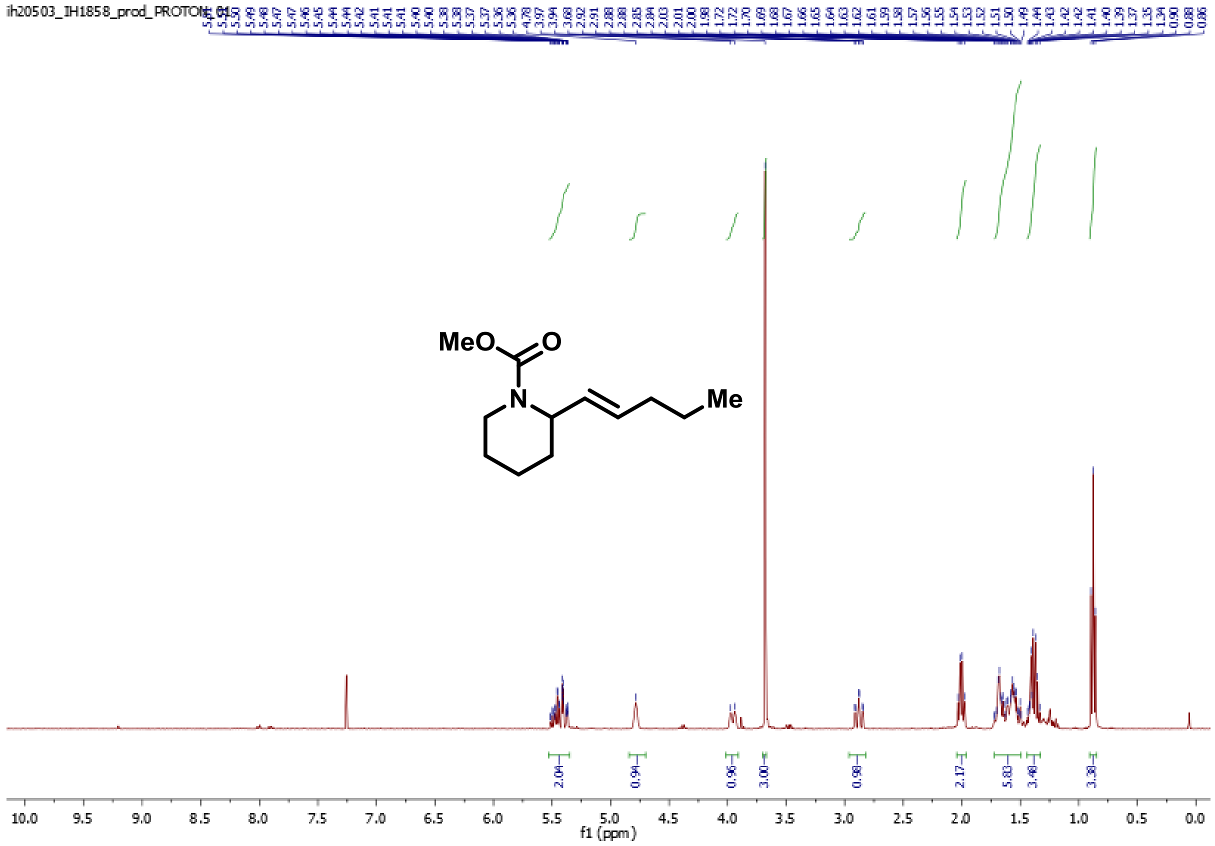
ih20503_IH1858_prod_PROTON_01
MeO
O
N
Me
f1 (ppm)

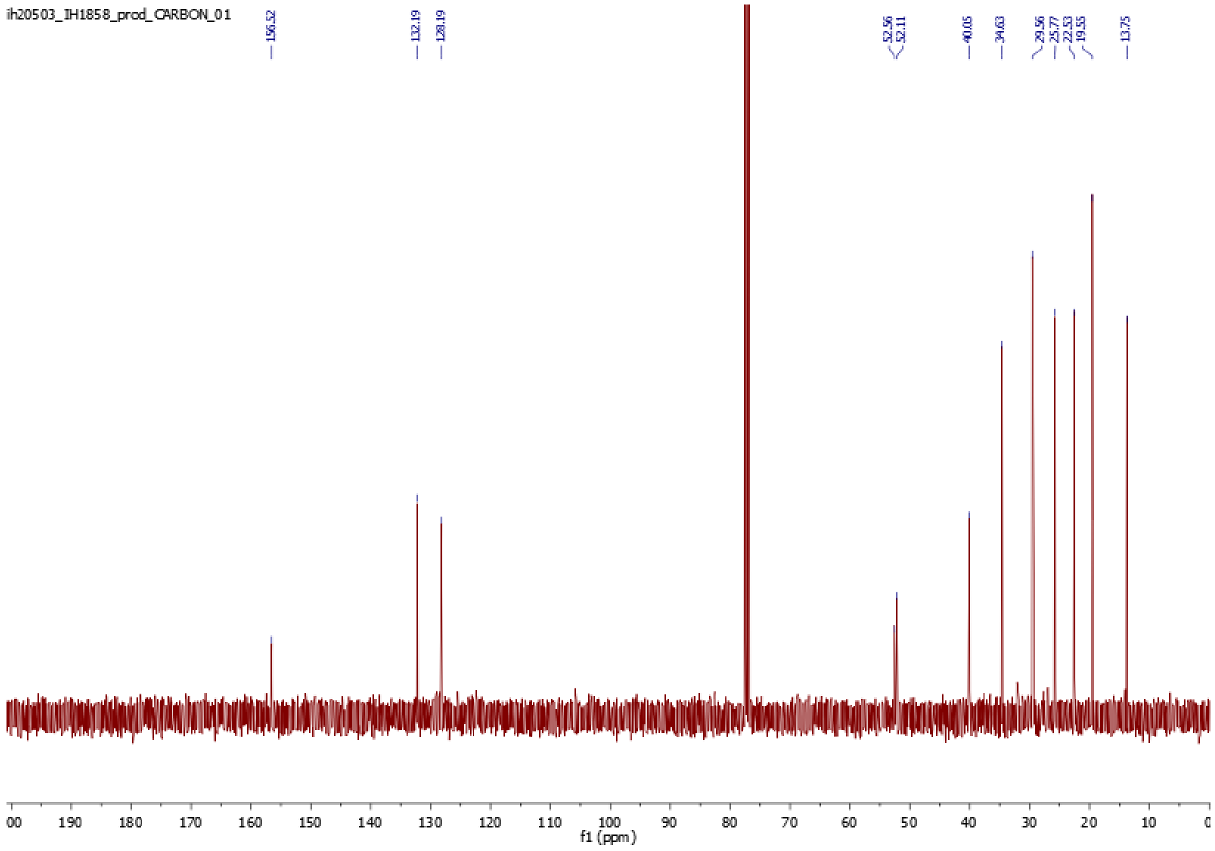
ih20503_IH1858_prod_CARBON_01
156.52
132.19
128.19
52.56
52.11
40.05
34.63
29.56
25.77
22.53
19.55
13.75
f1 (ppm)

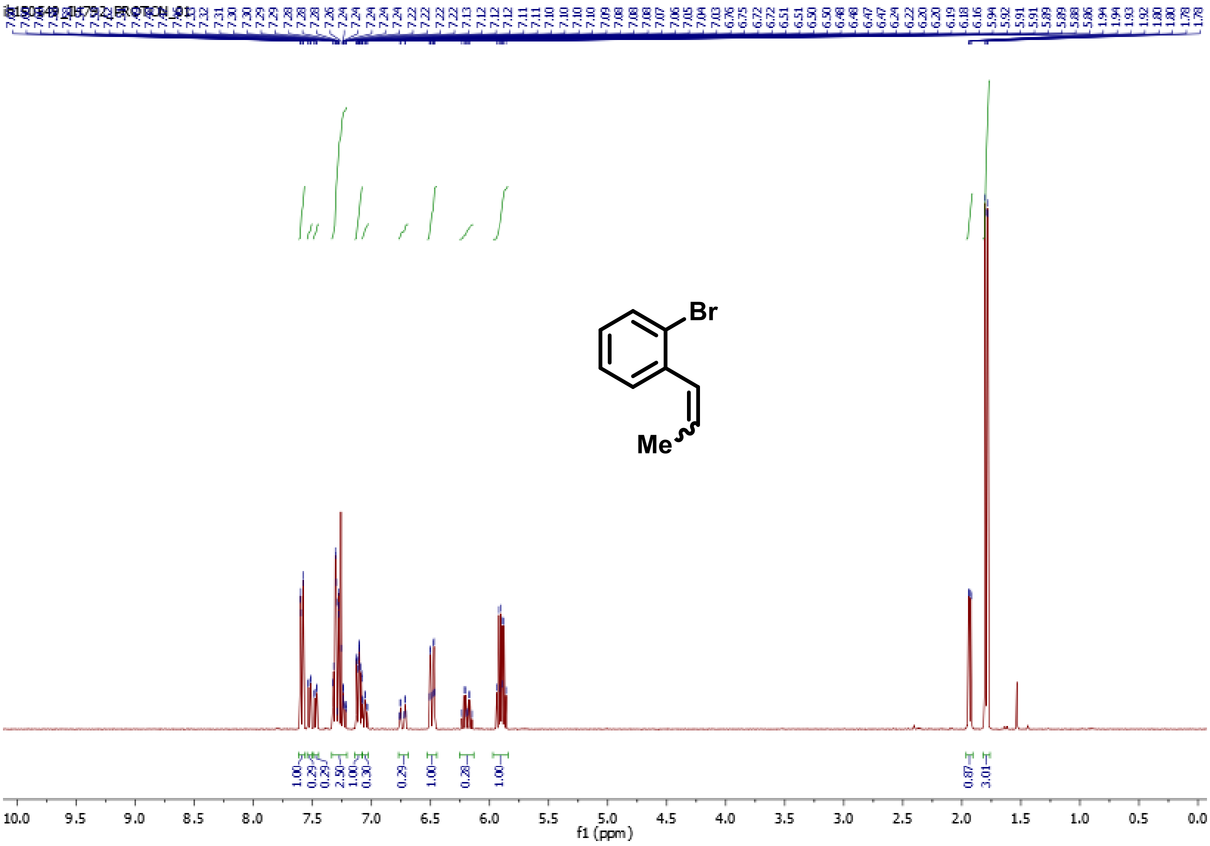
Br
Me
f1 (ppm)

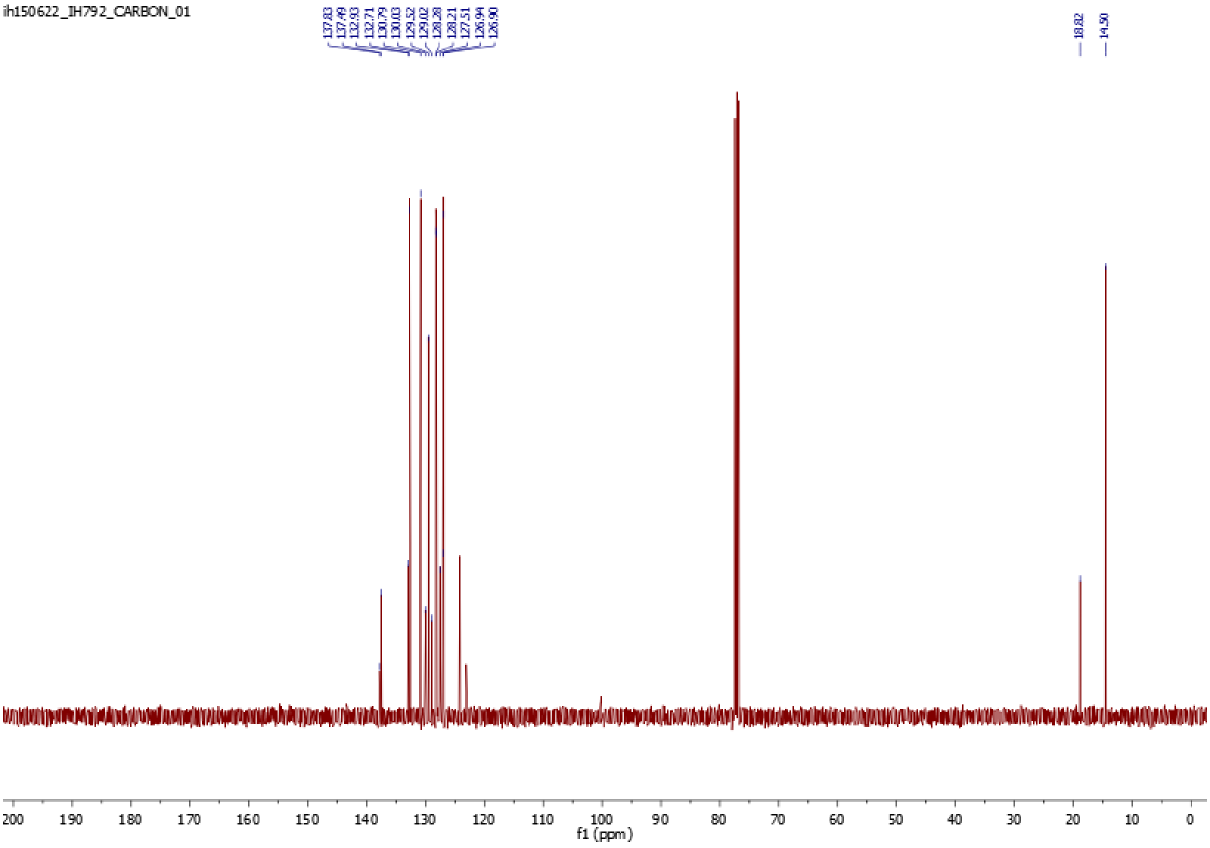
ih150622_IH792_CARBON_01
137.83
137.49
132.93
132.71
130.79
130.03
129.52
129.02
128.28
128.21
127.51
126.94
126.90
18.82
14.50
f1 (ppm)

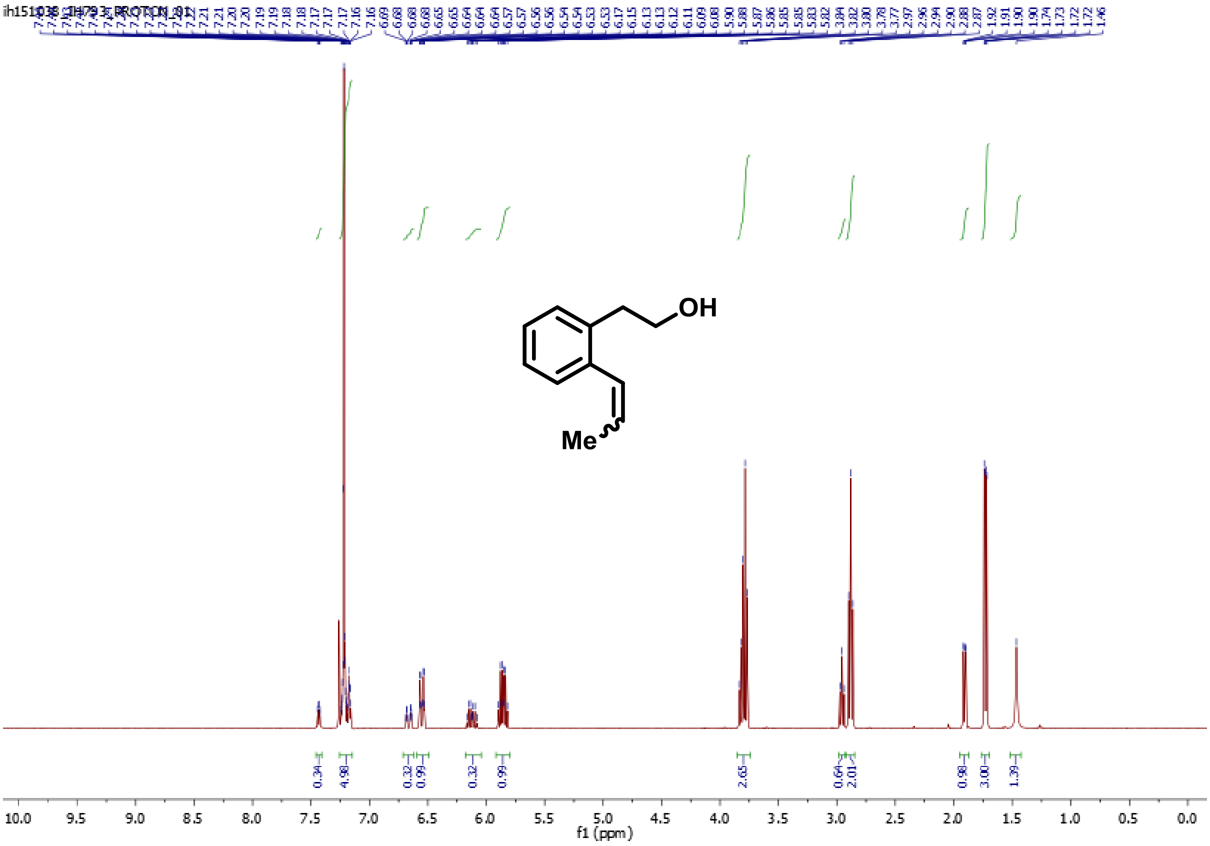
ih151038_IH793_PROTON_01
OH
Me
f1 (ppm)

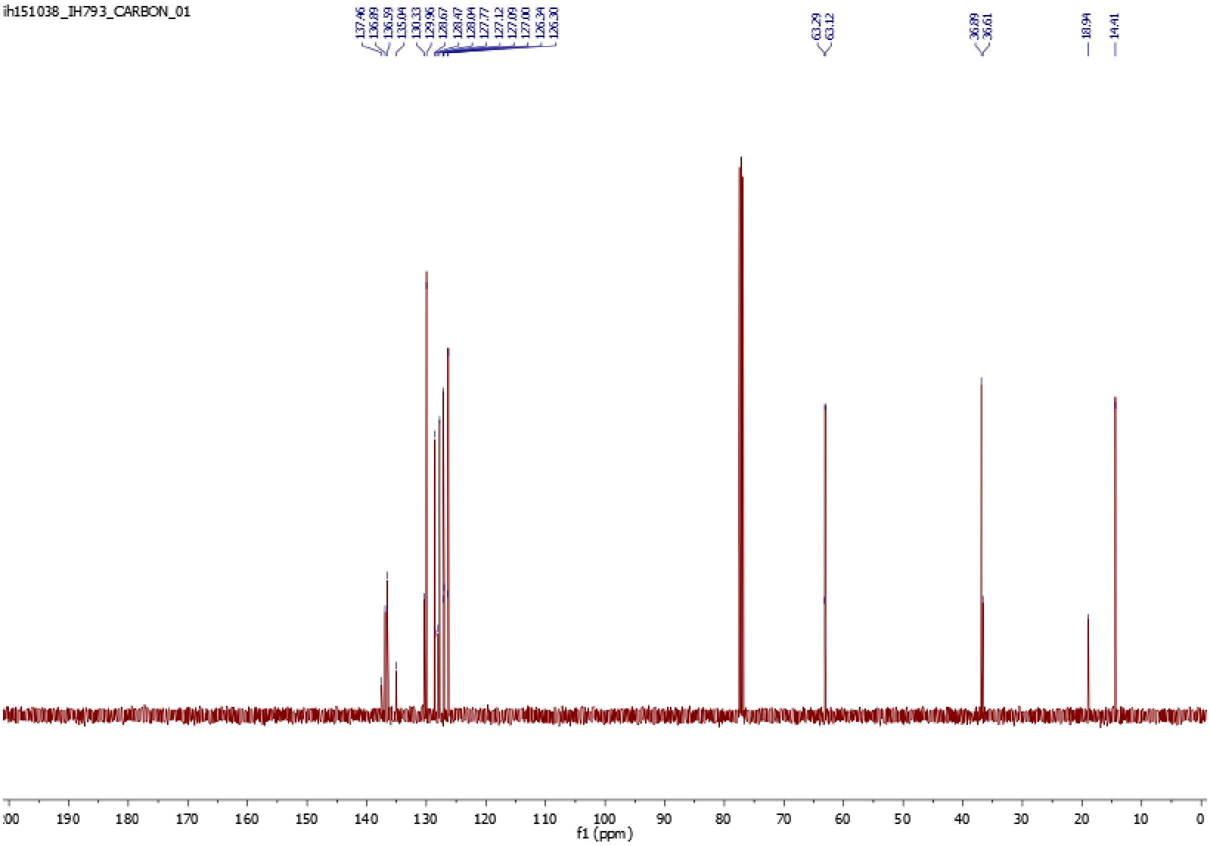
ih151038_IH793_CARBON_01
f1 (ppm)

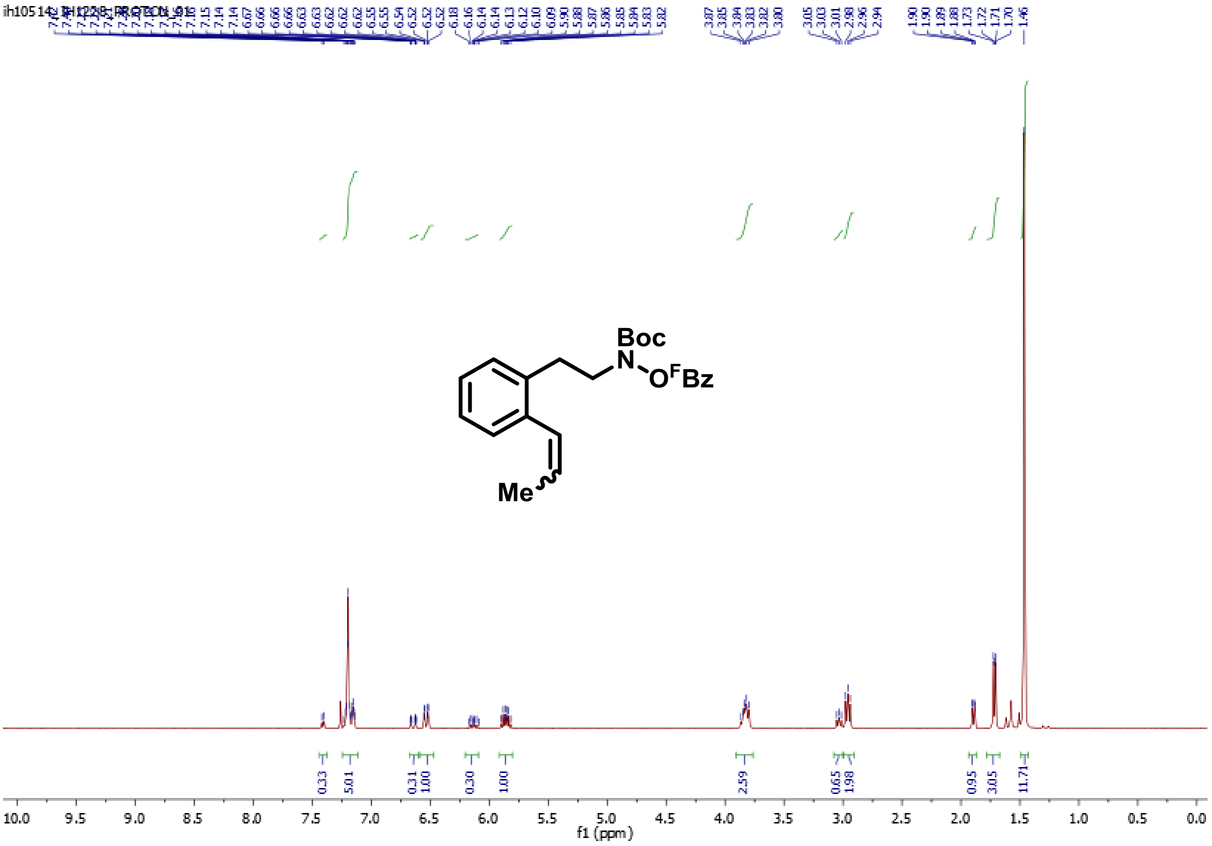

ih10514_IH1228_PROTON_01
Boc
N
O^FBz
Me
0.33
5.01
0.31
1.00
0.30
1.00
2.59
0.65
1.98
0.95
3.05
11.71
f1 (ppm)

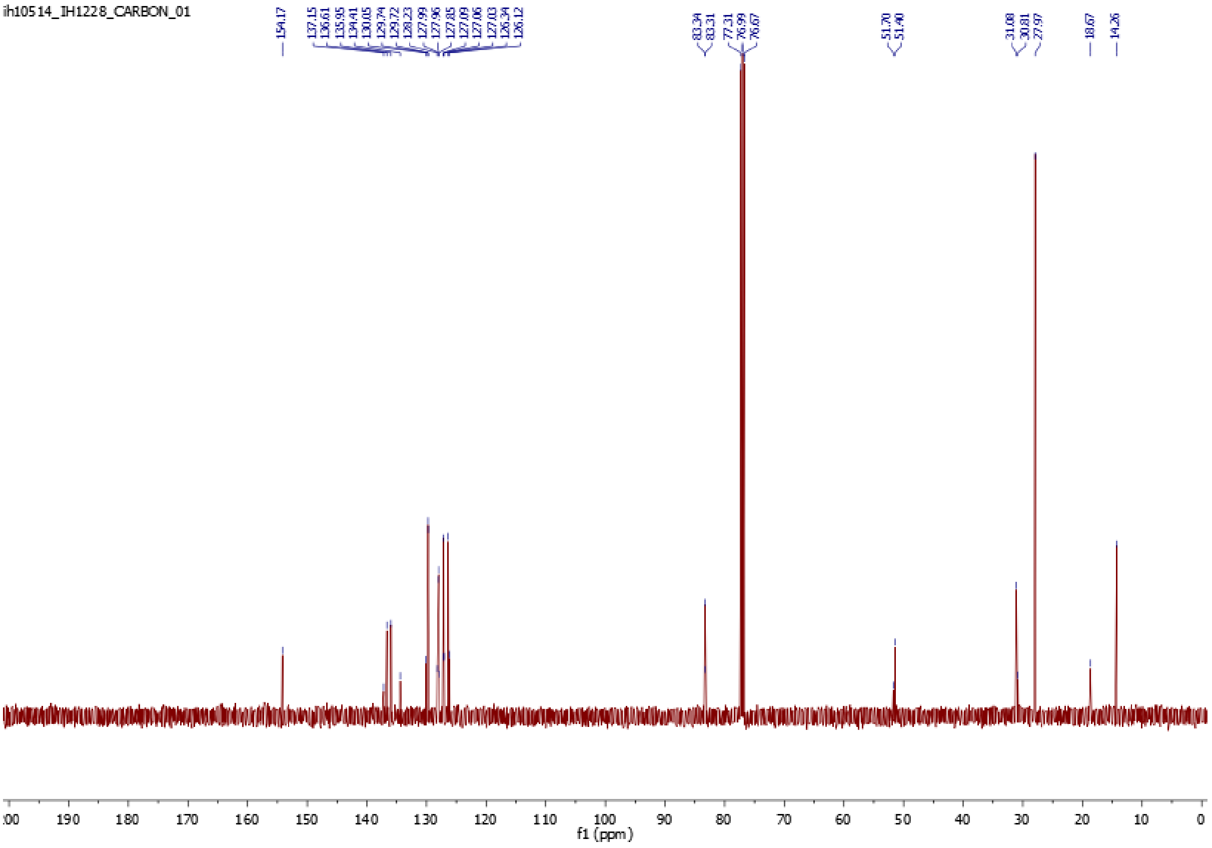

ih10514_IH1228_CARBON_01
154.17
137.15
136.61
135.95
134.41
130.05
129.74
129.72
128.23
127.99
127.96
127.85
127.09
127.06
127.03
126.34
126.12
83.34
83.31
77.31
76.99
76.67
51.70
51.40
31.08
30.81
27.97
18.67
14.26
f1 (ppm)

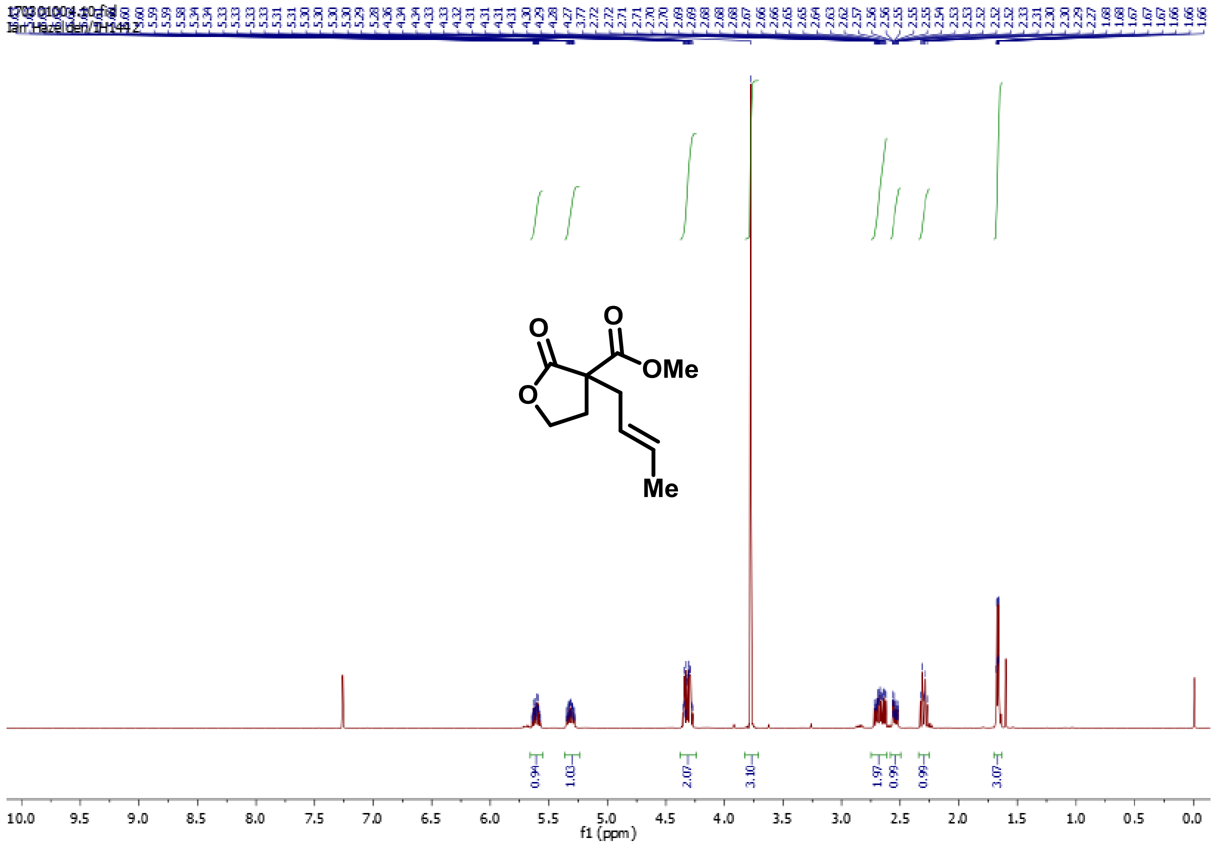
O
O
O
OMe
Me
0.94
1.03
2.07
3.10
1.97
0.99
0.99
3.07
10.0 9.5 9.0 8.5 8.0 7.5 7.0 6.5 6.0 5.5 5.0 4.5 4.0 3.5 3.0 2.5 2.0 1.5 1.0 0.5 0.0
f1 (ppm)

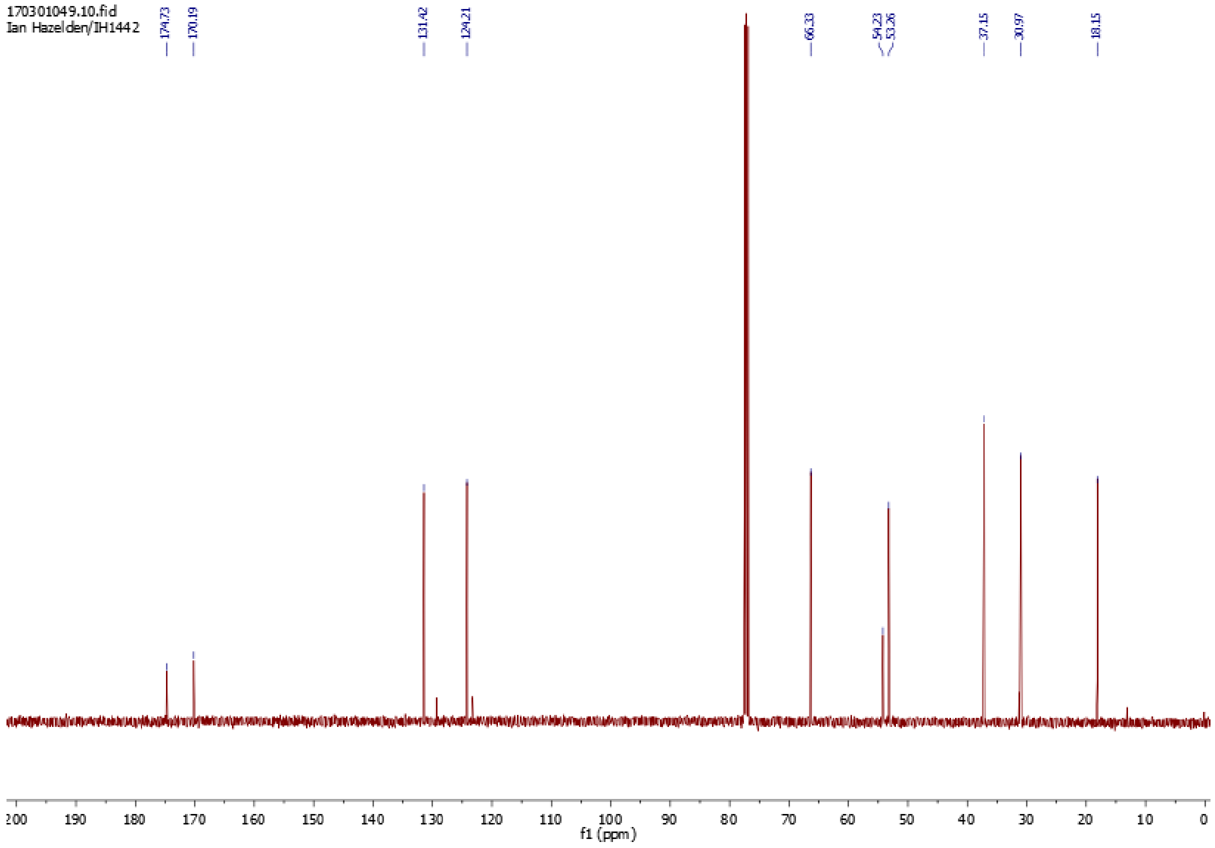
170301049.10.fid
Ian Hazelden/IH1442
174.73
170.19
131.42
124.21
66.33
54.23
53.26
37.15
30.97
18.15
200 190 180 170 160 150 140 130 120 110 100 90 80 70 60 50 40 30 20 10 0
f1 (ppm)

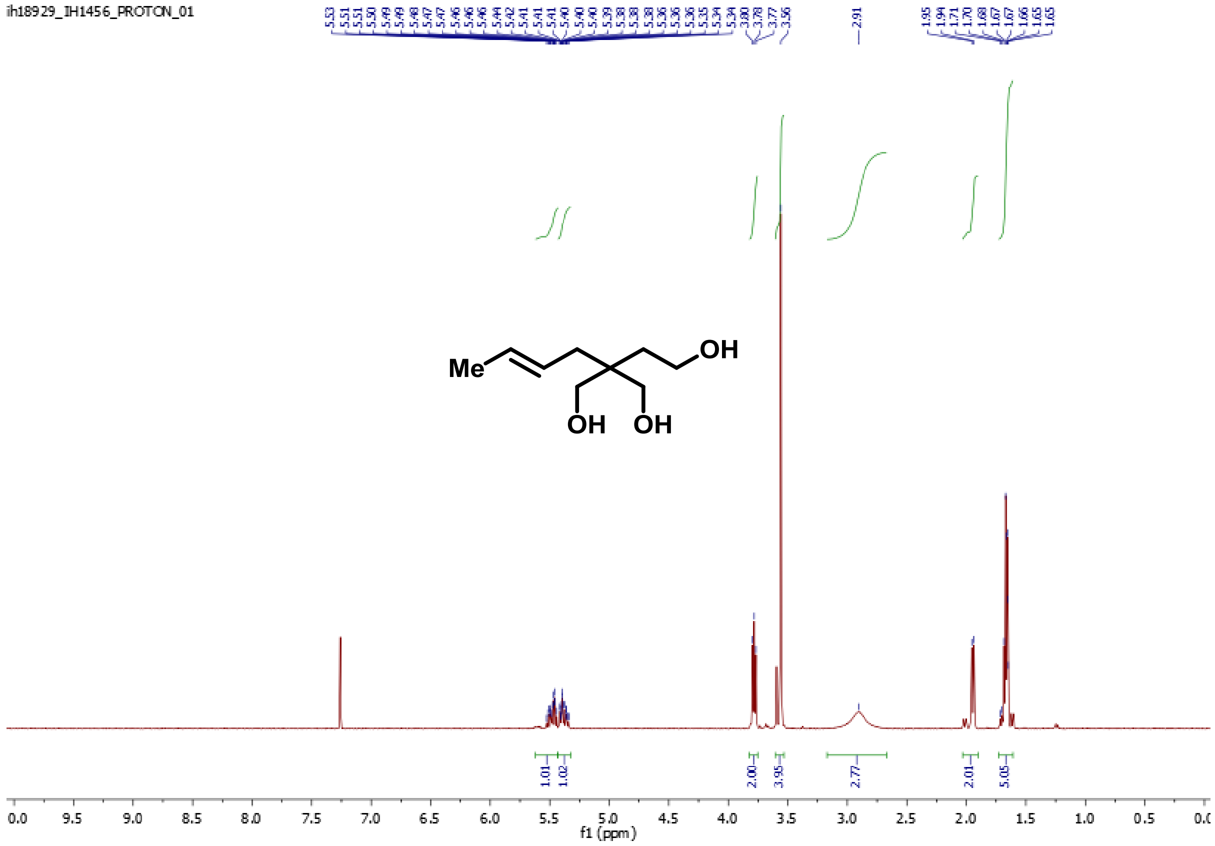
ih18929_IH1456_PROTON_01
f1 (ppm)
Me
OH
OH
OH

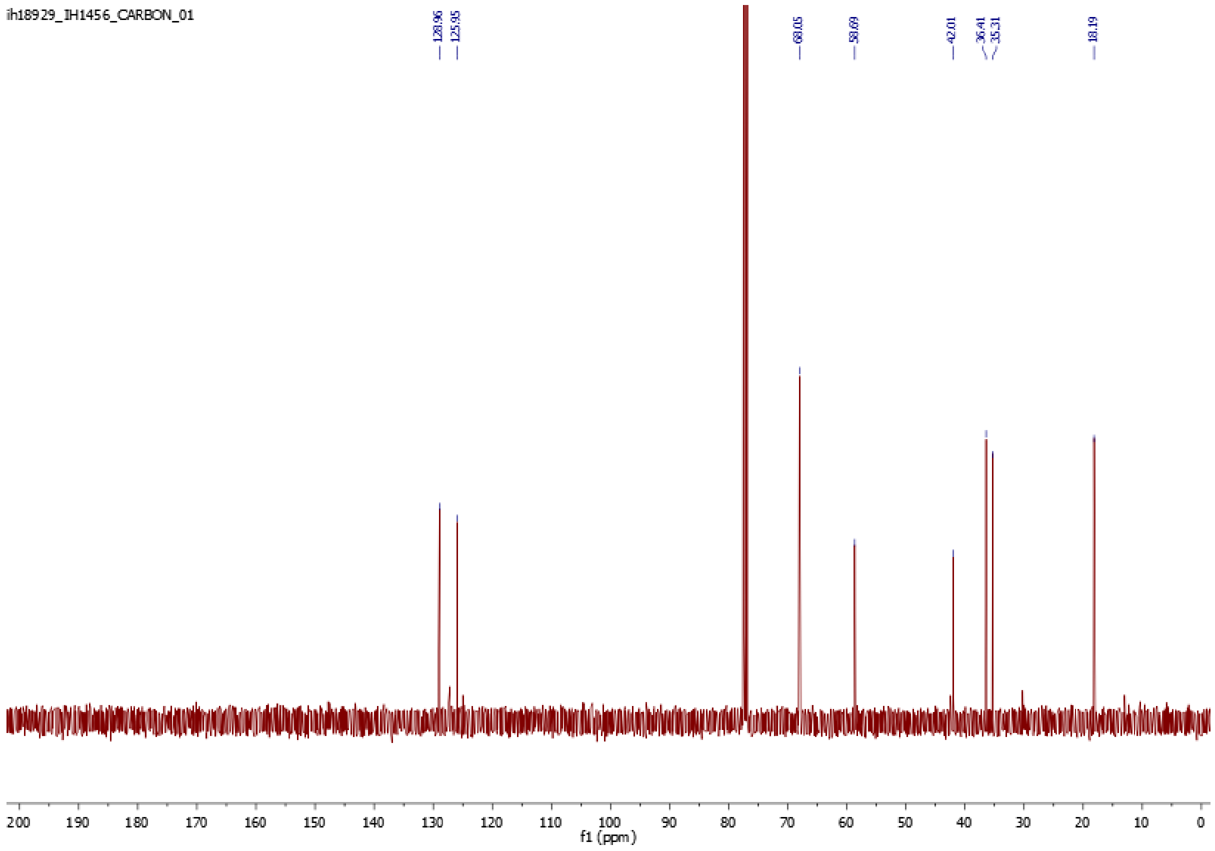
ih18929_IH1456_CARBON_01
f1 (ppm)

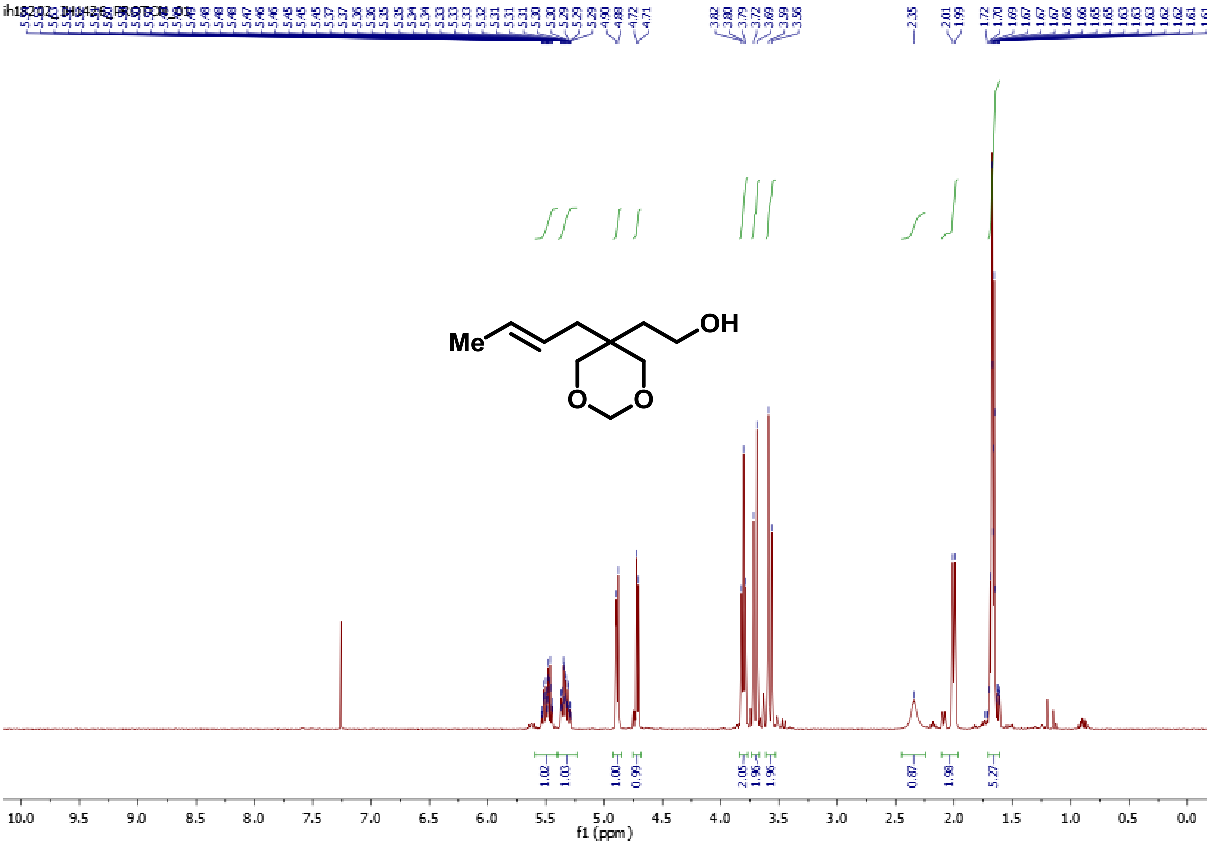
Me
OH
O
O
1.02
1.03
1.00
0.99
2.05
1.96
1.96
0.87
1.98
5.27
f1 (ppm)

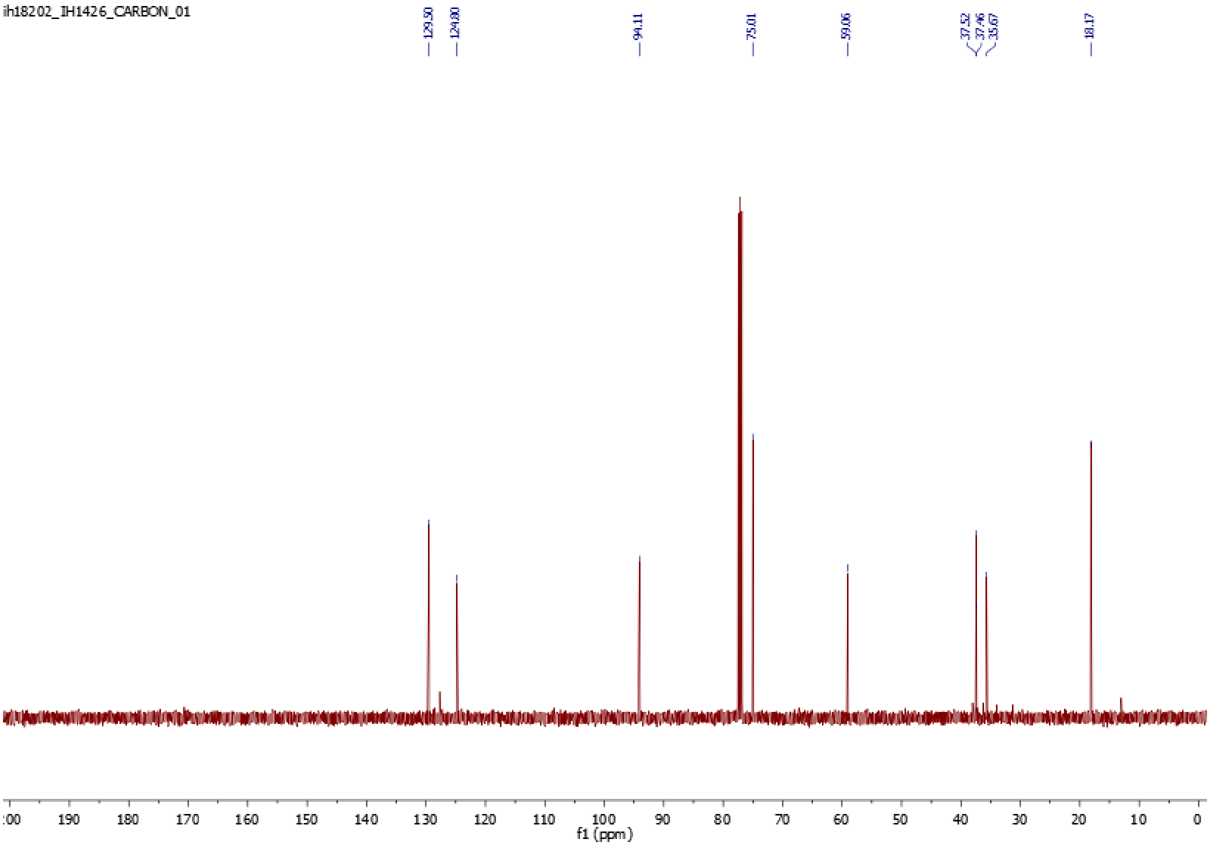
ih18202_IH1426_CARBON_01
129.50
124.80
94.11
75.01
59.06
37.52
37.46
35.67
18.17
f1 (ppm)

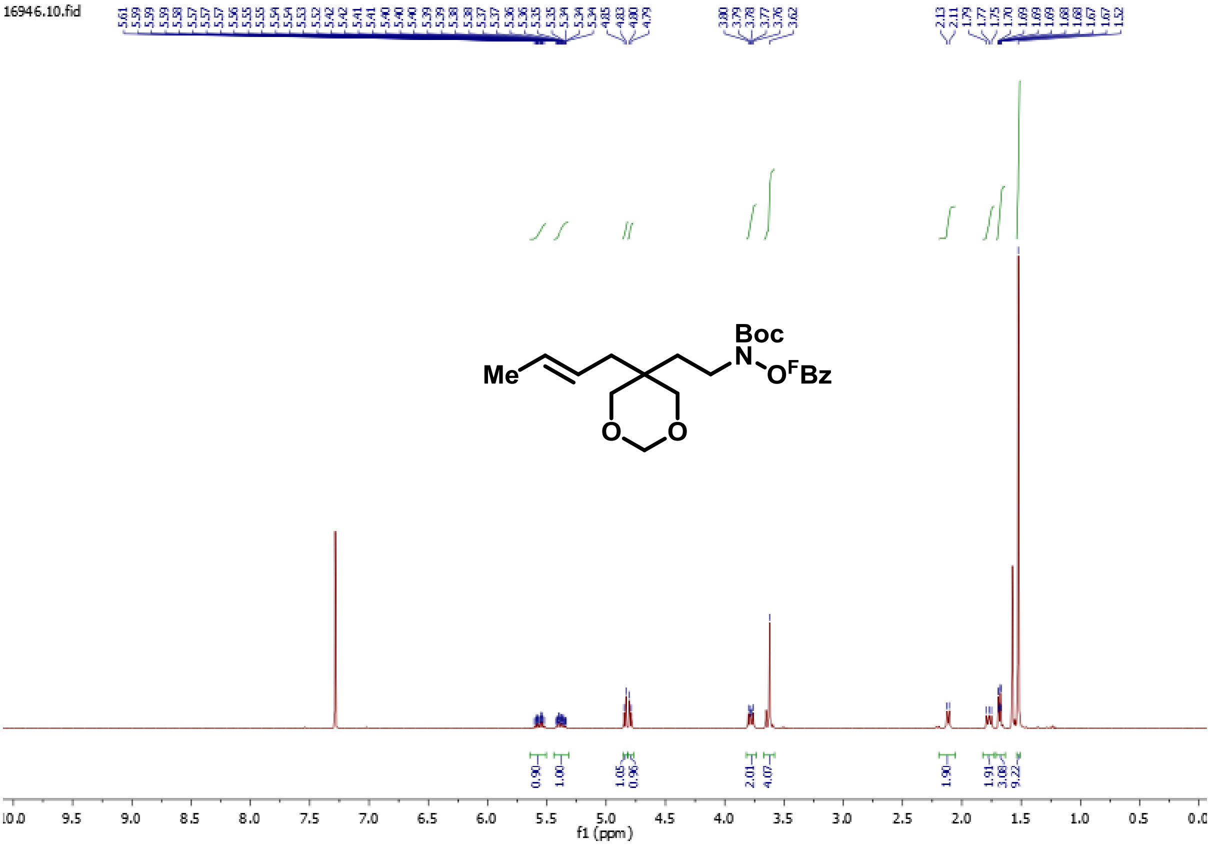
16946.10.fid
5.61 5.59 5.59 5.58 5.57 5.57 5.56 5.55 5.55 5.54 5.54 5.53 5.52 5.42 5.42 5.41 5.41 5.40 5.40 5.40 5.39 5.39 5.38 5.38 5.37 5.37 5.36 5.35 5.35 5.34 5.34 5.34 4.85 4.83 4.80 4.79
3.80 3.79 3.78 3.77 3.76 3.62
2.13 2.11 1.79 1.77 1.75 1.70 1.69 1.69 1.69 1.68 1.68 1.67 1.52
Boc
N
O
F
Bz
Me
O
O
0.90 1.00 1.05 0.90 2.01 4.07 1.90 1.91 3.08 9.22
10.0 9.5 9.0 8.5 8.0 7.5 7.0 6.5 6.0 5.5 5.0 4.5 4.0 3.5 3.0 2.5 2.0 1.5 1.0 0.5 0.0
f1 (ppm)

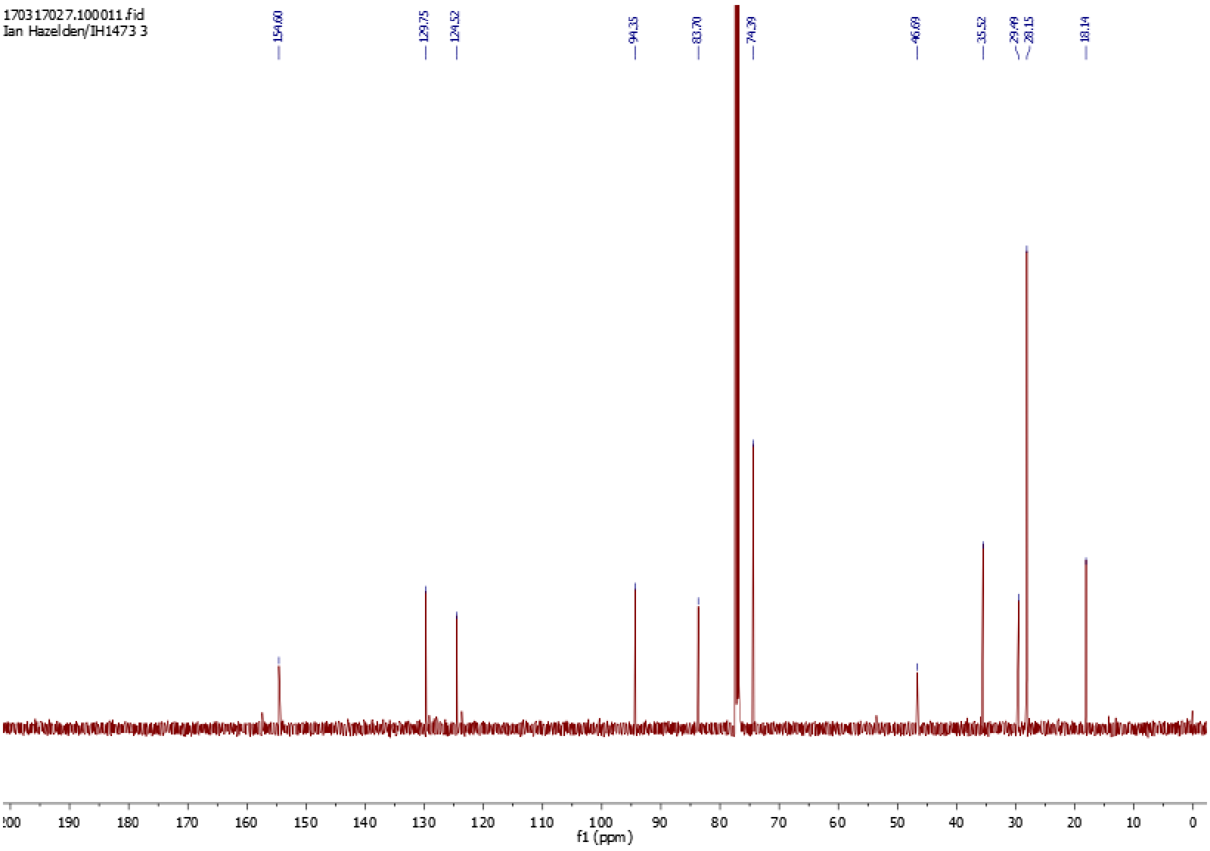
170317027.100011.fid
Ian Hazelden/IH1473 3
154.60 129.75 124.52 94.35 83.70 74.39 46.69 35.52 29.49 28.15 18.14
200 190 180 170 160 150 140 130 120 110 100 90 80 70 60 50 40 30 20 10 0
f1 (ppm)

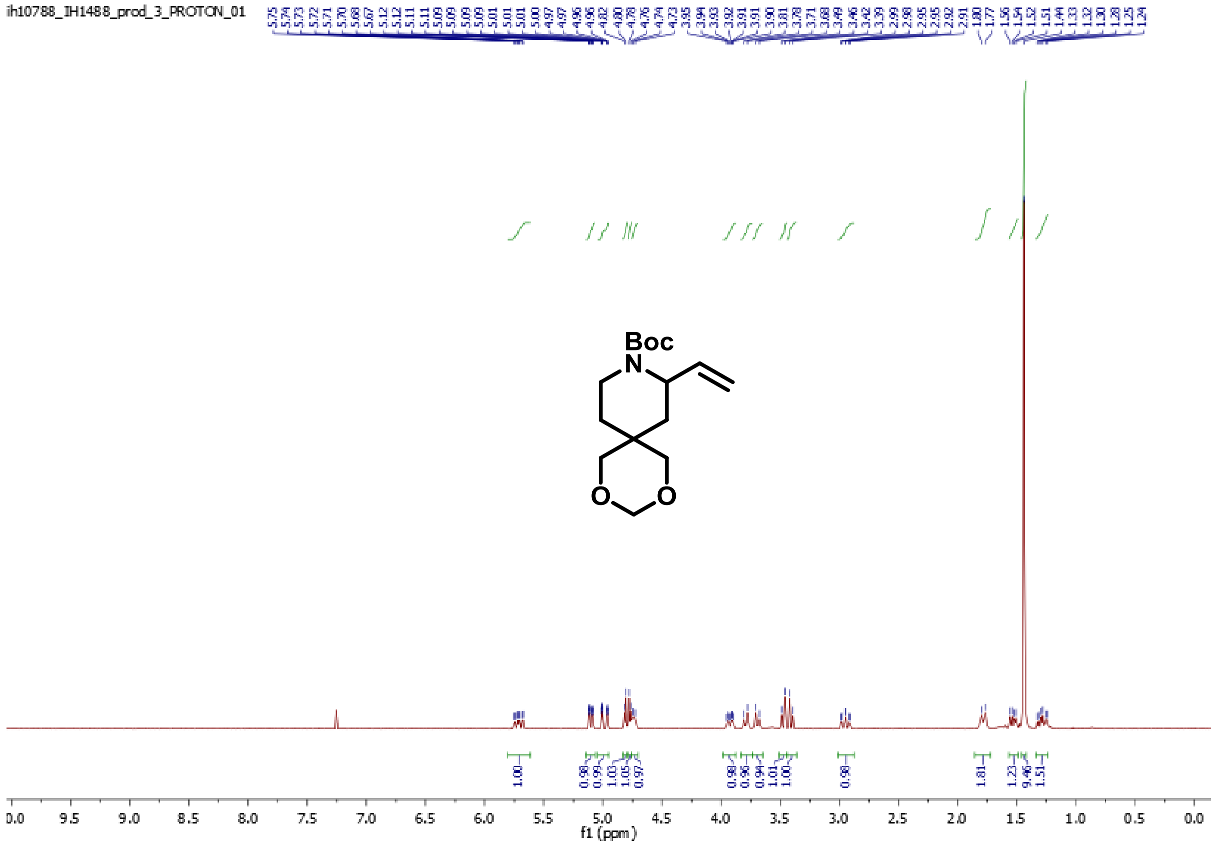
ih10788_IH1488_prod_3_PROTON_01
5.75 5.74 5.73 5.72 5.71 5.70 5.68 5.67 5.12 5.12 5.11 5.11 5.09 5.09 5.09 5.09 5.09 5.01 5.01 5.01 5.00 4.97 4.97 4.96 4.96 4.82 4.80 4.78 4.76 4.74 4.73 3.95 3.94 3.93 3.92 3.91 3.91 3.90 3.81 3.78 3.71 3.68 3.49 3.46 3.42 3.39 2.99 2.98 2.95 2.95 2.92 2.91 1.80 1.77 1.56 1.54 1.52 1.51 1.44 1.33 1.32 1.30 1.28 1.25 1.24
Boc
N
O
O
1.00
0.98
0.99
1.03
1.05
0.97
0.98
0.96
0.94
1.01
1.00
0.98
1.81
1.23
9.46
1.51
f1 (ppm)

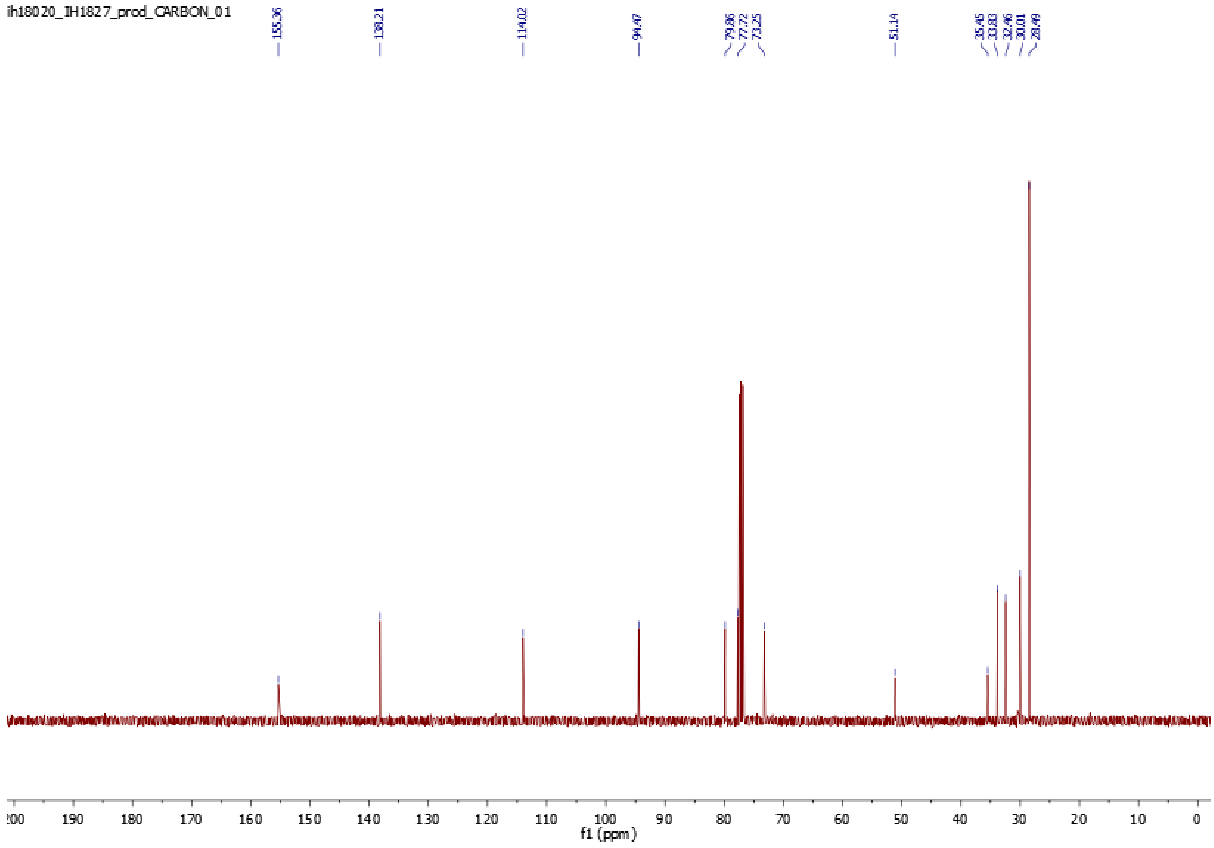
ih18020_IH1827_prod_CARBON_01
155.36
138.21
114.02
94.47
79.86
77.22
73.25
51.14
35.45
33.83
32.46
30.01
28.49
f1 (ppm)

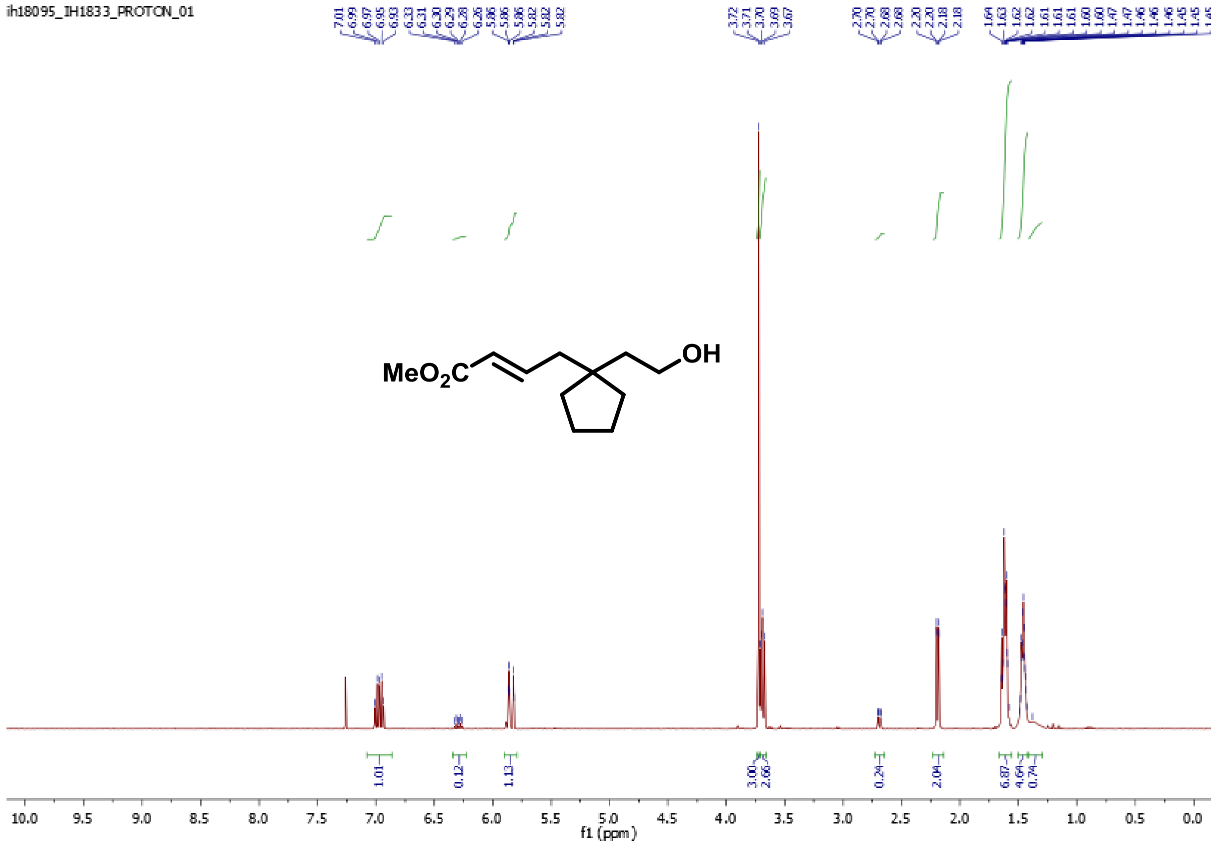
ih18095_IH1833_PROTON_01
MeO2C
OH
f1 (ppm)

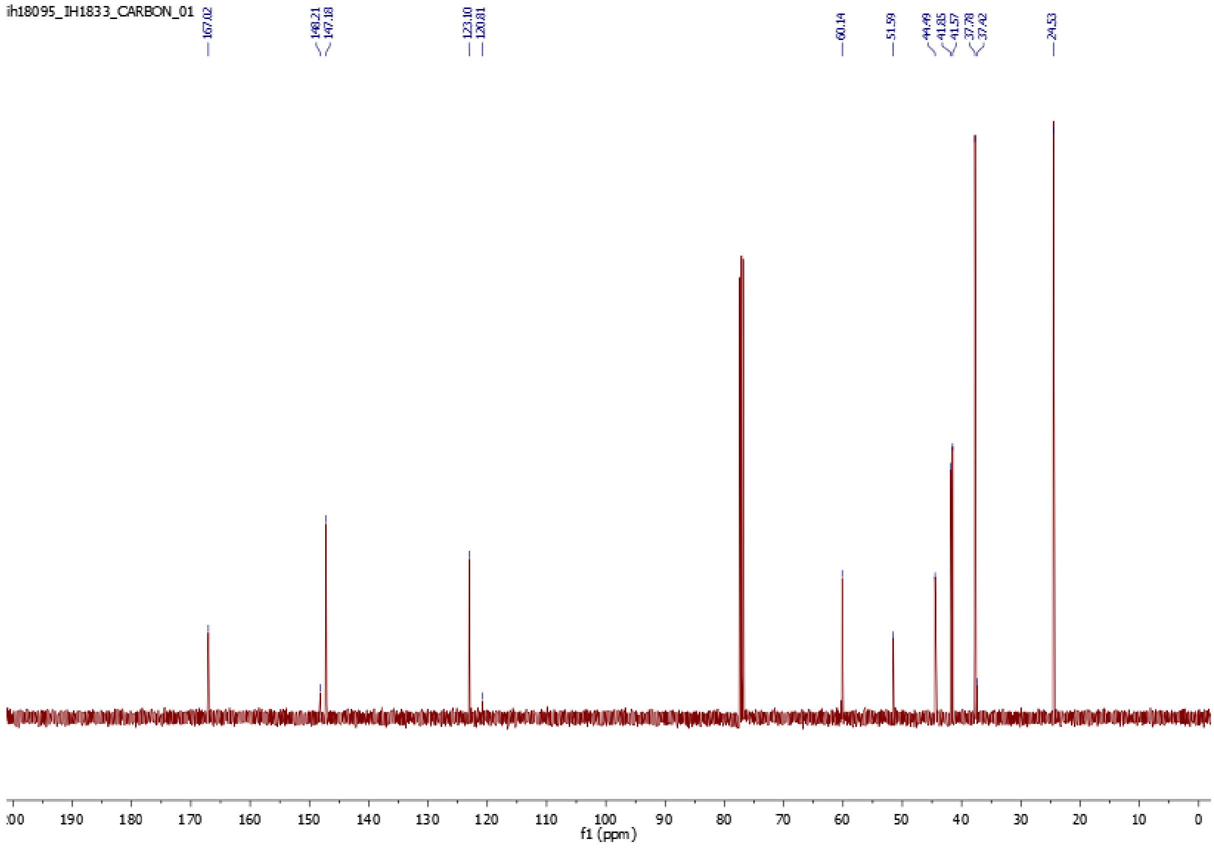
ih18095_IH1833_CARBON_01
167.02
148.21
147.18
123.10
120.81
60.14
51.59
44.49
41.85
41.57
37.78
37.42
24.53
f1 (ppm)

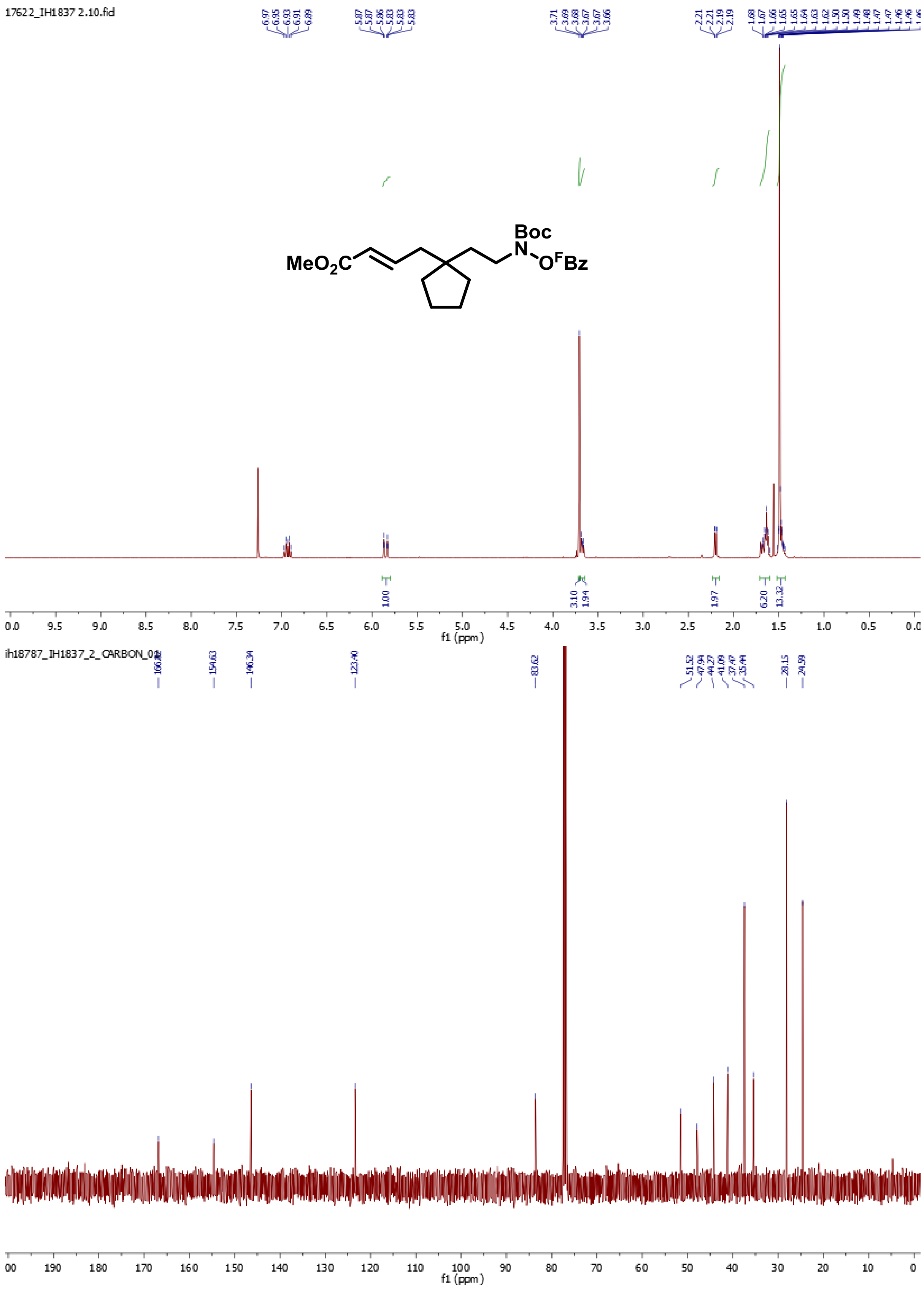
17622_IH1837 2.10.fid
Boc
N
MeO2C
OFBz
f1 (ppm)
ih18787_IH1837_2_CARBON_01
166.80
154.63
146.34
123.40
83.62
51.52
47.94
44.27
41.09
37.47
35.44
28.15
24.59
f1 (ppm)

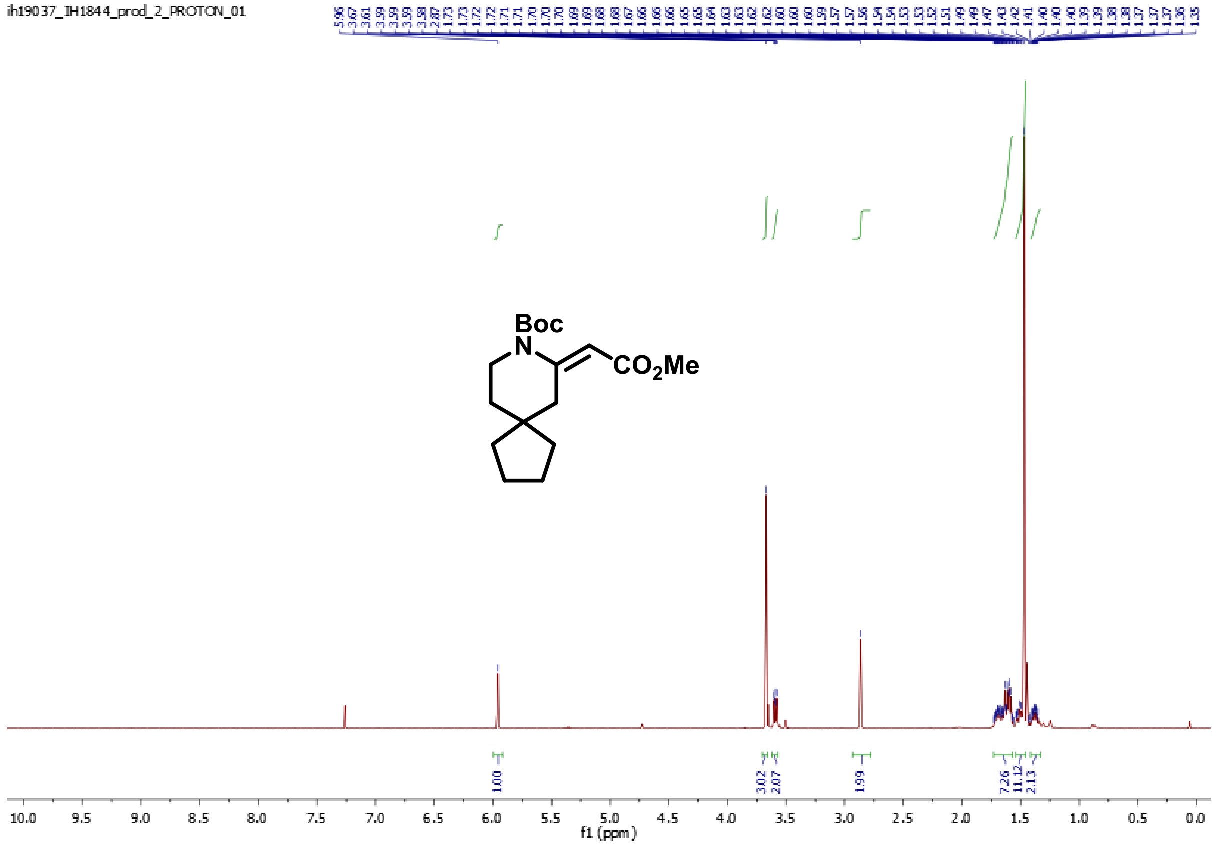

ih19037_IH1844_prod_2_PROTON_01
Boc
N
CO2Me
1.00
3.02
2.07
1.99
7.26
11.12
2.13
f1 (ppm)

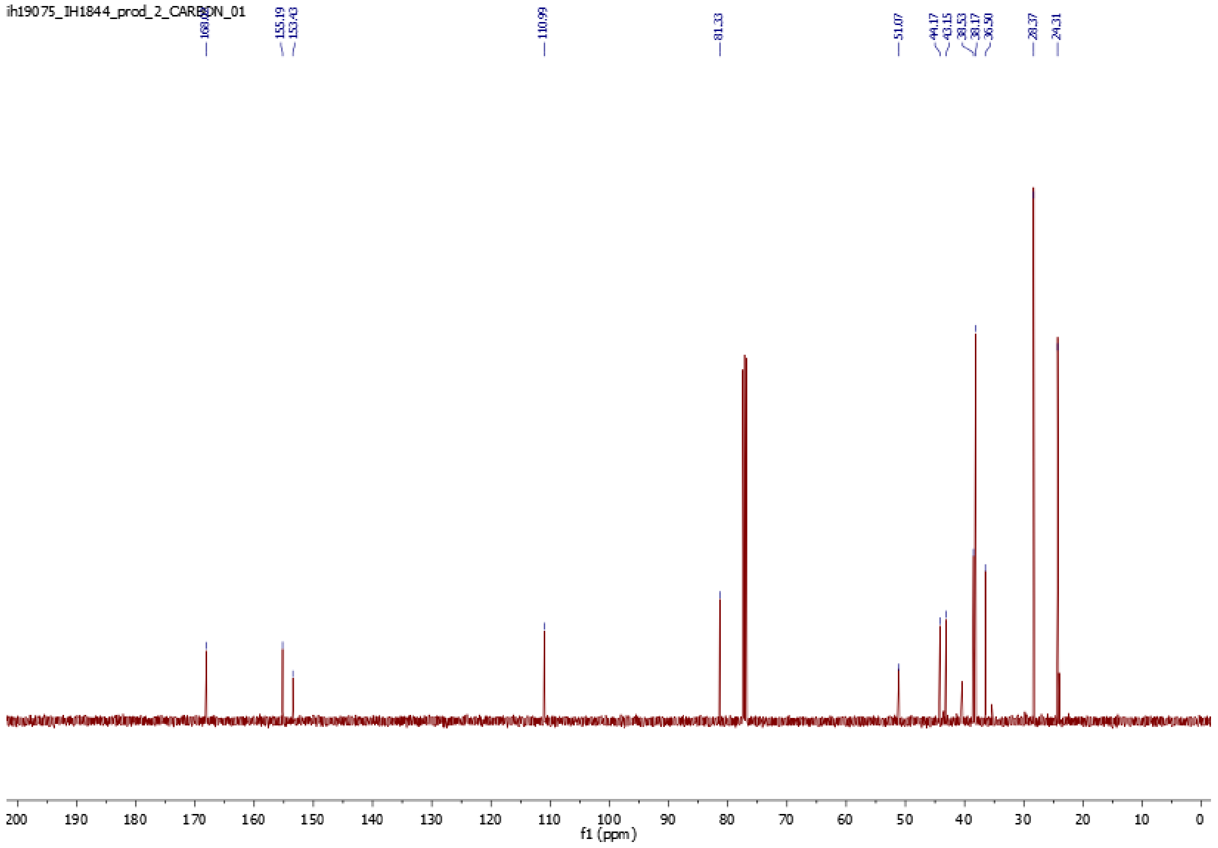

ih19075_IH1844_prod_2_CARBON_01
155.19
153.43
110.99
81.33
51.07
44.17
43.15
38.53
38.17
36.50
28.37
24.31
f1 (ppm)

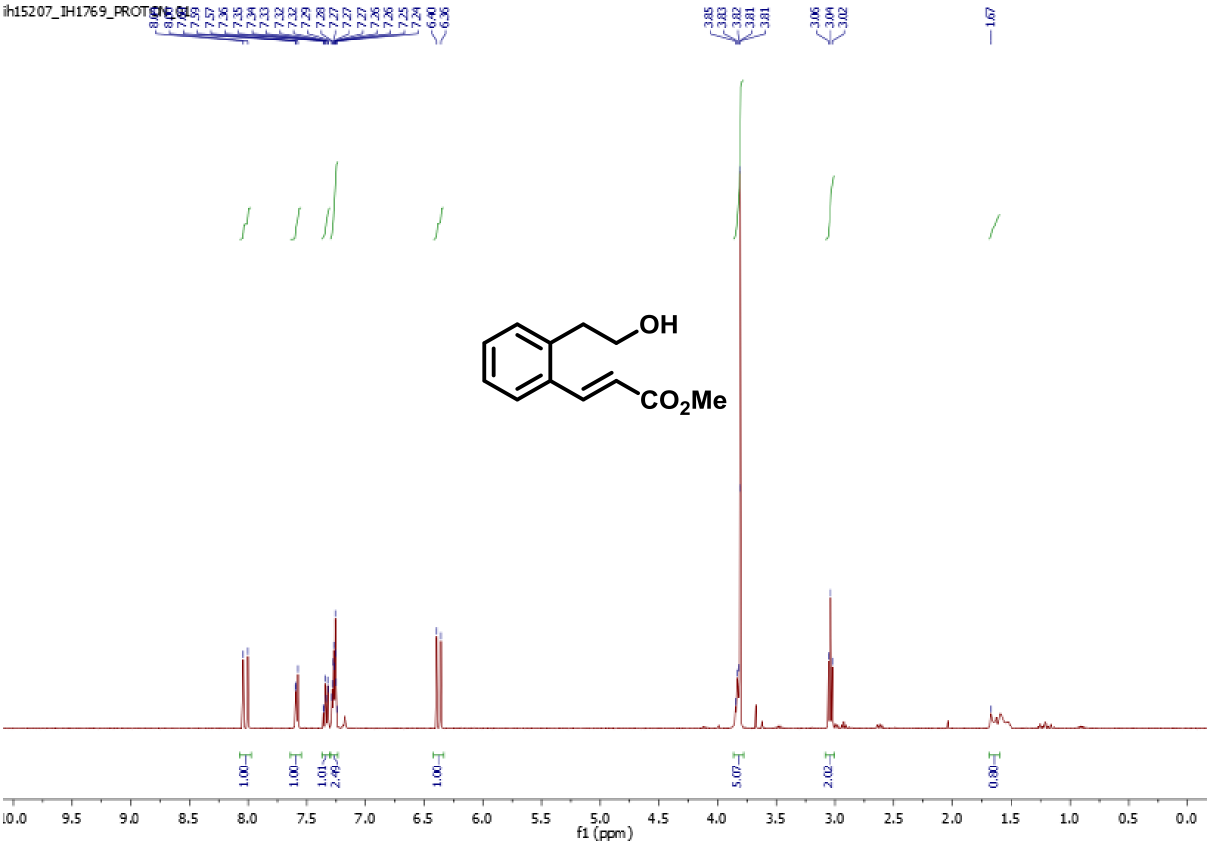
ih15207_IH1769_PROTON_01
6.40
6.36
3.85
3.83
3.82
3.81
3.81
3.06
3.04
3.02
1.67
OH
CO2Me
1.00
1.00
1.01
2.49
1.00
5.07
2.02
0.80
f1 (ppm)

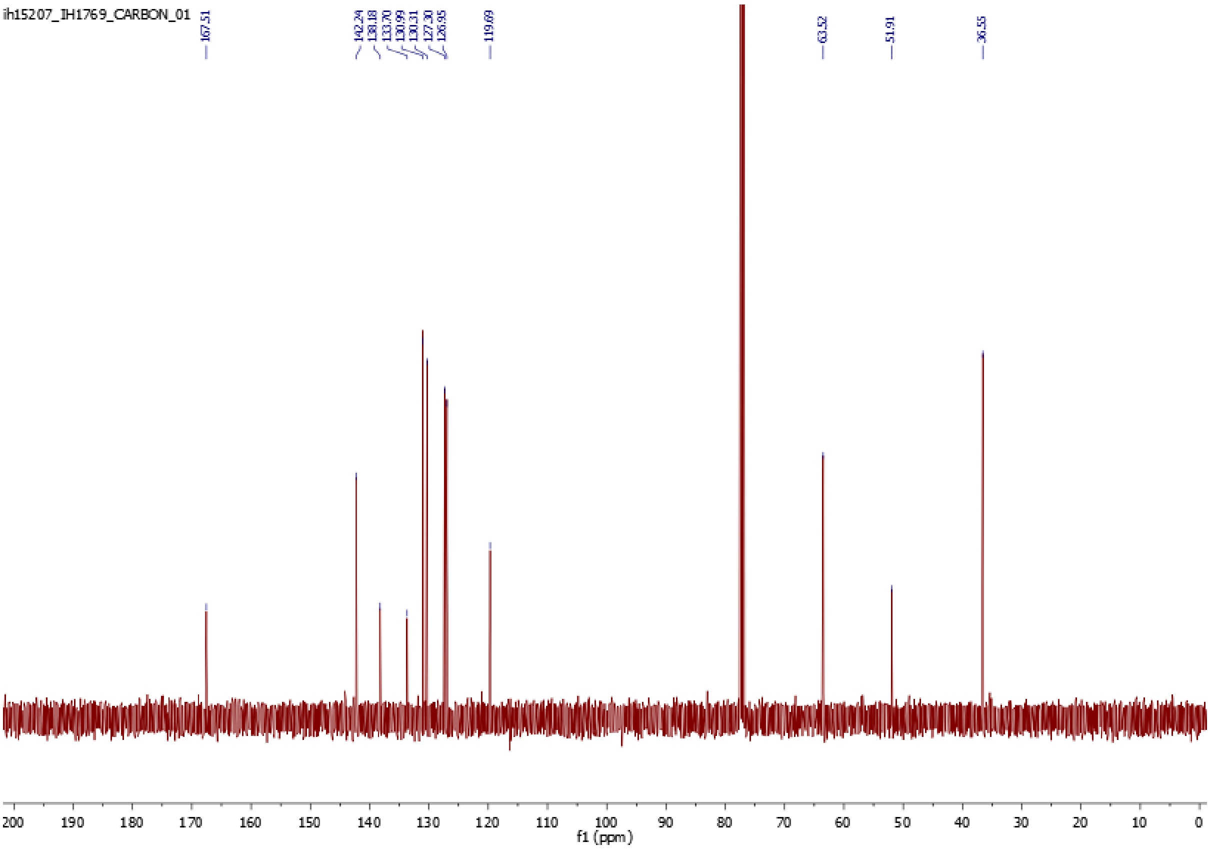
ih15207_IH1769_CARBON_01
167.51
142.24
138.18
133.70
130.99
130.31
127.30
126.95
119.69
63.52
51.91
36.55
f1 (ppm)

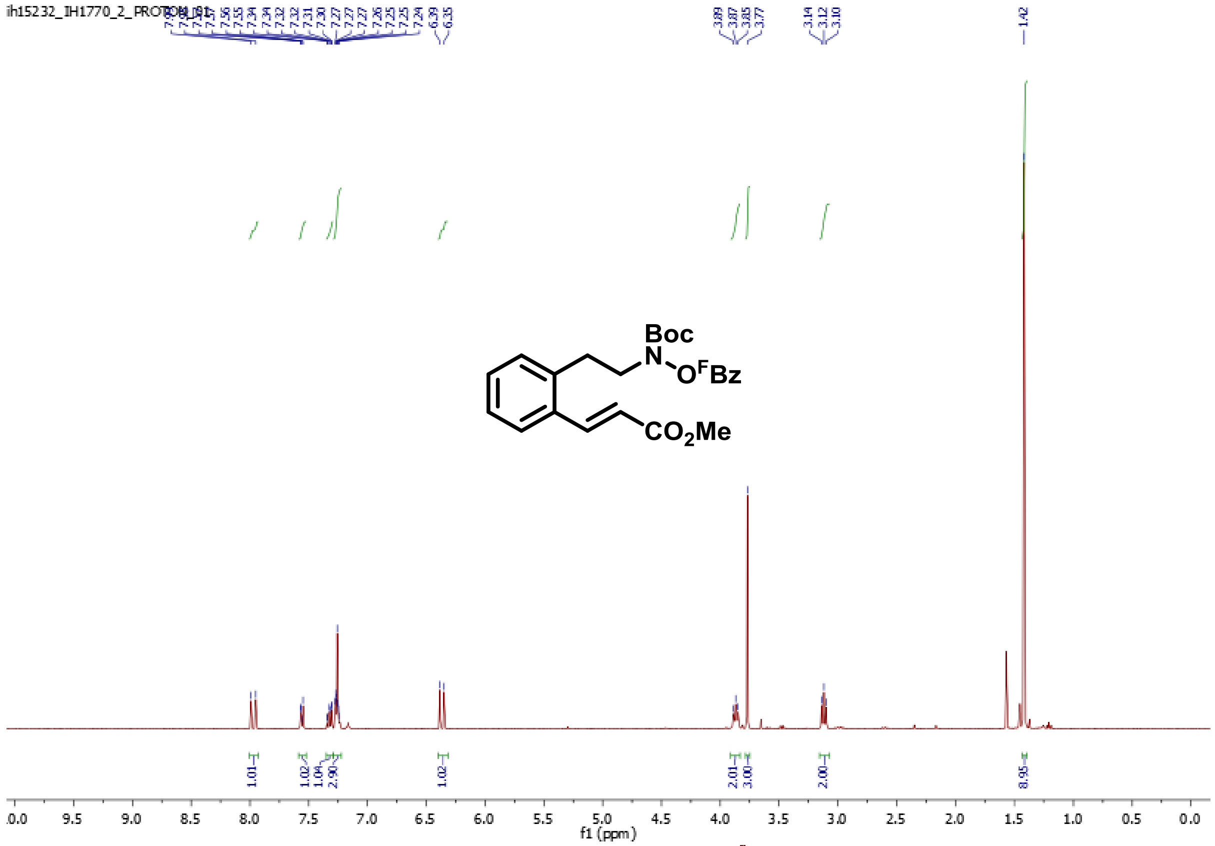
ih15232_IH1770_2_PROTON_01
7.56
7.55
7.34
7.34
7.32
7.32
7.31
7.30
7.27
7.27
7.27
7.26
7.25
7.25
7.24
6.39
6.35
3.89
3.87
3.85
3.77
3.14
3.12
3.10
1.42
Boc
N
O
F
Bz
CO2Me
1.01
1.02
1.04
2.90
1.02
2.01
3.00
2.00
8.95
f1 (ppm)

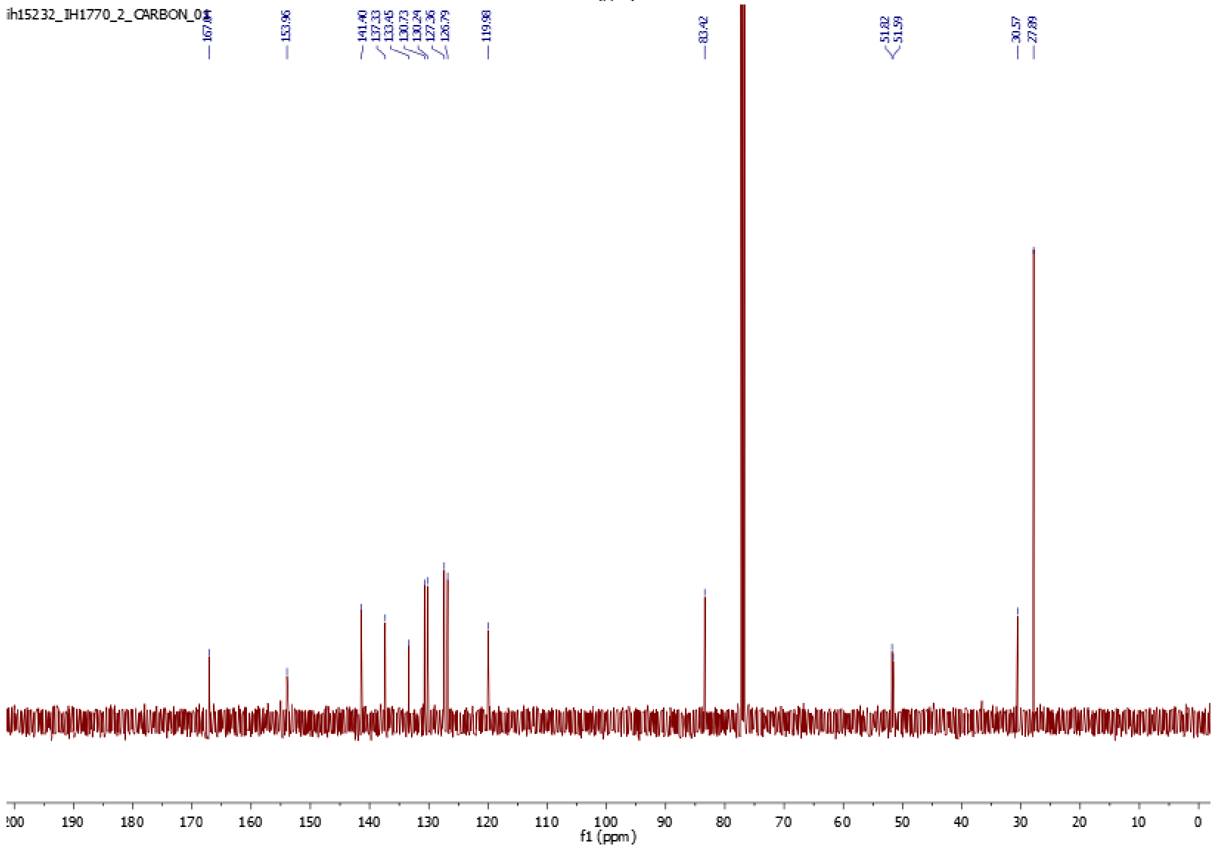
ih15232_IH1770_2_CARBON_01
153.96
141.90
137.33
133.45
130.73
130.24
127.36
126.79
119.98
83.42
51.82
51.59
30.57
27.89
f1 (ppm)

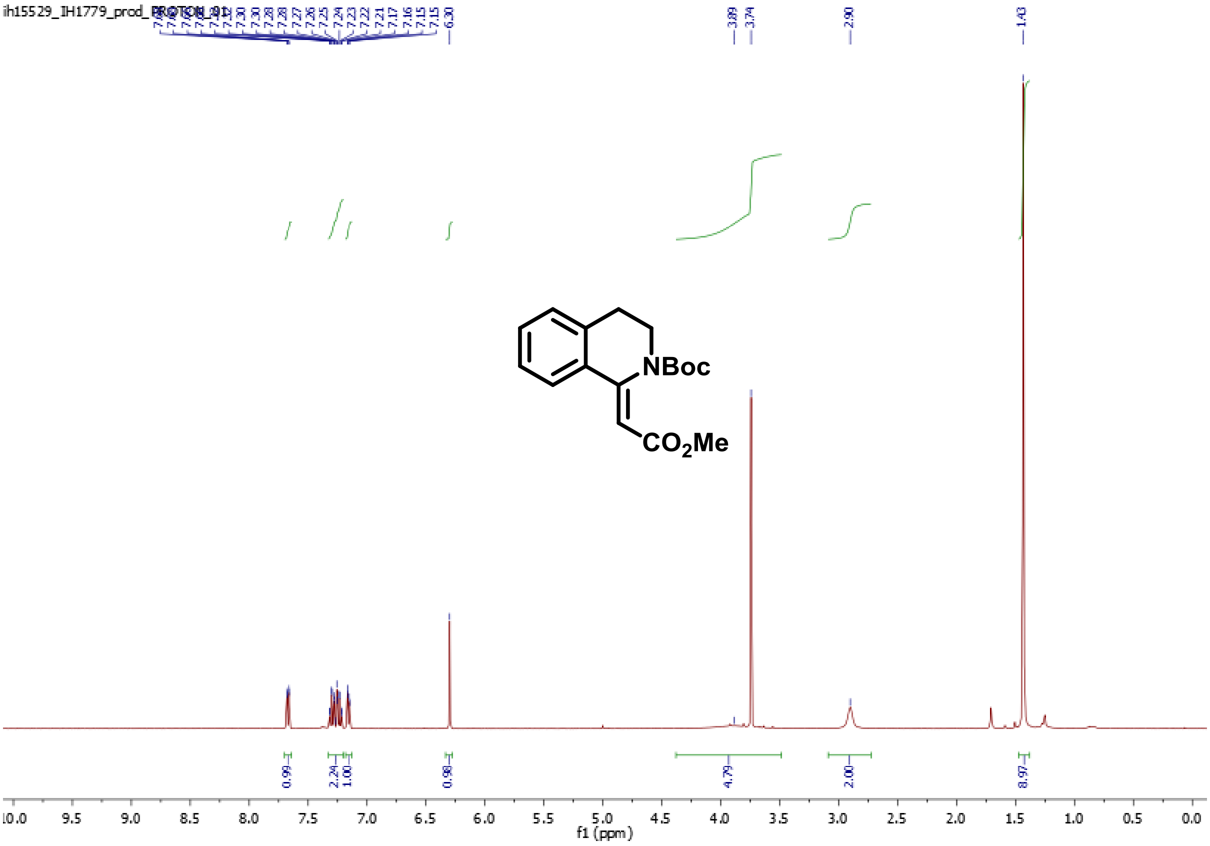
ih15529_IH1779_prod_PROTON_01
6.30
3.89
3.74
2.90
1.43
NBoc
CO2Me
0.99
2.24
1.00
0.98
4.79
2.00
8.97
f1 (ppm)

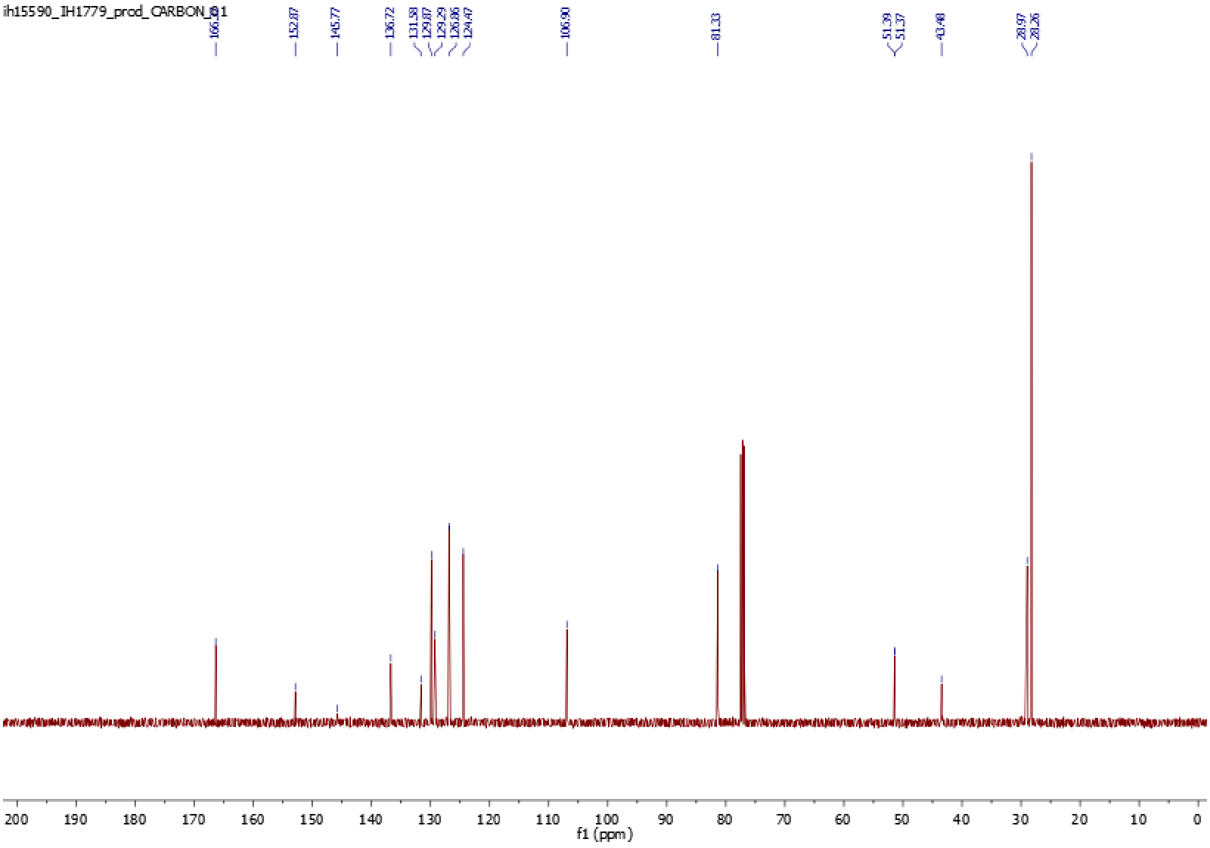
ih15590_IH1779_prod_CARBON_01
152.87
145.77
136.72
131.58
129.87
129.29
126.86
124.47
106.90
81.33
51.39
51.37
43.48
28.97
28.26
f1 (ppm)

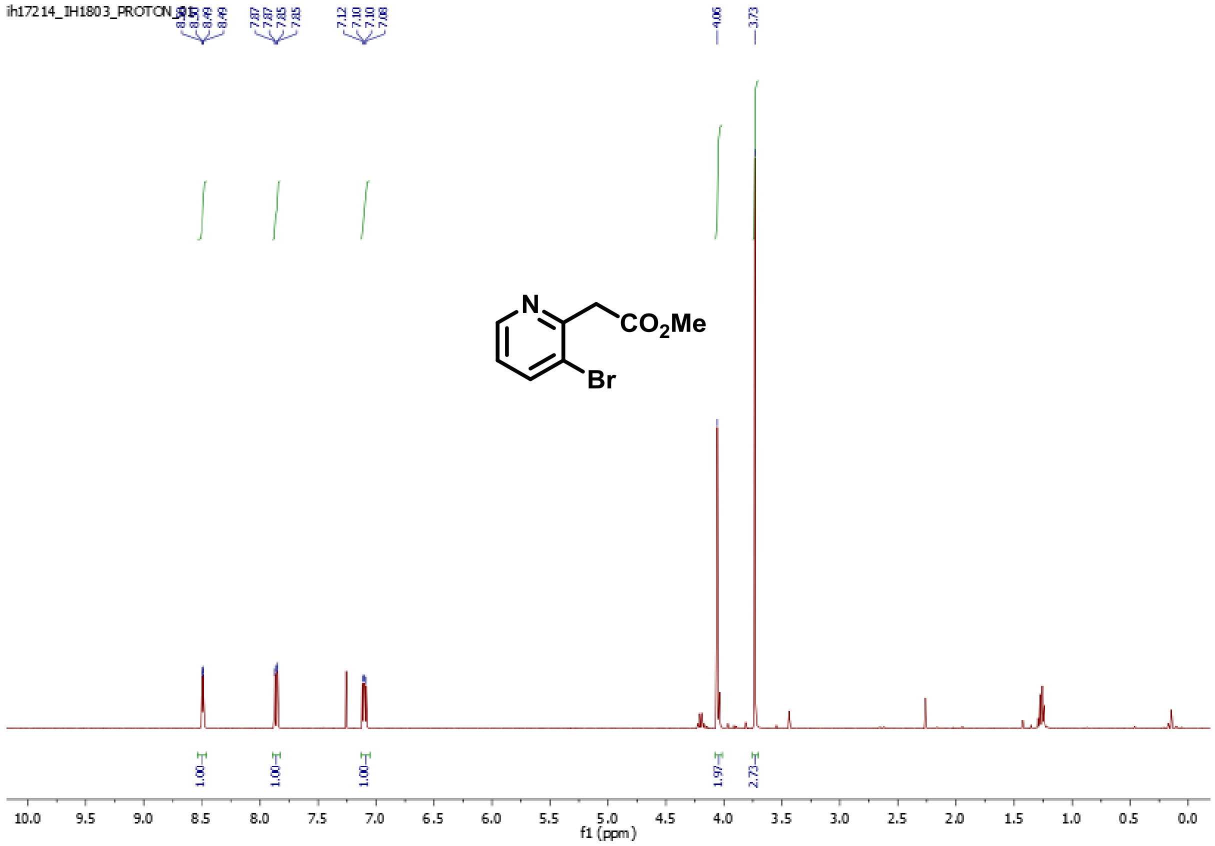
ih17214_IH1803_PROTON_01
4.06
3.73
N
CO_2Me
Br
1.00
1.00
1.00
1.97
2.73
f1 (ppm)

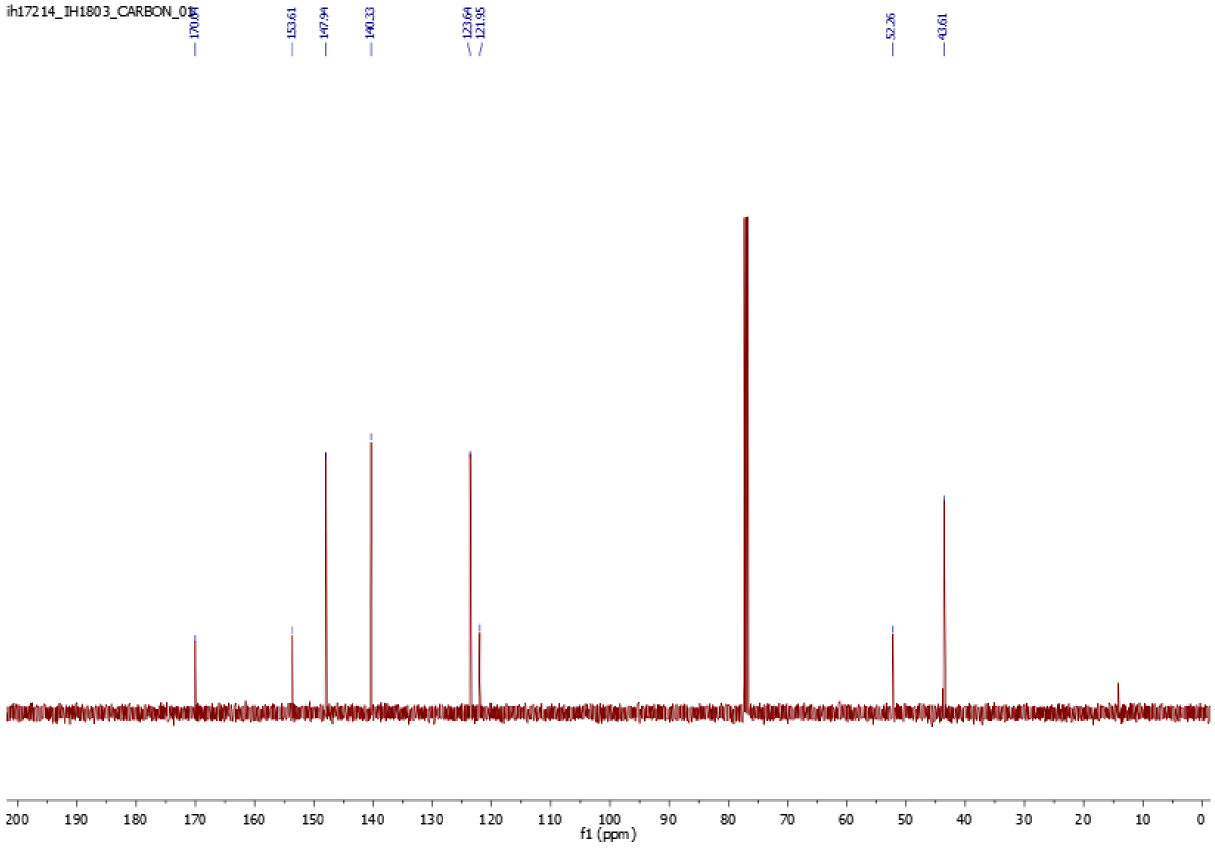
ih17214_IH1803_CARBON_01
153.61
147.94
140.33
123.64
121.95
52.26
43.61
f1 (ppm)

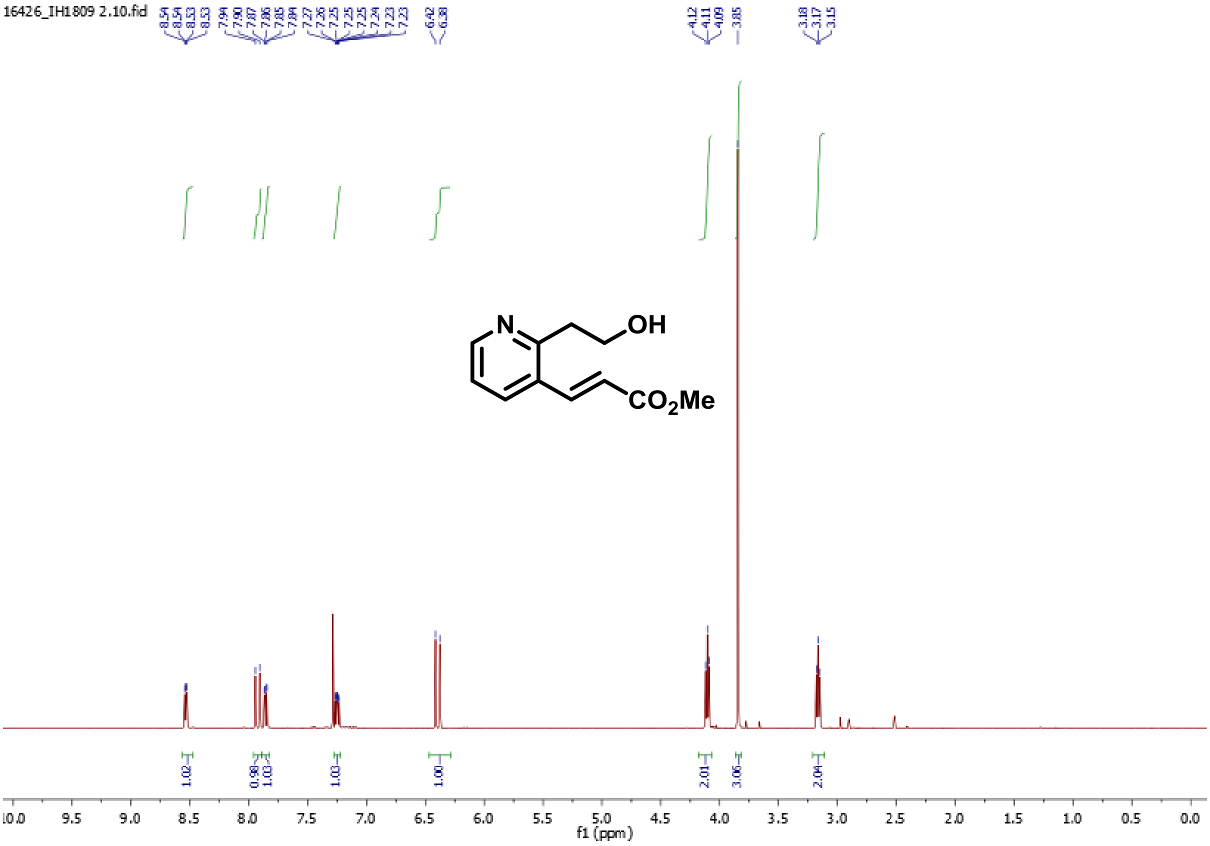
16426_IH1809 2.10.fid
8.54 8.54 8.53 8.53 7.94 7.90 7.87 7.86 7.85 7.84 7.27 7.26 7.25 7.25 7.25 7.24 7.23 7.23 6.42 6.38
4.12 4.11 4.09 3.85
3.18 3.17 3.15
N
OH
CO2Me
1.02 0.98 1.03 1.03 1.00 2.01 3.06 2.04
f1 (ppm)

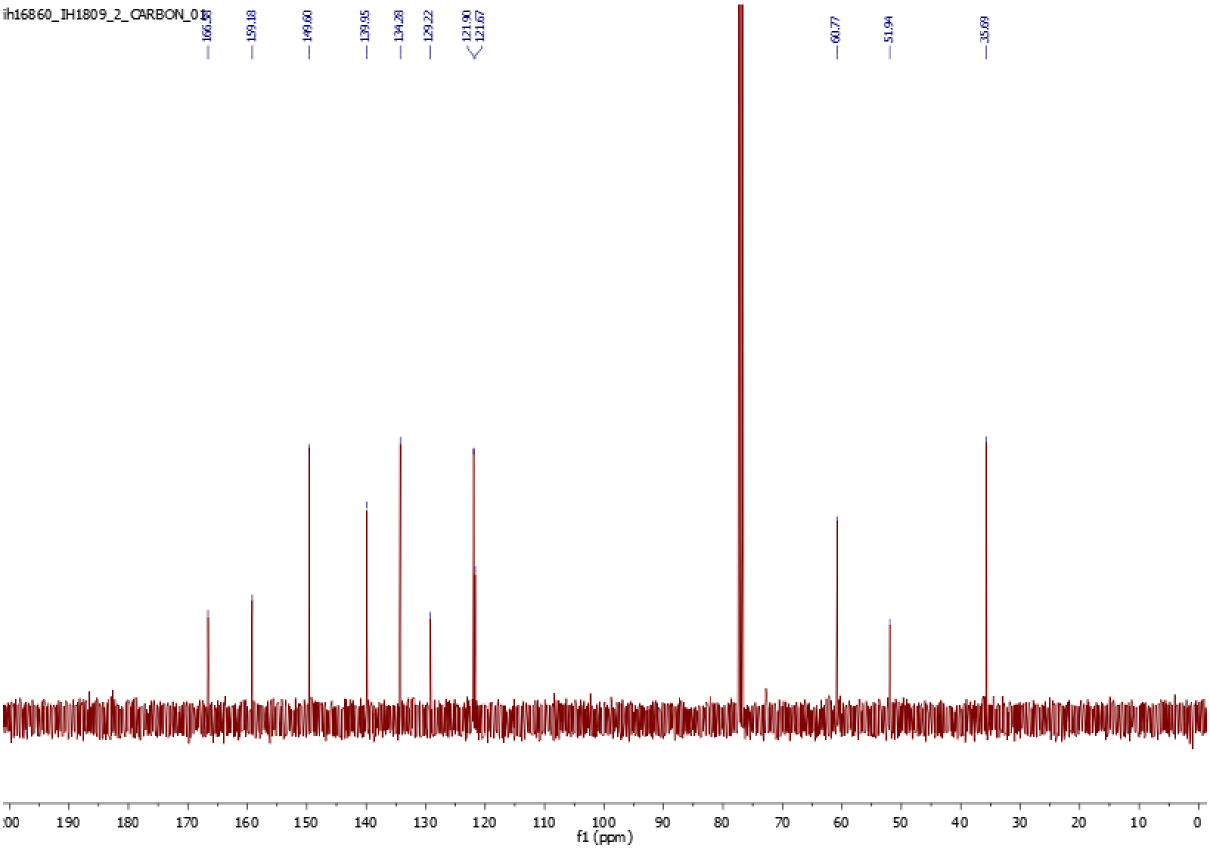
ih16860_IH1809_2_CARBON_01
159.18 149.60 139.95 134.28 129.22 121.90 121.67
60.77 51.94 35.69
f1 (ppm)

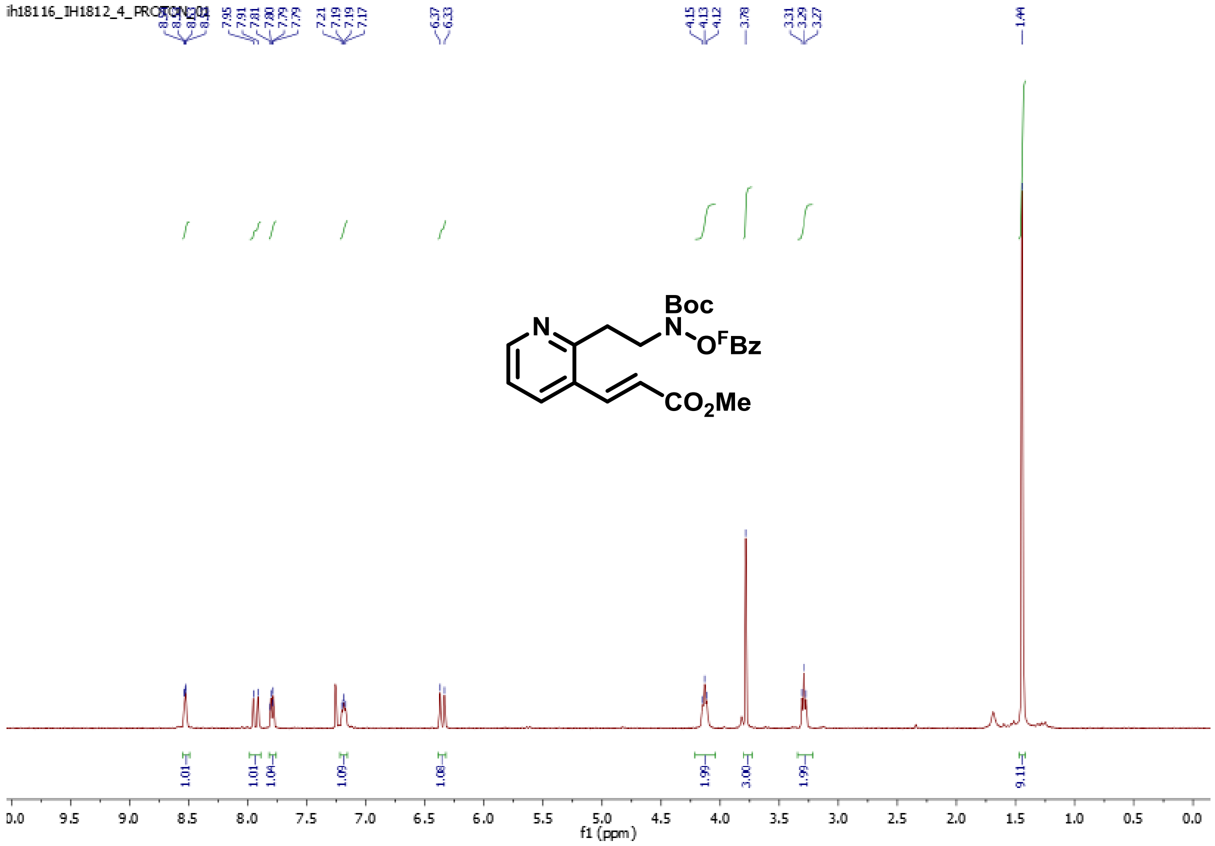
ih18116_IH1812_4_PROTON_01
7.95
7.91
7.81
7.80
7.79
7.79
7.21
7.19
7.19
7.17
6.37
6.33
4.15
4.13
4.12
3.78
3.31
3.29
3.27
1.44
1.01
1.01
1.04
1.09
1.08
1.99
3.00
1.99
9.11
f1 (ppm)
N
Boc
N
O^FBz
CO2Me

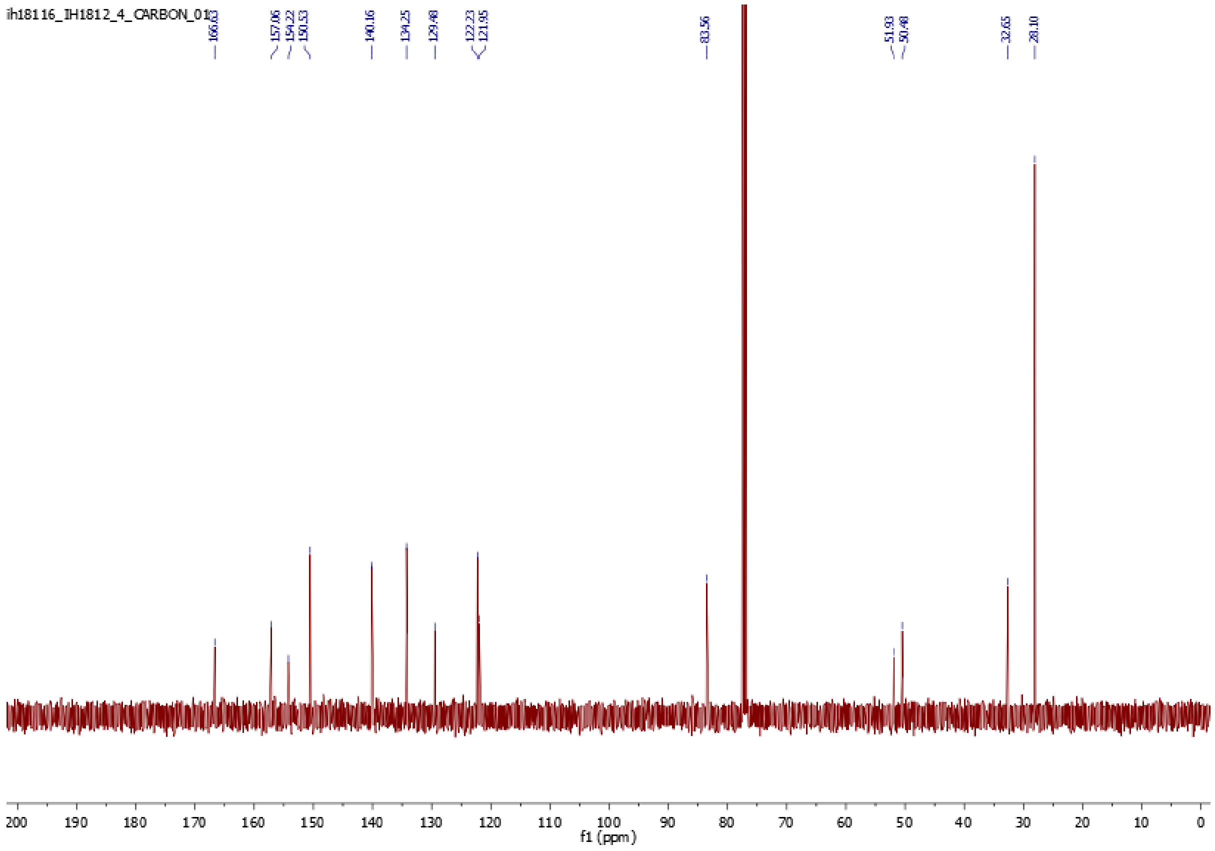
ih18116_IH1812_4_CARBON_01
157.06
154.22
150.53
140.16
134.25
129.48
122.23
121.95
83.56
51.93
50.48
32.65
28.10
f1 (ppm)

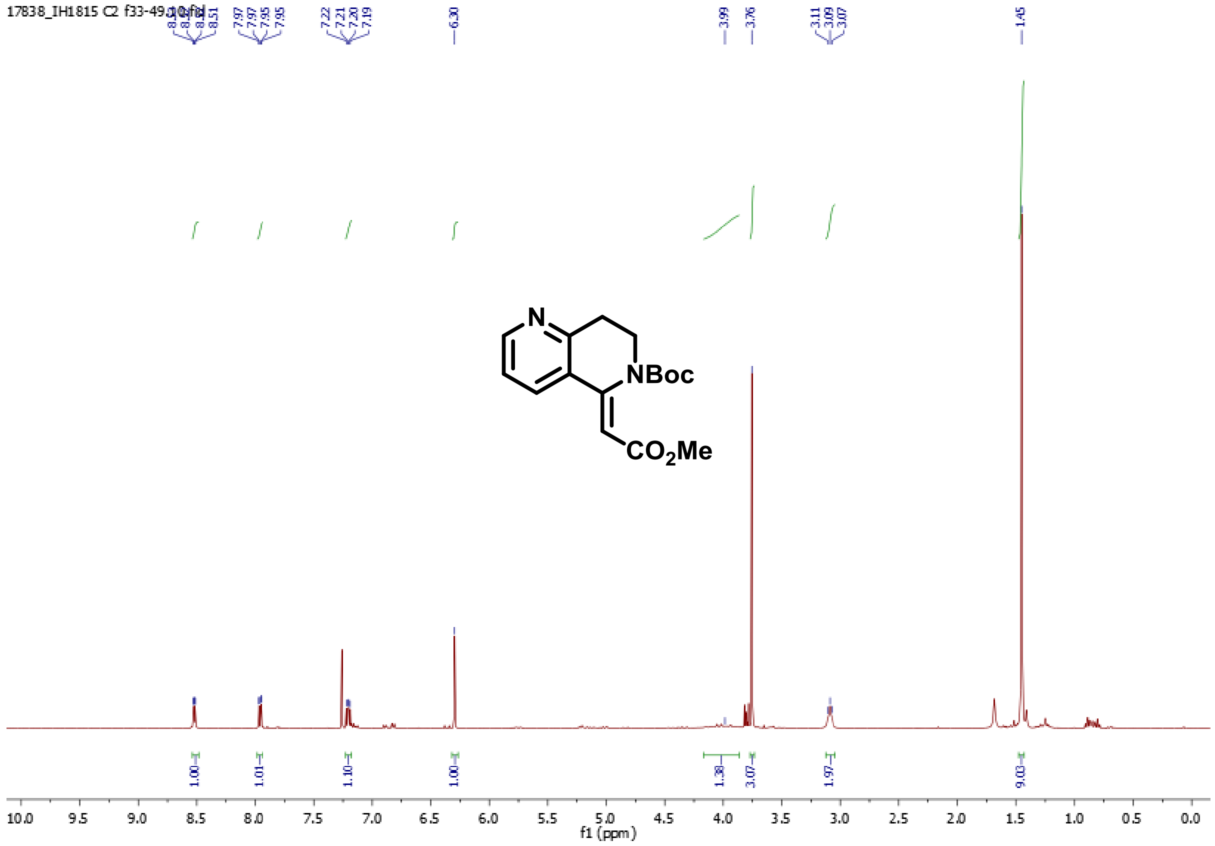
17838_IH1815 C2 f33-49.10.fid
7.97 7.97 7.95 7.95
7.22 7.21 7.20 7.19
6.30
3.99
3.76
3.11 3.09 3.07
1.45
N
NBoc
CO_2Me
1.00
1.01
1.10
1.00
1.38
3.07
1.97
9.03
10.0 9.5 9.0 8.5 8.0 7.5 7.0 6.5 6.0 5.5 5.0 4.5 4.0 3.5 3.0 2.5 2.0 1.5 1.0 0.5 0.0
f1 (ppm)

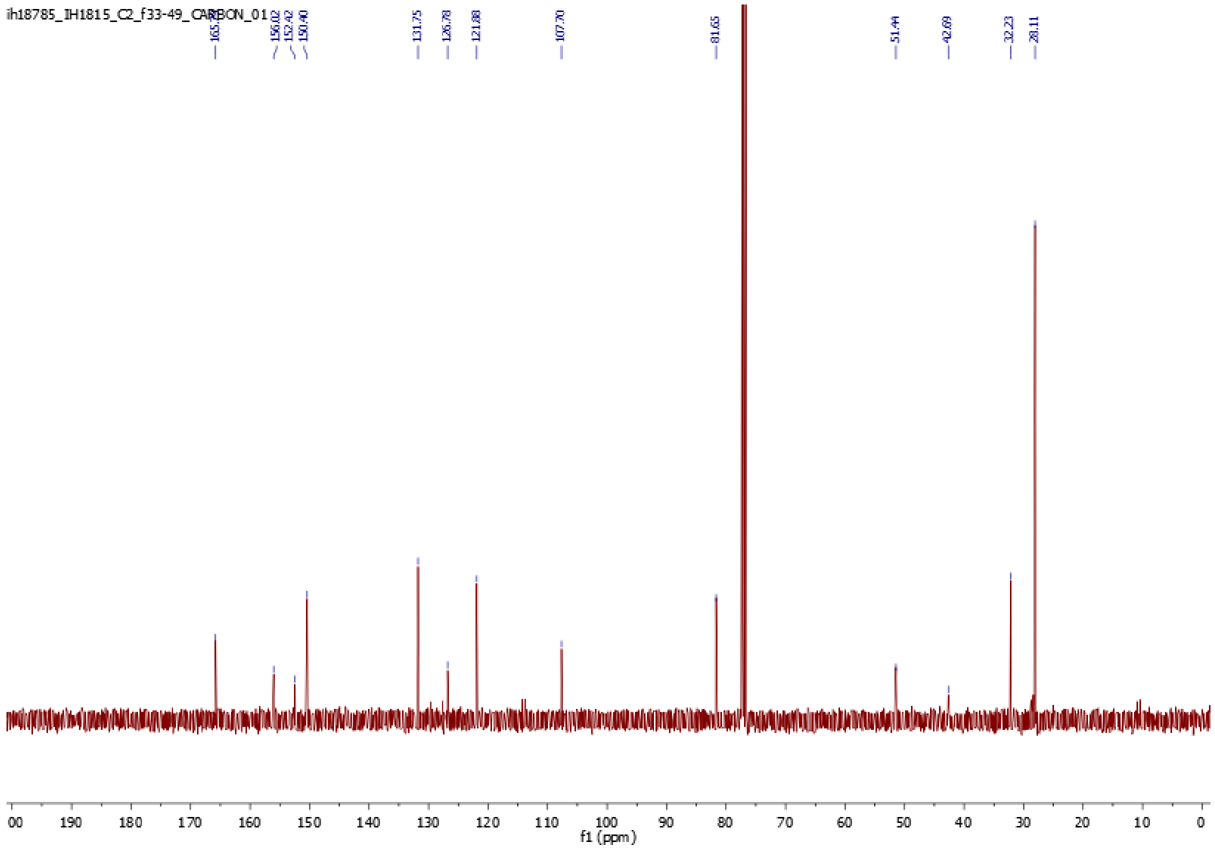
ih18785_IH1815_C2_f33-49_CARBON_01
156.02 152.42 150.40
131.75
126.78
121.88
107.70
81.65
51.44
42.69
32.23
28.11
190 180 170 160 150 140 130 120 110 100 90 80 70 60 50 40 30 20 10 0
f1 (ppm)

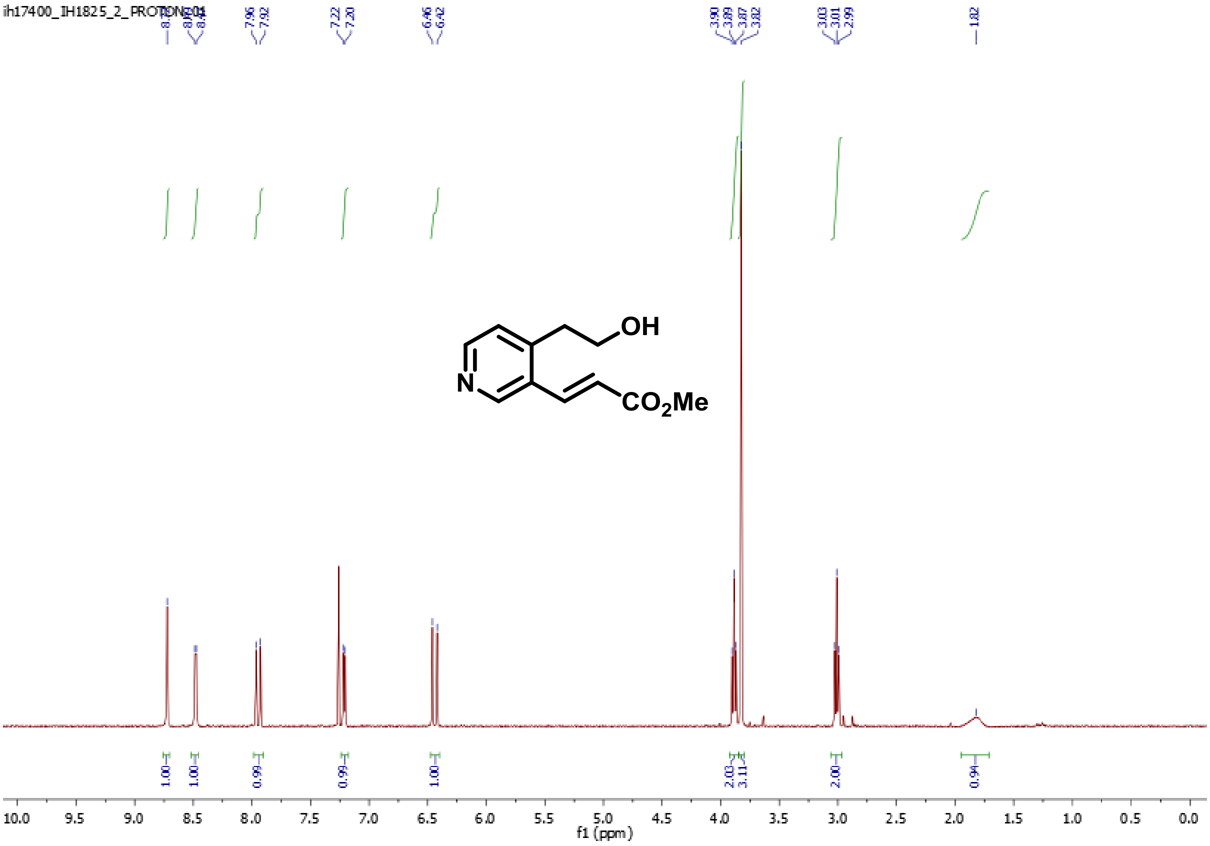
ih17400_IH1825_2_PROTON_01
7.96
7.92
7.22
7.20
6.46
6.42
3.90
3.89
3.87
3.82
3.03
3.01
2.99
1.82
OH
N
CO2Me
1.00
1.00
0.99
0.99
1.00
2.03
3.11
2.00
0.94
f1 (ppm)

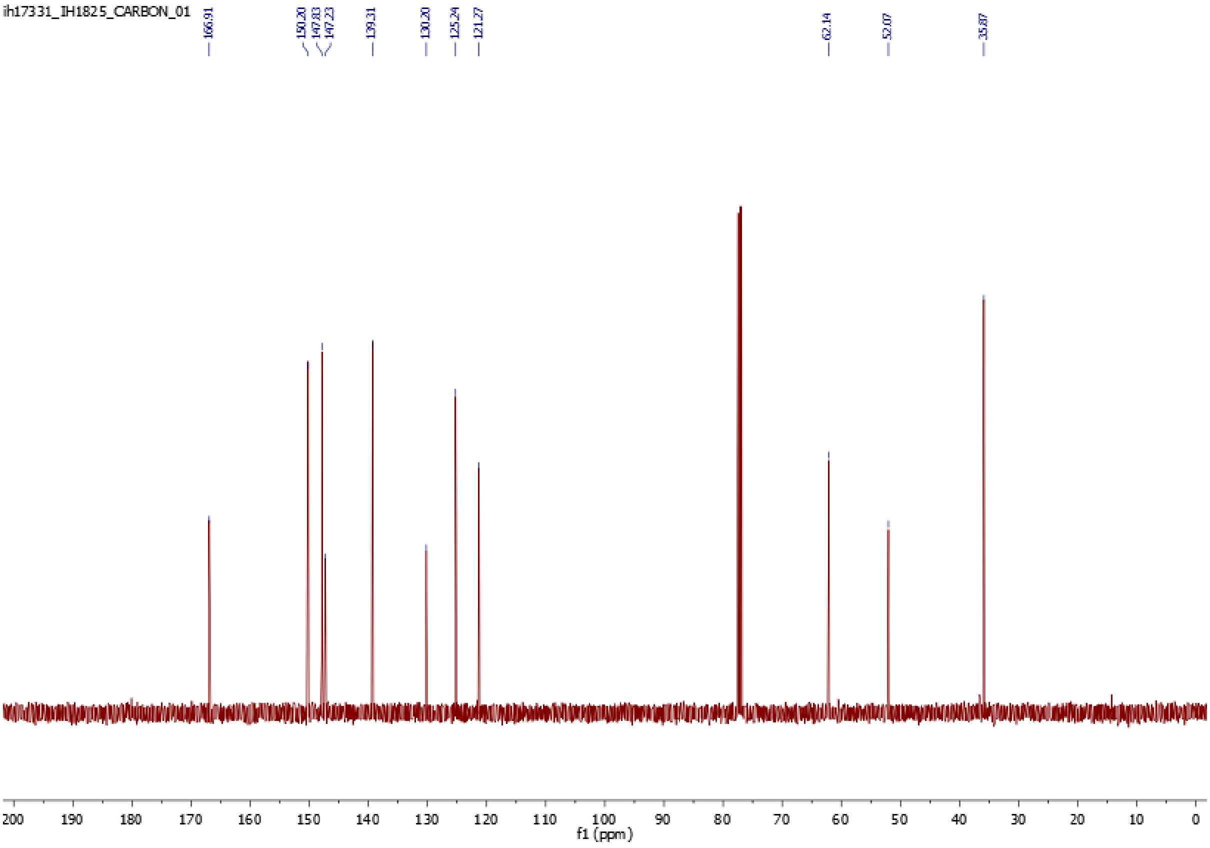
ih17331_IH1825_CARBON_01
166.91
150.20
147.83
147.23
139.31
130.20
125.24
121.27
62.14
52.07
35.87
f1 (ppm)

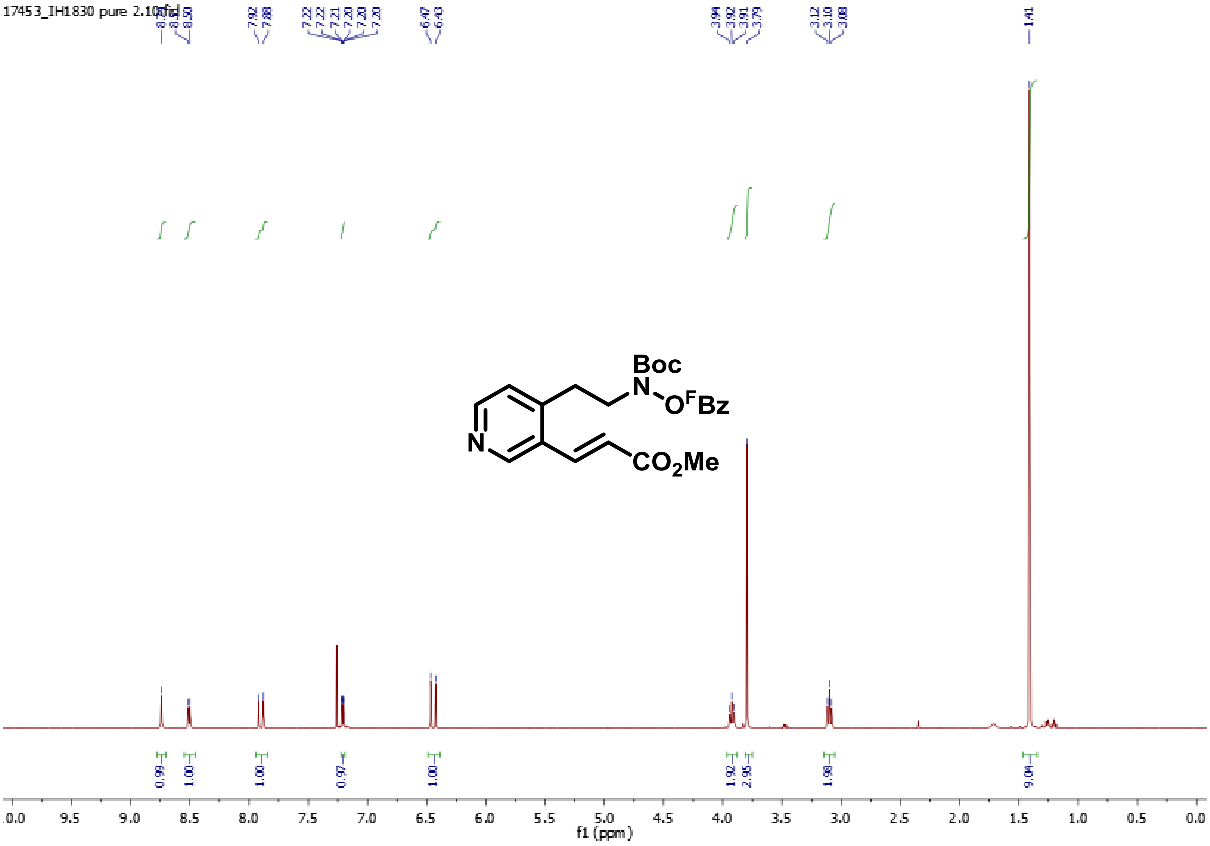
17453_IH1830 pure 2.10.fid
7.92
7.88
7.22
7.22
7.21
7.20
7.20
7.20
6.47
6.43
3.94
3.92
3.91
3.79
3.12
3.10
3.08
1.41
Boc
N
O
F
Bz
N
CO2Me
0.99
1.00
1.00
0.97
1.00
1.92
2.95
1.98
9.04
f1 (ppm)

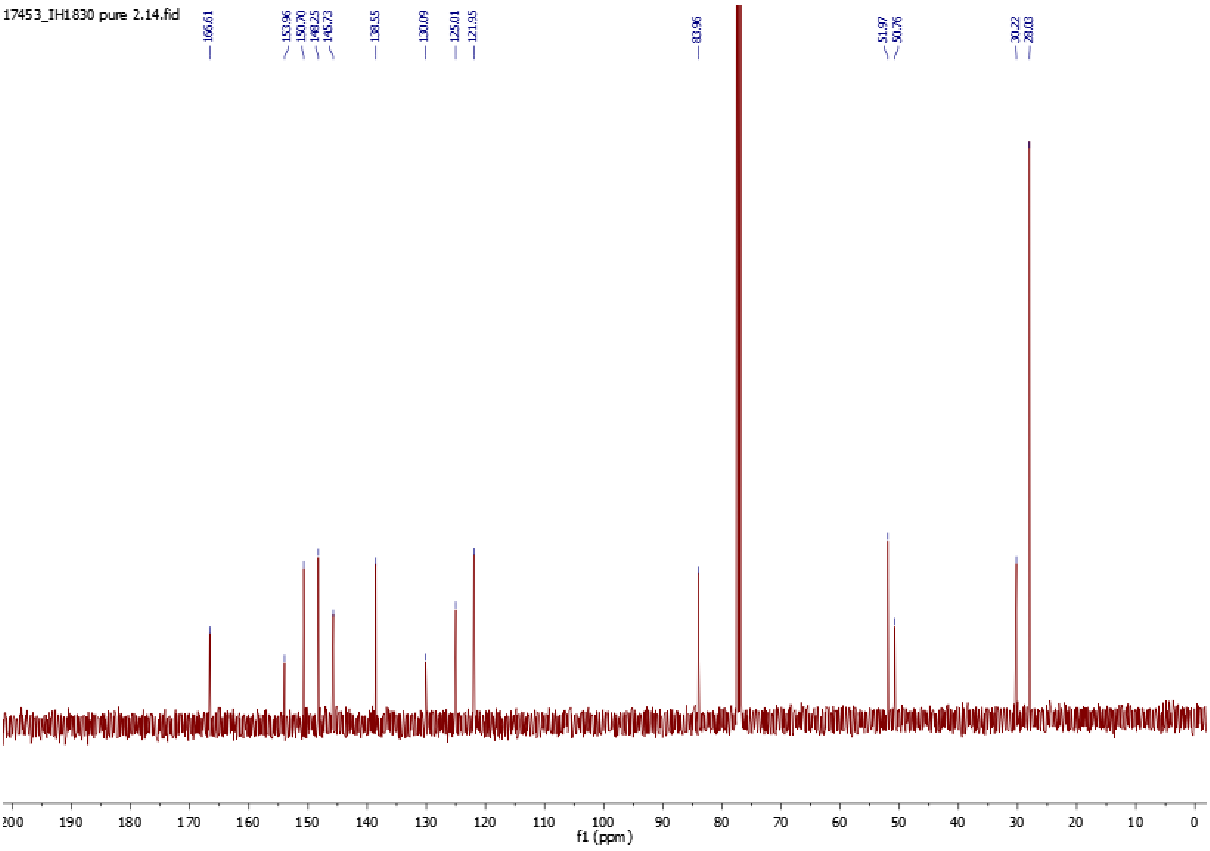
17453_IH1830 pure 2.14.fid
166.61
153.96
150.70
148.25
145.73
138.55
130.09
125.01
121.95
83.96
51.97
50.76
30.22
28.03
f1 (ppm)

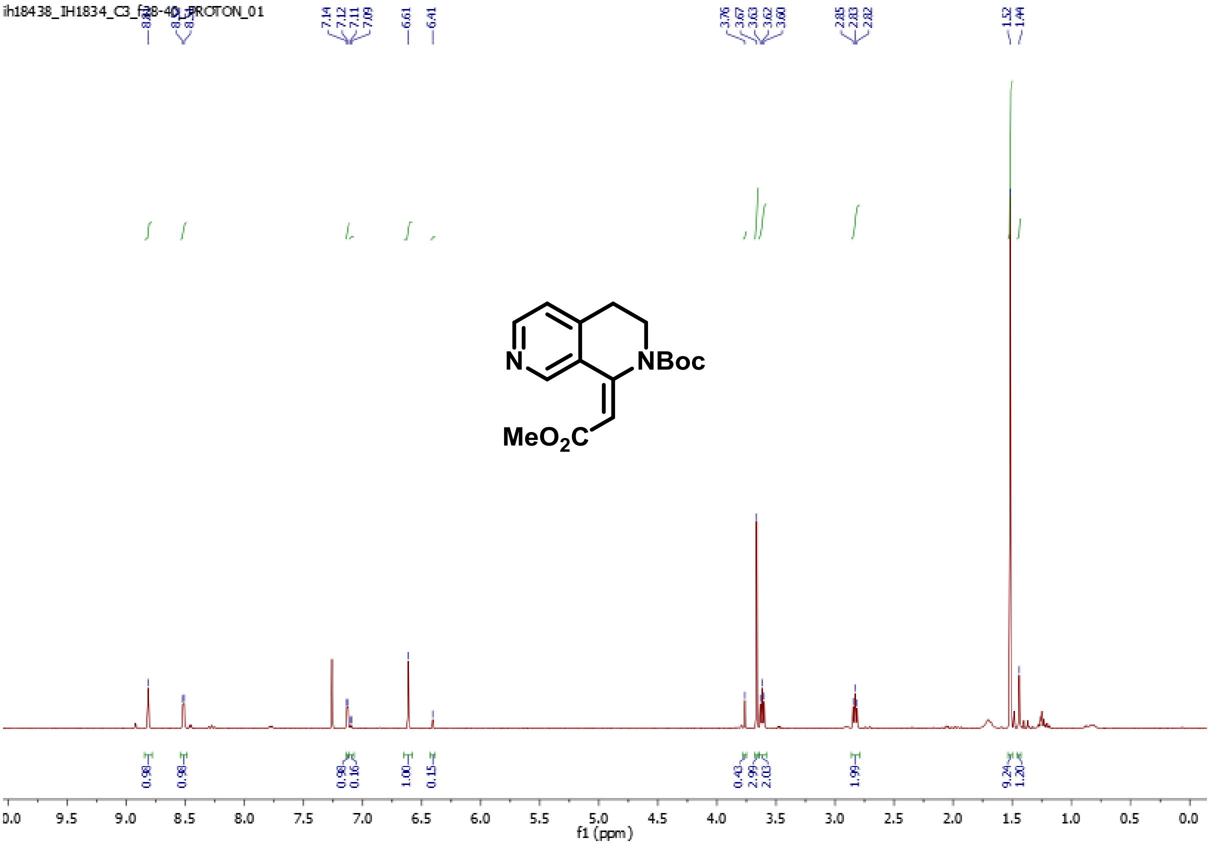
ih18438_IH1834_C3_f28-40_PROTON_01
7.14
7.12
7.11
7.09
6.61
6.41
3.76
3.67
3.63
3.62
3.60
2.85
2.83
2.82
1.52
1.44
N
NBoc
MeO2C
0.98
0.98
0.98
0.16
1.00
0.15
0.43
2.99
2.03
1.99
9.24
1.20
f1 (ppm)

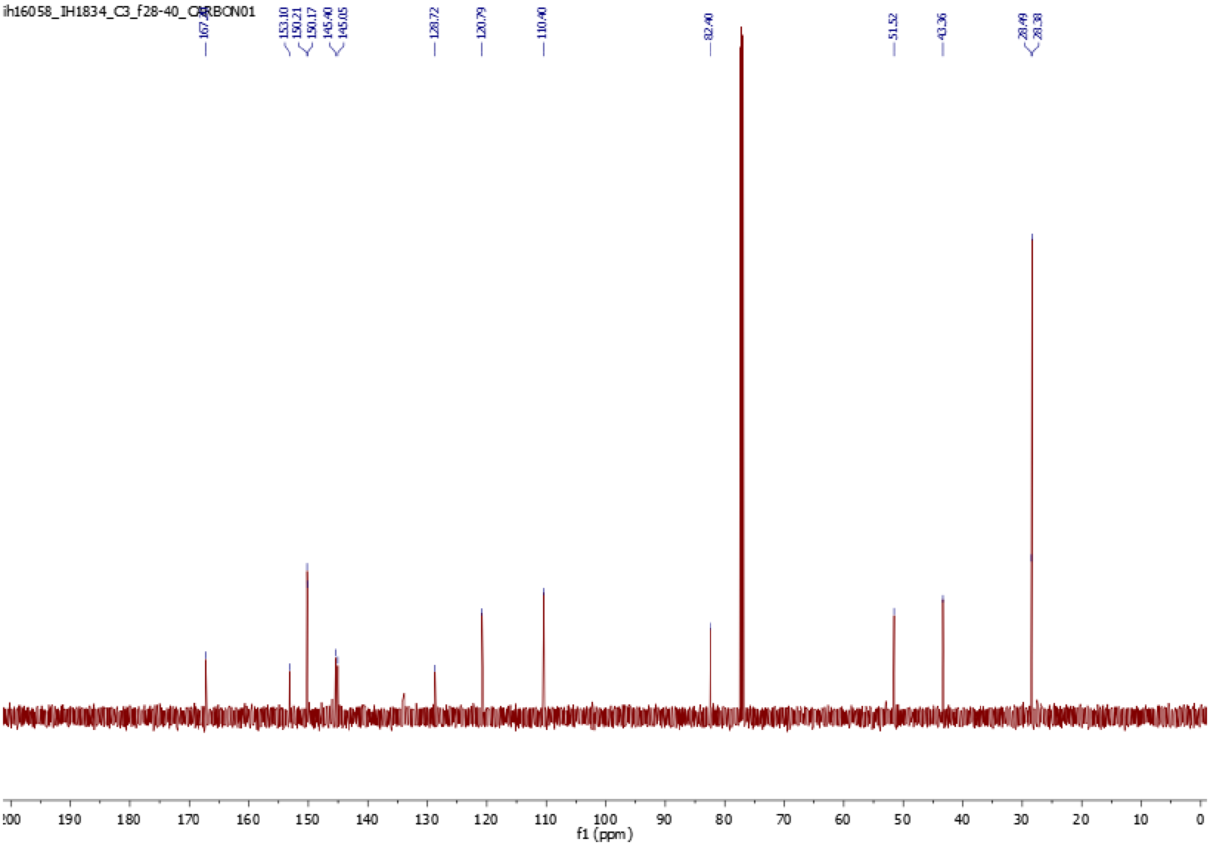
ih16058_IH1834_C3_f28-40_CARBON01
153.10
150.21
150.17
145.40
145.05
128.72
120.79
110.40
82.40
51.52
43.36
28.49
28.38
f1 (ppm)

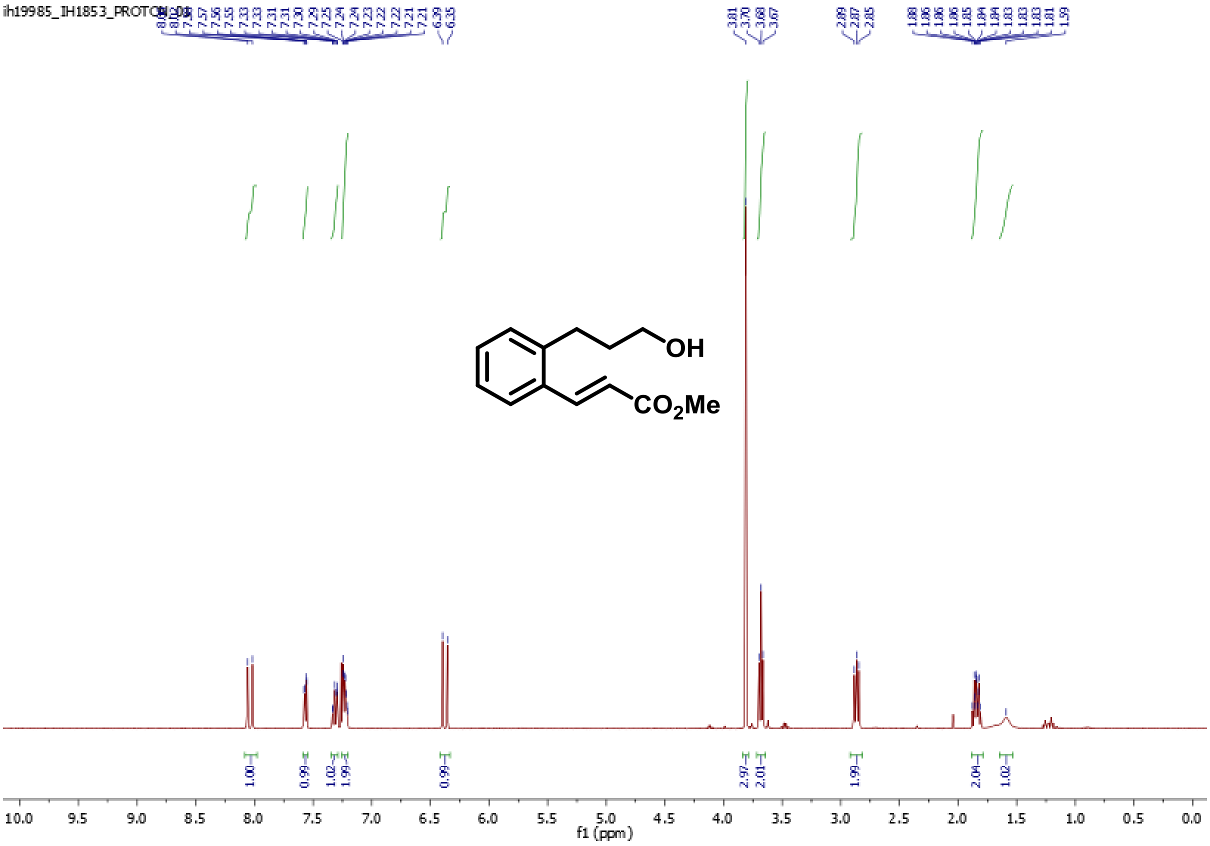
ih19985_IH1853_PROTON_01
OH
CO2Me
1.00
0.99
1.02
1.99
0.99
2.97
2.01
1.99
2.04
1.02
f1 (ppm)

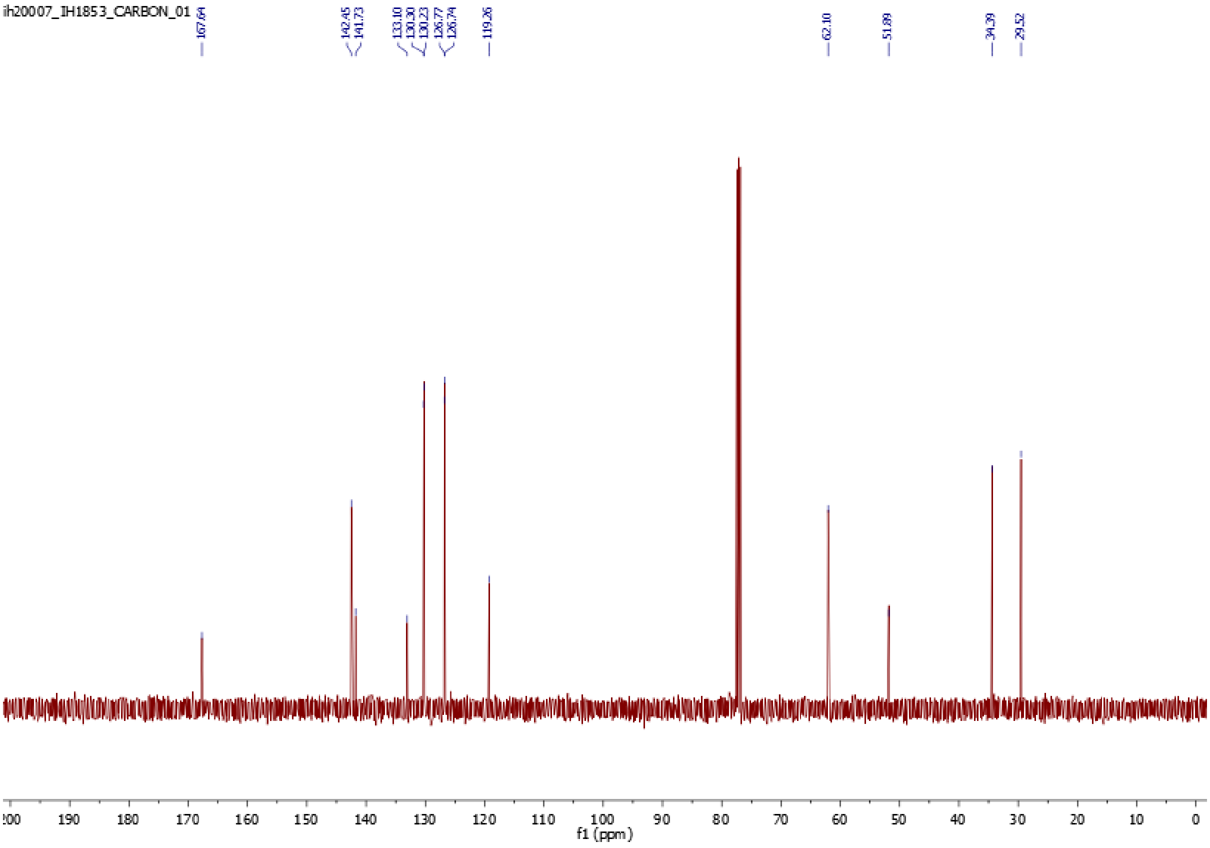
ih20007_IH1853_CARBON_01
167.64
142.45
141.73
133.10
130.30
130.23
126.77
126.74
119.26
62.10
51.89
34.39
29.52
f1 (ppm)

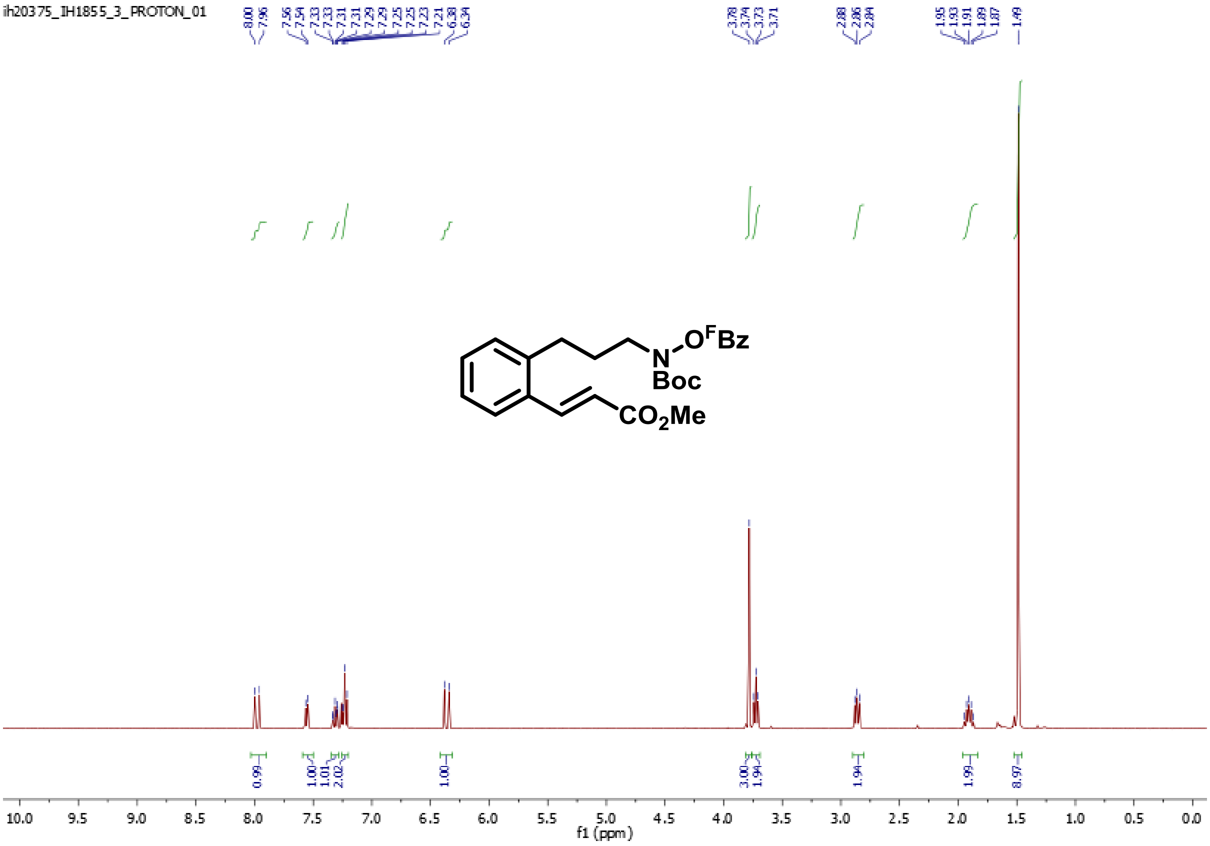
ih20375_IH1855_3_PROTON_01
8.00
7.96
7.56
7.54
7.33
7.31
7.31
7.29
7.29
7.25
7.25
7.23
7.21
6.38
6.34
3.78
3.74
3.73
3.71
2.88
2.86
2.84
1.95
1.93
1.91
1.89
1.87
1.49
N
O
FBz
Boc
CO2Me
0.99
1.00
1.01
2.02
1.00
3.00
1.94
1.94
1.99
8.97
f1 (ppm)

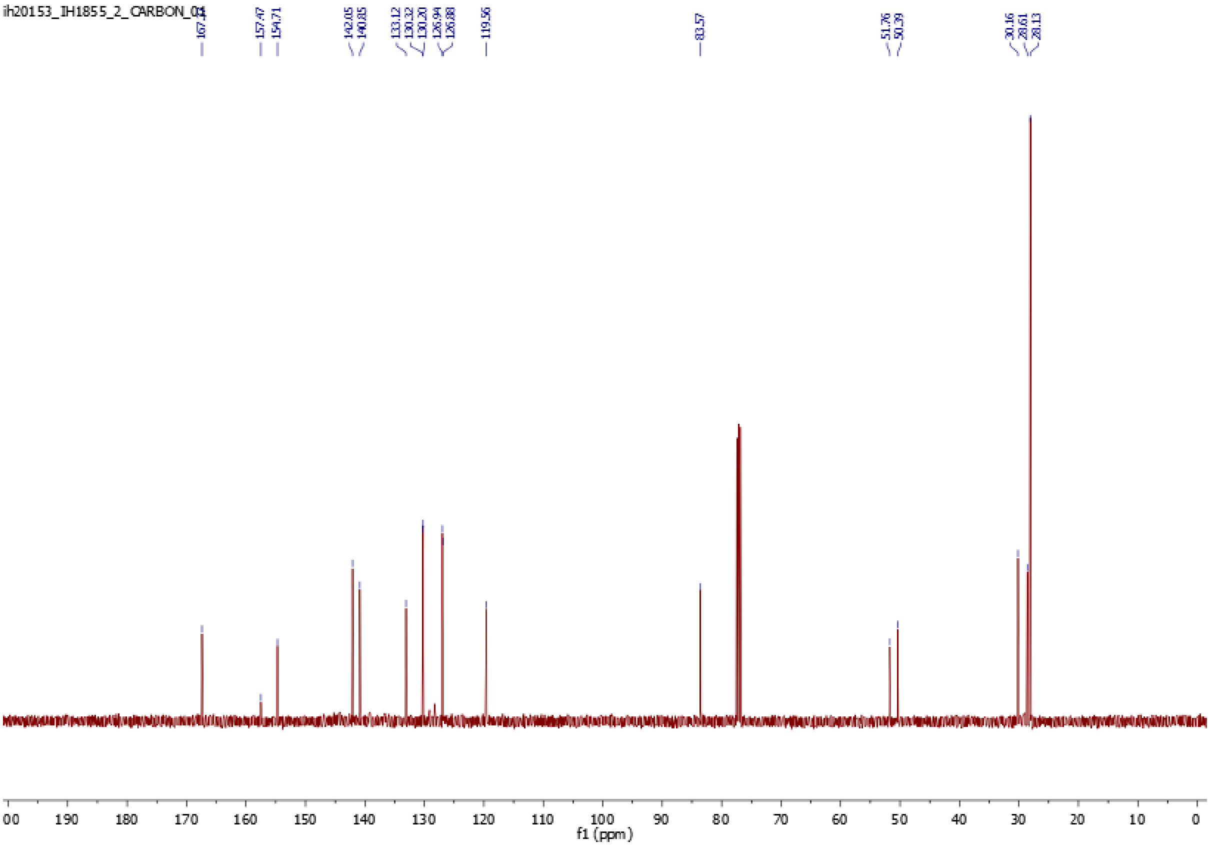
ih20153_IH1855_2_CARBON_01
167.41
157.47
154.71
142.05
140.85
133.12
130.32
130.20
126.94
126.88
119.56
83.57
51.76
50.39
30.16
28.61
28.13
f1 (ppm)

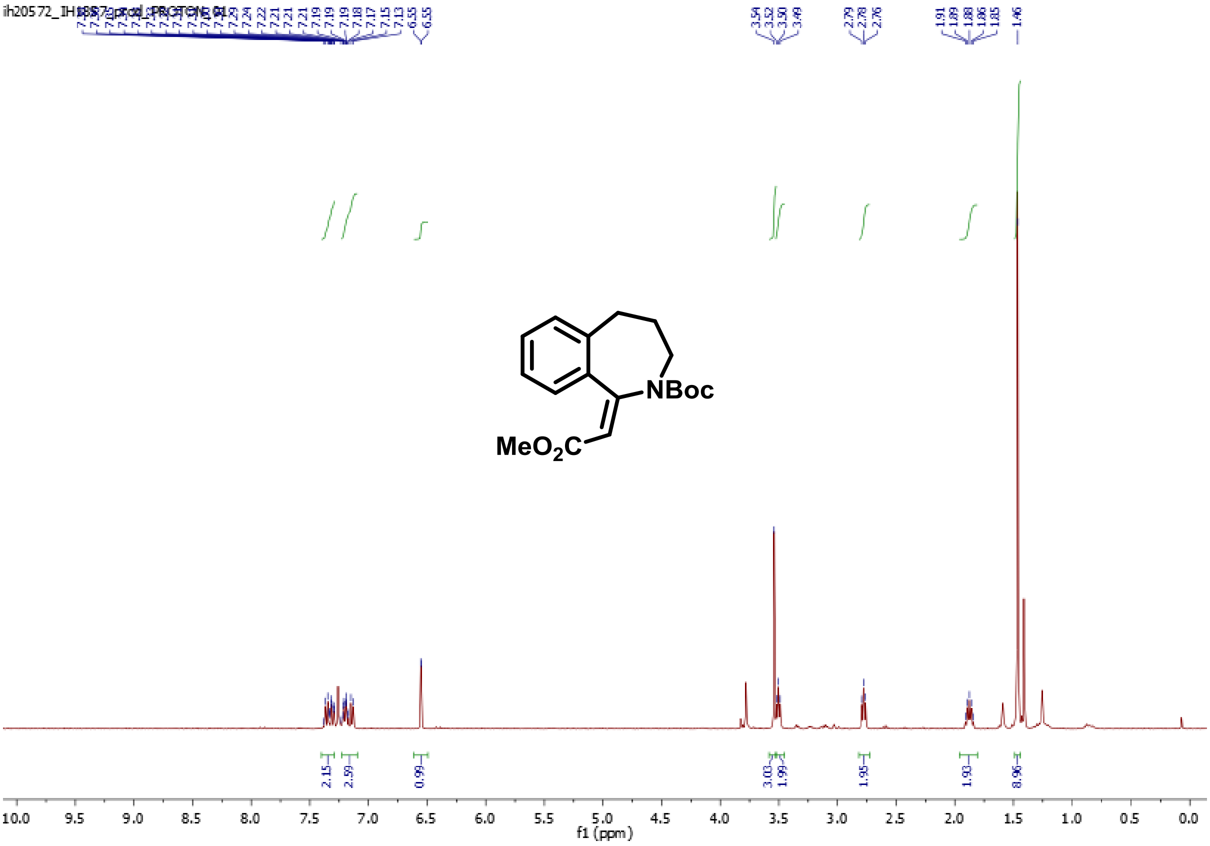
ih20572_IH1857_prod_PROTON_01
6.55
6.55
3.54
3.52
3.50
3.49
2.79
2.78
2.76
1.91
1.89
1.88
1.86
1.85
1.46
NBoc
MeO2C
2.15
2.59
0.99
3.03
1.99
1.95
1.93
8.96
f1 (ppm)

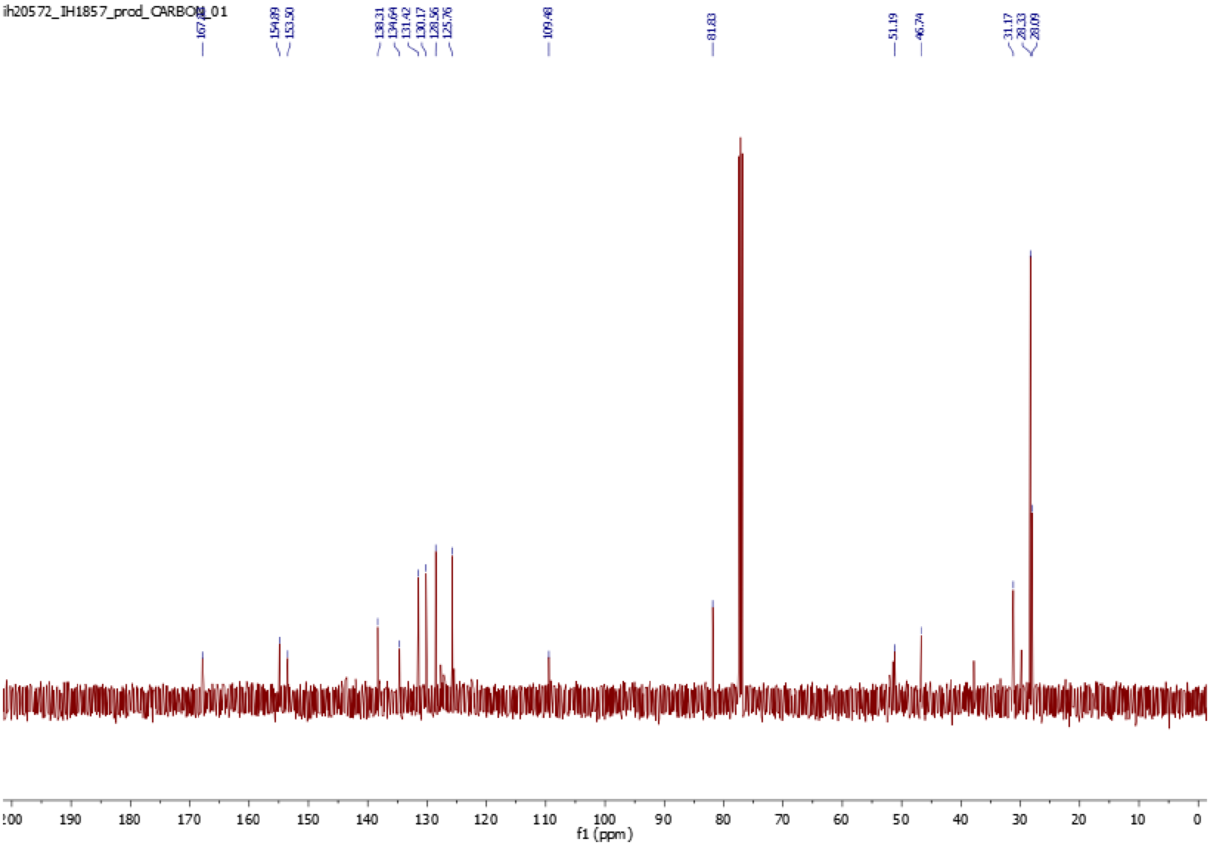
ih20572_IH1857_prod_CARBON_01
154.89
153.50
138.31
134.64
131.42
130.17
128.56
125.76
109.48
81.83
51.19
46.74
31.17
28.33
28.09
f1 (ppm)

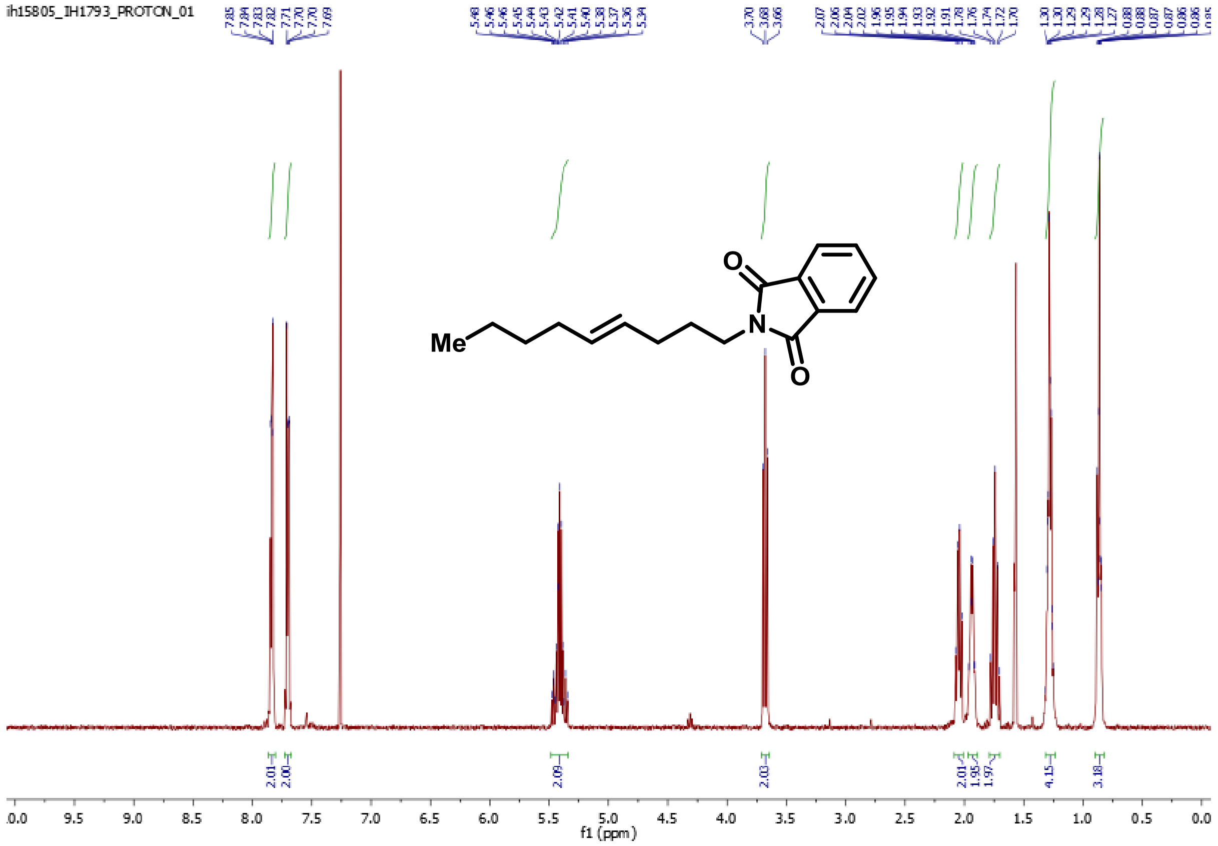
ih15805_IH1793_PROTON_01
Me
N
O
O
f1 (ppm)

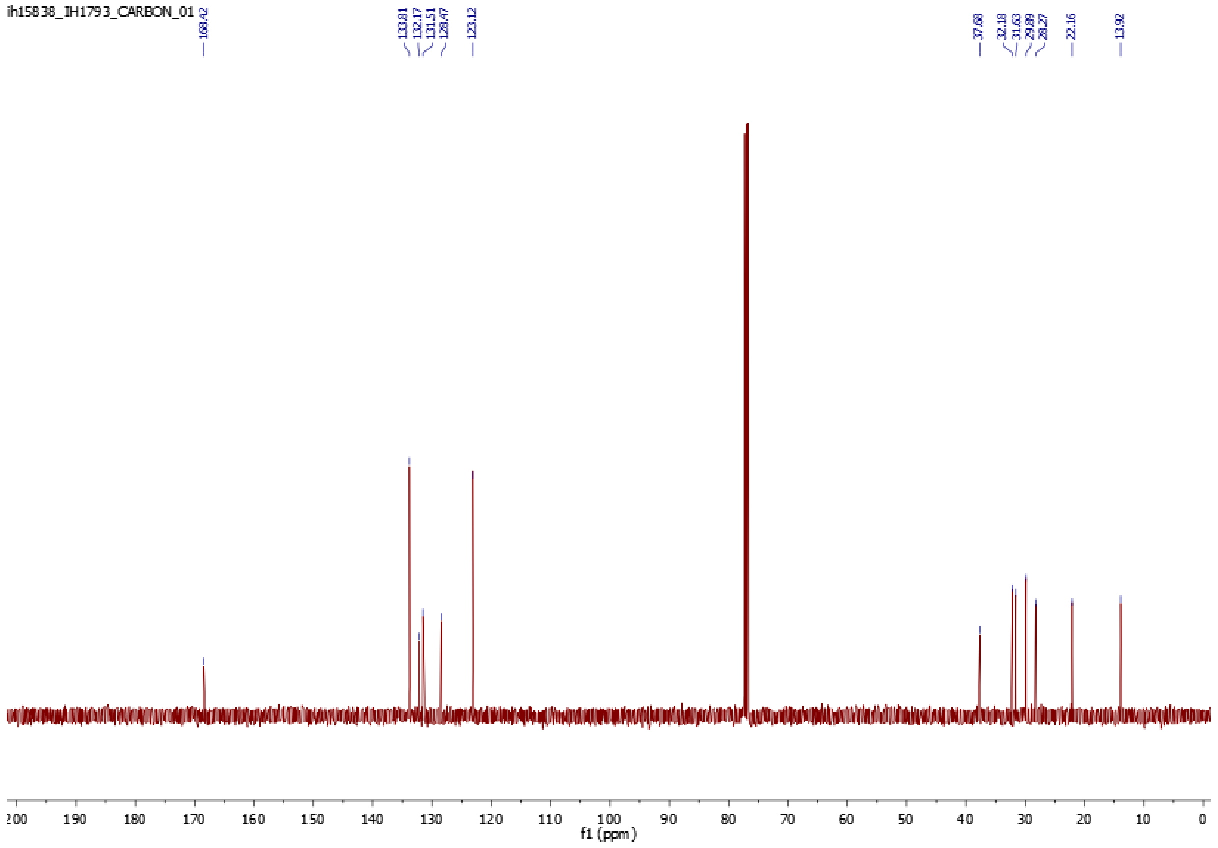
ih15838_IH1793_CARBON_01
168.42
133.81
132.17
131.51
128.47
123.12
37.68
32.18
31.63
29.89
28.27
22.16
13.92
f1 (ppm)

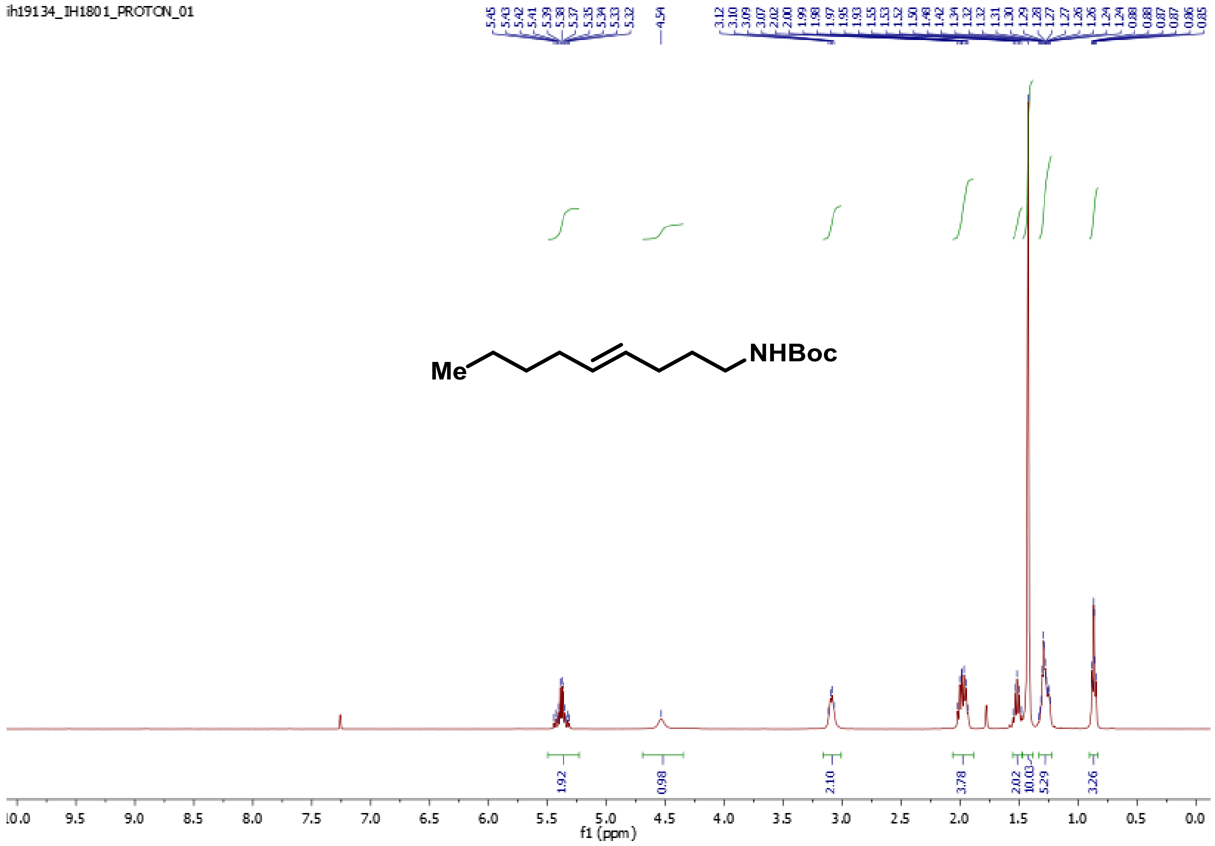

ih19134_IH1801_PROTON_01
Me
NHBoc
1.92
0.98
2.10
3.78
2.02
10.03
5.29
3.26
f1 (ppm)

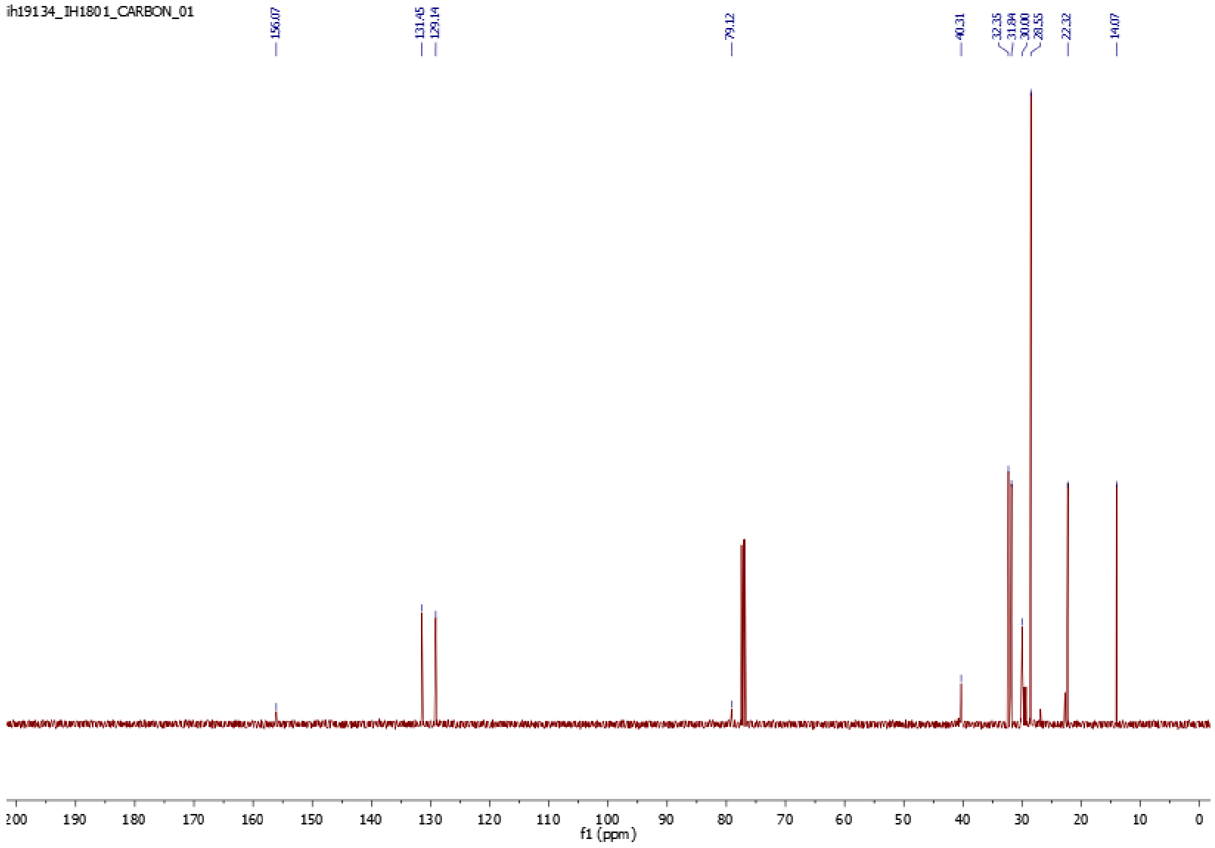

ih19134_IH1801_CARBON_01
156.07
131.45
129.14
79.12
40.31
32.35
31.84
30.00
28.55
22.32
14.07
f1 (ppm)

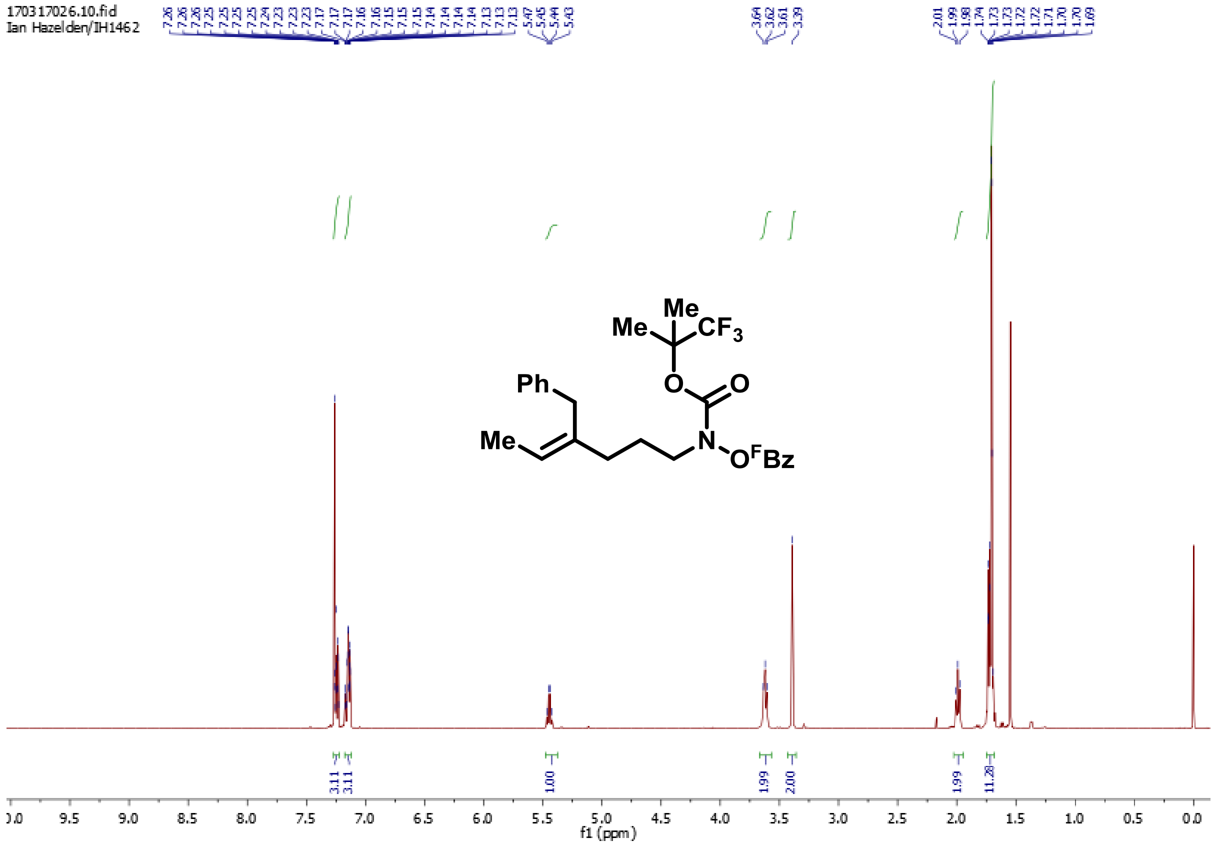
170317026.10.fid
Ian Hazelden/IH1462
7.26 7.26 7.26 7.25 7.25 7.25 7.24 7.23 7.23 7.23 7.17 7.17 7.16 7.16 7.15 7.15 7.14 7.14 7.14 7.13 7.13 7.13 5.47 5.45 5.44 5.43
3.64 3.62 3.61 3.39
2.01 1.99 1.98 1.74 1.73 1.73 1.72 1.72 1.71 1.70 1.70 1.69
Me
Me
CF3
O
O
Ph
Me
N
OFBz
3.11 3.11 1.00 1.99 2.00 1.99 11.28
f1 (ppm)

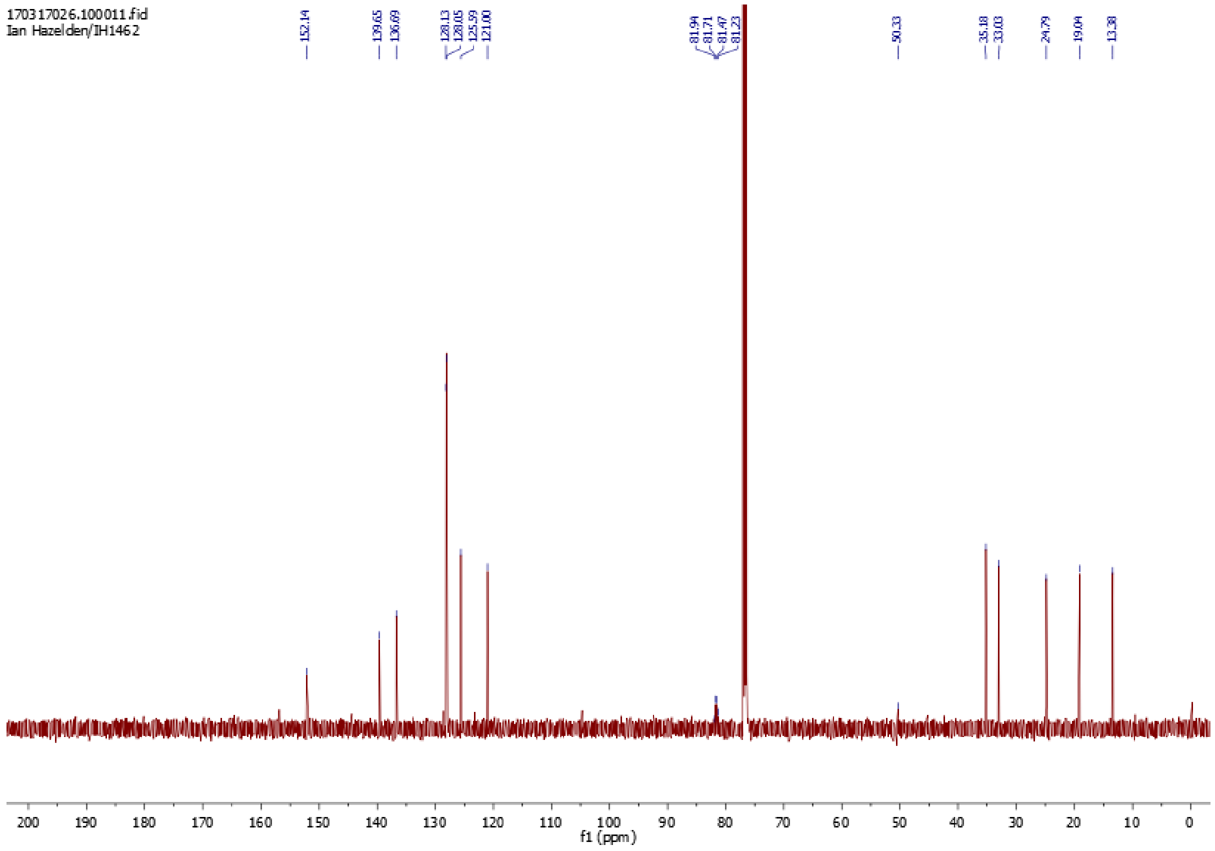
170317026.100011.fid
Ian Hazelden/IH1462
152.14
139.65 136.69
128.13 128.05 125.99 121.00
81.94 81.71 81.47 81.23
50.33
35.18 33.03
24.79
19.04
13.38
f1 (ppm)

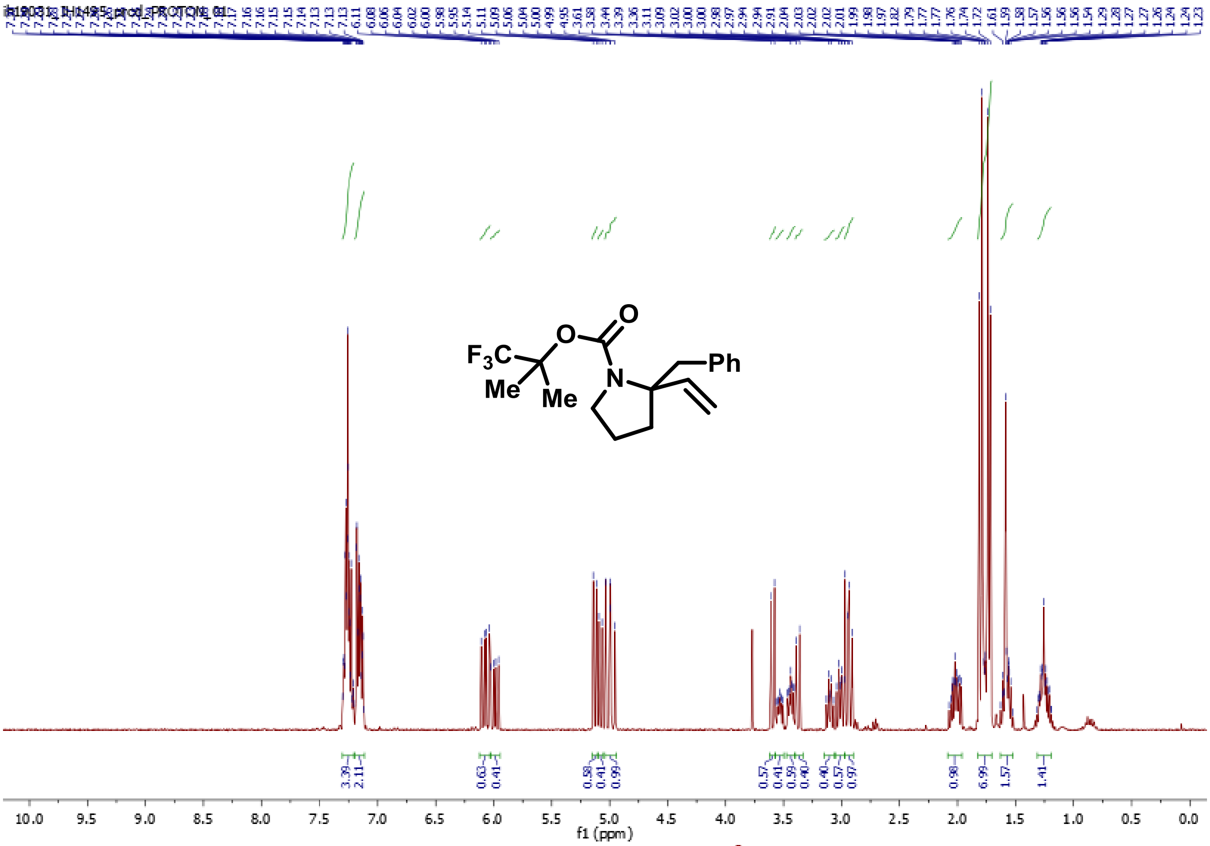
F3C
Me
Me
O
O
N
Ph
f1 (ppm)

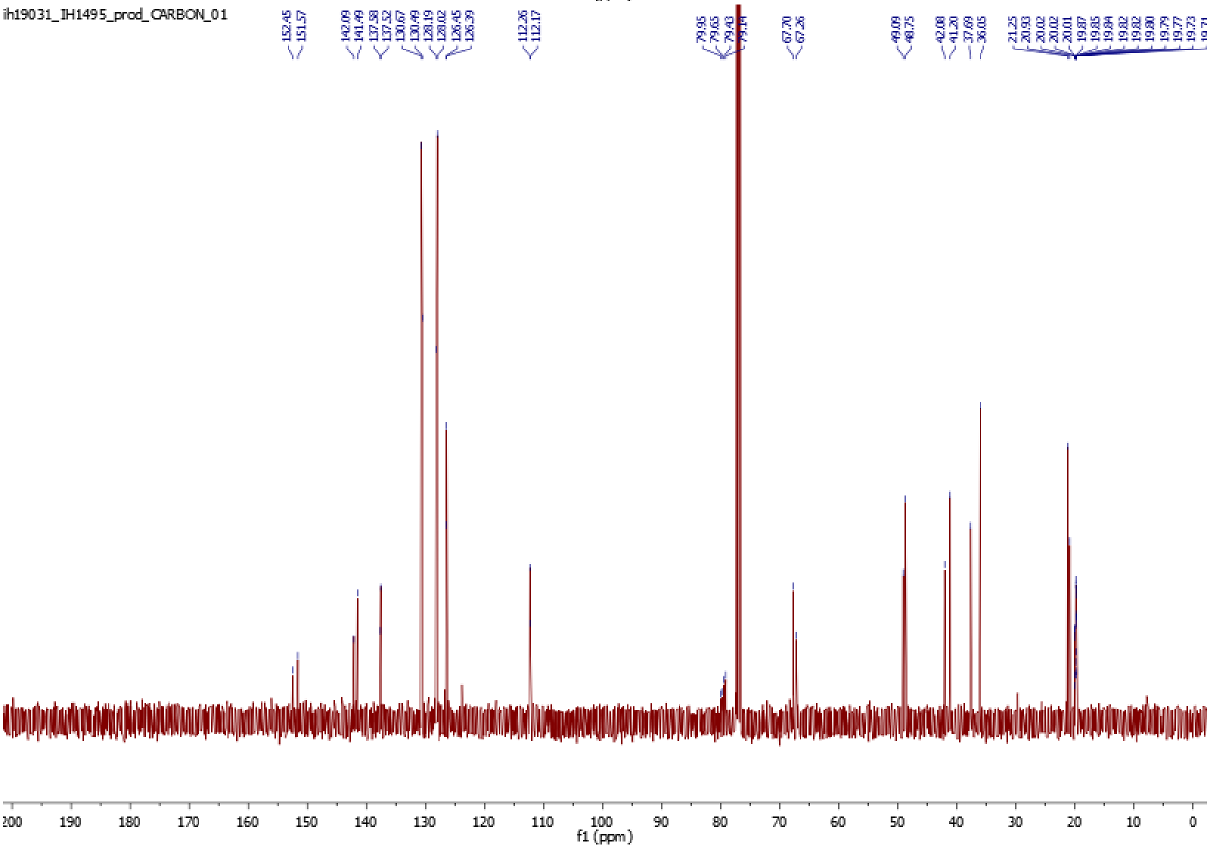
ih19031_IH1495_prod_CARBON_01
f1 (ppm)

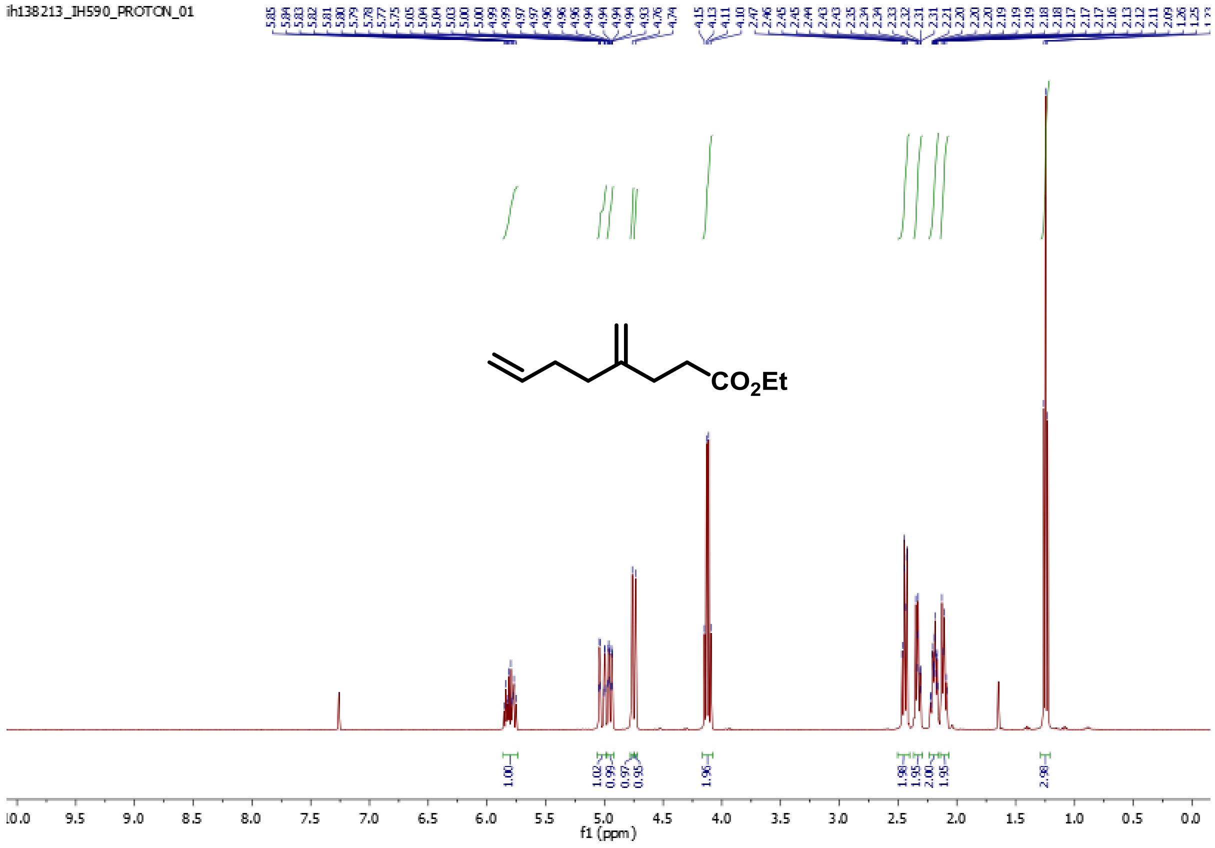
ih138213_IH590_PROTON_01
CO_2Et
f1 (ppm)

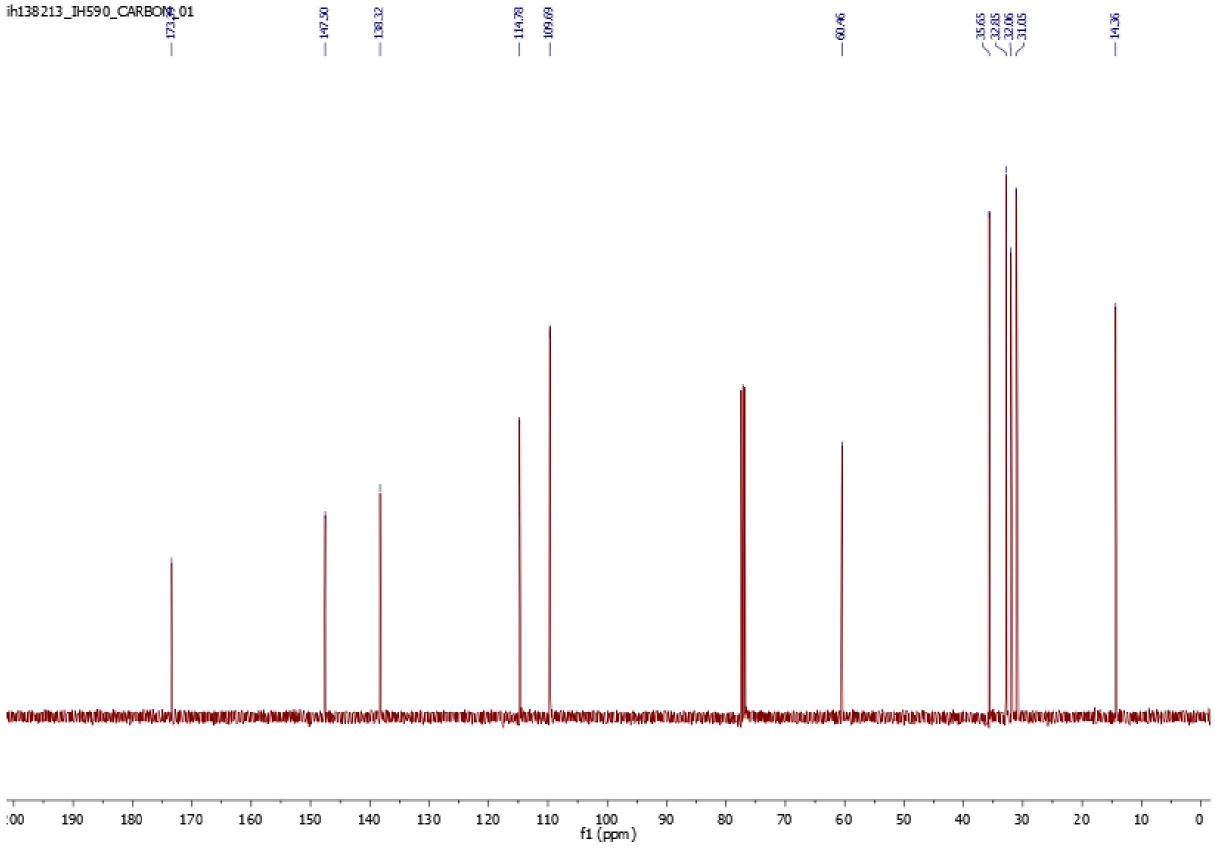
ih138213_IH590_CARBON_01
147.50
138.32
114.78
109.69
60.46
35.65
32.85
32.06
31.05
14.36
f1 (ppm)

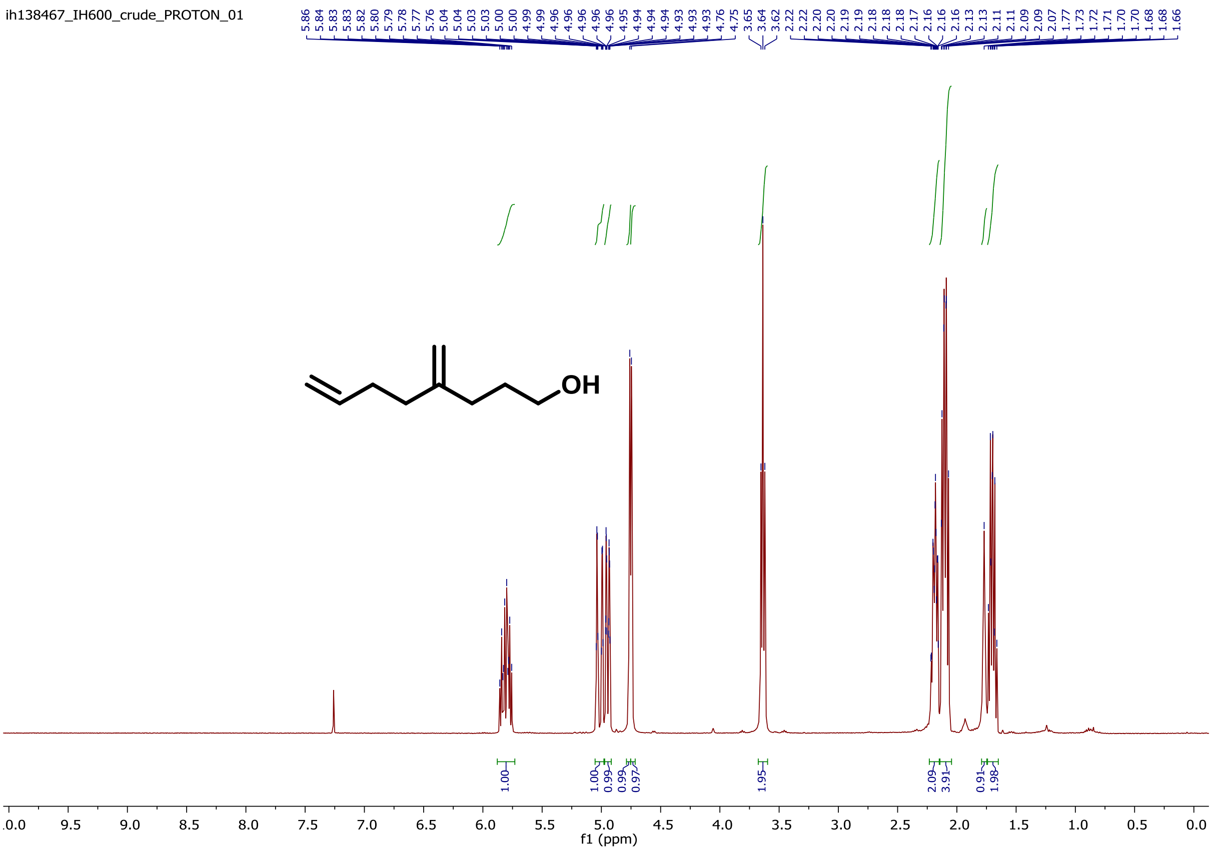
ih138467_IH600_crude_PROTON_01
5.86 5.84 5.83 5.83 5.82 5.80 5.79 5.78 5.77 5.76 5.04 5.04 5.03 5.03 5.00 5.00 4.99 4.99 4.96 4.96 4.96 4.96 4.96 4.95 4.94 4.94 4.94 4.93 4.93 4.93 4.76 4.75 3.65 3.64 3.62 2.22 2.22 2.20 2.19 2.19 2.18 2.18 2.18 2.17 2.16 2.16 2.16 2.13 2.13 2.11 2.11 2.09 2.09 2.07 1.77 1.73 1.72 1.71 1.70 1.70 1.68 1.66
OH
1.00 1.00 0.99 0.99 0.97 1.95 2.09 3.91 0.91 1.98
f1 (ppm)

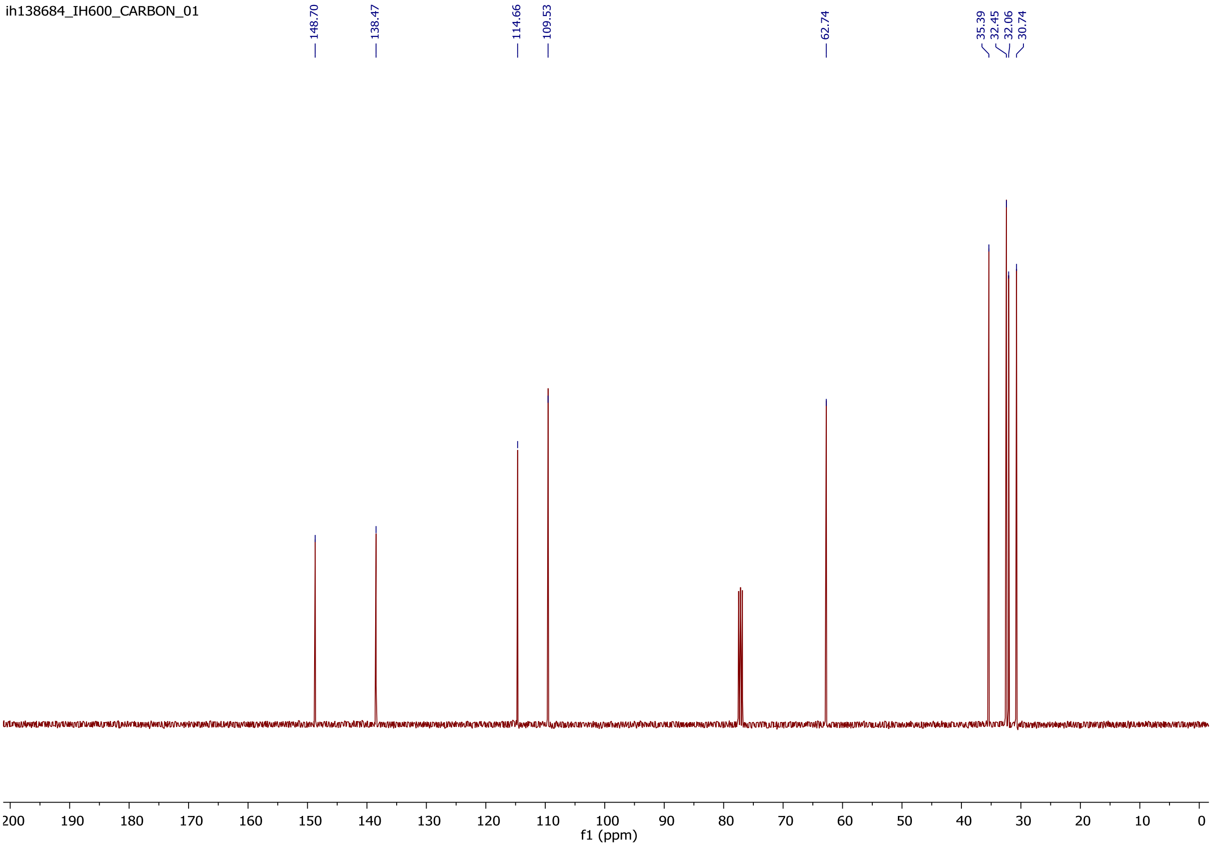
ih138684_IH600_CARBON_01
148.70
138.47
114.66
109.53
62.74
35.39 32.45 32.06 30.74
f1 (ppm)

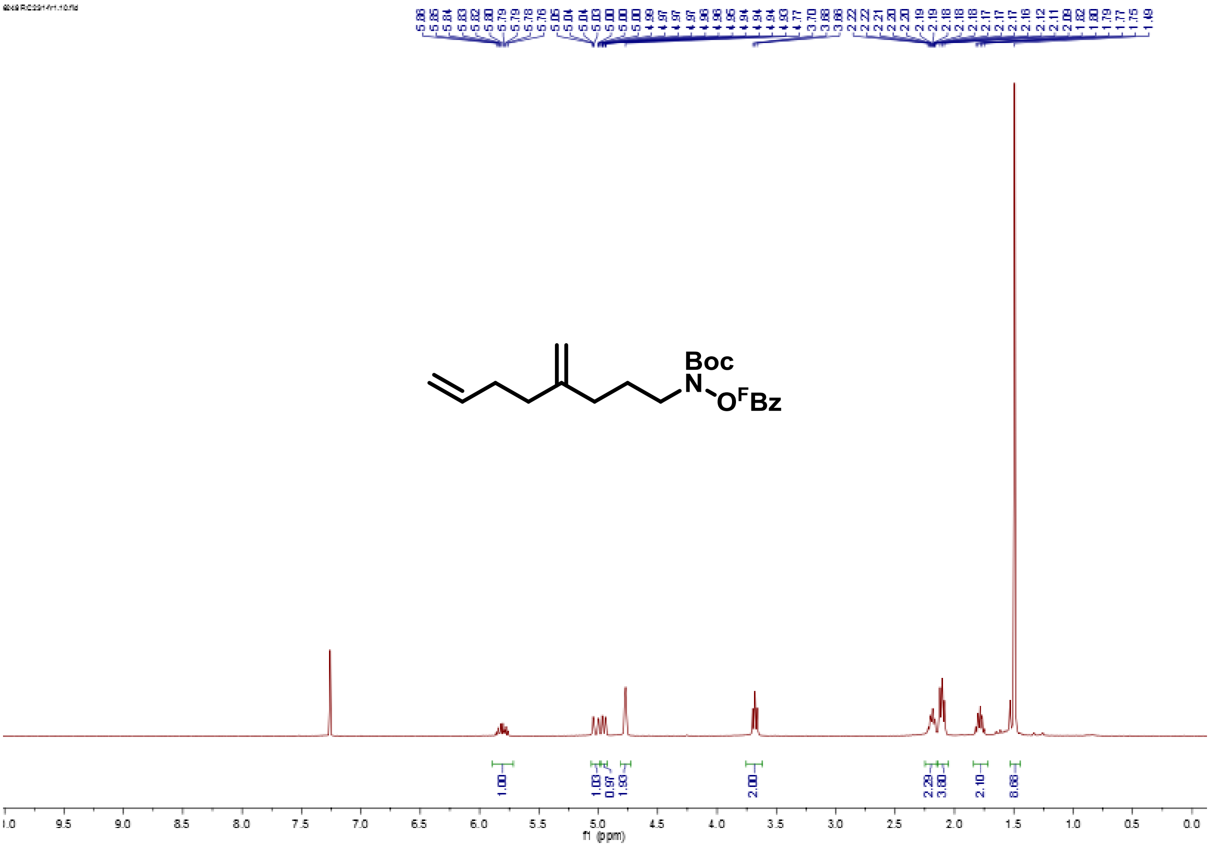
Boc
N
O^F Bz
1.00
1.03
0.97
1.93
2.00
2.29
3.80
2.10
8.68
f1 (ppm)

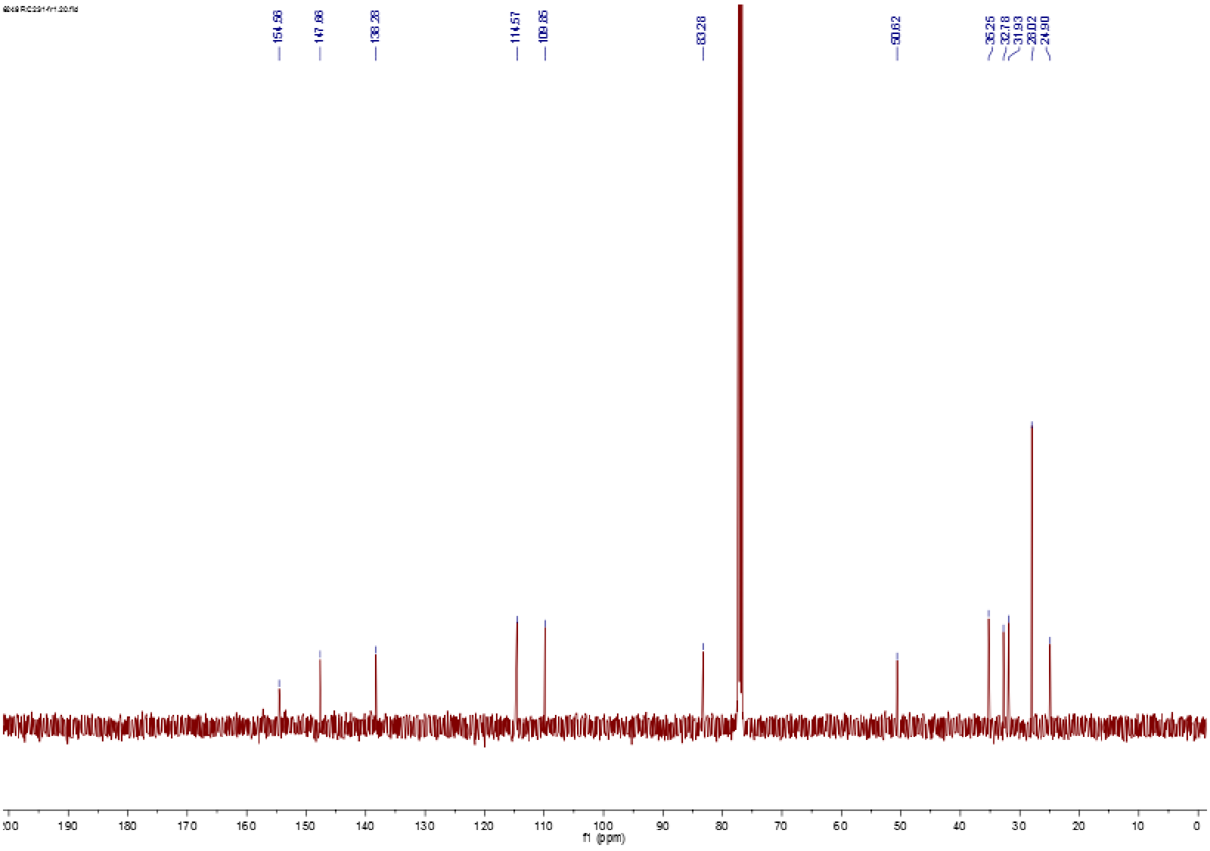
154.56
147.66
138.28
114.57
109.85
83.28
50.62
35.25
32.78
31.93
28.02
24.90
f1 (ppm)

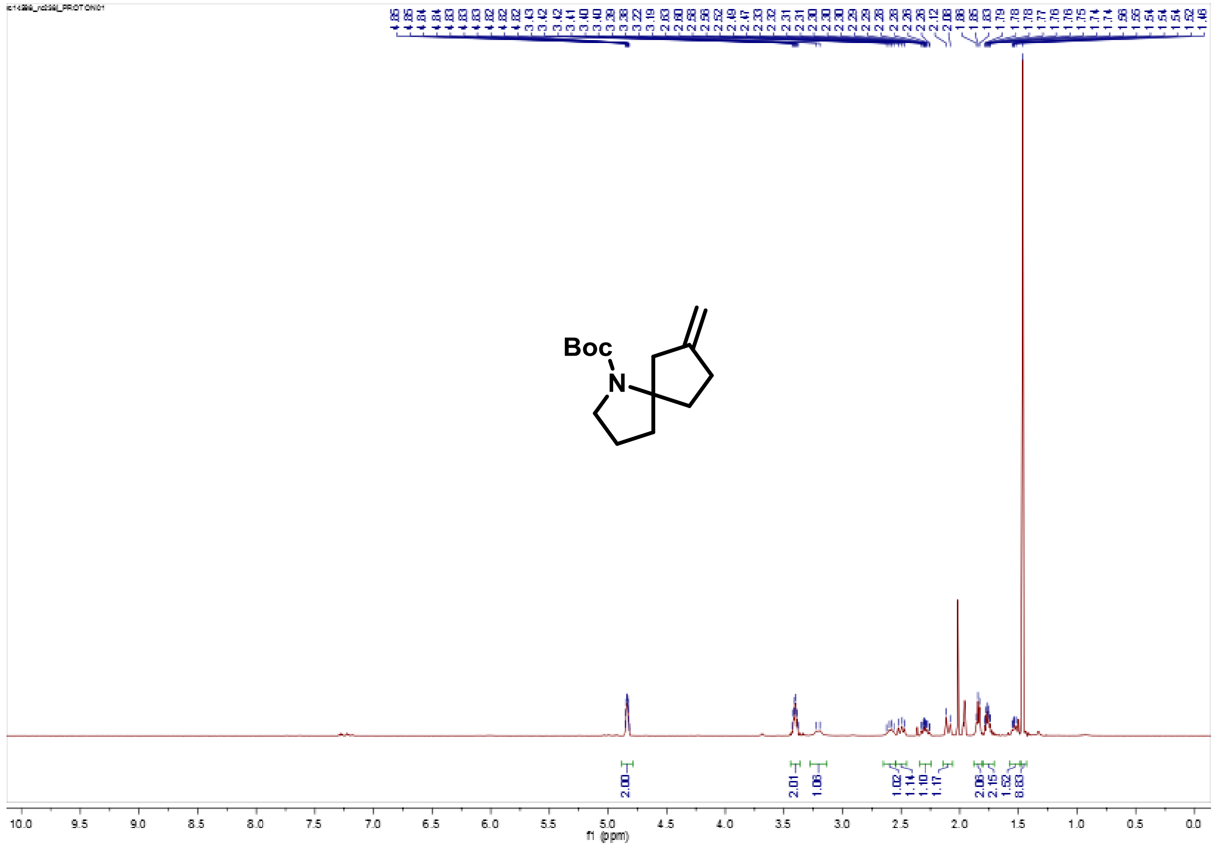
Boc
N
f1 (ppm)

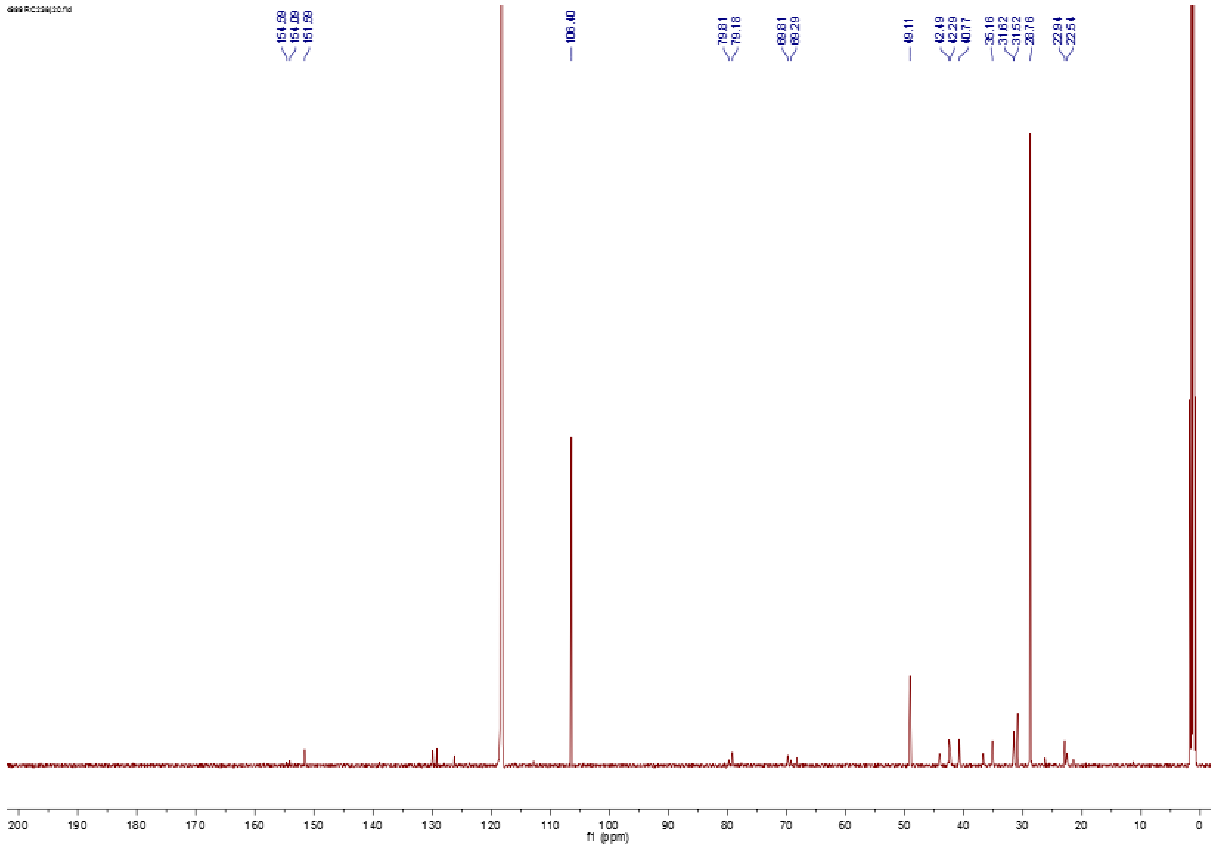
f1 (ppm)

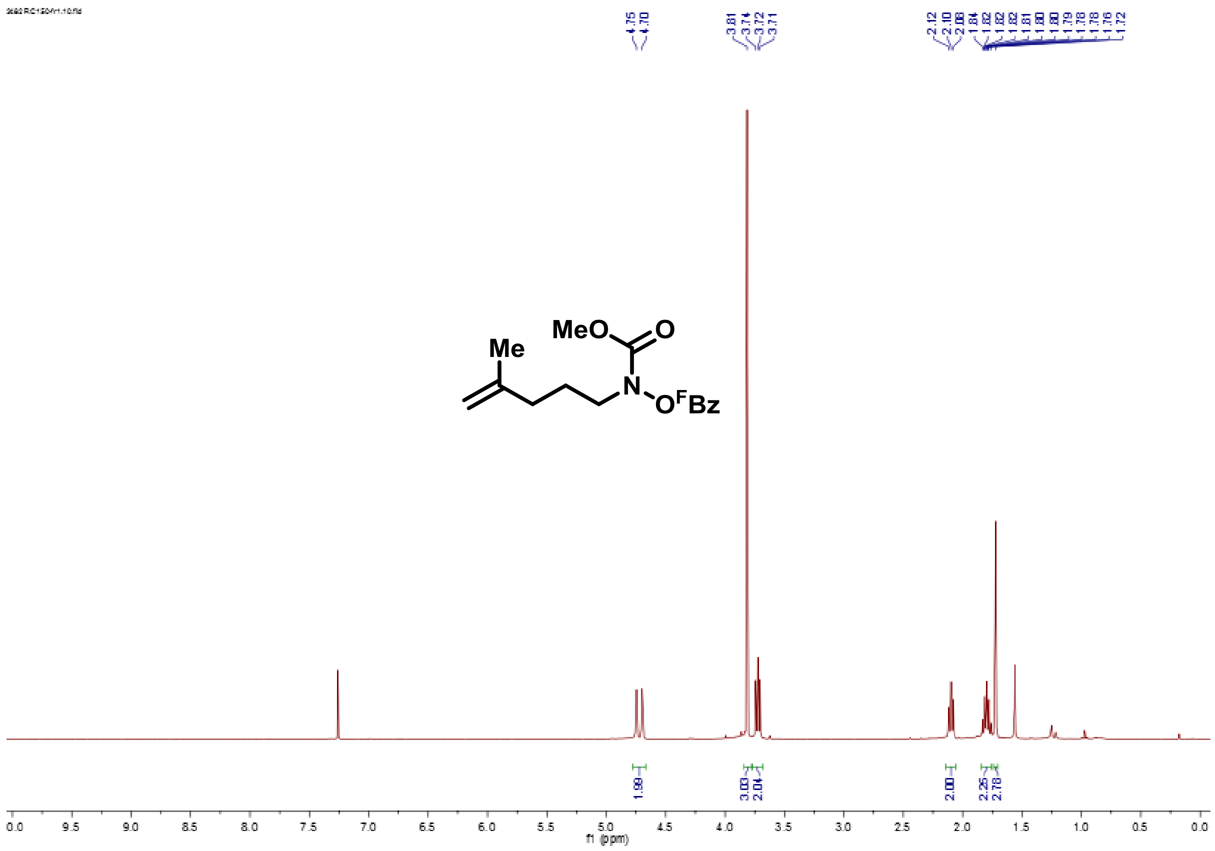
4.75
4.70
3.81
3.74
3.72
3.71
2.12
2.10
2.08
1.84
1.82
1.82
1.81
1.80
1.80
1.79
1.78
1.78
1.76
1.72
MeO
O
Me
N
O
F
Bz
1.99
3.03
2.04
2.00
2.25
2.78
f1 (ppm)

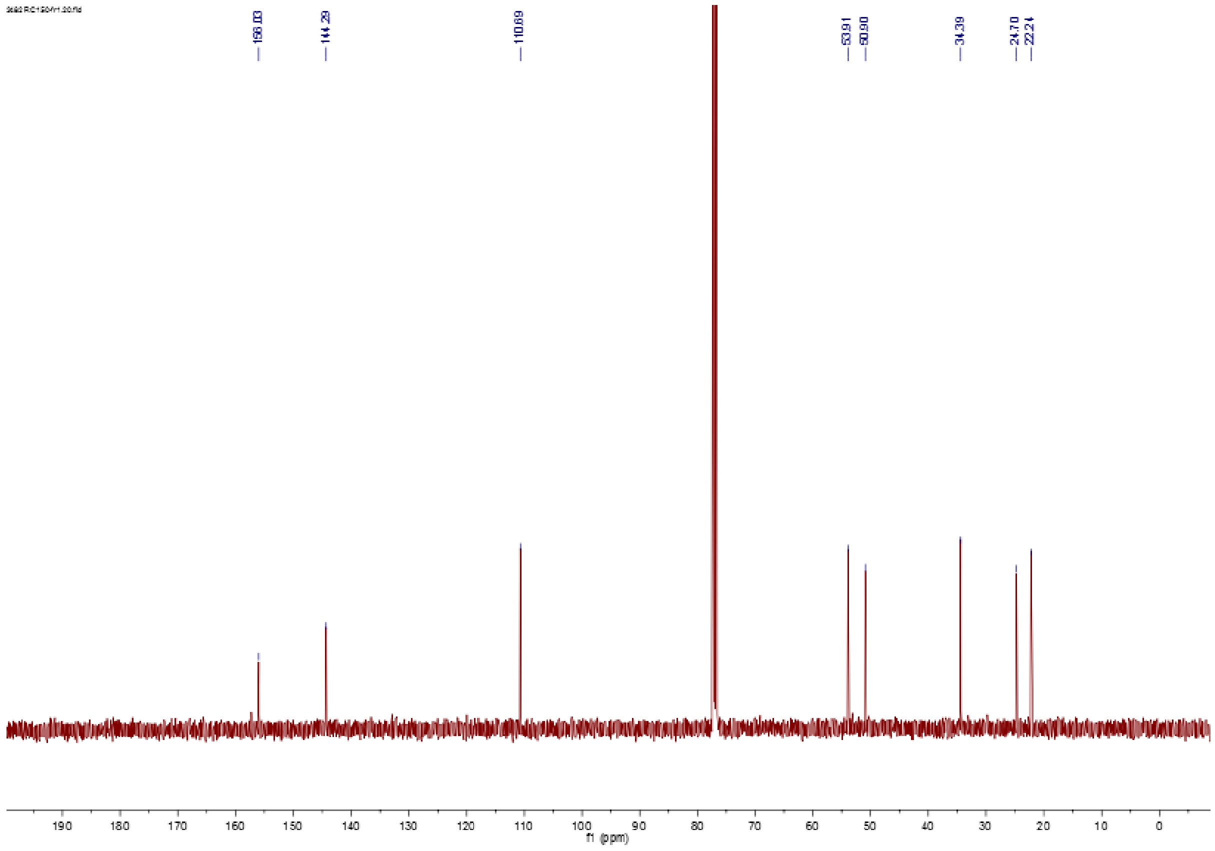
156.03
144.29
110.69
53.91
50.90
34.39
24.70
22.24
f1 (ppm)

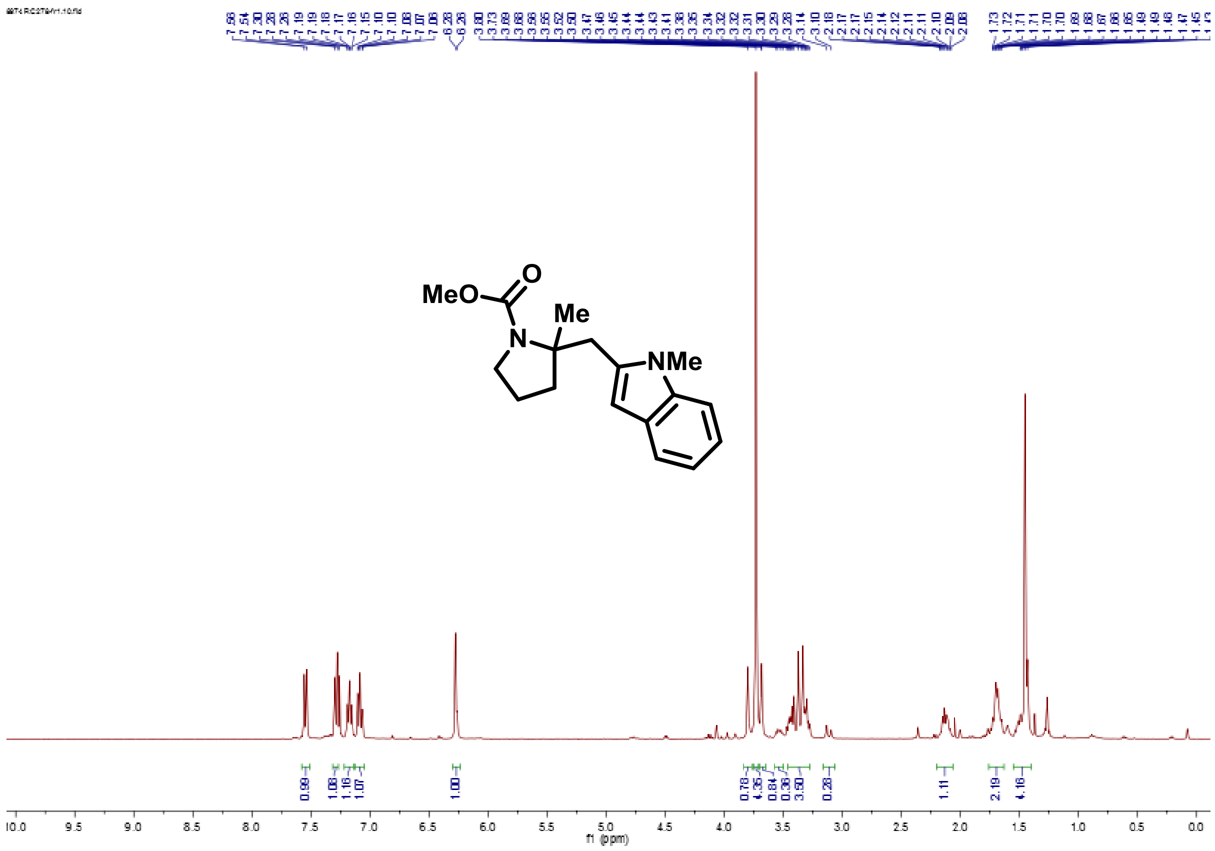
MeO
O
Me
N
NMe
0.99
1.08
1.16
1.07
1.00
0.78
4.35
0.84
0.36
3.50
0.28
1.11
2.19
4.16
f1 (ppm)

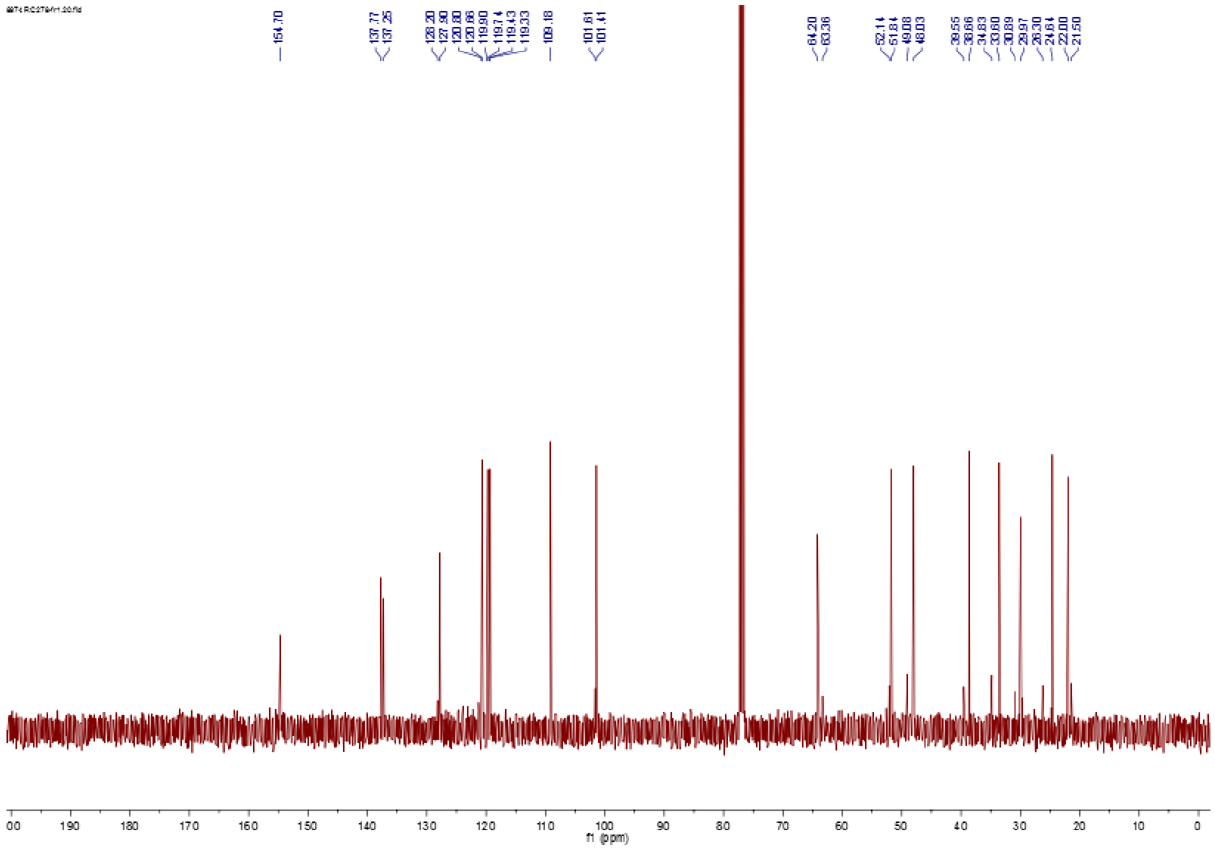
154.70
137.77
137.25
128.20
127.90
120.80
120.66
119.90
119.74
119.43
119.33
109.18
101.61
101.41
64.20
63.36
52.14
51.84
49.08
48.03
39.55
38.66
34.83
33.60
30.89
29.97
26.30
24.64
22.00
21.50
f1 (ppm)

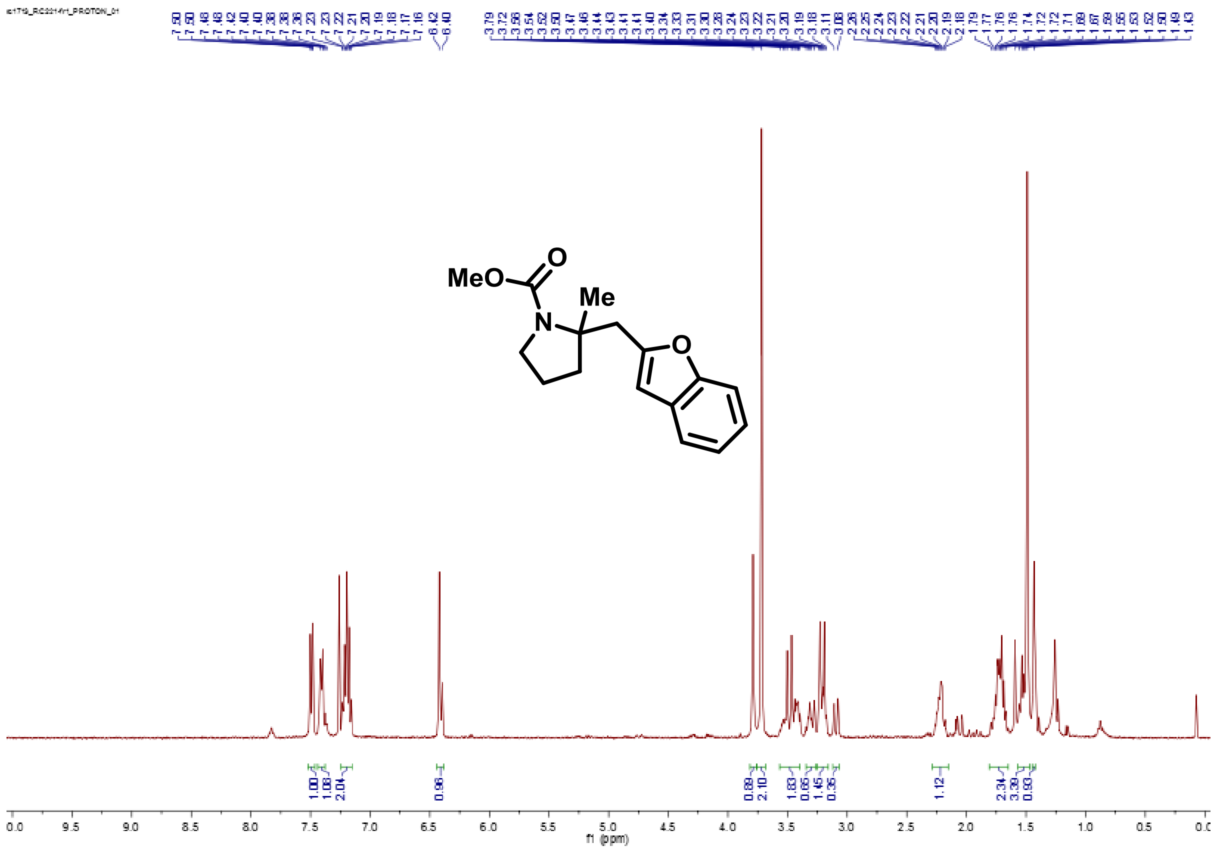

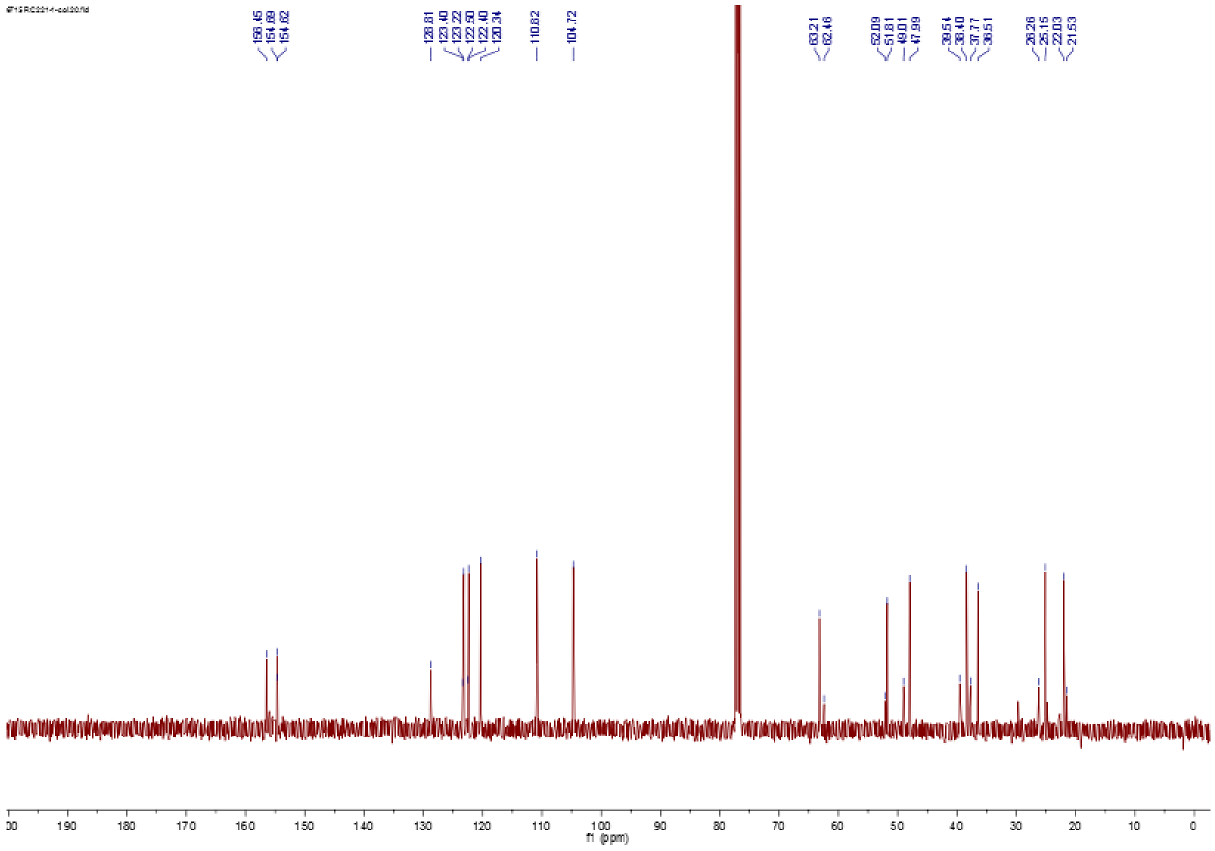

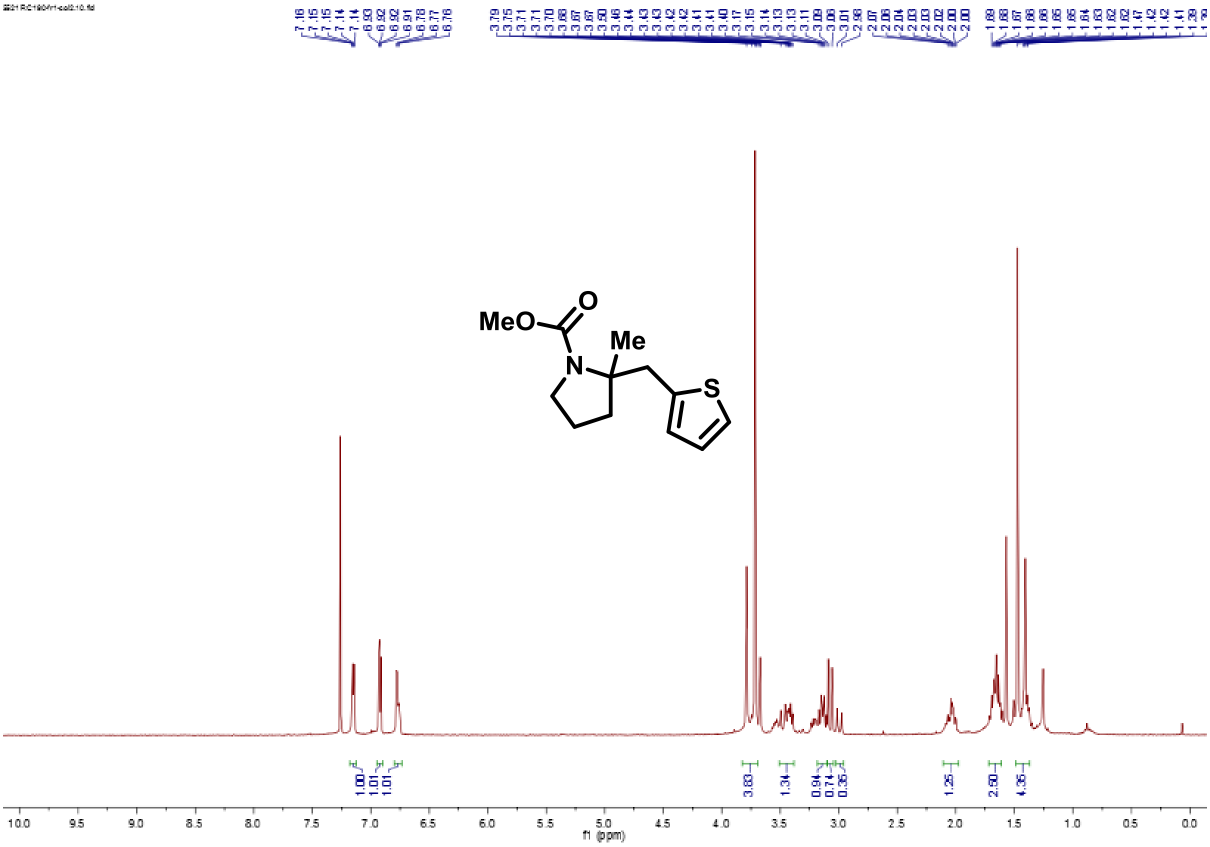
MeO
O
N
Me
S
7.16
7.15
7.15
7.14
6.93
6.92
6.92
6.91
6.78
6.77
6.76
1.00
1.01
1.01
3.83
1.34
0.94
0.74
0.35
1.25
2.50
4.35
f1 (ppm)

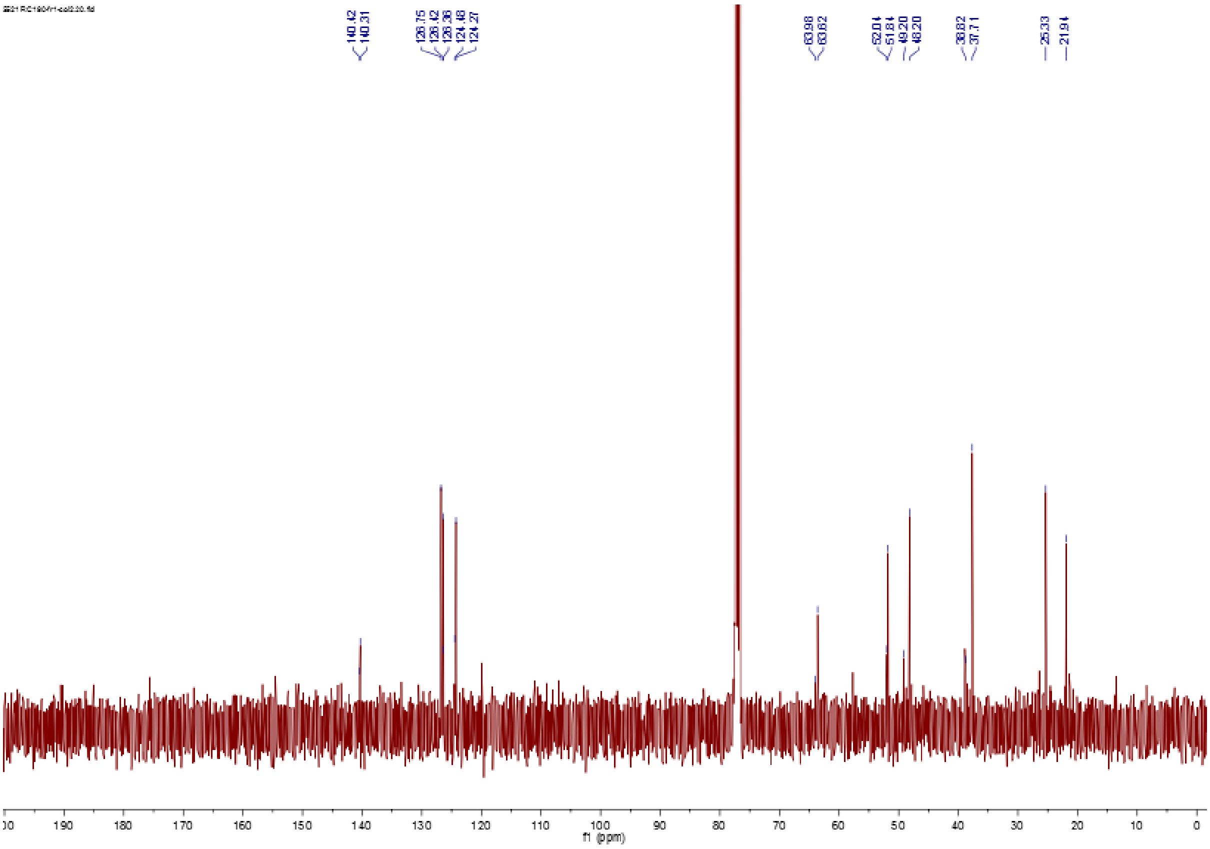
140.42
140.31
126.75
126.42
126.36
124.48
124.27
63.98
63.62
52.04
51.84
49.20
48.20
38.82
37.71
25.33
21.94
f1 (ppm)

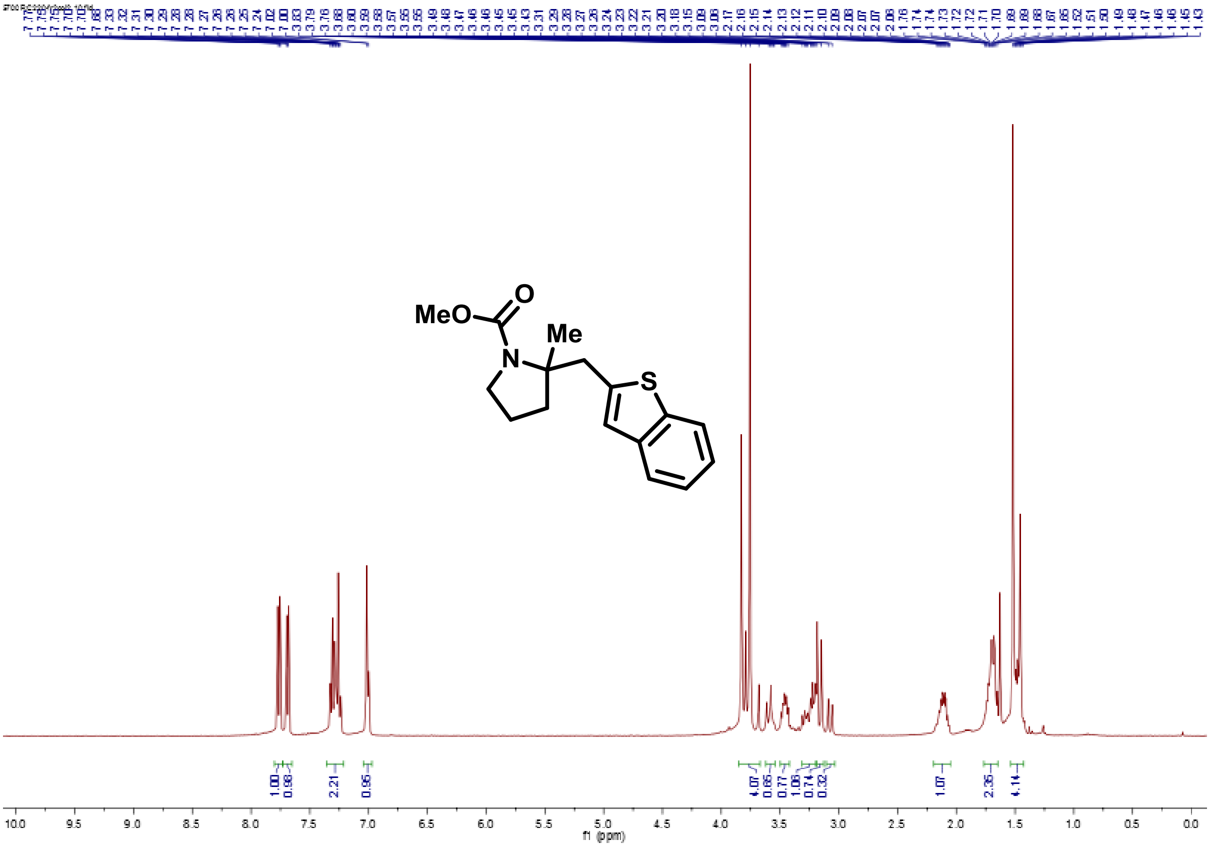

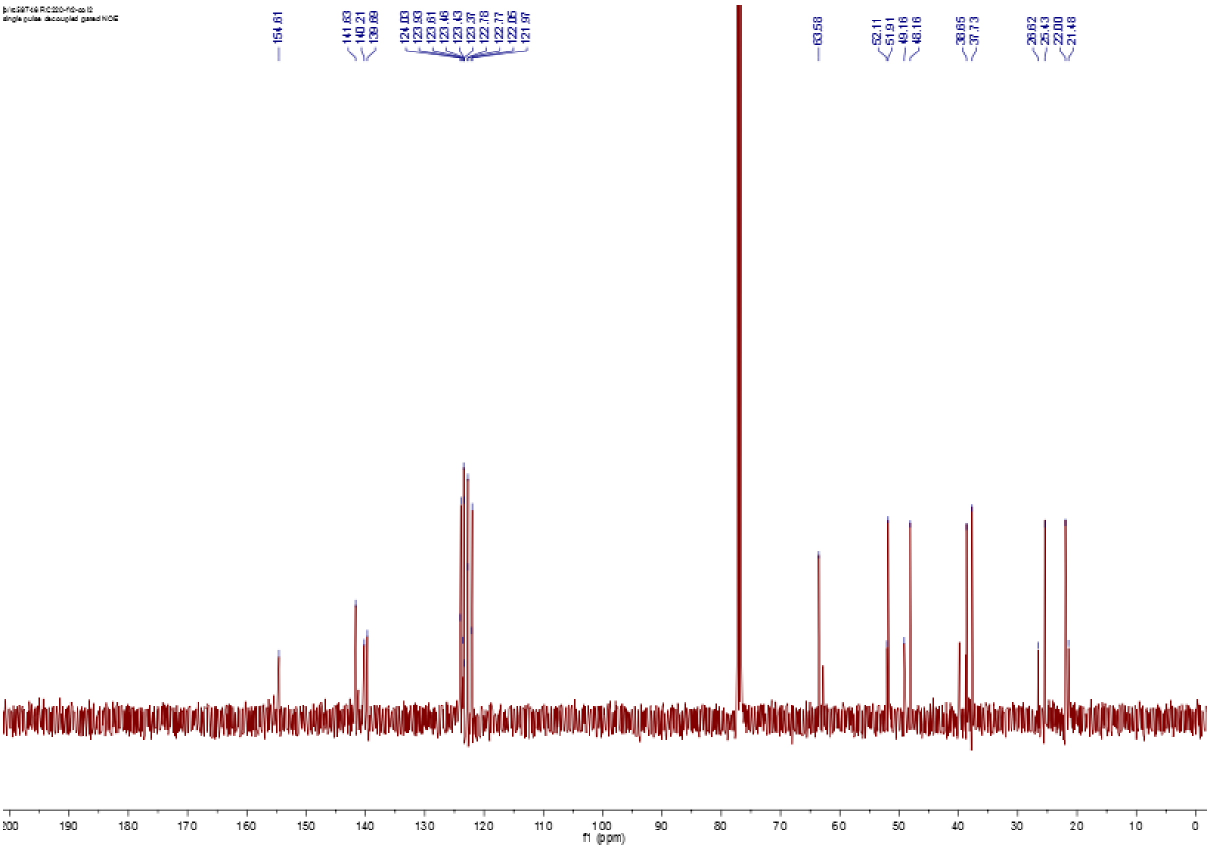

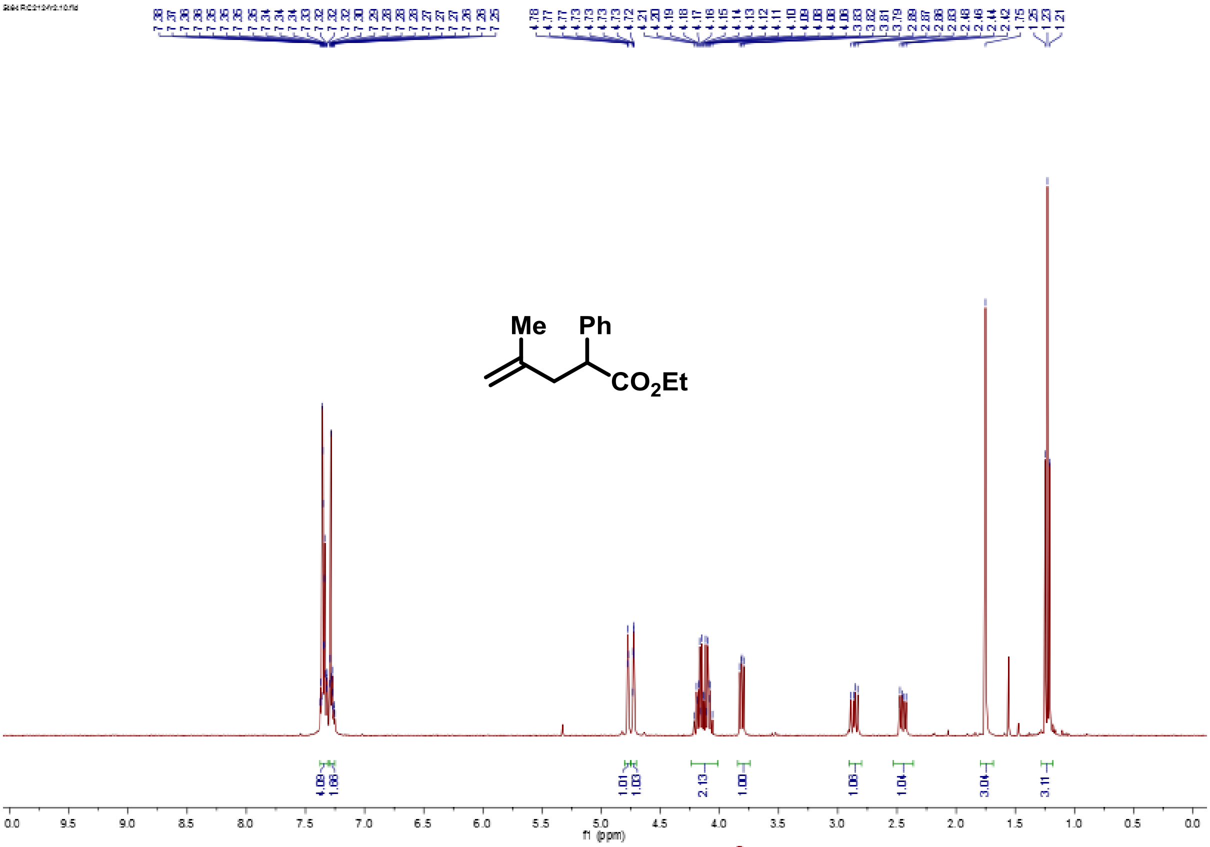
Me
Ph
CO2Et
f1 (ppm)

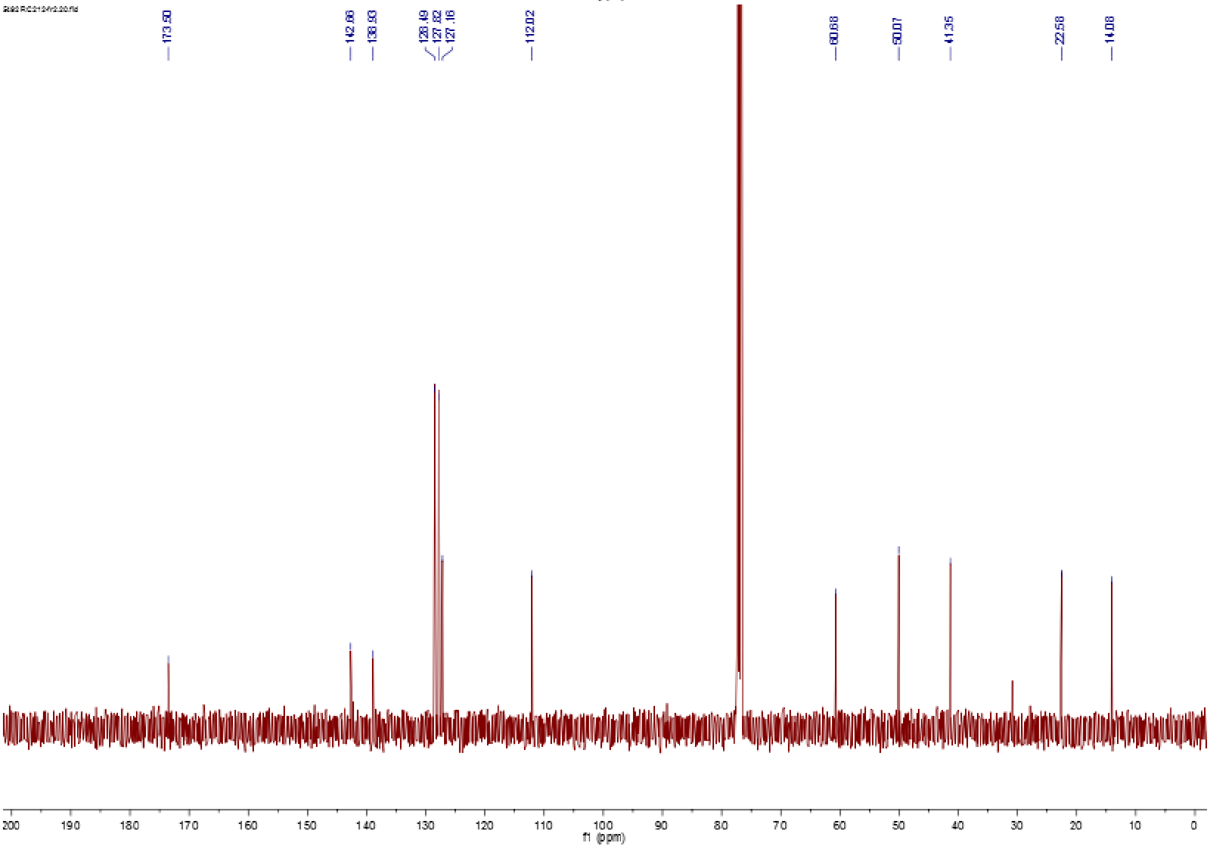
f1 (ppm)

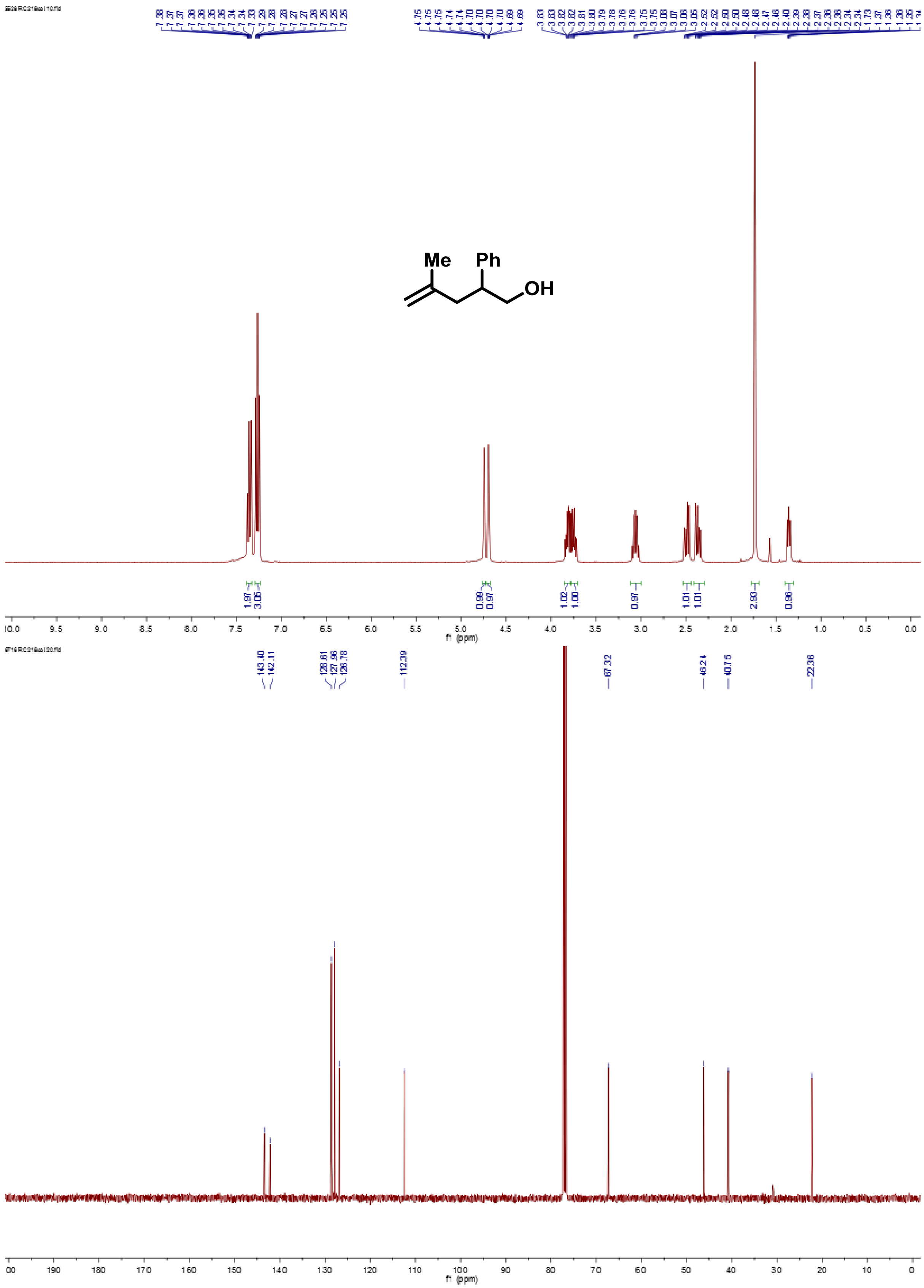

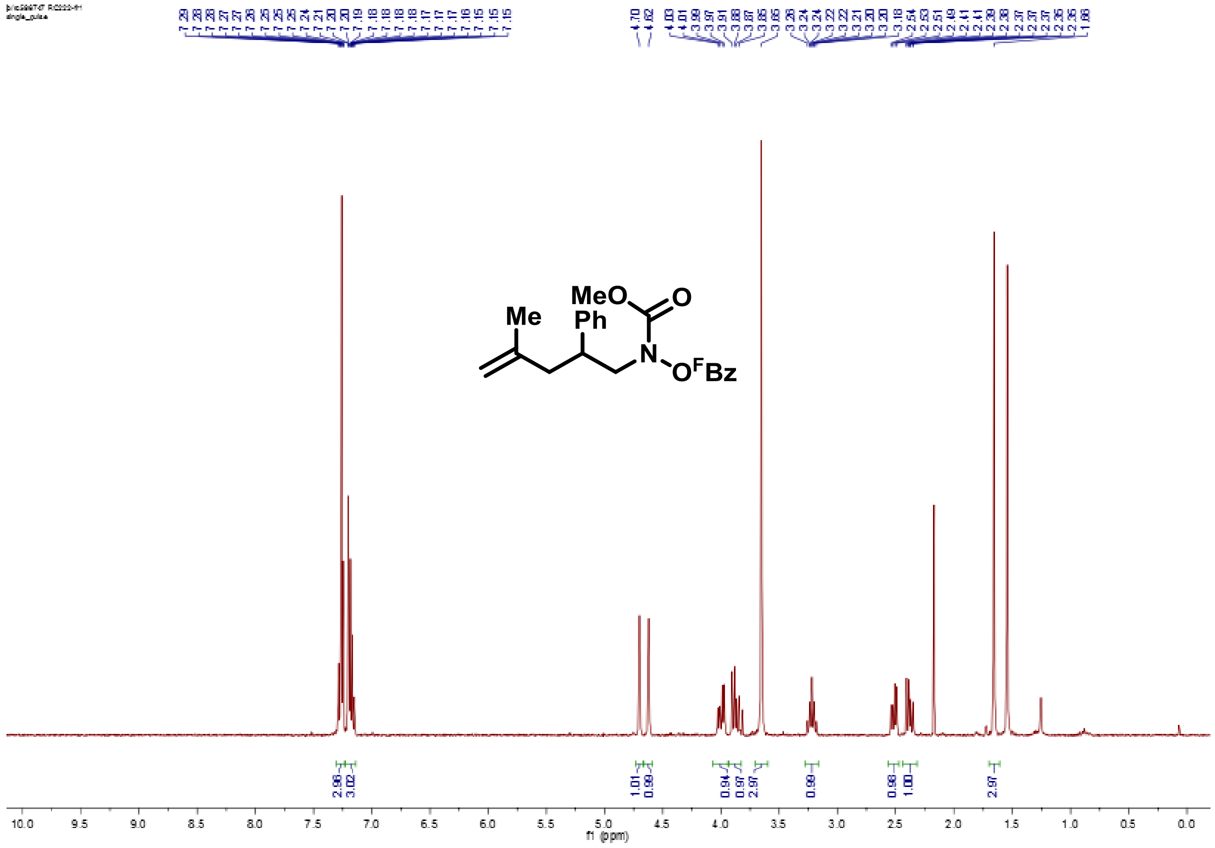
MeO
O
Me
Ph
N
O
F
Bz
f1 (ppm)

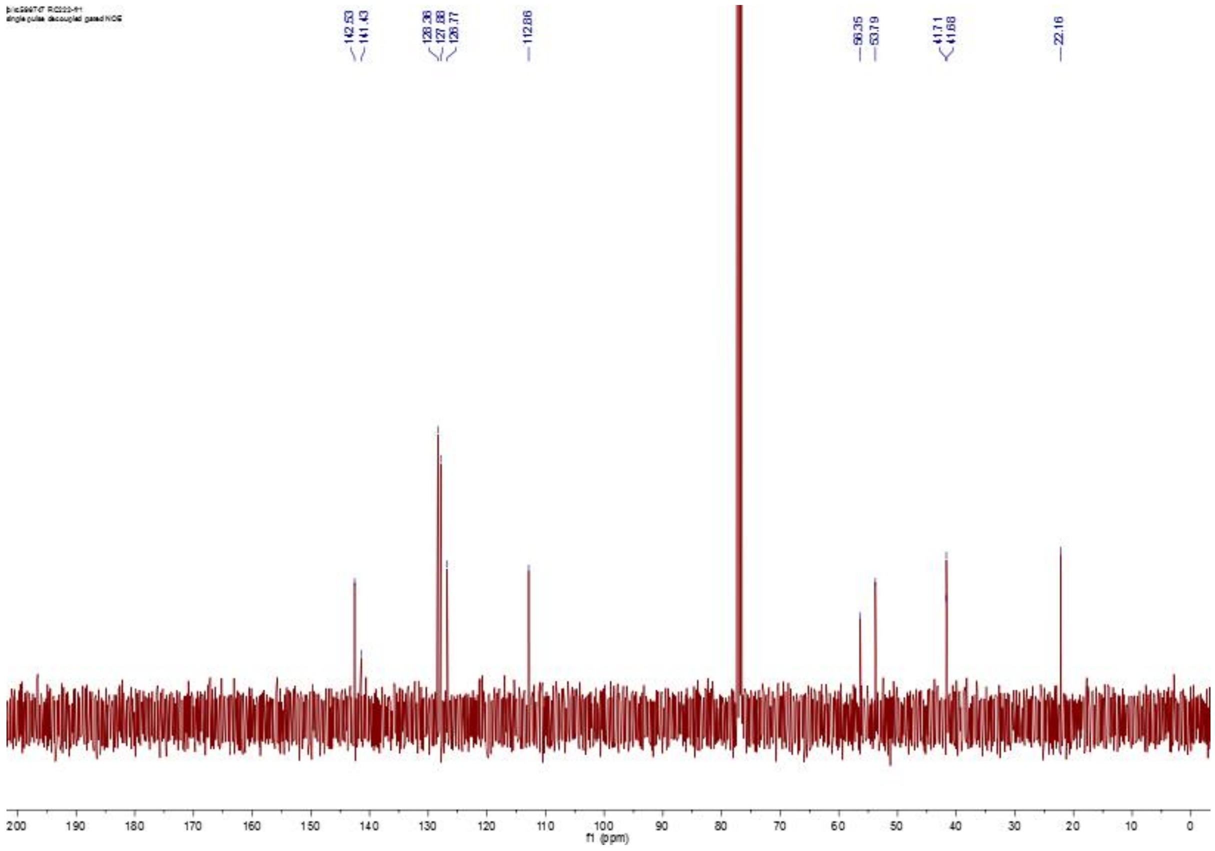
f1 (ppm)

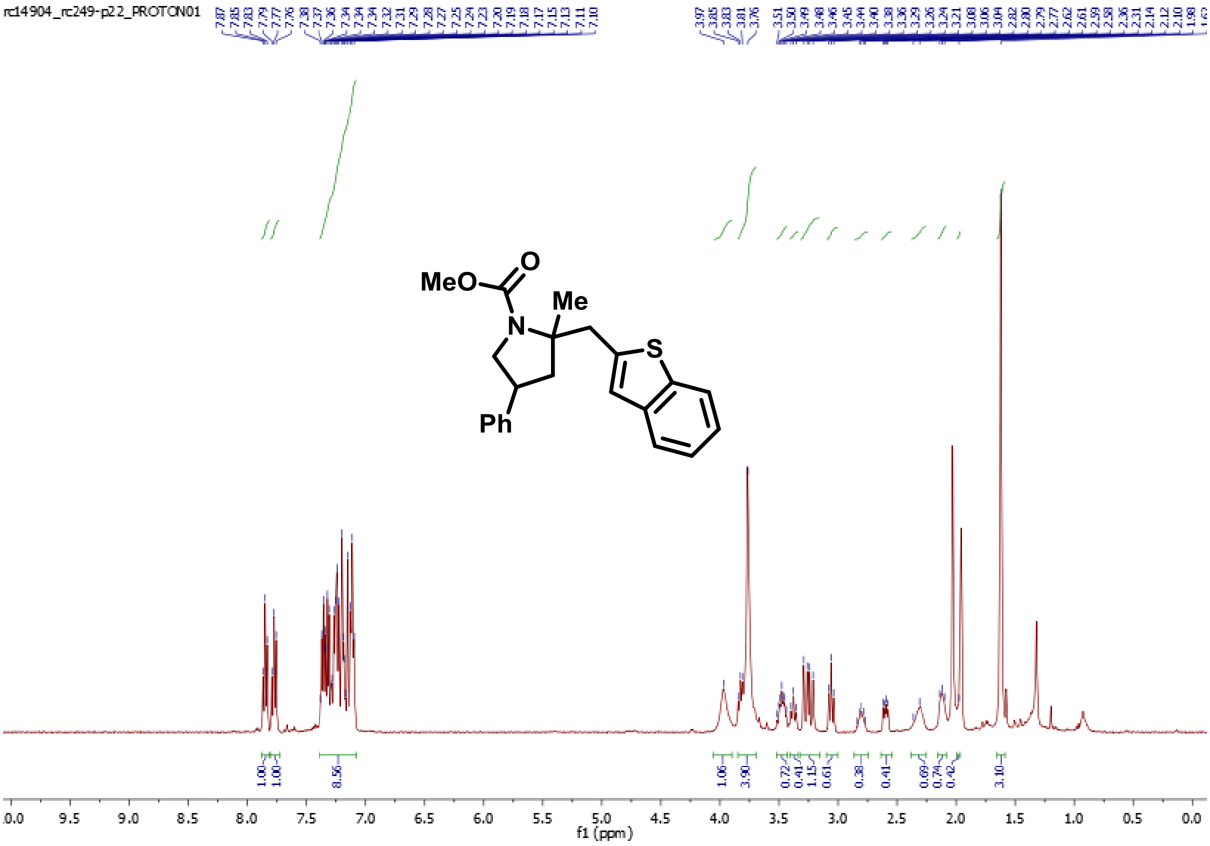
rc14904_rc249-p22_PROTON01
MeO
O
N
Me
S
Ph
f1 (ppm)

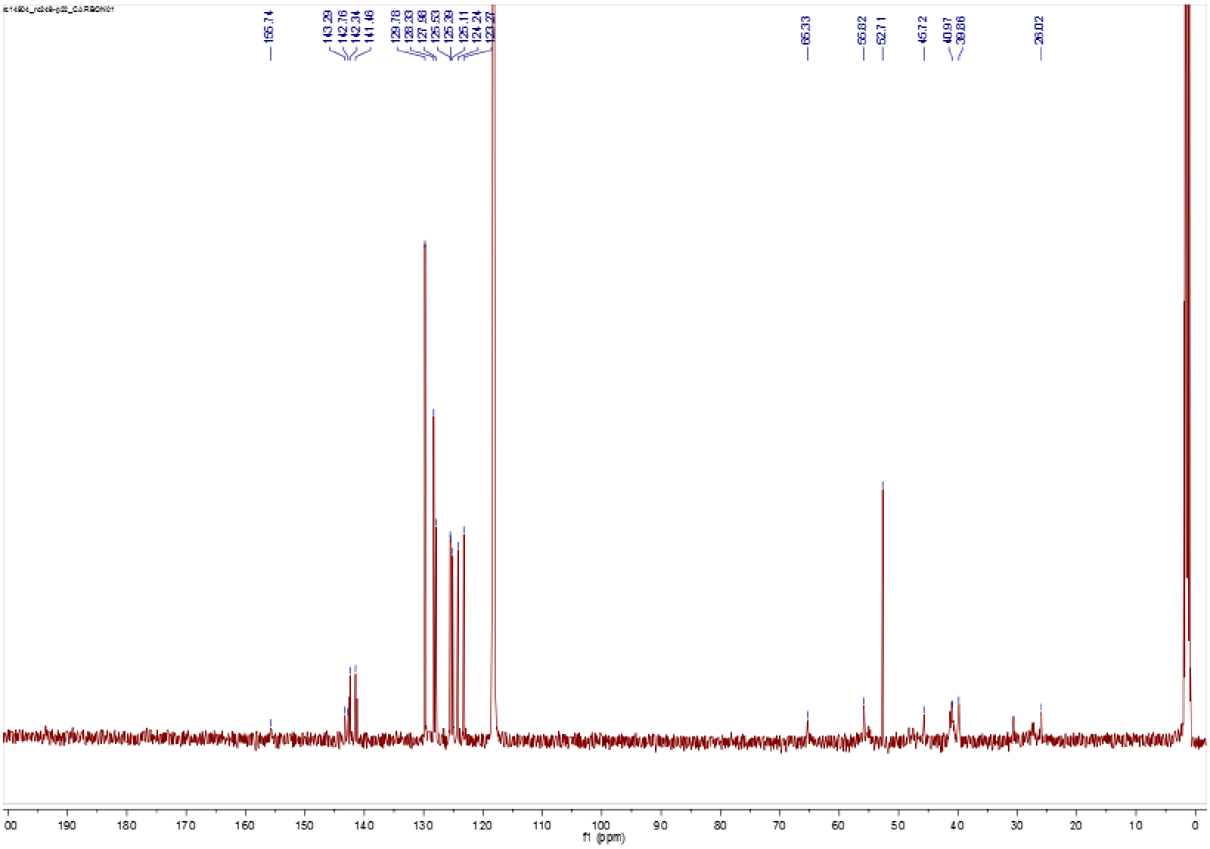
f1 (ppm)

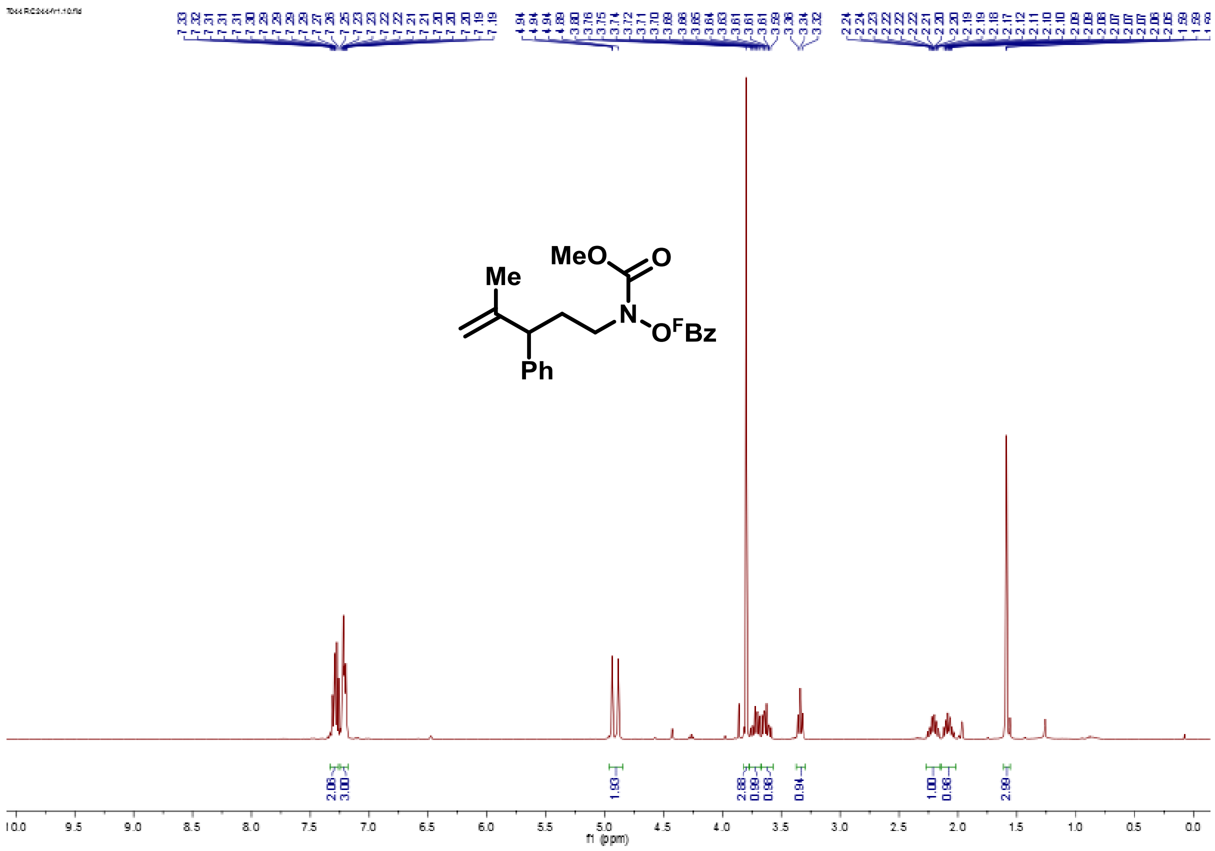

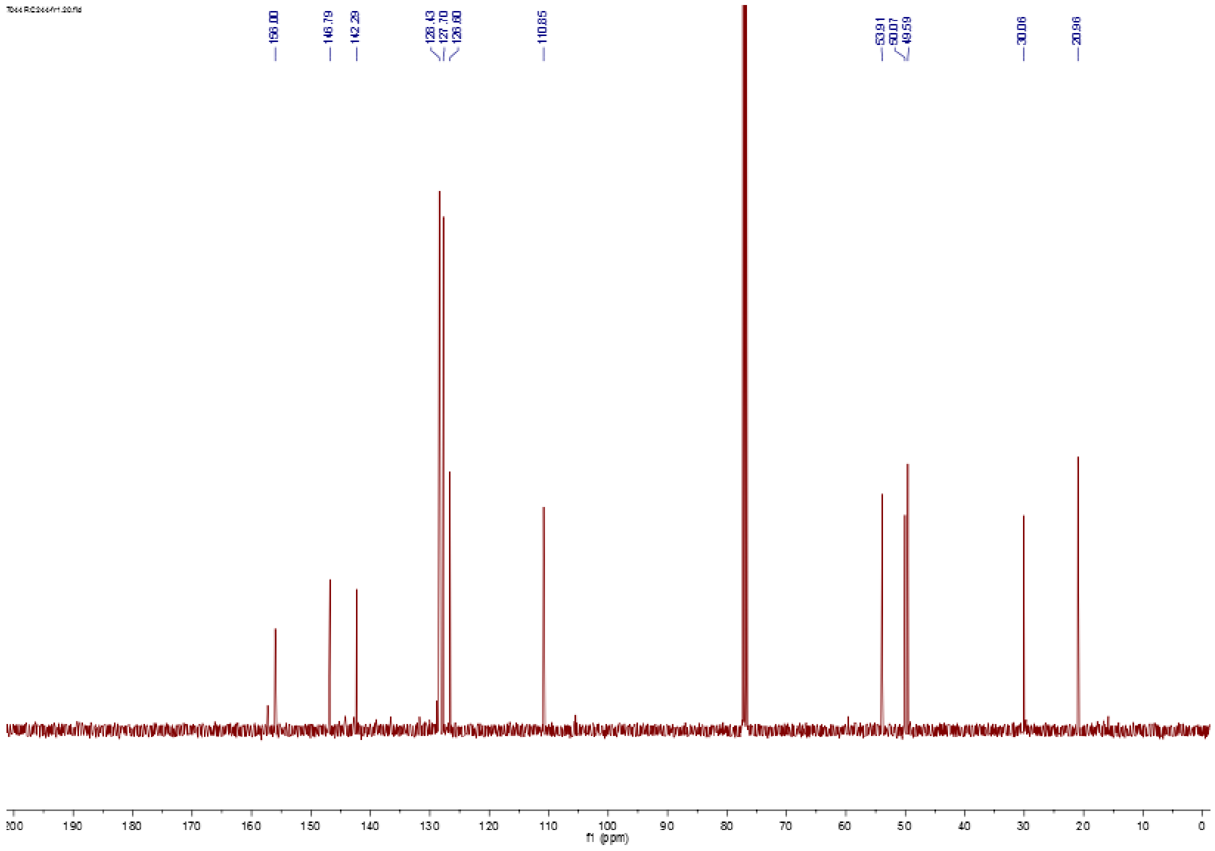

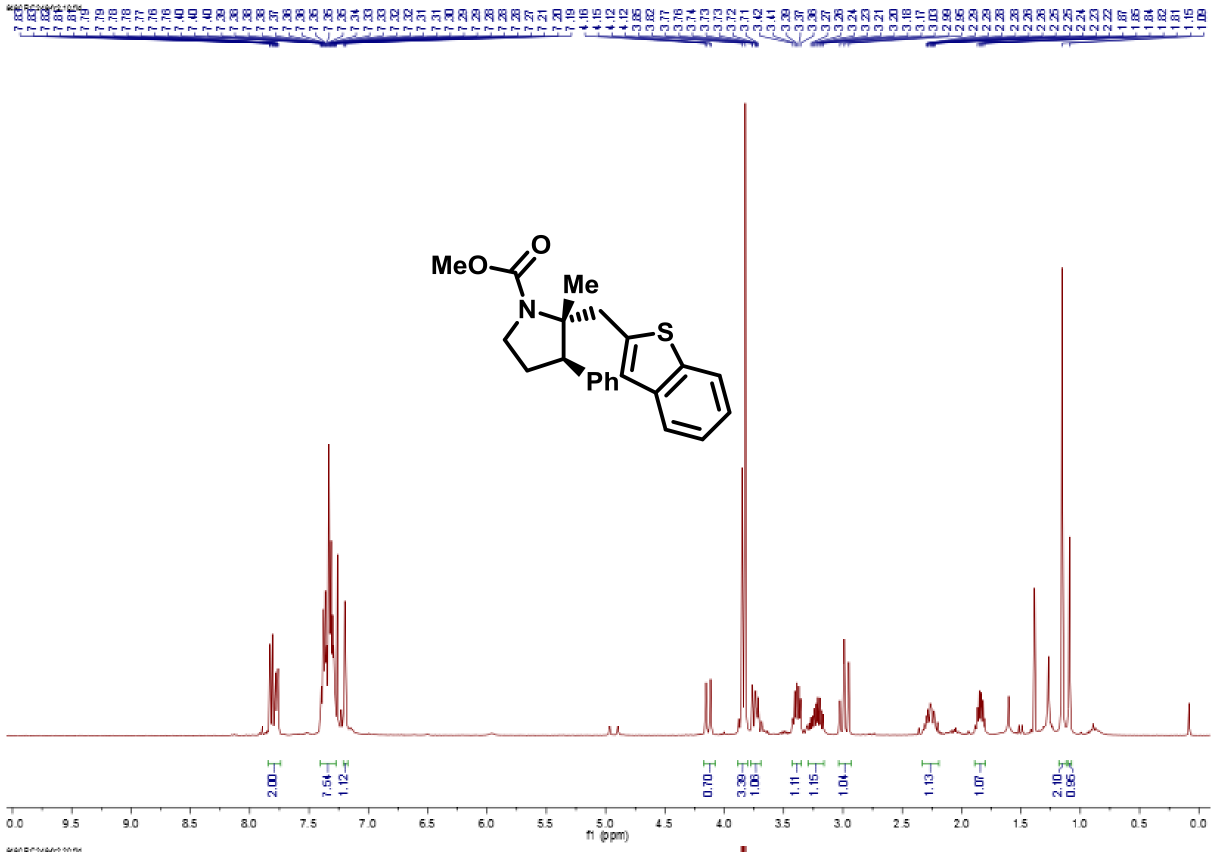

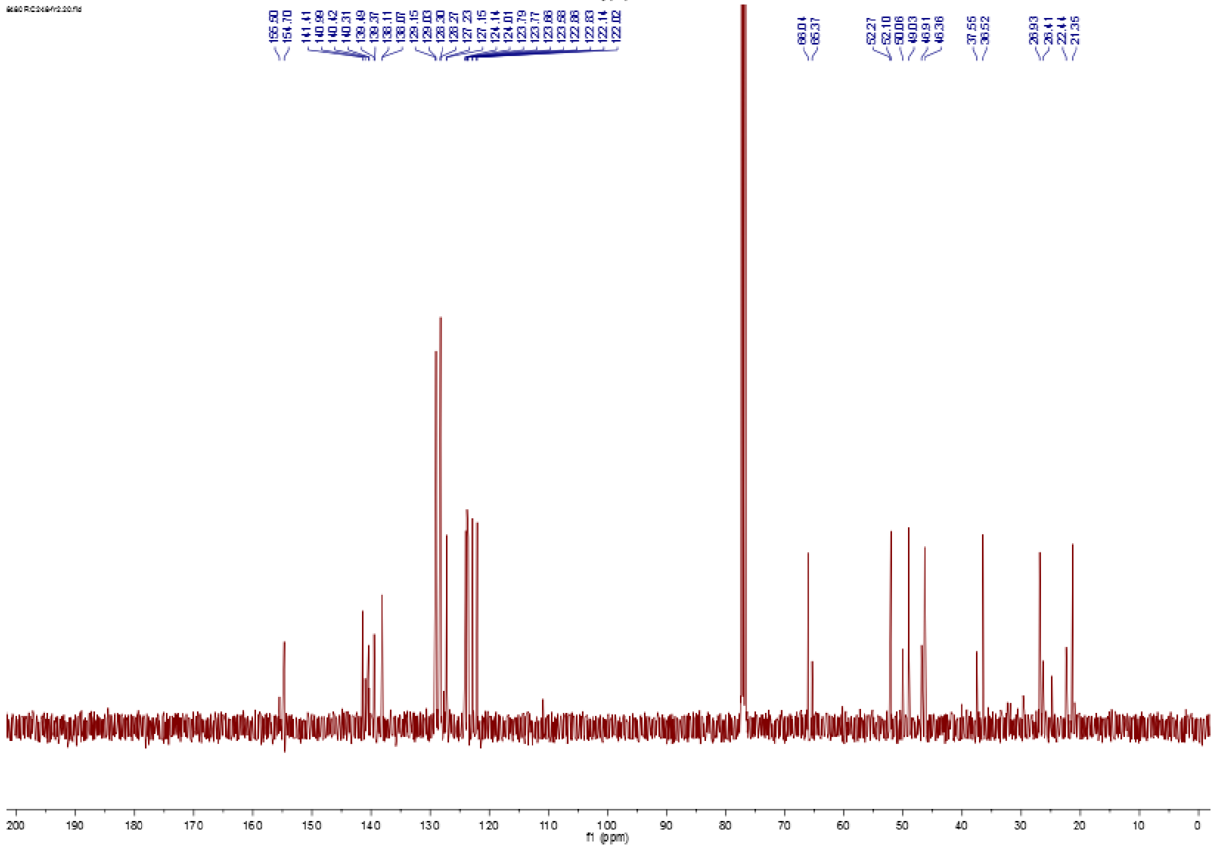

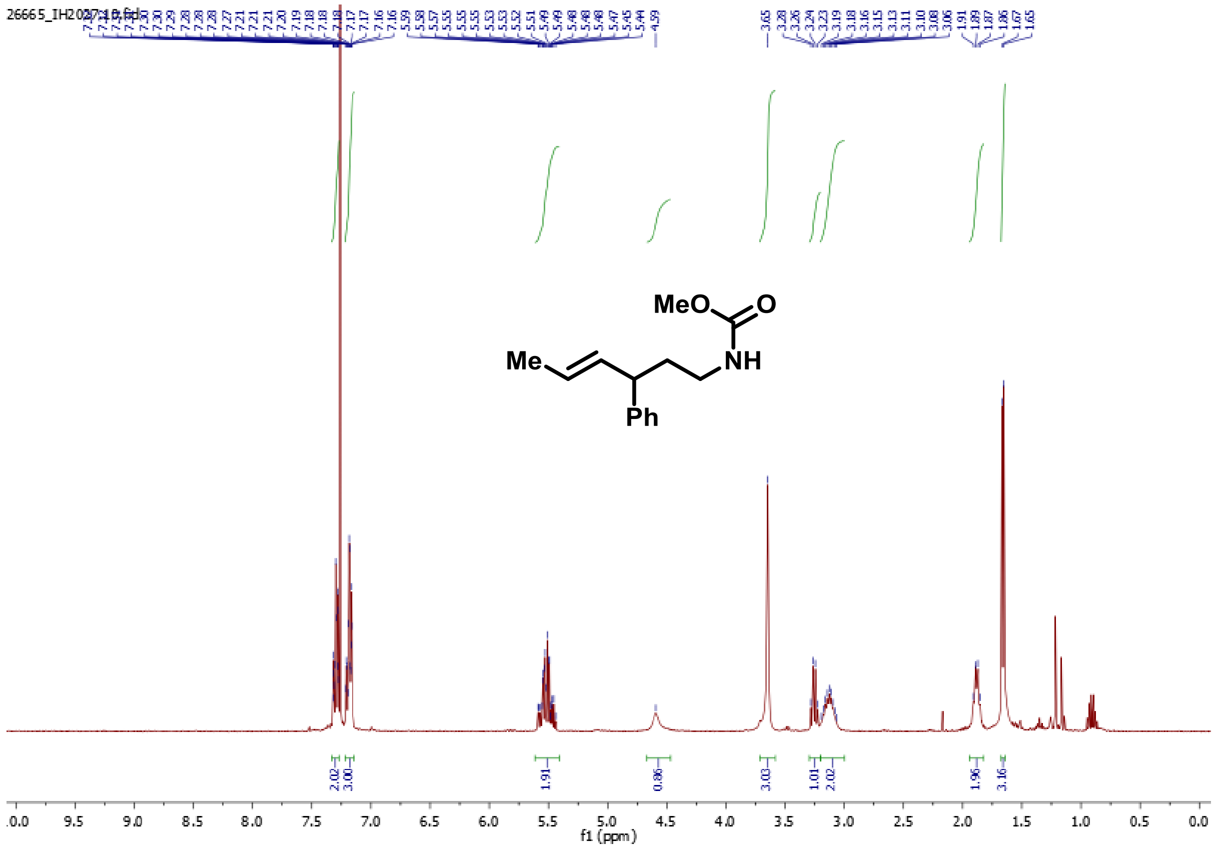
MeO
O
NH
Me
Ph
f1 (ppm)

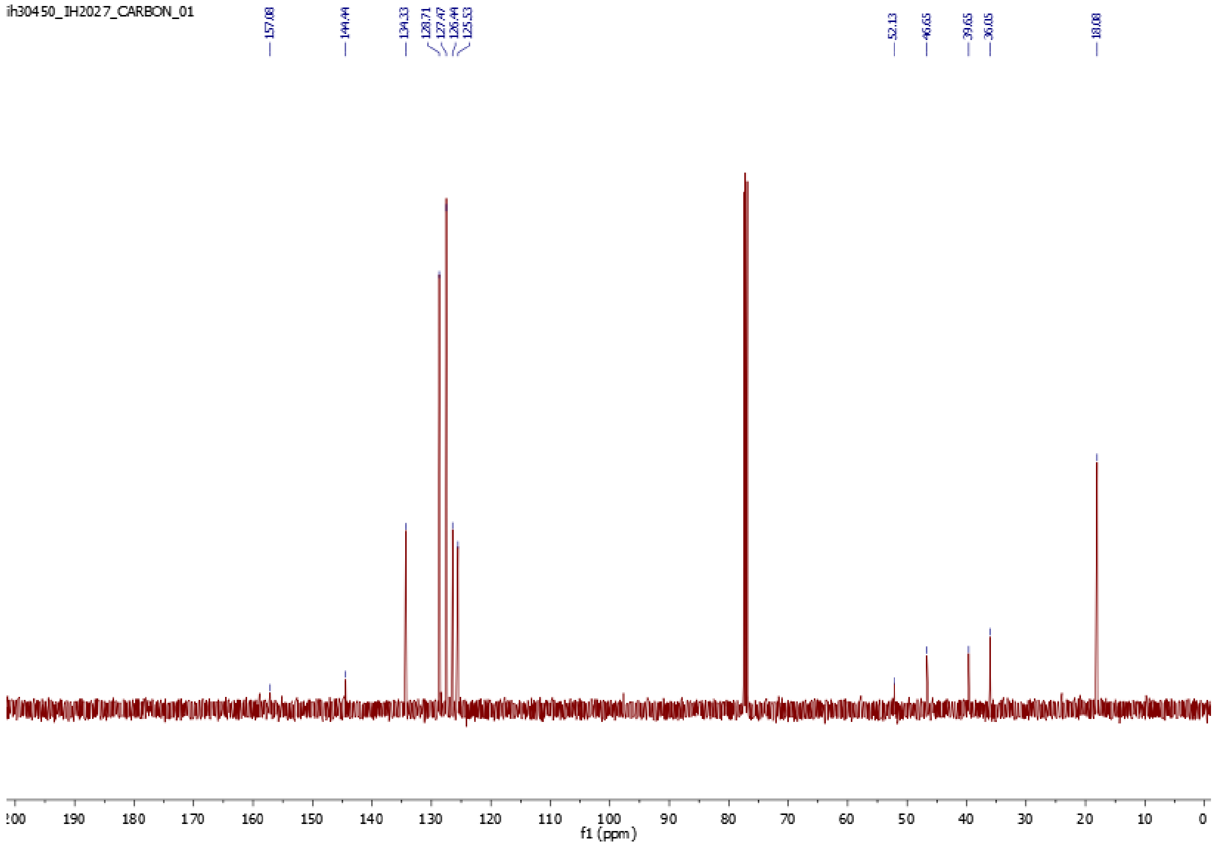
ih30450_IH2027_CARBON_01
157.08
144.44
134.33
128.71
127.47
126.44
125.53
52.13
46.65
39.65
36.05
18.08
f1 (ppm)
